# Tuning Ruthenium Carbene Complexes for Selective P−H Activation through Metal‐Ligand Cooperation

**DOI:** 10.1002/chem.202103151

**Published:** 2021-11-11

**Authors:** Kai‐Stephan Feichtner, Lennart T. Scharf, Thorsten Scherpf, Bert Mallick, Nils Boysen, Viktoria H. Gessner

**Affiliations:** ^1^ Chair of Inorganic Chemistry II Faculty of Chemistry and Biochemistry Ruhr University Bochum Universitätsstrasse 150 44780 Bochum Germany

**Keywords:** bond activations, carbene complexes, metal ligand cooperativity, reaction mechanisms, structure elucidation

## Abstract

The use of iminophosphoryl‐tethered ruthenium carbene complexes to activate secondary phosphine P−H bonds is reported. Complexes of type [(*p‐*cymene)‐RuC(SO_2_Ph)(PPh_2_NR)] (with R = SiMe_3_ or 4‐C_6_H_4_−NO_2_) were found to exhibit different reactivities depending on the electronics of the applied phosphine and the substituent at the iminophosphoryl moiety. Hence, the electron‐rich silyl‐substituted complex undergoes cyclometallation or shift of the imine moiety after cooperative activation of the P−H bond across the M=C linkage, depending on the electronics of the applied phosphine. Deuteration experiments and computational studies proved that cyclometallation is initiated by the activation process at the M=C bond and triggered by the high electron density at the metal in the phosphido intermediates. Consistently, replacement of the trimethylsilyl (TMS) group by the electron‐withdrawing 4‐nitrophenyl substituent allowed the selective cooperative P−H activation to form stable activation products.

## Graphical abstract

Metal ligand cooperation. Two different iminophosphoryl‐substituted methandiide‐derived carbene complexes have been applied in the P−H bond activation of secondary phosphines. Surprisingly versatile reaction outcomes were observed depending on the imine substituent at the methandiide ligand and the phosphine used. While more‐electron‐rich combinations led to either cyclometallation or an imine transfer, electron‐poor combinations enabled selective P−H bond activation, thus impressively demonstrating the importance of tuning the M=C in this type of carbene complex for bond activation.

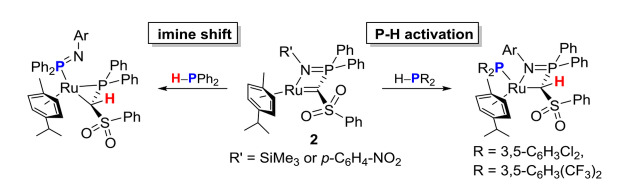

## Introduction

**Figure 1:**
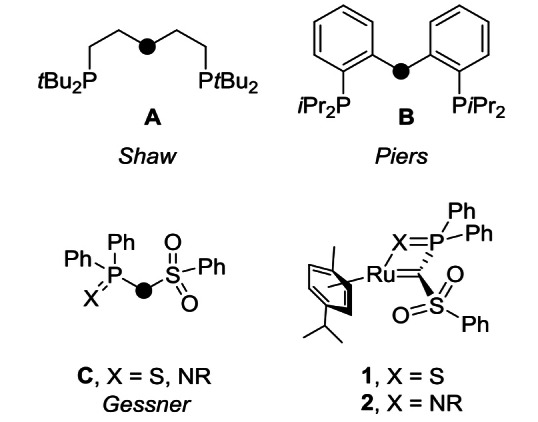
Carbene ligands applied in cooperative bond activation reactions.

Metal ligand cooperation has emerged as a powerful concept in bond activation chemistry and homogeneous catalysis.^[1]^ Here, both the metal and the ligand are directly involved in bond activation processes, whereas in “classical” transition metal catalysis, the ligands act solely as spectators, so that all transformations occur at the metal centre. A variety of different cooperating ligands have been developed over the years and applied in catalysis. While cooperative bond activation reactions through an aromatization/dearomatization mechanism in the ligand have led to remarkable results in the past decade,[^2^, ^3^] most ligands affect substrate activation through the direct participation of a M−L linkage.^[4]^ As such, many activation processes rely on transitions between an amido and amino (M−NR_2_→M−NR_2_H)[^5^, ^6^] and in fewer cases also imido and amido ligand (M=NR→M−N(H)R).^[7]^ Recently, carbene ligands have received renewed research interest in the context of metal ligand cooperation.^[8]^ Here, bond activation reactions involve the direct participation of the M=C bond leading to a transition from a carbene to an alkyl species (M=CR_2_→M−C(H)R_2_). Nonetheless, although the carbon‐centred reactivity of carbene complexes has been known for many years, only few examples are known where the former carbene ligand remains coordinated to the metal and/or where the substrate may also be eliminated (reversible activation) after substrate activation. Early successful bond activation reactions with carbene ligands were achieved with the PCP pincer ligand **A** reported by Shaw (Figure 1).^[9]^ These ligands have been further developed over the years by Ozerov, Piers and others (e. g., **B**),^[10]^ which resulted in a family of complexes that are more stable (due to their “immunity” to β‐hydride elimination), but still highly active in a variety of bond activation reactions.[^9a^, ^11^] For instance, the activation of protic and hydridic E−H bonds as well as H_2_ activation have been previously reported. Even dehydrogenation and catalytic transfer hydrogenation reactions have been realized with these and related pincer ligands[^10c^, ^12^] and most recently also cycloadditions with iron carbene complexes have been disclosed.[^11j^, ^13^]

Our group has particularly focussed on the use of nucleophilic late transition metal carbene complexes based on methandiide ligands such as **C** for cooperative bond activation reactions.^[14]^ The propensity of these complexes to undergo such reactions can be attributed to the special electronics of the M=C linkage.^[15]^ Due to the strongly electron‐withdrawing groups at the carbene carbon atom (which are necessary for the formation of the methandiide precursors) the metal‐carbon bond is considerably polarized towards the carbon end and thus can function as a Brønsted basic site for the cleavage of E−H bonds. This was shown by a series of different activation reactions with substrates with different E−H bond polarities.^[16]^

**Scheme 1:**
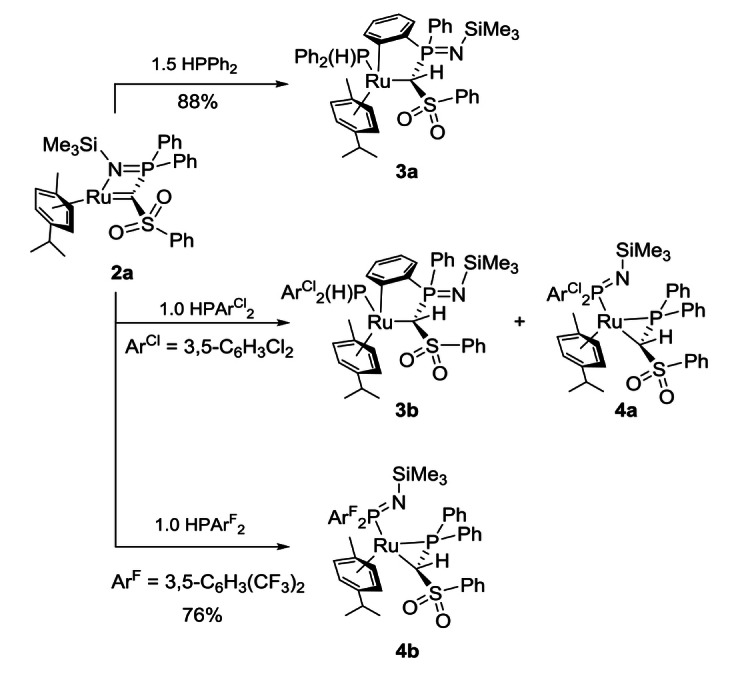
Competing P−H activation reactions with carbene complex **2 a**; cyclometallation and imine shift.

In the course of our research program on methandiide‐derived carbene complexes, we explored P−H bond activation reactions. Our previous studies on cooperative P−H activation reactions using the thiophosphoryl‐tethered ruthenium carbene complex **1** showed only selective transformations in the case of secondary phosphine oxides. In contrast, free phosphines delivered only complex product mixtures. Since bond activation processes across the M=C double bond strongly depend on the nature of the M−C interaction, we turned our attention towards iminophosphoryl‐tethered carbene complexes, which have shown more selective processes due to the less reactive metal carbon linkage. For example, complex **2a** (Scheme 1) showed selective B−H activation reactions across the M=C bond, while multiple transformations were observed for the thiophosphoryl system.^[17]^ Herein, we report P−H bond activation reactions, where higher selectivities were observed with the PNR tether, yet with surprisingly different products being formed depending on the nitrogen substituent.

## Results and Discussion

### P−H activation in phosphines with carbene complex 2 a

**Figure 2:**
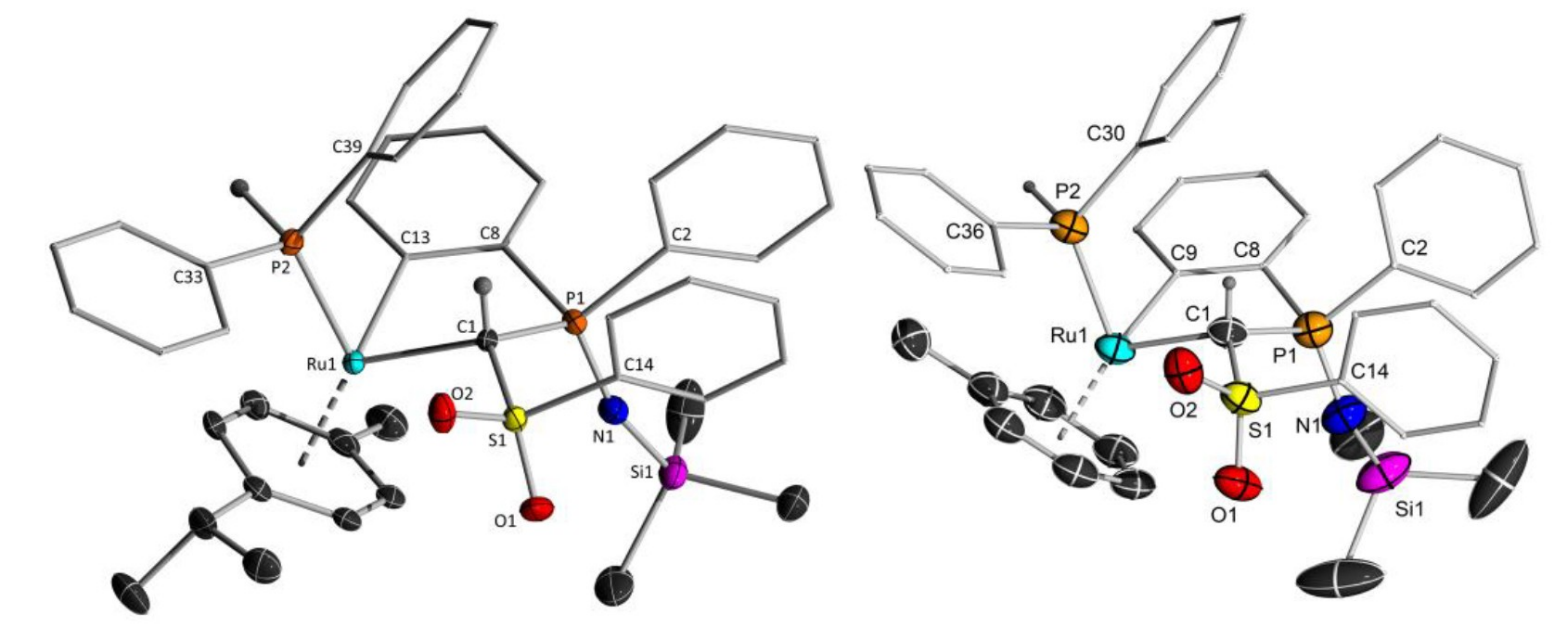
Molecular structures of **3 a** and **6**. Displacement ellipsoids are drawn at the 50 % probability level. All hydrogen atoms except for the methylene bridges and the phosphine moieties have been omitted for clarity. Selected bond lengths [Å] and angles [°]: **3 a**: C1−Ru1 2.181(2), C1−P1 1.813(2), C1−S1 1.7560(19), S1−O1 1.4421(17), S1−O2 1.4461(17), S1−C14 1.775(2), P1−N1 1.5366(19), P1−C2 1.831(2), P1−C8 1.804(2), Ru1−C13 2.074(2), Ru1−P2 2.2848(5), S1−C1−P1 117.52(10), Ru1−C1−P1 107.29(10), P1−N1−Si1 156.82(14). **6**: Ru1−C1 2.174(2), C1−S1 1.770(2), S1−O1 1.4457(16), S1−O2 1.4511(16), S1−C14 1.770(2), C1−P1 1.817(2), P1−N1 1.5455(19), P1−C8 1.803(2), P1−C2 1.825(2), N1−Si1 1.6633(19), Ru1−P2 2.2785(5), S1−C1−P1 115.81(11), P1−N1−Si1 146.89(14).

Initially, the reactivity of carbene complex **2 a** towards phosphines was first probed by means of diphenylphosphine Ph_2_PH. Treatment of the carbene complex with a slight excess of the phosphine at room temperature delivered over the course of one day a single new product as evidenced by ^31^P{^1^H} NMR spectroscopy (Scheme 1). However, the product was identified as the cyclometallated species **3 a** and not as the simple P−H activation product (similar cyclometallations have been observed before^[18]^). **3 a** is characterized by two doublets at *δ*_P_=13.9 ppm and *δ*_P_=38.8 ppm with a coupling constant of ^3^ *J*_PP_=3.8 Hz. The methylene moiety gives rise to a doublet of doublets in the ^1^H NMR (*δ*_H_=3.11 ppm; ^2^ *J*_HP_=6.5 Hz and ^3^ *J*_HP_=6.5 Hz) and ^13^C{^1^H} NMR spectra (*δ*_C_=53.1 ppm; ^1^ *J*_CP_=61.2 Hz and ^2^ *J*_CP_=9.3 Hz), while the H atom bound at the phosphorus atom appears as doublet at 6.95 ppm with a large coupling constant of ^1^ *J*_PH_=365.0 Hz. Recording of a ^1^H coupled ^31^P NMR spectrum clearly confirmed the P−H unit (Figure S1 in the Supporting Information). Single crystals of **3 a** were obtained by diffusion of pentane into a diethyl ether solution of the complex. XRD analysis unambiguously confirmed the nature of the new species (Figure 2).^[19]^ **3 a** crystallizes in the orthorhombic space group *Pbca*. In contrast to the carbene complex, the iminophosphoryl moiety in **3 a** does not coordinate to the ruthenium centre anymore due to the additional coordination of the Ph_2_PH ligand. Thus, the methanide ligand solely coordinates via the two carbanionic sites. The Ru−C1 bond length amounts to 2.181(2) Å and is thus considerably longer than in the starting carbene complex (1.955(2) Å) and within the range of a typical Ru−C single bond.^[20]^ The Ru−P bond length is slightly shorter compared with other ruthenium phosphine complexes and the P1−N1−Si1 angle is larger than the average angle reported.[^21^, ^22^] It is noteworthy that the cyclometallation proceeds in a diastereoselective manner. Only the isomer with the phosphine ligand and the hydrogen atom at the methylene bridge attached on the same side of the former double bond is found in the molecular structure. Also in solution, no evidence for the formation of a further isomer was observed.

**Scheme 2:**
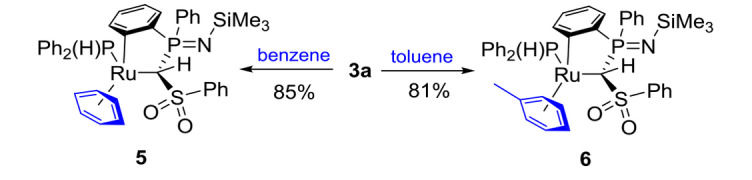
Arene exchange at the cyclometallated complex **3 a**.

Surprisingly, complex **3 a** is revealed to be unstable in solutions of benzene and C_6_D_6_. Monitoring the reaction process by ^31^P{^1^H} NMR spectroscopy revealed the continuous and selective formation of a single new species with similar NMR features compared to the starting material (*δ*_P_=14.7 and 40.2 ppm; ^3^ *J*_PP_=3.9 Hz). Along with this complex, the formation of free cymene was observed, thus suggesting replacement of the cymene ligand by benzene to form complex **5** (Scheme 2). A similar reaction was observed with toluene as solvent. Both complexes **5** and **6** could be isolated in high yields. Crystal structure analysis of **6** unambiguously confirmed the exchange of the arene ligand in **3 a** (Figure 2).

The facile replacement of the cymene ligand in **3 a** can most likely be attributed to steric reasons. The exchange of the sterically demanding cymene moiety with toluene or benzene reduces steric strain around the ruthenium centre, thus resulting in a faster reaction for the smaller benzene. As such, replacement of the cymene ligand at room temperatures requires 5 days for benzene, whereas 7 days are needed for toluene. Heating at 50 °C reduces the reaction time to 24 h for the toluene complex. Electronic effects seem to be less important, since the reaction with even more electron‐poor arenes such as trifluoromethylbenzene led to no conversion. Overall, the facile replacement of the cymene ligand in **3 a** is quite interesting. While arene‐exchange is well documented in ruthenium chemistry, most examples refer to cationic bis(arene) or naphthalene complexes^[23]^ and/or the replacement of the arene by a more electron‐rich arene at elevated temperatures.^[24]^ In contrast, facile arene exchange at neutral complexes has been observed less often, particularly at room temperature.^[25]^

**Figure 3:**
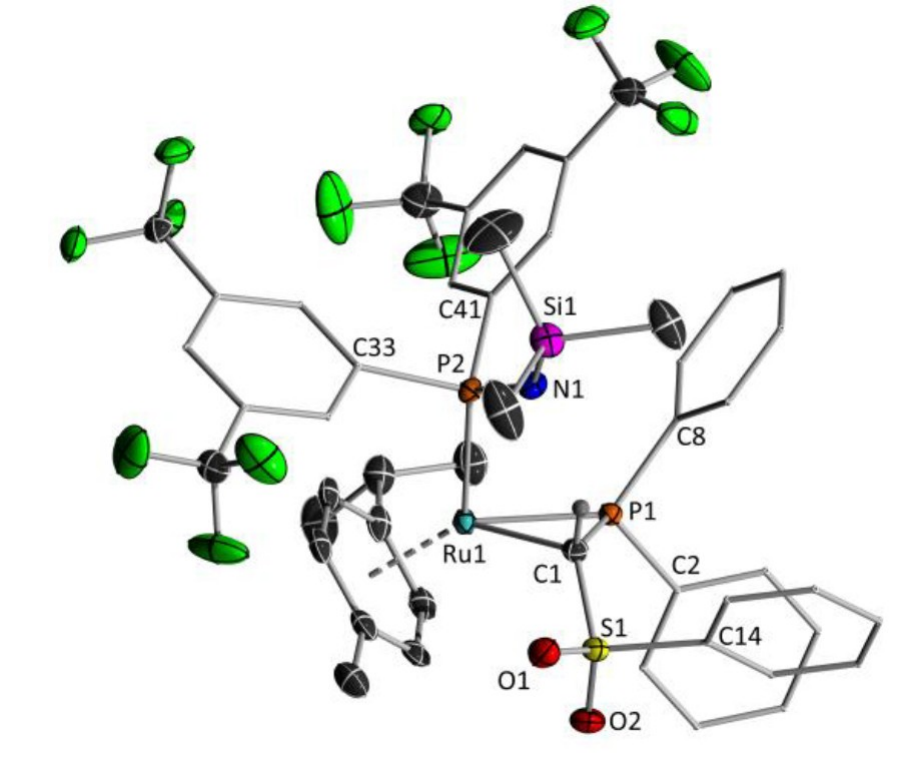
Molecular structure of **4 b**. Displacement ellipsoids are drawn at the 50 % probability level. All hydrogen atoms except for the methylene bridge have been omitted for clarity. Selected bond lengths [Å] and angles [°]: Ru1−C1 2.1618(16), Ru1−P1 2.2585(4), Ru1−P2 2.3856(4), C1−P1 1.7691(17), C1−S1 1.7295(16), P1−C8 1.8068(17), P1−C2 1.8237(17), S1−C14 1.7785(18), S1−O1 1.4428(13), S1−O2 1.4472(13), P2−N1 1.5689(14), S1−C1−P1 127.28(10), P2−N1−Si1 144.29(10).

Next, the reaction of carbene complex **2 a** with the less electron rich bis(3,5‐dichlorophenyl)phosphine (=HPAr${}^{\mathrm{Cl}{}_{2}}$ , Scheme 1) was tested. Monitoring of the reaction immediately after the start by means of ^31^P{^1^H} NMR spectroscopy revealed (besides as‐yet‐unreacted starting materials) the formation of three compounds (Figure S31, bottom). One compound showed two doublets in the ^31^P{^1^H} NMR spectrum at 32.5 and 47.0 ppm with a coupling constant of 5.0 Hz and revealed itself to be an intermediate of the reaction as the signals completely vanished during prolonged reaction times. The two remaining products exhibited a ratio of approximately 1:0.7, with the main product showing similar signals as the cyclometallated compound **3 a** (two doublets at *δ*_P_=13.9 and 41.7 ppm with a coupling constant of ^3^ *J*_PP_=5.1 Hz). Using ^1^H coupled ^31^P NMR spectroscopy, it was possible to identify the major product as the cyclometallated complex **3 b** (Figure S31, top). The second product however featured vastly different signals in the ^31^P{^1^H} NMR spectrum, namely two doublets at *δ*_P_=−13.2 and 35.6 ppm with a surprisingly large coupling constant of 47.0 Hz. Unfortunately, we could not identify the second compound at first due to the difficulties in the separation of both products. However, we assumed that the reaction with an even more electron poor phosphine would shift the reaction outcome completely to the new product. Therefore, **2 a** was reacted with di(3,5‐bis(trifluoromethyl)phenyl) phosphine (=HPAr${}^{F{}_{2}}$ ). This led to the selective formation of a single new product **4 b** which is characterized in the ^31^P{^1^H} NMR spectrum by two doublets at *δ*_P_=−13.4 and 34.5 ppm with a coupling constant of ^2^ *J*_PP_=47.5 Hz and a doublet in the ^1^H NMR spectrum at 4.93 ppm (^2^ *J*_HP_=15.4 Hz) for the methylene bridge. **4 b** could be characterized by NMR and XRD (Figure 3) as well as elemental analysis which revealed it to be the product of a P−H bond activation followed by a shift of the imino moiety resulting in the formation of a *κ* ^2^‐P,C‐bound phosphinomethanide ligand (Scheme 1). This also explains the large coupling constants in the ^31^P{^1^H} NMR spectra for **4 a** as well as **4 b. 4 b** crystallizes in the monoclinic space group *P*2_1_/*n*. The former carbene ligand now coordinates to the ruthenium centre via the methanide carbon atom and the phosphorous atom, thus forming a Ru1−C1−P1 three‐membered ring. Such *κ* ^2^‐P,C‐bound phosphinomethanide complexes have been described for various metals, albeit the number with unsupported ligands (without a further donor) is still rare.^[26]^ The Ru1−P1 bond length is shorter compared to simple ruthenium phosphine complexes,^[22]^ but similar to other *κ* ^2^‐phosphinomethanide complexes.^[26]^

**Scheme 3:**
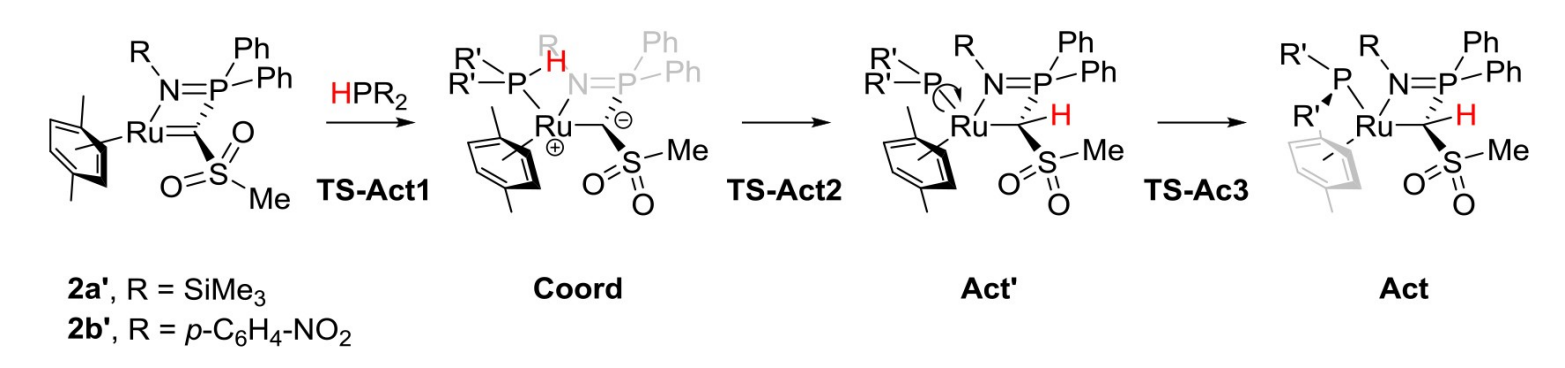
Calculated pathway for the activation of secondary phosphines.

In order to better understand the reasons for the different reactivities of **2 a** towards the different phosphines we performed computational studies at the PBE0‐D3/def2tzvp level of theory (Scheme 3). Model systems with *p*‐xylene instead of *p*‐cymene and a methyl group at the sulfonyl moiety were used to reduce the computational costs and to prevent the calculation of multiple possible conformers. Since no cyclometallation has been observed before with carbene complex **2 a**, we assumed that formation of **3** –as well as of **4**– is initiated in a P−H bond activation step by addition across the Ru=C bond to form intermediate **Act** (Scheme 3) This would also be in line with the observation of an intermediate compound with two coupling phosphorous atoms in the reaction of **2 a** with HPAr${}^{\mathrm{Cl}{}_{2}}$ (see above). Calculations revealed that the activation process proceeds via initial coordination of the corresponding phosphine to form the coordination complex **Coord**, followed by a proton transfer (**TS‐Act2**) to generate intermediate **Act’**. Rotation around the P−Ru bond finally gives the activation product **Act** observed in experiment. Since the P−H activation could also proceed via a concerted 1,2‐addition or an oxidative addition at the metal centre followed by proton transfer, these two pathways have also been investigated exemplarily for the combination **2a**′/HPPh_2_. The energy of the transition state for the concerted 1,2‐addition across the Ru=C linkage (**Conc**, Table 1) revealed to be significantly higher than all barriers calculated for the stepwise pathway shown in Scheme 3. The same holds true for the oxidative addition complex (**OxAdd**, Table 1) which features an η^2^ coordinated cymene ligand to reduce steric strain. These findings further confirm the proposed pathway for the activation process.

**Table 1:** Free energies for the activation pathway [PBE0‐D3/def2tzvp+LANL2TZ(f) for Ru] with **2a** and HPPh_2_. Free energies are given relative to **2a’** in kJ/mol.

	Δ*G* ^≠^		Δ*G* ^≠^
**2 a’**	0	**TS‐Act1**	37
**Coord**	−29	**TS‐Act2**	34
**Act’**	−53	**TS‐Act3**	−47
**Act**	−73	**Conc**	78
**OxAdd**	80

**Figure 4:**
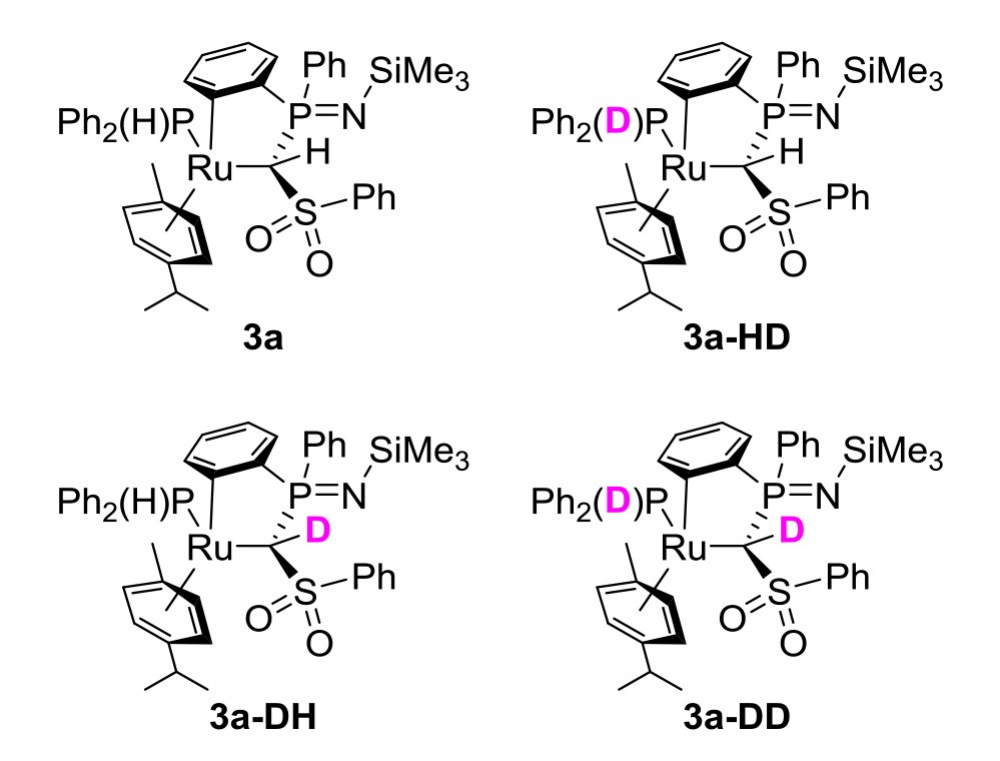
Products of the reaction of carbene complex 2a with DPPh_2_.

To experimentally prove that orthometallation proceeds via P−H bond activation, we performed deuteration experiments. To this end, **2 a** was reacted with one equivalent of Ph_2_PD. In case of a cooperative P−H/D activation, a deuterium should be placed at the methylene bridge of **3 a** after the activation process (**3 a‐DH**, Figure 4). Monitoring of the reaction via ^31^P{^1^H} NMR spectroscopy revealed three signals at *δ*_P_=13.8–13.9 (multiplet), 37.8 (triplet, ^2^ *J*_PD_=54.9 Hz) and 38.8–38.9 (multiplet) ppm (Figure S28). These findings can be explained by differently deuterated structures (Figure 4). The triplet at *δ*_P_=37.8 ppm originates from a compound with a deuterated phosphine bound to ruthenium (**3 a‐HD** or **3 a‐DD**). This is at first in contradiction to the assumed P−H activation across the Ru=C bond which suggests the formation of **3 a‐DH**. However, the presence of species like **3 a‐HD** or **3 a‐DD** in the reaction mixture can be explained by the exchange of the phosphine ligand in **3 a‐DH** with unreacted DPPh_2_ which, due to the overall slow reaction process, gives the double deuterated compound **3 a‐DD**. This additionally leads to the formation of free HPPh_2_ which can then be activated by still unreacted carbene complex leading to **3 a**. Here, the ruthenium bound phosphine can again be replaced by DPPh_2_ to afford **3 a‐HD**. This also explains the presence of a signal for the proton of the methylene bridge in the ^1^H NMR spectrum, but with an integral of only 0.5. An additionally performed ^2^H NMR spectrum (Figure S29) further confirms the presence of deuterium at the phosphine moiety and the carbon bridge. Presumably, all four compounds shown in Figure 4 are present in the reaction mixture. To further confirm this explanation and the exchange of phosphine ligands in the cyclometallated complex, complex **3 a** was reacted with DPPh_2_. Indeed, ^31^P{^1^H} NMR spectroscopy showed the formation of the aforementioned triplet at *δ*_P_=37.8 ppm (and free HPPh_2_; Figure S30). Furthermore, the signals of **3 a** in the ^31^P{^1^H} NMR spectrum and the integral of the methylene bound proton in the ^1^H NMR spectrum remained completely unaffected, suggesting that no H/D exchange at the methanide ligand takes place. This clearly confirms that the deuteration of the carbene centre must be the result of the P−H activation across the Ru−C bond (to **Act**; Scheme 3).

**Scheme 4:**
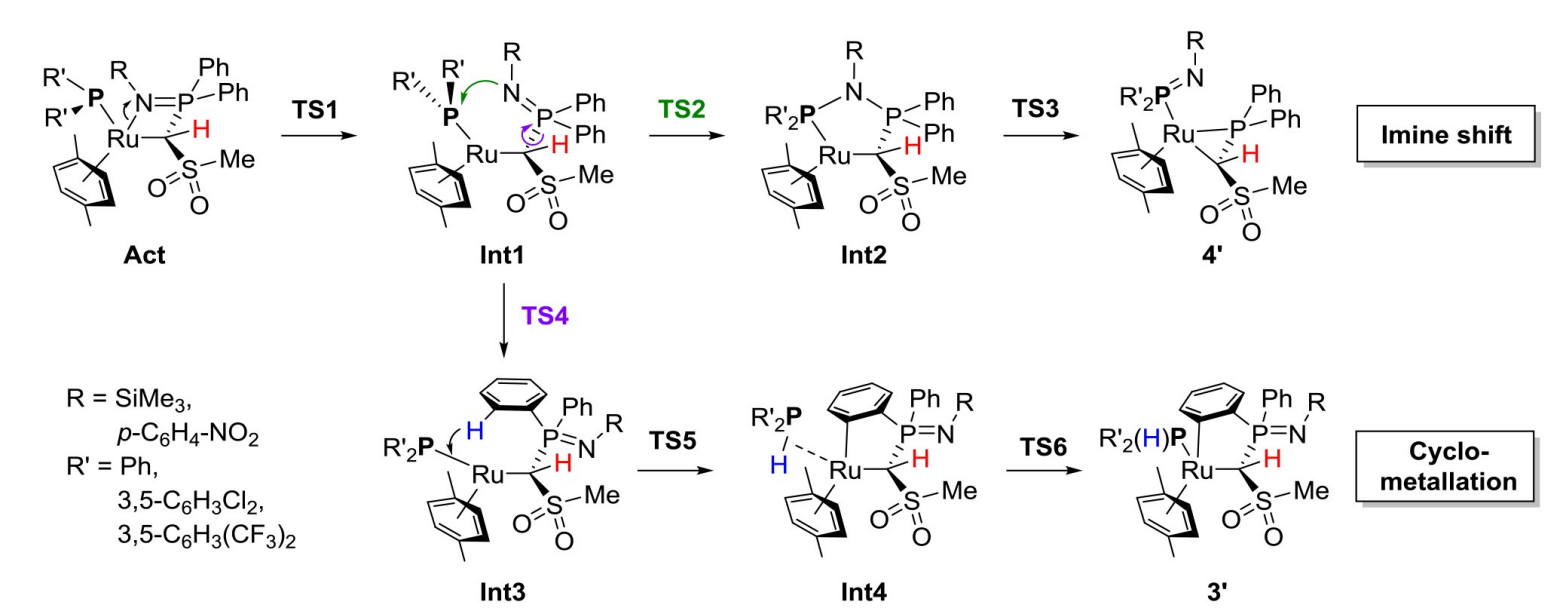
Calculated pathways for the cyclometallation and the imine shift starting from the P−H activation intermediate **Act**.

Having confirmed the possible formation of **Act** as first reaction intermediate, the next steps for the cyclometallation to **3** and imine shift to **4** were evaluated by DFT methods (Scheme 4). The first step after P−H activation for both reaction pathways is the de‐coordination of the iminophosphoryl moiety to form **Int1** with a free coordination site at ruthenium. It is important to note that this de‐coordination is supported by a change in the bonding between ruthenium and the phosphido ligand. Upon de‐coordination the phosphido moiety planarizes which suggests a partial Ru−P double bond character, as evidenced by the shortening of the Ru−P bond from 2.429 Å in **Act** to 2.193 Å in **Int1** (for R = TMS and R’ = Ph). From this point, the two reaction pathways diverge. For the imine shift to **4**’, the iminophosphoryl moiety attacks at the ruthenium bound phosphorous atom (**TS2**) thus forming a five‐membered Ru−P−N−P−C ring in **Int2**. Now the original P−N bond is cleaved (**TS3**) giving the final product of the imine shift (**4’**).

For the orthometallation, the iminophosphoryl group in **Int1** rotates about the P−C1 bond, so that the ortho C−H bond of one of the phenyl rings coordinates to the unsaturated ruthenium centre. This agostic interaction leads to a pyramidalization of the phosphido moiety in **Int3**. From this intermediate, a direct σ bond metathesis between the Ru−P and the C−H bond is energetically disfavoured. An oxidative addition of the C−H bond to the ruthenium centre as observed for other orthometallation reactions also does not take place.[^16^, ^27^] Instead, the proton directly inserts into the Ru−P bond to give **Int4** with an *η*_2_‐coordinated P−H bond. From here, the formed secondary phosphine changes its coordination mode to the typical Lewis base coordination which results in product **3**’.

Both pathways have been calculated for the TMS‐substituted system with the three phosphines HPPh_2_, PHAr^Cl^_2_ and HPAr^F^_2_. An overview of the respective energies is given in Table 2. The highest barrier ΔG≠max along the reaction pathway determines the selectivity of the reaction. ΔG≠max is defined by the energy difference between **Act** (which is the intermediate with the lowest energy for all complexes) and the highest transition state of the two reaction pathways. In the case of the imine shift **TS2** exhibits the highest barrier, while for the cyclometallation **TS4** required the highest energy. These two steps are compared in Table 3.

**Table 2:** Free energies for the orthometallation and the imine shift [PBE0‐D3/def2tzvp+LANL2TZ(f) for Ru] (free energies are given relative to Act in kJ/mol).

					Imine shift				Orthometallation					
R	R’	**Act**	**TS1**	**Int1**	**TS2**	**Int2**	**TS3**	**4**	**TS4**	**Int3**	**TS5**	**Int4**	**TS6**	**3**
SiMe_3_	Ph	0	45	40	80	36	73	−35	83	59	73	61	79	−46
SiMe_3_	3,5‐C_6_H_4_Cl_2_	0	57	51	89	45	81	−37	94	57	75	69	94	−31
SiMe_3_	3,5‐C_6_H_4_(CF_3_)_2_	0	–^[a]^	59	93	44	84	−36	96	54	74	70	99	−19
*p*‐C_6_H_4_−NO_2_	Ph	0	58	50	86	41	65	−36	103	80	89	76	95	−37
*p*‐C_6_H_4_−NO_2_	3,5‐C_6_H_4_Cl_2_	0	–^[a]^	65	99	49	81	−28	117	81	94	86	113	−17
*p*‐C_6_H_4_−NO_2_	3,5‐C_6_H_4_(CF_3_)_2_	0	–^[a]^	69	101	46	86	−25	117	78	92	83	116	−6

[a] Structure not found.

**Table 3:** ΔG≠max values in kJ/mol for both reaction pathways.

R	R’	ΔG≠max	ΔG≠max	Isolated
		cyclomet.	imine shift	product
SiMe3	Ph	83	80	cyclomet.
SiMe3	3,5‐C6H4Cl2	94	89	cyclomet and imine shift
SiMe3	3,5‐C6H4(CF3)2	99[a]	93	imine shift
p‐C6H4NO2	Ph	103	86	imine shift
p‐C6H4NO2	3,5‐C6H4Cl2	117	99	activation
p‐C6H4NO2	3,5‐C6H4(CF3)2	117	101	activation

[a] **TS6** used instead of **TS4**.

Overall, the ΔG≠max values reflect the experimental observations. For the TMS‐substituted carbene complex **2 a** both pathways are viable and exhibit barriers that can be overcome at room temperature. The barriers are slightly lower for the experimentally observed compounds, except for the combination of **2a**′ with HPPh_2_. Here, the cyclometallation product is formed despite its slightly higher activation barrier. This might also be explained from a thermodynamic perspective, since only in case of Ph_2_PH is the cyclometallation product thermodynamically more stable than the product of the imine shift. Furthermore, the computational results show that gradually reducing the electron density of the phosphine shifts the reaction outcome from the cyclometallation to the imine shift and most importantly results in an increase of the activation energies, thus suggesting that a further reduction of the electron density at ruthenium might lead to further increased reaction barriers and hence a stable P−H activation product.

### Carbene complex design for selective P−H activation

**Scheme 5:**
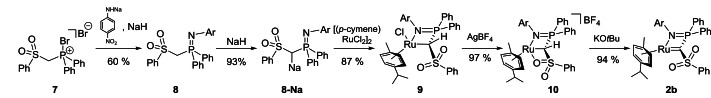
Synthesis of carbene complex **2 b** (Ar=4‐NO_2_−C_6_H_4_).

**Figure 5:**
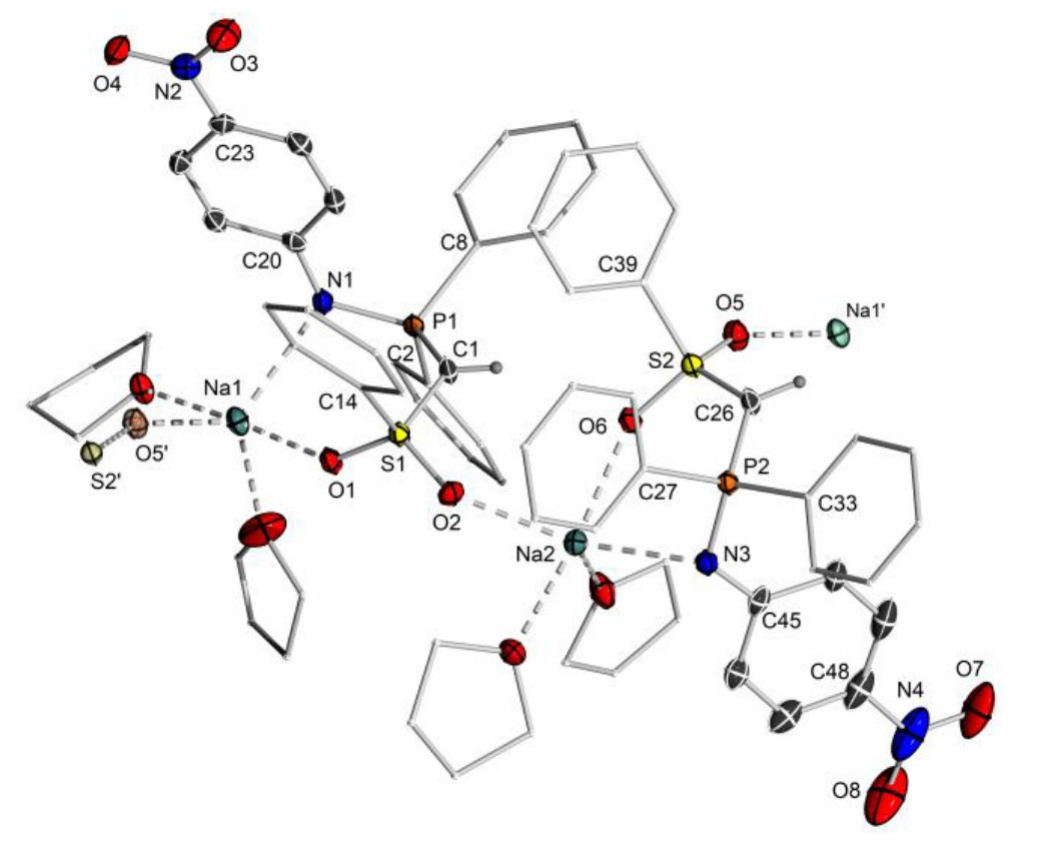
Molecular structure of **8**‐Na. Displacement ellipsoids are drawn at the 50 % probability level. All hydrogen atoms except for the methylene bridge have been omitted for clarity. Selected bond lengths [Å] and angles [°]: P1−C1 1.710(3), S1−C1 1.658(2), P1−N1 1.620(2), P1−C2 1.814(2), P1−C8 1.819(2), S1−O1 1.456(2), S1−O2 1.454(2), O1−Na1 2.332(2), N1−Na1 2.540(2), S1−C14 1.787(2), Na1 O5‘ 2.308(2), P2−C26 1.711(2), S2−C26 1.655(2), P2−N3 1.615(2), P2−C27 1.823(2), P2−C33 1.822(2), S2−O5 1.450(2), S2−O6 1.459(2), S2−C39 1.783(3), O6−Na2 2.349(2), O2−Na2 2.311(2), P1−C1−S1 124.4(2), P1−N1−C20 121.7(2), P2−C26−S2 122.3(2), P2−N3−C45 122.01(2).

**Figure 6:**
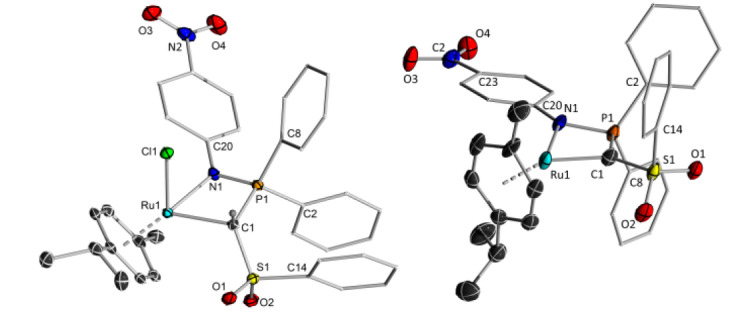
Molecular structures of complexes **9** (left) and **2 b** (right). Displacement ellipsoids are drawn at the 50 % probability level. All hydrogen atoms except for the methylene bridge in **9** have been omitted for clarity. Selected bond lengths [Å] and angles [°]: **9**: C1−S1 1.752(2), C1−P1 1.783(2), C1−Ru1 2.197(2), P1−N1 1.613(2), P1−C2 1.812(2), P1−C8 1.806(2), N1−C20 1.395(2), N1−Ru1 2.148(2), S1−O1 1.446(2), S1−O2 1.441(2), S1−C14 1.769(2), Ru1−Cl1 2.434(1), S1−C1−P1 122.6(1), P1−N1−C20 128.9(2).

In order to enhance the selectivity of the P−H activation process and the stability of the simple activation products we addressed the synthesis of a less electron‐rich carbene complex. We chose complex **2 b** (Scheme 5) with the electron‐withdrawing nitrophenyl group at the iminophosphoryl moiety as target complex. Due to the electron‐withdrawing nitro group, the ruthenium centre was supposed to be less electron‐rich so that cyclometallation or imine‐shift should be more difficult than in case of the silyl analogue **2 a**. Carbene **2 b** was prepared according to a stepwise protocol as outlined in Scheme 5. At first, the protonated ligand **8** was synthesized as yellow solid in 60 % yield by amination of the corresponding phosphine bromide **7** with *p*‐nitroaniline. Deprotonation with sodium hydride delivered methanide **8‐Na** in excellent yields of 93 %. Both compounds were characterized by NMR spectroscopy, elemental analysis as well as X‐ray crystallography (Figures S36 and 5). The sodium salt forms a polymeric structure in the solid state (triclinic space group *P*‐1). The asymmetric unit contains two methanide molecules which are connected by the sodium cations. Both metal cations feature a trigonal‐bipyramidal geometry due to coordination of the iminophosphoryl and sulfonyl groups as well as two THF molecules. Each sulfonyl moiety binds to two sodium atoms thus resulting in the polymeric nature of the compound. No contacts between the planar methanide carbon atoms and the metals are observed. The P−C and S−C bond lengths in **8‐Na** experience the typical shortening upon deprotonation due to the stronger electrostatic interactions within the P−C−S linkage compared to the protonated precursor **8** (Figure 5). For example, the C−S bond shortens from 1.777(2) Å in **8** to 1.658(2) Å (for S1−C1) in **8**‐Na. Since dimetallation of **8** proved to be difficult, carbene complex **2 b** was directly synthesized from methanide **8**‐Na. Treatment of the sodium salt with half an equivalent of [(*p*‐cymene)RuCl_2_]_2_ delivered the chloro complex **9** in 87 % yield. Compound **9** is characterized by a singlet in the ^31^P{^1^H} NMR spectrum at *δ*_P_=50.9 ppm and by doublets at *δ*=4.00 (^2^ *J*_HP_=7.4 Hz) and 34.1 ppm (^1^ *J*_CP_=64.4 Hz) in the ^1^H and ^13^C{^1^H} NMR spectrum, respectively. In the molecular structure (orthorhombic space group *Pbca*, Figure 6), the chloro complex exhibits the expected *P,N*‐coordination of the methanide ligand to the ruthenium centre. The P−N bond of 1.613(2) Å is slightly longer than in the related TMS‐substituted system (1.585(2) Å)),^[20]^ which is well in line with the electron‐withdrawing ability of the nitrophenyl moiety. Dehydrohalogenation of **9** to carbene complex **2 b** was selectively achieved with potassium *tert*‐butoxide. Treatment with 1 equiv. of base gave complex **2 b** as green solid. The ^31^P{^1^H} NMR signal of the carbene complex appears at *δ*_P_=65.3 ppm and the signal of the carbenic carbon atom at *δ*_C_=143.6 ppm (^1^ *J*_CP_=66.6 Hz), thus being in line with the formation of a carbene species. Unfortunately, separation of the carbene complex from the formed NaCl proved to be difficult, probably due to the coordination of the salt to the carbene complex.

To circumvent the formation of potassium chloride, halide abstraction from **9** with silver tetrafluoroborate was carried out, leading to the formation of the cationic ruthenium complex **10** in nearly quantitative yield. Complex **10** shows a very broad signal in the ^31^P{^1^H} NMR spectrum at 41.3 ppm. This is probably due to fluctional behaviour of the weakly coordinating sulfonyl moiety. The proton of the methylene bridge exhibits a doublet in the ^1^H NMR spectrum at *δ*_H_=4.50 ppm with a coupling constant of ^2^ *J*_HP_=3.7 ppm and the methylene carbon atom appears in the ^13^C{^1^H} carbon NMR spectrum at *δ*_C_=21.8 ppm. Subsequent deprotonation with potassium *tert*‐butoxide gave the desired carbene complex in 94 % yield and NaBF_4_ as by‐product which could be successfully removed by filtration. By slowly diffusing pentane in a saturated solution of **2 b** in DCM crystals suitable for XRD analysis could be obtained. Carbene complex **2 b** crystallizes in monoclinic space group *Pn* (Figure 6, right). Unfortunately, due to the highly disordered nature of the crystal structure, errors in bond lengths and angles are too high for quantitative discussion.

**Scheme 6:**
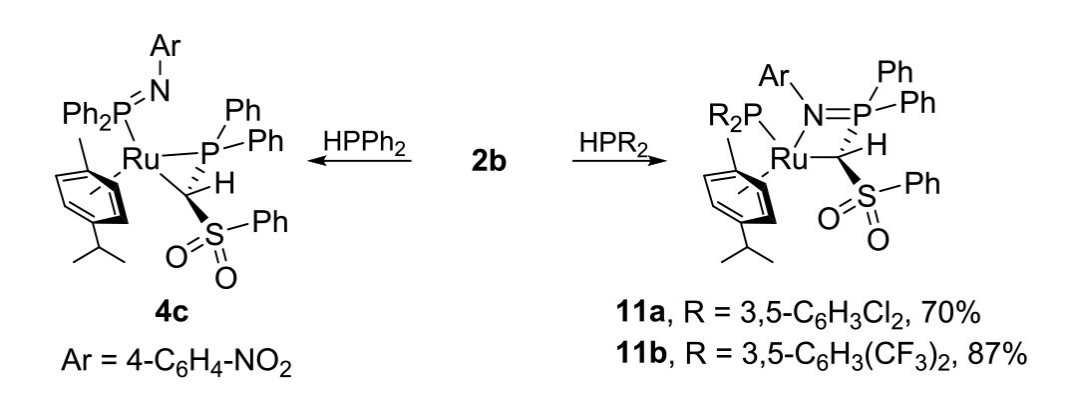
P−H bond activation reactions with carbene complex **2 b**.

Having carbene complex **2 b** in hand, its applicability in cooperative P−H bond activation reactions was examined. In line with our assumption, the reaction of **2 b** with HPPh_2_ selectively delivered complex **4 c**, that is, the product of the above discussed imine shift reaction (Scheme 6). No cyclometallation product could be observed. **4 c** shows two doublets in the ^31^P{^1^H} NMR spectrum at *δ*_P_=−14.7 and 35.7 ppm with a coupling constant of ^2^ *J*_PP_=44.3 Hz which is well in line with the observed NMR signals for the complexes **4 a** and **4 b**.

**Figure 7:**
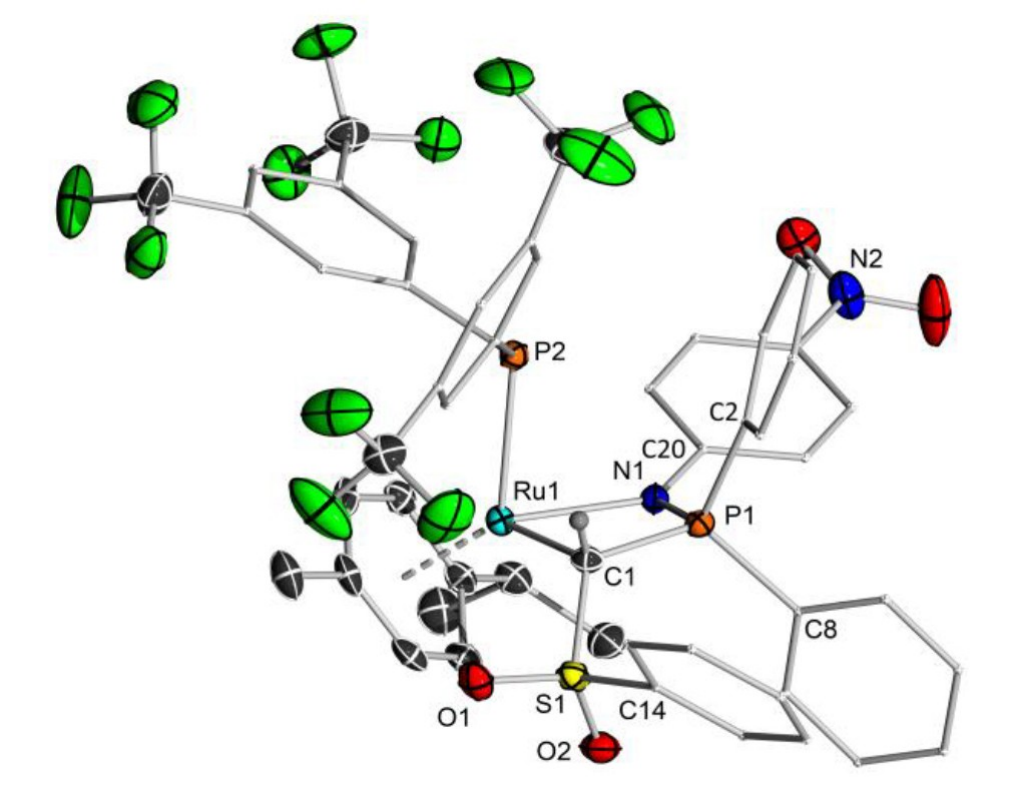
Molecular structure of **11 b**. Displacement ellipsoids are drawn at the 50 % probability level. All hydrogen atoms except for the methylene bridge have been omitted for clarity. Selected bond lengths [Å] and angles [°]: C1−Ru1 2.201(2), C1−P1 1.793(2), C1−S1 1.750(2), P1−N1 1.6198(18), N1−Ru1 2.1739(17), Ru1−P2 2.4136(6), S1−O1 1.4453(17), S1−O2 1.4398(17), S1−C14 1.774(2), P1−C8 1.810(2), P1−C2 1.807(2), N1−C20 1.389(3), S1−C1−P1 120.70(12), P1−N1−C20 125.50(15).

To our delight, the reactions of **2 b** with the electron poor phosphines HPAr^Cl^_2_ and HPAr^F^_2_ eventually and selectively delivered the desired activation products **11**. The activation products are characterized by two doublets in the ^31^P{^1^H} NMR spectrum at *δ*_P_=23.2 and 50.1 ppm (^3^ *J*_PP_=2.6 Hz) for **11 a** and 19.1 (broad singlet) and 49.8 ppm (doublet with ^3^ *J*_PP_=3.8 Hz) for **11 b** respectively. Both compounds were characterized by multinuclear NMR spectroscopy and CHN analyses. By slowly diffusing pentane into a saturated solution of **11 b** in toluene it was possible to obtain single crystals suitable for XRD analysis which unambiguously confirmed the nature of the activation products (Figure 7). **11 b** crystallizes in the monoclinic space group *P*2_1_/*c*. The phosphide moiety and the proton are attached on the same side of the former Ru−C. However, this cannot exclude the possible formation of an *anti* isomer during the reaction. Since the imine moiety is only weakly bound to the ruthenium centre, de‐coordination is feasible, followed by a rotation around the Ru−C bond. Calculations on the full systems at the PBE0‐D3/def2tzvp level of theory revealed **11 b** (*syn* isomer) to be 29 kJ/mol lower in energy than the corresponding *anti* isomer. This high energetic preference suggests that the *syn* isomer is also thermodynamically favoured und is thus in line with the experimental observation.

The above‐mentioned mechanisms for cyclometallation and imine shift have also been calculated for carbene complex **2 b** in combination with the three applied phosphines (see Tables 1 and 2 for free energies and ΔG≠max values). The ΔG≠max values reflect the observed reactivities very well. Overall, the activation energies increase for both mechanisms for **2 b** in comparison to **2 a**. For diphenylphosphine, the barrier in case of the imine shift amounts to 86 kJ/mol and is thus (in contrast to the cyclometallation) still viable at room temperature. However, for the two electron poor phosphines HPAr^Cl^_2_ and HPAr^F^_2_ both pathways show considerably higher values for ΔG≠max (99–117 kJ/mol) which explains why the reactions nearly stop after the P−H activation step which enables the isolation of the “true” activation products. In fact, ^31^P{^1^H} NMR experiments of a NMR sample of **11 a** after 4 days in solution showed slow decomposition of the substance to (besides several minor side products) the corresponding orthometallated and imine shift products (Figure S32) thus furthermore validating that both reactions proceed via the activation pathway (Scheme 4).

## Conclusion

In summary, we have examined the applicability of iminophosphoryl‐tethered ruthenium carbene complexes in cooperative P−H bond activation reactions. Both complexes underwent addition of the P−H bond across the M=C double bond. However, depending on the phosphine used and/or the nature of the imino moiety, the activation process was followed by cyclometallation to form complexes with a coordinating secondary phosphine or by an imine shift to yield *κ* ^2^‐P,C‐bound phosphinomethanide complexes. Gradually reducing the electron density at ruthenium changed the reaction outcome from cyclometallation to the product of the imine shift. DFT studies confirmed this tendency and showed that a more electron‐poor ruthenium centre should also allow the isolation of stable P−H activation products immune to cyclometallation and imine transfer. This was proven by using the electron‐poor nitroaniline‐substituted carbene complex **2 b** in combination with phosphines with electron‐withdrawing groups. This eventually led to the desired activation products **10**, which could be isolated at room temperature. This diverse reactivity impressively demonstrates the tunability of the electronics of the M=C linkage in methandiide‐derived carbene complexes and its importance for cooperative E−H bond activations. The results obtained are of particular importance for the design of further carbene complexes for bond activation. Here, the tuning of the M=C bond will be decisive for the development of reversible transformations that also allow transfer of the activated substrates and hence catalytic applications.

## Conflict of interest

The authors declare no conflict of interest.

## Supporting information

As a service to our authors and readers, this journal provides supporting information supplied by the authors. Such materials are peer reviewed and may be re‐organized for online delivery, but are not copy‐edited or typeset. Technical support issues arising from supporting information (other than missing files) should be addressed to the authors.

Supporting Information
