# Supplementary material for: Tuning Ruthenium Carbene Complexes for Selective P−H Activation through Metal‐Ligand Cooperation
Source: Chemistry. 2021 Nov 11;27(69):17351–60. doi: 10.1002/chem.202103151 (PMC9299219; doi:10.1002/chem.202103151)
Supplement: Supplementary file 1 — Supporting Information [file CHEM-27-17351-s001.pdf]

Chemistry–A European Journal

Supporting Information

Tuning Ruthenium Carbene Complexes for Selective P–H Activation through Metal-Ligand Cooperation

Kai-Stephan Feichtner, Lennart T. Scharf, Thorsten Scherpf, Bert Mallick, Nils Boysen, and Viktoria H. Gessner*

Index

1. Experimental details 3
2. ^{1}H NMR, ^{13}C NMR, and ^{31}P NMR Spectra 10
3. Crystal structure determination 36
3.1 General 36
3.2 Crystal Structure Determination of Compound 3b 39
3.3 Crystal Structure Determination of Compound 6 42
3.4 Crystal Structure Determination of Compound 4b 46
3.5 Crystal Structure Determination of Compound 8 50
3.6 Crystal Structure Determination of Compound 8-Na 53
3.7 Crystal Structure Determination of Compound 8 59
3.8 Crystal Structure Determination of Compound 2b 62
3.9 Crystal Structure Determination of Compound 11b 69
4. Computational studies 74
4.1 General information 74
4.2 Energies and coordinates of the calculated structures and transition states 74
4.2.1 Starting compounds 74
4.2.2 2a’ + $HPPh_2$ 80
4.2.3 2a’ + $HPAr^{Cl}_2$ 120
4.2.4 2a’ + $HPAr^{F}_2$ 160
4.2.5 2b’ + $HPPh_2$ 203
4.2.6 2b’ + $HPAr^{Cl}_2$ 243
4.2.7 2b’ + $HPAr^{F}_2$ 279
4.2.8 Conc and OxAdd 319
4.2.9 Optimizations of the full structures of 11b and 11b-anti 324
5. References 331

1. Experimental details

General conditions. All experiments were carried out under a dry, oxygen-free argon atmosphere using standard Schlenk techniques. Involved solvents were dried over sodium, potassium or P_4O_{10} and distilled prior to use or with a MBraun SPS-800. H_2O is distilled water. ^1H, $^{13}C\{^1H\}$, $^{29}Si\{^1H\}$, $^{31}P\{^1H\}$, ^{31}P NMR spectra were recorded on Avance-500, Avance-III-400, Avance-300 or DPX-250 spectrometers at 22 °C if not stated otherwise. All values of the chemical shift are in ppm regarding the δ-scale. All spin-spin coupling constants (*J*) are printed in Hertz (Hz). To display multiplicities and signal forms correctly the following abbreviations were used: s = singlet, d = doublet, t = triplet, q = quartet, sept = septet, dd = doublet of doublets m = multiplet, br = broad signal. Signal assignment was supported by APT, HMQC, HMBC and COSY experiments. For NMR spectra see the Supporting Information. Elemental analyses were performed on an Elementar Vario MICRO-cube elemental analyzer. All reagents were purchased from Sigma-Aldrich, ABCR, Acros Organics or TCI Chemicals and used without further purification. Phosphine bromide **7**[1], deuterated diphenylphosphine[2] bis(3,5-dichlorophenyl)phosphine and di(3,5-bis(trifluoromethyl)phenyl)phosphine were synthesized according to literature.[3]

Synthesis of complex 3a. 300 mg (454 µmol) of carbene complex **2a** were dissolved in 8 mL toluene. 127 mg (681 µmol) diphenyl phosphine were added at RT and the reaction mixture stirred for 16 hours leading to a brown solution. The solvent was removed in vacuo and the yellow residue washed five times with pentane (3 mL) giving compound **3a** as a pale yellow solid. (340 mg, 401 µmol, 88 %).

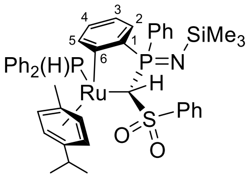

3a

^{1}H-NMR: (400.3 MHz, d^8-THF): δ = 0.13 (s, 9H; Si(CH_3)$_3$), 0.83 (d, $^3J_{HH}$ =6.9 Hz, 2H; CH(CH_3)$_2$), 1.21 (d, $^3J_{HH}$ = 6.7 Hz, 2H; CH(CH_3)$_2$), 1.62 (s, 3H; CCH_3), 1.90 (sept, $^3J_{HH}$ = 7.3 Hz, 1H; CH(CH$_3$)$_2$), 3.11 (dd, $^2J_{HP}$ = 6.5 Hz, $^3J_{HP}$ = 6.5 Hz, 1H; SCHP), 5.28 (br s, 1H; CH_{Cym}), 5.64 (d, $^3J_{HH}$ = 6.0 Hz, 1H; CH_{Cym}), 5.94 (br s, 1H; CH-2), 6.18 (d, $^3J_{HH}$ = 5.8 Hz, 1H; CH_{Cym}), 6.25-6.30 (m, 1H; C$H_{P(N)Ph,para}$), 6.39 (d, $^3J_{HH}$ = 5.9 Hz, 1H; CH_{Cym}), 6.54-6.57 (m, 2H; C$H_{P(N)Ph,ortho}$), 6.62-6.66 (m, 1H; CH-3), 6.81-6.86 (m, 3H; C$H_{SPh,meta}$ + C$H_{P(H)Ph,para}$), 6.95 (d, $^1J_{HP}$ = 365.0 Hz, 1H; PH), 7.00-7.03 (m, 2H; C$H_{SPh,para}$ + C$H_{P(H)Ph,para}$), 7.21-7.25 (m, 2H; C$H_{P(H)Ph,meta}$), 7.32-7.36 (m, 1H; CH-4), 7.38-7.45 (m, 8H; C$H_{P(H)Ph,meta,ortho}$ + C$H_{SPh,ortho}$ +C$H_{P(N)Ph,meta}$), 7.85 (m, 1H; CH-5), 8.05-8.09 (m, 2H; C$H_{P(H)Ph,ortho}$). **^{13}C{^{1}H}-APT-NMR:** (100.7 MHz, d^8-THF): δ = 5.53 (d, $^3J_{CP}$ = 2.1 Hz; Si(CH$_3$)$_3$), 18.1 (CCH$_3$), 21.4 (CH(CH$_3$)$_2$), 25.2 (CH(CH$_3$)$_2$), 31.2 (CH(CH$_3$)$_2$), 53.1 (dd, $^1J_{CP}$ = 61.2 Hz, $^2J_{CP}$ = 9.3 Hz; SCHP), 87.6 (d, $^2J_{CP}$ = 1.3 Hz; CH$_{Cym}$), 90.9 (d, $^2J_{CP}$ = 7.6 Hz; CH$_{Cym}$), 91.7 (CH$_{Cym}$), 92.7 (CH$_{Cym}$), 108.7 (d, $^2J_{CP}$ = 6.2 Hz; C_{Cym}), 119.4 (C_{Cym}), 122.9 (d, $^3J_{CP}$ = 12.3 Hz; CH-3), 127.3 (d, $^2J_{CP}$ = 11.1 Hz; CH$_{P(N)Ph,ortho}$), 128.3 (CH$_{SPh,meta}$), 128.7 (d, $^4J_{CP}$ = 2.8 Hz; CH$_{P(H)PPh,para}$), 128.8 (d, $^4J_{CP}$ = 2.5 Hz; CH$_{P(H)Ph,para}$), 128.9 (CH$_{SPh,ortho}$), 129.5 (d, $^3J_{CP}$ = 9.1 Hz; CH$_{P(H)Ph,meta}$), 129.6 (d, $^3J_{CP}$ = 8.7 Hz; CH$_{P(H)Ph,meta}$), 130.5 (CH$_{P(N)Ph,para}$), 130.6 (CH-4), 131.1 (d, $^3J_{CP}$ = 1.4 Hz; CH$_{P(N)Ph,meta}$), 131.7 (CH$_{SPh,para}$), 132.1 (d, $^2J_{CP}$ = 10.3 Hz; CH-2), 132.5 (d, $^1J_{CP}$ = 41.7 Hz; CH$_{P(H)Ph,ipso}$), 134.0 (d, $^2J_{CP}$ = 8.1 Hz; CH$_{P(H)Ph,ortho}$), 134.6 (d, $^2J_{CP}$ = 9.2 Hz; CH$_{P(H)Ph,ortho}$), 134.9 (d, $^1J_{CP}$ = 41.3 Hz, CH$_{P(H)Ph,ipso}$), 139.7 (d, $^1J_{CP}$ = 81.9 Hz; CH$_{P(N)Ph,ipso}$), 132.2 (dd, $^3J_{CP}$ =10.1 Hz, $^3J_{CP}$ = 15.0 Hz; CH-5), 145.4 ($C_{SPh,ipso}$), 154.2 (d, $^1J_{CP}$ = 124.1 Hz; C-1), 170.5 (dd, $^2J_{CP}$ = 26.9 Hz, $^2J_{CP}$ = 23.3 Hz; C-6). **^{31}P{^{1}H}-NMR:** (162.1 MHz, d^8-THF): δ = 13.9 (d, $^3J_{PP}$ = 3.8 Hz; P(N)Ph$_2$), 38.8 (d, $^3J_{PP}$ = 3.8 Hz; P(H)Ph$_2$). Anal. calcd. for $C_{44}H_{50}NO_2P_2RuSSi$: C, 62.39; H, 5.83; N, 1.65; S, 3.78. Found: C, 62.05; H, 5.83; N, 1.85; S, 3.49.

Synthesis of compound 4b. 100 mg (151 µmol) of carbene complex **2a** were dissolved in 3 mL toluene and the resulting solution cooled to –78 °C. 69.3 mg (151 µmol) of di(3,5-bis(trifluoromethyl)phenyl)phosphine were dissolved in 2 mL of toluene and the solution slowly added to the carbene solution. The reaction mixture was slowly warmed to room temperature and stirred for 16h. The solvent was removed under reduced pressure and 20 mL hexane were added. The resulting mixture was filtered and stored for 2 days at –80 °C. The supernatant solvent was removed and the resulting brown solid dried *in vacuo* to give compound **4b** in 76% (115 µmol, 129 mg) yield.

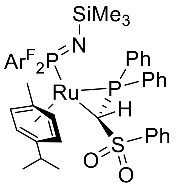

4b

^{1}H-NMR: (400.3 MHz, d^8-THF): δ = -0.07 (s, 9H; Si(C*H*$_3$)$_3$), 1.28 (d, $^3J_{HH}$ = 6.9 Hz, 3H; CH(C*H*$_3$)$_2$), 1.33 (d, $^3J_{HH}$ = 6.8 Hz, 3H; CH(C*H*$_3$)$_2$), 2.66 (s, 3H; CC*H*$_3$), 3.01 (sept, $^3J_{HH}$ = 6.1 Hz, 1H; C*H*(CH$_3$)$_2$), 4.93 (d, $^2J_{HP}$ = 15.4 Hz, 1H; SC*H*P), 4.97 (br s, 1H; C*H*$_{Cym}$), 5.15 (br s, 1H; C*H*$_{Cym}$), 5.74 (br d, $^3J_{HH}$ = 5.3 Hz, 1H; C*H*$_{Cym}$), 5.82 (br s, 1H; C*H*$_{Cym}$), 6.53 (dd, $^3J_{HH}$ = 12.5 Hz, $^3J_{HH}$ = 7.6 Hz, 2H; C*H*$_{PPh,ortho}$), 6.67-6.71 (m, 2H; CH$_{PPh,meta}$), 7.01 (t, $^3J_{HH}$ = 7.4 Hz, 1H; CH$_{PPh,para}$), 7.13 (dd, $^3J_{HP}$ = 13.0 Hz, $^3J_{HH}$ = 8.0 Hz, 2H; C*H*$_{PPh,ortho}$), 7.29-7.35 (m, 4H; C*H*$_{SPh,meta}$ + C*H*$_{PPh,meta}$), 7.37-7.39 (m, 2H; C*H*$_{SPh,ortho}$), 7.44-7.49 (m, 2H; C*H*$_{PPh,para}$ + C*H*$_{SPh,para}$), 7.77 (br s, 2H; P(N)CC*H*), 7.85 (s, 1H; CF$_3$CC*H*CCF$_3$), 7.92-7.94 (m, 3H; P(N)CC*H* + CF$_3$CC*H*CCF$_3$). **^{13}C{^{1}H}-APT-NMR:** (100.7 MHz, d^8-THF): δ = 4.73 (d, $^3J_{CP}$ = 2.2 Hz; Si(*C*H$_3$)$_3$), 20.4 (C*C*H$_3$), 23.7 (CH(*C*H$_3$)$_2$), 25.9 (CH(*C*H$_3$)$_2$), 32.5 (*C*H(CH$_3$)$_2$), 39.2 (dd, $^1J_{CP}$ = 19.0 Hz, $^2J_{CP}$ = 4.7 Hz; S*C*HP), 89.5 (d, $^2J_{CP}$ = 3.4 Hz; *C*H$_{Cym}$), 89.8 (d, $^2J_{CP}$ = 3.4 Hz; *C*H$_{Cym}$), 90.2 (d, $^2J_{CP}$ = 4.4 Hz; *C*H$_{Cym}$), 96.2 (br; *C*H$_{Cym}$), 103.8 (*C*$_{Cym}$), 115.9 (*C*$_{Cym}$), 122.5 (br; CF$_3$CCHCCF$_3$), 123.2 (br, CF$_3$C*C*HCCF$_3$), 124.4 (q, $^1J_{CF}$ = 272.7 Hz; *C*F$_3$), 124.6 (q, $^1J_{FC}$ = 272.3 Hz; *C*F$_3$), 125.4 (dd, $^1J_{CP}$ = 55.2 Hz, $^3J_{CP}$ = 4.7 Hz; P(N)*C*), 127.3 (*C*H$_{SPh,ortho}$), 128.1 (d, $^3J_{CP}$ = 12.2 Hz; *C*H$_{PPh,meta}$), 128.6 (d, $^3J_{CP}$ = 12.2 Hz; *C*H$_{PPh,meta}$), 129.2 (*C*H$_{SPh,meta}$), 129.6 (d, $^1J_{CP}$ = 50.5 Hz; P(N)*C*), 130.4 (br, P(N)C*C*H), 130.7 (q, $^2J_{FC}$ = 32.7 Hz; F$_3$C*C*), 130.8 (q, $^2J_{FC}$ = 32.8 Hz; F$_3$C*C*), 130.9 (d, $^4J_{CP}$ = 2.7 Hz; *C*H$_{PPh,para}$), 131.5 (br d, $^2J_{CP}$ = 11.9 Hz; P(N)C*C*H), 131.8 (d, $^4J_{CP}$ = 3.0 Hz; *C*H$_{PPh,para}$), 132.5 (*C*H$_{SPh,para}$), 134.4 (d, $^2J_{CP}$ = 11.4 Hz; *C*H$_{PPh,ortho}$), 136.3 (d, $^2J_{CP}$ = 11.8 Hz; *C*H$_{PPh,ortho}$), 146.3 (*C*$_{SPh,ipso}$), 150.1 (dd, $^1J_{CP}$ = 40.3 Hz, $^3J_{CP}$ = 3.3 Hz; *C*$_{PPh,ipso}$), 154.4 (d, $^1J_{CP}$ = 26.5 Hz; *C*$_{PPh,ipso}$). **^{31}P{^{1}H}-NMR:** (162.1 MHz, d^8-THF): δ = -13.4 (d, $^2J_{PP}$ = 47.5 Hz; *P*(N)), 34.5 (d, $^2J_{PP}$ = 47.5 Hz; *P*(Ru)). Anal. calcd. for $C_{48}H_{45}F_{12}NO_2P_2RuSSi$: C, 51.52; H, 4.05; N, 1.25; S, 2.86. Found: C, 52.08; H, 4.33; N, 1.11; S, 2.78.

Synthesis of complex 5. 100 mg (118 µmol) of compound **3a** were dissolved in 5 mL benzene and the reaction mixture stirred for 24 h at 50 °C. The solvent was removed under reduced pressure and the residue was washed three times with pentane (5 mL) affording compound **5** as a pale yellow solid. (93.4 mg, 100 µmol, 85 %).

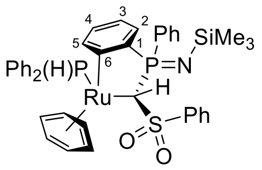

5

^{1}H-NMR: (400.3 MHz, d^8-THF): δ = 0.02 (s, 9H; Si(C*H*$_3$)$_3$), 2.87 (dd, $^2J_{HP}$ = 6.8 Hz, $^3J_{HP}$ = 5.4 Hz, 1H; SC*H*P), 5.80 (s, 6H; C*H*$_{Benzol}$), 6.18-6.23 (m, 1H; C*H*-2), 6.59-6.68 (m, 3H; C*H*$_{P(N)Ph,meta}$ + C*H*-3), 6.79-6.86 (m, 4H; C*H*$_{P(N)Ph,ortho}$ + C*H*$_{SPh,meta}$), 6.86 (d, $^1J_{HP}$ = 369.4 Hz, 1H; P*H*), 6.95-7.04 (m, 5H; C*H*$_{P(H)Ph,ortho,para}$ + C*H*$_{SPh,para}$), 7.11-7.14 (m, 2H; C*H*$_{P(H)Ph,meta}$), 7.27-7.32 (m, 1H; C*H*$_{P(N)Ph,para}$), 7.37-7.39 (m, 2H; C*H*$_{SPh,ortho}$), 7.50 (br s, 3H; C*H*$_{P(H)Ph,meta}$ + C*H*-4), 7.81-7.82 (m, 1H; C*H*-5), 8.35-8.39 (m, 2H; C*H*$_{P(H)Ph,ortho}$). **^{13}C{^{1}H}-APT-NMR:** (100.7 MHz, d^8-THF): δ = 5.20 (d, $^3J_{CP}$ = 2.6 Hz; Si(*C*H$_3$)$_3$), 55.0 (dd, $^1J_{CP}$ = 63.3 Hz, $^2J_{CP}$ = 9.4 Hz; S*C*HP), 93.5 (d, $^2J_{CP}$ = 3.0 Hz; *C*H$_{Benzol}$), 123.0 (d, $^3J_{CP}$ = 12.1 Hz; *C*H-3), 127.3 (d, $^3J_{CP}$ = 11.1 Hz; *C*H$_{P(N)Ph,meta}$), 128.4 (*C*H$_{SPh,meta}$), 128.8 (d, $^4J_{CP}$ = 2.8 Hz; *C*H$_{P(H)Ph,para}$), 129.0 (*C*H$_{SPh,ortho}$), 129.1 (d, $^4J_{CP}$ = 2.7 Hz; *C*H$_{P(H)Ph,para}$), 129.4 (d, $^3J_{CP}$ = 9.1 Hz; *C*H$_{P(H)Ph,meta}$), 129.7 (d, $^3J_{CP}$ = 9.8 Hz; *C*H$_{P(H)Ph,meta}$), 129.9 (dd, $^2J_{CP}$ = 18.3 Hz, $^4J_{CP}$ = 1.5 Hz; *C*H-2), 130.3 (d, $^4J_{CP}$ = 2.1 Hz; *C*H$_{P(N)Ph,para}$), 130.4 (d, $^1J_{CP}$ = 42.4 Hz; *C*$_{P(H)Ph,ipso}$), 131.6 (d, $^4J_{CP}$ = 1.8 Hz; *C*H-4), 131.8 (*C*H$_{SPh,para}$), 132.4 (br; *C*H$_{P(N)Ph,ortho}$), 133.6 (d, $^2J_{CP}$ = 8.0 Hz; *C*H$_{P(H)Ph,ortho}$), 135.3 (d, $^2J_{CP}$ = 10.2 Hz; *C*H$_{P(H)Ph,ortho}$), 136.9(d, $^1J_{CP}$ = 42.7 Hz; *C*$_{P(H)Ph,ipso}$), 138.6 (d, $^1J_{CP}$ = 83.8 Hz; *C*$_{P(N)Ph,ipso}$), 143.2 (dd, $^3J_{CP}$ = 11.0 Hz, $^3J_{CP}$ = 14.5 Hz; *C*H-5), 145.1 (*C*$_{SPh,ipso}$), 154.7 (d, $^1J_{CP}$ = 119.0; *C*-1), 166.3 (dd, $^2J_{CP}$ = 24.3 Hz, $^2J_{CP}$ = 26.4 Hz; *C*-6). **^{31}P{^{1}H}-NMR:** (162.1 MHz, d^8-THF): δ = 14.7 (d, $^3J_{PP}$ = 3.9 Hz; (N)*P*),

40.2 (d, $^3J_{PP}$ = 3.9 Hz; (H)*P*). Anal. calcd. for $C_{40}H_{41}NO_2P_2RuSSi$: C, 60.74; H, 5.23; N, 1.77; S, 4.05. Found: C, 61.02; H, 5.30; N, 1.93; S, 3.61.

Synthesis of complex 6. 150 mg (178 µmol) of **3a** were dissolved in 5 mL toluene and the reaction mixture stirred for 3 d at 50 °C. The solvent was removed under reduced pressure and the residue washed eight times with pentane (10 mL) giving compound **6** as a pale yellow solid. (116 mg, 144 µmol, 81 %).

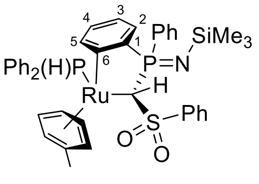

6

^{1}H-NMR: (400.3 MHz, d^8-THF): δ = 0.06 (s, 9H; Si(C*H*$_3$)$_3$), 1.81 (s, 3H; CC*H*$_3$), 2.92 (dd, $^2J_{HH}$ = 7.2 Hz, $^3J_{HH}$ = 5.2 Hz, 1H; SC*H*P), 5.18 (d, $^3J_{HH}$ = 6.0 Hz; C*H*$_{Tol}$), 5.75-5.79 (m, 2H; C*H*$_{Tol}$), 6.15-6.18 (m, 1H; C*H*$_{Tol}$), 6.21-6.27 (m, 2H; C*H*$_{Tol}$ + C*H*-2), 6.56-6.61 (m, 1H; C*H*$_{PPh,ortho}$), 6.64-6.69 (m, 1H; C*H*-3), 6.72 (d, $^1J_{HP}$ = 364.0 Hz, 1H; P*H*), 6.79-6.85 (m, 3H; C*H*$_{SPh,meta}$ + C*H*$_{P(H)Ph,para}$), 6.96-7.07 (m, 6H; C*H*$_{P(H)Ph,ortho}$ + C*H*$_{P(H)Ph,para}$ + C*H*$_{P(N)Ph,meta}$ + C*H*-4), 7.10-7.15 (m, 2H; C*H*$_{P(H)Ph,meta}$), 7.27-7.31 (m, 1H; C*H*$_{P(N)Ph,para}$), 7.38-7.40 (m, 2H; C*H*$_{SPh,ortho}$), 7.50-7.52 (m, 3H; C*H*$_{P(H)Ph,meta}$ + C*H*$_{SPh,para}$), 7.76-7-77 (m, 1H; C*H*-5), 8.37-8.42 (m, 2H; C*H*$_{P(H)Ph,ortho}$). **^{13}C{^{1}H}-APT-NMR:** (100.7 MHz, d^8-THF): δ = 4.95 (d, $^3J_{CP}$ = 2.3 Hz; Si(*C*H$_3$)$_3$), 18.3 (C*C*H$_3$), 53.7 (dd, $^2J_{CP}$ = 62.8 Hz, $^3J_{CP}$ = 9.2 Hz; S*C*HP), 91.7 (d, $^2J_{CP}$ = 5.6 Hz; *C*H$_{Tol}$), 92.0 (*C*H$_{Tol}$), 93.8 (*C*H$_{Tol}$), 94.1 (d, $^2J_{CP}$ = 4.4 Hz; *C*H$_{Tol}$), 95.0 (d, $^2J_{CP}$ = 5.7 Hz; *C*H$_{Tol}$), 104.0 (d, $^2J_{CP}$ = 2.4 Hz; *C*$_{Tol}$), 122.6 (d, $^3J_{CP}$ = 12.1 Hz; *C*H-3), 126.9 (d, $^2J_{CP}$ = 11.2 Hz; *C*H$_{P(N)Ph,ortho}$), 127.9 (*C*H$_{SPh,meta}$); 125.4 (d, $^4J_{CP}$ = 2.7 Hz; *C*H$_{P(H)Ph,para}$), 128.6 (*C*H$_{P(H)Ph,para}$ (found in HSQC NMR spectrum)), 128.6 (*C*H$_{SPh,ortho}$), 190.0 (d, $^3J_{CP}$ = 9.0 Hz; *C*H$_{P(H)Ph,meta}$), 129.3 (d, $^3J_{CP}$ = 9.7 Hz; *C*H$_{P(H)Ph,meta}$), 129.7 (dd, $^2J_{CP}$ = 18.4 Hz, $^4J_{CP}$ = 1.3 Hz; *C*H-2), 129.9 (d, $^4J_{CP}$ = 2.0 Hz; *C*H$_{P(N)Ph,para}$), 130.3 (d, $^1J_{CP}$ = 42.3 Hz; *C*$_{P(N)Ph,ipso}$), 131.2 (*C*H$_{SPh,para}$), 131.3 (*C*H$_{P(N)Ph,meta}$), 131.9 (d, $^4J_{CP}$ = 9.9 Hz; *C*H-4), 133.3 (d, $^2J_{CP}$ = 7.9 Hz; *C*H$_{P(H)Ph,ortho}$), 135.1 (d, $^2J_{CP}$ = 10.2 Hz; *C*H$_{P(H)Ph,ortho}$), 136.7 (d, $^1J_{CP}$ = 42.5 Hz; *C*$_{P(H)Ph,ipso}$), 138.6 (d, $^1J_{CP}$ = 82.7 Hz; *C*$_{P(H)Ph,ipso}$), 142.4 (dd, $^3J_{CP}$ = 14.7 Hz, $^3J_{CP}$ = 11.1 Hz; *C*H-5), 144.8 (*C*$_{SPh,ipso}$), 154.1 (d, $^1J_{CP}$ = 21.3 Hz; *C*-1), 168.4 (dd, $^2J_{CP}$ = 26.8 Hz, $^2J_{CP}$ = 23.8 Hz; *C*-6). **^{31}P{^{1}H}-NMR:** (162.1 MHz, d^8-THF): δ = 14.7 (d, $^3J_{PP}$ = 3.9 Hz; *P*(N)), 43.7 (d, $^3J_{PP}$ = 3.9 Hz; *P*(H)). **^{31}P-NMR:** (162.1 MHz, d^8-THF): δ = 14.8 (*P*(N)), 43.6 (d, $^1J_{HP}$ = 365.4 Hz; *P*(H)). Anal. calcd. for $C_{41}H_{43}NO_2P_2RuSSi$: C, 61.18; H, 5.38; N, 1.74; S, 3.98. Found: C, 60.72; H, 5.40; N, 1.85; S, 3.71.

Synthesis of ligand 8. 55 mL of THF were added to 525 mg (4.00 mmol) Nitroaniline and 345 mg (14.4 mmol) sodium hydride and the resulting reaction mixture was stirred until no further gas evolution could be observed leading to the formation of a brown solution and a yellow solid. A slurry of 2.00 g (4.00 mmol) of bromide **7** in 20 mL THF was added and the reaction mixture stirred until no further gas evolution was observed. The reaction mixture was cooled to 0 °C and 30 mL H_2O were added slowly (Caution: fierce gas evolution!). The resulting mixture was extracted three times with CH_2Cl_2 (30 mL) and the combined organic phases dried over Na_2SO_4. Filtration and removal of the solvent *in vacuo* lead to a yellow solid which was washed six times with Et_2O (20 mL) giving compound **8** as a yellow solid. (1.14 g, 2.39 mmol, 60%).

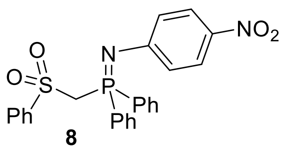

8

^{1}H-NMR: (300.2 MHz, CD_2Cl_2): δ = 4.52 (d, $^2J_{HP}$ = 9.5 Hz, 2H; SC*H*$_2$P), 6.39-6.45 (m, 2H; PNCC*H*), 7.35-7.41 (m, 2H; C*H*$_{SPh,meta}$), 7.49-7.57 (m, 5H; C*H*$_{PPh,meta}$ + C*H*$_{SPh,para}$), 7.60-7.67 (m, 2H; C*H*$_{PPh,para}$), 7.73-7.81 (m, 8H; C*H*$_{SPh,ortho}$ + C*H*$_{PPh,ortho}$ + O_2NCC*H*). **^{13}C{^{1}H}-NMR:** (75.5 MHz, CD_2Cl_2): δ = 54.2 (d, $^1J_{CP}$ = 53.1 Hz; S*C*H$_2$P), 122.7 (d, $^3J_{CP}$ = 20.2 Hz; PNC*C*H), 125.4 (d, $^4J_{CP}$ = 2.0 Hz; *C*H$_{PPh,para}$), 128.1 (d, $^1J_{CP}$ = 103.0 Hz; C$_{PPh,ipso}$), 128.6 (*C*H$_{SPh,meta}$), 129.5 (*C*H$_{SPh,ortho}$), 129.6 (d, $^3J_{CP}$ = 12.7 Hz; *C*H$_{PPh,meta}$), 132.1 (d, $^2J_{CP}$ = 10.1 Hz; *C*H$_{PPh,ortho}$), 133.4 (d, $^4J_{CP}$ = 3.1 Hz; O_2NC*C*H), 134.5 (*C*H$_{SPPh,para}$), 139.1

($C_{\mathrm{SPh,ipso}}$). **^{31}P{^{1}H}-NMR:** (121.5 MHz, CD_2Cl_2): δ = –4.55. Anal. calcd. for $C_{25}H_{21}N_2O_4PS$: C, 63.02; H, 4.44; N, 5.88; S, 6.73. Found: C, 62.99; H, 4.45; N, 5.84; S, 6.60.

Synthesis of monoanion 8-Na. 500 mg (1.05 mmol) of compound **7** and 25.2 mg (1.05 mmol) sodium hydride were suspended in 10 mL THF and the resulting brown mixture stirred until no further gas evolution could be observed. The solvent was removed in vacuo giving rise to monoanion **7-Na** as a brown solid (520 mg, 973 µmol, 93%).

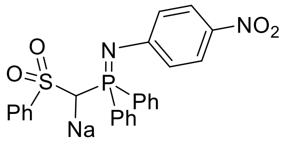

8-Na

^{1}H-NMR: (500.1 MHz, d^{6}-DMSO): δ = 1.76 m (2H; $CH_{2,\mathrm{THF}}$), 2.90 (d, $^{2}J_{HP}$ = 12.9 Hz, 1H; SC*H*NaP), 3.60 (m, 2H; $OCH_{2,\mathrm{THF}}$), 6.54 (d, $^{3}J_{HH}$ = 9.2 Hz, 2H; PNCC*H*), 7.03-7.11 (m, 3H; $CH_{\mathrm{SPh,para}}$ + $CH_{\mathrm{PPh,para}}$), 7.31-7.38 (m, 6H; $CH_{\mathrm{PPh,meta}}$ + $CH_{\mathrm{SPh,meta}}$), 7.41-7.42 (m, 2H; $CH_{\mathrm{SPh,ortho}}$), 7.56-7.68 (m, 2H; O_2NCCH), 7.80-7.84 (m, 4H; $CH_{\mathrm{PPh,ortho}}$). **^{13}C{^{1}H}-NMR:** (125.8 MHz, d^{6}-DMSO): δ = 25.1 ($CH_{2,\mathrm{THF}}$), 41.8 (d, $^{1}J_{CP}$ = 8.2 Hz; S*C*HNaP), 67.0 ($OCH_{2,\mathrm{THF}}$), 121.0 (d, $^{3}J_{CP}$ = 22.8 Hz; PNC*C*H), 124.4 ($CH_{\mathrm{SPh,meta}}$), 124.8 (d, $^{4}J_{CP}$ = 2.2 Hz; O_2NCCH), 124.4 ($CH_{\mathrm{SPh,ortho}}$), 127.9 (d, $^{3}J_{CP}$ = 11.7 Hz; $CH_{\mathrm{PPh,meta}}$), 128.2 ($CH_{\mathrm{SPh,para}}$), 129.9 (d, $^{4}J_{CP}$ = 2.4 Hz; $CH_{\mathrm{PPh,para}}$), 131.6 (d, $^{2}J_{CP}$ = 9.1 Hz; $CH_{\mathrm{PPh,ortho}}$), 133.2 (PN*C*), 135.6 (d, $^{1}J_{CP}$ = 112.2 Hz; $C_{\mathrm{PPh,ipso}}$), 151.2 (O_2NC), 164.3 (d, $^{3}J_{CP}$ = 3.9 Hz; $C_{\mathrm{SPh,ipso}}$). **^{31}P{^{1}H}-NMR:** (202.5 MHz, d^{6}-DMSO): δ = 4.95. Anal. calcd. for $C_{31}H_{32}N_2NaO_{5.5}PS$: C, 61.38; H, 5.32; N, 4.62; S, 5.28. Found: C, 61.49; H, 5.55; N, 4.94; S, 5.31.

Synthesis of chloro complex 9. 50.0 mg (93.5 µmol) of monoanion **8-Na** and 28.6 mg (46.8 µmol) [(*p*-cymene)$RuCl_2$]$_2$ were suspended in 5 mL toluene. The reaction mixture was stirred for 18 h at RT, filtrated and the solvent removed under reduced pressure. The residue was washed with 5 mL Et_2O giving chlorido complex **9** as an orange solid (yield: 87%, 60.7 mg, 81.3 mmol).

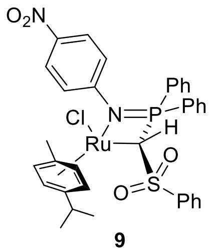

9

^{1}H-NMR: (300.1 MHz, CD_2Cl_2): δ = 1.20 (d, $^{3}J_{HH}$ = 6.9 Hz, 3H; CH(CH_3)$_2$), 1.30 (d, $^{3}J_{HH}$ = 7.0 Hz, 3H; CH(CH_3)$_2$), 2.06 (s, 3H; CCH_3), 2.84 (sept, $^{3}J_{HH}$ = 7.1 Hz, 1H; C*H*(CH_3)$_2$), 4.00 (d, $^{2}J_{HP}$ = 7.4 Hz, 1H; SC*H*P), 5.64 (d, $^{3}J_{HH}$ = 6.3 Hz, 1H; CH_{Cym}), 5.80-5.90 (m, 3H; CH_{Cym}), 6.97-7.01 (m, 2H; PNCC*H*), 7.04-7.11 (m, 2H; $CH_{\mathrm{PPh,meta}}$), 7.17-7.24 (m, 2H; $CH_{\mathrm{SPh,meta}}$), 7.29-7.37 (m, 4H; $CH_{\mathrm{PPh,ortho,meta}}$), 7.40-7.46 (m, 1H; $CH_{\mathrm{PPh,para}}$), 7.46-7.58 (m, 5H; $CH_{\mathrm{PPh,ortho}}$ + $CH_{\mathrm{SPh,ortho,para}}$), 7.75-7.80 (m, 1H; $CH_{\mathrm{PPh,para}}$), 7.82-7.85 (m, 2H; O_2NCCH). **^{13}C{^{1}H}-NMR:** (75.5 MHz, CD_2Cl_2): δ = 19.2 (CCH_3), 21.6 (CH(CH_3)$_2$), 32.8 (CH(CH_3)$_2$), 31.1 (*C*(CH_3)$_2$), 34.1 (d, $^{1}J_{CP}$ = 64.4 Hz; S*C*HP), 80.6 (CH_{Cym}), 81.3 (CH_{Cym}), 86.1 (CH_{Cym}), 88.6 (CH_{Cym}), 95.2 (C_{Cym}), 101.3 (C_{Cym}), 123.4 (d, $^{3}J_{CP}$ = 12.2 Hz; PNC*C*H), 124.9 ($CH_{\mathrm{SPh,meta}}$), 125.5 (d, $^{1}J_{CP}$ = 78.5 Hz; $C_{\mathrm{PPh,ipso}}$), 126.8 ($CH_{\mathrm{SPh,ortho}}$), 128.7 (d, $^{3}J_{CP}$ = 11.9 Hz; $CH_{\mathrm{PPh,meta}}$),128.8 (d, $^{1}J_{CP}$ = 74.2 Hz; $C_{\mathrm{PPh,ipso}}$), 129.0 (d, $^{3}J_{CP}$ = 11.9 Hz; $CH_{\mathrm{PPh,meta}}$), 129.2 (O_2NCCH), 123.4 (d, $^{2}J_{CP}$ = 10.0 Hz; $CH_{\mathrm{PPh,ortho}}$), 132.8 ($CH_{\mathrm{SPh,para}}$), 133.0 (d, $^{4}J_{CP}$ = 3.0 Hz; $CH_{\mathrm{PPh,para}}$), 134.3 (d, $^{4}J_{CP}$ = 2.6 Hz; $CH_{\mathrm{PPh,para}}$), 134.4 (d, $^{2}J_{CP}$ = 8.0 Hz; $CH_{\mathrm{PPh,ortho}}$), 140.3 (PN*C*), 144.6 (O_2NC), 157.2 ($C_{\mathrm{SPh,ipso}}$). **^{31}P{^{1}H}-NMR:** (121.5 MHz, CD_2Cl_2): δ = 50.9. Anal. calcd. for $C_{35}H_{34}ClN_2O_4PRuS$: C, 56.34; H, 4.59; N, 3.75; S, 4.30. Found: C, 55.93; H, 4.63; N, 3.57; S, 3.99.

Synthesis of tetrafluroborate complex 10. 1.29 g (1.73 mmol) of chlorido complex **9** and 337 mg (1.73 mmol) $AgBF_4$ were suspended in 20 mL dichloromethane and the resulting brown suspension stirred for 16 h. The mixture was filtrated through a 4 Å frit with a 2 cm Celite pad. The solvent was removed in vacuo and the brown residue washed with 20 mL Hexane, giving compound **10** as a dark brown solid. (1.34 g, 1.68 mmol, 97%).

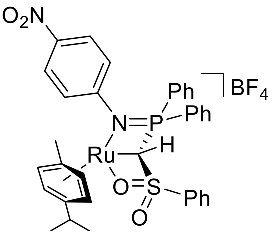

10

^{1}H-NMR: (400.3 MHz,CD_2Cl_2): δ = 1.26 (d, $^{3}J_{HH}$ = 6.9 Hz, 3H; CHC*H*$_3$), 1.30 (d, $^{3}J_{HH}$ = 6.8 Hz, 3H; CHC*H*$_3$), 1.706 (s, 3H; CC*H*$_3$), 2.69 (sept, $^{3}J_{HH}$ = 6.9 Hz, 1H; C*H*(CH_3)$_2$), 4.50 (d, $^{2}J_{HP}$ = 3.7 Hz, 1H; PC*H*S), 5.45-5.50 (m, 2H; CH_{Cym}), 5.69 (d, $^{3}J_{HH}$ = 5.9 Hz, 1H; CH_{Cym}), 5.92 (d, $^{3}J_{HH}$ = 5.8 Hz, 1H; CH_{Cym}), 7.06-7.11 (m, 2H; C$H_{PPh,meta}$), 7.17-7.22 (m, 4H; C$H_{PPh,meta}$ + PNCCH), 7.34-7.38 (m, 2H; C$H_{SPh,meta}$), 7.47-7.55 (m, 4H; C$H_{PPh,para}$ + C$H_{SPh,para}$ + O_2NCC*H*), 7.73-7.86 (m, 5H; C$H_{PPh,ortho}$ + C$H_{PPh,para}$), 8.05 (d, $^{3}J_{HH}$ = 8.9, 2H; C$H_{SPh,ortho}$). **^{13}C{^{1}H}-NMR:** (100.7 MHz, CD_2Cl_2): δ = 18.7 (*C*CH_3), 21.8 (S*C*HP, found in HSQC NMR spectrum), 22.7 (CH(*C*H_3)$_2$), 22.8 (CH(*C*H_3)$_2$), 32.0 (*C*H(CH_3)$_2$), 81.6 (*C*H_{Cym}), 81.8 (*C*H_{Cym}), 82.3 (*C*H_{Cym}), 84.5 (*C*H_{Cym}), 95.3 (*C*$_{Cym}$), 106.2 (*C*$_{Cym}$), 123.3 (d, $^{3}J_{CP}$ = 12.1 Hz; NC*C*H), 125.0 (d, $^{1}J_{CP}$ = 71.5 Hz; *C*$_{PPh}$), 125.6 (O_2NC*C*H), 125.7 (*C*$H_{SPh,ortho/meta}$), 126.4 (d, $^{1}J_{CP}$ = 90.7 Hz; *C*$_{PPh}$), 129.5 (d, $^{3}J_{CP}$ = 12.3 Hz; *C*$H_{PPh,meta}$), 129.8 (*C*$H_{SPh,ortho/meta}$), 130.5 (d, $^{2}J_{CP}$ = 12.6 Hz; *C*$H_{PPh,ortho}$), 131.6 (d, $^{2}J_{CP}$ = 19.7 Hz; *C*$H_{PPh,ortho}$), 133.3 (d, $^{3}J_{CP}$ = 11.2 Hz; *C*$H_{PPh,meta}$), 134.3 (*C*$H_{SPh,para}$), 134.8 (d, $^{4}J_{CP}$ = 2.8 Hz; *C*$H_{PPh,para}$), 135.0 (br s; *C*$H_{PPh,para}$), 142.2 (d, $^{3}J_{CP}$ = 9.8 Hz; *C*$H_{SPh,para}$), 155.0 (O_2N*C*). **^{31}P{^{1}H}-NMR:** (162.1 MHz, CD_2Cl_2): δ = 41.3 (br). Anal. calcd. for $C_{35}H_{34}BF_4N_2O_4PRuS$: C, 52.71; H, 4.30; N, 3.51; S, 4.02. Found: C, 52.84; H, 4.57; N, 3.37; S, 3.85.

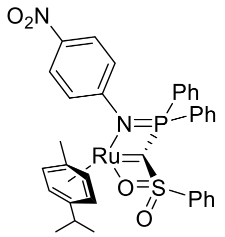

2b

Synthesis of carbene complex 2b. 1.20 g (1.50 mmol) of complex **9** and 187 mg (1.50 mmol) of KOtBu were dissolved in 30 mL THF and the reaction mixture stirred for 18 h at RT during which a colour shift to green appeared. The solvent was removed under reduced pressure and 30 ml DCM were added. The resulting solution was filtered through a 4 Å frit with a three cm Celite pad. Removal of the solvent *in vacuo* gave carbene complex **2b** as a dark green solid. (1.01 g, 1.42 mmol, 94 %)

^{1}H-NMR: (300.1 MHz, d^{8}-THF): δ = 1.18 (d, $^{3}J_{HH}$ = 6.9 Hz, 6H; CH(C*H*$_3$)$_2$), 2.16 (s, 3H; CC*H*$_3$), 2.48 (sept, $^{3}J_{HH}$ = 6.9 Hz, 1H; C*H*(CH_3)$_2$), 5.68 (br s, 4H; CH_{Cym}), 6.91-6.94 (m, 2H; PNCC*H*), 7.14-7.20 (m, 2H; C$H_{SPh,ortho}$), 7.28-7.34 (m, 1H; C$H_{SPh,para}$), 7.38-7.44 (m, 6H; C$H_{SPh,meta}$ + C$H_{PPh,meta}$), 7.51-7.57 (m, 2H; C$H_{PPh,para}$), 7.65-7.73 (m, 4H; C$H_{PPh,ortho}$), 7.85-7.88 (m, 2H; O_2NC*H*). **^{13}C{^{1}H}-APT-NMR:** (75.5 MHz, d^{8}-THF): δ = 20.2 (C*C*H_3), 24.0 (CH(*C*H_3)$_2$), 32.4 (*C*H(CH_3)$_2$), 80.6 (*C*H_{Cym}), 81.2 (*C*H_{Cym}), 87.1 (*C*$_{Cym}$), 99.0 (*C*$_{Cym}$), 120.0 (d, $^{3}J_{CP}$ = 14.8 Hz; PNC*C*H), 125.0 (O_2NC*C*H), 126.9 (*C*$H_{SPh,meta}$), 128.0 (d, $^{1}J_{CP}$ = 70.5 Hz; *C*$_{PPh,ipso}$), 128.6 (*C*$H_{SPh,ortho}$), 129.2 (d, $^{3}J_{CP}$ = 11.3 Hz; *C*$H_{PPh,meta}$), 130.7 (*C*$H_{SPh,para}$), 132.9 (d, $^{4}J_{CP}$ = 2.4 Hz; *C*$H_{PPh,para}$), 133.2 (d, $^{2}J_{CP}$ = 10.6 Hz; *C*$H_{PPh,ortho}$), 139.2 (PN*C*), 143.6 (d, $^{1}J_{CP}$ = 66.6 Hz; S*C*P), 148.3 (d, $^{3}J_{CP}$ = 2.6 Hz; *C*$_{SPh,ipso}$), 157.8 (O_2N*C*). **^{31}P{^{1}H}-NMR:** (101.3 MHz, d^{8}-THF): δ = 65.3. Anal. calcd. for $C_{35}H_{33}N_2O_4PRuS$: C, 59.23; H, 4.69; N, 3.95; S, 4.52. Found: C, 58.84; H, 4.72; N, 4.27; S, 4.28.

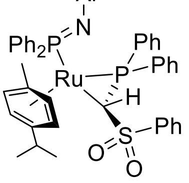

4c

Ar = 4-C_6H_4-NO_2

Synthesis of compound 4c. 159 mg (224 µmol) of **2b** were dissolved in 10 ml toluene and the mixture cooled to -78 °C. 41.7 mg (224 µmol) of $HPPh_2$ were added, the icebath removed, and the reaction stirred for 15 min at RT. The solvent was removed *in vacuo* and the residue washed three times with 10 ml pentane. The residue was further washed two times with a mixture of 20 ml pentane and 10 ml toluene and additional two times with 20 ml pentane. Afterwards, the solid was dried 6 hours at 45 °C under reduced pressure to remove residual solvent giving **4c** as a dark brown solid (yield: 105 mg, 117 mmol, 52 %).

^{1}H-NMR: (400.3 MHz, C_6D_6): δ = 0.95 (d, $^{3}J_{HH}$ = 6.9 Hz, 3H; CH(C*H*$_3$)$_2$), 1.01 (d, $^{3}J_{HH}$ = 6.8 Hz, 3H; CH(C*H*$_3$)$_2$), 2.37 (s, 3H; CC*H*$_3$), 2.58 (sept, $^{3}J_{HH}$ = 6.8 Hz, 1H, C*H*(CH_3)$_2$), 4.27 (br d, $^{3}J_{HH}$ = 3.5 Hz, 1H; CH_{Cym}), 4.99 (br d, $^{3}J_{HH}$ = 6.2 Hz, 1H; CH_{Cym}), 5.39 (br d, $^{3}J_{HH}$ = 5.7 Hz, 1H; CH_{Cym}), 5.50 (d, $^{2}J_{HP}$ = 14.6 Hz, 1H; SC*H*P), 5.00 (br d, $^{3}J_{HH}$ = 6.4 Hz, 1H; CH_{Cym}), 6.43 (d, $^{3}J_{HH}$ = 9.1 Hz, 2H; PNCC*H*), 6.49-6.53 (m, 2H; C$H_{PPh,meta}$), 6.57-6.61 (m, 2H; C$H_{PPh,ortho}$), 6.68-673 (m, 1H; C$H_{PPh,para}$), 6.88-6.93 (m, 5H;

$CH_{SPh,meta}$ + $CH_{PPh,meta}$ + $CH_{SPh,para}$), 6.94-7.07 (m, 7H; 3 x $CH_{PPh,meta}$ + $CH_{PPh,para}$), 7.31-7.37 (m, 2H; $CH_{PPh,ortho}$), 7.48-7.58 (m, 4H; 2 x $CH_{PPh,ortho}$), 7.68 (d, $^3J_{HH}$ = 7.6 Hz, 2H; $CH_{SPh,ortho}$), 8.21 (d, $^3J_{HH}$ = 9.0 Hz, 2H; O_2NCCH). **$^{13}C\{^1H\}$-NMR:** (100.7 MHz, C_6D_6): δ = 19.7 (CCH_3), 24.1 ($CH(CH_3)_2$), 25.0 ($CH(CH_3)_2$), 31.7 ($CH(CH_3)_2$), 34.3 (dd, $^1J_{CP}$ = 22.3 Hz, $^2J_{CP}$ = 6.4 Hz; SCHP), 88.7 (d, $^2J_{CP}$ = 6.0 Hz; CH_{Cym}), 89.3 (d, $^2J_{CP}$ = 5.80 Hz; CH_{Cym}), 89.6 (CH_{Cym}), 97.4 (br; CH_{Cym}), 103.7 (d, $^2J_{CP}$ = 4.9 Hz; C_{Cym}), 114.7 (C_{Cym}), 121.3 (d, $^2J_{CP}$ = 19.2 Hz; PNCCH), 125.1 (d, $^1J_{CP}$ = 50.9 Hz; $C_{PPh,ipso}$), 125.8 (d, $^4J_{CP}$ = 1.8 Hz; O_2NCCH), 126.7 ($CH_{SPh,ortho}$), 127.5 (d, $^3J_{CP}$ = 10.3 Hz; $CH_{PPh,meta}$), 128.0 ($CH_{PPh,meta}$; found in HSQC), 128.1 ($CH_{PPh,meta}$; found in HSQC), 128.2 ($CH_{PPh,meta}$; found in HSQC), 128.9 ($CH_{SPh,meta}$), 129.0 (br, $CH_{PPh,para}$), 130.1 (br d, $^4J_{CP}$ = 3.1 Hz; $CH_{PPh,para}$), 130.4 (d, $^1J_{CP}$ = 49.3 Hz; $C_{PPh,ipso}$), 130.8 (d, $^2J_{CP}$ = 9.4 Hz; $CH_{PPh,ortho}$), 131.0 (br d, $^4J_{CP}$ = 3.3 Hz; $CH_{PPh,para}$), 132.1 ($CH_{SPh,para}$), 132.2 (d, $^2J_{CP}$ = 11.1 Hz; $CH_{PPh,ortho}$), 133.5 (d, $^2J_{CP}$ = 11.3 Hz; $CH_{PPh,ortho}$), 135.2 (d, $^2J_{CP}$ = 11.6 Hz; $CH_{PPh,ortho}$), 136.0 (O_2NC), 141.4 (br, $C_{PPh,ipso}$), 143.6 (d, $^1J_{CP}$ = 32.0 Hz; $C_{PPh,ipso}$), 145.8 ($C_{SPh,ipso}$), 162.3 (d, $^2J_{CP}$ = 13.7 Hz; PNC). **$^{31}P\{^1H\}$-NMR:** (162.1 MHz, C_6D_6): δ = –14.9 (d, $^2J_{PP}$ = 44.3 Hz; PN), 35.7 (d, $^2J_{PP}$ = 44.3 Hz; SCHP). **4c** revealed itself to be surprisingly unstable in solution (especially in polar coordinating or chlorinated solvents) thus preventing its completely clean isolation and therefore characterization via EA. Furthermore, NMR signals where broadened, hinting on fluxional behavior. This could not be resolved by means of low temperature NMR experiments and (in combination with the fast decomposition of **4c**) impeeded a complete NMR assignment (see Figures S22-S23 in the supporting information).

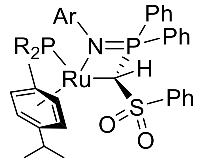

11a, R = 3,5-$C_6H_3Cl_2$

Synthesis of complex 11a. 200 mg (282 µmol) of carbene complex **2b** were dissolved in 4 mL toluene. 91.3 mg bis(3,5-dichlorophenyl)phosphine were dissolved in 3 mL toluene. Both solutions were cooled to –78 °C and the phosphine solution afterwards transferred into the solution of the carbene complex leading to an instantaneous colour change from dark green to brown. The solution was slowly warmed to RT and stirred for 17 hours. The solvent was removed under reduced pressure and the residue washed with 15 mL pentane giving compound **10a** as a dark brown solid. (204 mg, 197 µmol, 70%).

^1H-NMR: (400.3 MHz, d^8-THF): δ = 1.19 (d, $^3J_{HH}$ = 6.8 Hz, 3H; $CH(CH_3)_2$), 1.22 (d, $^3J_{HH}$ = 6.8 Hz, 3H; $CH(CH_3)2$), 2.02 (s, 3H; CCH_3), 2.80 (sept, $^3J_{HH}$ = 6.8 Hz, 1H; $CH(CH_3)_2$), 3.44 (dd, $^2J_{HP}$ = 7.3 Hz, $^3J_{HP}$ = 7.3 Hz, 1H; SCHP), 5.71-5.75 (m, 2H; CH_{Cym}), 5.81-5.84 (m, 2H; CH_{Cym}), 6.52-6.58 (m, 4H; NCCH + $CH_{PPh,ortho}$), 7.02-7.07 (m, 2H; $CH_{PPh,meta}$), 7.09-7.13 (m, 2H; $CH_{SPh,meta}$), 7.16-7.19 (m, 2H; ClCCHCCl), 7.25-7.26 (m, 4H; RuPCCH), 7.28-7.32 (m, 2H; $CH_{SPh,ortho}$), 7.38-7.41 (m, 1H; $CH_{PPh,para}$), 7.51-7.55 (m, 1H; $CH_{SPh,para}$), 7.58-7.63 (m, 2H; $CH_{PPh,meta}$), 7.74-7.84 (m, 5H; $CH_{PPh,ortho}$ + $CH_{PPh,para}$ + O2NCCH). **$^{13}C\{^1H\}$-NMR:** (100.7 MHz, d^8-THF): δ = 19.1 (CCH_3), 23.1 ($CH(CH_3)_2$), 23.2 ($CH(CH_3)_2$), 30.6 (dd, $^1J_{CP}$ = 59.8 Hz, $^2J_{CP}$ = 3.3 Hz; SCHP), 31.1 (d, $^3J_{CP}$ = 3.1 Hz; $CH(CH_3)_2$), 82.5 (CH_{Cym}), 86.0 (CH_{Cym}), 90.8 (CH_{Cym}), 92.2 (CH_{Cym}), 100.4 (C_{Cym}), 109.1 (C_{Cym}), 122.6 (d, $^2J_{CP}$ = 14.2 Hz; NCCH), 125.0 (O_2NCCH), 126.3 (d, $^1J_{CP}$ = 72.8 Hz; $C_{PPh,ipso}$), 126.5 (ClCCHCCl), 127.9 ($CH_{SPh,meta}$), 128.8 (d, $^3J_{CP}$ = 11.5 Hz; $CH_{PPh,meta}$), 129,3 (d, $^1J_{CP}$ = 74.0 Hz; $C_{PPh,ipso}$), 129.6 (d, $^3J_{CP}$ = 11.7 Hz; $CH_{PPh,meta}$), 129.7 ($CH_{SPh,ortho}$), 132.5 (dd, $^2J_{CP}$ = 9.9 Hz, $^5J_{CP}$ = 4.0 Hz; $CH_{PPh,ortho}$), 133.0 (d, $^2J_{CP}$ = 20.0 Hz; RuPCCH), 133.3 ($CH_{SPh,ortho}$), 133.5 (d, $^4J_{CP}$ = 2.7 Hz; $CH_{PPh,para}$), 135.0 (d, $^2J_{CP}$ = 10.1 Hz; $CH_{PPh,ortho}$), 135.1 (d, $^4J_{CP}$ = 2.8 Hz; $CH_{PPh,para}$), 135.3 (d, $^3J_{CP}$ = 4.8 Hz; ClC), 140.0 (NC), 145.3 (d, $^3J_{CP}$ = 1.1 Hz; $C_{SPh,ipso}$), 153.4 (br; RuPC), 157.1 (O_2NC). **$^{31}P\{^1H\}$-NMR:** (162.1 MHz, d^8-THF): δ = 23.2 (d, $^3J_{PP}$ = 2.6 Hz; RuP), 50.1 (d, $^3J_{PP}$ = 2.6 Hz; P(N)). Anal. calcd. for $C_{47}H_{40}Cl_4N_2O_4PRuS$: C, 54.61; H, 3.90; N, 2.71; S, 3.10. Found: C, 54.45; H, 4.01; N, 3.02; S, 2.78.

Synthesis of complex 11b. 100 mg (141 µmol) of carbene complex **2b** and 64.6 mg (141 µmol) of di(3,5-bis(trifluoromethyl)phenyl)phosphine were dissolved in 3 mL benzene and the reaction mixture stirred for 45 min. The solvent was removed under reduced pressure and the residue washed once with 6 mL pentane which after drying i*n vacuo* afforded complex **11b** in 87 % (143 mg, 123 µmol) yield as a brown solid.

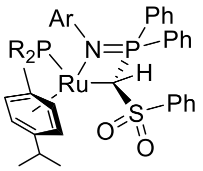

11b, R = 3,5-$C_6H_3(CF_3)_2$

^{1}H-NMR: (400.3 MHz, C_6D_6): δ = 0.97 (d, $^3J_{HH}$ = 6.9 Hz, 3H; CH(C*H*$_3$)$_2$), 0.99 (d, $^3J_{HH}$ = 6.8 Hz, 3H; CH(C*H*$_3$)$_2$), 1.70 (s, 3H; CC*H*$_3$), 2.80 (sept, $^3J_{HH}$ = 6.8 Hz, 1H; C*H*(CH$_3$)$_2$) 3.51 (dd, $^2J_{HP}$ = 7.2 Hz, $^3J_{HP}$ = 7.2 Hz, 1H; SC*H*P), 5.29 (d, $^3J_{HH}$ = 5.9 Hz, 1H; C*H*$_{Cym}$), 5.41 (d, $^3J_{HH}$ = 5.9 Hz, 1H; C*H*$_{Cym}$), 5.50 (d, $^3J_{HH}$ = 5.9 Hz, 1H; C*H*$_{Cym}$), 5.73 (d, $^3J_{HH}$ = 5.9 Hz, 1H; CH$_{Cym}$), 6.54-6.61 (m, 6H; C*H*$_{PPh,meta}$ + NCC*H* + C*H*$_{PPh,ortho}$), 6.73-6.76 (m, 2H; C*H*$_{SPh,meta}$), 6.79-6.82 (m, 1H; C*H*$_{PPh,para}$), 6.74-6.88 (m, 1H; C*H*$_{SPh,para}$), 7.04-7.08 (m, 2H; C*H*$_{PPh,meta}$), 7.13-7.14 (m, 2H; C*H*$_{SPh,ortho}$), 7.18-7.21 (m, 1H; C*H*$_{PPh,para}$), 7.60-7.65 (m, 4H; C*H*$_{PPh,ortho}$ + F$_3$CCC*H*CCF$_3$), 7.81 (d, $^3J_{HH}$ = 8.9 Hz, 2H; O$_2$NCC*H*), 8.06 (d, $^2J_{HP}$ = 3.8 Hz, 3H; RuPCCH). **^{13}C{^{1}H}-NMR:** (100.7 MHz, C_6D_6): δ = 18.7 (C*C*H$_3$), 22.4 (CH(*C*H$_3$)$_2$), 23.1 (CH(*C*H$_3$)$_2$), 30.1 (dd, $^2J_{HP}$ = 60.1 Hz, $^3J_{HP}$ = 3.7 Hz; SCHP), 30.7 (*C*H(CH$_3$)$_2$), 83.1 (*C*H$_{Cym}$), 84.4 (*C*H$_{Cym}$), 89.9 (*C*H$_{Cym}$), 90.7 (*C*H$_{Cym}$), 98.9 (*C*$_{Cym}$), 110.4 (*C*$_{Cym}$), 120.1 (br; CF$_3$C*C*HCCF$_3$), 121.7 (d, $^3J_{CP}$ = 14.2 Hz; NC*C*H), 124.1 (q, $^1J_{CF}$ = 273.3 Hz; *C*F$_3$), 124.8 (O2NC*C*H), 125.9 (d, $^1J_{CP}$ = 72.8 Hz; *C*$_{PPh,ipso}$), 127.2 (*C*H$_{SPh,ortho}$), 128,3 (d, $^3J_{CP}$ = 16.7 Hz; *C*H$_{PPh,meta}$), 128.7 (d, $^3J_{CP}$ = 11.7 Hz; *C*H$_{PPh,meta}$), 128.9 (*C*H$_{SPh,meta}$), 128.9 (d, $^1J_{CP}$ = 106.8 Hz; *C*$_{PPh,ipso}$), 131.6 (dq, $^2J_{CF}$ = 32.8 Hz, $^3J_{CP}$ = 0.1 Hz; F$_3$C*C*), 131.7 (d, $^2J_{CP}$ = 14.1 Hz; *C*H$_{PPh,ortho}$), 132.4 (*C*H$_{SPh,para}$), 132.9 (d, $^4J_{CP}$ = 2.8 Hz; *C*H$_{PPh,para}$), 134.1 (*C*H$_{PPh,para}$), 134.1(d, $^2J_{CP}$ = 10.1 Hz; *C*H$_{PPh,ortho}$), 134.4 (d, $^2J_{CP}$ = 19.1 Hz; RuPC*C*H), 140.2 (N*C*), 144.6 (*C*$_{SPh,ipso}$), 152.2 (d, $^1J_{CP}$ = 47.5 Hz; RuP*C*), 155.8 (O$_2$N*C*). **^{31}P{^{1}H}-NMR:** (162.1 MHz, C_6D_6): δ = 19.1 (br; Ru*P*), 49.8 (d, $^3J_{PP}$ = 3.8 Hz; *P*(N)). Anal. calcd. for $C_{51}H_{40}F_{12}N_2O_4PRuS$: C, 52.45; H, 3.45; N, 2.40; S, 2.74. Found: C, 52.52; H, 3.54; N, 2.35; S, 2.45.

Exchange experiment of 3a with DPPh$_2$. 70 mg (82.5 µmol) of compound **3a** were dissolved in 6 ml THF. 15.5 mg (82.5 µmol) of DPPh$_2$ were added and the reaction stirred at RT for 18 h. The solvent was removed under reduced pressure and the reaction monitored by NMR spectroscopy without further purification.

2. ^{1}H NMR, ^{13}C NMR, and ^{31}P NMR Spectra

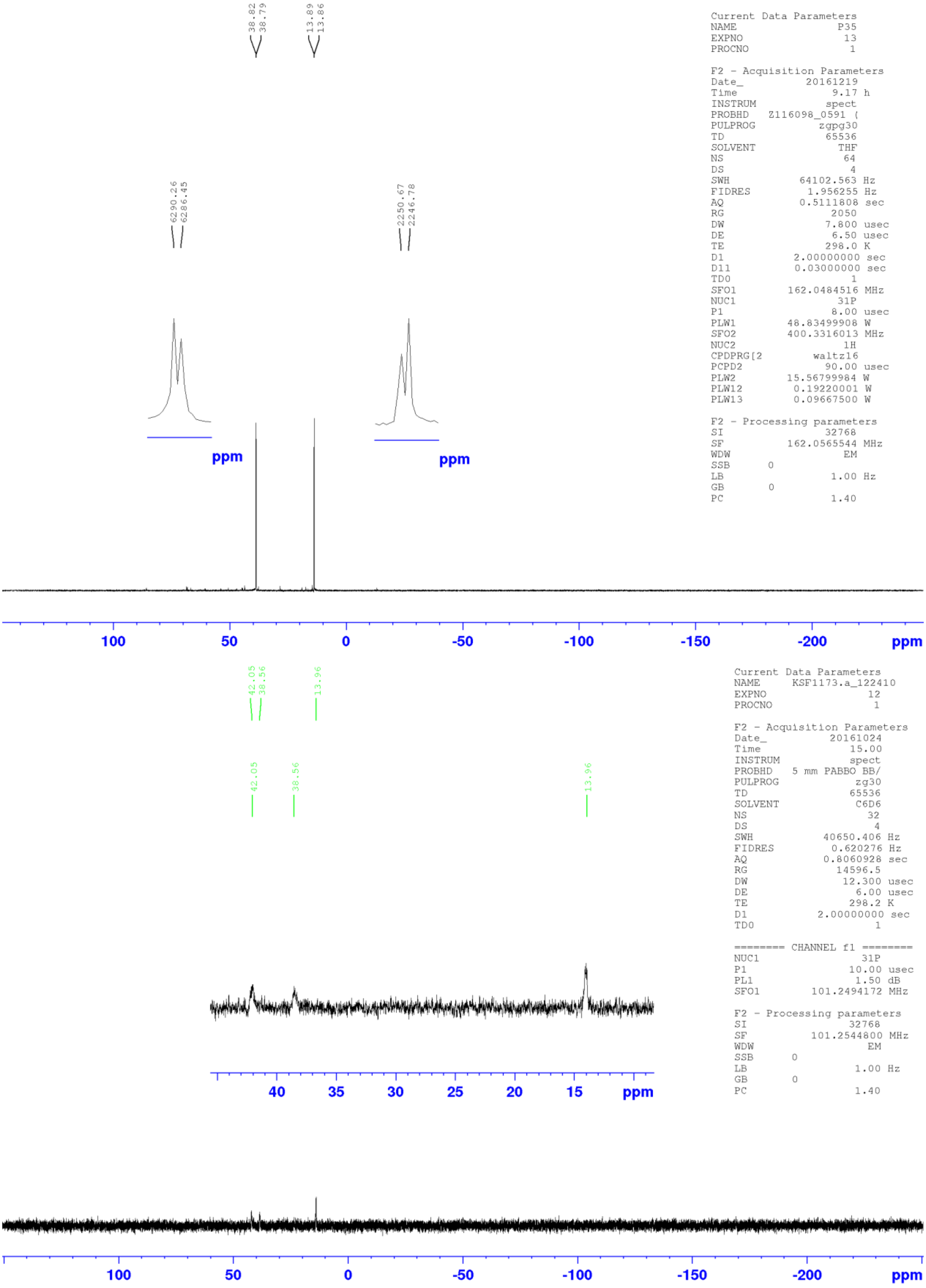

Figure S1. $^{31}P\{^{1}H\}$ NMR (top) and ^{31}P NMR (bottom) spectrum of **3a**.

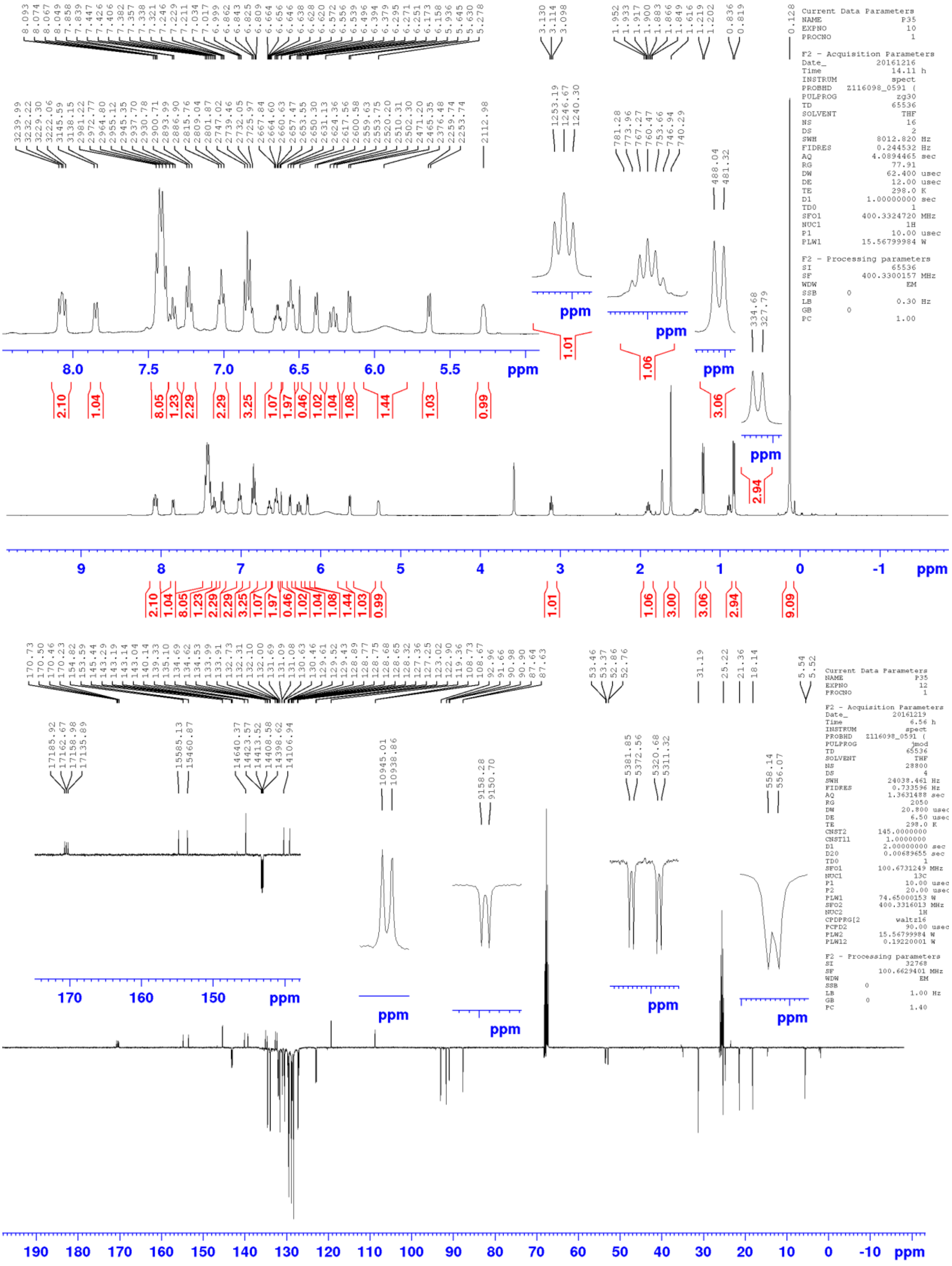

Figure S2. ^{1}H NMR (top) and $^{13}C\{^{1}H\}$-APT NMR (bottom) spectrum of **3a**.

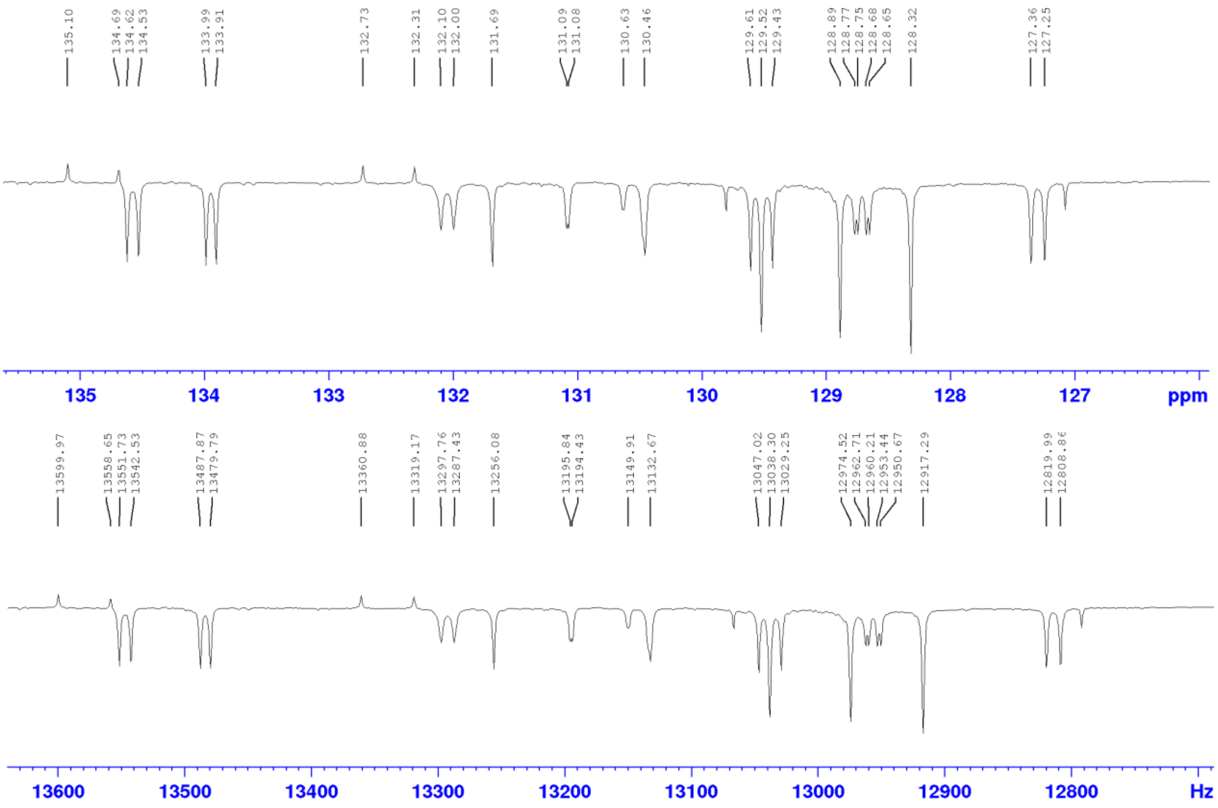

Figure S3. Section of the $^{13}C\{^{1}H\}$-APT NMR spectrum of **3a**.

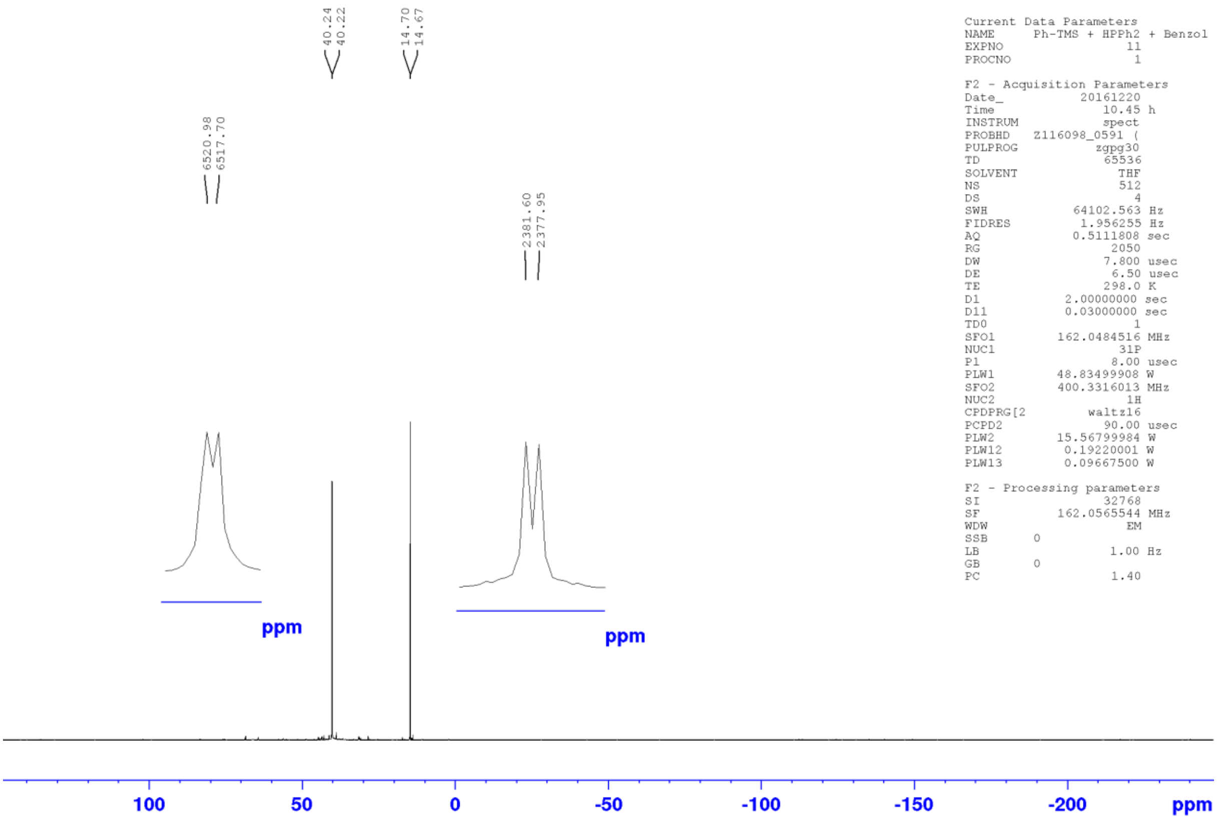

Figure S4. $^{31}P\{^{1}H\}$ NMR spectrum of **5**.

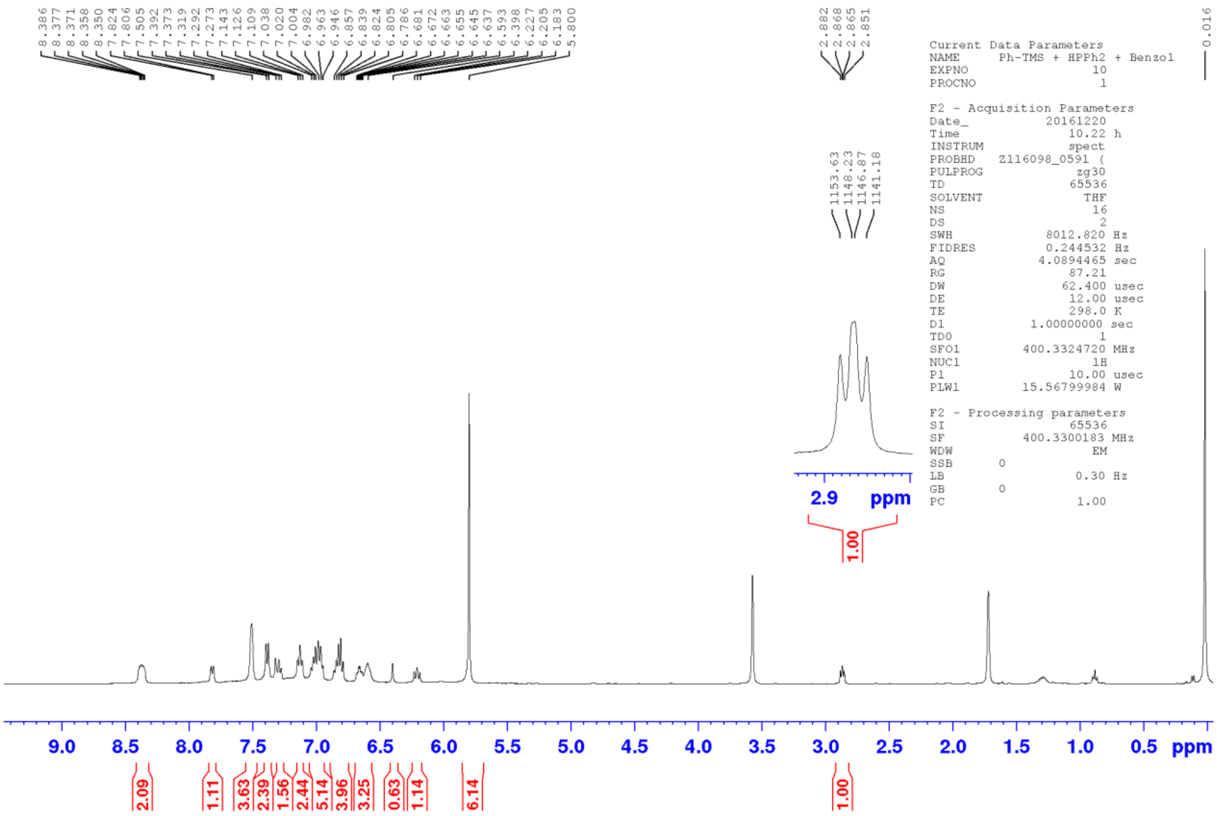

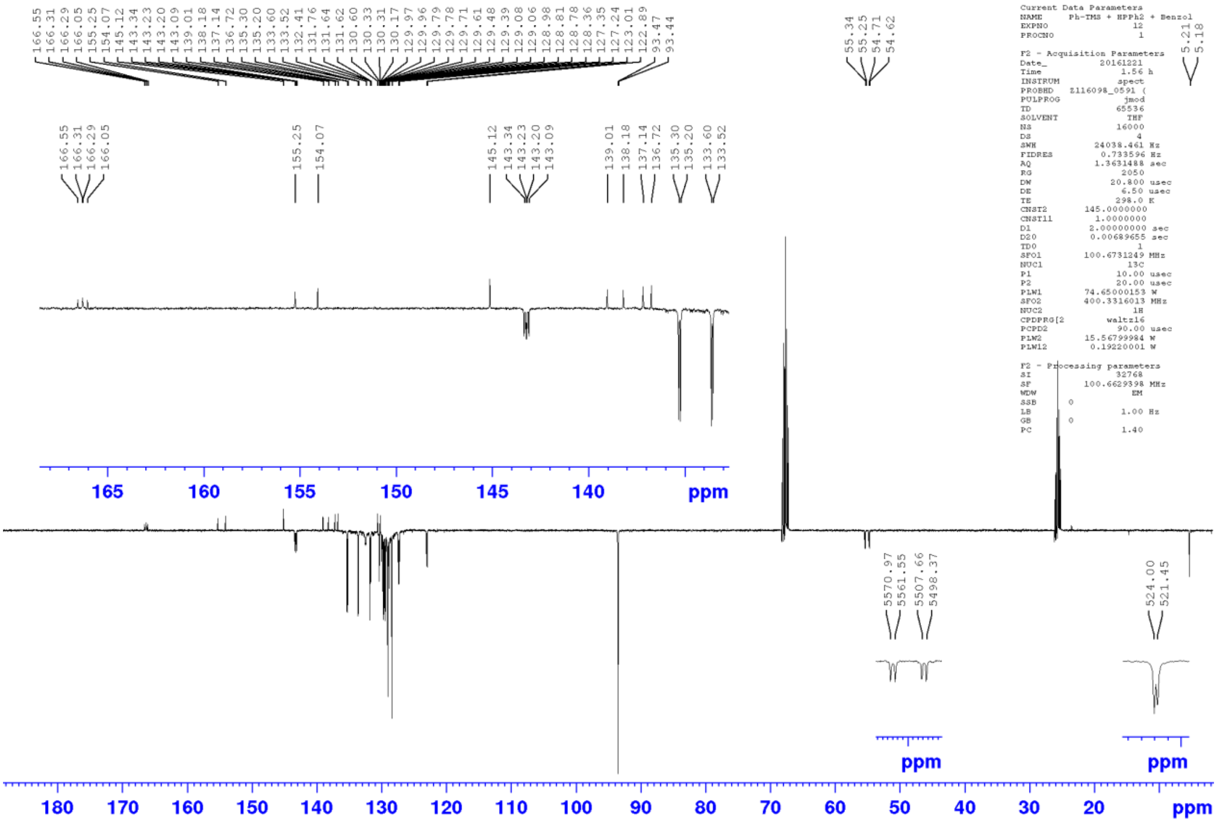

Figure S5. ^{1}H NMR (top) and $^{13}C\{^{1}H\}$-APT NMR (bottom) spectrum of **5**.

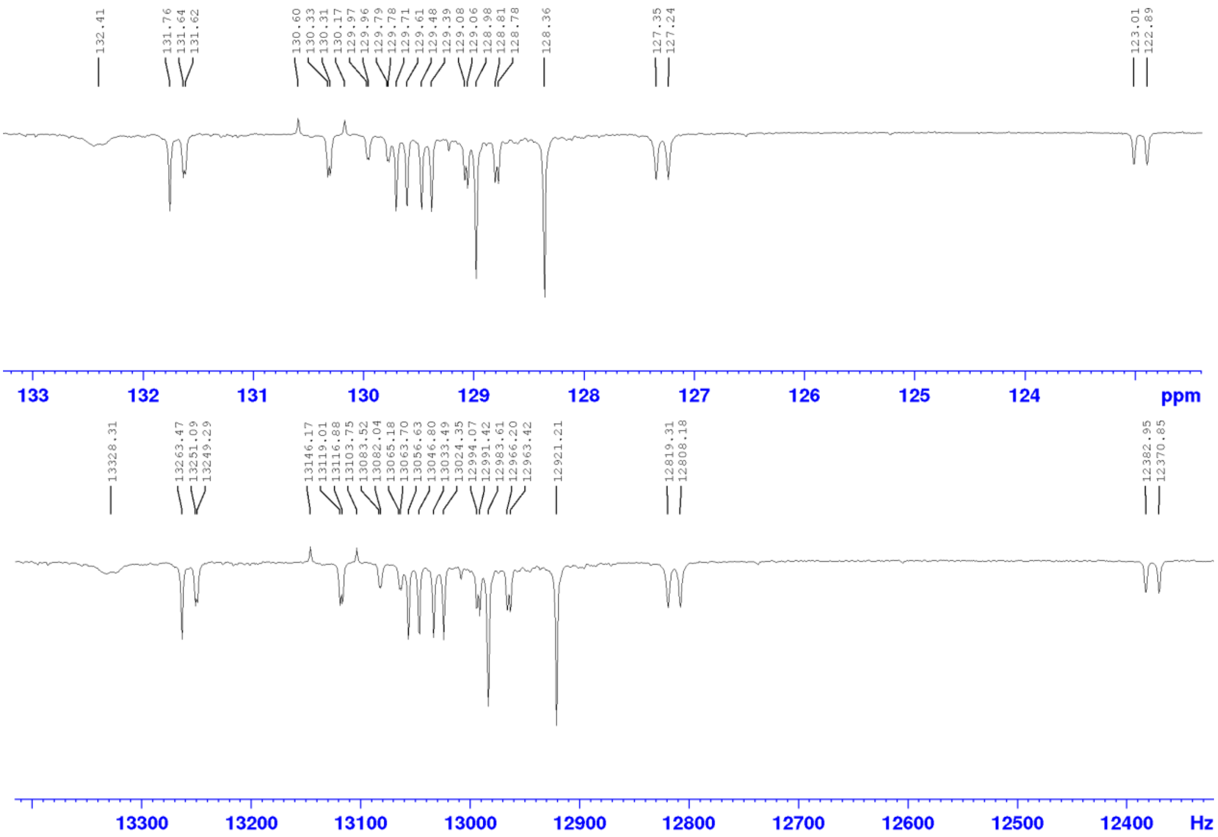

Figure S6. Section of the $^{31}C\{^{1}H\}$ NMR spectrum of **5**.

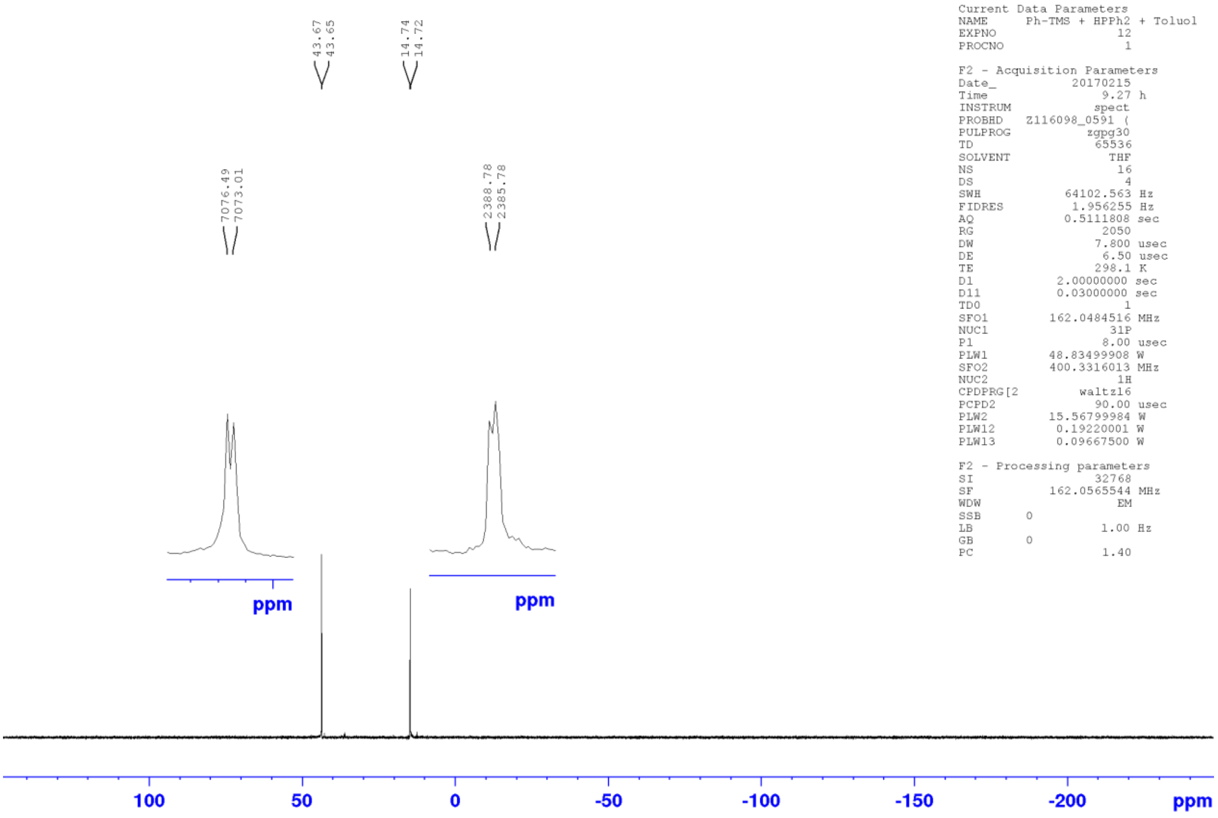

Figure S7. $^{31}P\{^{1}H\}$ NMR spectrum of **6**.

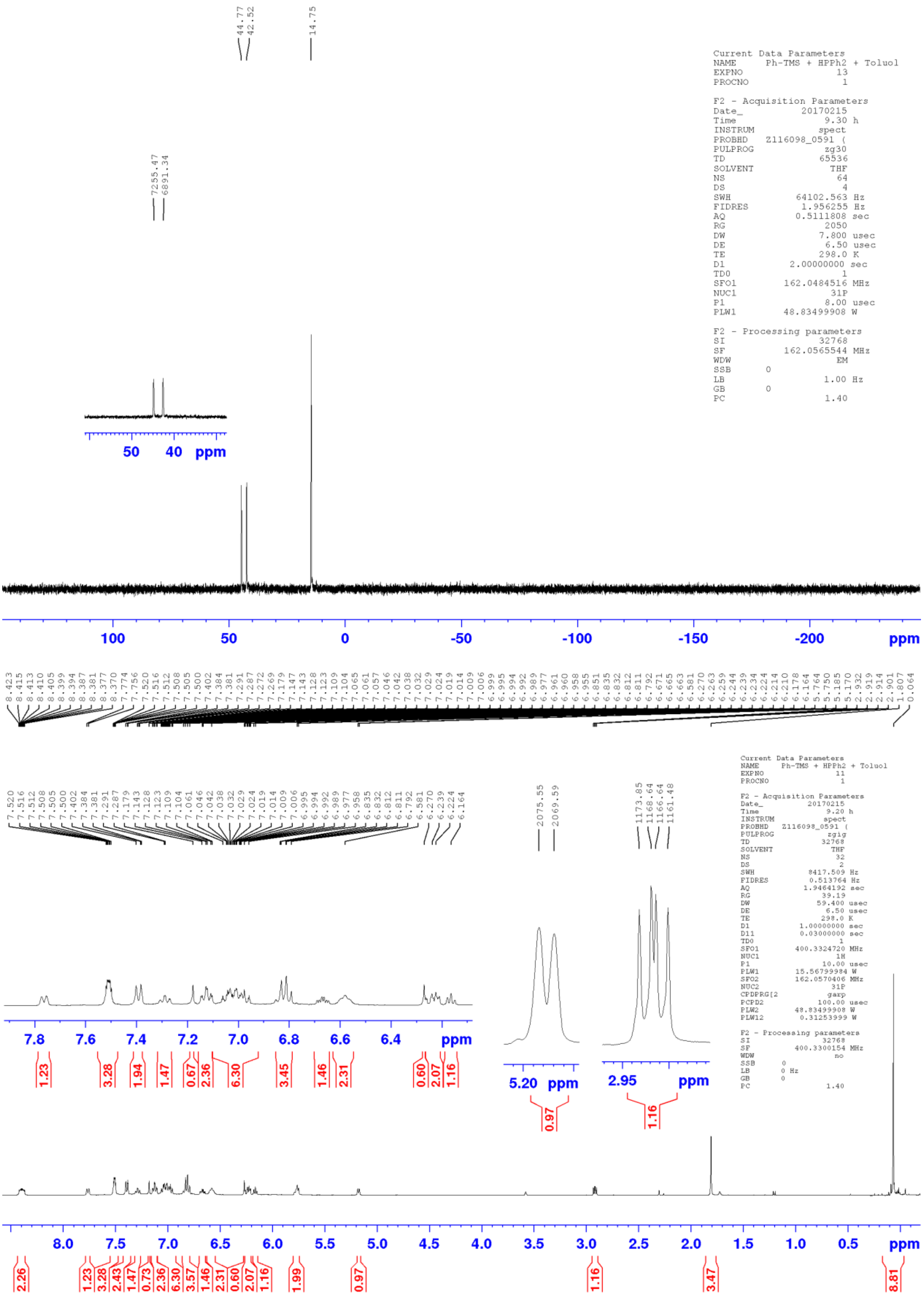

Figure S8. ^{31}P NMR (top) and ^{1}H NMR (bottom) spectrum of **6**.

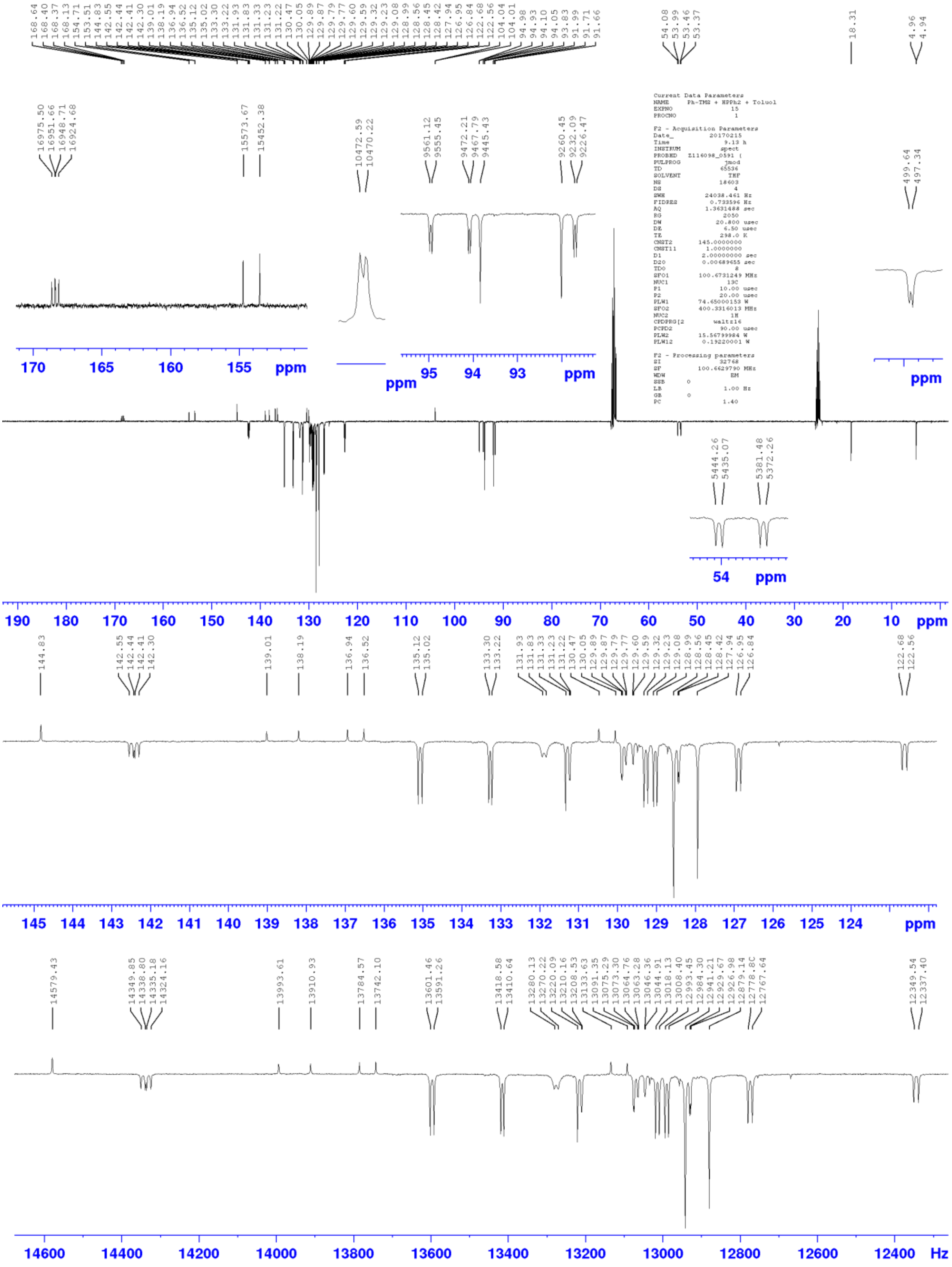

Figure S9. $^{13}C\{^1H\}$-APT NMR (top) and Section of the $^{13}C\{^1H\}$-APT NMR (bottom) spectrum of **6**.

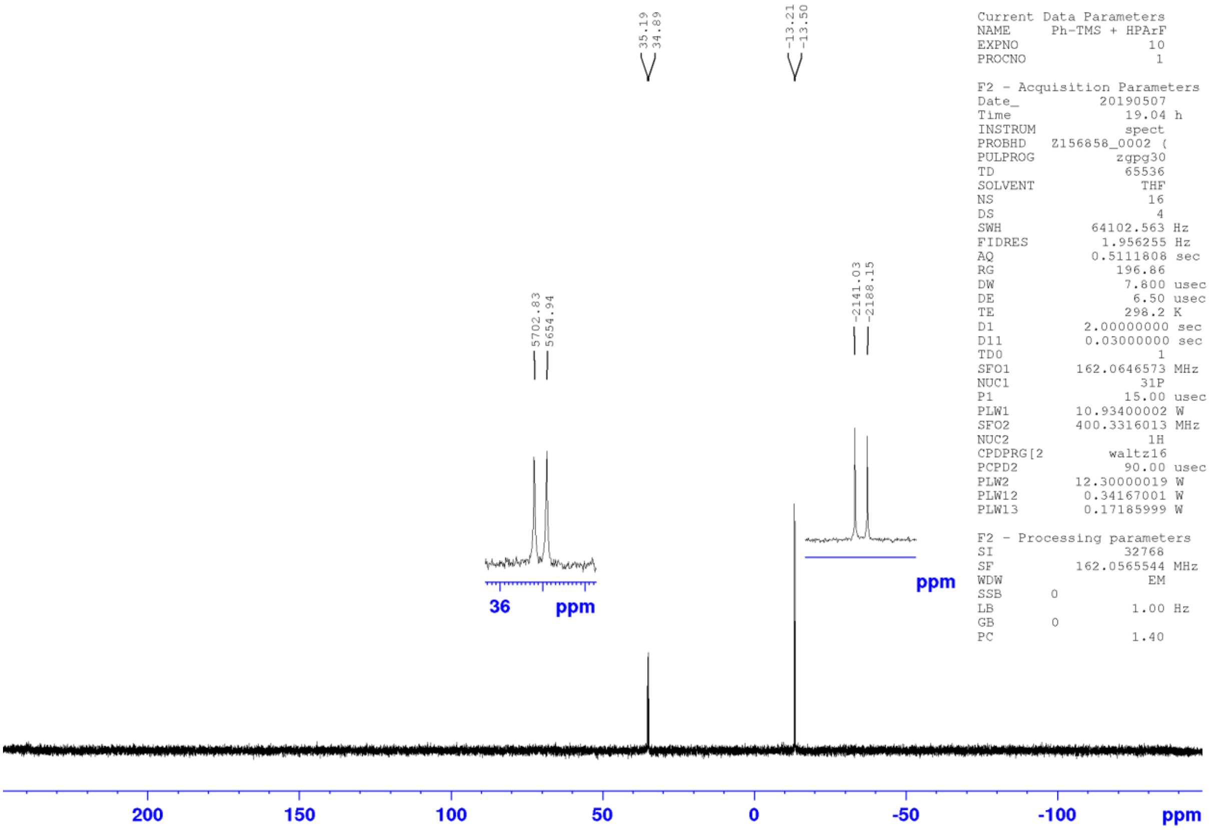

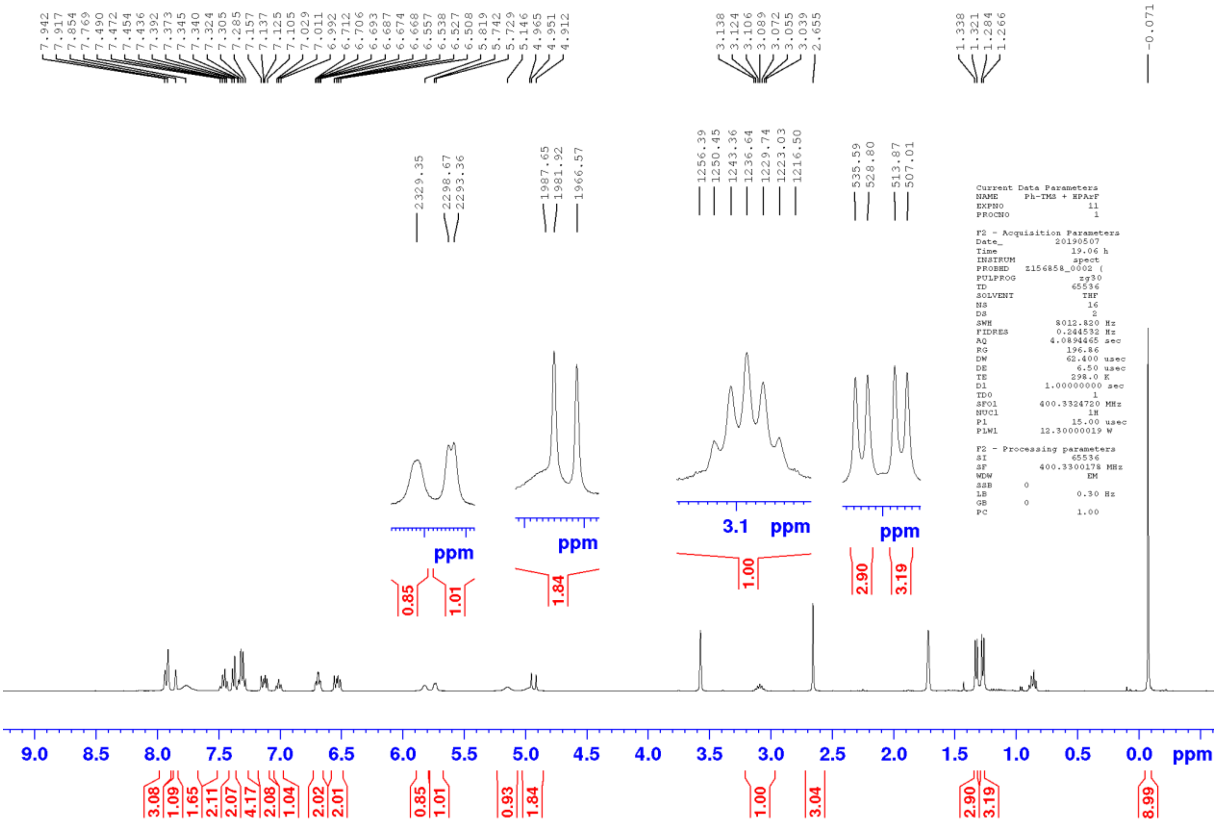

Figure S10. $^{31}P\{^{1}H\}$ NMR (top) and ^{1}H NMR (bottom) spectrum of **4b**.

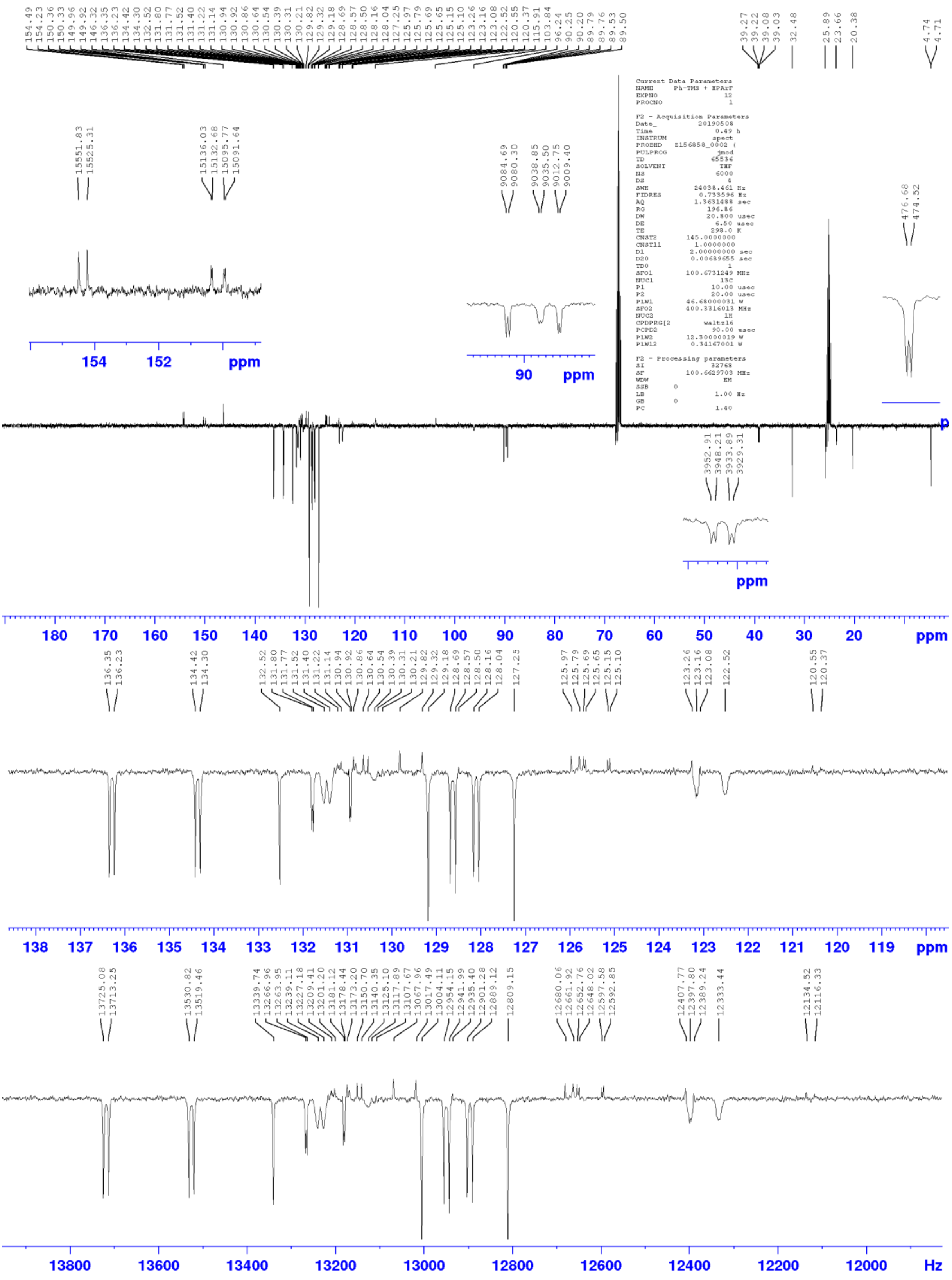

Figure S11. $^{13}C\{^{1}H\}$-APT NMR (top) and Section of the $^{13}C\{^{1}H\}$-APT NMR (bottom) spectrum of **4b**.

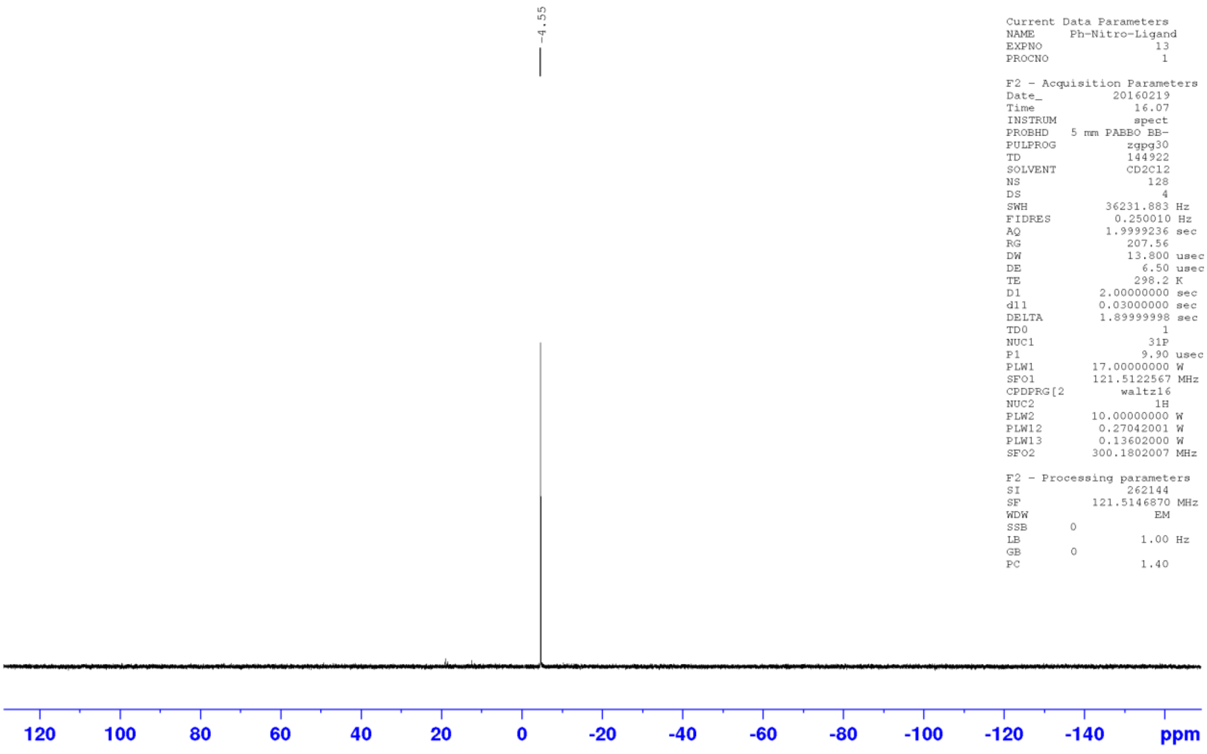

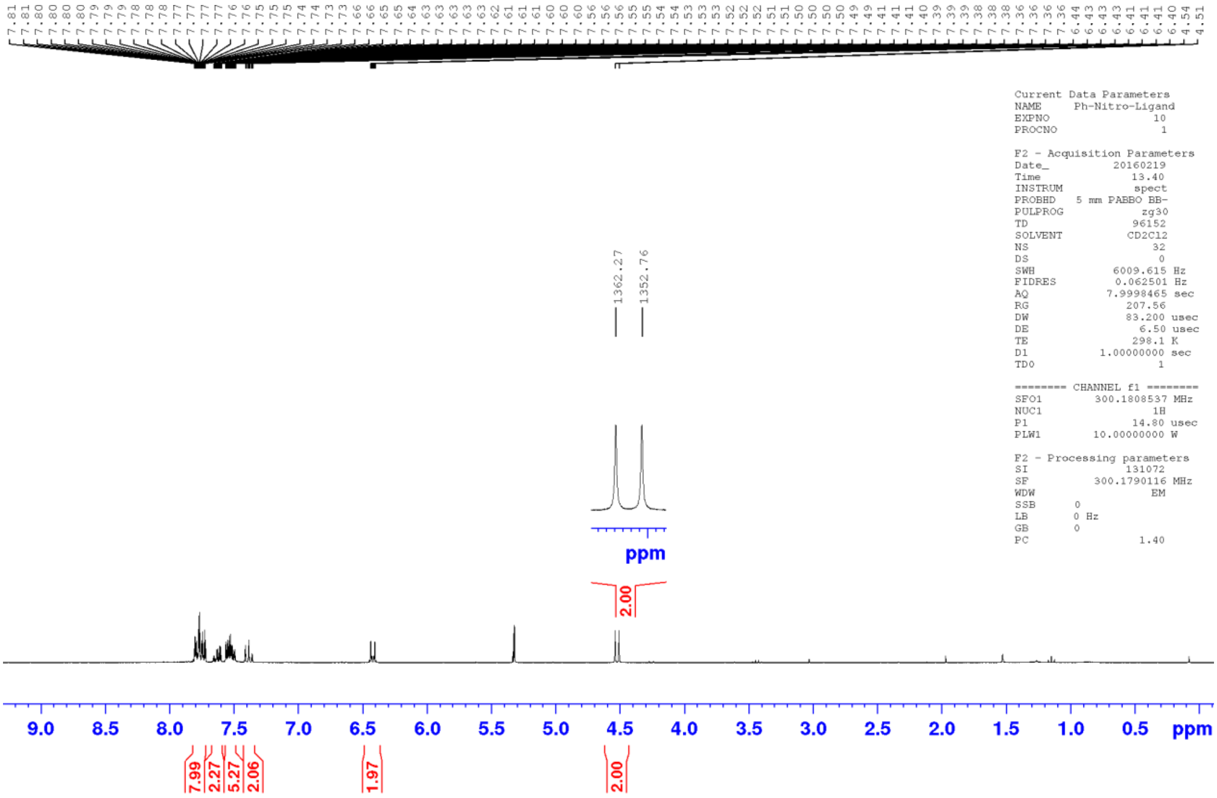

Figure S12. ${}^{31}P\{{}^{1}H\}$ NMR (top) and ${}^{1}H$ NMR (bottom) spectrum of **8**.

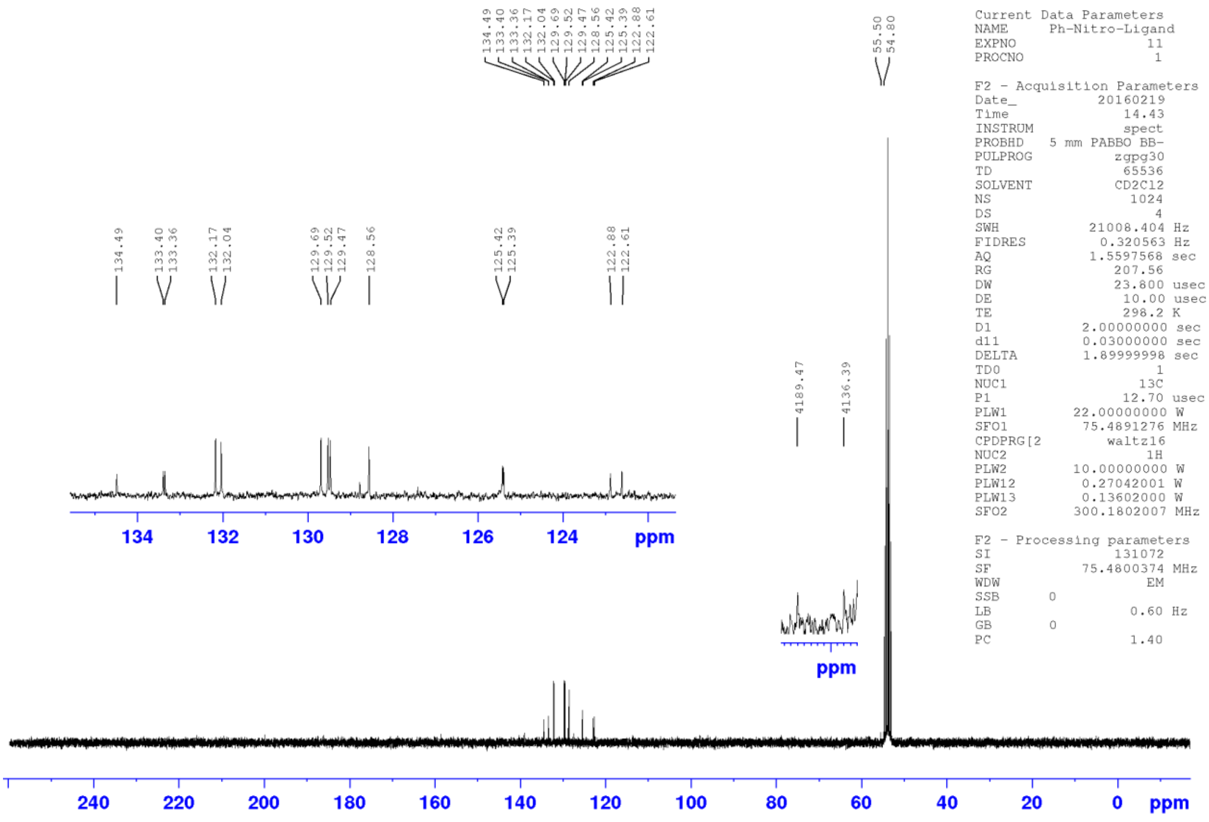

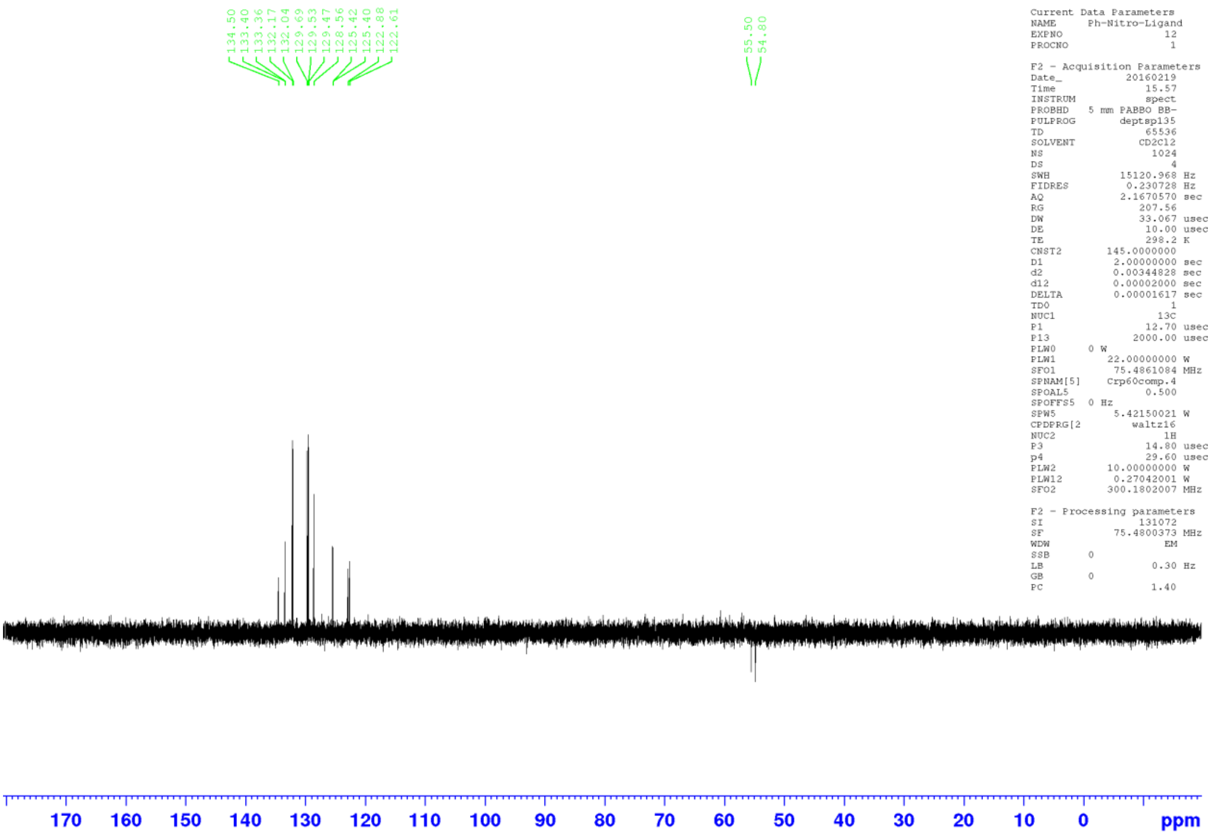

Figure S13. $^{13}C\{^1H\}$ NMR (top) and ^{13}C-DEPT-NMR (bottom) spectrum of **8**.

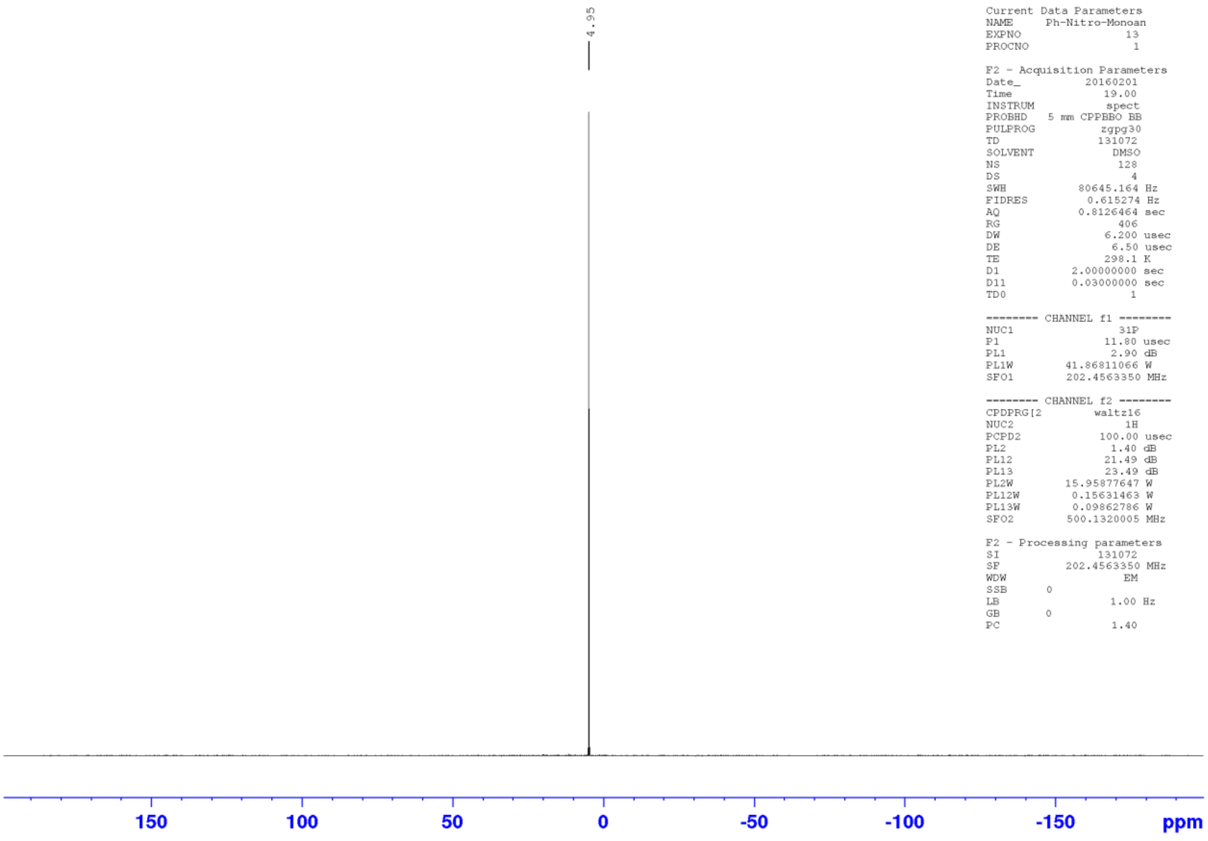

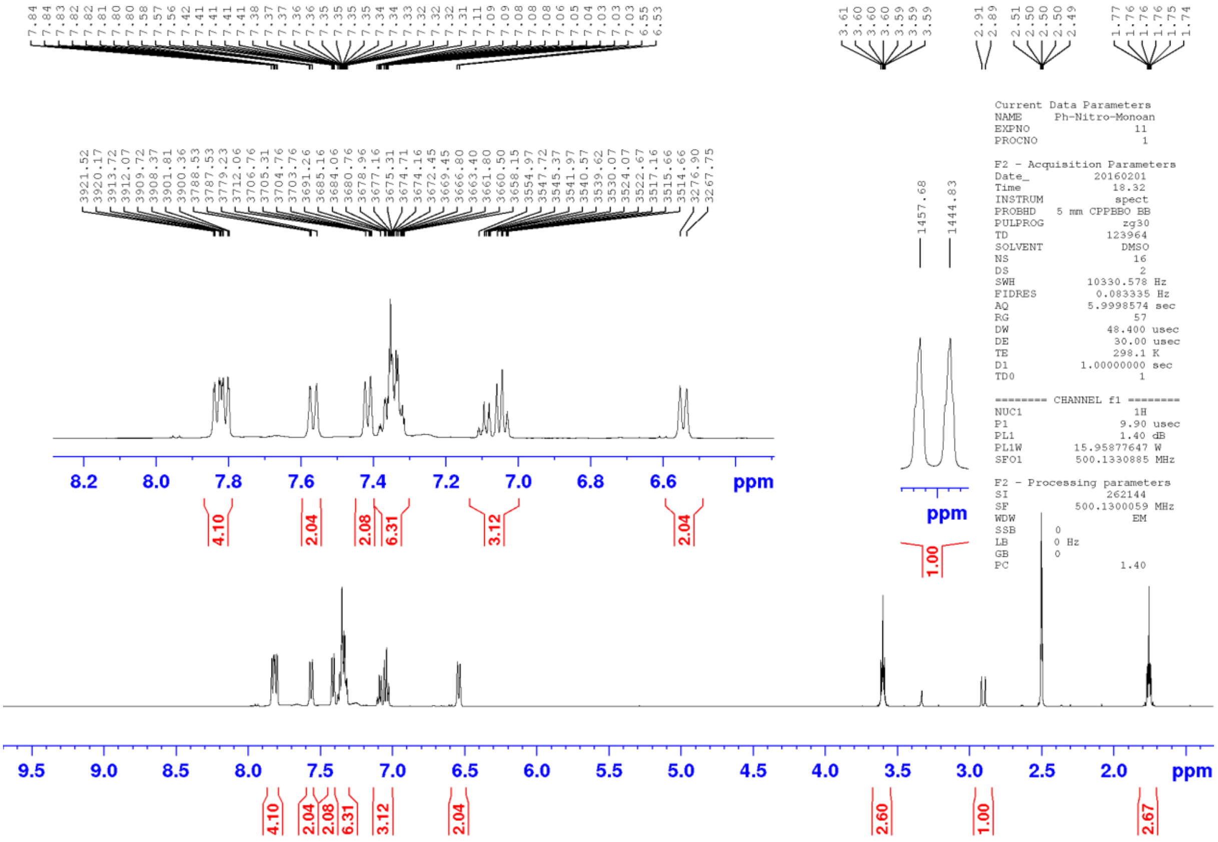

Figure S14. $^{31}P\{^{1}H\}$ NMR (top) and ^{1}H NMR (bottom) spectrum of **8-Na**.

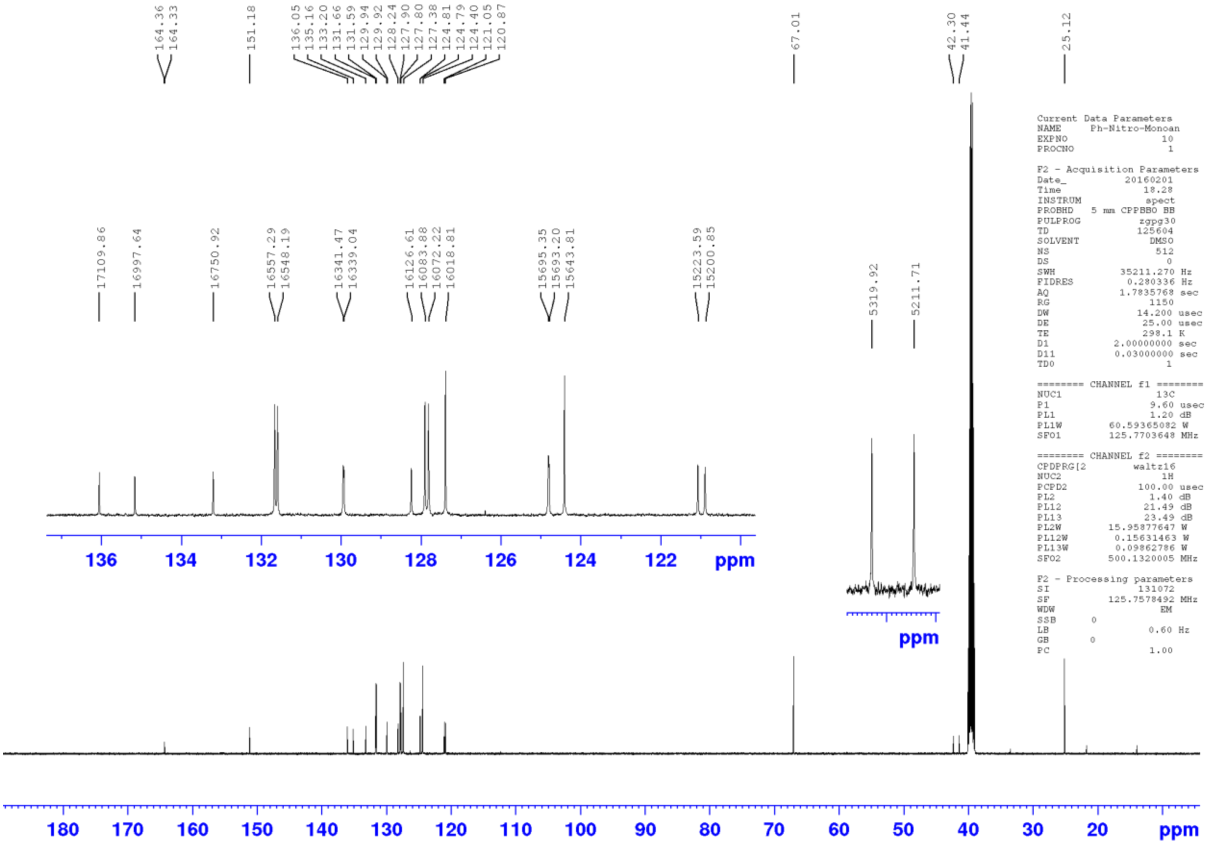

Figure S15. $^{13}C\{^{1}H\}$ NMR spectrum of **8-Na**.

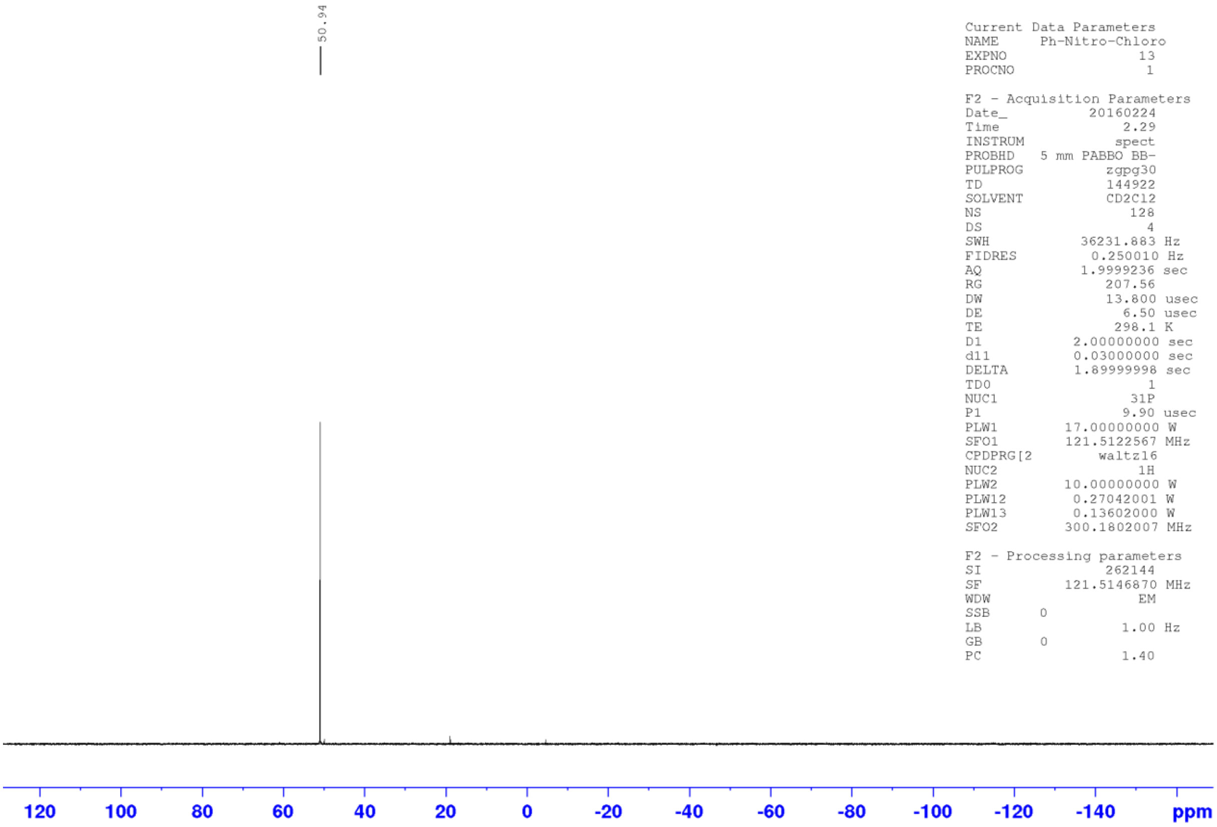

Figure S16. $^{31}P\{^{1}H\}$ NMR spectrum of **9**.

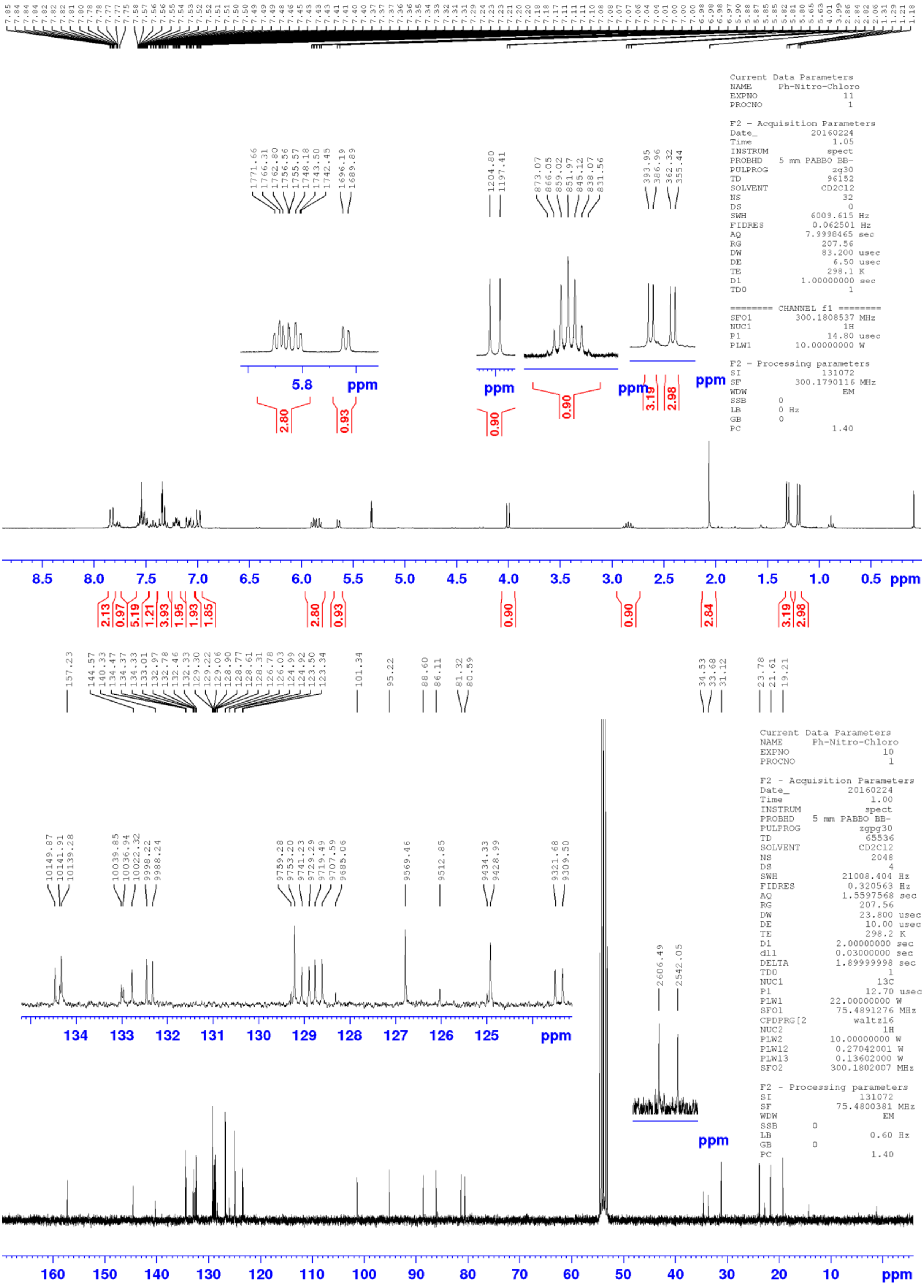

Figure S17. ^{1}H NMR (top) and ^{31}C{^{1}H} NMR (bottom) spectrum of **9**.

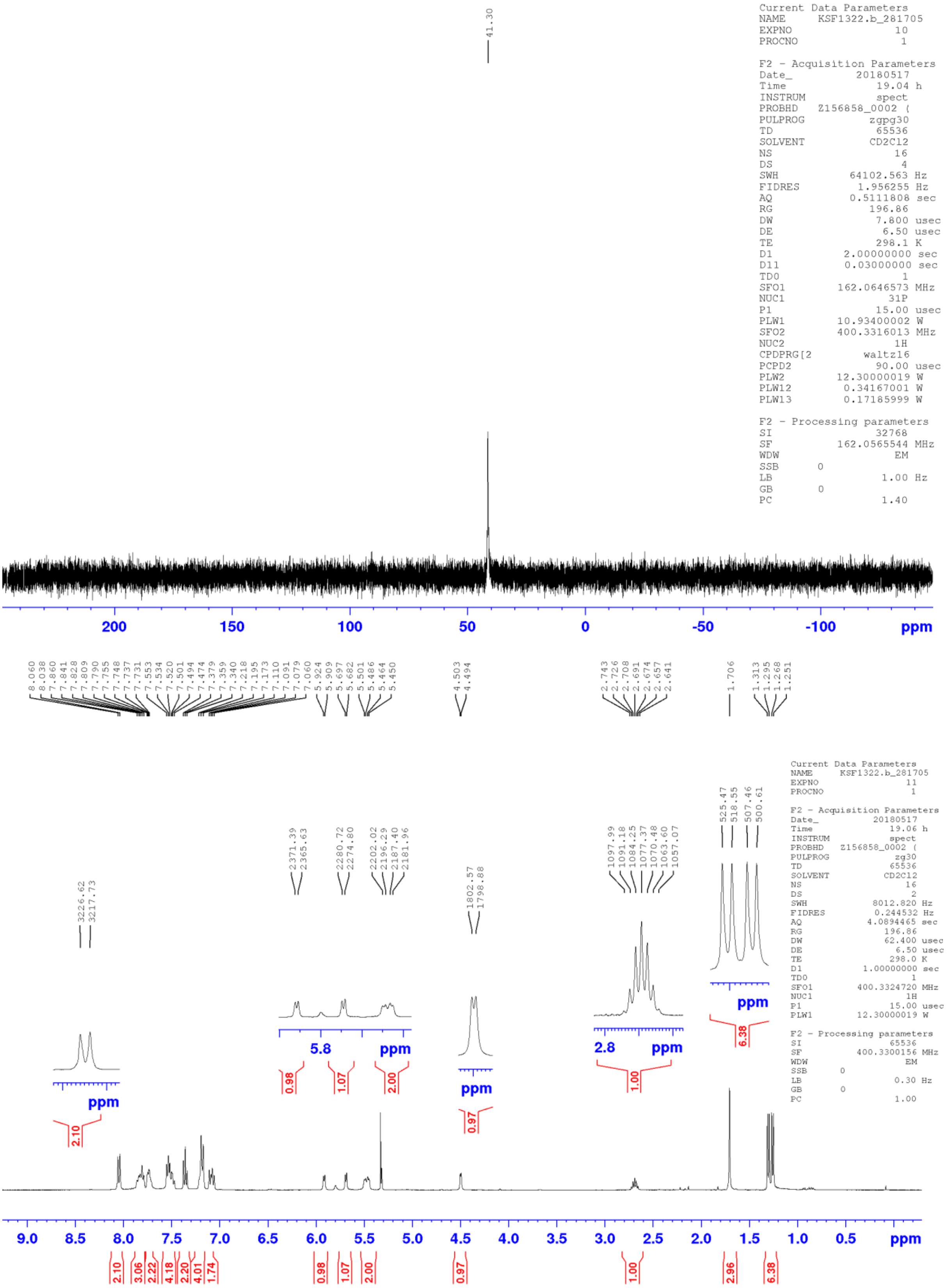

Figure S18. $^{31}P\{^{1}H\}$ NMR (top) and ^{1}H NMR (bottom) spectrum of **10**.

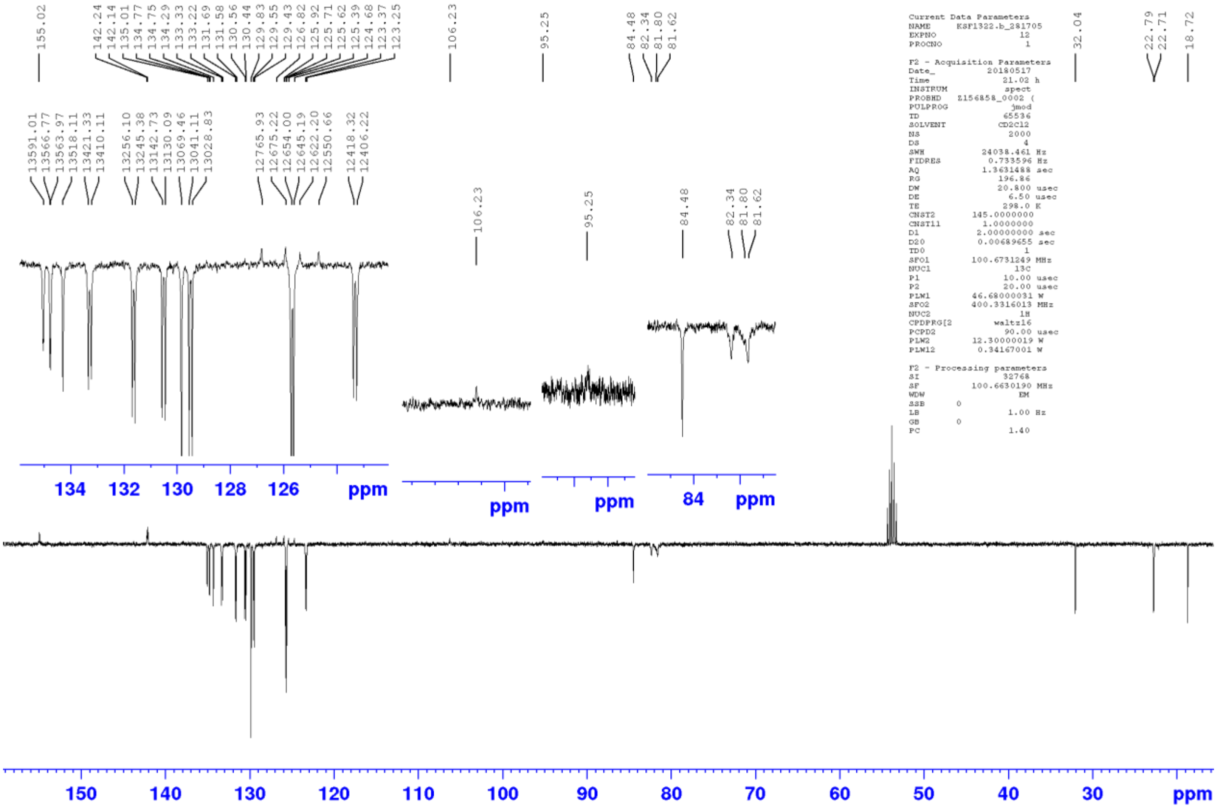

Figure S19. $^{13}C\{^{1}H\}$-APT NMR (bottom) spectrum of **10**.

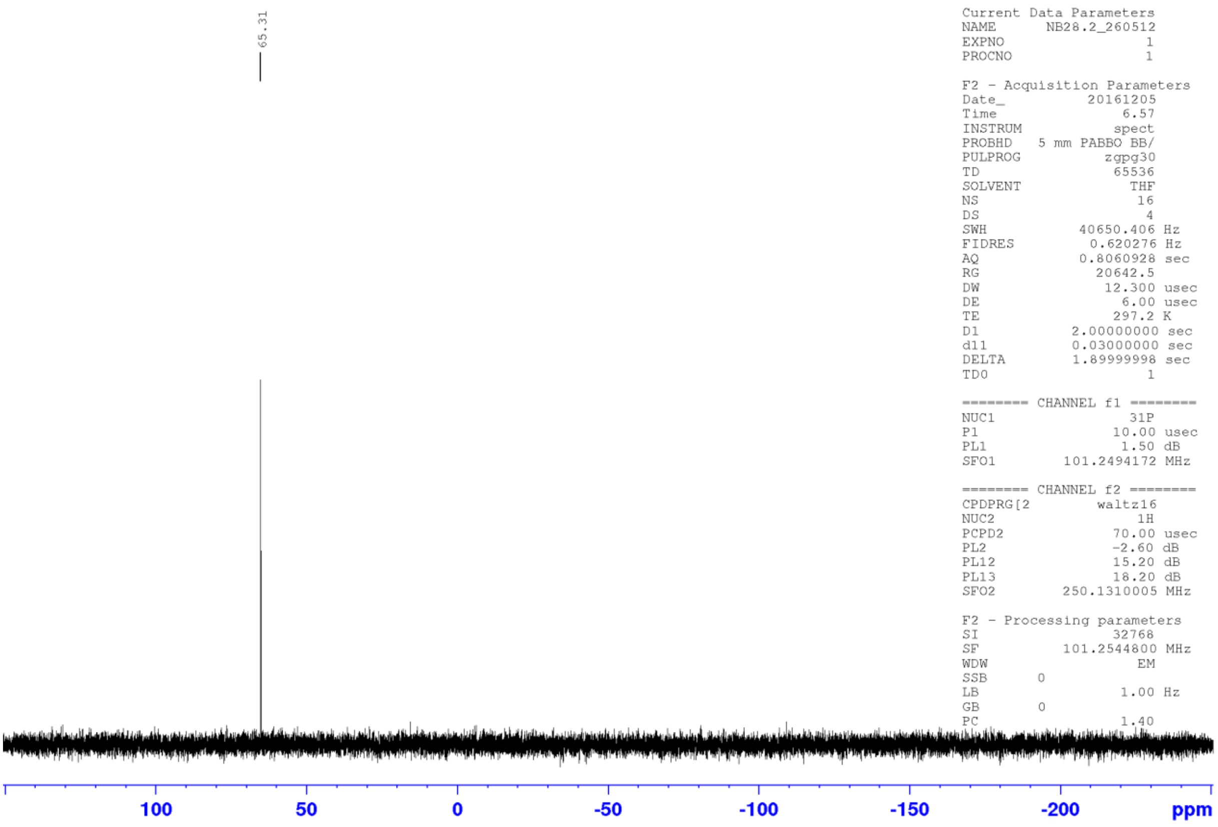

Figure S20. $^{31}P\{^{1}H\}$ NMR (bottom) spectrum of **2b**.

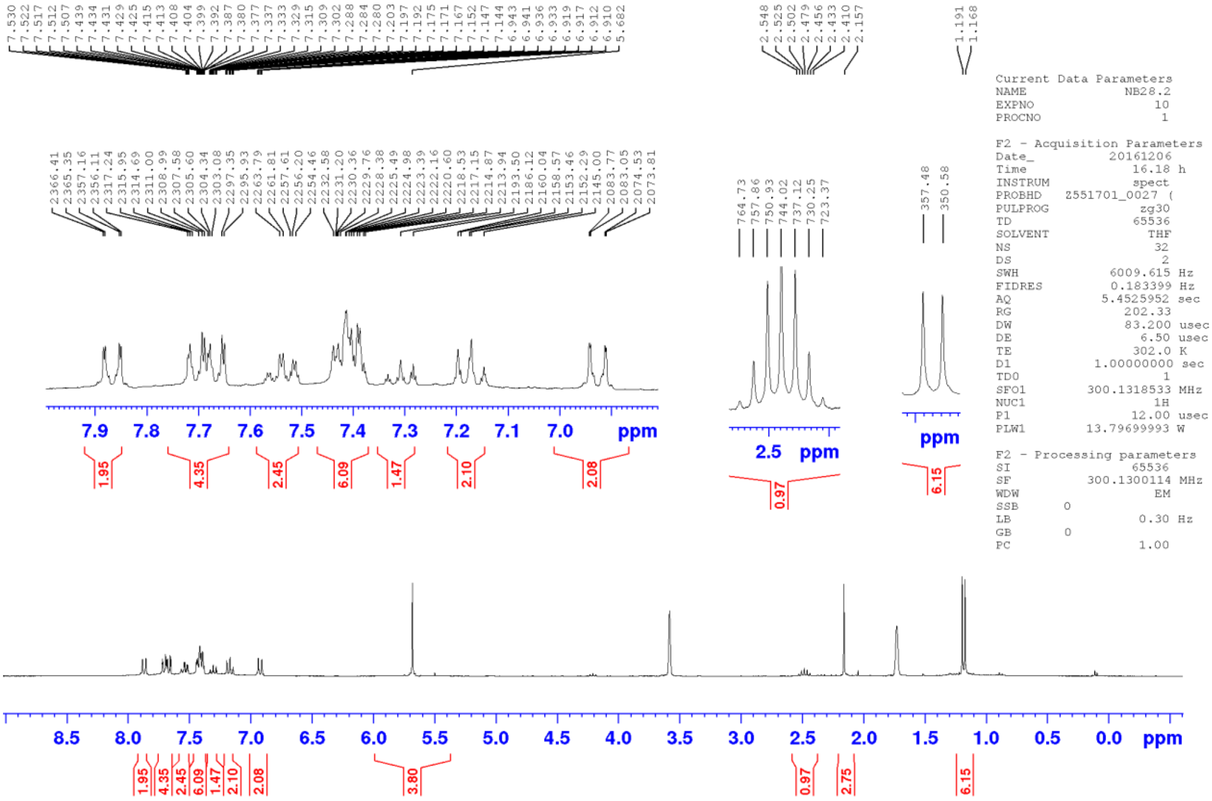

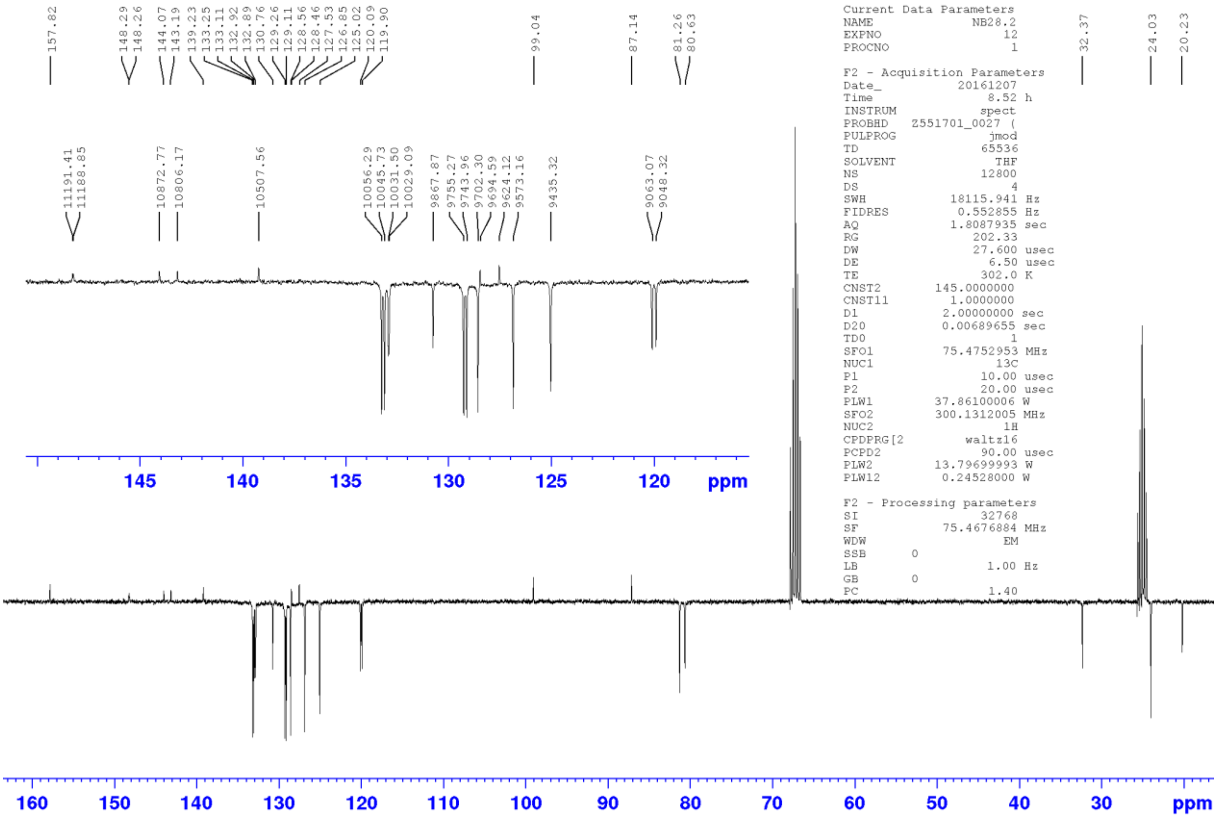

Figure S21. ^{1}H NMR (top) and $^{13}C\{^{1}H\}$-APT NMR (bottom) spectrum of **2b**.

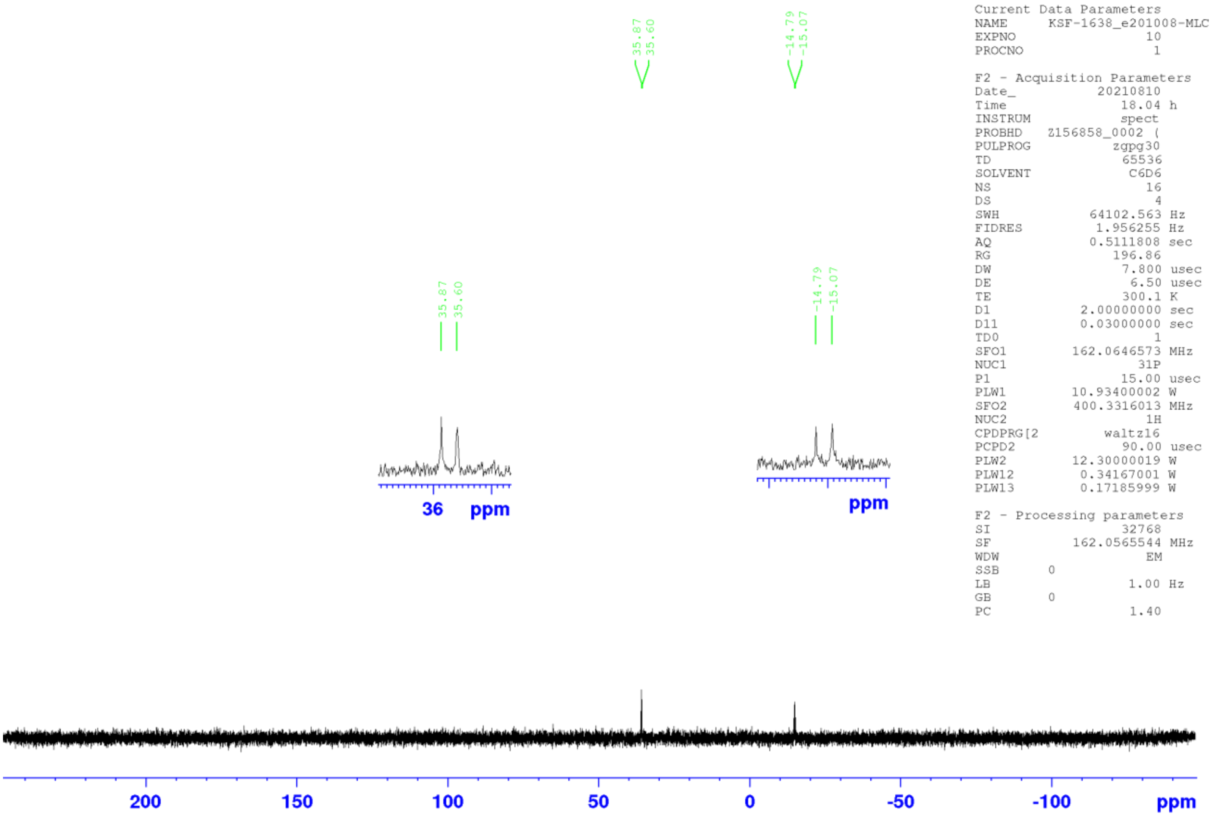

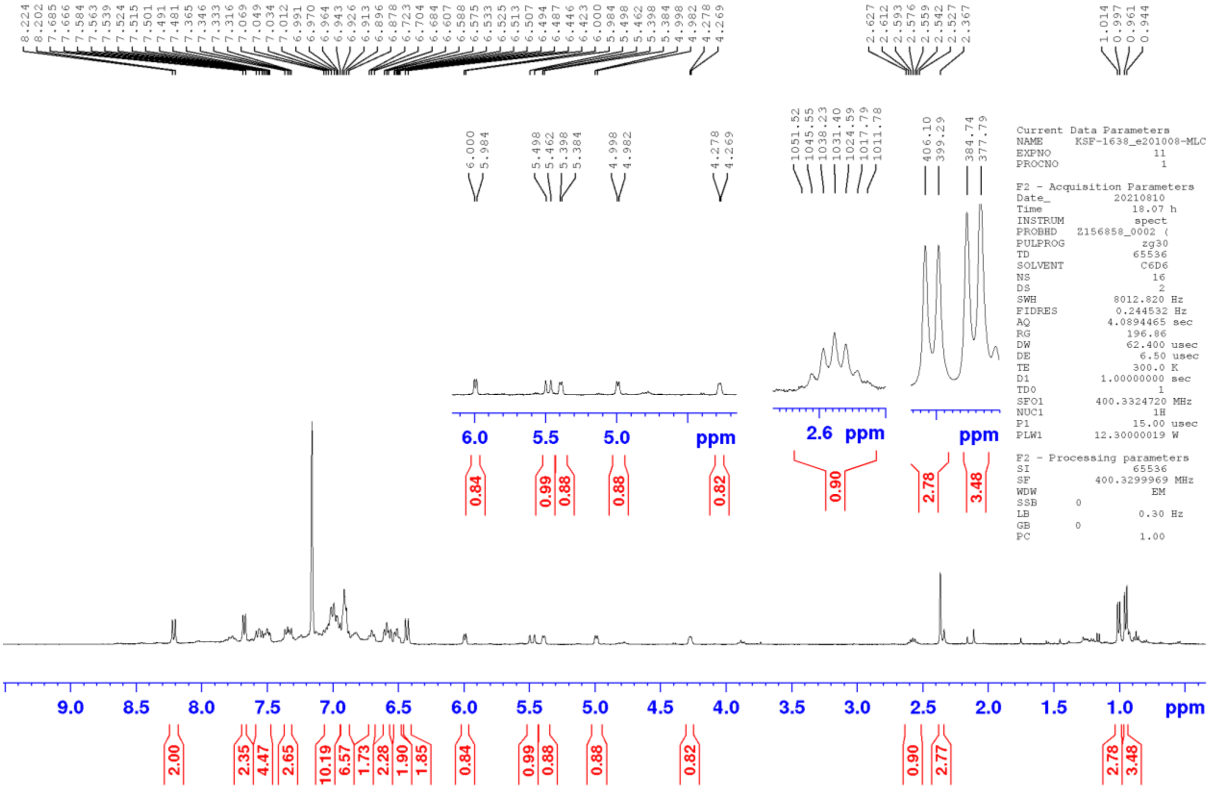

Figure S22. $^{31}P\{^{1}H\}$-APT NMR (top) and ^{1}H NMR (bottom) spectrum of **4c**.

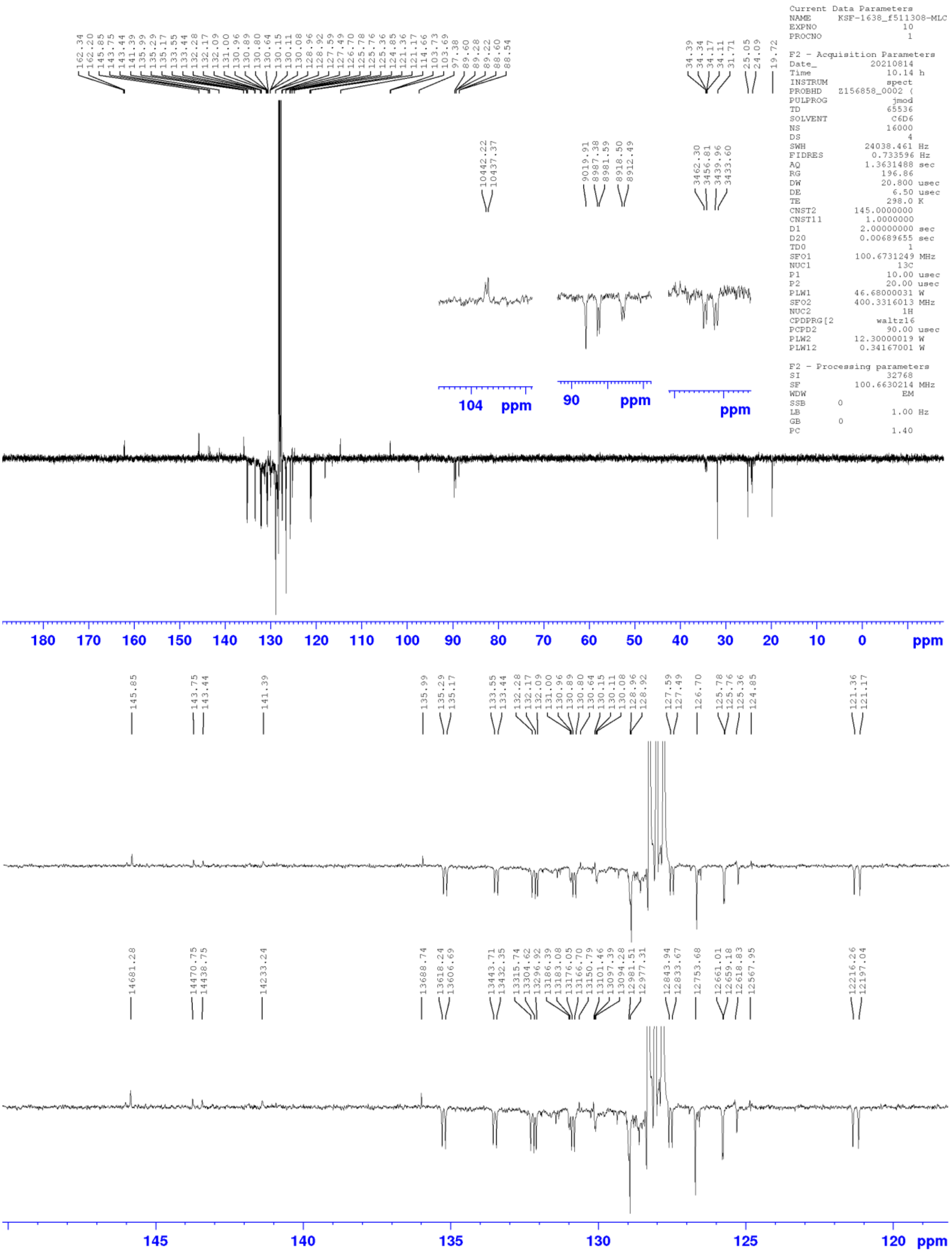

Figure S23. $^{13}C\{^{1}H\}$-APT NMR spectrum of **4c** (top) and section of the $^{13}C\{^{1}H\}$-APT NMR spectrum of **4c** (bottom).

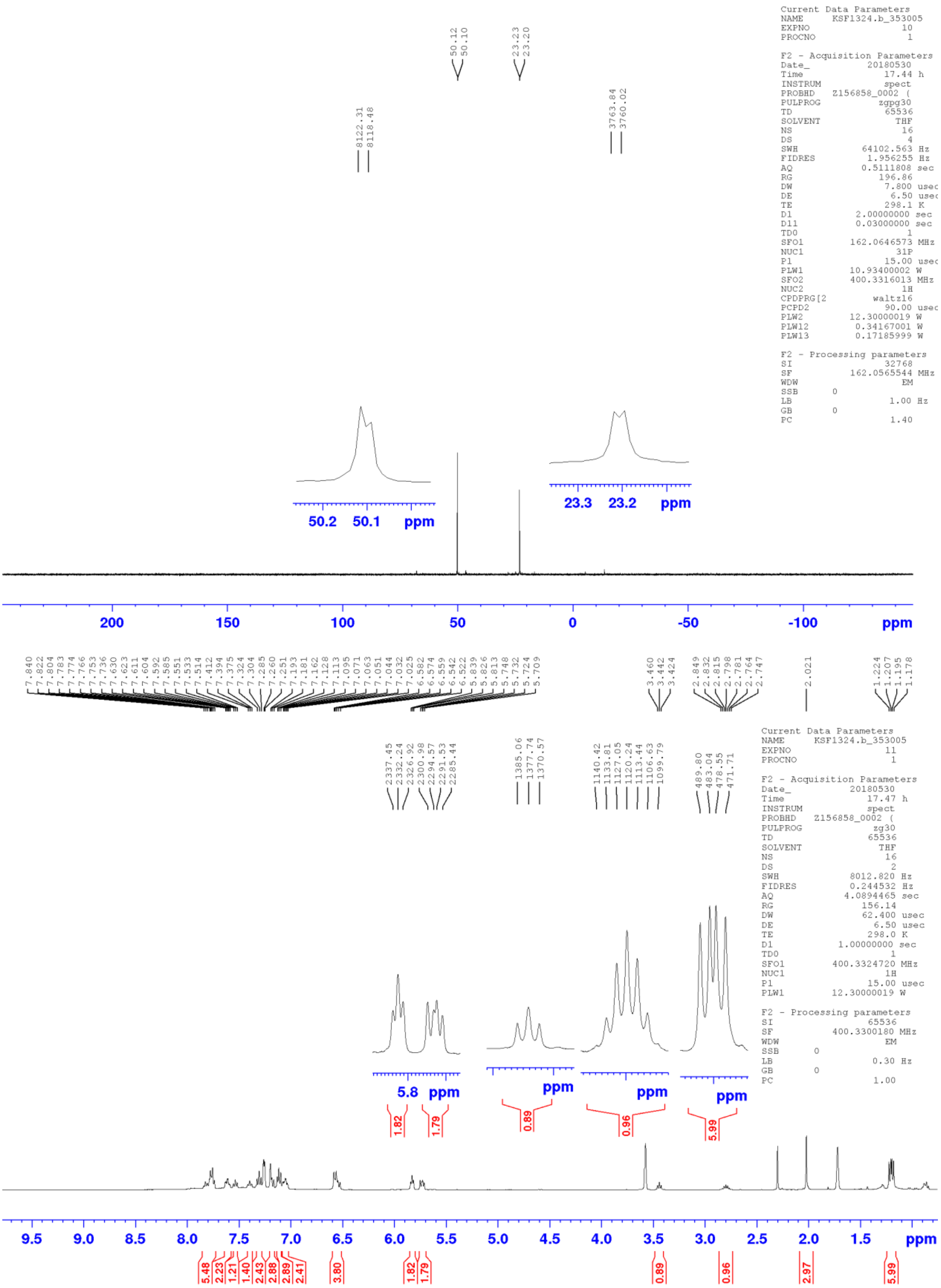

Figure S24. $^{31}P\{^{1}H\}$ NMR (top) and ^{1}H NMR (bottom) spectrum of **11a**.

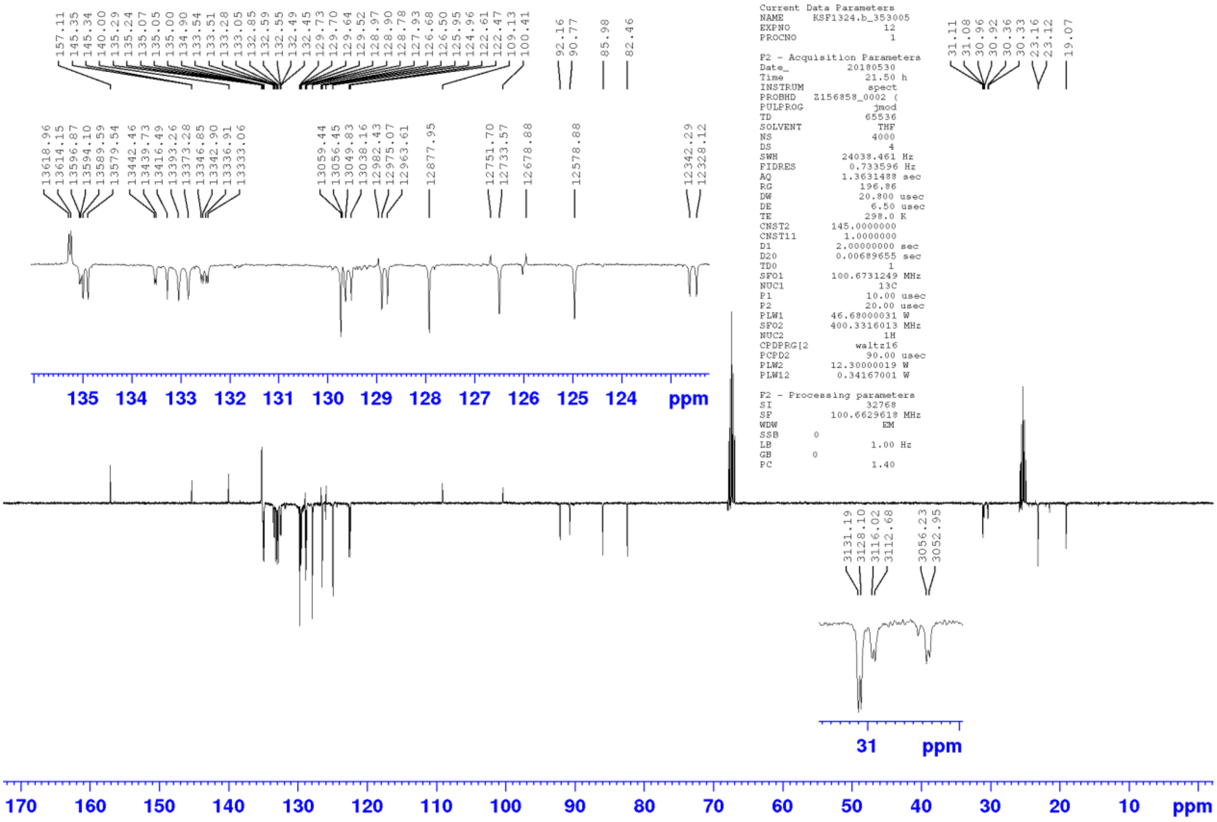

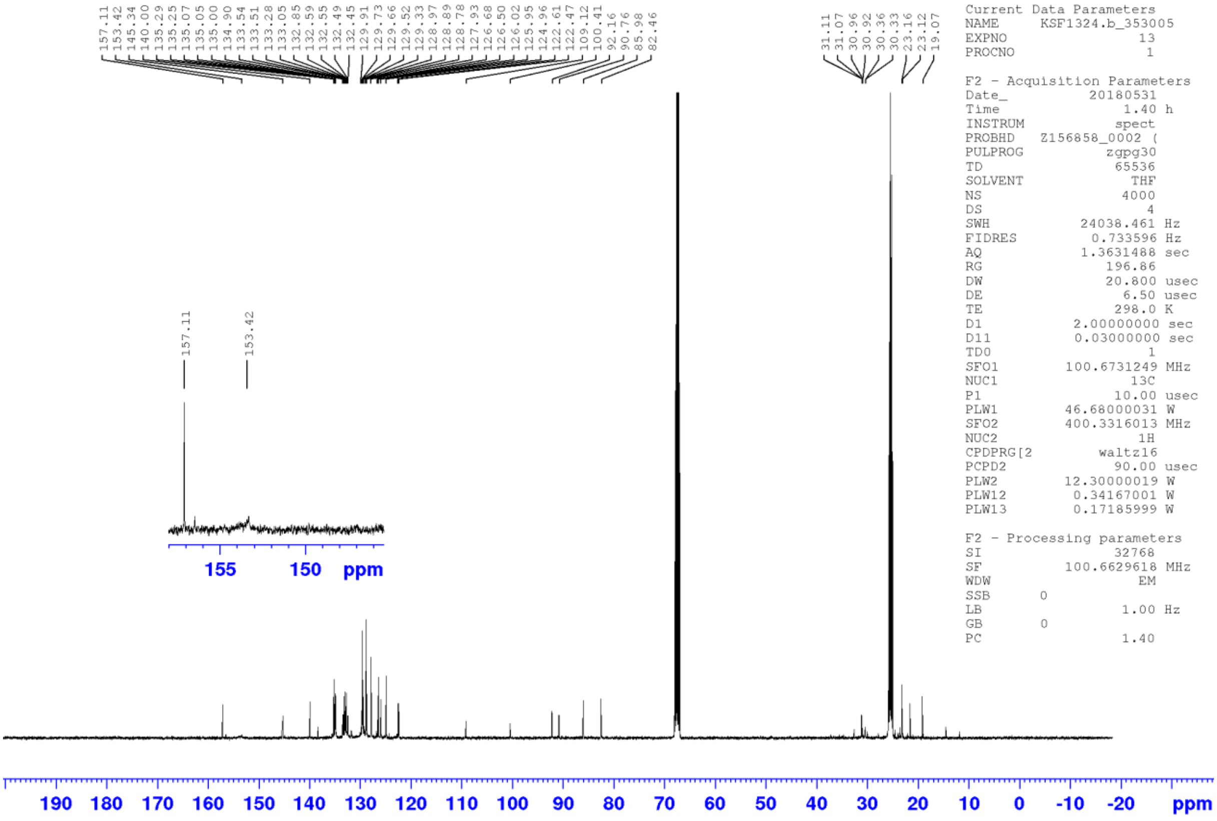

Figure S25. $^{13}C\{^1H\}$-APT NMR (top) and $^{13}C\{^1H\}$ NMR (bottom) spectrum of **11a**.

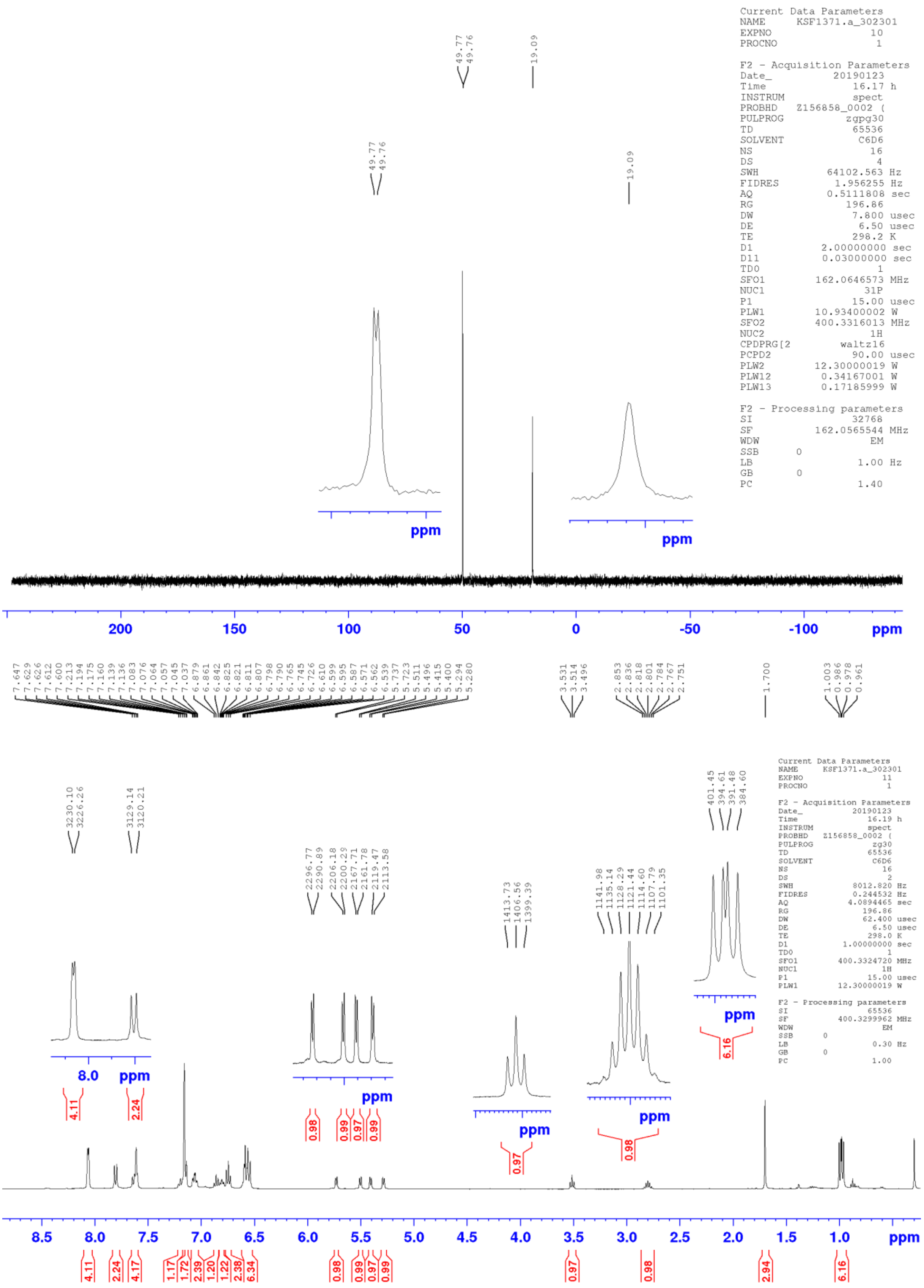

Figure S26. $^{31}P\{^{1}H\}$ NMR (top) and ^{1}H NMR (bottom) spectrum of **11b**.

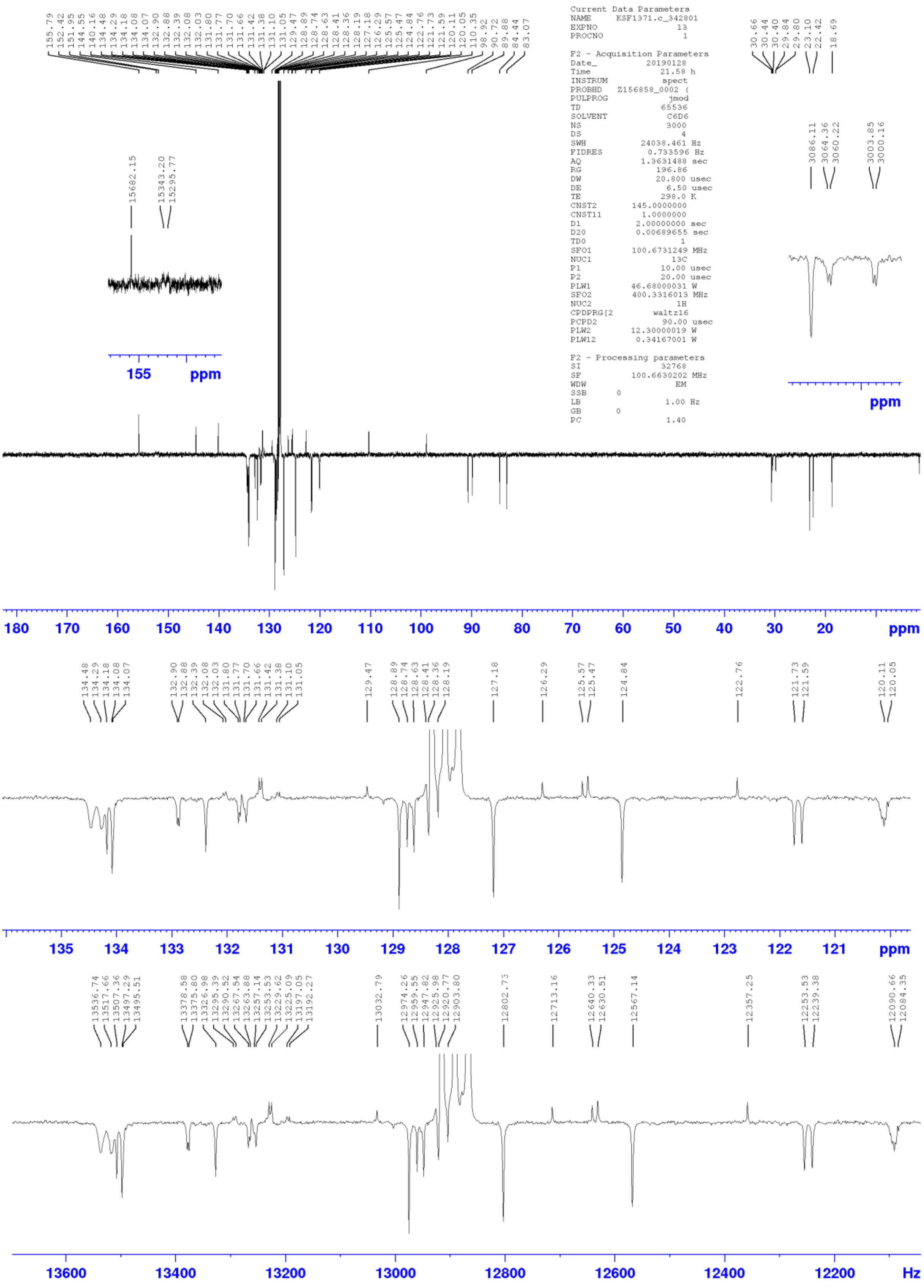

Figure S27. $^{13}C\{^1H\}$-APT NMR (top) and Section of the $^{13}C\{^1H\}$-APT NMR (bottom) spectrum of **11b**.

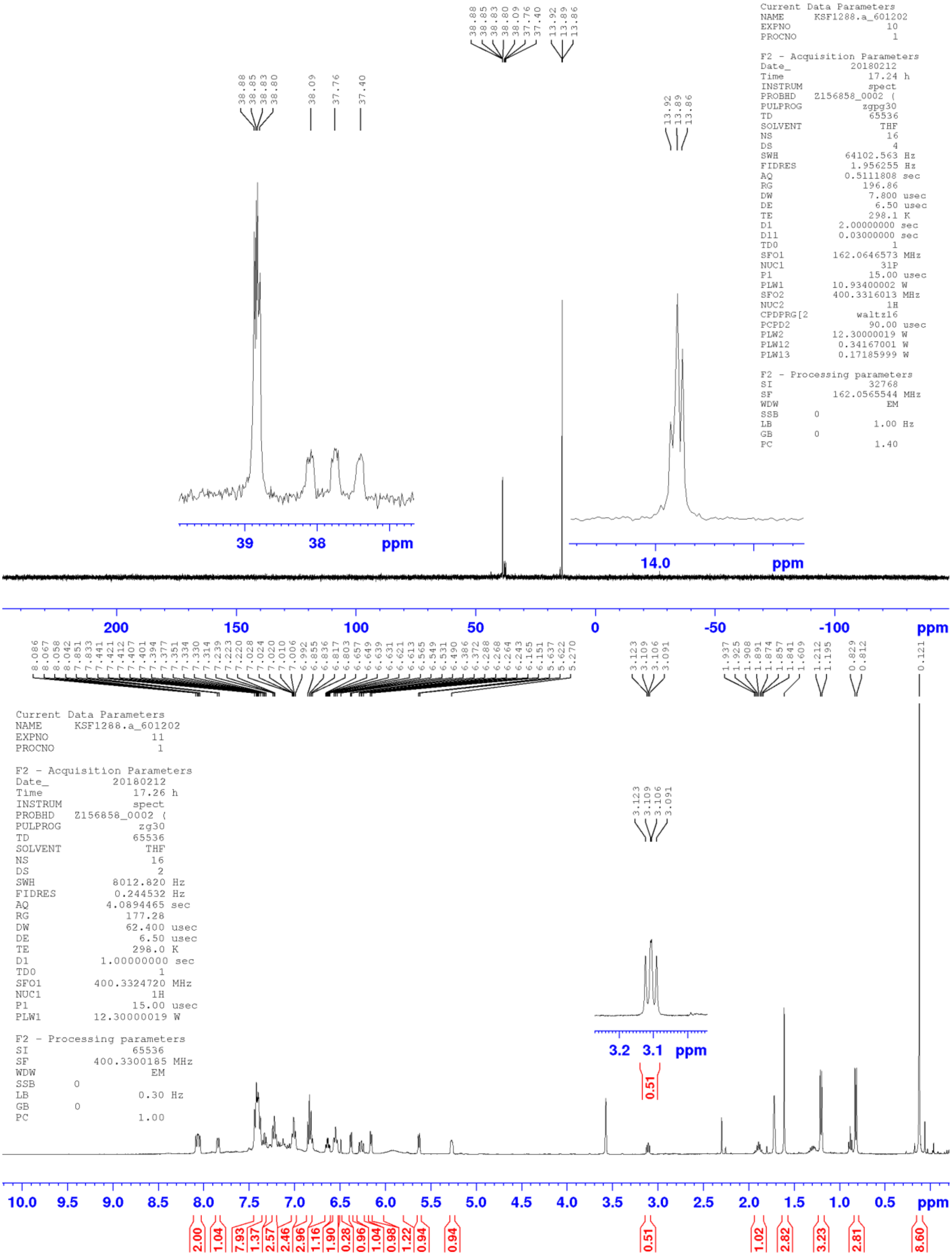

Figure S28. $^{31}P\{^{1}H\}$ NMR spectrum (top) and ^{1}H NMR spectrum (bottom) of the reaction of **2a** with Ph_2PD.

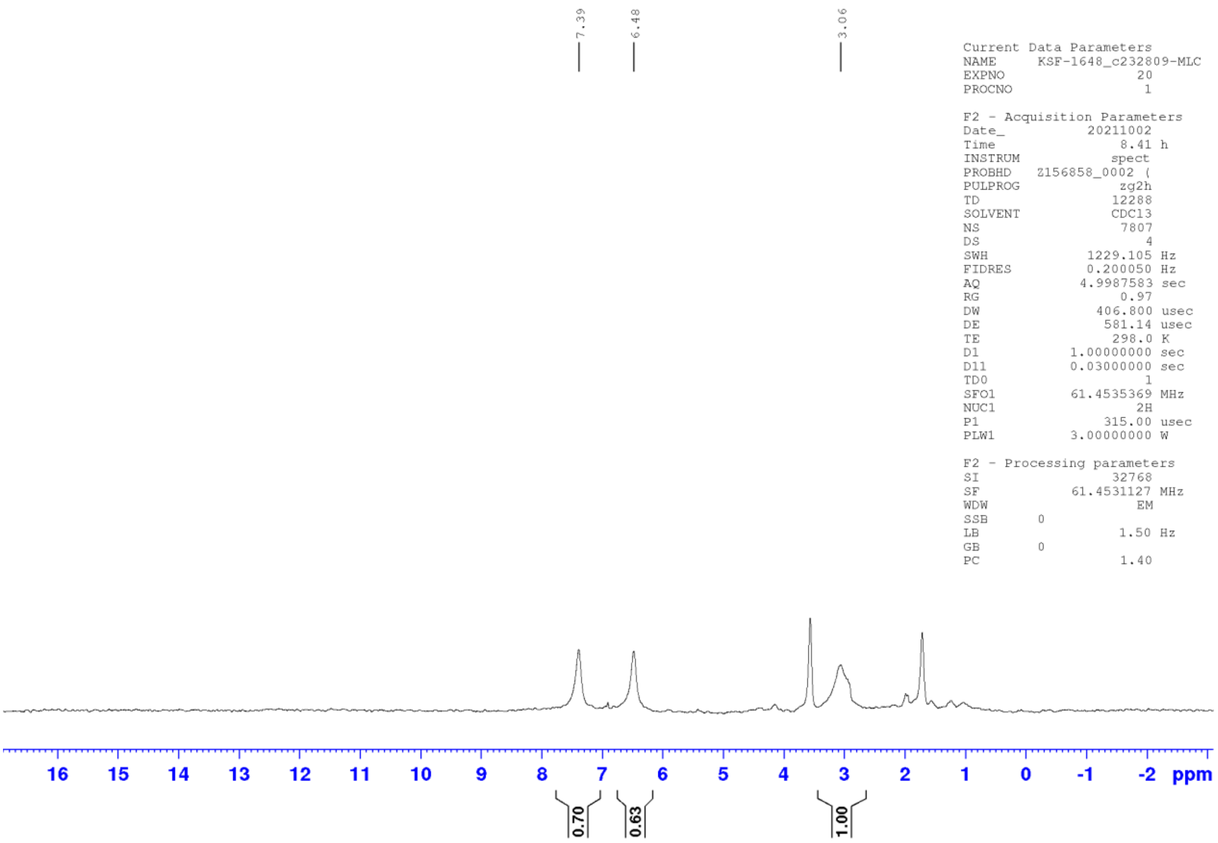

Figure S29. ^{2}H NMR spectrum of the reaction of **2a** with Ph2PD in THF (signals at 1.79 and 3.58 correspond to the natural occurring partly deuterated THF).

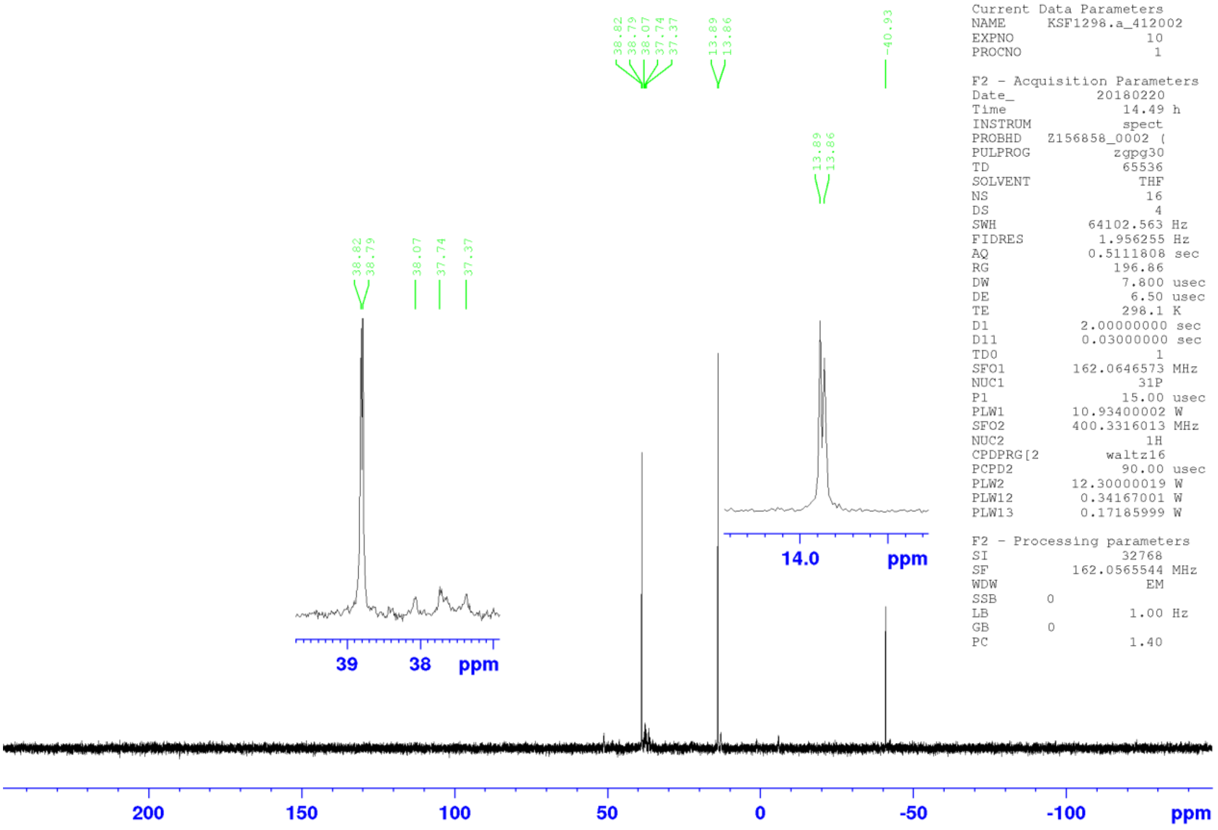

Figure S30. $^{31}P\{^{1}H\}$ NMR spectrum of the reaction of **3a** with Ph_2PD.

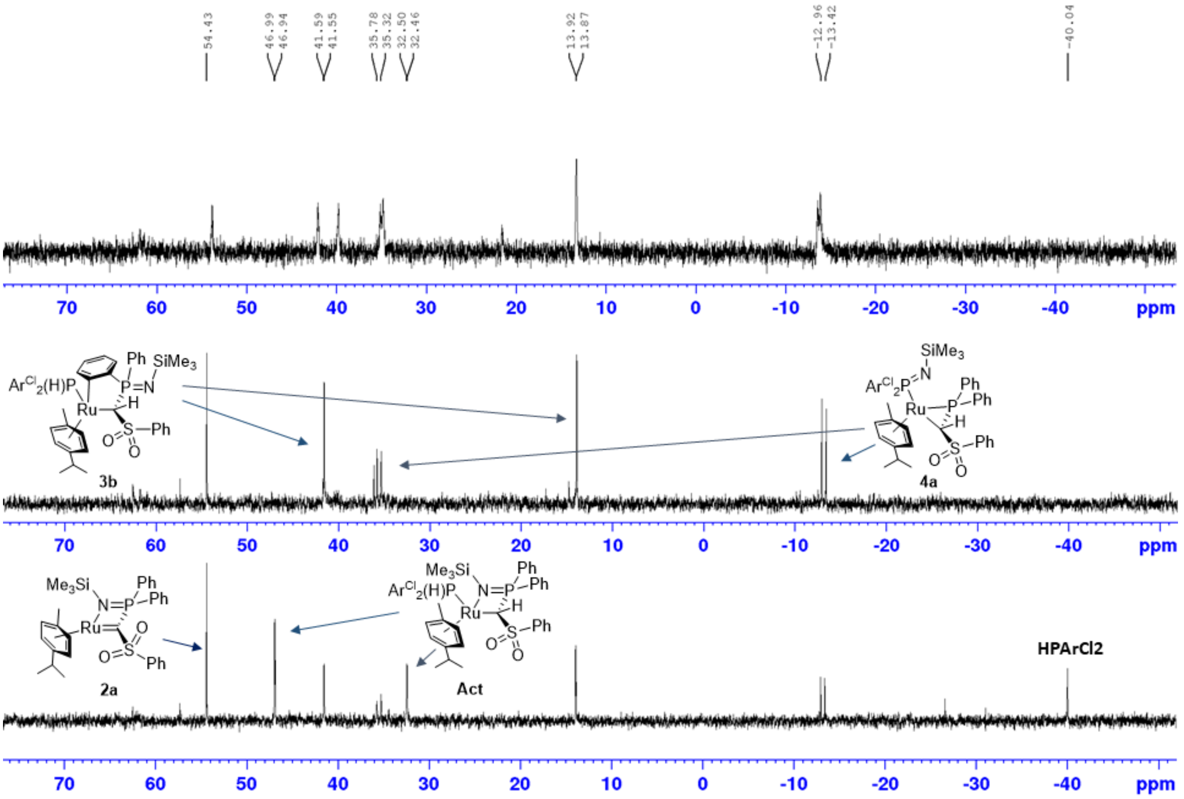

Figure S31. $^{31}P\{^1H\}$ NMR spectrum of the reaction of **3a** with $HPAr^{Cl}_2$ after 5 minutes (bottom), after 16 hours (middle) and ^1H coupled ^{31}P NMR spectrum (top).

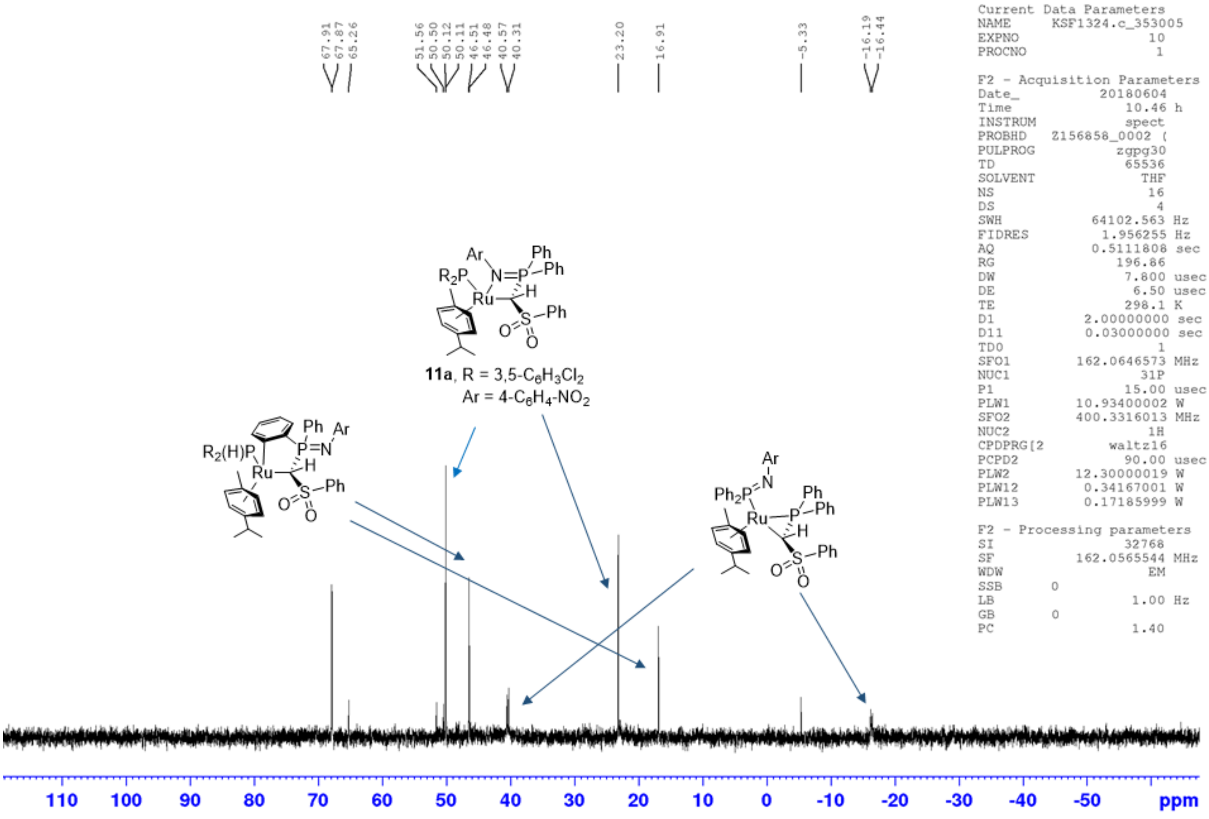

Figure S32. $^{31}P\{^1H\}$ NMR spectrum of **11a** after 4 days in solution.

3. Crystal structure determination

3.1 General

Data collection of the compounds was conducted with an Oxford Synergy, Bruker APEX$_2$-CCD, or Oxford SuperNova (Cu-µsource, Atlas). The structures were solved using direct methods, refined with the Shelx software package and expanded using Fourier techniques.[4] The crystals of all compounds were mounted in an inert oil (perfluoropolyalkylether). Crystal structure determination were affected at 100 K. Crystallographic data (including structure factors) have been deposited with the Cambridge Crystallographic Data Centre as supplementary publication no. CCDC 2105835-2105842. Copies of the data can be obtained free of charge on application to Cambridge Crystallographic Data Centre, 12 Union Road, Cambridge CB2 1EZ, UK; [fax: (+44) 1223-336-033; email: **deposit@ccdc.cam.ac.uk**].

Table S1. Data collection and structure refinement details for compounds **3a, 6** and **4b**.

Parameter	**3a**	**6**	**4b**
CCDC No.	2105842	2105841	2105840
Formula	$C_{44}H_{49}NO_2P_2RuSSi$	$C_{41}H_{43}NO_2P_2RuSSi$	$C_{48}H_{45}F_{12}NO_2P_2RuSSi$
Formula weight [g·mol^{-1}]	847.00	804.92	1119.01
Temperature [K]	100(2)	100(2)	100(2)
Wave length [Å]	1.54184	1.54178	1.54178
Crystal system	Orthorhombic	Triclinic	Monoclinic
Space group	*P*bca	*P*-1	*P*2$_1$/n
a [Å]	22.1257(2)	11.0012(4)	14.1576(2)
b [Å]	15.97910(10)	11.6401(4)	15.6694(2)
c [Å]	23.4967(3)	15.0140(5)	22.6239(3)
α [°]	90	90.304(3)	90
β [°]	90	94.009(3)	94.9630(10)
γ [°]	90	g = 101.466(3)	90
Volume [Å^3]	8307.23(14)	1879.27(11)	5000.09(12)
Z	8	2	4
Calc. density [Mg·m^{-3}]	1.354	1.422	1.486
µ (Mo$_{K\alpha}$) [mm^{-1}]	4.812	5.289	4.504
F(000)	3520	832	2272
Crystal dimensions [mm]	0.110 x 0.090 x 0.010	0.100 x 0.100 x 0.030	0.245 x 0.180 x 0.109
Theta range [°]	3.762 to 67.076	3.875 to 74.991	3.435 to 67.051
Index ranges	-26 ≤ h ≤ 25	-13 ≤ h ≤ 13	-16 ≤ h ≤ 16
	-19 ≤ k ≤ 10	-14 ≤ k ≤ 14	-18 ≤ k ≤ 17
	-28 ≤ l ≤ 23	-18 ≤ l ≤ 18	-22 ≤ l ≤ 27
Reflections collected	32209	30478	32981
Independent reflections	7400 [*R*(int) = 0.0364]	7693 [*R*(int) = 0.0210]	8917 [*R*(int) = 0.0277]
Data/Restraints/Parameter	7400 / 0 / 483	7693 / 0 / 452	8917 / 3 / 651
Goodness-of-fit on F^2	1.034	1.044	1.049

Final *R* indices [I>2sigma(I)]	*R*1 = 0.0267, w*R*2 = 0.0617	*R*1 = 0.0296, w*R*2 = 0.0778	*R*1 = 0.0245, w*R*2 = 0.0622
Largest diff. peak and hole [e·Å^{-3}]	0.536 and -0.382	1.251 and -0.459	0.434 and -0.524

Table S2. Data collection and structure refinement details for compounds **8, 8-Na** and **9**.

Parameter	**8**	**8-Na**	**9**
CCDC No.	2105838	2105839	2105837
Formula	$C_{25}H_{21}N_2O_4PS$	$C_{66}H_{72}N_4Na_2O_{12}P_2S_2$	$C_{35}H_{34}ClN_2O_4PRuS$
Formula weight [g·mol^{-1}]	476.47	1285.31	746.19
Temperature [K]	100(2)	100(2)	100(2)
Wave length [Å]	0.71073	0.71073	0.71073
Crystal system	Monoclinic	Triclinic	Orthorhombic
Space group	$P2_1/n$	*P*-1	*P*bca
a [Å]	9.9256(8)	10.4725(4)	16.7758(7)
b [Å]	23.809(2)	12.8262(5)	11.9678(5)
c [Å]	10.2372(8)	26.3510(11)	32.1875(14)
α [°]	90	76.5772(14)	90
β [°]	115.465(3)	86.5732(14)	90
γ [°]	90	77.1140(14)	90
Volume [Å^3]	2184.2(3)	3356.0(2)	6462.3(5)
Z	4	2	8
Calc. density [Mg·m^{-3}]	1.449	1.272	1.534
μ ($Mo_{K\alpha}$) [mm^{-1}]	0.259	0.202	0.725
F(000)	992	1352	3056
Crystal dimensions [mm]	0.160 x 0.160 x 0.110	0.140 x 0.100 x 0.080	0.180 x 0.090 x 0.090
Theta range [°]	1.711 to 24.999	1.589 to 25.000	1.753 to 24.996
Index ranges	-11 ≤ h ≤ 11	-12 ≤ h ≤ 12	-19 ≤ h ≤ 19
	-28 ≤ k ≤ 27	-15 ≤ k ≤ 15	-14 ≤ k ≤ 14
	-12 ≤ l ≤ 8	-31 ≤ l ≤ 31	-38 ≤ l ≤ 38
Reflections collected	13809	53171	96091
Independent reflections	3852 [*R*(int) = 0.0425]	11830 [*R*(int) = 0.0387]	5692 [*R*(int) = 0.0424]
Data/Restraints/Parameter	3852 / 0 / 306	11830 / 12 / 858	5692 / 0 / 413
Goodness-of-fit on F^2	1.019	1.083	1.007
Final R indices [I>2sigma(I)]	*R*1 = 0.0380 w*R*2 = 0.0859	*R*1 = 0.0461 w*R*2 = 0.1056	*R*1 = 0.0215 w*R*2 = 0.0517
Largest diff. peak and hole hole [e·Å^{-3}]	0.352 and -0.372	0.449 and -0.433	0.376 and -0.300

Table S3. Data collection and structure refinement details for compounds **2a** and **11b**.

Parameter	**2b**	**11b**
CCDC No.	2105835	2105836
Formula	$C_{70}H_{66}N_4O_8P_2Ru_2S_2$	$C_{51}H_{40}F_{12}N_2O_4P_2RuS$
Formula weight [g·mol^{-1}]	1419.46	1167.92
Temperature [K]	100(2)	100(2)
Wave length [Å]	1.54184	1.54184
Crystal system	Monoclinic	Monoclinic
Space group	*P*n	*P*2$_1$/c
a [Å]	11.6283(2)	20.9628(3)
b [Å]	19.5630(3)	23.0578(3)
c [Å]	13.4339(2)	10.5054(2)
α [°]	90	90
β [°]	91.609(2)	102.9030(10)
γ [°]	90	90
Volume [Å^3]	3054.80(8)	4949.63(14)
Z	4	4
Calc. density [Mg·m^{-3}]	1.543	1.567
μ ($Mo_{K\alpha}$) [mm^{-1}]	5.641	4.395
F(000)	1456.0	2360
Crystal dimensions [mm]	0.239 × 0.101 × 0.027	0.176 x 0.078 x 0.044
Theta range [°]	7.986 to 134.136	3.834 to 67.070°.
Index ranges	-13 ≤ h ≤ 13	-24 ≤ h ≤ 25
	-17 ≤ k ≤ 23	-27 ≤ k ≤ 27
	-16 ≤ l ≤ 15	-12 ≤ l ≤ 12
Reflections collected	19537	33945
Independent reflections	9384 [*R*(int) = 0.0365]	8837 [*R*(int) = 0.0348]
Data/Restraints/Parameter	9384/130/868	8837 / 42 / 702
Goodness-of-fit on F^2	1.040	1.009
Final R indices [I>2sigma(I)]	*R*1 = 0.0393 w*R*2 = 0.1040	*R*1 = 0.0281 w*R*2 = 0.0662
Largest diff. peak and hole hole [e·Å^{-3}]	1.49 and -0.77	0.548 and -0.492

3.2 Crystal Structure Determination of Compound 3b

Additional information concerning the structure refinement: All hydrogen atoms were placed on ideal positions except for H45 on P2 and H1 at C1 which were found in the difference Fourier map and refined independently.

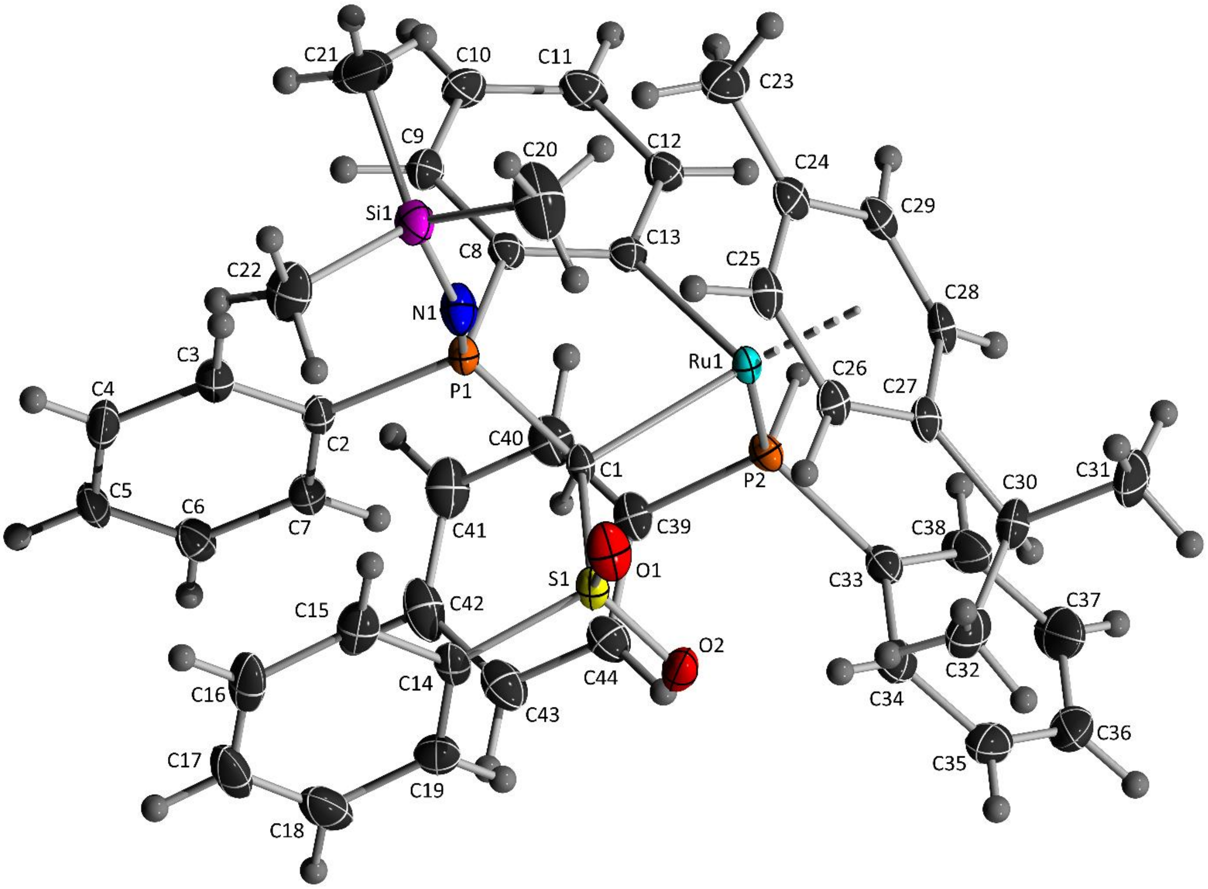

Figure S33. ORTEP Plot of **3a**. Ellipsoids are drawn at the 50% probability level.

Table S4. Atomic coordinates (x 10^4) and equivalent isotropic displacement parameters ($Å^2$x 10^3) for **3a**. U(eq) is defined as one third of the trace of the orthogonalized U_{ij} tensor.

	x	y	z	U(eq)
Ru(1)	7368(1)	6231(1)	5839(1)	14(1)
O(1)	7069(1)	8565(1)	5958(1)	29(1)
S(1)	6763(1)	7995(1)	6337(1)	19(1)
P(1)	6143(1)	7070(1)	5362(1)	15(1)
N(1)	6363(1)	7688(1)	4906(1)	24(1)
Si(1)	6388(1)	8146(1)	4271(1)	23(1)
C(1)	6593(1)	7038(1)	6007(1)	14(1)
P(2)	7103(1)	5407(1)	6593(1)	16(1)
O(2)	7052(1)	7809(1)	6874(1)	27(1)
C(2)	5349(1)	7244(1)	5547(1)	17(1)
C(4)	4379(1)	7835(1)	5292(1)	27(1)

C(5)	4137(1)	7569(1)	5804(1)	25(1)
C(6)	4496(1)	7134(1)	6185(1)	24(1)
C(7)	5097(1)	6969(1)	6057(1)	19(1)
C(8)	6189(1)	5965(1)	5211(1)	18(1)
C(9)	5756(1)	5549(1)	4883(1)	23(1)
C(3)	4982(1)	7674(1)	5166(1)	24(1)
C(14)	6058(1)	8458(1)	6512(1)	22(1)
C(11)	6325(1)	4284(1)	4975(1)	25(1)
C(12)	6753(1)	4692(1)	5304(1)	20(1)
C(13)	6699(1)	5550(1)	5430(1)	17(1)
C(10)	5826(1)	4707(1)	4761(1)	27(1)
C(15)	5757(1)	8937(1)	6108(1)	27(1)
C(16)	5188(1)	9248(1)	6237(1)	37(1)
C(17)	4930(1)	9082(2)	6759(1)	42(1)
C(18)	5233(1)	8610(2)	7160(1)	39(1)
C(19)	5802(1)	8293(1)	7039(1)	28(1)
C(20)	7175(1)	8492(2)	4102(1)	48(1)
C(21)	6118(2)	7426(2)	3695(1)	51(1)
C(22)	5923(1)	9127(1)	4238(1)	33(1)
C(23)	7587(1)	6010(2)	4407(1)	31(1)
C(24)	7835(1)	6231(1)	4984(1)	22(1)
C(25)	7811(1)	7057(1)	5189(1)	22(1)
C(26)	8074(1)	7270(1)	5725(1)	19(1)
C(27)	8361(1)	6672(1)	6063(1)	20(1)
C(28)	8322(1)	5817(1)	5881(1)	21(1)
C(29)	8084(1)	5603(1)	5345(1)	22(1)
C(30)	8735(1)	6890(1)	6584(1)	24(1)
C(31)	9405(1)	6838(2)	6417(1)	38(1)
C(32)	8597(1)	7744(2)	6842(1)	32(1)
C(33)	7569(1)	5429(1)	7234(1)	21(1)
C(34)	7719(1)	6186(1)	7492(1)	24(1)
C(35)	8097(1)	6203(2)	7963(1)	30(1)
C(36)	8332(1)	5468(2)	8180(1)	38(1)
C(37)	8185(1)	4719(2)	7926(1)	42(1)
C(38)	7804(1)	4693(2)	7456(1)	32(1)
C(39)	6322(1)	5434(1)	6856(1)	20(1)
C(40)	5870(1)	4993(1)	6568(1)	24(1)
C(41)	5273(1)	5037(1)	6752(1)	30(1)
C(42)	5120(1)	5522(2)	7218(1)	32(1)
C(43)	5564(1)	5964(2)	7503(1)	30(1)
C(44)	6164(1)	5920(1)	7326(1)	25(1)

Table S5. Anisotropic displacement parameters (Å^{2}x 10^3) for **3a**. The anisotropic displacement factor exponent takes the form: $-2p^2[h^2a^{*2}U^{11} + ... + 2 h k a^* b^* U^{12}]$.

	U^{11}	U^{22}	U^{33}	U^{23}	U^{13}	U^{12}
Ru(1)	11(1)	12(1)	18(1)	1(1)	0(1)	1(1)
O(1)	21(1)	15(1)	49(1)	3(1)	1(1)	-5(1)
S(1)	15(1)	11(1)	31(1)	-3(1)	-4(1)	0(1)
P(1)	12(1)	15(1)	20(1)	1(1)	-1(1)	0(1)
N(1)	17(1)	27(1)	29(1)	7(1)	2(1)	0(1)
Si(1)	24(1)	24(1)	22(1)	5(1)	-1(1)	5(1)
C(1)	11(1)	11(1)	21(1)	2(1)	0(1)	1(1)
P(2)	17(1)	12(1)	20(1)	1(1)	1(1)	2(1)
O(2)	25(1)	21(1)	35(1)	-9(1)	-13(1)	6(1)
C(2)	14(1)	13(1)	25(1)	-3(1)	-3(1)	0(1)
C(4)	18(1)	30(1)	34(1)	-2(1)	-8(1)	4(1)
C(5)	12(1)	25(1)	38(2)	-10(1)	1(1)	2(1)
C(6)	18(1)	22(1)	31(1)	-5(1)	4(1)	-3(1)
C(7)	16(1)	14(1)	27(1)	-3(1)	-1(1)	-1(1)
C(8)	15(1)	19(1)	19(1)	-1(1)	2(1)	-1(1)
C(9)	17(1)	26(1)	25(1)	-4(1)	-1(1)	-1(1)
C(3)	19(1)	27(1)	26(1)	0(1)	-2(1)	2(1)
C(14)	18(1)	11(1)	36(1)	-8(1)	-4(1)	1(1)
C(11)	25(1)	18(1)	33(1)	-7(1)	4(1)	-3(1)
C(12)	20(1)	18(1)	24(1)	-2(1)	1(1)	0(1)
C(13)	17(1)	18(1)	16(1)	0(1)	2(1)	-1(1)
C(10)	22(1)	29(1)	30(1)	-11(1)	-1(1)	-6(1)
C(15)	26(1)	12(1)	43(2)	-2(1)	-9(1)	-1(1)
C(16)	28(1)	19(1)	64(2)	-12(1)	-18(1)	9(1)
C(17)	23(1)	34(1)	70(2)	-28(1)	-5(1)	9(1)
C(18)	33(1)	37(1)	46(2)	-21(1)	6(1)	1(1)
C(19)	28(1)	23(1)	33(1)	-12(1)	-4(1)	2(1)
C(20)	35(2)	55(2)	54(2)	33(2)	13(1)	6(1)
C(21)	72(2)	49(2)	33(2)	-15(1)	-21(2)	25(2)
C(22)	33(1)	25(1)	40(2)	7(1)	-7(1)	2(1)
C(23)	28(1)	42(1)	24(1)	-2(1)	5(1)	-4(1)
C(24)	12(1)	31(1)	23(1)	1(1)	6(1)	-1(1)
C(25)	12(1)	24(1)	28(1)	9(1)	4(1)	-2(1)
C(26)	11(1)	20(1)	27(1)	2(1)	2(1)	-4(1)
C(27)	10(1)	24(1)	25(1)	2(1)	2(1)	-1(1)
C(28)	10(1)	21(1)	32(1)	4(1)	1(1)	3(1)
C(29)	14(1)	21(1)	30(1)	-2(1)	6(1)	2(1)
C(30)	16(1)	29(1)	29(1)	4(1)	-5(1)	0(1)

C(31)	15(1)	59(2)	40(2)	-1(1)	-6(1)	-1(1)
C(32)	27(1)	31(1)	38(2)	-4(1)	-10(1)	-6(1)
C(33)	19(1)	23(1)	19(1)	2(1)	3(1)	5(1)
C(34)	22(1)	26(1)	23(1)	1(1)	2(1)	0(1)
C(35)	26(1)	43(1)	22(1)	-5(1)	3(1)	-2(1)
C(36)	33(1)	62(2)	21(1)	-2(1)	-4(1)	13(1)
C(37)	52(2)	45(2)	30(2)	5(1)	-7(1)	25(1)
C(38)	43(2)	27(1)	27(1)	-1(1)	-1(1)	14(1)
C(39)	20(1)	14(1)	26(1)	7(1)	2(1)	1(1)
C(40)	25(1)	18(1)	30(1)	6(1)	3(1)	-1(1)
C(41)	22(1)	27(1)	40(2)	12(1)	1(1)	-5(1)
C(42)	22(1)	34(1)	40(2)	16(1)	8(1)	4(1)
C(43)	27(1)	33(1)	30(1)	4(1)	9(1)	4(1)
C(44)	25(1)	24(1)	27(1)	2(1)	5(1)	2(1)

3.3 Crystal Structure Determination of Compound 6

Additional information concerning the structure refinement: All hydrogen atoms were placed on ideal positions except for H2 on P2 and H1 on C1 which were found in the difference Fourier map and refined independently.

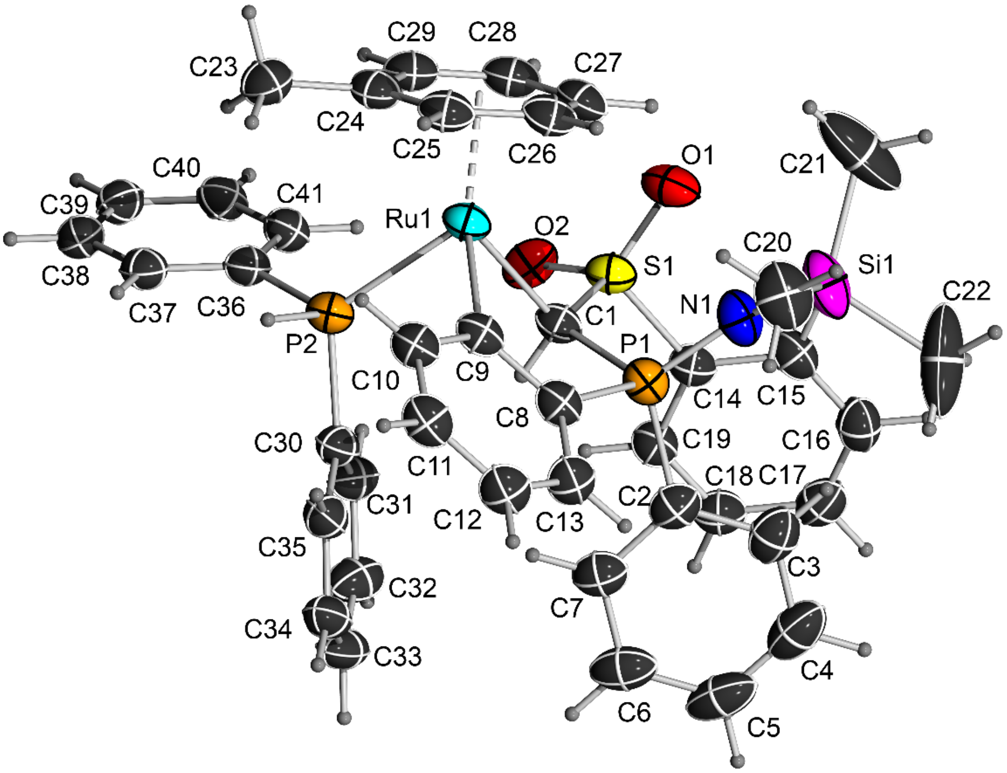

Figure S34. ORTEP Plot of **6**. Ellipsoids are drawn at the 50% probability level.

Table S6. Atomic coordinates (x 10^4) and equivalent isotropic displacement parameters ($Å^2$x 10^3) for **6**. U(eq) is defined as one third of the trace of the orthogonalized U_{ij} tensor.

	x	y	z	U(eq)
Ru(1)	10343(1)	8970(1)	7634(1)	33(1)
S(1)	8139(1)	7566(1)	8891(1)	36(1)
P(1)	8035(1)	6947(1)	6914(1)	35(1)
P(2)	11880(1)	7982(1)	8039(1)	34(1)
C(2)	7502(2)	5361(2)	6939(1)	40(1)
Si(1)	6081(1)	8142(1)	5940(1)	57(1)
O(1)	7492(2)	8533(1)	8835(1)	48(1)
O(2)	8999(2)	7548(1)	9666(1)	44(1)
N(1)	6961(2)	7607(2)	6715(1)	44(1)
C(1)	8973(2)	7431(2)	7942(1)	33(1)
C(8)	9290(2)	7138(2)	6184(1)	36(1)
C(9)	10318(2)	8031(2)	6449(1)	35(1)
C(3)	6243(2)	4877(2)	6776(2)	52(1)
C(10)	11271(2)	8256(2)	5857(1)	39(1)
C(4)	5836(3)	3664(3)	6795(2)	64(1)
C(11)	11209(2)	7622(2)	5063(1)	43(1)
C(5)	6670(3)	2940(2)	6975(2)	63(1)
C(12)	10198(2)	6724(2)	4831(2)	45(1)
C(6)	7916(3)	3413(2)	7135(2)	53(1)
C(13)	9232(2)	6487(2)	5393(2)	42(1)
C(7)	8334(2)	4618(2)	7116(2)	44(1)
C(14)	6972(2)	6280(2)	8919(1)	35(1)
C(15)	5760(2)	6315(2)	8613(2)	42(1)
C(16)	4844(2)	5320(2)	8669(2)	46(1)
C(17)	5142(2)	4307(2)	9023(2)	46(1)
C(18)	6360(2)	4279(2)	9315(2)	44(1)
C(19)	7281(2)	5262(2)	9258(1)	38(1)
C(20)	6866(3)	8498(3)	4888(2)	61(1)
C(21)	5708(5)	9533(5)	6389(3)	112(2)
C(22)	4578(3)	7099(5)	5642(3)	110(2)
C(23)	13037(2)	11022(2)	7772(2)	48(1)
C(24)	11637(2)	10726(2)	7746(2)	41(1)
C(25)	10907(2)	10632(2)	6930(2)	41(1)
C(26)	9592(2)	10384(2)	6906(2)	42(1)
C(27)	9000(2)	10190(2)	7703(2)	41(1)
C(28)	9734(2)	10295(2)	8537(2)	41(1)
C(29)	11020(2)	10564(2)	8554(2)	41(1)
C(30)	11642(2)	6380(2)	7960(1)	36(1)
C(31)	11232(2)	5678(2)	8671(2)	41(1)
C(32)	11090(2)	4465(2)	8598(2)	49(1)

C(33)	11361(2)	3951(2)	7825(2)	51(1)
C(34)	11765(2)	4638(2)	7115(2)	47(1)
C(35)	11895(2)	5847(2)	7174(2)	41(1)
C(36)	12734(2)	8300(2)	9125(1)	36(1)
C(37)	14029(2)	8442(2)	9198(2)	38(1)
C(38)	14680(2)	8635(2)	10032(2)	42(1)
C(39)	14036(2)	8688(2)	10787(2)	43(1)
C(40)	12750(2)	8548(2)	10719(2)	42(1)
C(41)	12095(2)	8360(2)	9890(2)	39(1)

Table S7. Anisotropic displacement parameters ($Å^2$x 10^3) for **6**. The anisotropic displacement factor exponent takes the form: $-2p^2[h^2a^{*2}U^{11} + ... + 2 h k a^* b^* U^{12}]$.

	U^{11}	U^{22}	U^{33}	U^{23}	U^{13}	U^{12}
Ru(1)	41(1)	29(1)	31(1)	3(1)	6(1)	11(1)
S(1)	44(1)	32(1)	32(1)	1(1)	8(1)	9(1)
P(1)	38(1)	40(1)	29(1)	2(1)	3(1)	11(1)
P(2)	39(1)	30(1)	33(1)	1(1)	5(1)	9(1)
C(2)	46(1)	44(1)	29(1)	-4(1)	5(1)	5(1)
Si(1)	49(1)	86(1)	43(1)	17(1)	6(1)	32(1)
O(1)	59(1)	36(1)	52(1)	4(1)	22(1)	17(1)
O(2)	52(1)	45(1)	30(1)	-1(1)	5(1)	0(1)
N(1)	42(1)	57(1)	37(1)	6(1)	4(1)	18(1)
C(1)	40(1)	32(1)	31(1)	3(1)	6(1)	12(1)
C(8)	41(1)	40(1)	30(1)	4(1)	4(1)	14(1)
C(9)	44(1)	36(1)	29(1)	5(1)	4(1)	15(1)
C(3)	49(1)	62(2)	42(1)	-9(1)	4(1)	2(1)
C(10)	43(1)	39(1)	36(1)	3(1)	6(1)	12(1)
C(4)	60(2)	67(2)	56(2)	-19(1)	12(1)	-13(1)
C(11)	47(1)	53(1)	31(1)	5(1)	9(1)	17(1)
C(5)	83(2)	45(1)	54(2)	-15(1)	24(1)	-10(1)
C(12)	50(1)	56(1)	31(1)	-5(1)	2(1)	15(1)
C(6)	75(2)	40(1)	43(1)	-6(1)	20(1)	6(1)
C(13)	44(1)	50(1)	34(1)	-2(1)	1(1)	11(1)
C(7)	53(1)	41(1)	36(1)	-2(1)	9(1)	6(1)
C(14)	40(1)	36(1)	29(1)	0(1)	6(1)	9(1)
C(15)	43(1)	49(1)	38(1)	3(1)	6(1)	18(1)
C(16)	37(1)	60(1)	42(1)	-5(1)	4(1)	12(1)
C(17)	44(1)	47(1)	45(1)	-6(1)	9(1)	0(1)
C(18)	51(1)	38(1)	43(1)	2(1)	6(1)	8(1)
C(19)	40(1)	39(1)	36(1)	2(1)	3(1)	9(1)
C(20)	64(2)	82(2)	41(1)	20(1)	9(1)	21(1)

C(21)	147(4)	150(4)	75(2)	13(2)	8(2)	118(4)
C(22)	37(1)	215(6)	73(2)	50(3)	-4(1)	10(2)
C(23)	50(1)	38(1)	55(1)	2(1)	5(1)	4(1)
C(24)	49(1)	28(1)	48(1)	3(1)	6(1)	10(1)
C(25)	55(1)	31(1)	40(1)	7(1)	9(1)	11(1)
C(26)	55(1)	34(1)	41(1)	7(1)	3(1)	17(1)
C(27)	49(1)	33(1)	47(1)	5(1)	9(1)	19(1)
C(28)	57(1)	31(1)	39(1)	2(1)	10(1)	15(1)
C(29)	53(1)	30(1)	40(1)	0(1)	4(1)	11(1)
C(30)	39(1)	31(1)	38(1)	-2(1)	-2(1)	11(1)
C(31)	52(1)	35(1)	36(1)	0(1)	-5(1)	10(1)
C(32)	63(1)	35(1)	45(1)	6(1)	-12(1)	7(1)
C(33)	60(1)	32(1)	60(2)	-5(1)	-17(1)	15(1)
C(34)	49(1)	44(1)	50(1)	-13(1)	-8(1)	19(1)
C(35)	41(1)	41(1)	42(1)	-2(1)	0(1)	13(1)
C(36)	41(1)	30(1)	37(1)	-1(1)	2(1)	9(1)
C(37)	42(1)	31(1)	43(1)	0(1)	6(1)	6(1)
C(38)	39(1)	34(1)	50(1)	0(1)	0(1)	4(1)
C(39)	46(1)	37(1)	43(1)	-5(1)	-4(1)	7(1)
C(40)	48(1)	42(1)	38(1)	-4(1)	3(1)	15(1)
C(41)	42(1)	38(1)	39(1)	-1(1)	3(1)	13(1)

3.4 Crystal Structure Determination of Compound 4b

Additional information concerning the structure refinement: All hydrogen atoms were placed on ideal positions except for H1 on C2 which was found in the difference Fourier map and refined independently. A disordered CF_3 moiety (occupancy 0.55 : 0.45) was solved using the PART and SAME instructions.

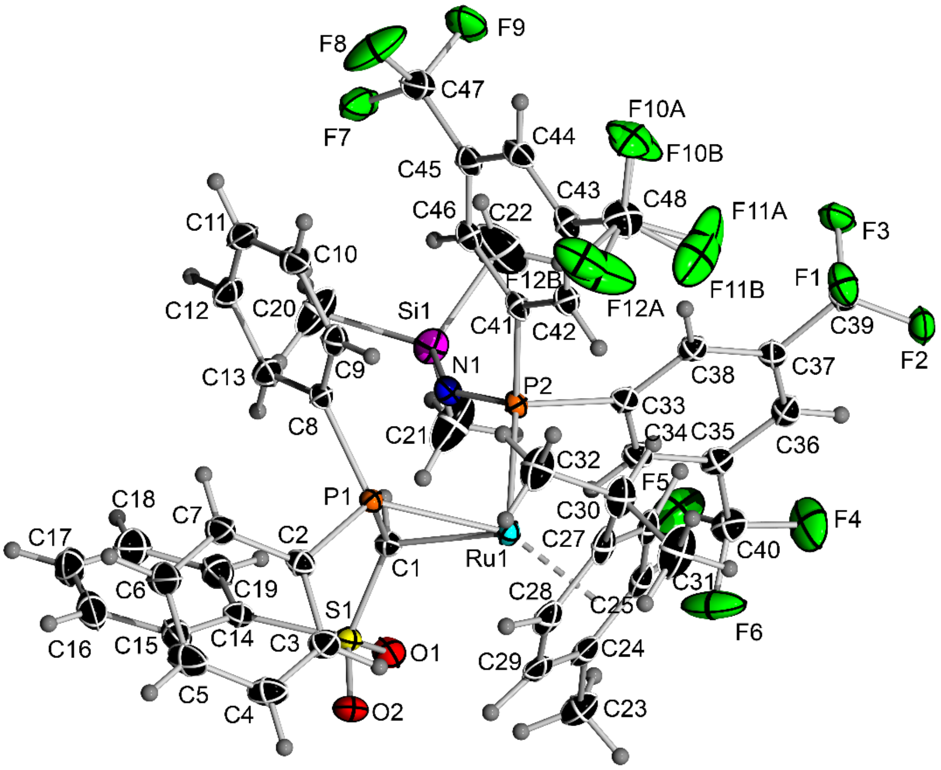

Figure S35. ORTEP Plot of **4b**. Ellipsoids are drawn at the 50% probability level.

Table S8. Atomic coordinates (x 10^4) and equivalent isotropic displacement parameters ($Å^2$x 10^3) for **4b**. U(eq) is defined as one third of the trace of the orthogonalized U_{ij} tensor.

	x	y	z	U(eq)
C(2)	6726(1)	1618(1)	4503(1)	19(1)
F(2)	5183(1)	6507(1)	1056(1)	29(1)
P(2)	5035(1)	3165(1)	2734(1)	14(1)
O(2)	7744(1)	792(1)	3330(1)	26(1)
Ru(1)	6610(1)	2962(1)	3178(1)	15(1)
N(1)	4431(1)	2356(1)	2543(1)	18(1)
F(1)	4950(1)	6490(1)	1981(1)	31(1)
Si(1)	3569(1)	1979(1)	2053(1)	24(1)
P(1)	6041(1)	2129(1)	3881(1)	14(1)
O(1)	6743(1)	709(1)	2379(1)	25(1)
S(1)	6808(1)	782(1)	3017(1)	18(1)
C(1)	6126(1)	1653(1)	3178(1)	16(1)
C(48)	4503(2)	5892(1)	4066(1)	33(1)
C(47)	1796(1)	3877(1)	3631(1)	28(1)

C(46)	3382(1)	3618(1)	3249(1)	19(1)
C(45)	2809(1)	4119(1)	3576(1)	21(1)
C(44)	3161(1)	4867(1)	3845(1)	23(1)
C(43)	4090(1)	5095(1)	3786(1)	22(1)
C(42)	4673(1)	4585(1)	3467(1)	20(1)
C(41)	4315(1)	3849(1)	3186(1)	17(1)
C(40)	5973(1)	3432(1)	561(1)	26(1)
C(39)	4748(1)	6083(1)	1469(1)	22(1)
C(38)	4888(1)	4711(1)	2023(1)	18(1)
C(37)	5014(1)	5157(1)	1503(1)	19(1)
C(36)	5361(1)	4758(1)	1021(1)	21(1)
C(35)	5566(1)	3891(1)	1063(1)	20(1)
C(34)	5435(1)	3440(1)	1577(1)	19(1)
C(9)	4578(1)	2741(1)	4491(1)	20(1)
F(9)	1197(1)	4364(1)	3298(1)	54(1)
C(3)	7715(1)	1615(1)	4544(1)	23(1)
F(3)	3814(1)	6182(1)	1326(1)	37(1)
C(5)	7754(2)	758(1)	5427(1)	33(1)
F(5)	5438(1)	2786(1)	356(1)	51(1)
C(4)	8222(1)	1186(1)	5004(1)	28(1)
F(4)	6067(1)	3934(1)	95(1)	52(1)
C(6)	6775(2)	763(2)	5393(1)	35(1)
F(6)	6823(1)	3108(1)	722(1)	54(1)
C(7)	6261(1)	1192(1)	4936(1)	26(1)
F(7)	1601(1)	3078(1)	3472(1)	46(1)
C(8)	4843(1)	2119(1)	4099(1)	16(1)
F(8)	1560(1)	3955(1)	4186(1)	65(1)
C(10)	3674(1)	2737(1)	4683(1)	25(1)
C(11)	3031(1)	2114(1)	4479(1)	27(1)
C(12)	3295(1)	1492(1)	4091(1)	27(1)
C(13)	4200(1)	1489(1)	3898(1)	21(1)
C(14)	6174(1)	-93(1)	3290(1)	21(1)
C(15)	6432(2)	-410(1)	3853(1)	28(1)
C(16)	5892(2)	-1063(1)	4070(1)	35(1)
C(17)	5116(2)	-1385(1)	3731(1)	39(1)
C(18)	4870(2)	-1065(1)	3169(1)	37(1)
C(19)	5400(1)	-415(1)	2942(1)	29(1)
C(20)	2879(2)	1156(2)	2432(1)	40(1)
C(21)	4069(2)	1470(2)	1401(1)	50(1)
C(22)	2714(2)	2819(2)	1754(1)	63(1)
C(23)	8122(2)	2344(1)	2125(1)	33(1)
C(24)	7857(1)	2944(1)	2600(1)	24(1)

C(25)	7314(1)	3680(1)	2461(1)	22(1)
C(26)	7086(1)	4264(1)	2908(1)	22(1)
C(27)	7433(1)	4155(1)	3507(1)	23(1)
C(28)	7989(1)	3411(1)	3640(1)	24(1)
C(29)	8188(1)	2819(1)	3206(1)	24(1)
C(30)	7305(1)	4832(1)	3968(1)	28(1)
C(31)	8240(2)	5328(2)	4093(1)	43(1)
C(32)	6970(2)	4476(1)	4542(1)	35(1)
C(33)	5110(1)	3848(1)	2070(1)	17(1)
F(10A)	3846(8)	6420(7)	4282(5)	31(3)
F(11A)	4870(20)	6372(12)	3639(14)	69(4)
F(12A)	5171(18)	5756(14)	4471(16)	69(4)
F(10B)	3875(13)	6391(11)	4247(8)	56(5)
F(11B)	5079(18)	6313(13)	3762(10)	58(3)
F(12B)	5055(15)	5691(13)	4595(8)	46(3)

Table S9. Anisotropic displacement parameters (Å^{2}x 10^3) for **4b**. The anisotropic displacement factor exponent takes the form: $-2p^2[h^2a^{*2}U^{11} + ... + 2 h k a^* b^* U^{12}]$.

	U^{11}	U^{22}	U^{33}	U^{23}	U^{13}	U^{12}
C(2)	19(1)	19(1)	16(1)	0(1)	0(1)	2(1)
F(2)	32(1)	26(1)	28(1)	11(1)	2(1)	-3(1)
P(2)	16(1)	13(1)	14(1)	0(1)	0(1)	0(1)
O(2)	18(1)	28(1)	31(1)	2(1)	3(1)	6(1)
Ru(1)	13(1)	17(1)	14(1)	2(1)	0(1)	-2(1)
N(1)	19(1)	16(1)	19(1)	0(1)	-2(1)	1(1)
F(1)	46(1)	20(1)	26(1)	1(1)	1(1)	0(1)
Si(1)	23(1)	27(1)	21(1)	-4(1)	-4(1)	-6(1)
P(1)	13(1)	16(1)	15(1)	2(1)	1(1)	0(1)
O(1)	32(1)	24(1)	20(1)	-1(1)	7(1)	6(1)
S(1)	18(1)	19(1)	20(1)	1(1)	4(1)	5(1)
C(1)	14(1)	16(1)	17(1)	1(1)	1(1)	1(1)
C(48)	38(1)	28(1)	34(1)	-11(1)	4(1)	1(1)
C(47)	25(1)	29(1)	31(1)	1(1)	4(1)	7(1)
C(46)	22(1)	18(1)	17(1)	3(1)	0(1)	1(1)
C(45)	23(1)	22(1)	18(1)	6(1)	1(1)	5(1)
C(44)	29(1)	23(1)	17(1)	2(1)	2(1)	10(1)
C(43)	31(1)	18(1)	16(1)	0(1)	-1(1)	3(1)
C(42)	23(1)	20(1)	17(1)	1(1)	0(1)	0(1)
C(41)	22(1)	15(1)	14(1)	3(1)	0(1)	2(1)
C(40)	25(1)	36(1)	18(1)	0(1)	2(1)	2(1)
C(39)	19(1)	24(1)	22(1)	6(1)	-3(1)	-1(1)

C(38)	15(1)	21(1)	17(1)	0(1)	-1(1)	-1(1)
C(37)	14(1)	21(1)	20(1)	3(1)	-4(1)	-2(1)
C(36)	18(1)	28(1)	15(1)	5(1)	-3(1)	-3(1)
C(35)	15(1)	28(1)	15(1)	-2(1)	-2(1)	-2(1)
C(34)	17(1)	21(1)	20(1)	-1(1)	-1(1)	0(1)
C(9)	22(1)	21(1)	17(1)	2(1)	2(1)	0(1)
F(9)	24(1)	45(1)	89(1)	24(1)	-10(1)	5(1)
C(3)	20(1)	30(1)	20(1)	3(1)	0(1)	0(1)
F(3)	19(1)	29(1)	60(1)	10(1)	-4(1)	4(1)
C(5)	33(1)	45(1)	22(1)	8(1)	-2(1)	14(1)
F(5)	55(1)	54(1)	49(1)	-30(1)	20(1)	-14(1)
C(4)	20(1)	40(1)	24(1)	0(1)	-3(1)	6(1)
F(4)	90(1)	48(1)	23(1)	6(1)	25(1)	11(1)
C(6)	32(1)	46(1)	27(1)	16(1)	9(1)	12(1)
F(6)	35(1)	101(1)	28(1)	-9(1)	4(1)	31(1)
C(7)	22(1)	33(1)	24(1)	9(1)	5(1)	6(1)
F(7)	28(1)	29(1)	84(1)	-4(1)	18(1)	-4(1)
C(8)	15(1)	19(1)	16(1)	5(1)	1(1)	1(1)
F(8)	39(1)	115(1)	44(1)	-16(1)	24(1)	-15(1)
C(10)	27(1)	30(1)	20(1)	5(1)	7(1)	9(1)
C(11)	17(1)	40(1)	26(1)	11(1)	5(1)	3(1)
C(12)	17(1)	32(1)	32(1)	6(1)	1(1)	-6(1)
C(13)	20(1)	21(1)	23(1)	1(1)	1(1)	-1(1)
C(14)	23(1)	18(1)	24(1)	0(1)	7(1)	6(1)
C(15)	34(1)	22(1)	28(1)	3(1)	5(1)	6(1)
C(16)	50(1)	24(1)	34(1)	8(1)	14(1)	7(1)
C(17)	45(1)	21(1)	53(1)	2(1)	23(1)	-1(1)
C(18)	32(1)	27(1)	53(1)	-4(1)	6(1)	-4(1)
C(19)	29(1)	24(1)	33(1)	0(1)	2(1)	2(1)
C(20)	39(1)	52(1)	29(1)	-13(1)	6(1)	-26(1)
C(21)	60(2)	59(2)	31(1)	-22(1)	15(1)	-37(1)
C(22)	54(2)	62(2)	65(2)	3(1)	-40(1)	8(1)
C(23)	28(1)	42(1)	30(1)	0(1)	11(1)	-2(1)
C(24)	15(1)	34(1)	24(1)	3(1)	7(1)	-6(1)
C(25)	18(1)	29(1)	20(1)	5(1)	2(1)	-10(1)
C(26)	21(1)	22(1)	23(1)	6(1)	1(1)	-11(1)
C(27)	21(1)	23(1)	22(1)	3(1)	-2(1)	-13(1)
C(28)	15(1)	33(1)	22(1)	6(1)	-4(1)	-9(1)
C(29)	10(1)	33(1)	29(1)	7(1)	3(1)	-3(1)
C(30)	32(1)	28(1)	24(1)	0(1)	-2(1)	-10(1)
C(31)	50(1)	47(1)	30(1)	-6(1)	-2(1)	-26(1)
C(32)	43(1)	36(1)	26(1)	-3(1)	6(1)	-8(1)

C(33)	14(1)	20(1)	16(1)	1(1)	-2(1)	-1(1)
F(10A)	42(4)	22(4)	32(4)	-10(3)	8(3)	10(3)
F(11A)	113(9)	38(3)	64(7)	-25(4)	43(7)	-44(5)
F(12A)	61(5)	56(5)	83(8)	-49(5)	-40(6)	18(4)
F(10B)	57(8)	32(6)	77(9)	-30(5)	-5(5)	9(5)
F(11B)	78(6)	39(3)	61(6)	-30(4)	32(5)	-27(4)
F(12B)	55(4)	40(3)	40(5)	-20(4)	-18(3)	12(2)

3.5 Crystal Structure Determination of Compound 8

Additional information concerning the structure refinement: All hydrogen atoms were placed on ideal positions except for H1 and H1A on C1 which were found in the difference Fourier map and refined independently.

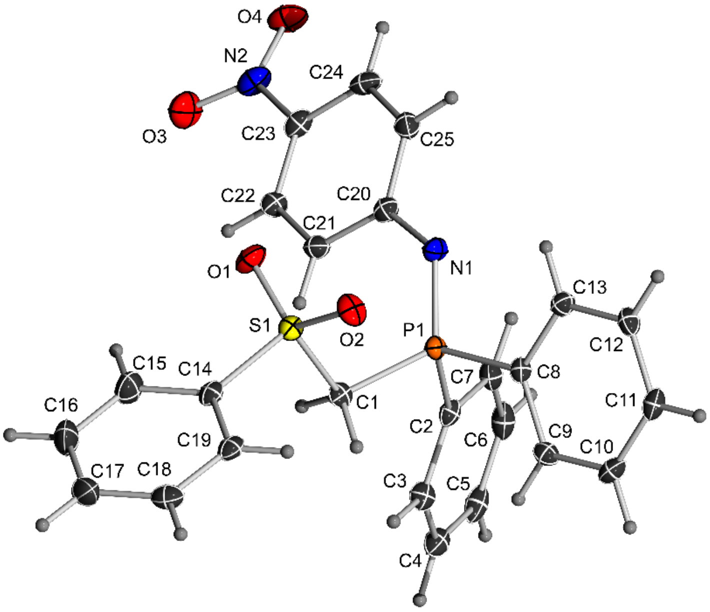

Figure S36. ORTEP Plot of **8**. Ellipsoids are drawn at the 50% probability level.

Table S10. Atomic coordinates (x 10^4) and equivalent isotropic displacement parameters (Å^{2}x 10^3) for **8**. U(eq) is defined as one third of the trace of the orthogonalized U_{ij} tensor.

	x	y	z	U(eq)
S(1)	4627(1)	2387(1)	9639(1)	14(1)
P(1)	5309(1)	3385(1)	8177(1)	13(1)
N(1)	5033(2)	3799(1)	9231(2)	16(1)
O(1)	3544(2)	2671(1)	9980(2)	22(1)
C(1)	4561(3)	2665(1)	8002(3)	15(1)
O(2)	6162(2)	2382(1)	10691(2)	20(1)
N(2)	-380(2)	4851(1)	8260(2)	23(1)
C(3)	4176(2)	3261(1)	5142(2)	17(1)
O(3)	-1571(2)	4608(1)	7529(2)	31(1)
C(4)	3625(2)	3474(1)	3745(3)	20(1)
O(4)	-316(2)	5330(1)	8739(2)	28(1)

C(5)	3404(2)	4048(1)	3512(3)	22(1)
C(6)	3770(3)	4408(1)	4676(3)	22(1)
C(7)	4360(2)	4199(1)	6076(3)	19(1)
C(8)	7291(2)	3300(1)	8815(2)	13(1)
C(9)	7870(2)	3039(1)	7944(2)	15(1)
C(10)	9394(3)	2981(1)	8437(3)	18(1)
C(11)	10352(3)	3181(1)	9793(3)	17(1)
C(12)	9779(2)	3441(1)	10652(3)	17(1)
C(13)	8253(2)	3503(1)	10172(2)	16(1)
C(14)	4047(2)	1683(1)	9193(2)	15(1)
C(15)	2561(3)	1546(1)	8798(3)	26(1)
C(16)	2125(3)	990(1)	8484(3)	35(1)
C(17)	3148(3)	582(1)	8568(3)	28(1)
C(18)	4625(3)	725(1)	8957(3)	21(1)
C(19)	5077(3)	1276(1)	9265(2)	17(1)
C(20)	3665(3)	4026(1)	8995(2)	16(1)
C(21)	2277(2)	3833(1)	7945(2)	17(1)
C(22)	976(2)	4101(1)	7729(2)	16(1)
C(23)	1008(3)	4563(1)	8547(3)	17(1)
C(24)	2351(3)	4767(1)	9614(3)	21(1)
C(25)	3645(3)	4491(1)	9844(3)	18(1)
C(2)	4557(2)	3621(1)	6315(2)	15(1)

Table S11. Anisotropic displacement parameters (Å^{2}x 10^3) for **8**. The anisotropic displacement factor exponent takes the form: $-2p^2[\, h^2a^{*2}U^{11} + ... + 2\, h\, k\, a^*\, b^*\, U^{12}\,]$.

	U^{11}	U^{22}	U^{33}	U^{23}	U^{13}	U^{12}
S(1)	13(1)	17(1)	13(1)	2(1)	5(1)	1(1)
P(1)	11(1)	15(1)	12(1)	0(1)	4(1)	1(1)
N(1)	12(1)	20(1)	16(1)	-2(1)	5(1)	2(1)
O(1)	24(1)	23(1)	28(1)	2(1)	18(1)	6(1)
C(1)	10(1)	19(1)	12(1)	1(1)	1(1)	1(1)
O(2)	16(1)	22(1)	16(1)	3(1)	0(1)	1(1)
N(2)	24(1)	23(1)	23(1)	6(1)	14(1)	7(1)
C(3)	13(1)	19(1)	16(1)	3(1)	4(1)	2(1)
O(3)	19(1)	38(1)	33(1)	0(1)	8(1)	4(1)
C(4)	14(1)	30(2)	14(1)	1(1)	5(1)	-1(1)
O(4)	38(1)	21(1)	31(1)	2(1)	20(1)	11(1)
C(5)	12(1)	33(2)	20(1)	11(1)	5(1)	1(1)
C(6)	15(1)	19(1)	31(2)	7(1)	9(1)	0(1)
C(7)	14(1)	18(1)	23(1)	0(1)	7(1)	-2(1)
C(8)	11(1)	14(1)	12(1)	3(1)	4(1)	0(1)

C(9)	17(1)	15(1)	13(1)	0(1)	5(1)	0(1)
C(10)	20(1)	20(1)	20(1)	2(1)	13(1)	4(1)
C(11)	12(1)	18(1)	20(1)	7(1)	7(1)	2(1)
C(12)	15(1)	18(1)	14(1)	1(1)	3(1)	-4(1)
C(13)	16(1)	16(1)	17(1)	0(1)	9(1)	1(1)
C(14)	16(1)	18(1)	12(1)	3(1)	7(1)	1(1)
C(15)	19(1)	27(2)	36(2)	-2(1)	15(1)	1(1)
C(16)	26(2)	33(2)	53(2)	-13(2)	24(1)	-12(1)
C(17)	35(2)	21(1)	33(2)	-7(1)	22(1)	-8(1)
C(18)	28(1)	21(1)	18(1)	0(1)	11(1)	3(1)
C(19)	17(1)	24(1)	12(1)	2(1)	7(1)	1(1)
C(20)	18(1)	16(1)	15(1)	2(1)	8(1)	1(1)
C(21)	20(1)	16(1)	16(1)	-2(1)	9(1)	1(1)
C(22)	14(1)	19(1)	11(1)	2(1)	3(1)	2(1)
C(23)	16(1)	19(1)	19(1)	6(1)	8(1)	6(1)
C(24)	29(1)	18(1)	20(1)	0(1)	16(1)	4(1)
C(25)	18(1)	19(1)	18(1)	0(1)	7(1)	-2(1)
C(2)	9(1)	18(1)	16(1)	3(1)	5(1)	2(1)

3.6 Crystal Structure Determination of Compound 8-Na

Additional information concerning the structure refinement: All hydrogen atoms were placed on ideal positions except for H1 on C1 and H26 on C26 which were found in the difference Fourier map and refined independently. One noncoordinating disordered THF molecule was squeezed with Platon (Version: 150216). Three disordered THF molecules (occupancies: 0.72 : 0.28 ; 0.78 : 0.22 ; 0.74 : 0.26) were solved using the PART, EADP and ISOR instructions.

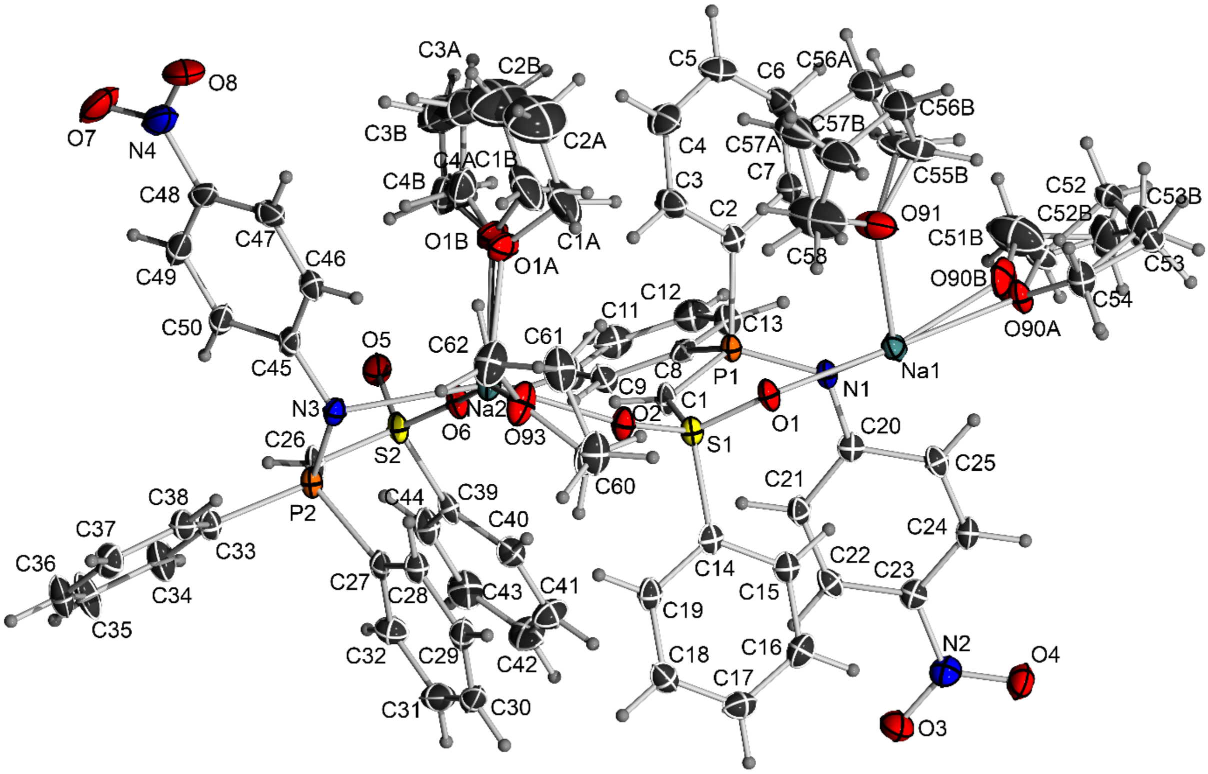

Figure S37. ORTEP Plot of **8-Na**. Ellipsoids are drawn at the 50% probability level.

Table S12. Atomic coordinates (x 10^4) and equivalent isotropic displacement parameters ($Å^2$x 10^3) for **8-Na**. U(eq) is defined as one third of the trace of the orthogonalized U_{ij} tensor.

	x	y	z	U(eq)
S(1)	-2928(1)	689(1)	2374(1)	16(1)
S(2)	-7735(1)	187(1)	2368(1)	17(1)
P(1)	-3451(1)	1569(1)	1239(1)	14(1)
P(2)	-8088(1)	-384(1)	3510(1)	16(1)
Na(1)	-525(1)	1992(1)	1603(1)	20(1)
Na(2)	-5813(1)	1211(1)	3187(1)	20(1)
O(1)	-1888(2)	1300(1)	2267(1)	19(1)
O(2)	-3738(2)	803(1)	2834(1)	21(1)
O(3)	338(2)	-3502(2)	767(1)	35(1)
O(4)	1966(2)	-2746(2)	472(1)	31(1)
O(5)	-8613(2)	826(1)	1949(1)	23(1)
O(6)	-6756(2)	707(1)	2518(1)	20(1)
O(7)	-12888(2)	4494(2)	3648(1)	57(1)

O(8)	-11423(2)	5186(2)	3929(1)	68(1)
O(9A)	874(7)	2735(8)	968(3)	20(1)
O(10)	-1145(2)	3707(2)	1797(1)	40(1)
N(1)	-1935(2)	1324(2)	1049(1)	16(1)
N(2)	840(2)	-2691(2)	651(1)	24(1)
N(3)	-7856(2)	692(2)	3679(1)	17(1)
N(4)	-11745(3)	4436(2)	3775(1)	48(1)
C(1)	-3853(2)	930(2)	1853(1)	18(1)
C(2)	-3914(2)	3053(2)	1131(1)	16(1)
C(3)	-4808(3)	3525(2)	1464(1)	26(1)
C(4)	-5331(3)	4641(2)	1345(1)	34(1)
C(5)	-4983(3)	5301(2)	889(1)	28(1)
C(6)	-4066(3)	4842(2)	561(1)	28(1)
C(7)	-3526(2)	3730(2)	680(1)	23(1)
C(8)	-4543(2)	1308(2)	794(1)	16(1)
C(9)	-5607(2)	828(2)	962(1)	22(1)
C(10)	-6459(3)	726(2)	603(1)	29(1)
C(11)	-6259(3)	1081(2)	76(1)	29(1)
C(12)	-5189(3)	1537(2)	-96(1)	26(1)
O(12)	-4656(2)	1228(2)	3936(1)	31(1)
C(13)	-4333(2)	1651(2)	259(1)	22(1)
C(14)	-2098(2)	-722(2)	2518(1)	17(1)
C(15)	-961(2)	-1057(2)	2248(1)	19(1)
C(16)	-339(2)	-2156(2)	2343(1)	25(1)
C(17)	-864(3)	-2919(2)	2706(1)	26(1)
C(18)	-1992(2)	-2589(2)	2976(1)	24(1)
C(19)	-2608(2)	-1488(2)	2889(1)	20(1)
C(20)	-1331(2)	329(2)	944(1)	16(1)
C(21)	-1869(2)	-619(2)	1036(1)	19(1)
C(22)	-1168(2)	-1590(2)	937(1)	19(1)
C(23)	96(2)	-1655(2)	734(1)	18(1)
C(24)	654(2)	-741(2)	626(1)	19(1)
C(25)	-42(2)	225(2)	731(1)	19(1)
C(26)	-8565(2)	-302(2)	2890(1)	20(1)
C(27)	-6526(2)	-1347(2)	3673(1)	18(1)
C(28)	-5681(2)	-1198(2)	4022(1)	21(1)
C(29)	-4555(2)	-1997(2)	4177(1)	24(1)
C(30)	-4279(3)	-2937(2)	3983(1)	26(1)
C(31)	-5116(3)	-3082(2)	3632(1)	31(1)
C(32)	-6246(3)	-2293(2)	3476(1)	24(1)
C(33)	-9260(2)	-1019(2)	3943(1)	19(1)
C(34)	-10025(3)	-1652(2)	3795(1)	27(1)

C(35)	-10827(3)	-2173(3)	4156(1)	33(1)
C(36)	-10870(3)	-2071(2)	4669(1)	31(1)
C(37)	-10113(3)	-1443(2)	4820(1)	25(1)
C(38)	-9312(2)	-918(2)	4461(1)	20(1)
C(39)	-6799(2)	-911(2)	2106(1)	18(1)
C(40)	-5505(2)	-1354(2)	2256(1)	22(1)
C(41)	-4781(3)	-2205(2)	2055(1)	28(1)
C(42)	-5337(3)	-2623(2)	1703(1)	32(1)
C(43)	-6623(3)	-2180(2)	1551(1)	31(1)
C(44)	-7357(3)	-1317(2)	1748(1)	25(1)
C(45)	-8849(2)	1579(2)	3688(1)	19(1)
C(46)	-8567(3)	2457(2)	3877(1)	24(1)
C(47)	-9502(3)	3381(2)	3904(1)	29(1)
C(48)	-10768(3)	3464(2)	3749(1)	31(1)
C(49)	-11097(3)	2632(2)	3565(1)	32(1)
C(50)	-10161(2)	1711(2)	3533(1)	24(1)
C(51)	638(6)	3159(6)	415(4)	22(1)
C(52)	1400(4)	4074(4)	253(2)	22(1)
C(53)	2547(5)	3658(5)	623(2)	20(1)
C(54)	1901(3)	3147(2)	1111(1)	31(1)
C(58)	-1762(5)	3853(3)	2275(1)	71(1)
C(63)	-3299(3)	646(2)	4002(1)	30(1)
C(64)	-2842(3)	852(3)	4499(1)	33(1)
C(65)	-3717(3)	1953(3)	4527(1)	33(1)
C(66)	-5004(3)	1848(3)	4329(1)	36(1)
C(55A)	-1349(5)	4756(4)	1433(2)	29(1)
C(56A)	-1881(6)	5609(4)	1737(2)	44(1)
C(57A)	-2490(7)	4983(4)	2212(2)	61(2)
C(55B)	-927(15)	4772(12)	1573(6)	29(1)
C(56B)	-1200(15)	5423(12)	1832(7)	44(1)
C(57B)	-1620(20)	4780(12)	2373(7)	61(2)
O(9B)	500(30)	2930(30)	927(16)	40(7)
C(53B)	2260(30)	3790(30)	521(12)	55(5)
C(52B)	1407(16)	3470(20)	139(8)	55(5)
C(51B)	180(40)	3410(30)	425(18)	61(10)
O(11A)	-6301(6)	3055(7)	2926(3)	28(1)
C(60A)	-6287(9)	4876(6)	2898(3)	85(2)
C(61A)	-7615(7)	4859(5)	2730(3)	59(2)
C(62A)	-7405(6)	3696(7)	2625(3)	39(2)
C(59A)	-5478(7)	3790(5)	2926(3)	54(2)
O(11B)	-6580(20)	3170(20)	2836(10)	28(1)
C(59B)	-6048(19)	3947(17)	3043(9)	54(2)

C(60B)	-7030(30)	4990(20)	2777(12)	85(2)
C(61B)	-8089(19)	4673(14)	2560(8)	59(2)
C(62B)	-7730(20)	3560(30)	2542(11)	39(2)

Table S13. Anisotropic displacement parameters ($Å^2$x 10^3) for **8-Na**. The anisotropic displacement factor exponent takes the form: $-2p^2[h^2a^{*2}U^{11} + ... + 2 h k a^* b^* U^{12}]$.

	U^{11}	U^{22}	U^{33}	U^{23}	U^{13}	U^{12}
S(1)	14(1)	21(1)	13(1)	-3(1)	0(1)	-6(1)
S(2)	14(1)	24(1)	13(1)	-3(1)	0(1)	-7(1)
P(1)	12(1)	19(1)	13(1)	-3(1)	0(1)	-5(1)
P(2)	13(1)	23(1)	13(1)	-4(1)	1(1)	-7(1)
Na(1)	17(1)	21(1)	22(1)	-3(1)	-1(1)	-8(1)
Na(2)	16(1)	26(1)	21(1)	-9(1)	2(1)	-7(1)
O(1)	16(1)	25(1)	20(1)	-4(1)	1(1)	-10(1)
O(2)	19(1)	29(1)	14(1)	-6(1)	3(1)	-5(1)
O(3)	37(1)	23(1)	49(1)	-12(1)	11(1)	-11(1)
O(4)	23(1)	34(1)	39(1)	-17(1)	11(1)	-6(1)
O(5)	19(1)	29(1)	18(1)	-1(1)	-2(1)	-5(1)
O(6)	18(1)	27(1)	20(1)	-7(1)	2(1)	-12(1)
O(7)	33(1)	49(2)	65(2)	6(1)	5(1)	21(1)
O(8)	55(2)	18(1)	116(2)	-4(1)	28(2)	2(1)
O(9A)	17(3)	29(3)	13(2)	4(2)	-3(2)	-9(2)
O(10)	63(2)	21(1)	31(1)	-3(1)	11(1)	-4(1)
N(1)	12(1)	21(1)	16(1)	-3(1)	1(1)	-6(1)
N(2)	25(1)	25(1)	24(1)	-10(1)	0(1)	-6(1)
N(3)	13(1)	20(1)	18(1)	-4(1)	-2(1)	-2(1)
N(4)	41(2)	31(2)	51(2)	13(1)	18(1)	10(1)
C(1)	11(1)	27(1)	16(1)	-2(1)	-1(1)	-7(1)
C(2)	15(1)	21(1)	14(1)	-3(1)	-3(1)	-6(1)
C(3)	35(2)	22(1)	20(1)	-3(1)	8(1)	-6(1)
C(4)	40(2)	26(2)	34(2)	-11(1)	14(1)	-2(1)
C(5)	36(2)	17(1)	32(2)	-6(1)	-4(1)	-6(1)
C(6)	31(2)	25(2)	24(2)	1(1)	4(1)	-10(1)
C(7)	21(1)	24(1)	25(1)	-5(1)	6(1)	-6(1)
C(8)	14(1)	17(1)	17(1)	-5(1)	-2(1)	-1(1)
C(9)	21(1)	24(1)	21(1)	-1(1)	-2(1)	-8(1)
C(10)	21(1)	32(2)	37(2)	-4(1)	-7(1)	-13(1)
C(11)	29(2)	29(2)	32(2)	-10(1)	-13(1)	-5(1)
C(12)	33(2)	27(2)	18(1)	-6(1)	-5(1)	-3(1)
O(12)	20(1)	48(1)	29(1)	-20(1)	-2(1)	-7(1)
C(13)	23(1)	25(1)	18(1)	-5(1)	0(1)	-7(1)

C(14)	13(1)	24(1)	16(1)	-5(1)	-6(1)	-6(1)
C(15)	17(1)	24(1)	16(1)	-5(1)	-2(1)	-6(1)
C(16)	19(1)	30(2)	26(2)	-9(1)	-1(1)	-2(1)
C(17)	25(1)	21(1)	31(2)	-5(1)	-7(1)	-1(1)
C(18)	24(1)	25(2)	22(1)	1(1)	-3(1)	-10(1)
C(19)	16(1)	28(2)	18(1)	-3(1)	-2(1)	-6(1)
C(20)	16(1)	21(1)	11(1)	-4(1)	-4(1)	-4(1)
C(21)	13(1)	23(1)	20(1)	-3(1)	-1(1)	-6(1)
C(22)	21(1)	22(1)	17(1)	-2(1)	-2(1)	-10(1)
C(23)	20(1)	22(1)	13(1)	-6(1)	-1(1)	-4(1)
C(24)	15(1)	25(1)	17(1)	-6(1)	2(1)	-6(1)
C(25)	19(1)	23(1)	19(1)	-5(1)	-1(1)	-10(1)
C(26)	13(1)	31(2)	16(1)	-4(1)	0(1)	-11(1)
C(27)	16(1)	22(1)	15(1)	-2(1)	4(1)	-6(1)
C(28)	20(1)	26(1)	18(1)	-4(1)	3(1)	-7(1)
C(29)	17(1)	30(2)	23(1)	1(1)	1(1)	-4(1)
C(30)	21(1)	24(2)	23(1)	6(1)	5(1)	3(1)
C(31)	39(2)	20(2)	30(2)	-3(1)	6(1)	-1(1)
C(32)	30(1)	21(1)	23(1)	-5(1)	-1(1)	-5(1)
C(33)	16(1)	25(1)	16(1)	-1(1)	-1(1)	-6(1)
C(34)	27(1)	44(2)	14(1)	-5(1)	0(1)	-18(1)
C(35)	31(2)	53(2)	25(2)	-6(1)	0(1)	-30(1)
C(36)	25(1)	45(2)	24(2)	-2(1)	5(1)	-19(1)
C(37)	26(1)	32(2)	16(1)	-4(1)	3(1)	-5(1)
C(38)	20(1)	22(1)	17(1)	-4(1)	1(1)	-5(1)
C(39)	20(1)	24(1)	12(1)	-2(1)	3(1)	-11(1)
C(40)	22(1)	24(1)	21(1)	-4(1)	-4(1)	-7(1)
C(41)	26(1)	28(2)	31(2)	-8(1)	-6(1)	-2(1)
C(42)	35(2)	32(2)	33(2)	-15(1)	1(1)	-6(1)
C(43)	34(2)	36(2)	28(2)	-16(1)	-1(1)	-13(1)
C(44)	22(1)	36(2)	20(1)	-5(1)	0(1)	-11(1)
C(45)	16(1)	23(1)	13(1)	1(1)	5(1)	-2(1)
C(46)	21(1)	25(1)	23(1)	-1(1)	6(1)	-6(1)
C(47)	36(2)	19(1)	28(2)	0(1)	13(1)	-5(1)
C(48)	28(2)	21(2)	30(2)	8(1)	11(1)	5(1)
C(49)	19(1)	40(2)	24(2)	7(1)	4(1)	4(1)
C(50)	16(1)	28(2)	23(1)	-2(1)	2(1)	-2(1)
C(51)	28(3)	19(3)	17(3)	1(2)	-3(3)	-7(2)
C(52)	22(2)	18(2)	23(2)	1(2)	0(2)	-6(2)
C(53)	17(3)	20(2)	22(3)	1(2)	-2(2)	-4(2)
C(54)	25(2)	37(2)	31(2)	-6(1)	4(1)	-11(1)
C(58)	126(4)	46(2)	34(2)	-10(2)	31(2)	-13(2)

C(63)	21(1)	41(2)	32(2)	-15(1)	-4(1)	-5(1)
C(64)	26(2)	49(2)	25(2)	-10(1)	-2(1)	-12(1)
C(65)	31(2)	51(2)	24(2)	-16(1)	3(1)	-18(1)
C(66)	31(2)	52(2)	32(2)	-24(2)	2(1)	-9(1)
C(55A)	39(3)	21(2)	24(3)	2(2)	-13(2)	-5(2)
C(56A)	43(3)	25(2)	59(3)	-7(2)	20(3)	-5(3)
C(57A)	83(5)	26(3)	71(4)	-13(3)	41(4)	-12(3)
C(55B)	39(3)	21(2)	24(3)	2(2)	-13(2)	-5(2)
C(56B)	43(3)	25(2)	59(3)	-7(2)	20(3)	-5(3)
C(57B)	83(5)	26(3)	71(4)	-13(3)	41(4)	-12(3)
O(9B)	17(13)	50(16)	52(12)	-3(10)	-6(10)	-16(10)
C(53B)	35(9)	62(11)	47(10)	22(8)	11(7)	-8(8)
C(52B)	35(9)	62(11)	47(10)	22(8)	11(7)	-8(8)
C(51B)	100(30)	47(19)	39(14)	-4(14)	6(19)	-21(16)
O(11A)	19(3)	23(2)	41(3)	-3(2)	-10(2)	-1(2)
C(60A)	100(4)	59(3)	108(4)	-36(3)	-12(4)	-20(3)
C(61A)	59(4)	45(3)	60(4)	-11(2)	8(3)	9(3)
C(62A)	18(4)	50(3)	40(4)	4(2)	0(3)	-4(3)
C(59A)	59(5)	50(3)	64(4)	-18(3)	20(3)	-39(4)
O(11B)	19(3)	23(2)	41(3)	-3(2)	-10(2)	-1(2)
C(59B)	59(5)	50(3)	64(4)	-18(3)	20(3)	-39(4)
C(60B)	100(4)	59(3)	108(4)	-36(3)	-12(4)	-20(3)
C(61B)	59(4)	45(3)	60(4)	-11(2)	8(3)	9(3)
C(62B)	18(4)	50(3)	40(4)	4(2)	0(3)	-4(3)

3.7 Crystal Structure Determination of Compound 8

Additional information concerning the structure refinement: All hydrogen atoms were placed on ideal positions except for H1 on C1 which was found in the difference Fourier map and refined independently.

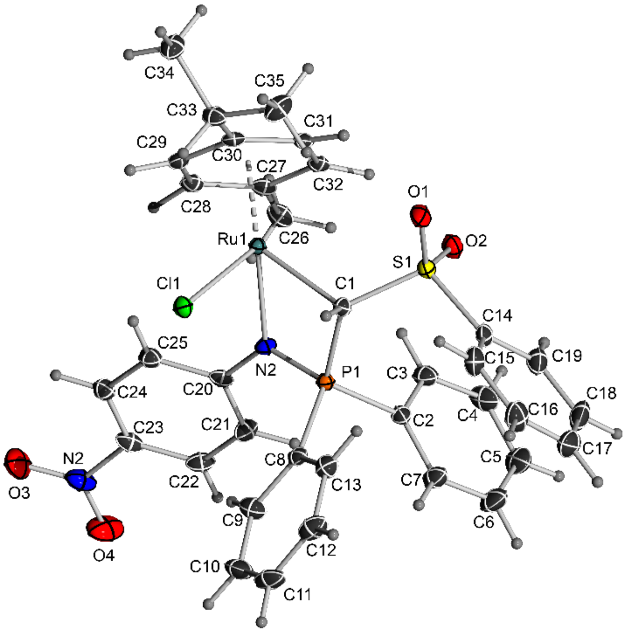

Figure S38. ORTEP Plot of **9**. Ellipsoids are drawn at the 50% probability level.

Table S14. Atomic coordinates (x 10^4) and equivalent isotropic displacement parameters ($Å^2$x 10^3) for **9**. U(eq) is defined as one third of the trace of the orthogonalized U_{ij} tensor.

	x	y	z	U(eq)
Ru(1)	3626(1)	962(1)	8236(1)	10(1)
O(1)	4194(1)	-1751(1)	7698(1)	21(1)
N(1)	4179(1)	1041(1)	8838(1)	14(1)
P(1)	4379(1)	-270(1)	8892(1)	12(1)
S(1)	4693(1)	-1398(1)	8040(1)	15(1)
C(1)	4057(1)	-721(2)	8391(1)	13(1)
Cl(1)	2433(1)	330(1)	8597(1)	16(1)
C(4)	6794(1)	-100(2)	8894(1)	26(1)
O(4)	4601(1)	4359(1)	10341(1)	36(1)
C(5)	7021(1)	-929(2)	9169(1)	29(1)
C(6)	6450(1)	-1559(2)	9367(1)	28(1)
C(7)	5645(1)	-1376(2)	9289(1)	21(1)
C(8)	3840(1)	-1006(2)	9293(1)	15(1)
C(9)	3702(1)	-510(2)	9678(1)	22(1)
C(10)	3373(1)	-1133(2)	9997(1)	27(1)
C(11)	3184(1)	-2243(2)	9936(1)	27(1)
C(12)	3297(1)	-2738(2)	9554(1)	25(1)
C(13)	3625(1)	-2119(2)	9233(1)	20(1)

C(14)	5039(1)	-2627(2)	8287(1)	16(1)
C(15)	4498(1)	-3471(2)	8376(1)	23(1)
C(16)	4749(1)	-4404(2)	8594(1)	28(1)
C(17)	5538(1)	-4498(2)	8712(1)	30(1)
C(18)	6078(1)	-3668(2)	8610(1)	29(1)
C(19)	5832(1)	-2718(2)	8400(1)	22(1)
C(20)	4180(1)	1883(2)	9137(1)	14(1)
C(21)	4784(1)	1972(2)	9438(1)	19(1)
C(22)	4761(1)	2802(2)	9736(1)	20(1)
C(23)	4133(1)	3554(2)	9735(1)	19(1)
C(24)	3546(1)	3522(2)	9433(1)	18(1)
C(25)	3576(1)	2688(2)	9136(1)	17(1)
C(26)	5017(1)	2951(2)	8217(1)	21(1)
C(27)	4333(1)	2380(2)	8006(1)	16(1)
C(28)	3530(1)	2758(2)	8068(1)	16(1)
C(29)	2894(1)	2165(2)	7891(1)	16(1)
C(30)	3016(1)	1202(2)	7634(1)	14(1)
C(31)	3811(1)	869(2)	7565(1)	15(1)
C(32)	4464(1)	1449(2)	7746(1)	15(1)
C(33)	2301(1)	601(2)	7456(1)	18(1)
C(34)	1889(1)	1357(2)	7139(1)	22(1)
C(35)	2504(1)	-522(2)	7262(1)	24(1)
C(3)	5995(1)	105(2)	8818(1)	20(1)
O(3)	3514(1)	5025(1)	10074(1)	31(1)
C(2)	5416(1)	-543(2)	9012(1)	15(1)
N(2)	4080(1)	4373(2)	10070(1)	24(1)
O(2)	5381(1)	-718(1)	7951(1)	20(1)

Table S15. Anisotropic displacement parameters (Å^{2}x 10^3) for **9**. The anisotropic displacement factor exponent takes the form: $-2p^2[h^2a^{*2}U^{11} + ... + 2 h k a^* b^* U^{12}]$.

	U^{11}	U^{22}	U^{33}	U^{23}	U^{13}	U^{12}
Ru(1)	11(1)	12(1)	9(1)	0(1)	0(1)	0(1)
O(1)	23(1)	25(1)	16(1)	-7(1)	-3(1)	7(1)
N(1)	14(1)	15(1)	12(1)	0(1)	-3(1)	-1(1)
P(1)	11(1)	14(1)	10(1)	1(1)	0(1)	0(1)
S(1)	14(1)	16(1)	13(1)	-2(1)	1(1)	3(1)
C(1)	12(1)	14(1)	12(1)	0(1)	0(1)	0(1)
Cl(1)	12(1)	20(1)	17(1)	2(1)	2(1)	0(1)
C(4)	15(1)	41(1)	22(1)	4(1)	1(1)	-7(1)
O(4)	56(1)	31(1)	22(1)	-9(1)	-12(1)	-2(1)
C(5)	14(1)	45(1)	27(1)	3(1)	-5(1)	3(1)

C(6)	22(1)	35(1)	27(1)	9(1)	-7(1)	4(1)
C(7)	16(1)	25(1)	22(1)	7(1)	-1(1)	0(1)
C(8)	10(1)	21(1)	14(1)	5(1)	-1(1)	1(1)
C(9)	23(1)	26(1)	16(1)	0(1)	0(1)	-3(1)
C(10)	25(1)	42(1)	14(1)	3(1)	2(1)	-2(1)
C(11)	19(1)	39(1)	23(1)	16(1)	2(1)	-4(1)
C(12)	20(1)	24(1)	30(1)	11(1)	-2(1)	-4(1)
C(13)	20(1)	20(1)	20(1)	2(1)	-1(1)	0(1)
C(14)	17(1)	15(1)	17(1)	-3(1)	1(1)	5(1)
C(15)	19(1)	20(1)	30(1)	-4(1)	2(1)	3(1)
C(16)	31(1)	17(1)	37(1)	2(1)	9(1)	4(1)
C(17)	41(1)	22(1)	27(1)	1(1)	2(1)	13(1)
C(18)	23(1)	31(1)	34(1)	-5(1)	-6(1)	14(1)
C(19)	17(1)	24(1)	25(1)	-3(1)	0(1)	4(1)
C(20)	17(1)	14(1)	11(1)	1(1)	1(1)	-4(1)
C(21)	20(1)	18(1)	19(1)	0(1)	-5(1)	1(1)
C(22)	23(1)	20(1)	15(1)	1(1)	-6(1)	-5(1)
C(23)	28(1)	15(1)	13(1)	-2(1)	2(1)	-6(1)
C(24)	21(1)	17(1)	17(1)	1(1)	3(1)	0(1)
C(25)	16(1)	21(1)	14(1)	0(1)	-1(1)	-1(1)
C(26)	23(1)	21(1)	20(1)	-1(1)	3(1)	-4(1)
C(27)	20(1)	15(1)	13(1)	4(1)	2(1)	-3(1)
C(28)	24(1)	13(1)	12(1)	3(1)	2(1)	0(1)
C(29)	17(1)	18(1)	13(1)	6(1)	0(1)	3(1)
C(30)	18(1)	16(1)	9(1)	4(1)	-2(1)	0(1)
C(31)	19(1)	17(1)	9(1)	2(1)	0(1)	0(1)
C(32)	17(1)	16(1)	12(1)	5(1)	2(1)	-1(1)
C(33)	15(1)	24(1)	14(1)	1(1)	-2(1)	-3(1)
C(34)	22(1)	23(1)	22(1)	-5(1)	-7(1)	3(1)
C(35)	23(1)	20(1)	29(1)	0(1)	-10(1)	-3(1)
C(3)	19(1)	26(1)	15(1)	2(1)	-2(1)	-2(1)
O(3)	43(1)	24(1)	28(1)	-9(1)	3(1)	5(1)
C(2)	12(1)	20(1)	12(1)	-2(1)	-1(1)	1(1)
N(2)	37(1)	18(1)	16(1)	-2(1)	1(1)	-6(1)
O(2)	18(1)	20(1)	22(1)	1(1)	6(1)	1(1)

3.8 Crystal Structure Determination of Compound 2b

Additional information concerning the structure refinement: All hydrogen atoms were placed on ideal positions. The highly disordered Ru-Cymene moieties (occupancy: 0.80 : 0.20 ; 0.95 : 0.05) were solved using the PART, RIGU, FLAT, EADP and SAME instructions. Additionally, a twin refinement with the TWIN and BASF instructions was conducted.

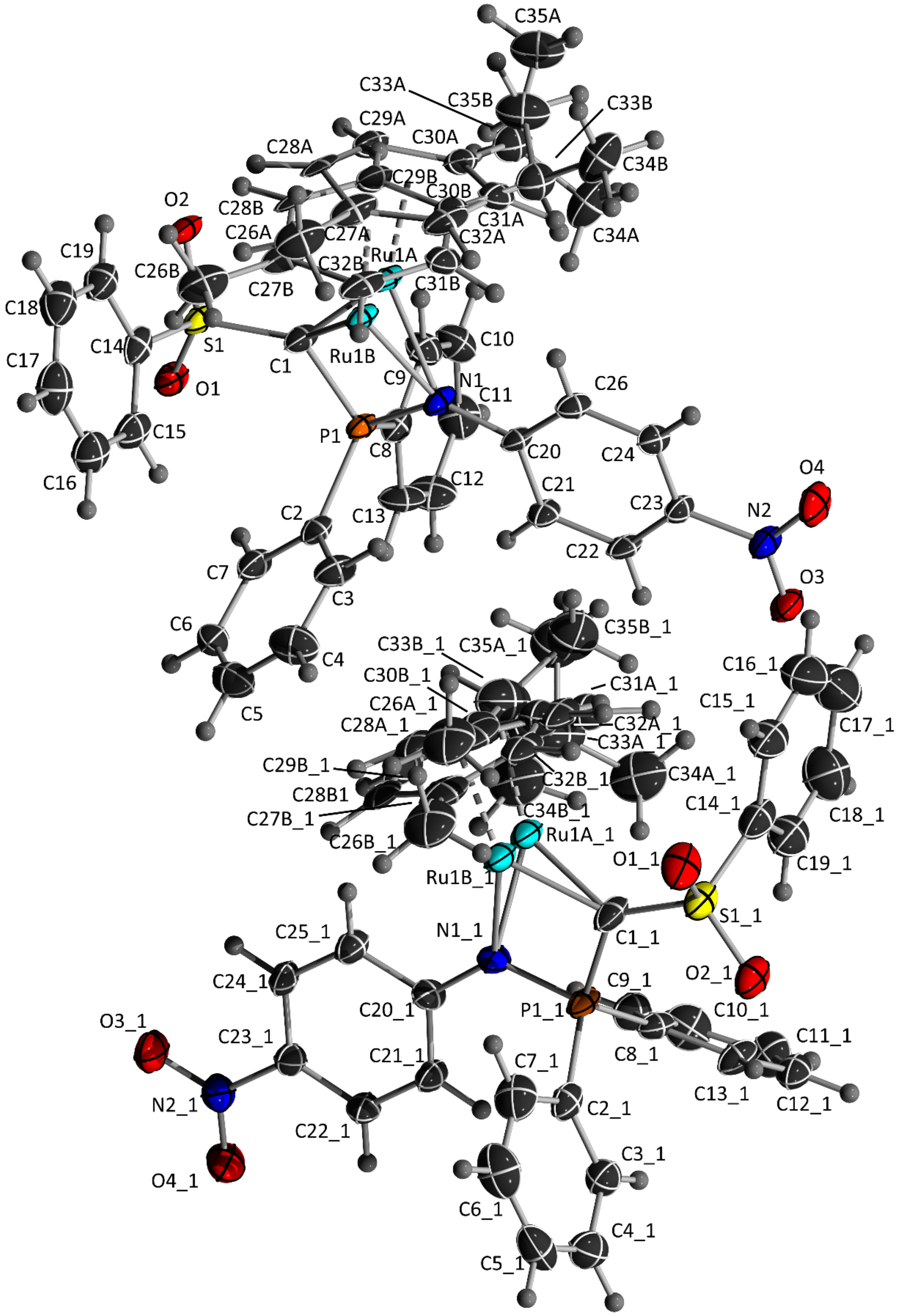

Figure S39. ORTEP Plot of **2b**. Ellipsoids are drawn at the 50% probability level.

Table S16. Atomic coordinates (x 10^4) and equivalent isotropic displacement parameters (Å^{2}x 10^3) for **2b**. U(eq) is defined as one third of the trace of the orthogonalized U_{ij} tensor.

	x	y	z	U(eq)
O3	631(5)	7881(3)	2379(4)	38.9(12)
O4	1228(5)	6881(2)	2796(4)	36.7(11)
O2	7559(5)	9929(2)	7407(4)	29.9(10)
C25	3372(6)	8807(3)	3815(4)	23.5(13)
C19	9050(7)	10337(4)	5797(6)	35.8(17)
N2	1342(5)	7500(3)	2796(4)	28.8(12)
S1	7723.0(14)	9365.3(7)	6725.1(11)	23.4(3)
P1	5991.2(14)	8414.1(7)	5684.0(11)	20.1(3)
N1	4984(5)	8737(2)	4946(4)	21.6(10)
O1	8186(4)	8737(2)	7148(3)	28.5(10)
C1	6499(6)	9207(3)	6043(5)	27.0(13)
C4	7806(8)	7508(4)	3531(7)	46.5(19)
C3	6978(7)	7866(3)	4018(5)	31.3(14)
C2	6976(6)	7862(3)	5054(5)	24.0(12)
C10	4159(7)	7929(4)	8102(6)	39.3(17)
C9	4704(7)	8268(3)	7357(6)	31.3(15)
C8	5375(6)	7925(3)	6687(5)	23.8(13)
C7	7820(7)	7496(3)	5586(6)	32.5(15)
C6	8654(7)	7136(4)	5079(6)	36.2(16)
C5	8642(7)	7144(4)	4049(7)	43.0(18)
C13	5498(7)	7217(3)	6792(5)	35.2(16)
C12	4975(8)	6879(4)	7547(6)	42.3(18)
C11	4299(8)	7222(4)	8209(6)	40.9(18)
C24	2475(6)	8514(3)	3277(5)	23.8(12)
C18	9783(8)	10551(4)	5061(7)	44.9(19)
C20	4140(6)	8414(3)	4405(4)	19.7(12)
C14	8730(6)	9656(3)	5820(5)	28.4(14)
C23	2319(6)	7809(3)	3312(4)	22.5(12)
C22	3085(6)	7398(3)	3852(5)	25.2(13)
C16	9856(7)	9407(4)	4408(6)	40.4(17)
C21	3984(6)	7689(3)	4387(5)	24.8(13)
C15	9133(7)	9193(4)	5136(5)	34.8(16)
C17	10171(8)	10088(5)	4360(6)	46.2(19)
Ru1A	5264.9(4)	9760.3(2)	5457.5(3)	21.79(14)
C26A	6516(9)	10898(4)	4069(6)	45(2)

C27A	5619(7)	10737(3)	4767(5)	30.4(16)
C28A	5806(7)	10797(3)	5821(5)	26.8(14)
C29A	4972(7)	10586(3)	6486(5)	29.4(14)
C30A	3866(6)	10338(3)	6135(5)	26.9(14)
C31A	3631(7)	10358(3)	5107(6)	30.4(15)
C32A	4478(8)	10546(3)	4416(6)	34.6(18)
C33A	3018(7)	10068(4)	6890(6)	35.8(16)
C34A	2225(9)	9517(5)	6438(7)	50(2)
C35A	2347(9)	10652(4)	7338(7)	49(2)
Ru1B	5801(9)	9722(4)	4863(7)	21.79(14)
C26B	7920(50)	10690(30)	4200(70)	45(2)
C27B	6720(40)	10625(18)	4450(40)	30.4(16)
C28B	6310(50)	10707(19)	5440(40)	26.8(14)
C29B	5150(50)	10620(20)	5610(40)	29.4(14)
C30B	4320(30)	10460(30)	4840(50)	26.9(14)
C31B	4700(40)	10420(30)	3880(40)	30.4(15)
C32B	5880(50)	10490(30)	3660(30)	34.6(18)
C33B	3070(40)	10380(40)	5130(60)	35.8(16)
C34B	2240(60)	10720(70)	4380(80)	50(2)
C35B	2880(90)	10600(70)	6200(60)	49(2)
C5_1	6589(7)	2451(5)	1170(6)	49(2)
C4_1	5835(8)	2081(4)	1702(6)	41.2(18)
O4_1	9734(5)	1826(3)	6572(5)	42.5(13)
C3_1	5315(7)	2356(4)	2520(6)	36.0(16)
O3_1	10347(5)	2823(3)	7036(5)	47.3(14)
C2_1	5552(7)	3021(4)	2790(5)	30.4(15)
O2_1	2796(5)	3831(3)	2337(4)	36.4(11)
N2_1	9633(5)	2454(3)	6607(5)	33.1(13)
C1_1	4320(7)	4238(3)	3638(6)	36.2(17)
S1_1	3193.3(16)	4430.6(8)	2879.7(13)	33.0(4)
N1_1	5858(5)	3717(3)	4675(5)	34.3(14)
P1_1	4905.6(15)	3431.5(8)	3856.2(13)	26.2(3)
O1_1	3440(5)	5029(3)	2293(5)	43.9(14)
C8_1	3926(6)	2822(3)	4382(5)	26.3(13)
C7_1	6296(8)	3412(4)	2236(7)	47(2)
C6_1	6841(9)	3127(6)	1433(7)	55(2)
C9_1	3009(7)	2560(3)	3808(5)	31.9(15)
C10_1	2231(7)	2118(4)	4222(6)	35.1(16)

C13_1	4068(7)	2613(4)	5364(5)	35.3(15)
C12_1	3301(8)	2164(5)	5763(6)	46.4(19)
C11_1	2355(8)	1934(4)	5200(7)	43.9(18)
C14_1	2089(8)	4674(4)	3714(7)	40.6(18)
C17_1	468(11)	5084(8)	5082(10)	75(3)
C16_1	935(10)	5544(6)	4448(9)	64(3)
C15_1	1718(8)	5349(4)	3747(8)	50(2)
C18_1	856(10)	4374(6)	5018(9)	66(3)
C20_1	6762(6)	3384(3)	5134(5)	27.6(14)
C19_1	1636(8)	4192(5)	4338(7)	47(2)
C21_1	6960(6)	2676(3)	5072(5)	28.4(14)
C23_1	8649(6)	2769(3)	6105(5)	26.7(13)
C22_1	7886(6)	2368(3)	5556(5)	27.2(13)
C25_1	7572(7)	3774(3)	5716(5)	31.9(15)
C24_1	8491(6)	3471(4)	6200(5)	29.7(14)
Ru1A_1	5053.5(6)	4691.1(3)	4820.8(5)	32.1(2)
C26A_1	5964(13)	6118(5)	3849(11)	64(3)
C27A_1	5481(11)	5752(4)	4726(9)	48(3)
C28A_1	6205(10)	5496(4)	5524(9)	44(3)
C29A_1	5741(10)	5142(6)	6281(9)	50(3)
C30A_1	4510(10)	5006(6)	6342(8)	45(2)
C31A_1	3777(10)	5309(5)	5597(9)	45(2)
C32A_1	4279(10)	5683(5)	4819(10)	46(3)
C33A_1	4031(14)	4554(7)	7164(10)	67(4)
C34A_1	3106(13)	4098(7)	6826(10)	63(3)
C35A_1	3656(13)	5033(8)	8002(10)	64(3)
Ru1B_1	5555(3)	4779.2(13)	4262(2)	32.1(2)
C26B_1	6600(40)	5873(16)	2750(20)	64(3)
C27B_1	6190(30)	5721(10)	3726(19)	48(3)
C28B_1	6900(20)	5515(13)	4570(20)	44(3)
C29B_1	6430(30)	5337(19)	5480(20)	50(3)
C30B_1	5240(30)	5415(15)	5669(16)	45(2)
C31B_1	4540(30)	5663(19)	4900(20)	45(2)
C32B_1	4990(30)	5824(12)	3960(20)	46(3)
C33B_1	4740(40)	5190(19)	6660(20)	67(4)
C34B_1	4750(50)	4407(18)	6750(40)	63(3)
C35B_1	3590(40)	5510(30)	6880(40)	64(3)

Table S17. Anisotropic displacement parameters (Å^{2}x 10^3) for **8**. The anisotropic displacement factor exponent takes the form: $-2p^2[h^2a^{*2}U^{11} + ... + 2 h k a^* b^* U^{12}]$.

	U^{11}	U^{22}	U^{33}	U^{23}	U^{13}	U^{12}
O3	37(3)	38(3)	41(3)	5(2)	-22(2)	-1(2)
O4	35(3)	24(2)	51(3)	-6(2)	-10(2)	-5(2)
O2	40(3)	20(2)	29(2)	-6.8(18)	-12(2)	-3(2)
C25	34(3)	16(3)	21(3)	0(2)	-4(3)	3(2)
C19	36(4)	29(4)	42(4)	2(3)	-7(3)	-4(3)
N2	32(3)	26(3)	28(3)	-3(2)	-8(2)	-5(2)
S1	30.6(8)	17.0(6)	22.0(7)	-1.0(5)	-11.2(6)	0.2(6)
P1	26.9(8)	13.4(6)	19.3(7)	-0.7(5)	-9.1(6)	0.5(6)
N1	30(3)	17(2)	17(2)	0.4(18)	-8(2)	-2(2)
O1	37(3)	23(2)	25(2)	1.2(17)	-11.3(19)	1.9(19)
C1	37(4)	18(3)	25(3)	-3(2)	-10(3)	-1(3)
C4	55(5)	43(4)	42(4)	2(3)	14(4)	3(4)
C3	47(4)	22(3)	25(3)	2(2)	-3(3)	1(3)
C2	30(3)	15(3)	26(3)	-1(2)	-4(3)	-4(2)
C10	38(4)	43(4)	37(4)	-1(3)	6(3)	9(3)
C9	34(4)	24(3)	36(4)	3(3)	-3(3)	8(3)
C8	29(3)	20(3)	22(3)	4(2)	-6(3)	-3(2)
C7	39(4)	24(3)	34(4)	-6(3)	-15(3)	1(3)
C6	29(4)	29(3)	49(4)	-8(3)	-10(3)	7(3)
C5	40(4)	37(4)	53(5)	-2(3)	16(4)	3(3)
C13	52(4)	20(3)	35(4)	-2(3)	1(3)	11(3)
C12	59(5)	21(3)	48(4)	8(3)	6(4)	6(3)
C11	43(5)	41(4)	38(4)	12(3)	1(3)	-1(3)
C24	30(3)	21(3)	20(3)	1(2)	-6(2)	3(2)
C18	40(5)	38(4)	57(5)	9(4)	-9(4)	-6(3)
C20	28(3)	12(3)	20(3)	-2(2)	-2(2)	-1(2)
C14	29(3)	27(3)	28(3)	7(2)	-14(3)	-3(3)
C23	26(3)	22(3)	20(3)	-4(2)	-6(2)	-2(2)
C22	35(4)	11(3)	29(3)	-3(2)	-6(3)	1(2)
C16	41(4)	49(4)	31(4)	-1(3)	-6(3)	-1(3)
C21	30(3)	13(3)	32(3)	1(2)	-7(3)	1(2)
C15	41(4)	35(4)	28(3)	-4(3)	-12(3)	-2(3)
C17	39(4)	63(5)	37(4)	10(4)	-6(3)	-7(4)
Ru1A	31.3(3)	11.2(2)	22.2(2)	-0.84(18)	-11.50(19)	1.3(2)
C26A	68(6)	31(4)	35(4)	7(3)	-2(4)	-2(4)
C27A	60(5)	12(3)	19(3)	8(2)	-8(3)	-3(3)

C28A	44(4)	6(2)	30(4)	-7(2)	-14(3)	0(3)
C29A	39(4)	24(3)	25(3)	-9(2)	-8(3)	3(3)
C30A	32(4)	22(3)	27(3)	-9(2)	-4(3)	5(3)
C31A	34(4)	22(3)	35(4)	-5(3)	-7(3)	10(3)
C32A	60(5)	11(3)	32(4)	2(3)	-19(4)	6(3)
C33A	43(4)	36(4)	28(4)	1(3)	-5(3)	2(3)
C34A	59(6)	53(5)	39(4)	-1(4)	-6(4)	-19(4)
C35A	68(6)	38(4)	43(5)	1(3)	11(4)	7(4)
Ru1B	31.3(3)	11.2(2)	22.2(2)	-0.84(18)	-11.50(19)	1.3(2)
C26B	68(6)	31(4)	35(4)	7(3)	-2(4)	-2(4)
C27B	60(5)	12(3)	19(3)	8(2)	-8(3)	-3(3)
C28B	44(4)	6(2)	30(4)	-7(2)	-14(3)	0(3)
C29B	39(4)	24(3)	25(3)	-9(2)	-8(3)	3(3)
C30B	32(4)	22(3)	27(3)	-9(2)	-4(3)	5(3)
C31B	34(4)	22(3)	35(4)	-5(3)	-7(3)	10(3)
C32B	60(5)	11(3)	32(4)	2(3)	-19(4)	6(3)
C33B	43(4)	36(4)	28(4)	1(3)	-5(3)	2(3)
C34B	59(6)	53(5)	39(4)	-1(4)	-6(4)	-19(4)
C35B	68(6)	38(4)	43(5)	1(3)	11(4)	7(4)
C5_1	35(4)	76(6)	36(4)	-1(4)	0(3)	17(4)
C4_1	45(5)	45(4)	34(4)	-7(3)	-8(3)	8(4)
O4_1	40(3)	31(3)	55(3)	7(2)	-11(3)	5(2)
C3_1	36(4)	35(4)	37(4)	-3(3)	-3(3)	0(3)
O3_1	40(3)	41(3)	59(4)	-6(3)	-25(3)	5(2)
C2_1	32(4)	29(3)	29(3)	3(3)	-11(3)	-2(3)
O2_1	41(3)	32(2)	35(3)	5(2)	-16(2)	-3(2)
N2_1	32(3)	35(3)	31(3)	1(2)	-7(2)	4(2)
C1_1	42(4)	19(3)	47(4)	-1(3)	-19(3)	-5(3)
S1_1	39.1(9)	23.3(8)	35.6(9)	5.1(6)	-17.1(8)	-0.4(7)
N1_1	33(3)	19(3)	51(4)	-7(2)	-16(3)	8(2)
P1_1	33.8(9)	15.6(7)	28.6(8)	0.3(6)	-11.9(7)	-0.8(6)
O1_1	47(3)	32(3)	51(3)	17(2)	-19(3)	-1(2)
C8_1	38(4)	18(3)	23(3)	-2(2)	-5(3)	4(3)
C7_1	47(5)	41(4)	54(5)	11(4)	-2(4)	-4(4)
C6_1	47(5)	72(6)	48(5)	14(4)	9(4)	-2(4)
C9_1	46(4)	25(3)	24(3)	2(2)	-7(3)	-5(3)
C10_1	36(4)	24(3)	44(4)	0(3)	-10(3)	1(3)
C13_1	42(4)	36(4)	28(3)	-1(3)	-6(3)	3(3)

C12_1	54(5)	52(5)	32(4)	9(3)	3(4)	6(4)
C11_1	43(5)	41(4)	47(5)	9(3)	8(4)	-7(3)
C14_1	45(5)	32(4)	44(4)	1(3)	-18(4)	-5(3)
C17_1	61(7)	87(8)	76(8)	-18(6)	-2(6)	17(6)
C16_1	66(7)	52(6)	73(7)	-14(5)	-9(6)	17(5)
C15_1	51(5)	32(4)	66(6)	-1(4)	-21(5)	8(4)
C18_1	60(6)	72(7)	66(7)	6(5)	4(5)	0(5)
C20_1	33(4)	21(3)	28(3)	1(2)	-10(3)	7(3)
C19_1	50(5)	38(4)	53(5)	4(4)	-11(4)	4(4)
C21_1	31(4)	27(3)	26(3)	-4(2)	-9(3)	-3(3)
C23_1	28(3)	26(3)	25(3)	-1(2)	-2(3)	2(3)
C22_1	30(3)	20(3)	32(3)	2(2)	-4(3)	1(2)
C25_1	37(4)	22(3)	36(4)	0(3)	-13(3)	-1(3)
C24_1	29(3)	29(3)	31(3)	-3(3)	-12(3)	-2(3)
Ru1A_1	34.5(4)	19.5(3)	41.5(4)	-7.1(3)	-15.3(3)	2.5(3)
C26A_1	72(8)	30(5)	90(9)	11(5)	-6(7)	-7(5)
C27A_1	69(8)	15(4)	58(6)	-5(4)	-6(5)	-8(4)
C28A_1	47(6)	13(4)	72(7)	-14(4)	-21(5)	-1(4)
C29A_1	54(7)	39(5)	54(6)	-23(5)	-28(5)	12(5)
C30A_1	49(6)	44(6)	40(5)	-16(4)	-4(4)	11(5)
C31A_1	44(6)	40(5)	50(6)	-11(4)	-3(5)	8(4)
C32A_1	53(7)	15(4)	67(7)	-8(4)	-27(5)	9(4)
C33A_1	85(10)	62(7)	52(7)	-5(6)	-14(7)	20(7)
C34A_1	81(9)	55(7)	55(7)	6(5)	21(6)	-12(6)
C35A_1	68(8)	75(8)	49(6)	-4(6)	8(6)	-5(7)
Ru1B_1	34.5(4)	19.5(3)	41.5(4)	-7.1(3)	-15.3(3)	2.5(3)
C26B_1	72(8)	30(5)	90(9)	11(5)	-6(7)	-7(5)
C27B_1	69(8)	15(4)	58(6)	-5(4)	-6(5)	-8(4)
C28B_1	47(6)	13(4)	72(7)	-14(4)	-21(5)	-1(4)
C29B_1	54(7)	39(5)	54(6)	-23(5)	-28(5)	12(5)
C30B_1	49(6)	44(6)	40(5)	-16(4)	-4(4)	11(5)
C31B_1	44(6)	40(5)	50(6)	-11(4)	-3(5)	8(4)
C32B_1	53(7)	15(4)	67(7)	-8(4)	-27(5)	9(4)
C33B_1	85(10)	62(7)	52(7)	-5(6)	-14(7)	20(7)
C34B_1	81(9)	55(7)	55(7)	6(5)	21(6)	-12(6)
C35B_1	68(8)	75(8)	49(6)	-4(6)	8(6)	-5(7)

3.9 Crystal Structure Determination of Compound 11b

Additional information concerning the structure refinement: All hydrogen atoms were placed on ideal positions except for H1 on C1 which was found in the difference Fourier map and refined independently. A disordered CF_3 (occupancy: 0.62 : 0.38) moiety was solved using the RIGU, SAME and PART instructions.

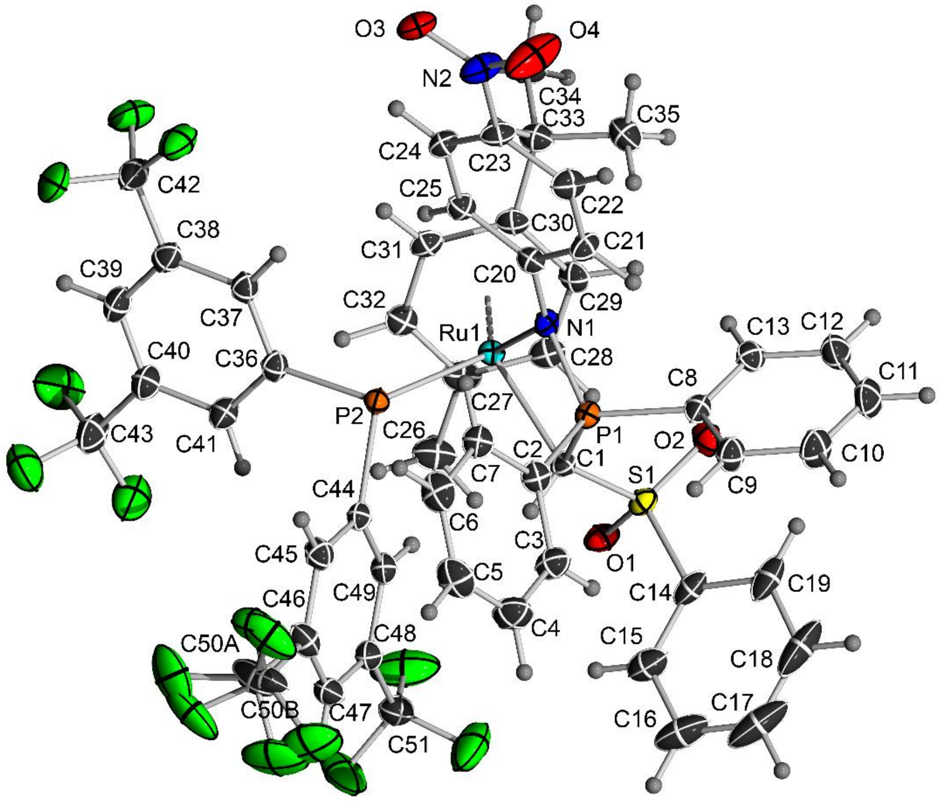

Figure S40. ORTEP Plot of **11b**. Ellipsoids are drawn at the 50% probability level.

Table S18. Atomic coordinates (x 10^4) and equivalent isotropic displacement parameters (Å^{2}x 10^3) for **11b**. U(eq) is defined as one third of the trace of the orthogonalized U_{ij} tensor.

	x	y	z	U(eq)
Ru(1)	1955(1)	4655(1)	5548(1)	15(1)
N(1)	1802(1)	4623(1)	3433(2)	16(1)
F(1)	3855(1)	2512(1)	5378(2)	37(1)
P(1)	1824(1)	5314(1)	3166(1)	16(1)
O(1)	1474(1)	6107(1)	6594(2)	24(1)
S(1)	1276(1)	5994(1)	5208(1)	19(1)
C(1)	1882(1)	5552(1)	4813(2)	16(1)
C(2)	2547(1)	5580(1)	2690(2)	20(1)
N(2)	2140(1)	2855(1)	-47(2)	30(1)
F(2)	3790(1)	2213(1)	7282(2)	43(1)
O(2)	634(1)	5756(1)	4707(2)	27(1)
P(2)	3088(1)	4672(1)	5404(1)	17(1)
O(3)	2443(1)	2416(1)	393(2)	29(1)
F(3)	4723(1)	2441(1)	6907(2)	47(1)

C(3)	2680(1)	6176(1)	2799(3)	28(1)
C(4)	3237(1)	6395(1)	2479(3)	35(1)
F(4)	4603(1)	3729(1)	10943(2)	58(1)
O(4)	1896(2)	2922(1)	-1213(2)	57(1)
C(5)	3665(1)	6027(1)	2049(3)	35(1)
F(5)	5312(1)	4165(1)	10118(2)	52(1)
C(6)	3539(1)	5438(1)	1947(3)	31(1)
F(6)	4481(1)	4627(1)	10417(2)	48(1)
C(7)	2984(1)	5212(1)	2274(2)	23(1)
F(7)	3079(1)	7101(1)	7541(2)	60(1)
C(8)	1133(1)	5566(1)	1934(2)	20(1)
F(8)	3941(1)	6931(1)	8964(2)	62(1)
C(9)	1203(1)	5946(1)	940(2)	26(1)
F(9)	3093(1)	6422(1)	8906(2)	64(1)
C(10)	655(1)	6133(1)	31(3)	33(1)
C(11)	39(1)	5946(1)	117(3)	33(1)
C(12)	-34(1)	5562(1)	1087(2)	30(1)
C(14)	1323(1)	6672(1)	4434(2)	27(1)
C(13)	511(1)	5365(1)	1988(2)	23(1)
C(16)	1883(2)	7573(1)	4405(3)	48(1)
C(15)	1823(1)	7048(1)	4998(3)	35(1)
C(17)	1443(2)	7719(1)	3264(4)	60(1)
C(18)	949(2)	7349(2)	2709(3)	55(1)
C(19)	876(2)	6814(1)	3293(3)	38(1)
C(20)	1880(1)	4190(1)	2561(2)	17(1)
C(21)	1654(1)	4252(1)	1199(2)	21(1)
C(22)	1737(1)	3816(1)	357(2)	25(1)
C(23)	2052(1)	3310(1)	859(2)	22(1)
C(24)	2276(1)	3228(1)	2192(2)	21(1)
C(25)	2185(1)	3665(1)	3030(2)	20(1)
C(26)	2170(1)	5135(1)	8693(2)	31(1)
C(27)	1885(1)	4711(1)	7632(2)	23(1)
C(28)	1250(1)	4789(1)	6822(2)	23(1)
C(29)	958(1)	4379(1)	5879(2)	22(1)
C(30)	1311(1)	3871(1)	5680(2)	20(1)
C(31)	1947(1)	3786(1)	6470(2)	21(1)
C(32)	2226(1)	4203(1)	7424(2)	22(1)
C(33)	1016(1)	3404(1)	4715(2)	24(1)
C(34)	735(1)	2931(1)	5468(3)	34(1)
C(35)	498(1)	3619(1)	3548(2)	28(1)
C(36)	3577(1)	4179(1)	6626(2)	20(1)
C(37)	3632(1)	3604(1)	6240(2)	22(1)

C(38)	4011(1)	3208(1)	7078(2)	24(1)
C(39)	4342(1)	3371(1)	8321(2)	27(1)
C(40)	4294(1)	3941(1)	8707(2)	27(1)
C(41)	3919(1)	4343(1)	7870(2)	24(1)
C(42)	4089(1)	2596(1)	6653(3)	30(1)
C(43)	4658(1)	4117(1)	10044(3)	35(1)
C(44)	3504(1)	5358(1)	6011(2)	19(1)
C(45)	4052(1)	5497(1)	5527(2)	23(1)
C(47)	4202(1)	6394(1)	6741(2)	27(1)
C(48)	3660(1)	6259(1)	7239(2)	23(1)
C(49)	3313(1)	5748(1)	6878(2)	21(1)
C(51)	3449(1)	6673(1)	8159(3)	31(1)
C(46)	4394(1)	6009(1)	5884(2)	27(1)
C(50A)	5023(7)	6124(6)	5436(13)	38(3)
F(10A)	4991(10)	5905(9)	4255(15)	59(3)
F(11A)	5135(8)	6684(5)	5360(20)	72(3)
F(12A)	5538(6)	5890(10)	6220(12)	68(3)
C(50B)	4952(11)	6161(7)	5309(19)	42(4)
F(10B)	5031(12)	5807(8)	4350(20)	41(3)
F(11B)	4913(15)	6694(5)	4800(30)	75(5)
F(12B)	5517(9)	6154(16)	6188(15)	75(5)

Table S19. Anisotropic displacement parameters (Å^{2}x 10^3) for **11b**. The anisotropic displacement factor exponent takes the form: $-2p^2[h^2a^{*2}U^{11} + ... + 2 h k a^* b^* U^{12}]$.

	U^{11}	U^{22}	U^{33}	U^{23}	U^{13}	U^{12}
Ru(1)	16(1)	15(1)	14(1)	0(1)	4(1)	-1(1)
N(1)	19(1)	17(1)	14(1)	1(1)	3(1)	1(1)
F(1)	48(1)	26(1)	36(1)	-5(1)	9(1)	7(1)
P(1)	17(1)	16(1)	15(1)	1(1)	4(1)	2(1)
O(1)	33(1)	20(1)	22(1)	-4(1)	9(1)	2(1)
S(1)	20(1)	18(1)	21(1)	-1(1)	7(1)	3(1)
C(1)	16(1)	16(1)	18(1)	-1(1)	4(1)	0(1)
C(2)	20(1)	25(1)	16(1)	3(1)	6(1)	0(1)
N(2)	49(1)	19(1)	23(1)	0(1)	13(1)	1(1)
F(2)	57(1)	23(1)	49(1)	7(1)	14(1)	-3(1)
O(2)	19(1)	32(1)	31(1)	-3(1)	7(1)	2(1)
P(2)	16(1)	17(1)	17(1)	1(1)	3(1)	1(1)
O(3)	39(1)	16(1)	33(1)	-2(1)	11(1)	1(1)
F(3)	34(1)	32(1)	71(1)	-6(1)	4(1)	14(1)
C(3)	30(1)	23(1)	34(1)	1(1)	13(1)	0(1)
C(4)	36(1)	29(1)	44(2)	4(1)	14(1)	-6(1)

F(4)	82(1)	57(1)	27(1)	13(1)	-7(1)	-16(1)
O(4)	115(2)	34(1)	19(1)	-2(1)	12(1)	21(1)
C(5)	27(1)	45(2)	35(1)	5(1)	14(1)	-7(1)
F(5)	35(1)	68(1)	48(1)	-10(1)	-3(1)	1(1)
C(6)	27(1)	40(2)	29(1)	-3(1)	13(1)	2(1)
F(6)	59(1)	49(1)	30(1)	-10(1)	-2(1)	18(1)
C(7)	24(1)	26(1)	21(1)	0(1)	6(1)	2(1)
F(7)	73(1)	45(1)	56(1)	-8(1)	2(1)	32(1)
C(8)	22(1)	20(1)	19(1)	0(1)	3(1)	4(1)
F(8)	39(1)	69(1)	72(1)	-49(1)	0(1)	-2(1)
C(9)	25(1)	27(1)	24(1)	5(1)	5(1)	2(1)
F(9)	97(2)	43(1)	68(1)	-24(1)	55(1)	-18(1)
C(10)	35(1)	35(1)	27(1)	13(1)	3(1)	4(1)
C(11)	28(1)	39(2)	29(1)	9(1)	-4(1)	4(1)
C(12)	24(1)	34(1)	29(1)	3(1)	0(1)	-1(1)
C(14)	33(1)	20(1)	31(1)	6(1)	16(1)	10(1)
C(13)	24(1)	23(1)	20(1)	0(1)	3(1)	-1(1)
C(16)	71(2)	23(1)	63(2)	3(1)	43(2)	0(1)
C(15)	41(2)	23(1)	45(2)	2(1)	21(1)	3(1)
C(17)	109(3)	26(2)	63(2)	15(2)	56(2)	23(2)
C(18)	87(3)	44(2)	39(2)	18(2)	23(2)	41(2)
C(19)	49(2)	33(1)	33(1)	3(1)	12(1)	24(1)
C(20)	17(1)	20(1)	17(1)	-3(1)	5(1)	-2(1)
C(21)	27(1)	18(1)	18(1)	2(1)	4(1)	3(1)
C(22)	34(1)	23(1)	16(1)	2(1)	5(1)	-2(1)
C(23)	30(1)	17(1)	20(1)	-3(1)	9(1)	-2(1)
C(24)	24(1)	16(1)	22(1)	2(1)	7(1)	0(1)
C(25)	22(1)	19(1)	18(1)	2(1)	5(1)	-2(1)
C(26)	44(2)	31(1)	19(1)	-4(1)	11(1)	-9(1)
C(27)	33(1)	25(1)	14(1)	0(1)	11(1)	-6(1)
C(28)	28(1)	22(1)	24(1)	1(1)	17(1)	-2(1)
C(29)	18(1)	25(1)	25(1)	0(1)	10(1)	-2(1)
C(30)	22(1)	19(1)	22(1)	2(1)	10(1)	-5(1)
C(31)	25(1)	18(1)	21(1)	4(1)	10(1)	-1(1)
C(32)	27(1)	24(1)	15(1)	6(1)	6(1)	-3(1)
C(33)	24(1)	23(1)	25(1)	-3(1)	8(1)	-4(1)
C(34)	37(1)	29(1)	38(2)	0(1)	9(1)	-13(1)
C(35)	24(1)	33(1)	27(1)	-5(1)	4(1)	-4(1)
C(36)	19(1)	21(1)	21(1)	3(1)	6(1)	2(1)
C(37)	18(1)	23(1)	25(1)	3(1)	5(1)	1(1)
C(38)	22(1)	21(1)	32(1)	3(1)	9(1)	2(1)
C(39)	28(1)	24(1)	29(1)	8(1)	6(1)	7(1)

C(40)	27(1)	28(1)	25(1)	3(1)	4(1)	4(1)
C(41)	25(1)	22(1)	24(1)	2(1)	5(1)	4(1)
C(42)	30(1)	23(1)	39(2)	4(1)	8(1)	6(1)
C(43)	40(2)	34(1)	27(1)	5(1)	1(1)	9(1)
C(44)	17(1)	19(1)	18(1)	3(1)	0(1)	1(1)
C(45)	20(1)	26(1)	24(1)	-2(1)	6(1)	0(1)
C(47)	24(1)	23(1)	30(1)	0(1)	-1(1)	-6(1)
C(48)	22(1)	20(1)	24(1)	0(1)	-1(1)	1(1)
C(49)	18(1)	22(1)	22(1)	2(1)	1(1)	2(1)
C(51)	29(1)	27(1)	35(1)	-6(1)	3(1)	-3(1)
C(46)	21(1)	33(1)	27(1)	1(1)	4(1)	-5(1)
C(50A)	26(5)	49(5)	44(6)	-6(4)	20(4)	-15(4)
F(10A)	47(6)	95(8)	42(4)	-19(5)	25(3)	-39(6)
F(11A)	69(5)	52(3)	110(8)	-16(4)	56(5)	-35(3)
F(12A)	21(3)	120(8)	64(3)	4(4)	15(2)	1(4)
C(50B)	33(7)	42(6)	47(7)	-14(5)	1(5)	-18(5)
F(10B)	34(4)	41(4)	56(7)	-16(3)	24(4)	-9(3)
F(11B)	86(10)	44(3)	119(12)	9(5)	73(9)	-15(4)
F(12B)	27(5)	148(14)	49(4)	-24(6)	7(3)	-38(7)

4. Computational studies

4.1 General information

All calculations were performed without symmetry restrictions. Starting coordinates for **2a**, **3a**, **4b** and **11b** were obtained from the crystal structure analyses, for the other structures via GaussView 3.0.[5] The geometry optimization and transition state search were carried out with the Gaussian09 (Revision E.01) program package[6] using Density-Functional Theory (DFT)[7] with the PBE0-D3 functional[8] with Grimme's D3 dispersion correction with Becke-Johnson damping[9] and the LANL2TZ(f) basis set with the corresponding ECP for ruthenium[10] and the def2svp basis set[11a] for all other atoms. The metrical parameters of the energy-optimized geometry compared well with those determined by X-ray diffraction. Harmonic vibrational frequency analysis was performed on the same levels of theory to determine the nature of the structures.[12] The vibrational frequency analyses showed no imaginary frequencies for the ground states and one imaginary frequency for the transition states, corresponding to the expected translational motion of the transition states. Single point energies were calculated with the PBE0-D3 functional with Grimme's D3 dispersion correction with Becke-Johnson damping and the LANL2TZ(f) basis set and the corresponding ECP for Ruthenium and the def2tzvp basis[11b] set for all other atoms.

4.2 Energies and coordinates of the calculated structures and transition states

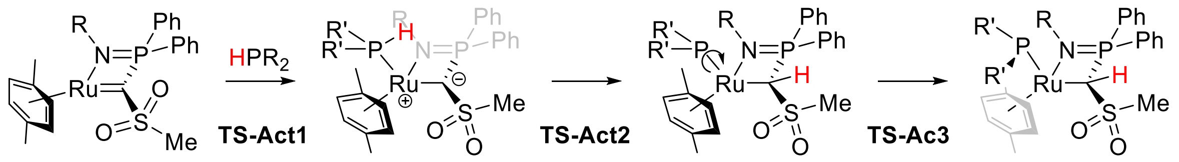

2a', R = $SiMe_3$
2b', R = p-C_6H_4-NO_2

Scheme S1. Calculated mechanism for the activation process of secondary phosphines with the carbene complexes **2a'** and **2b'**.

4.2.1 Starting compounds

Table S20. SCF energies, enthalpy and free energy corrections.

	E_{SCF}/E_H	corrH/E_H	corrG/E_H
**2a'**	-2298.926973	0.552078	0.448052
**2b'**	-2325.686655	0.534699	0.428786
**$HPPh_2$**	-804.756642	0.204686	0.153306
**$HPAr^{Cl}_2$**	-2642.632516	0.170923	0.106033
**$HPAr^{F}_2$**	-2152.281709	0.239707	0.144901

Table S21. Cartesian coordinates of **$HPPh_2$**

P -0.071903 1.712982 0.050053

C -1.437838 0.469018 0.057505

C -2.721502 0.924124 -0.269851

H -2.871167 1.977050 -0.526600
C -3.614929 -1.299552 0.022087
H -4.461663 -1.990078 0.007364
C -2.337320 -1.764991 0.332402
H -2.181192 -2.821807 0.563202
C -1.255246 -0.887452 0.352584
H -0.257478 -1.260110 0.597159
C 1.410002 0.634652 0.009979
C 1.834314 0.137713 -1.230872
H 1.274949 0.394442 -2.135225
C 2.957402 -0.679930 -1.322376
H 3.270941 -1.064961 -2.295779
C 3.687234 -0.997099 -0.176479
H 4.573174 -1.632431 -0.248892
C 3.283722 -0.497544 1.060331
H 3.851259 -0.741423 1.961794
C 2.149363 0.308164 1.153994
H 1.830917 0.683251 2.130195
C -3.805703 0.048187 -0.277500
H -4.802178 0.418869 -0.530197
H -0.046007 1.970730 1.455359

Table S22. Cartesian coordinates of **$HPAr^{Cl}_2$**
P -0.207974 -1.763660 -1.346744
C -1.495834 -0.648369 -0.625247
C -2.811184 -1.124002 -0.621593
H -3.050366 -2.114177 -1.015107
C -3.562521 0.929195 0.437339
H -4.362841 1.541256 0.852712
C -2.243585 1.379840 0.439585
C -1.209091 0.609326 -0.086409
H -0.189637 0.997555 -0.069965
C 1.325871 -0.994207 -0.695759
C 1.723610 -1.333062 0.603419
H 1.135708 -2.027859 1.206778
C 2.882924 -0.775108 1.135717
C 3.673960 0.100711 0.392957
H 4.584104 0.525867 0.815471
C 3.272870 0.416831 -0.902599
C 2.106587 -0.114897 -1.452894
H 1.814960 0.169539 -2.465139

C -3.829794 -0.327085 -0.101511
H -0.155061 -1.159507 -2.639175
Cl -5.457369 -0.913487 -0.107282
Cl -1.888284 2.935569 1.108835
Cl 4.236974 1.502081 -1.842722
Cl 3.363964 -1.182563 2.745731

Table S23. Cartesian coordinates of **HPAr$^{F}_{2}$**
P -0.247917 -0.669235 -2.381965
C -1.520095 -0.157091 -1.140818
C -2.843316 -0.532664 -1.385851
H -3.091110 -1.112053 -2.279197
C -3.562640 0.560435 0.647486
H -4.358204 0.852497 1.334467
C -2.240054 0.919404 0.904570
C -1.225577 0.568760 0.018250
H -0.199667 0.873675 0.233543
C 1.286913 -0.479414 -1.394803
C 1.638429 -1.512075 -0.515630
H 1.005454 -2.398780 -0.428734
C 2.794003 -1.420738 0.254134
C 3.636063 -0.315262 0.137543
H 4.547806 -0.250322 0.732904
C 3.301577 0.703257 -0.748580
C 2.129961 0.629606 -1.503644
H 1.872841 1.453594 -2.172965
C -3.858093 -0.167511 -0.500220
H -0.152877 0.578674 -3.068434
C -5.270971 -0.613394 -0.771862
C -1.908987 1.659977 2.173905
C 3.122006 -2.501322 1.251540
C 4.223539 1.880883 -0.929013
F -5.553921 -0.563270 -2.075713
F -6.159798 0.146482 -0.130717
F -5.463328 -1.874811 -0.375270
F -2.875763 2.519399 2.502545
F -1.763918 0.816548 3.199852
F -0.771143 2.347643 2.060376
F 2.604980 -3.676521 0.889677
F 2.631919 -2.204996 2.458533
F 4.440192 -2.656921 1.386303

F 3.537231 3.006236 -1.142010
F 4.997809 2.069154 0.139803
F 5.028850 1.704849 -1.980505

Table S24. Cartesian coordinates of **2a’**
Ru 1.695172 -0.097603 0.173681
N 0.017619 1.259238 0.347181
O 0.973929 -3.557857 -0.750858
P -1.034649 0.045141 0.092005
S -0.185066 -2.884076 -0.136748
C 0.143180 -1.221598 0.096516
C -2.298209 -0.104388 1.381353
O -1.505279 -3.041493 -0.775321
C -2.249914 0.729486 2.501569
H -1.476523 1.499620 2.555603
Si -0.061841 2.953308 -0.038911
C -4.135151 -0.444867 3.448464
H -4.859180 -0.575614 4.256467
C -4.171574 -1.290227 2.337730
H -4.919191 -2.084879 2.278848
C -3.255481 -1.126499 1.301634
H -3.258986 -1.799975 0.439065
C -1.935774 0.179606 -1.478052
C -3.242853 0.671370 -1.548703
H -3.783286 0.923316 -0.633190
C -3.859338 0.837237 -2.788008
H -4.881440 1.219746 -2.838818
C -3.174581 0.511877 -3.957305
H -3.660672 0.638485 -4.927695
C -1.872983 0.012275 -3.888984
H -1.341224 -0.258691 -4.803970
C -1.255276 -0.158027 -2.654537
H -0.244741 -0.568462 -2.586474
C -1.836994 3.581950 0.012757
H -2.336002 3.333230 0.962269
H -2.434335 3.157365 -0.808126
H -1.849743 4.678443 -0.097382
C 0.634506 3.281540 -1.755928
H 0.565317 4.347471 -2.025977
H 0.075343 2.698980 -2.505507
H 1.691658 2.981892 -1.817915

C 0.942763 3.898234 1.240889
H 0.886981 4.984243 1.064494
H 2.002813 3.606661 1.211993
H 0.567248 3.695441 2.256217
C 2.951758 -1.503493 -2.470376
H 2.292669 -2.369474 -2.314263
H 3.902335 -1.852390 -2.906478
H 2.480572 -0.816840 -3.188264
C 3.200607 -0.816298 -1.164723
C 3.269600 -1.564715 0.043902
H 3.072801 -2.637330 0.012127
C 3.336550 -0.884178 1.286254
H 3.248618 -1.454219 2.214004
C 3.483580 0.540787 1.363360
C 3.541117 1.260654 0.147876
H 3.590576 2.350581 0.172414
C 3.354335 0.607753 -1.090278
H 3.276046 1.196986 -2.005590
C -3.173457 0.561199 3.532138
H -3.140366 1.216716 4.405661
C -0.337067 -3.578147 1.504658
H -0.561021 -4.644940 1.366293
H 0.613437 -3.441722 2.034533
H -1.157984 -3.070418 2.027594
C 3.565768 1.224784 2.691814
H 4.607819 1.248038 3.051869
H 3.204179 2.260020 2.632601
H 2.962062 0.696286 3.443326

Table S25. Cartesian coordinates of **2b'**

Ru 1.026494 -1.594548 -0.167166
C 2.165873 -3.136121 -2.852804
H 1.285561 -2.906988 -3.469021
H 2.571315 -4.107227 -3.181987
H 2.929681 -2.369403 -3.040619
S 3.370057 0.801467 0.060891
P 0.340442 1.087788 -0.029345
O 3.270935 2.115389 0.723793
O 4.244244 -0.226452 0.646578
O -6.677253 -1.882296 -0.406269
O -6.891296 0.198944 0.092806

N -0.625216 -0.236868 -0.264979
N -6.221243 -0.781739 -0.164547
C -0.088312 4.350786 -3.224363
H -0.168671 5.121205 -3.995027
C -1.985136 -0.334766 -0.219503
C -2.836837 0.759278 0.077005
H -2.407023 1.734370 0.306683
C -4.212348 0.613841 0.095666
H -4.869620 1.451626 0.329493
C -4.778945 -0.629020 -0.185271
C -3.972683 -1.726316 -0.492174
H -4.448671 -2.680365 -0.720344
C -2.598961 -1.577807 -0.508832
H -1.955342 -2.416646 -0.769309
C 0.126671 1.850877 1.599500
C 0.682449 1.194833 2.704494
H 1.278474 0.292862 2.545731
C 0.496462 1.709612 3.982776
H 0.937351 1.200084 4.842439
C -0.236240 2.883213 4.164533
H -0.376122 3.288966 5.169259
C -0.779819 3.545546 3.065444
H -1.345677 4.469258 3.205390
C -0.598961 3.032397 1.782910
H -1.015729 3.561967 0.922324
C -0.896100 3.216439 -3.289513
H -1.608228 3.095885 -4.108982
C -0.797328 2.231070 -2.308711
H -1.421322 1.336130 -2.358744
C 0.106087 2.394644 -1.253773
C 0.929036 3.529688 -1.191403
H 1.658762 3.628031 -0.381618
C 0.826271 4.503950 -2.180650
H 1.465385 5.388905 -2.137061
C 0.514383 -3.633372 -0.984492
H -0.227695 -3.893941 -1.741580
C 1.807622 -3.192456 -1.400861
C 2.763455 -2.844633 -0.397888
H 3.746972 -2.464156 -0.676551
C 2.381154 -2.793359 0.961168
H 3.083280 -2.372022 1.682568

C 1.053948 -3.151109 1.369024
C 0.156411 -3.629457 0.378385
H -0.868459 -3.867800 0.668442
C 1.794778 0.163092 -0.173912
C 0.634060 -3.033584 2.800631
H -0.438803 -2.810307 2.881094
H 0.830032 -3.977109 3.336437
H 1.192750 -2.236332 3.309975
C 4.000139 1.117891 -1.581538
H 5.010568 1.527959 -1.446470
H 4.032092 0.173220 -2.138026
H 3.345239 1.846280 -2.076950

4.2.2 2a' + $HPPh_2$

Table S26. SCF energies, enthalpy and free energy corrections and barriers

	E_{SCF}/E_H	corrH/E_H	corrG/E_H	ΔH/kJ/mol	ΔG/kJ/mol
**TS-Act1**	-3103.696658	0.758320	0.628619	-30.159318	37.329159
**Coord**	-3103.725935	0.759640	0.632364	-103.560684	-29.705369
**TS-Act2**	-3103.697813	0.756438	0.628842	-38.134878	34.880277
**Act'**	-3103.737086	0.761603	0.634635	-127.685563	-53.021594
**TS-Act3**	-3103.736387	0.760512	0.636187	-128.713683	-47.110517
**Act**	-3103.743736	0.761494	0.633592	-145.430504	-73.218752
**TS1**	-3103.723781	0.760470	0.630914	-95.726323	-27.857148
**Int1**	-3103.723873	0.761441	0.628809	-93.418220	-33.625083
**TS4**	-3103.710593	0.760626	0.632082	-60.691993	9.834188
**Int3**	-3103.722072	0.760607	0.634565	-90.879808	-13.784626
**TS5**	-3103.712618	0.755929	0.630350	-78.341286	-0.030498
**Int4**	-3103.717445	0.758103	0.630506	-85.307184	-12.294655
**TS6**	-3103.709331	0.756925	0.629399	-67.095745	6.103195
**3'**	-3103.761485	0.759671	0.633636	-196.817079	-119.703518
**TS2**	-3103.714890	0.760169	0.635268	-73.172990	6.917888
**Int2**	-3103.730592	0.761156	0.634267	-111.807616	-36.936232
**TS3**	-3103.718155	0.760014	0.635914	-82.153486	0.040417
**4'**	-3103.754871	0.761117	0.631269	-175.656100	-108.553571

Table S27. Cartesian coordinates of **TS-Act1**

Ru 0.223073 1.397786 -0.843610
N 1.016306 -0.605611 -1.105146
O 0.529508 2.909681 2.439175
P 1.583445 -0.652211 0.417127
S 1.034192 1.516985 2.474802

C 0.808270 0.788767 0.960398
C 1.106027 -2.160882 1.312476
O 2.398195 1.296198 2.997801
C 0.119002 -3.006255 0.797235
H -0.301553 -2.799023 -0.189823
Si 1.413477 -1.429525 -2.578444
C 0.205749 -4.338573 2.809115
H -0.143724 -5.192608 3.394428
C 1.188103 -3.493526 3.329344
H 1.605312 -3.683915 4.321087
C 1.640403 -2.406356 2.585342
H 2.404320 -1.735672 2.991115
C 3.402620 -0.655763 0.488395
C 4.127665 -1.850743 0.412022
H 3.604625 -2.810062 0.423518
C 5.518312 -1.821014 0.330291
H 6.079513 -2.756661 0.271090
C 6.190643 -0.599172 0.331051
H 7.281532 -0.576585 0.270453
C 5.471998 0.592377 0.429254
H 5.998290 1.549396 0.458593
C 4.082694 0.565592 0.512522
H 3.510974 1.484687 0.635642
C 2.136088 -3.143135 -2.271298
H 1.528250 -3.740807 -1.575528
H 3.157361 -3.078153 -1.867598
H 2.191434 -3.690453 -3.226513
C 2.689050 -0.471667 -3.583625
H 3.049643 -1.072511 -4.434156
H 3.555232 -0.227874 -2.947357
H 2.288450 0.469418 -3.988673
C -0.175724 -1.623096 -3.565883
H 0.013867 -2.039945 -4.567710
H -0.679563 -0.652660 -3.683509
H -0.873227 -2.292997 -3.038671
C 2.364120 3.819347 -0.322032
H 2.247118 3.702235 0.764278
H 2.501581 4.890802 -0.541642
H 3.266420 3.288023 -0.653346
C 1.149079 3.310418 -1.027962
C -0.149233 3.496991 -0.464991

H -0.235856 3.912291 0.539933
C -1.277254 2.941573 -1.105258
H -2.244517 2.981350 -0.600430
C -1.190359 2.286617 -2.377252
C 0.084683 2.169460 -2.966472
H 0.198333 1.607760 -3.895302
C 1.240589 2.628481 -2.282358
H 2.229218 2.412637 -2.691192
C -0.328355 -4.094700 1.544294
H -1.100611 -4.750130 1.134690
C -0.030523 0.656046 3.633159
H 0.158333 1.118196 4.611811
H -1.080137 0.780373 3.341509
H 0.248218 -0.405113 3.649546
C -2.425532 1.760458 -3.035593
H -2.923452 2.561272 -3.606909
H -2.196149 0.942551 -3.732525
H -3.142242 1.393424 -2.286957
P -2.137173 -0.254685 0.057535
H -1.499190 -0.802463 1.199247
C -3.171887 -1.708624 -0.379931
C -3.524095 -2.682632 0.565548
C -3.575149 -1.880327 -1.709880
C -4.265773 -3.799215 0.187516
H -3.211456 -2.562719 1.606153
C -4.326817 -2.993031 -2.086968
H -3.286828 -1.138747 -2.459163
C -4.670420 -3.955230 -1.139282
H -4.533139 -4.551965 0.933505
H -4.635989 -3.113191 -3.128052
H -5.252326 -4.831659 -1.434522
C -3.341530 0.842045 0.914281
C -4.726950 0.691053 0.781616
C -2.844615 1.931140 1.650717
C -5.597907 1.602687 1.379330
H -5.130667 -0.151105 0.214211
C -3.718966 2.829027 2.257593
H -1.766488 2.094026 1.743540
C -5.098975 2.670445 2.122096
H -6.676979 1.469995 1.267161
H -3.311728 3.662289 2.835611

H -5.783184 3.379527 2.594100

Table S28. Cartesian coordinates of **Coord**

Ru 0.488478 0.879903 -0.931400
N -0.841958 1.046800 0.804511
O 0.052300 -2.239027 -2.858854
P -1.629886 -0.357460 0.472233
S -0.770491 -2.312771 -1.626963
C -0.502388 -0.962189 -0.660058
C -1.876499 -1.423235 1.927715
O -2.223961 -2.559327 -1.787851
C -1.232132 -1.164129 3.140071
H -0.651660 -0.245051 3.247404
Si -1.462126 2.520188 1.499845
C -2.079621 -3.238359 4.040492
H -2.161760 -3.947363 4.867929
C -2.718815 -3.504472 2.828005
H -3.298036 -4.422810 2.704589
C -2.622118 -2.601115 1.772489
H -3.109688 -2.808528 0.814433
C -3.338261 -0.039256 -0.089664
C -4.392108 0.115752 0.817027
H -4.237430 -0.097251 1.877590
C -5.645153 0.531902 0.370200
H -6.464174 0.653798 1.083139
C -5.853109 0.784454 -0.985337
H -6.835908 1.109478 -1.335609
C -4.812245 0.599139 -1.896362
H -4.982215 0.764316 -2.963223
C -3.561352 0.183718 -1.450427
H -2.749034 -0.010501 -2.148229
C -2.422656 2.198464 3.091150
H -1.800522 1.707736 3.854693
H -3.315466 1.578196 2.930413
H -2.760461 3.163213 3.504311
C -2.619002 3.463478 0.344508
H -3.085273 4.302657 0.886267
H -3.424017 2.810186 -0.025925
H -2.097369 3.884942 -0.527204
C -0.011410 3.633128 1.961709
H -0.322394 4.689713 1.986931

H 0.820058 3.530329 1.250970
H 0.372776 3.378627 2.961926
C -1.127436 0.554422 -4.058502
H -1.168372 -0.519588 -3.822938
H -0.782369 0.646486 -5.100818
H -2.128775 0.998088 -3.991082
C -0.157322 1.239169 -3.155022
C 1.161665 0.699182 -2.991539
H 1.392867 -0.247376 -3.481094
C 2.141619 1.385029 -2.230194
H 3.151056 0.972170 -2.156613
C 1.837557 2.604965 -1.545563
C 0.487373 3.010557 -1.572398
H 0.187242 3.873792 -0.975721
C -0.489465 2.371278 -2.405461
H -1.514532 2.744795 -2.418910
C -1.337480 -2.068332 4.196108
H -0.839321 -1.857745 5.145723
C -0.210787 -3.787566 -0.772613
H -0.492504 -4.631994 -1.415842
H 0.872959 -3.754030 -0.624794
H -0.734859 -3.846806 0.189908
C 2.893728 3.386685 -0.832649
H 3.414827 4.048139 -1.544522
H 2.463171 4.018632 -0.044315
H 3.643925 2.729071 -0.373292
P 1.735718 -0.321020 0.507756
H 1.054398 -0.712767 1.680417
C 3.145603 0.596203 1.254873
C 2.871172 1.531818 2.258786
C 4.456730 0.463724 0.784344
C 3.887382 2.327880 2.779767
H 1.846803 1.643565 2.621369
C 5.474140 1.264576 1.304225
H 4.686008 -0.276253 0.012822
C 5.191689 2.198799 2.299736
H 3.660348 3.055634 3.562744
H 6.495282 1.154067 0.931073
H 5.989693 2.824710 2.706083
C 2.553364 -1.861330 -0.007402
C 2.999296 -2.759411 0.971278

C 2.771589 -2.147115 -1.356516
C 3.645180 -3.934868 0.599915
H 2.830482 -2.541373 2.029444
C 3.425249 -3.322039 -1.725861
H 2.374294 -1.480997 -2.119758
C 3.861350 -4.216353 -0.750732
H 3.978292 -4.637923 1.366998
H 3.570008 -3.546999 -2.784621
H 4.363755 -5.141900 -1.041843

Table S29. Cartesian coordinates of **TS-Act2**
Ru -0.432101 1.060405 -0.860023
N 0.825168 -0.732629 -0.917107
O -0.065133 3.064993 2.208972
P 1.631086 -0.378919 0.461424
S 0.779133 1.855233 2.349212
C 0.519031 0.813643 1.030925
C 1.759255 -1.795804 1.591656
O 2.216078 2.056197 2.626450
C 0.933175 -2.906669 1.400044
H 0.298307 -2.950876 0.512314
Si 1.429995 -1.557455 -2.333284
C 1.714899 -3.845167 3.483348
H 1.700510 -4.649137 4.223347
C 2.531260 -2.730299 3.683231
H 3.152330 -2.659935 4.579468
C 2.556277 -1.704467 2.740914
H 3.183071 -0.821539 2.898857
C 3.360141 0.116076 0.181778
C 4.376635 -0.839686 0.068002
H 4.167220 -1.890590 0.280493
C 5.661104 -0.453451 -0.308347
H 6.449827 -1.204033 -0.398820
C 5.938960 0.889487 -0.562605
H 6.946977 1.191973 -0.856715
C 4.935575 1.848067 -0.419868
H 5.158609 2.904586 -0.587326
C 3.651822 1.463279 -0.044513
H 2.869482 2.202488 0.112947
C 2.273094 -3.179584 -1.870991
H 1.570205 -3.866594 -1.375951

H 3.136844 -3.042490 -1.205534
H 2.636864 -3.672425 -2.787430
C 2.672128 -0.508608 -3.292694
H 3.132211 -1.105629 -4.096940
H 3.477710 -0.142546 -2.638019
H 2.195518 0.364916 -3.762500
C 0.015394 -2.008832 -3.483827
H 0.420927 -2.368208 -4.443337
H -0.646171 -1.156144 -3.689357
H -0.596270 -2.810865 -3.046630
C 1.329471 4.094422 -0.605797
H 1.344871 3.930977 0.480954
H 1.049730 5.147292 -0.771498
H 2.331152 3.941355 -1.026757
C 0.313836 3.214218 -1.256948
C -1.015868 3.150290 -0.723482
H -1.232065 3.658063 0.216635
C -2.026128 2.422671 -1.389752
H -3.030728 2.398378 -0.962375
C -1.754178 1.692368 -2.591090
C -0.410541 1.641694 -3.011959
H -0.144302 1.020474 -3.869173
C 0.610177 2.420351 -2.373767
H 1.636328 2.372796 -2.742084
C 0.916161 -3.932093 2.343404
H 0.272244 -4.801194 2.187127
C 0.142512 1.007410 3.792933
H 0.327967 1.671794 4.647629
H -0.932417 0.829099 3.661231
H 0.683837 0.059750 3.911759
C -2.860090 1.024536 -3.345288
H -3.337440 1.746954 -4.028042
H -2.489956 0.189011 -3.955539
H -3.636824 0.650281 -2.663717
P -1.851737 -0.352168 0.337632
H -0.645296 -0.191556 1.359539
C -2.446601 -1.973652 -0.242005
C -2.561457 -3.013090 0.693449
C -2.783328 -2.223792 -1.575641
C -3.005385 -4.273146 0.299862
H -2.292218 -2.826464 1.736546

C -3.225765 -3.485661 -1.970429
H -2.686899 -1.423096 -2.307815
C -3.336573 -4.512705 -1.034457
H -3.090363 -5.074398 1.038241
H -3.481831 -3.667615 -3.017152
H -3.680699 -5.502291 -1.344588
C -3.333674 0.463283 1.013975
C -4.625632 -0.056500 0.861932
C -3.160912 1.664907 1.724761
C -5.723138 0.609929 1.407959
H -4.775159 -0.989668 0.313471
C -4.260407 2.320058 2.270884
H -2.160891 2.095090 1.847832
C -5.546225 1.797963 2.113682
H -6.725459 0.193707 1.278067
H -4.108164 3.250041 2.824473
H -6.407223 2.317599 2.540803

Table S30. Cartesian coordinates of **Act'**
Ru -0.460787 -0.856751 -0.795210
Si 0.780423 2.583328 -1.401466
P 1.741925 0.385592 0.424582
O 2.488828 -1.900225 2.724988
S 1.627707 -2.364072 1.624798
N 0.735033 0.977264 -0.689608
H 0.175825 -0.617077 1.888052
C 0.725607 -0.979793 1.002554
C 2.132377 1.563423 1.751497
O 2.247727 -3.153468 0.550266
C 1.046778 2.039303 2.501555
H 0.034882 1.670819 2.288711
C 1.254324 2.989074 3.496675
H 0.405837 3.355623 4.079043
C 3.617342 3.010829 2.993970
H 4.622764 3.394122 3.182554
C 3.417568 2.057426 1.997205
H 4.264714 1.704079 1.406274
C 3.325142 -0.168924 -0.270852
C 5.546252 -1.095768 -0.079108
H 6.336273 -1.535489 0.533760
C 4.344189 -0.724621 0.515569

H 4.183739 -0.893219 1.582376
C 5.740890 -0.916834 -1.449220
H 6.688620 -1.208248 -1.908555
C -0.157667 3.876110 -0.416383
H -1.219904 3.640242 -0.281298
H -0.082670 4.848989 -0.929269
H 0.298654 3.986038 0.579781
C 2.552708 3.238400 -1.477291
H 3.016141 3.277651 -0.479349
H 2.517605 4.272639 -1.858060
H 3.214870 2.660386 -2.137250
C 3.516666 -0.007925 -1.644419
H 2.700550 0.400787 -2.243126
C 4.724513 -0.374817 -2.232803
H 4.869410 -0.243094 -3.307758
C 0.151205 2.477371 -3.170766
H -0.873796 2.087831 -3.224119
H 0.802261 1.829197 -3.778449
H 0.153514 3.479217 -3.628881
C 1.982262 -2.763249 -2.535692
H 2.542066 -2.883204 -1.598553
H 2.511923 -2.059732 -3.189301
H 1.958401 -3.741115 -3.044687
C -1.614848 -2.691072 -1.156635
H -2.219343 -3.232527 -0.427692
C -2.227063 -1.686166 -1.958745
C -1.404416 -0.939267 -2.836744
H -1.837977 -0.148412 -3.449400
C -0.020342 -1.243063 -2.960895
H 0.592438 -0.632032 -3.627732
C 0.583447 -2.310675 -2.268201
C -0.229834 -2.958153 -1.287577
H 0.246810 -3.703478 -0.649013
C 2.538814 3.474748 3.744759
H 2.699151 4.221992 4.525784
C -3.707556 -1.484940 -1.927499
H -4.150287 -2.018337 -2.784761
H -4.151061 -1.885549 -1.008809
H -3.982557 -0.425237 -2.007980
C 0.394983 -3.410077 2.377654
H -0.335535 -3.734017 1.627472

H 0.964279 -4.267256 2.761162
H -0.083820 -2.869991 3.203158
P -1.895447 0.314285 0.769400
C -3.284640 -0.781372 1.275092
C -2.961142 -1.995834 1.905879
C -4.647128 -0.466053 1.147277
C -3.942902 -2.867473 2.364119
H -1.909621 -2.254779 2.029481
C -5.634380 -1.334414 1.613444
H -4.944145 0.466136 0.662765
C -5.293350 -2.542411 2.218362
H -3.653553 -3.805466 2.846236
H -6.686527 -1.062340 1.493151
H -6.069347 -3.221791 2.578379
C -2.845976 1.686739 -0.004454
C -3.456726 2.611663 0.859954
C -2.930944 1.924258 -1.378920
C -4.138125 3.719445 0.362922
H -3.381077 2.458498 1.940513
C -3.597926 3.040745 -1.883334
H -2.436841 1.229054 -2.055234
C -4.207545 3.941907 -1.013808
H -4.604773 4.424802 1.055276
H -3.634562 3.209573 -2.962853
H -4.728116 4.819617 -1.404137

Table S31. Cartesian coordinates of **TS-Act3**
Ru -0.454052 -0.693087 -0.921695
Si 0.739642 2.800080 -1.034483
P 1.754400 0.369814 0.443569
O 2.473269 -2.213580 2.438205
S 1.609515 -2.515168 1.284527
N 0.749353 1.105232 -0.580928
H 0.193422 -0.790480 1.781023
C 0.724980 -1.048527 0.849522
C 2.156379 1.355362 1.915690
O 2.230294 -3.151108 0.112797
C 1.069978 1.768467 2.701045
H 0.050943 1.472903 2.418849
C 1.287636 2.565441 3.820585
H 0.438781 2.884445 4.429827

C 3.662355 2.557432 3.374276
H 4.676289 2.869951 3.634343
C 3.452747 1.757199 2.252713
H 4.300296 1.453518 1.635504
C 3.331887 -0.089773 -0.329717
C 5.537036 -1.071463 -0.294138
H 6.315146 -1.626323 0.234816
C 4.335896 -0.792385 0.351036
H 4.166059 -1.142303 1.371511
C 5.746113 -0.653931 -1.609048
H 6.693084 -0.874602 -2.107712
C -0.339532 3.863331 0.073109
H -1.370573 3.497752 0.155767
H -0.369843 4.894335 -0.315920
H 0.094956 3.897584 1.084277
C 2.472795 3.539476 -0.896738
H 2.852240 3.490369 0.135434
H 2.416332 4.605588 -1.172591
H 3.212256 3.061094 -1.555092
C 3.538086 0.313001 -1.651050
H 2.733572 0.836031 -2.171925
C 4.745477 0.037637 -2.288501
H 4.902047 0.358229 -3.321239
C 0.222022 2.934315 -2.839729
H -0.771865 2.506269 -3.028113
H 0.947046 2.418611 -3.489440
H 0.192998 3.992466 -3.144952
C 2.006224 -2.360313 -2.868061
H 2.553832 -2.619373 -1.952624
H 2.543660 -1.566767 -3.401392
H 1.989079 -3.250689 -3.518393
C -1.605170 -2.464313 -1.516227
H -2.214262 -3.090585 -0.861975
C -2.208424 -1.370504 -2.200512
C -1.377516 -0.519577 -2.968439
H -1.809892 0.339883 -3.481108
C 0.008093 -0.801254 -3.111626
H 0.628391 -0.109214 -3.685896
C 0.603935 -1.950077 -2.554358
C -0.219174 -2.716375 -1.672433
H 0.251521 -3.536907 -1.129215

C 2.582812 2.959755 4.158666
H 2.751051 3.587461 5.037182
C -3.688498 -1.175608 -2.176076
H -4.111994 -1.612860 -3.095245
H -4.144204 -1.681582 -1.317400
H -3.963694 -0.113221 -2.145796
C 0.366738 -3.650565 1.874335
H -0.451977 -3.731252 1.149561
H 0.901018 -4.604307 1.977750
H 0.004575 -3.307777 2.849859
P -1.901154 0.298839 0.760305
C -3.101171 -0.972037 1.346260
C -2.610362 -1.953076 2.224469
C -4.470547 -1.016927 1.037806
C -3.422287 -2.966494 2.722616
H -1.567737 -1.896633 2.539147
C -5.293869 -2.020689 1.548345
H -4.903635 -0.250249 0.392741
C -4.774931 -3.010943 2.380418
H -3.002774 -3.715344 3.400255
H -6.355908 -2.027359 1.288292
H -5.421283 -3.797971 2.775745
C -3.058910 1.550757 0.074324
C -3.978098 2.158855 0.949270
C -2.978443 2.051549 -1.229312
C -4.794510 3.201949 0.523658
H -4.044560 1.808741 1.983375
C -3.783499 3.107037 -1.657523
H -2.249304 1.608739 -1.905803
C -4.701384 3.684209 -0.784175
H -5.502849 3.653254 1.223308
H -3.687371 3.481019 -2.680417
H -5.335705 4.510375 -1.113892

Table S32. Cartesian coordinates of **Act**
Ru 0.624498 -1.156857 -0.285822
Si -0.868858 -0.611123 2.984006
P -1.773983 0.225733 0.205033
O -1.952310 -0.719696 -3.046845
S -1.018629 0.383277 -2.792092
N -0.894792 -0.649502 1.230964

H -0.047600 1.406988 -0.984182

C -0.571263 0.450534 -1.097705

C -2.364827 1.824536 0.844932

O 0.209516 0.451607 -3.600255

C -1.537610 2.952049 0.763861

H -0.571726 2.902667 0.256646

C -1.923681 4.148255 1.361672

H -1.266240 5.017982 1.293830

C -3.962709 3.109505 2.134514

H -4.911276 3.168846 2.673109

C -3.582304 1.912089 1.533559

H -4.234920 1.038477 1.602916

C -3.279962 -0.633718 -0.326149

C -5.420948 -0.713987 -1.442898

H -6.212292 -0.215201 -2.006869

C -4.297559 0.002349 -1.045342

H -4.224852 1.065769 -1.284280

C -5.537072 -2.067198 -1.121289

H -6.420037 -2.628052 -1.436899

C -0.617419 1.107401 3.702115

H 0.308483 1.535195 3.288008

H -0.518055 1.042418 4.798234

H -1.451321 1.788547 3.477574

C -2.516941 -1.283048 3.617415

H -3.361634 -0.678493 3.250279

H -2.549574 -1.263727 4.718932

H -2.687417 -2.321567 3.291953

C -3.408334 -1.981330 0.009337

H -2.614611 -2.446785 0.592700

C -4.533810 -2.698912 -0.389690

H -4.629143 -3.754682 -0.124897

C 0.518990 -1.709130 3.605326

H 1.478537 -1.319348 3.237742

H 0.418211 -2.755804 3.286055

H 0.538748 -1.689268 4.706539

C 2.938674 -1.285099 -2.774002

H 2.368030 -0.636281 -3.451840

H 3.476705 -2.032711 -3.380517

H 3.687260 -0.685201 -2.238362

C 1.588691 -3.055658 0.372363

H 1.946393 -3.319421 1.368220

C 0.257757 -3.389580 -0.021948

C -0.186593 -3.015762 -1.309401

H -1.209791 -3.218889 -1.628245

C 0.672639 -2.271383 -2.161012

H 0.288115 -1.921687 -3.118729

C 2.017504 -1.967856 -1.814323

C 2.441997 -2.363588 -0.517131

H 3.445270 -2.096001 -0.184408

C -3.134075 4.228327 2.049414

H -3.433357 5.166524 2.522725

C -0.647581 -4.105791 0.932444

H -1.518212 -4.528149 0.414058

H -0.113173 -4.924701 1.435667

H -1.008275 -3.415547 1.708794

C -1.867813 1.921371 -3.123737

H -1.174263 2.747238 -2.919938

H -2.133364 1.891964 -4.188758

H -2.767930 1.996969 -2.503038

P 1.899008 0.577147 0.840063

C 2.016359 2.125595 -0.170148

C 2.066622 3.343473 0.525506

C 2.023006 2.166044 -1.572701

C 2.119060 4.560808 -0.154114

H 2.055721 3.325967 1.619293

C 2.059690 3.381274 -2.256120

H 1.955780 1.234662 -2.137235

C 2.108746 4.583123 -1.548833

H 2.162222 5.496979 0.408947

H 2.046399 3.381582 -3.349154

H 2.138252 5.535918 -2.083539

C 3.685701 0.128365 0.863604

C 4.660218 0.656273 0.002077

C 4.113422 -0.787908 1.842825

C 5.995522 0.263253 0.097120

H 4.371490 1.387502 -0.755847

C 5.442874 -1.188972 1.932465

H 3.382763 -1.195474 2.546861

C 6.395080 -0.666092 1.055406

H 6.731121 0.690469 -0.589779

H 5.740949 -1.906643 2.701402

H 7.440982 -0.973661 1.126685

Table S33. Cartesian coordinates of **TS1**

Ru 0.877488 -1.377603 -0.332252
Si -0.742093 0.699031 3.234270
P -1.839711 0.150447 0.337255
O -1.764928 -1.249832 -3.034526
S -0.941533 -0.068921 -2.718376
N -1.083212 0.057312 1.694452
H -0.140128 1.052528 -0.935219
C -0.592562 0.050948 -0.992640
C -2.695812 1.744453 0.047067
O 0.329535 0.068161 -3.449777
C -1.903473 2.901844 -0.002390
H -0.820317 2.835297 0.132439
C -2.485944 4.145807 -0.219537
H -1.853475 5.035828 -0.259560
C -4.663789 3.110000 -0.318579
H -5.747231 3.189328 -0.436556
C -4.081771 1.860152 -0.102306
H -4.710979 0.968514 -0.053118
C -3.165117 -1.104979 0.244386
C -4.886751 -2.411053 -0.843461
H -5.394279 -2.722797 -1.759495
C -3.836025 -1.497824 -0.919968
H -3.513163 -1.134847 -1.895138
C -5.285584 -2.928827 0.388416
H -6.110907 -3.643302 0.441195
C -0.804993 2.584111 3.285885
H -0.076584 3.027428 2.589674
H -0.578385 2.955192 4.298810
H -1.803043 2.954165 3.002835
C -1.983146 0.060901 4.511615
H -3.008926 0.374843 4.258523
H -1.753713 0.443732 5.519456
H -1.969258 -1.040362 4.563659
C -3.571313 -1.626537 1.478717
H -3.035828 -1.312800 2.376740
C -4.626998 -2.532656 1.551590
H -4.933709 -2.931680 2.521532
C 0.970366 0.130981 3.777788
H 1.740837 0.501226 3.084029

H 1.029474 -0.969599 3.770086
H 1.209848 0.480040 4.795073
C 2.673154 -2.377917 -2.869606
H 2.109341 -1.688483 -3.513569
H 2.927867 -3.281284 -3.448191
H 3.607095 -1.886146 -2.564617
C 1.605865 -3.141399 0.743655
H 2.052255 -3.155716 1.740325
C 0.184295 -3.302337 0.624366
C -0.372758 -3.222375 -0.668531
H -1.455582 -3.269216 -0.795162
C 0.438920 -2.921657 -1.796309
H -0.046111 -2.746550 -2.756380
C 1.847811 -2.738115 -1.675922
C 2.428108 -2.917855 -0.379655
H 3.497657 -2.747365 -0.242075
C -3.868510 4.252300 -0.382754
H -4.326334 5.229306 -0.555438
C -0.650732 -3.518660 1.845056
H -1.717439 -3.548644 1.592323
H -0.378747 -4.468157 2.333478
H -0.498820 -2.698770 2.562004
C -1.908088 1.364034 -3.169856
H -1.346851 2.269948 -2.908091
H -2.042853 1.285862 -4.256781
H -2.874343 1.347059 -2.650837
P 2.169430 0.355623 0.155956
C 1.943391 2.085778 -0.377860
C 2.013257 3.104223 0.585849
C 1.643828 2.426530 -1.706111
C 1.773022 4.430918 0.235173
H 2.247014 2.847916 1.622506
C 1.394472 3.755497 -2.050561
H 1.573123 1.639612 -2.462476
C 1.454534 4.758655 -1.083650
H 1.823867 5.211025 0.998646
H 1.155506 4.006372 -3.087346
H 1.258089 5.798209 -1.357579
C 3.951313 0.242393 0.468104
C 4.890603 1.060953 -0.179463
C 4.412279 -0.706742 1.396785

C 6.251059 0.916532 0.080487
H 4.548485 1.812160 -0.895427
C 5.773034 -0.851212 1.650636
H 3.684432 -1.316728 1.938470
C 6.698368 -0.041428 0.990723
H 6.970161 1.557525 -0.435759
H 6.113818 -1.590587 2.379779
H 7.766446 -0.150699 1.192835

Table S34. Cartesian coordinates of **Int1**
Ru 0.896842 -1.449475 -0.385658
Si -0.537899 1.049609 3.166739
P -1.815173 0.195752 0.398613
O -1.883160 -1.480126 -2.882426
S -1.056729 -0.275886 -2.678810
N -1.031371 0.282162 1.735512
H -0.171431 0.956807 -1.011359
C -0.628979 -0.043951 -0.977082
C -2.701409 1.740075 -0.036531
O 0.181460 -0.179049 -3.470430
C -1.928042 2.900569 -0.189850
H -0.844589 2.862586 -0.046535
C -2.529942 4.108968 -0.523986
H -1.912489 5.002233 -0.644962
C -4.690017 3.031833 -0.532282
H -5.774153 3.081840 -0.659741
C -4.088260 1.817494 -0.198869
H -4.702392 0.923357 -0.068807
C -3.129579 -1.069940 0.504762
C -4.830714 -2.552513 -0.367308
H -5.333703 -3.007766 -1.223917
C -3.794111 -1.645258 -0.584969
H -3.475385 -1.427380 -1.604896
C -5.220969 -2.882509 0.930063
H -6.034695 -3.593548 1.093607
C -0.639289 2.932070 3.080884
H 0.018071 3.335881 2.295880
H -0.341501 3.384383 4.041025
H -1.666720 3.258326 2.854269
C -1.627372 0.492640 4.609021
H -2.677105 0.786314 4.445327

H -1.298329 0.939379 5.561373

H -1.597109 -0.603123 4.726541

C -3.526949 -1.402933 1.805514

H -2.995386 -0.950187 2.644908

C -4.568112 -2.303467 2.017708

H -4.867924 -2.555734 3.037787

C 1.233046 0.544942 3.571788

H 1.925693 0.854487 2.774195

H 1.303887 -0.552090 3.650014

H 1.575813 0.980198 4.524269

C 2.467296 -2.870614 -2.878314

H 1.903039 -2.221819 -3.562893

H 2.624064 -3.848850 -3.362024

H 3.448532 -2.411845 -2.696142

C 1.610080 -3.094652 0.863624

H 2.120560 -3.015228 1.825692

C 0.180258 -3.189599 0.853549

C -0.452456 -3.246204 -0.408654

H -1.542119 -3.246558 -0.461976

C 0.290717 -3.160623 -1.615083

H -0.249708 -3.087329 -2.558589

C 1.708570 -3.034867 -1.599840

C 2.366097 -3.068134 -0.328983

H 3.449387 -2.941356 -0.279896

C -3.913359 4.176349 -0.700832

H -4.386507 5.125183 -0.965072

C -0.590318 -3.180085 2.136997

H -1.627293 -3.501309 1.976987

H -0.124953 -3.847476 2.877628

H -0.619435 -2.157003 2.543697

C -2.055281 1.112524 -3.194834

H -1.502910 2.041945 -3.007832

H -2.213383 0.959741 -4.270624

H -3.008865 1.116683 -2.653545

P 2.209224 0.288515 -0.127868

C 1.924735 2.015914 -0.628363

C 2.062297 3.046212 0.315404

C 1.517650 2.336791 -1.933174

C 1.782104 4.365501 -0.031946

H 2.381395 2.807891 1.333104

C 1.232128 3.659215 -2.273361

H 1.399278 1.537663 -2.671073
C 1.359942 4.674599 -1.326164
H 1.884512 5.155443 0.716011
H 0.912013 3.895790 -3.291342
H 1.136128 5.709552 -1.596374
C 3.951427 0.273049 0.363873
C 4.901741 1.135151 -0.207615
C 4.374433 -0.644640 1.339746
C 6.237938 1.065990 0.176596
H 4.588071 1.860831 -0.961846
C 5.711737 -0.712807 1.719187
H 3.633251 -1.287826 1.819159
C 6.649016 0.140765 1.136757
H 6.966783 1.739851 -0.280559
H 6.022682 -1.427135 2.485538
H 7.698264 0.090878 1.437064

Table S35. Cartesian coordinates of **TS4**
Ru 1.046708 -1.378078 -0.343663
Si -2.412538 -0.550059 3.127759
P -1.860588 0.269509 0.282409
O -1.757620 -1.318808 -2.794062
S -0.826494 -0.193434 -2.627474
N -1.675081 -0.599619 1.576249
H 0.011577 1.082168 -0.950236
C -0.465242 0.093241 -0.914900
C -1.945336 2.070747 0.636315
O 0.455322 -0.243137 -3.353635
C -0.760946 2.809893 0.755553
H 0.204504 2.353023 0.533207
C -0.787853 4.135160 1.182195
H 0.150015 4.689951 1.256973
C -3.182017 4.009411 1.419093
H -4.135677 4.470743 1.686909
C -3.155157 2.683486 0.991574
H -4.088471 2.120044 0.943163
C -3.444268 -0.145774 -0.547949
C -5.440800 0.324375 -1.842783
H -6.059437 1.036459 -2.394300
C -4.244759 0.749347 -1.266219
H -3.949347 1.796069 -1.364764

C -5.848594 -1.001027 -1.710068
H -6.784473 -1.334304 -2.164832
C -1.439570 0.595208 4.265395
H -0.388952 0.272126 4.340475
H -1.862523 0.631800 5.282330
H -1.441496 1.618288 3.855080
C -4.217805 0.014918 3.128599
H -4.305832 1.096932 2.950289
H -4.683647 -0.197700 4.104823
H -4.800637 -0.507786 2.353299
C -3.875808 -1.468878 -0.402127
H -3.268111 -2.156980 0.189579
C -5.063665 -1.897026 -0.985167
H -5.381602 -2.935918 -0.870415
C -2.413657 -2.298900 3.839402
H -1.400343 -2.715679 3.932101
H -2.998751 -2.975145 3.194710
H -2.874286 -2.311623 4.840423
C 0.814985 -3.349703 -3.113974
H -0.145810 -2.972137 -3.483469
H 0.905244 -4.417176 -3.377642
H 1.615427 -2.793195 -3.618637
C 2.284579 -3.020339 0.428165
H 3.270020 -2.921030 0.885660
C 1.111994 -2.889761 1.229227
C -0.158406 -2.991919 0.591531
H -1.057339 -2.816604 1.180089
C -0.247807 -3.145852 -0.808761
H -1.221828 -3.092834 -1.297559
C 0.909386 -3.194236 -1.631103
C 2.177905 -3.115188 -0.978730
H 3.084116 -3.093010 -1.587224
C -1.999255 4.741370 1.510099
H -2.021556 5.781463 1.844193
C 1.191396 -2.662855 2.705353
H 0.870970 -3.565342 3.249961
H 2.214725 -2.423107 3.021910
H 0.525349 -1.836158 2.987119
C -1.623470 1.265935 -3.284108
H -0.999558 2.140350 -3.058197
H -1.672208 1.096295 -4.367664

H -2.629061 1.367044 -2.867516
P 2.472858 0.278401 -0.138052
C 2.398882 2.000263 -0.710696
C 2.823817 3.046695 0.126636
C 1.889489 2.308614 -1.982240
C 2.719955 4.370042 -0.290425
H 3.232502 2.816450 1.114049
C 1.776918 3.638641 -2.387523
H 1.585585 1.494627 -2.647887
C 2.186039 4.670925 -1.545347
H 3.053281 5.174253 0.370502
H 1.378890 3.867176 -3.379661
H 2.098997 5.711059 -1.868327
C 4.155444 0.153447 0.532854
C 4.370704 -0.499758 1.755278
C 5.261478 0.674068 -0.158434
C 5.658555 -0.647208 2.266308
H 3.509207 -0.870539 2.314890
C 6.546951 0.526902 0.354458
H 5.108257 1.195208 -1.106898
C 6.750542 -0.137485 1.565156
H 5.808748 -1.151866 3.223995
H 7.399155 0.931695 -0.197049
H 7.760897 -0.250360 1.965339

Table S36. Cartesian coordinates of **Int3**
Ru 0.909107 -1.259387 -0.271156
P -1.781228 0.231562 0.608203
O -2.171148 -0.979545 -2.458978
S -1.210634 0.132306 -2.370431
N -2.906688 -0.834986 0.662719
H -0.048390 1.228601 -0.731435
C -0.541726 0.246482 -0.738783
C -0.662580 0.022986 2.034332
O -0.103437 0.149536 -3.337342
C 0.715031 -0.159725 1.863535
H 1.256016 0.155626 0.907133
C 1.536790 -0.399643 2.970376
H 2.613729 -0.501631 2.829975
C -0.401326 -0.324215 4.411348
H -0.840622 -0.398308 5.409056

C -1.218561 -0.079250 3.310874
H -2.301450 0.012399 3.429166
C -2.381693 1.953775 0.788558
C -2.023086 4.347278 0.781735
H -1.350009 5.197026 0.646545
C -1.531114 3.054319 0.627332
H -0.475106 2.917125 0.382878
C -3.365069 4.552390 1.104817
H -3.750381 5.568204 1.222112
C -3.721063 2.164324 1.128558
H -4.372674 1.300036 1.272736
C -4.212368 3.459825 1.283065
H -5.261913 3.615594 1.543922
C 2.309763 -2.529510 -3.036585
H 1.771159 -1.791674 -3.647350
H 2.386088 -3.475284 -3.597476
H 3.321112 -2.143145 -2.852650
C 1.531174 -3.174052 0.666254
H 2.069147 -3.288470 1.610697
C 0.094323 -3.159758 0.689197
C -0.574710 -2.935863 -0.523893
H -1.656683 -2.813757 -0.534462
C 0.162836 -2.700498 -1.721738
H -0.382902 -2.441926 -2.628181
C 1.576325 -2.762163 -1.758138
C 2.259398 -3.022446 -0.528799
H 3.350322 -3.035091 -0.513051
C 0.976624 -0.479080 4.242298
H 1.617921 -0.659778 5.108090
C -0.664661 -3.328965 1.965618
H -0.856670 -4.398535 2.150637
H -0.099929 -2.932777 2.821328
H -1.627721 -2.802273 1.902215
C -2.128002 1.640918 -2.632065
H -1.451860 2.494510 -2.496527
H -2.485845 1.583787 -3.668645
H -2.971896 1.688102 -1.932906
P 2.608640 0.225267 -1.177498
C 2.109130 1.994699 -0.967600
C 2.293691 2.733879 0.213146
C 1.525845 2.644216 -2.069318

C 1.929008 4.077726 0.283910
H 2.747509 2.255901 1.084870
C 1.145672 3.983877 -1.992524
H 1.361958 2.075905 -2.988567
C 1.355220 4.709786 -0.819700
H 1.075360 5.764867 -0.766943
C 4.063400 0.228274 -0.033542
C 5.063683 1.197067 -0.245376
C 4.317319 -0.751735 0.935363
C 6.243175 1.193770 0.490847
H 4.909202 1.967872 -1.005409
C 5.504686 -0.765320 1.669921
H 3.562991 -1.514633 1.126603
C 6.473604 0.210303 1.455581
H 7.402501 0.205552 2.030512
Si -4.408919 -1.379737 0.042851
C -5.172287 -0.256232 -1.266801
H -5.285120 0.783404 -0.921475
H -4.534184 -0.265510 -2.162908
H -6.171326 -0.624087 -1.552492
C -5.606863 -1.504000 1.498637
H -5.797080 -0.518442 1.954285
H -6.578441 -1.922518 1.189676
H -5.189079 -2.154736 2.283525
C -4.240187 -3.094590 -0.715806
H -3.610912 -3.045077 -1.617739
H -3.793524 -3.810868 -0.007616
H -5.226536 -3.487169 -1.011067
H 2.090924 4.633230 1.211254
H 6.996293 1.963898 0.304576
H 0.699409 4.470052 -2.864229
H 5.666805 -1.544100 2.420319

Table S37. Cartesian coordinates of **TS5**
Ru 0.890972 -1.271408 -0.141171
P -1.808783 0.247842 0.633317
O -2.115821 -1.134170 -2.413281
S -1.176217 -0.002929 -2.342914
N -2.885578 -0.866994 0.743250
H -0.065703 1.176939 -0.722456
C -0.556166 0.194098 -0.698934

C -0.623903 0.216432 2.003274
O -0.039955 -0.003165 -3.276653
C 0.644041 -0.342600 1.751011
H 1.582012 0.010577 0.597590
C 1.527055 -0.461297 2.836272
H 2.532965 -0.852347 2.677446
C -0.118719 0.464387 4.353545
H -0.412651 0.771621 5.359728
C -1.005315 0.603446 3.289453
H -2.008248 1.007862 3.453708
C -2.487806 1.950601 0.678121
C -2.228327 4.349847 0.478462
H -1.592380 5.213640 0.270338
C -1.685505 3.069647 0.422825
H -0.626249 2.953158 0.183109
C -3.575282 4.525910 0.799668
H -4.001240 5.531385 0.840808
C -3.831196 2.134268 1.016157
H -4.445854 1.259443 1.237601
C -4.374281 3.417592 1.074535
H -5.426720 3.550701 1.336302
C 2.352787 -2.736778 -2.925851
H 1.692806 -2.751863 -3.801667
H 3.126841 -3.510492 -3.036793
H 2.851107 -1.748642 -2.897470
C 1.536082 -3.200124 0.796202
H 2.076555 -3.302632 1.739715
C 0.103087 -3.177657 0.811028
C -0.552925 -2.972059 -0.416508
H -1.632415 -2.832738 -0.435806
C 0.181273 -2.853212 -1.635793
H -0.372519 -2.664196 -2.554359
C 1.580472 -2.928768 -1.666254
C 2.253622 -3.058991 -0.408126
H 3.345188 -3.067144 -0.392583
C 1.148907 -0.067853 4.119732
H 1.859632 -0.172760 4.943521
C -0.674830 -3.293958 2.081901
H -0.948022 -4.346688 2.259469
H -0.090578 -2.938605 2.941982
H -1.596449 -2.696943 2.008059

C -2.116068 1.473214 -2.695205
H -1.462245 2.347416 -2.584319
H -2.444922 1.358884 -3.736686
H -2.979051 1.533728 -2.020895
P 2.570777 0.198761 -1.088941
C 2.064644 1.976038 -1.008824
C 2.156744 2.756218 0.154963
C 1.567274 2.567934 -2.179020
C 1.779486 4.097280 0.142935
H 2.535514 2.309860 1.078187
C 1.177991 3.908253 -2.185895
H 1.466093 1.956908 -3.079365
C 1.291421 4.678093 -1.029048
H 0.998655 5.730975 -1.038926
C 4.079655 0.289364 -0.024594
C 5.005993 1.321684 -0.255905
C 4.436510 -0.710018 0.889702
C 6.224112 1.358172 0.415736
H 4.767162 2.111216 -0.973035
C 5.657647 -0.677378 1.562226
H 3.738687 -1.524140 1.087223
C 6.558128 0.359422 1.331319
H 7.514408 0.389802 1.858458
Si -4.380236 -1.460755 0.153417
C -5.143324 -0.434477 -1.235436
H -5.284366 0.621771 -0.957864
H -4.488041 -0.484810 -2.117957
H -6.128881 -0.841268 -1.515395
C -5.595590 -1.515595 1.599022
H -5.807823 -0.508498 1.993537
H -6.556030 -1.967942 1.303064
H -5.177506 -2.110384 2.426810
C -4.189959 -3.217659 -0.500828
H -3.574742 -3.216597 -1.414033
H -3.721693 -3.881497 0.243553
H -5.173395 -3.643475 -0.757377
H 6.921939 2.176232 0.219595
H 5.902174 -1.467815 2.276738
H 1.859751 4.690366 1.057226
H 0.795146 4.356121 -3.106549

Table S38. Cartesian coordinates of **Int4**

Ru 0.852788 -1.306678 -0.026595
P -1.878443 0.241699 0.632322
O -2.016809 -1.114714 -2.455583
S -1.086350 0.016696 -2.310805
N -2.977133 -0.857213 0.649973
H -0.045513 1.158309 -0.613844
C -0.551309 0.182568 -0.632771
C -0.755092 0.201635 2.043477
O 0.096803 0.037495 -3.187627
C 0.491789 -0.404741 1.809996
H 1.910513 -0.051470 0.381718
C 1.376889 -0.465630 2.899472
H 2.368609 -0.906589 2.775188
C -0.228703 0.614706 4.361861
H -0.502275 1.003224 5.345504
C -1.119996 0.699928 3.297667
H -2.104508 1.156969 3.434014
C -2.533162 1.956228 0.635892
C -2.213979 4.351932 0.477850
H -1.547020 5.205654 0.335328
C -1.691039 3.062458 0.464139
H -0.615907 2.926683 0.323615
C -3.581687 4.550784 0.675168
H -3.991875 5.563610 0.683818
C -3.897616 2.162981 0.852159
H -4.544900 1.298328 1.012027
C -4.421417 3.455822 0.869339
H -5.490726 3.606633 1.035925
C 2.426200 -2.797597 -2.759127
H 3.408998 -2.364092 -2.525632
H 1.920185 -2.129429 -3.469599
H 2.586652 -3.776123 -3.239808
C 1.437312 -3.231544 0.937546
H 1.931252 -3.342751 1.904693
C 0.007097 -3.170211 0.879289
C -0.584798 -2.951582 -0.383616
H -1.659241 -2.785899 -0.448639
C 0.203452 -2.870647 -1.572897
H -0.303834 -2.677225 -2.517314
C 1.599778 -2.954425 -1.525629

C 2.207664 -3.093601 -0.234329

H 3.297477 -3.099991 -0.164854

C 1.023099 0.033438 4.153408

H 1.738775 -0.029905 4.977847

C -0.840641 -3.275527 2.105268

H -1.143656 -4.324428 2.255895

H -0.297548 -2.937239 2.998090

H -1.745266 -2.660394 1.984894

C -2.009601 1.497484 -2.688010

H -1.368402 2.371950 -2.520451

H -2.275580 1.402925 -3.749240

H -2.911035 1.541592 -2.064888

P 2.587117 0.203568 -1.041589

C 2.119873 1.986340 -0.922271

C 2.223476 2.719665 0.269273

C 1.641334 2.619862 -2.076291

C 1.864162 4.064737 0.301188

H 2.585971 2.230977 1.177502

C 1.278046 3.967300 -2.039768

H 1.527492 2.037025 -2.993632

C 1.394099 4.691749 -0.854783

H 1.115466 5.748205 -0.829080

C 4.184540 0.258559 -0.111380

C 5.161568 1.176431 -0.526045

C 4.513942 -0.653026 0.897482

C 6.422909 1.186165 0.062399

H 4.928366 1.897753 -1.314338

C 5.778799 -0.646418 1.484485

H 3.762562 -1.368018 1.238579

C 6.738812 0.273792 1.069944

H 7.729258 0.282834 1.530609

Si -4.437896 -1.441547 -0.021948

C -5.112663 -0.418521 -1.458703

H -5.268416 0.639007 -1.193953

H -4.404270 -0.472965 -2.298898

H -6.080105 -0.824199 -1.797377

C -5.739009 -1.483500 1.347389

H -5.965094 -0.473398 1.726227

H -6.683867 -1.928479 0.995312

H -5.376142 -2.079170 2.200180

C -4.218293 -3.202069 -0.658644

H -3.550567 -3.206960 -1.534268
H -3.795853 -3.863970 0.114372
H -5.186389 -3.626065 -0.970595
H 1.945525 4.625496 1.235366
H 0.907157 4.453756 -2.945410
H 7.167523 1.913602 -0.270332
H 6.010543 -1.361330 2.278099

Table S39. Cartesian coordinates of **TS6**
Ru 0.779294 -1.366952 0.021369
P -1.957028 0.214036 0.617658
O -1.923114 -1.018130 -2.552606
S -1.012954 0.107314 -2.289044
N -3.092652 -0.837352 0.504536
H -0.053020 1.145499 -0.487328
C -0.570183 0.178319 -0.578531
C -0.921129 0.056919 2.087529
O 0.214659 0.186492 -3.101272
C 0.316323 -0.580353 1.883306
H 2.019433 0.177006 0.842389
C 1.136739 -0.733414 3.015561
H 2.117634 -1.209281 2.918436
C -0.510164 0.320228 4.450066
H -0.825302 0.661562 5.438831
C -1.338738 0.497997 3.346333
H -2.313047 0.983022 3.457565
C -2.545514 1.953400 0.672124
C -2.127262 4.339579 0.671926
H -1.420080 5.171893 0.637453
C -1.651387 3.032190 0.638711
H -0.572397 2.860457 0.590899
C -3.499858 4.583262 0.750979
H -3.872649 5.610212 0.775193
C -3.915832 2.204401 0.769826
H -4.604441 1.358122 0.819552
C -4.392779 3.514977 0.806993
H -5.467080 3.700728 0.880223
C 2.693320 -2.545583 -2.610995
H 3.592601 -2.042019 -2.226938
H 2.226256 -1.890900 -3.359113
H 3.011131 -3.479479 -3.102389

C 1.310660 -3.329979 0.897235

H 1.700821 -3.507438 1.901161

C -0.106313 -3.242128 0.697296

C -0.556773 -2.926815 -0.612673

H -1.619130 -2.753194 -0.780850

C 0.349462 -2.792298 -1.712575

H -0.052450 -2.537004 -2.692508

C 1.730251 -2.833711 -1.504982

C 2.198094 -3.076923 -0.164844

H 3.273281 -3.062245 0.026896

C 0.731752 -0.293029 4.276499

H 1.396154 -0.429511 5.134502

C -1.080944 -3.437171 1.812789

H -1.417976 -4.486422 1.835752

H -0.628905 -3.193942 2.784073

H -1.956489 -2.787879 1.659181

C -1.924469 1.604451 -2.631332

H -1.302006 2.470990 -2.374944

H -2.129420 1.572963 -3.709690

H -2.859906 1.607082 -2.058957

P 2.540815 0.360402 -0.497574

C 2.172187 2.151309 -0.603499

C 2.301017 2.991222 0.512520

C 1.710153 2.680178 -1.815777

C 1.970443 4.340582 0.416224

H 2.653549 2.580240 1.461776

C 1.377279 4.032478 -1.905468

H 1.567756 2.013219 -2.670085

C 1.507215 4.862171 -0.793182

H 1.244126 5.920343 -0.866315

C 4.297194 0.265690 -0.004048

C 5.250219 1.057893 -0.656701

C 4.724060 -0.653996 0.962656

C 6.601409 0.936675 -0.339291

H 4.931630 1.780957 -1.412391

C 6.076329 -0.776640 1.272241

H 3.986544 -1.267770 1.486725

C 7.020414 0.018011 0.622226

H 8.080627 -0.076338 0.867498

Si -4.503010 -1.398018 -0.279589

C -5.084372 -0.318138 -1.716388

H -5.254518 0.729045 -1.420234
H -4.325765 -0.342935 -2.513121
H -6.029801 -0.705514 -2.130373
C -5.889552 -1.481949 1.000626
H -6.128442 -0.483910 1.402977
H -6.814267 -1.902183 0.572810
H -5.586459 -2.113675 1.850781
C -4.240569 -3.133030 -0.966829
H -3.530404 -3.100747 -1.807747
H -3.848213 -3.816889 -0.197292
H -5.187818 -3.554164 -1.340624
H 7.333752 1.565450 -0.851549
H 2.067367 4.986768 1.291892
H 6.393594 -1.491707 2.035303
H 1.011514 4.438025 -2.852011

Table S40. Cartesian coordinates of **3’**
Ru 0.928415 -1.143618 0.626780
O -0.993978 -1.603547 -2.537002
S -0.334698 -0.312042 -2.289477
P -1.960162 0.160934 0.262438
N -2.920913 -1.057932 0.161496
C -0.335281 0.101480 -0.579356
H 0.054203 1.129035 -0.574992
P 2.451134 0.552099 0.672103
C -2.750578 1.756973 -0.185621
O 1.039279 -0.136966 -2.802732
C -2.005117 2.938629 -0.278714
H -0.931771 2.931026 -0.078918
C -2.626143 4.137471 -0.615811
H -2.030937 5.050865 -0.689640
Si -4.082466 -1.904567 -0.761758
C -4.753235 3.002837 -0.739762
H -5.832083 3.025388 -0.911776
C -4.129314 1.801010 -0.404185
H -4.710545 0.882629 -0.302322
C -1.269339 0.471378 1.905514
C 0.030275 -0.032141 2.115297
C 0.576263 0.160904 3.396948
H 1.581637 -0.211074 3.621160
C -0.131602 0.819810 4.403577

H 0.325932 0.950863 5.388530
C -1.416345 1.311210 4.163821
H -1.967915 1.827661 4.952844
C -1.986873 1.131281 2.907009
H -2.993161 1.506328 2.698012
C -4.363973 -1.214130 -2.497349
H -4.670458 -0.156276 -2.493337
H -5.149609 -1.787476 -3.016293
H -3.431376 -1.311372 -3.073894
H 3.007866 0.763235 1.963522
C -3.584266 -3.710194 -0.980578
H -2.706395 -3.788251 -1.641074
H -4.404121 -4.281731 -1.444958
H -3.345291 -4.188157 -0.017196
C -5.725640 -1.880534 0.171829
H -5.592445 -2.290886 1.185591
H -6.492484 -2.481608 -0.343365
H -6.120662 -0.857604 0.282687
C -1.235657 -2.996707 2.451948
H -1.029480 -2.472272 3.395235
H -1.496808 -4.041910 2.684428
H -2.094973 -2.514891 1.961046
C -0.049675 -2.957392 1.542806
C -0.204853 -3.010387 0.144390
H -1.205278 -3.009497 -0.283454
C 0.924505 -2.985147 -0.731284
H 0.746929 -3.010632 -1.806191
C 2.232320 -2.889047 -0.234110
C 2.388436 -2.736342 1.183098
H 3.389489 -2.589941 1.596239
C 1.280607 -2.792717 2.052848
H 1.429443 -2.676262 3.127735
C 4.002496 0.409526 -0.277115
C 3.952357 0.172482 -1.656910
H 2.983340 0.038018 -2.149637
C 5.136417 0.077247 -2.385226
H 5.091775 -0.101845 -3.462194
C 6.370280 0.188941 -1.744877
H 7.295875 0.102683 -2.319254
C 6.422531 0.405571 -0.368210
H 7.386824 0.490117 0.138594

C 5.242124 0.523647 0.363348
H 5.287133 0.709252 1.440148
C 1.905477 2.274532 0.348665
C 1.885232 2.797518 -0.950880
H 2.192689 2.178622 -1.796521
C 1.455166 4.104742 -1.171997
H 1.447753 4.504770 -2.188660
C 1.039641 4.898649 -0.102912
H 0.711664 5.926126 -0.278098
C 1.034317 4.373391 1.188358
H 0.692053 4.981743 2.028524
C 1.460727 3.065563 1.415148
H 1.436663 2.654973 2.427512
C -4.001542 4.170727 -0.852979
H -4.488672 5.111928 -1.119570
C 3.418930 -2.967390 -1.138864
H 4.272705 -2.401119 -0.744383
H 3.730395 -4.020154 -1.242714
H 3.178372 -2.582665 -2.138229
C -1.307298 0.932476 -3.123342
H -2.350207 0.875391 -2.789582
H -0.879370 1.920711 -2.914340
H -1.228085 0.689007 -4.191137

Table S41. Cartesian coordinates of **TS2**
Ru 1.153696 -1.535207 -0.439664
Si -0.742565 3.056186 1.290529
P -1.360631 0.225636 0.413246
O -2.192077 -3.006411 -0.867074
S -1.768260 -1.963355 -1.818838
N -0.404053 1.497803 0.583808
H -0.664106 0.030110 -1.808332
C -0.768710 -0.737703 -1.023378
C -3.058184 0.795262 0.066540
O -1.044755 -2.375261 -3.030604
C -3.207894 1.686987 -1.006322
H -2.324072 2.021210 -1.556869
C -4.469205 2.146985 -1.367029
H -4.574985 2.840623 -2.204630
C -5.452220 0.850836 0.416530
H -6.329936 0.526796 0.980671

C -4.188085 0.386611 0.781782
H -4.082410 -0.294017 1.628635
C -1.469100 -0.780174 1.925249
C -2.331432 -2.622682 3.229919
H -2.889278 -3.560709 3.284501
C -2.185937 -1.982174 2.000640
H -2.599505 -2.426625 1.092299
C -1.772353 -2.073442 4.385275
H -1.899460 -2.576161 5.347147
C -1.345620 4.331586 0.033956
H -0.809901 4.271551 -0.923694
H -1.211182 5.347752 0.439763
H -2.418807 4.195084 -0.168473
C -2.075343 3.010645 2.630168
H -3.032019 2.638884 2.231893
H -2.244832 4.036511 2.997740
H -1.802612 2.386118 3.493406
C -0.881085 -0.251351 3.078231
H -0.294608 0.665477 2.994788
C -1.037542 -0.891662 4.305894
H -0.578796 -0.467819 5.202379
C 0.835735 3.691416 2.097757
H 1.682711 3.767427 1.399487
H 1.155675 3.020753 2.910205
H 0.659733 4.689488 2.530617
C 2.093984 -3.505192 -3.056755
H 1.109993 -3.384566 -3.530545
H 2.473829 -4.516159 -3.282552
H 2.788126 -2.776334 -3.499186
C 2.856175 -2.467813 0.587431
H 3.640358 -1.935804 1.130612
C 1.687912 -2.897801 1.283730
C 0.667243 -3.522707 0.523221
H -0.267031 -3.820388 0.999399
C 0.819221 -3.719858 -0.874572
H -0.018337 -4.134634 -1.434392
C 1.971504 -3.298689 -1.580335
C 2.983484 -2.643765 -0.815254
H 3.864963 -2.247794 -1.323183
C -5.596403 1.726194 -0.658067
H -6.587810 2.087467 -0.941146

C 1.583487 -2.773245 2.769488
H 0.536105 -2.746197 3.092124
H 2.066689 -3.638243 3.254679
H 2.084621 -1.866529 3.135546
C -3.281059 -1.238471 -2.439216
H -3.042591 -0.382764 -3.081701
H -3.729615 -2.047107 -3.031618
H -3.940496 -0.948234 -1.614478
P 1.632794 0.563082 -0.444767
C 1.355485 1.696341 -1.842451
C 1.401883 3.092371 -1.736247
C 1.125540 1.118995 -3.101035
C 1.219681 3.892969 -2.860838
H 1.575477 3.557126 -0.764465
C 0.918716 1.922017 -4.220662
H 1.111375 0.026753 -3.182113
C 0.969686 3.310923 -4.104309
H 1.261724 4.980669 -2.763306
H 0.730961 1.457197 -5.191552
H 0.821540 3.941783 -4.984162
C 2.947304 1.316055 0.545188
C 3.921198 2.195771 0.051962
C 3.000486 0.924491 1.891791
C 4.923160 2.674143 0.893672
H 3.901026 2.499837 -0.996833
C 4.000661 1.405868 2.730112
H 2.232281 0.241918 2.265878
C 4.963449 2.285312 2.232876
H 5.681797 3.354583 0.499191
H 4.026680 1.100801 3.779197
H 5.747821 2.668063 2.890186

Table S42. Cartesian coordinates of **Int2**
Ru 0.567221 -1.570620 -0.474269
Si 0.052025 3.019030 1.270334
P -1.232325 0.330871 0.218666
O -2.940158 -2.254798 -1.233300
S -2.245647 -1.365554 -2.182014
N 0.088016 1.361224 0.545343
H -0.670993 0.316165 -2.108738
C -1.006210 -0.430953 -1.371556

C -2.559106 1.616449 -0.056619

O -1.643506 -1.959455 -3.380049

C -2.392514 2.488055 -1.144480

H -1.452760 2.481009 -1.703929

C -3.405222 3.362582 -1.523861

H -3.251343 4.039926 -2.367684

C -4.799232 2.504973 0.246093

H -5.743401 2.506333 0.796247

C -3.776816 1.637060 0.633286

H -3.928702 0.976303 1.488459

C -1.849013 -0.556373 1.665865

C -3.469757 -1.945102 2.802964

H -4.353695 -2.586064 2.758814

C -2.990450 -1.369514 1.627509

H -3.477071 -1.577130 0.673805

C -2.833881 -1.716336 4.021494

H -3.217586 -2.170363 4.937955

C 0.024139 4.396207 -0.012000

H 0.578213 4.137023 -0.924920

H 0.485186 5.300101 0.418530

H -1.008338 4.645973 -0.294135

C -1.410156 3.254184 2.426447

H -2.331701 3.509849 1.887583

H -1.162080 4.094876 3.095691

H -1.612247 2.374196 3.053844

C -1.218515 -0.316693 2.895848

H -0.344476 0.334502 2.930359

C -1.702365 -0.898528 4.062028

H -1.197370 -0.704254 5.011481

C 1.586595 3.196683 2.341481

H 2.544241 3.081807 1.816631

H 1.582705 2.468107 3.165763

H 1.561416 4.207252 2.781698

C 3.422651 -2.558186 -2.106090

H 3.139120 -2.270255 -3.128132

H 4.103134 -3.423661 -2.174324

H 3.985419 -1.725565 -1.659158

C 1.148026 -3.145214 0.959774

H 1.234017 -3.078038 2.046540

C -0.054830 -3.619734 0.367390

C -0.107578 -3.649477 -1.049678

H -1.052170 -3.907349 -1.528862
C 1.006890 -3.324475 -1.875042
H 0.895654 -3.361262 -2.959274
C 2.215271 -2.896910 -1.285928
C 2.260337 -2.805573 0.142806
H 3.164805 -2.418459 0.618466
C -4.617122 3.370712 -0.830021
H -5.417408 4.051227 -1.130185
C -1.212392 -4.085500 1.187390
H -2.157394 -3.857464 0.676376
H -1.160695 -5.177075 1.340808
H -1.223395 -3.602908 2.174179
C -3.493206 -0.230473 -2.775770
H -3.027965 0.506196 -3.441647
H -4.200693 -0.858291 -3.333693
H -3.987741 0.259664 -1.928460
P 1.449835 0.387600 -0.026133
C 2.125912 1.426671 -1.395908
C 2.705825 2.693807 -1.245601
C 2.008030 0.887169 -2.682487
C 3.150059 3.406069 -2.357347
H 2.819249 3.134240 -0.253181
C 2.446614 1.601696 -3.797044
H 1.563146 -0.110675 -2.779232
C 3.017738 2.862457 -3.636069
H 3.601258 4.392616 -2.225094
H 2.343755 1.167640 -4.794598
H 3.365671 3.423438 -4.506971
C 2.772947 0.417504 1.238719
C 4.092304 0.788898 0.958474
C 2.478298 -0.135320 2.493104
C 5.086486 0.650054 1.928815
H 4.353543 1.181486 -0.025830
C 3.465761 -0.259804 3.463992
H 1.466892 -0.497631 2.686808
C 4.774877 0.139638 3.186095
H 6.112939 0.943099 1.695230
H 3.217721 -0.684838 4.439690
H 5.553591 0.036170 3.945447

Table S43. Cartesian coordinates of **TS3**

Ru -0.241090 -1.504048 -0.341054

Si 1.444726 2.575539 1.715370

P -1.100452 0.565488 0.115635

O -3.928690 -1.343171 -0.774443

S -3.117419 -0.922339 -1.932299

N 0.795832 1.121079 0.964046

H -1.216546 0.284392 -2.318556

C -1.595282 -0.253539 -1.440095

C -1.149240 2.289002 -0.546885

O -2.928727 -1.897264 -3.017808

C -0.468516 2.639333 -1.721308

H 0.175908 1.915486 -2.222267

C -0.585956 3.917536 -2.255174

H -0.040420 4.168811 -3.167976

C -2.050330 4.540862 -0.451645

H -2.670630 5.281964 0.058531

C -1.934996 3.256689 0.086614

H -2.468876 3.014468 1.007168

C -2.253481 0.558516 1.518677

C -4.454153 0.607794 2.515714

H -5.537138 0.697375 2.404032

C -3.639424 0.689188 1.387721

H -4.091945 0.824103 0.406904

C -3.894476 0.408744 3.776601

H -4.538109 0.351000 4.657740

C 1.605047 4.057488 0.563380

H 2.145667 3.819287 -0.363819

H 2.155105 4.859408 1.083371

H 0.620003 4.454091 0.279428

C 0.287437 3.071728 3.112822

H -0.745386 3.199804 2.757602

H 0.618570 4.030814 3.542974

H 0.275483 2.324322 3.920545

C -1.695894 0.344021 2.784616

H -0.612589 0.233101 2.861739

C -2.511704 0.278910 3.909963

H -2.066962 0.122352 4.895726

C 3.148472 2.276225 2.479965

H 3.894418 1.896987 1.765006

H 3.100820 1.553764 3.307926

H 3.525361 3.230550 2.883802

C 1.912645 -3.725826 -1.936333

H 1.622717 -3.533181 -2.978376

H 2.256265 -4.771372 -1.866369

H 2.766565 -3.079135 -1.688384

C -0.053972 -2.990389 1.308754

H 0.183556 -2.799351 2.356523

C -1.409286 -3.067981 0.875871

C -1.631486 -3.310616 -0.497080

H -2.655424 -3.307367 -0.866512

C -0.570209 -3.497793 -1.433113

H -0.823156 -3.618751 -2.487199

C 0.764809 -3.486879 -1.006595

C 0.993006 -3.200395 0.378241

H 2.023531 -3.119521 0.726130

C -1.382261 4.875733 -1.625415

H -1.473441 5.880059 -2.045325

C -2.561916 -2.908113 1.812964

H -3.384717 -2.385689 1.305326

H -2.923939 -3.897703 2.136687

H -2.276554 -2.337335 2.706042

C -3.969805 0.472488 -2.664030

H -3.407279 0.813778 -3.541582

H -4.957720 0.096877 -2.961628

H -4.059343 1.277512 -1.923434

P 1.497535 -0.049772 0.015567

C 2.357018 0.616334 -1.467444

C 3.360699 1.589967 -1.392987

C 1.963158 0.135476 -2.720852

C 3.941610 2.090632 -2.555299

H 3.694127 1.958668 -0.420563

C 2.549292 0.631875 -3.885445

H 1.175463 -0.626257 -2.757110

C 3.533789 1.615112 -3.803311

H 4.718067 2.856639 -2.488468

H 2.232254 0.251508 -4.859598

H 3.990256 2.011065 -4.713833

C 2.892976 -0.775665 0.956182

C 4.077127 -1.206658 0.349566

C 2.703475 -1.005625 2.324736

C 5.055514 -1.859104 1.101132

H 4.238202 -1.029208 -0.716579

C 3.678921 -1.656659 3.073299
H 1.776792 -0.662395 2.791426
C 4.858514 -2.086459 2.461792
H 5.978936 -2.189087 0.618945
H 3.522517 -1.828504 4.141111
H 5.625610 -2.596839 3.048944

Table S44. Cartesian coordinates of **4’**
C 3.527633 0.468168 -0.243263
P -1.515798 0.221635 -0.082035
O 2.935409 -2.477192 1.395593
Ru 0.303469 -1.183305 -0.782983
Si -2.377912 0.791561 2.897508
P 1.707222 0.463461 -0.097842
O 0.951682 -2.482293 2.945953
S 1.926874 -1.719031 2.155148
C 1.033000 -0.618987 1.141560
H 0.205758 -0.162685 1.728953
C -2.204421 2.877171 -0.600765
H -2.478213 2.871258 0.457006
C -2.362444 4.020046 -1.381915
C -1.979860 4.012109 -2.723237
H -2.104578 4.909377 -3.334723
C -1.425430 2.858593 -3.278297
C -1.262151 1.719456 -2.492155
H -0.794107 0.823847 -2.905281
C -1.662387 1.715005 -1.153800
C -3.828704 -0.447127 -1.657761
H -3.502155 0.302434 -2.382650
C -4.991001 -1.182926 -1.899450
C -5.432816 -2.124427 -0.972423
H -6.344535 -2.696067 -1.161728
C -4.709478 -2.325551 0.205285
C -3.549957 -1.593814 0.444372
H -2.977220 -1.753978 1.361115
C 1.313916 3.059050 -0.955274
H 1.559800 2.661783 -1.943313
C 4.225140 -0.707781 -0.548999
H 3.681027 -1.642066 -0.660660
C 6.324387 0.484955 -0.445098
H 7.414069 0.490779 -0.524988

C 5.613440 -0.697495 -0.645103
H 6.142824 -1.625840 -0.871322
C 5.638764 1.658050 -0.136379
H 6.186736 2.588623 0.029034
C 4.249226 1.652836 -0.033268
H 3.723766 2.576970 0.212971
C 1.286580 2.205725 0.155543
C 1.016215 4.408843 -0.805304
H 1.027702 5.067191 -1.676273
C 0.682217 4.911190 0.453070
H 0.440231 5.970233 0.570442
C 0.643225 4.062169 1.556843
H 0.369298 4.449284 2.540662
C 0.942269 2.708942 1.411209
H 0.888348 2.039795 2.269489
C -1.615389 2.178560 3.933458
H -2.162243 2.313831 4.880615
H -1.639556 3.133364 3.385011
H -0.564980 1.957980 4.185219
C -2.239957 -0.793732 3.919772
H -2.518529 -0.616869 4.971610
H -1.210968 -1.188344 3.892473
H -2.901321 -1.588070 3.537885
C -4.208235 1.204145 2.664045
H -4.715408 0.431582 2.065211
H -4.332874 2.164429 2.137744
H -4.727242 1.280317 3.633725
C -0.144968 -3.395523 -0.665902
C -1.109092 -2.784143 -1.492091
H -2.163290 -2.885894 -1.237608
C -0.746211 -1.960817 -2.598495
H -1.533940 -1.470331 -3.172826
C 0.610247 -1.760189 -2.936931
C 1.589363 -2.430697 -2.137799
H 2.644566 -2.279518 -2.370494
C 1.230719 -3.205201 -1.020557
H 2.008881 -3.611638 -0.373056
N -1.521789 0.675862 1.433081
C -3.092853 -0.652475 -0.487483
C -0.530867 -4.231563 0.512359
H -0.370288 -5.298823 0.289248

H 0.066065 -3.963401 1.395949
H -1.591270 -4.087459 0.759032
C 1.029942 -0.945890 -4.121212
H 1.926670 -0.352373 -3.894104
H 1.267305 -1.606159 -4.971418
H 0.233634 -0.262134 -4.444475
C 2.828796 -0.680946 3.293685
H 2.112686 -0.063995 3.851078
H 3.359776 -1.363263 3.970816
H 3.538079 -0.064907 2.725373
H -1.109973 2.850873 -4.325141
H -2.781560 4.926824 -0.938477
H -5.560204 -1.009587 -2.816323
H -5.053358 -3.055266 0.942764

4.2.3 2a' + $HPAr^{Cl}_2$

Table S45. SCF energies, enthalpy and free energy corrections and barriers

	E_{SCF}/E_H	$corrH/E_H$	$corrG/E_H$	ΔH/kJ/mol	ΔG/kJ/mol
**TS-Act1**	-4941.574212	0.724512	0.581178	-34.69025891	32.47528209
**Coord**	-4941.603206	0.725947	0.585683	-107.0466497	-31.82082371
**TS-Act2**	-4941.577267	0.722855	0.582622	-47.06282264	28.24439386
**Act'**	-4941.621551	0.728162	0.587195	-149.3962798	-76.01618027
**TS-Act3**	-4941.620924	0.726986	0.590128	-150.8376268	-66.66934776
**Act**	-4941.626218	0.727802	0.586638	-162.5943007	-89.7314247
**TS1**	-4941.602904	0.726804	0.584937	-104.0035902	-32.98644069
**Int1**	-4941.60254	0.727743	0.582561	-100.5813034	-38.26768644
**TS4**	-4941.589459	0.726937	0.585826	-68.35462995	4.647397552
**Int3**	-4941.605685	0.727024	0.587728	-110.7283884	-32.96107835
**TS5**	-4941.593662	0.722288	0.582702	-91.59652738	-14.59061239
**Int4**	-4941.596476	0.724196	0.583123	-93.97441648	-20.87261998
**TS6**	-4941.586085	0.723069	0.582224	-69.65178448	4.048626019
**3‘**	-4941.637999	0.725918	0.586634	-198.4705767	-120.6717607
**TS2**	-4941.594062	0.726554	0.588485	-81.44524168	-0.456443176
**Int2**	-4941.60977	0.727413	0.587517	-120.4322626	-44.24025261
**TS3**	-4941.596841	0.726157	0.588276	-89.78312079	-8.30072829
**4‘**	-4941.638571	0.727613	0.585086	-195.5231379	-126.2388184

Table S46. Cartesian coordinates of **TS-Act1**

Ru -1.065909 1.009029 1.294294
N -1.487696 -1.081545 0.810206
O -1.733948 3.478696 -1.266615

P -2.113077 -0.675691 -0.635318

S -1.967218 2.120767 -1.806775

C -1.561103 0.961397 -0.632327

C -1.499018 -1.690042 -2.013787

O -3.280582 1.836970 -2.421216

C -0.434928 -2.578493 -1.836448

H -0.021006 -2.723756 -0.835954

Si -1.764467 -2.394473 1.914138

C -0.436054 -3.057422 -4.202525

H -0.020468 -3.592926 -5.059485

C -1.497363 -2.168174 -4.384239

H -1.911673 -2.006743 -5.382268

C -2.031430 -1.484136 -3.294990

H -2.856026 -0.777097 -3.431836

C -3.917104 -0.905285 -0.702285

C -4.472598 -2.139264 -1.059425

H -3.829467 -2.944738 -1.422423

C -5.848073 -2.340490 -0.963331

H -6.276994 -3.305636 -1.243094

C -6.674185 -1.308976 -0.518164

H -7.753156 -1.466356 -0.446552

C -6.125485 -0.070540 -0.184227

H -6.774108 0.747978 0.136826

C -4.751954 0.132996 -0.279795

H -4.313340 1.105948 -0.063173

C -2.164345 -4.010332 1.030805

H -1.417277 -4.276704 0.268601

H -3.150941 -3.970725 0.546005

H -2.193267 -4.826265 1.771439

C -3.209596 -2.031005 3.068361

H -3.475956 -2.923009 3.657911

H -4.092250 -1.736827 2.477562

H -2.988663 -1.219059 3.776806

C -0.183649 -2.641624 2.902181

H -0.308975 -3.409054 3.682211

H 0.127589 -1.705666 3.387916

H 0.637839 -2.956266 2.239399

C -3.563641 3.148215 1.539272

H -3.451476 3.413785 0.478955

H -3.827756 4.061948 2.096375

H -4.387848 2.432592 1.658394

C -2.282210 2.593653 2.071665
C -1.030117 3.143224 1.664718
H -1.010344 3.889020 0.868612
C 0.171216 2.574905 2.140281
H 1.120709 2.929127 1.733532
C 0.190413 1.525659 3.115529
C -1.050031 1.023722 3.557834
H -1.074469 0.170271 4.237592
C -2.263745 1.515157 3.010605
H -3.206850 1.033030 3.273665
C 0.094846 -3.263576 -2.929778
H 0.931283 -3.952833 -2.788872
C -0.794677 1.935009 -3.149775
H -1.104081 2.659246 -3.915724
H 0.218851 2.158628 -2.796847
H -0.859370 0.911234 -3.539646
C 1.492388 1.004438 3.634119
H 1.825320 1.603849 4.497359
H 1.410413 -0.040568 3.963561
H 2.276561 1.072413 2.866486
P 1.427967 0.042746 0.054367
H 0.864077 -0.245470 -1.212534
C 2.633231 -1.345893 0.106252
C 2.925457 -2.093402 -1.040580
C 3.172519 -1.728199 1.339044
C 3.737582 -3.219761 -0.935961
H 2.514251 -1.812855 -2.011411
C 4.004177 -2.843859 1.411858
H 2.935140 -1.175573 2.249144
C 4.291186 -3.608880 0.282692
H 4.924296 -4.493148 0.352838
C 2.487413 1.467361 -0.436131
C 3.883860 1.415588 -0.414809
C 1.838700 2.670988 -0.751141
C 4.616436 2.564642 -0.713488
H 4.411446 0.491014 -0.175936
C 2.597144 3.797382 -1.055968
H 0.747916 2.744507 -0.749568
C 3.991960 3.765142 -1.040241
H 4.575113 4.655605 -1.273433
Cl 4.056372 -4.170014 -2.349363

Cl 4.667908 -3.310875 2.939490
Cl 6.346213 2.495367 -0.683882
Cl 1.791061 5.276880 -1.446261

Table S47. Cartesian coordinates of **Coord**
Ru -0.341616 0.584268 -1.304155
N -1.560851 1.220083 0.401912
O -0.439415 -3.008247 -1.990979
P -2.185703 -0.278740 0.656377
S -1.208262 -2.750037 -0.751059
C -1.036280 -1.135970 -0.285138
C -2.216957 -0.783263 2.404149
O -2.632713 -3.156045 -0.731486
C -1.536512 -0.058859 3.386262
H -1.070422 0.892327 3.119789
Si -2.318818 2.779877 0.616848
C -2.064244 -1.761757 5.014984
H -2.006367 -2.143946 6.036966
C -2.740670 -2.491160 4.035204
H -3.209910 -3.444755 4.288934
C -2.821008 -2.005808 2.732443
H -3.341178 -2.576931 1.956676
C -3.943080 -0.350515 0.173926
C -4.958207 0.000996 1.070109
H -4.723204 0.177873 2.122432
C -6.274204 0.118097 0.626049
H -7.062885 0.395201 1.329557
C -6.582522 -0.124660 -0.711945
H -7.614524 -0.033710 -1.059472
C -5.576474 -0.505338 -1.601151
H -5.821287 -0.727492 -2.642760
C -4.262680 -0.622274 -1.158574
H -3.472323 -0.960306 -1.825324
C -3.121400 2.933541 2.315870
H -2.385898 2.828304 3.127530
H -3.918086 2.194550 2.480510
H -3.574908 3.934369 2.405247
C -3.658984 3.131906 -0.664039
H -4.197953 4.054861 -0.394456
H -4.391946 2.311774 -0.706856
H -3.251440 3.274750 -1.675654

C -1.000523 4.121644 0.509722
H -1.452162 5.082796 0.216368
H -0.222611 3.869066 -0.223830
H -0.500813 4.279044 1.477870
C -2.086275 -0.967834 -3.965843
H -1.988318 -1.891294 -3.376079
H -1.808432 -1.214356 -5.003015
H -3.128829 -0.625709 -3.965005
C -1.159714 0.079227 -3.443786
C 0.224868 -0.236415 -3.242861
H 0.546659 -1.265361 -3.407898
C 1.153183 0.764419 -2.861864
H 2.210227 0.505408 -2.759492
C 0.739013 2.108573 -2.599291
C -0.650948 2.345094 -2.626440
H -1.022026 3.327298 -2.328764
C -1.586900 1.363373 -3.092177
H -2.649698 1.607344 -3.128979
C -1.464025 -0.545950 4.690714
H -0.938218 0.027375 5.458084
C -0.468619 -3.804065 0.495889
H -0.685592 -4.834013 0.182802
H 0.610850 -3.632215 0.546413
H -0.943310 -3.587013 1.461328
C 1.730412 3.186777 -2.300058
H 2.104875 3.623211 -3.240603
H 1.281605 3.998234 -1.711564
H 2.594770 2.796695 -1.746382
P 1.087349 0.051408 0.336344
H 0.555361 0.047073 1.642497
C 2.430205 1.299577 0.583950
C 2.081404 2.491976 1.223615
C 3.711550 1.137859 0.052525
C 3.015511 3.518638 1.315380
H 1.078032 2.627757 1.629563
C 4.629549 2.183441 0.157334
H 4.006887 0.208110 -0.437638
C 4.298432 3.383025 0.783792
H 5.022901 4.193727 0.857353
C 2.063840 -1.483615 0.296597
C 2.634363 -1.961647 1.481296

C 2.268431 -2.153581 -0.908670
C 3.394187 -3.127528 1.444237
H 2.482311 -1.445882 2.431171
C 3.041097 -3.313636 -0.919083
H 1.782489 -1.817565 -1.820660
C 3.609269 -3.816059 0.249678
H 4.204109 -4.729060 0.232782
Cl 4.086361 -3.741463 2.906119
Cl 3.287741 -4.153195 -2.407409
Cl 6.213835 1.992635 -0.512844
Cl 2.577696 5.003279 2.091375

Table S48. Cartesian coordinates of **TS-Act2**
Ru -0.382447 -0.901540 -1.236034
N -1.422734 0.924990 -0.627657
O -0.686248 -3.689448 1.098916
P -2.176260 0.237924 0.654411
S -1.431912 -2.546663 1.676706
C -1.172316 -1.164941 0.722363
C -2.064234 1.246218 2.161766
O -2.859984 -2.753023 1.989970
C -1.136594 2.289416 2.225169
H -0.579761 2.557900 1.325315
Si -2.024505 2.194756 -1.670491
C -1.632120 2.591430 4.567307
H -1.463280 3.116886 5.510372
C -2.557700 1.547587 4.508617
H -3.111807 1.256601 5.404252
C -2.775854 0.872070 3.309694
H -3.489118 0.043473 3.262432
C -3.964138 0.008661 0.411402
C -4.868102 1.042988 0.681940
H -4.524137 1.958692 1.168376
C -6.210833 0.904963 0.338254
H -6.911928 1.716161 0.547903
C -6.658436 -0.269699 -0.266493
H -7.712234 -0.377698 -0.534586
C -5.765857 -1.314099 -0.508145
H -6.121016 -2.247398 -0.951695
C -4.423528 -1.177311 -0.166375
H -3.722872 -1.997805 -0.306002

C -2.615508 3.674990 -0.664338
H -1.789885 4.119581 -0.088206
H -3.422132 3.426290 0.039453
H -3.000613 4.447562 -1.349740
C -3.457584 1.608968 -2.749738
H -3.890192 2.461964 -3.297595
H -4.257031 1.152462 -2.146765
H -3.133923 0.868853 -3.496954
C -0.656974 2.824494 -2.793431
H -1.092295 3.445920 -3.592352
H -0.094571 2.008222 -3.266414
H 0.058912 3.445797 -2.237663
C -2.440970 -3.715811 -1.703198
H -2.360957 -3.884018 -0.620242
H -2.285904 -4.689771 -2.194345
H -3.449151 -3.365393 -1.957316
C -1.389912 -2.764394 -2.171792
C -0.021797 -2.976098 -1.799881
H 0.216749 -3.768472 -1.090049
C 1.001822 -2.159293 -2.328600
H 2.036053 -2.350768 -2.034684
C 0.714650 -1.077550 -3.221757
C -0.645272 -0.791583 -3.450777
H -0.912241 0.081238 -4.049423
C -1.688614 -1.641913 -2.957803
H -2.730156 -1.400159 -3.175701
C -0.921361 2.962871 3.426164
H -0.188141 3.772270 3.469973
C -0.649411 -2.231521 3.256365
H -0.841641 -3.117177 3.876784
H 0.428076 -2.090496 3.102532
H -1.101142 -1.336785 3.704177
C 1.819882 -0.304595 -3.869789
H 2.126992 -0.803464 -4.803795
H 1.509767 0.716985 -4.130551
H 2.705209 -0.252315 -3.220501
P 1.245489 -0.054167 0.176425
H 0.142790 -0.383942 1.242567
C 1.953571 1.620620 0.043914
C 2.157887 2.352546 1.219983
C 2.233510 2.200506 -1.194327

C 2.621359 3.662731 1.137773
H 1.933210 1.913200 2.193332
C 2.706748 3.509364 -1.248027
H 2.067528 1.643658 -2.113732
C 2.901787 4.260217 -0.090626
H 3.255798 5.289533 -0.144445
C 2.684004 -1.137854 0.449179
C 4.001596 -0.675488 0.364912
C 2.430094 -2.485821 0.753024
C 5.053018 -1.565005 0.583671
H 4.220712 0.366814 0.128835
C 3.503249 -3.344747 0.965486
H 1.407049 -2.872220 0.822786
C 4.825910 -2.904871 0.884293
H 5.655677 -3.591674 1.048643
Cl 3.197571 -5.009368 1.328951
Cl 6.683656 -0.991173 0.467731
Cl 2.825641 4.584670 2.590697
Cl 3.024992 4.230426 -2.789556

Table S49. Cartesian coordinates of **Act’**
Ru -0.342757 0.574739 -1.086358
Si -1.273253 -2.952328 -0.441313
P -2.352681 -0.348532 0.637777
O -3.193308 2.453657 2.150767
S -2.424731 2.638463 0.908820
N -1.338810 -1.200482 -0.287256
H -0.809374 1.168991 1.585383
C -1.427784 1.195954 0.671578
C -2.583460 -1.045150 2.298455
O -3.158822 3.009867 -0.309429
C -1.423669 -1.208267 3.070292
H -0.453016 -0.892689 2.668237
C -1.506046 -1.783708 4.334222
H -0.600322 -1.907851 4.932225
C -3.892176 -2.050846 4.063828
H -4.857859 -2.384671 4.450153
C -3.817479 -1.473638 2.797647
H -4.721546 -1.365867 2.195648
C -4.001747 -0.169821 -0.101303
C -6.286158 0.612220 -0.097610

H -7.087030 1.185060 0.375135
C -5.034525 0.556550 0.508380
H -4.849518 1.098139 1.438225
C -6.517739 -0.051504 -1.302830
H -7.504354 -0.006072 -1.770305
C -0.104071 -3.774105 0.776749
H 0.938114 -3.448240 0.681330
H -0.136457 -4.865704 0.627235
H -0.433972 -3.567320 1.806746
C -2.958444 -3.720877 -0.067393
H -3.292199 -3.490352 0.955904
H -2.852750 -4.816348 -0.134620
H -3.754470 -3.417996 -0.761997
C -4.232051 -0.817462 -1.317280
H -3.408445 -1.354589 -1.791474
C -5.489277 -0.765428 -1.914029
H -5.663626 -1.278265 -2.862925
C -0.831916 -3.396768 -2.213286
H 0.172868 -3.064662 -2.506693
H -1.561464 -2.968711 -2.918825
H -0.856798 -4.491689 -2.333244
C -3.062413 1.668038 -3.102643
H -3.566884 2.045407 -2.203501
H -3.571550 0.763371 -3.455387
H -3.154199 2.434763 -3.889599
C 0.615574 2.261427 -2.126868
H 1.219097 3.050199 -1.676057
C 1.253657 1.089357 -2.625509
C 0.438424 0.051988 -3.139305
H 0.893386 -0.865142 -3.515792
C -0.971944 0.216004 -3.215297
H -1.575505 -0.611615 -3.594596
C -1.615035 1.410363 -2.836644
C -0.792037 2.389073 -2.197563
H -1.282346 3.271579 -1.783914
C -2.739758 -2.204503 4.832334
H -2.802219 -2.657786 5.824584
C 2.744829 1.002383 -2.672297
H 3.087527 1.417457 -3.634300
H 3.209921 1.585693 -1.868566
H 3.103799 -0.032388 -2.605223

C -1.280801 3.964988 1.235573
H -0.639499 4.149008 0.366296
H -1.927672 4.833038 1.420822
H -0.691808 3.737015 2.131792
P 1.276957 0.060653 0.631544
C 2.543976 1.392377 0.675673
C 2.066563 2.701747 0.845509
C 3.932174 1.201379 0.611170
C 2.943255 3.773653 0.920377
H 0.995069 2.876882 0.894758
C 4.792767 2.294375 0.703600
H 4.359092 0.208381 0.469528
C 4.325300 3.598076 0.854155
H 5.010005 4.443090 0.913029
C 2.349639 -1.371350 0.201898
C 3.166316 -1.913726 1.206388
C 2.312050 -1.999160 -1.042826
C 3.934590 -3.044361 0.942338
H 3.192253 -1.465280 2.201616
C 3.080728 -3.133959 -1.282652
H 1.657090 -1.607103 -1.815428
C 3.904998 -3.676507 -0.302151
H 4.502219 -4.567359 -0.493844
Cl 6.503429 2.027382 0.602504
Cl 2.296582 5.380317 1.079121
Cl 4.941902 -3.700337 2.189654
Cl 3.006539 -3.882140 -2.847818

Table S50. Cartesian coordinates of **TS-Act3**
Ru -0.317362 0.341591 -1.160801
Si -1.295858 -3.026064 0.015570
P -2.382515 -0.276106 0.631426
O -3.190006 2.761465 1.672803
S -2.390181 2.724242 0.437488
N -1.365105 -1.275157 -0.125458
H -0.838903 1.339601 1.383690
C -1.427012 1.239211 0.455401
C -2.666676 -0.701076 2.373402
O -3.091334 2.908354 -0.841443
C -1.526120 -0.778378 3.186258
H -0.538179 -0.563445 2.760509

C -1.650244 -1.143224 4.523090
H -0.759528 -1.201557 5.152843
C -4.039992 -1.369443 4.245886
H -5.023361 -1.605342 4.658889
C -3.923639 -1.003113 2.906420
H -4.812876 -0.962651 2.274823
C -4.008336 -0.188653 -0.171471
C -6.269008 0.635542 -0.367124
H -7.064969 1.299372 -0.022434
C -5.034750 0.655032 0.275817
H -4.858351 1.342496 1.105694
C -6.489232 -0.218809 -1.448007
H -7.462269 -0.230483 -1.945076
C -0.097471 -3.615628 1.335377
H 0.924570 -3.242328 1.197342
H -0.062708 -4.717350 1.336140
H -0.449677 -3.289347 2.326291
C -2.975700 -3.718418 0.530921
H -3.289719 -3.334820 1.513584
H -2.879878 -4.812907 0.623889
H -3.780696 -3.512609 -0.189118
C -4.226617 -1.030278 -1.264903
H -3.406410 -1.659825 -1.615436
C -5.466792 -1.051482 -1.898191
H -5.632390 -1.715332 -2.750048
C -0.879241 -3.763307 -1.663636
H 0.133329 -3.518650 -2.011765
H -1.600680 -3.431897 -2.427267
H -0.939952 -4.861921 -1.604665
C -2.975873 1.197533 -3.373098
H -3.486249 1.719643 -2.553718
H -3.504039 0.263011 -3.597011
H -3.029178 1.838170 -4.268875
C 0.709543 1.817746 -2.421673
H 1.335812 2.642254 -2.076636
C 1.310585 0.567984 -2.751056
C 0.464860 -0.509121 -3.104603
H 0.895023 -1.485065 -3.333847
C -0.938593 -0.315506 -3.220138
H -1.566856 -1.170513 -3.479370
C -1.541884 0.943251 -3.039122

C -0.693306 1.978639 -2.534258
H -1.157977 2.928065 -2.264682
C -2.906567 -1.438130 5.054036
H -3.001826 -1.726305 6.103713
C 2.796193 0.425637 -2.804453
H 3.124557 0.633097 -3.836158
H 3.297103 1.142332 -2.143693
H 3.126355 -0.587950 -2.544017
C -1.214212 4.056311 0.560320
H -0.452594 3.969538 -0.223523
H -1.818887 4.961553 0.416000
H -0.763243 4.061919 1.558811
P 1.265737 0.064498 0.649426
C 2.451696 1.473353 0.609391
C 1.955502 2.716585 1.032552
C 3.797974 1.398991 0.224774
C 2.761478 3.846632 1.013361
H 0.933053 2.792852 1.395135
C 4.591770 2.544711 0.227668
H 4.241731 0.452723 -0.084797
C 4.094647 3.788927 0.607068
H 4.724579 4.677559 0.598678
C 2.433089 -1.326329 0.358545
C 3.407429 -1.604177 1.331503
C 2.307042 -2.194824 -0.726173
C 4.237158 -2.711569 1.187897
H 3.516895 -0.961828 2.207646
C 3.143878 -3.300049 -0.846772
H 1.537107 -2.004526 -1.468978
C 4.122863 -3.580385 0.100049
H 4.776164 -4.446621 0.001278
Cl 2.098595 5.372172 1.516913
Cl 6.242893 2.422429 -0.289400
Cl 5.440981 -3.035722 2.391458
Cl 2.965499 -4.339899 -2.226747

Table S51. Cartesian coordinates of **Act**
Ru 0.027392 -1.324083 -0.603620
Si -1.383581 -1.605405 2.729656
P -2.348547 -0.095386 0.264398
O -2.581654 -0.107924 -3.117998

S -1.601737 0.851950 -2.596560
N -1.448817 -1.196139 1.023672
H -0.623813 1.339619 -0.582740
C -1.154258 0.453225 -0.947071
C -2.931660 1.286000 1.295825
O -0.367793 1.071420 -3.365619
C -2.104866 2.398773 1.494496
H -1.153963 2.484938 0.966985
C -2.468762 3.396743 2.394002
H -1.802506 4.248870 2.548242
C -4.500074 2.190265 2.903212
H -5.439333 2.108089 3.454917
C -4.137340 1.190594 2.003622
H -4.793315 0.329710 1.852996
C -3.860100 -0.798312 -0.444369
C -5.991445 -0.593762 -1.561212
H -6.762371 0.028800 -2.020493
C -4.852041 -0.002743 -1.027747
H -4.744775 1.083685 -1.057639
C -6.148872 -1.980027 -1.511561
H -7.044916 -2.441345 -1.933507
C -1.109051 -0.122157 3.850703
H -0.179206 0.388072 3.557104
H -1.008557 -0.459630 4.895395
H -1.936004 0.601752 3.807551
C -3.019968 -2.413485 3.213894
H -3.870318 -1.739886 3.022020
H -3.028509 -2.660338 4.287960
H -3.200904 -3.344009 2.652909
C -4.027839 -2.181256 -0.381625
H -3.252548 -2.775601 0.100861
C -5.170305 -2.773168 -0.916675
H -5.298165 -3.857015 -0.864623
C 0.012950 -2.824136 3.017058
H 0.965511 -2.357550 2.729447
H -0.105554 -3.760007 2.453294
H 0.065275 -3.077459 4.087769
C 2.261974 -0.847764 -3.132704
H 1.693479 -0.018641 -3.574378
H 2.735772 -1.416932 -3.949631
H 3.064371 -0.444978 -2.499077

C 0.970234 -3.345491 -0.494378
H 1.357527 -3.866292 0.382144
C -0.381080 -3.541576 -0.905522
C -0.859339 -2.838252 -2.035126
H -1.898486 -2.934858 -2.352709
C -0.009273 -1.921048 -2.707140
H -0.416501 -1.335007 -3.530325
C 1.353395 -1.738061 -2.346486
C 1.807902 -2.460063 -1.211054
H 2.830374 -2.307411 -0.865898
C -3.665837 3.290801 3.101721
H -3.950622 4.070058 3.812574
C -1.271667 -4.465637 -0.133844
H -2.169118 -4.725990 -0.709604
H -0.740284 -5.394168 0.120281
H -1.588867 -3.993809 0.807248
C -2.379991 2.458799 -2.509819
H -1.625116 3.188474 -2.190651
H -2.714200 2.680127 -3.532113
H -3.231942 2.434007 -1.819874
P 1.360559 0.012461 0.915275
C 1.436639 1.793113 0.397430
C 1.392954 2.740606 1.428634
C 1.435185 2.234799 -0.934004
C 1.324629 4.099257 1.125744
H 1.374775 2.412989 2.469955
C 1.367018 3.599109 -1.209617
H 1.421269 1.519265 -1.757768
C 1.309729 4.550530 -0.190969
H 1.239640 5.613488 -0.420991
C 3.138946 -0.416845 0.715026
C 4.061912 0.316803 -0.042312
C 3.591084 -1.565368 1.388581
C 5.382952 -0.115398 -0.143210
H 3.762510 1.224385 -0.566956
C 4.911420 -1.980526 1.262195
H 2.908337 -2.146614 2.011142
C 5.833733 -1.267767 0.495642
H 6.868172 -1.596903 0.405077
Cl 6.494624 0.795149 -1.114623
Cl 5.431528 -3.420470 2.078847

Cl 1.201735 5.256161 2.414595
Cl 1.304430 4.138084 -2.855747

Table S52. Cartesian coordinates of **TS1**
Ru 0.153845 -1.673704 -0.713407
Si -0.903151 -0.073604 3.336237
P -2.357190 -0.093921 0.532031
O -2.712239 -0.917324 -3.046724
S -1.776924 0.139112 -2.618547
N -1.459045 -0.403752 1.761843
H -0.740940 0.880101 -0.763285
C -1.269096 -0.069212 -0.937217
C -3.121177 1.569745 0.551022
O -0.557018 0.314523 -3.426040
C -2.250862 2.664082 0.664397
H -1.175163 2.497115 0.763852
C -2.746266 3.963097 0.660564
H -2.054561 4.804184 0.748895
C -4.993096 3.102915 0.457507
H -6.069906 3.272960 0.383308
C -4.497713 1.797992 0.459717
H -5.186877 0.953516 0.385469
C -3.767236 -1.248410 0.420456
C -5.628771 -2.344589 -0.667155
H -6.210634 -2.536508 -1.571880
C -4.530742 -1.486829 -0.728467
H -4.245787 -1.045426 -1.684049
C -5.980975 -2.957863 0.534578
H -6.843476 -3.627721 0.575703
C -0.909393 1.764251 3.762304
H -0.230325 2.346520 3.121032
H -0.593930 1.919160 4.807101
H -1.919040 2.189589 3.647628
C -1.983443 -0.944753 4.621429
H -3.016365 -0.560581 4.599473
H -1.592744 -0.791662 5.640563
H -2.021362 -2.031321 4.439069
C -4.124738 -1.866930 1.625072
H -3.514721 -1.679382 2.510707
C -5.227639 -2.715855 1.682800
H -5.496473 -3.192607 2.628521

C 0.847835 -0.743940 3.535072
H 1.541910 -0.262155 2.829431
H 0.872637 -1.824826 3.321056
H 1.224585 -0.589479 4.558838
C 1.510104 -2.421458 -3.591889
H 0.948560 -1.588908 -4.038518
H 1.582478 -3.239945 -4.326823
H 2.525521 -2.072378 -3.360089
C 0.847957 -3.630378 -0.003995
H 1.414123 -3.833431 0.907658
C -0.584902 -3.646594 0.060402
C -1.293033 -3.337428 -1.121773
H -2.382065 -3.276390 -1.096776
C -0.609604 -2.951391 -2.305013
H -1.194409 -2.598943 -3.154627
C 0.813298 -2.893463 -2.356190
C 1.535106 -3.306491 -1.190956
H 2.625254 -3.246601 -1.186085
C -4.120422 4.185268 0.551209
H -4.511224 5.205546 0.546202
C -1.275934 -3.974401 1.343588
H -1.142924 -5.041765 1.584402
H -0.863580 -3.376322 2.168279
H -2.349747 -3.762809 1.278390
C -2.663539 1.681994 -2.743163
H -2.008487 2.507030 -2.436963
H -2.917603 1.770205 -3.807841
H -3.568602 1.648435 -2.124927
P 1.616582 -0.124846 -0.121632
C 1.442248 1.686813 -0.238700
C 1.674198 2.457589 0.909553
C 0.983990 2.301679 -1.412639
C 1.428035 3.827349 0.878799
H 2.020514 1.991511 1.833509
C 0.730101 3.672340 -1.403668
H 0.782364 1.708873 -2.309615
C 0.948637 4.453640 -0.271519
H 0.743969 5.524075 -0.281233
C 3.399392 -0.360215 0.085518
C 4.344538 0.570723 -0.370365
C 3.838219 -1.530226 0.724708

C 5.701109 0.311896 -0.194982
H 4.030956 1.490279 -0.866524
C 5.200019 -1.768033 0.872362
H 3.115110 -2.238933 1.129409
C 6.152424 -0.856438 0.418613
H 7.217572 -1.047057 0.545939
Cl 6.863953 1.461888 -0.762990
Cl 5.726715 -3.224350 1.649172
Cl 0.097193 4.427903 -2.829507
Cl 1.669541 4.769561 2.310120

Table S53. Cartesian coordinates of **Int1**
Ru 0.135447 -1.692374 -0.783527
Si -0.740966 0.078785 3.336651
P -2.335680 -0.066517 0.603353
O -2.827399 -0.983607 -2.946426
S -1.884903 0.088287 -2.575837
N -1.411458 -0.317753 1.825291
H -0.768420 0.861775 -0.776903
C -1.304845 -0.089480 -0.911292
C -3.112272 1.591661 0.579698
O -0.699104 0.260232 -3.433608
C -2.249552 2.697006 0.615722
H -1.169569 2.543925 0.687780
C -2.757544 3.990232 0.565011
H -2.072223 4.840807 0.585727
C -5.001305 3.103057 0.478113
H -6.081596 3.260476 0.431941
C -4.493151 1.803893 0.524462
H -5.175766 0.950967 0.511011
C -3.737383 -1.236113 0.584918
C -5.616778 -2.394280 -0.403527
H -6.224386 -2.625817 -1.281668
C -4.533586 -1.525099 -0.530067
H -4.284755 -1.114415 -1.509476
C -5.921384 -2.969159 0.829922
H -6.772023 -3.649028 0.922364
C -0.690089 1.933187 3.675277
H -0.043728 2.470501 2.965202
H -0.304655 2.130455 4.688937
H -1.698194 2.371670 3.605416

C -1.749597 -0.716991 4.723774
H -2.778867 -0.322816 4.738596
H -1.301790 -0.520766 5.711507
H -1.807072 -1.810378 4.595437
C -4.048852 -1.813675 1.821777
H -3.416412 -1.583047 2.681338
C -5.136400 -2.675185 1.944347
H -5.369255 -3.120538 2.914609
C 1.007502 -0.619656 3.436311
H 1.647159 -0.196337 2.646813
H 0.989527 -1.711013 3.284261
H 1.475279 -0.417017 4.412960
C 1.288899 -2.652589 -3.686716
H 0.725665 -1.825699 -4.141848
H 1.287360 -3.509669 -4.379952
H 2.327285 -2.325984 -3.540484
C 0.823150 -3.618815 0.004710
H 1.439213 -3.790291 0.890010
C -0.601186 -3.594733 0.157435
C -1.370516 -3.336206 -1.000376
H -2.454146 -3.245753 -0.913866
C -0.756949 -3.053663 -2.247932
H -1.385870 -2.740564 -3.081238
C 0.660054 -3.035264 -2.385138
C 1.443828 -3.390081 -1.241482
H 2.533355 -3.361045 -1.305749
C -4.136579 4.195672 0.489936
H -4.537090 5.211295 0.447494
C -1.225721 -3.785606 1.504495
H -2.273384 -4.100007 1.413468
H -0.678522 -4.540329 2.087576
H -1.210638 -2.829671 2.052177
C -2.791717 1.619398 -2.701667
H -2.139816 2.458069 -2.427408
H -3.073783 1.684470 -3.760976
H -3.680459 1.586869 -2.060541
P 1.628929 -0.142097 -0.328540
C 1.451747 1.669781 -0.378968
C 1.791335 2.422883 0.754357
C 0.893092 2.303925 -1.498328
C 1.554403 3.794281 0.763266

H 2.220470 1.944418 1.636173
C 0.645709 3.674559 -1.445982
H 0.618587 1.724538 -2.384977
C 0.973383 4.439054 -0.328854
H 0.777696 5.510940 -0.305199
C 3.398394 -0.380907 -0.029188
C 4.365974 0.539965 -0.457881
C 3.803887 -1.544829 0.640465
C 5.712570 0.278558 -0.221336
H 4.078114 1.454217 -0.978783
C 5.157179 -1.785592 0.850711
H 3.060482 -2.245113 1.020678
C 6.131559 -0.883263 0.427049
H 7.189453 -1.075678 0.603163
Cl -0.115268 4.452508 -2.795194
Cl 1.944987 4.717190 2.173608
Cl 6.903673 1.416307 -0.753441
Cl 5.642786 -3.232510 1.669898

Table S54. Cartesian coordinates of **TS4**
Ru 0.200242 -1.494075 -1.024737
Si -2.627821 -1.667733 3.047292
P -2.416672 -0.001368 0.558866
O -2.840696 -0.465268 -2.862596
S -1.793286 0.491726 -2.477209
N -2.143235 -1.268876 1.444302
H -0.649495 1.085722 -0.605263
C -1.211259 0.169222 -0.832410
C -2.242707 1.566464 1.497993
O -0.602079 0.606562 -3.340061
C -0.946790 2.032156 1.763001
H -0.083717 1.520950 1.332378
C -0.738134 3.125830 2.598513
H 0.278589 3.477960 2.787101
C -3.115116 3.290648 2.965936
H -3.970166 3.772678 3.445963
C -3.323630 2.195541 2.128423
H -4.338468 1.823163 1.980316
C -4.124190 -0.024377 -0.108823
C -6.188603 0.960647 -0.915640
H -6.781968 1.849472 -1.143072

C -4.895816 1.102558 -0.414495
H -4.499903 2.105761 -0.245050
C -6.723766 -0.309327 -1.120089
H -7.735850 -0.419621 -1.516539
C -1.331360 -1.070464 4.277777
H -0.342144 -1.497428 4.047369
H -1.587842 -1.340163 5.314944
H -1.238857 0.026378 4.221442
C -4.299742 -0.949154 3.556965
H -4.245071 0.141118 3.695590
H -4.632471 -1.388642 4.511556
H -5.071959 -1.158647 2.799407
C -4.681081 -1.294273 -0.296338
H -4.090112 -2.171035 -0.022312
C -5.967421 -1.437608 -0.806107
H -6.384427 -2.436232 -0.955626
C -2.791654 -3.544413 3.160285
H -1.850032 -4.060628 2.922679
H -3.561173 -3.905944 2.458683
H -3.094507 -3.850701 4.174523
C -0.572569 -2.402035 -4.212851
H -1.530304 -1.874831 -4.294470
H -0.616064 -3.321375 -4.820968
H 0.211577 -1.748916 -4.617313
C 1.341164 -3.380455 -1.021855
H 2.379221 -3.512806 -0.713231
C 0.297886 -3.454326 -0.054224
C -1.043141 -3.242797 -0.489313
H -1.836761 -3.213982 0.255622
C -1.322566 -2.918807 -1.834719
H -2.337343 -2.643674 -2.125892
C -0.286445 -2.763330 -2.792142
C 1.053396 -2.986585 -2.348098
H 1.874915 -2.815291 -3.046449
C -1.824948 3.763310 3.196535
H -1.663715 4.622257 3.852172
C 0.576966 -3.762092 1.382668
H 0.159096 -4.746327 1.646454
H 1.653886 -3.790916 1.594121
H 0.098619 -3.007821 2.022522
C -2.516219 2.122124 -2.535764

H -1.783269 2.860348 -2.187050
H -2.735592 2.285324 -3.599209
H -3.438616 2.147127 -1.950520
P 1.809155 -0.083542 -0.498131
C 1.829368 1.729319 -0.409555
C 2.426365 2.380752 0.681698
C 1.176640 2.478701 -1.399398
C 2.334427 3.763577 0.784653
H 2.936516 1.813466 1.462147
C 1.092518 3.862048 -1.254486
H 0.733094 1.978937 -2.266297
C 1.662671 4.525459 -0.171232
H 1.584384 5.607567 -0.070458
C 3.505421 -0.535162 -0.036273
C 3.687738 -1.612766 0.838777
C 4.624971 0.130109 -0.556607
C 4.974324 -2.035524 1.158842
H 2.822586 -2.102006 1.284929
C 5.900720 -0.301929 -0.204668
H 4.508874 0.976356 -1.235757
C 6.097696 -1.391073 0.646252
H 7.102801 -1.722205 0.906196
Cl 3.022470 4.561153 2.161248
Cl 0.251968 4.790393 -2.453744
Cl 7.283023 0.517703 -0.846833
Cl 5.176306 -3.387591 2.222964

Table S55. Cartesian coordinates of **Int3**
Ru 0.176350 -1.522298 -0.705216
P -2.235974 -0.046608 0.819717
O -3.075332 -0.560874 -2.350492
S -2.012363 0.440276 -2.168385
N -3.474110 -0.973594 0.772810
H -0.575906 1.083649 -0.499871
C -1.151484 0.158687 -0.646795
C -1.007151 -0.680728 2.010823
O -1.022227 0.575461 -3.247919
C 0.320524 -0.930334 1.641597
H 0.789768 -0.449484 0.719144
C 1.211936 -1.496296 2.560682
H 2.251802 -1.667037 2.281629

C -0.558030 -1.592733 4.203101
H -0.904328 -1.859027 5.204550
C -1.445256 -1.029914 3.289546
H -2.495284 -0.875908 3.551934
C -2.570256 1.640392 1.446130
C -1.787999 3.830737 2.119396
H -0.961603 4.529695 2.267481
C -1.526977 2.555882 1.626344
H -0.497230 2.272931 1.395600
C -3.096681 4.200230 2.433990
H -3.303559 5.202020 2.818048
C -3.874399 2.006635 1.786021
H -4.678431 1.276874 1.673154
C -4.137755 3.287309 2.271062
H -5.160912 3.570590 2.529236
C 1.134322 -2.294929 -3.828550
H 0.619157 -1.403947 -4.213427
H 1.044315 -3.108168 -4.567046
H 2.196760 -2.047528 -3.703071
C 0.678007 -3.644003 -0.264939
H 1.295518 -4.007531 0.560665
C -0.738662 -3.510954 -0.062740
C -1.499684 -2.968913 -1.110023
H -2.556886 -2.753987 -0.961341
C -0.869029 -2.546893 -2.317196
H -1.472828 -2.054585 -3.078207
C 0.517387 -2.723699 -2.539462
C 1.290747 -3.302058 -1.484449
H 2.368926 -3.417359 -1.608430
C 0.770677 -1.823176 3.839763
H 1.469217 -2.258797 4.557679
C -1.377799 -3.896793 1.231497
H -1.661963 -4.961462 1.204446
H -0.690745 -3.751111 2.076666
H -2.281860 -3.294658 1.399782
C -2.809501 2.026645 -2.005314
H -2.050937 2.804322 -1.853445
H -3.331171 2.182008 -2.959054
H -3.525896 1.995000 -1.175201
P 1.912783 -0.031423 -1.520424
C 1.614959 1.699754 -0.943729

C 1.988760 2.180494 0.320869

C 0.946777 2.561335 -1.827467

C 1.702571 3.496469 0.675341

H 2.502705 1.537489 1.037163

C 0.653808 3.864117 -1.433586

H 0.624378 2.196696 -2.804425

C 1.035047 4.360908 -0.189342

H 0.806280 5.385625 0.101468

C 3.453576 -0.360139 -0.553133

C 4.528501 0.540797 -0.659959

C 3.649668 -1.531314 0.184824

C 5.734121 0.272275 -0.022034

H 4.428031 1.459882 -1.240282

C 4.866805 -1.779631 0.814080

H 2.840951 -2.250406 0.289765

C 5.930236 -0.889511 0.729270

H 6.878370 -1.085210 1.228666

Si -5.080521 -1.300702 0.277903

C -5.883603 0.069268 -0.740633

H -5.910100 1.038235 -0.218150

H -5.327020 0.190398 -1.681764

H -6.922979 -0.202128 -0.987772

C -6.107401 -1.572819 1.838450

H -6.127584 -0.667660 2.467037

H -7.149417 -1.840632 1.599905

H -5.676344 -2.385462 2.444945

C -5.150662 -2.873707 -0.755113

H -4.643140 -2.715241 -1.719309

H -4.675565 -3.722565 -0.237693

H -6.195399 -3.153183 -0.966028

Cl 5.031081 -3.227540 1.761905

Cl 7.038086 1.404927 -0.157609

Cl 2.127988 4.062703 2.259288

Cl -0.223208 4.901250 -2.513180

Table S56. Cartesian coordinates of **TS5**

Ru 0.181934 -1.560388 -0.533167

P -2.286624 -0.046113 0.842867

O -2.983107 -0.701501 -2.370312

S -1.934852 0.311884 -2.170800

N -3.475081 -1.039090 0.790876

H -0.589483 1.032471 -0.453883

C -1.154248 0.100349 -0.594772

C -1.001250 -0.503344 2.032994

O -0.888664 0.412136 -3.201267

C 0.173584 -1.089612 1.526167

H 1.094634 -0.543916 0.332906

C 1.132983 -1.521789 2.456328

H 2.073300 -1.957746 2.116272

C -0.265434 -0.831958 4.311349

H -0.433741 -0.736480 5.386292

C -1.224459 -0.386572 3.407130

H -2.161198 0.051672 3.763364

C -2.731365 1.661827 1.333354

C -2.076230 3.932240 1.867607

H -1.288483 4.678038 1.997848

C -1.738630 2.636535 1.489567

H -0.688575 2.378993 1.334612

C -3.413044 4.264368 2.094972

H -3.680551 5.281870 2.390050

C -4.063400 1.993210 1.588295

H -4.830302 1.221890 1.499260

C -4.404288 3.293603 1.961141

H -5.449466 3.546683 2.154306

C 1.207940 -2.562269 -3.717766

H 0.473486 -2.316162 -4.494148

H 1.854164 -3.379594 -4.070708

H 1.838978 -1.667919 -3.557004

C 0.686215 -3.706213 -0.091486

H 1.294563 -4.060609 0.743559

C -0.728244 -3.560509 0.084035

C -1.463150 -3.032320 -0.993068

H -2.517749 -2.794424 -0.863535

C -0.834656 -2.728079 -2.238386

H -1.445162 -2.301735 -3.033198

C 0.537233 -2.928164 -2.438655

C 1.302771 -3.378950 -1.314730

H 2.382929 -3.496665 -1.424161

C 0.914148 -1.397636 3.828211

H 1.681913 -1.744056 4.524738

C -1.400089 -3.887047 1.378143

H -1.768755 -4.925234 1.353064

H -0.708716 -3.785832 2.225831
H -2.255953 -3.213485 1.534325
C -2.749132 1.897744 -2.112471
H -2.004789 2.686010 -1.945516
H -3.214794 2.013007 -3.100269
H -3.511989 1.891782 -1.324220
P 1.898793 -0.092020 -1.385877
C 1.586065 1.673185 -0.928884
C 1.853253 2.194581 0.344954
C 1.009096 2.492703 -1.905976
C 1.544430 3.523667 0.620678
H 2.289398 1.571299 1.127983
C 0.692304 3.813373 -1.592209
H 0.760821 2.084237 -2.886789
C 0.962690 4.352549 -0.337583
H 0.711469 5.387430 -0.106605
C 3.488655 -0.330377 -0.472143
C 4.500184 0.636940 -0.579001
C 3.771013 -1.518276 0.207426
C 5.742439 0.411023 0.006211
H 4.327443 1.573765 -1.111479
C 5.019436 -1.720011 0.787759
H 3.008398 -2.287818 0.304564
C 6.026824 -0.764898 0.702561
H 7.001108 -0.925259 1.162836
Si -5.066876 -1.423159 0.295213
C -5.863890 -0.154054 -0.852694
H -5.952071 0.846834 -0.402347
H -5.262009 -0.075399 -1.770421
H -6.878779 -0.481475 -1.132076
C -6.136373 -1.599282 1.840688
H -6.189028 -0.653015 2.403228
H -7.166478 -1.901203 1.591210
H -5.710654 -2.358286 2.516286
C -5.089679 -3.071017 -0.620209
H -4.584891 -2.975117 -1.594371
H -4.593872 -3.866284 -0.040860
H -6.126057 -3.392733 -0.811443
Cl 5.304188 -3.191857 1.661886
Cl 6.973110 1.621960 -0.124456
Cl 1.825976 4.152693 2.210647

Cl -0.082996 4.806207 -2.781952

Table S57. Cartesian coordinates of **Int4**

Ru 0.156805 -1.607534 -0.437866
P -2.364935 -0.055005 0.819549
O -2.890606 -0.705458 -2.440769
S -1.846751 0.296794 -2.174871
N -3.568435 -1.023750 0.693104
H -0.579507 0.990847 -0.386909
C -1.154260 0.069929 -0.560332
C -1.137678 -0.510050 2.063012
O -0.744489 0.394291 -3.148605
C 0.035533 -1.115918 1.581117
H 1.309593 -0.544341 0.130164
C 0.997274 -1.484248 2.536991
H 1.938645 -1.943012 2.227066
C -0.401709 -0.694227 4.354356
H -0.566938 -0.536498 5.422493
C -1.363776 -0.307473 3.428054
H -2.296579 0.158555 3.758709
C -2.806373 1.667792 1.265245
C -2.138824 3.932437 1.810463
H -1.345665 4.664088 1.982175
C -1.805191 2.624498 1.473380
H -0.753019 2.341619 1.393410
C -3.480204 4.295552 1.945603
H -3.744445 5.322642 2.208724
C -4.144332 2.030583 1.428812
H -4.920298 1.273944 1.299894
C -4.480984 3.343172 1.761226
H -5.530842 3.620306 1.882899
C 1.298813 -2.625356 -3.577025
H 2.347541 -2.359886 -3.383028
H 0.812515 -1.766901 -4.061158
H 1.283193 -3.481832 -4.270040
C 0.629890 -3.742879 0.046201
H 1.199621 -4.096774 0.907712
C -0.786555 -3.564300 0.161924
C -1.470526 -3.037046 -0.953368
H -2.525166 -2.780196 -0.862363
C -0.793642 -2.764498 -2.181070

H -1.366496 -2.337451 -3.003269
C 0.584957 -2.970538 -2.312181
C 1.295519 -3.427965 -1.154942
H 2.379629 -3.546060 -1.215304
C 0.781790 -1.277827 3.899869
H 1.553300 -1.576033 4.614943
C -1.521151 -3.866960 1.427176
H -1.901436 -4.900776 1.392225
H -0.868794 -3.764715 2.304808
H -2.374985 -3.181726 1.536032
C -2.648838 1.890065 -2.157506
H -1.911032 2.670800 -1.935459
H -3.048828 2.016520 -3.172322
H -3.460534 1.884748 -1.419810
P 1.922187 -0.097589 -1.315215
C 1.619622 1.666918 -0.851774
C 1.856438 2.158769 0.438715
C 1.081919 2.504439 -1.833655
C 1.548598 3.484788 0.729040
H 2.258883 1.513529 1.222146
C 0.772585 3.824532 -1.507622
H 0.852532 2.111510 -2.825283
C 1.007083 4.335465 -0.234518
H 0.757016 5.368116 0.007819
C 3.554466 -0.329287 -0.472037
C 4.580971 0.597031 -0.699098
C 3.824695 -1.472009 0.283494
C 5.843414 0.372633 -0.156485
H 4.405526 1.499625 -1.287620
C 5.094871 -1.673538 0.815735
H 3.038847 -2.200800 0.477883
C 6.124194 -0.760473 0.608511
H 7.115434 -0.920996 1.031195
Si -5.132919 -1.385176 0.106016
C -5.843517 -0.110846 -1.092870
H -5.944750 0.893026 -0.651852
H -5.187954 -0.043988 -1.974045
H -6.844627 -0.425905 -1.430251
C -6.297639 -1.537919 1.583714
H -6.368777 -0.588348 2.138631
H -7.315411 -1.824476 1.272915

H -5.925885 -2.300371 2.286688

C -5.123122 -3.036935 -0.803136

H -4.555372 -2.952642 -1.743151

H -4.675748 -3.835424 -0.189690

H -6.149423 -3.345930 -1.058820

Cl 5.384557 -3.086729 1.777236

Cl 7.101571 1.528729 -0.430913

Cl 1.780541 4.083535 2.337291

Cl 0.045968 4.845941 -2.702449

Table S58. Cartesian coordinates of **TS6**

Ru 0.063618 -1.711217 -0.296922

P -2.504393 -0.071191 0.764462

O -2.756770 -0.746559 -2.561927

S -1.747058 0.253375 -2.183474

N -3.738663 -0.978247 0.538230

H -0.611248 0.909573 -0.306503

C -1.195451 0.001598 -0.520105

C -1.403573 -0.579367 2.103367

O -0.563883 0.376792 -3.056246

C -0.229598 -1.243100 1.703500

H 1.419225 -0.354286 0.733101

C 0.641419 -1.653463 2.729085

H 1.578508 -2.161878 2.481478

C -0.843373 -0.790700 4.440706

H -1.077681 -0.622446 5.494319

C -1.717294 -0.359045 3.447576

H -2.645935 0.155094 3.712370

C -2.899477 1.675226 1.169360

C -2.186082 3.931906 1.693655

H -1.379473 4.645913 1.877558

C -1.878521 2.608267 1.394211

H -0.830725 2.298324 1.352834

C -3.520732 4.334886 1.774569

H -3.764195 5.374174 2.008229

C -4.231090 2.080085 1.274856

H -5.021978 1.341590 1.126952

C -4.541339 3.408002 1.570979

H -5.586356 3.717800 1.647534

C 1.729806 -2.541736 -3.213737

H 2.692200 -2.192935 -2.811272

H 1.284464 -1.726125 -3.799349
H 1.932885 -3.391767 -3.885011
C 0.473755 -3.835242 0.189107
H 0.900782 -4.219277 1.117423
C -0.939125 -3.608861 0.103333
C -1.430661 -3.036279 -1.099857
H -2.482404 -2.759021 -1.165344
C -0.575944 -2.771060 -2.216373
H -1.006577 -2.317599 -3.108402
C 0.806167 -2.949960 -2.111818
C 1.321217 -3.468013 -0.871839
H 2.401684 -3.579286 -0.756669
C 0.340413 -1.432868 4.074054
H 1.040744 -1.766475 4.844865
C -1.864909 -3.929664 1.231069
H -2.281261 -4.941102 1.094834
H -1.343551 -3.894050 2.197294
H -2.694512 -3.206834 1.251232
C -2.553753 1.844705 -2.213625
H -1.846420 2.622147 -1.900147
H -2.847972 1.993328 -3.261060
H -3.436240 1.821742 -1.563035
P 1.913747 -0.040758 -0.591235
C 1.668185 1.771600 -0.464108
C 1.886284 2.443987 0.745729
C 1.165935 2.456592 -1.574841
C 1.584549 3.800597 0.831461
H 2.262730 1.915135 1.622978
C 0.868313 3.813824 -1.453307
H 0.936734 1.920526 -2.498425
C 1.073969 4.501553 -0.260694
H 0.825399 5.559512 -0.177195
C 3.671440 -0.305309 -0.171020
C 4.655075 0.588182 -0.605699
C 4.038547 -1.465625 0.518862
C 5.994364 0.310764 -0.335372
H 4.392340 1.500304 -1.144303
C 5.383291 -1.719540 0.767886
H 3.280357 -2.165170 0.874573
C 6.380825 -0.841074 0.347589
H 7.431558 -1.047193 0.548890

Si -5.236860 -1.329356 -0.201396
C -5.823230 -0.021365 -1.431039
H -5.921076 0.978834 -0.980288
H -5.106250 0.034689 -2.263949
H -6.807706 -0.297084 -1.843342
C -6.535643 -1.499322 1.157957
H -6.654921 -0.556427 1.716141
H -7.520909 -1.777492 0.749951
H -6.232999 -2.272490 1.882033
C -5.148121 -2.961281 -1.139806
H -4.499834 -2.853710 -2.023507
H -4.752151 -3.771059 -0.506489
H -6.147142 -3.266278 -1.490787
Cl 5.825344 -3.157111 1.624891
Cl 7.210021 1.421269 -0.863583
Cl 1.789209 4.628670 2.336907
Cl 0.186686 4.656204 -2.802005

Table S59. Cartesian coordinates of **3’**
Ru 0.286700 -1.439229 0.907724
O -1.334060 -2.048343 -2.390936
S -0.675046 -0.755386 -2.155047
P -2.523427 -0.084433 0.202775
N -3.512477 -1.275957 0.080880
C -0.829604 -0.244955 -0.478205
H -0.423571 0.775067 -0.497332
P 1.864301 0.187454 0.980009
C -3.190981 1.507580 -0.420516
O 0.750232 -0.636329 -2.531636
C -2.401370 2.663711 -0.424020
H -1.385317 2.631769 -0.025867
C -2.906472 3.861376 -0.919244
H -2.277792 4.754754 -0.921748
Si -4.625709 -2.158148 -0.869451
C -5.015162 2.778949 -1.379101
H -6.044131 2.823115 -1.743962
C -4.505567 1.577753 -0.884900
H -5.129121 0.682665 -0.851208
C -1.962971 0.316358 1.877055
C -0.696551 -0.192210 2.229466
C -0.247383 0.089539 3.531644

H 0.726008 -0.285878 3.865107

C -1.016430 0.836713 4.425630

H -0.634212 1.036953 5.430690

C -2.267332 1.327708 4.048041

H -2.866294 1.913863 4.748623

C -2.741908 1.061218 2.767132

H -3.719559 1.436907 2.451086

C -4.681205 -1.645244 -2.685821

H -4.957461 -0.588581 -2.827190

H -5.415770 -2.257138 -3.234623

H -3.690004 -1.814218 -3.133834

H 2.328933 0.492687 2.288720

C -4.207839 -3.997223 -0.845116

H -3.282489 -4.192922 -1.409664

H -5.014396 -4.580674 -1.317761

H -4.079308 -4.373107 0.182402

C -6.349857 -1.956875 -0.124637

H -6.347463 -2.273878 0.930487

H -7.096612 -2.562806 -0.663000

H -6.687506 -0.908117 -0.149629

C -2.118190 -3.079543 2.639879

H -1.977826 -2.496065 3.560229

H -2.440748 -4.096112 2.917628

H -2.909255 -2.606714 2.038337

C -0.853759 -3.147358 1.846084

C -0.880233 -3.291729 0.445481

H -1.836089 -3.281003 -0.074746

C 0.324769 -3.373970 -0.318891

H 0.246164 -3.468960 -1.401707

C 1.583554 -3.298970 0.294924

C 1.614160 -3.051847 1.706971

H 2.578445 -2.920781 2.204216

C 0.429441 -2.998103 2.468342

H 0.483686 -2.811538 3.542291

C 3.484873 -0.078873 0.175699

C 3.519924 -0.455684 -1.170128

H 2.592848 -0.602047 -1.735441

C 4.756182 -0.682126 -1.769810

C 5.949525 -0.552487 -1.060264

H 6.908556 -0.744365 -1.541375

C 5.887566 -0.182139 0.281479

C 4.666708 0.060142 0.909274
H 4.652606 0.355589 1.960022
C 1.430342 1.898324 0.471949
C 1.404810 2.247307 -0.883210
H 1.646134 1.519929 -1.661395
C 1.003277 3.531490 -1.241545
C 0.650003 4.481189 -0.284117
H 0.349263 5.486448 -0.578754
C 0.663179 4.105046 1.057147
C 1.042178 2.820499 1.447157
H 1.011445 2.547708 2.503291
C -4.214101 3.918834 -1.405095
H -4.611314 4.858560 -1.796308
C 2.845344 -3.498847 -0.480997
H 3.690167 -2.958626 -0.033691
H 3.100719 -4.571513 -0.488003
H 2.732630 -3.172339 -1.523029
C -1.524257 0.455308 -3.153973
H -2.593445 0.452619 -2.910649
H -1.078865 1.443847 -2.984629
H -1.362217 0.130472 -4.190363
Cl 0.174783 5.248235 2.257207
Cl 0.893323 3.947436 -2.917558
Cl 4.814038 -1.181529 -3.424048
Cl 7.355261 -0.023003 1.184171

Table S60. Cartesian coordinates of **TS2**
Ru 0.530189 -1.094188 1.758097
Si -0.727948 0.807786 -3.027686
P 1.470960 0.038430 -1.102362
O 4.141266 -0.626638 1.148517
S 3.310673 0.563975 1.404711
N -0.040008 0.383063 -1.482427
H 1.272832 1.389435 0.793234
C 1.700178 0.378404 0.686486
C 2.597280 1.131001 -2.031272
O 3.103864 0.985578 2.797609
C 2.323284 2.504902 -1.957603
H 1.458397 2.851461 -1.385024
C 3.140750 3.420165 -2.610861
H 2.920158 4.488345 -2.546761

C 4.506385 1.608886 -3.437932
H 5.357242 1.254592 -4.024489
C 3.688415 0.686976 -2.783623
H 3.899793 -0.380758 -2.864680
C 1.932188 -1.666264 -1.543964
C 3.495529 -3.498580 -1.746125
H 4.471792 -3.935157 -1.521702
C 3.183436 -2.230199 -1.259352
H 3.894470 -1.686457 -0.632879
C 2.572418 -4.209318 -2.515509
H 2.828019 -5.199494 -2.900462
C -0.904244 2.667757 -3.284994
H -1.405740 3.187694 -2.457951
H -1.494324 2.851071 -4.198017
H 0.081971 3.133478 -3.432644
C 0.290651 0.205541 -4.501501
H 1.296000 0.654372 -4.501531
H -0.211759 0.515999 -5.432652
H 0.408942 -0.887360 -4.532855
C 0.998030 -2.396022 -2.286146
H 0.013975 -1.960910 -2.469255
C 1.317745 -3.660762 -2.775658
H 0.578885 -4.220575 -3.353765
C -2.433654 0.016902 -3.154901
H -3.106589 0.315161 -2.336399
H -2.366160 -1.082132 -3.131441
H -2.911198 0.305164 -4.105124
C 1.298521 -0.584860 5.036282
H 2.079519 0.137154 4.761721
H 1.621309 -1.131314 5.938486
H 0.381078 -0.032805 5.284880
C -0.447465 -2.920335 2.503892
H -1.463357 -3.243694 2.266880
C 0.633664 -3.350108 1.681060
C 1.926385 -2.845896 1.982058
H 2.775104 -3.105602 1.349448
C 2.128071 -1.969740 3.079694
H 3.121961 -1.551893 3.236000
C 1.063565 -1.531358 3.902548
C -0.239851 -2.010933 3.571960
H -1.099528 -1.650244 4.139704

C 4.238261 2.973511 -3.349932
H 4.880878 3.692016 -3.863997
C 0.429575 -4.363263 0.601781
H 1.177486 -4.253487 -0.191773
H 0.531440 -5.377183 1.024187
H -0.571718 -4.295010 0.154615
C 4.180815 1.936705 0.660806
H 3.560580 2.838969 0.719306
H 5.071503 2.046636 1.293781
H 4.462447 1.707427 -0.372010
P -1.068548 -0.016541 0.799393
C -1.338464 1.781199 0.844728
C -2.201959 2.454330 -0.025993
C -0.647193 2.496559 1.832260
C -2.362414 3.830673 0.101351
H -2.741606 1.920838 -0.807991
C -0.799621 3.878017 1.912367
H 0.008350 1.965549 2.528632
C -1.661951 4.564116 1.058208
H -1.790102 5.643265 1.139554
C -2.647496 -0.751177 0.307577
C -3.915603 -0.206045 0.540092
C -2.525021 -1.984929 -0.343511
C -5.041937 -0.904470 0.109457
H -4.037872 0.748779 1.053041
C -3.665150 -2.657421 -0.762771
H -1.530615 -2.391210 -0.536886
C -4.937764 -2.131063 -0.547824
H -5.828562 -2.659117 -0.887171
Cl -6.613715 -0.238948 0.396794
Cl -3.493579 -4.166908 -1.597428
Cl -3.432790 4.662294 -0.977343
Cl 0.088784 4.764145 3.104663

Table S61. Cartesian coordinates of **Int2**
Ru 0.499740 0.466727 -1.754078
Si -0.426264 -0.631372 3.025649
P 1.696852 -0.244427 0.733477
O 4.102987 -0.324014 -1.745153
S 3.178359 -1.470471 -1.706671
N 0.108664 -0.291901 1.326920

H 1.169233 -2.021524 -0.730522
C 1.700316 -1.060897 -0.843751
C 2.576769 -1.337529 1.928681
O 2.783312 -2.092905 -2.972507
C 2.162148 -2.674636 2.028796
H 1.274321 -3.011137 1.487290
C 2.868667 -3.578561 2.814371
H 2.527517 -4.613675 2.892792
C 4.444237 -1.842722 3.388550
H 5.340796 -1.513294 3.918907
C 3.728930 -0.931739 2.610888
H 4.065029 0.104824 2.542625
C 2.460317 1.371341 0.962082
C 4.296912 2.926736 0.756106
H 5.276221 3.175476 0.340066
C 3.723030 1.696285 0.446303
H 4.231166 0.997116 -0.220022
C 3.633811 3.841001 1.574670
H 4.089495 4.806952 1.803761
C -0.954894 -2.429473 3.235933
H -1.066123 -2.943432 2.271254
H -1.923487 -2.498088 3.755517
H -0.209126 -2.977261 3.831412
C 0.894460 -0.234829 4.302431
H 1.632159 -1.039792 4.415256
H 0.371532 -0.116844 5.266486
H 1.433982 0.701194 4.099362
C 1.810520 2.277727 1.814650
H 0.840840 2.019562 2.240330
C 2.385336 3.511212 2.102666
H 1.850516 4.216412 2.743070
C -1.844500 0.538834 3.415556
H -2.727143 0.459839 2.768530
H -1.508410 1.586421 3.375525
H -2.166169 0.328221 4.448821
C -1.833974 -0.175585 -4.184428
H -1.596330 -1.233950 -4.362441
H -2.209652 0.249957 -5.130112
H -2.651197 -0.127596 -3.449786
C 0.330385 2.540789 -2.488064
H 0.162242 3.453059 -1.910810

C 1.652941 2.146009 -2.829168
C 1.792233 0.929618 -3.547275
H 2.796620 0.547119 -3.730325
C 0.682409 0.166884 -4.010757
H 0.864792 -0.765253 -4.546298
C -0.632110 0.579570 -3.705042
C -0.784129 1.776629 -2.931671
H -1.788470 2.090621 -2.636121
C 4.014470 -3.163848 3.497101
H 4.573117 -3.873855 4.111303
C 2.844796 2.973090 -2.475120
H 3.722826 2.332274 -2.318130
H 3.081109 3.679576 -3.289293
H 2.670132 3.554942 -1.559665
C 4.029877 -2.744597 -0.785513
H 3.366131 -3.608321 -0.659058
H 4.889718 -3.012664 -1.413870
H 4.361721 -2.353119 0.183469
P -0.869298 0.062366 -0.112634
C -1.971072 -1.433202 -0.167454
C -2.945678 -1.778359 0.774127
C -1.732348 -2.276772 -1.255171
C -3.656612 -2.965946 0.622855
H -3.161269 -1.148293 1.636111
C -2.460966 -3.456414 -1.389841
H -0.970013 -1.980644 -1.985678
C -3.429337 -3.819696 -0.456100
H -3.996131 -4.744217 -0.565912
C -2.018109 1.427318 0.323657
C -3.407808 1.355324 0.208980
C -1.407662 2.652440 0.613871
C -4.171840 2.503363 0.428745
H -3.910717 0.425474 -0.056832
C -2.191447 3.774606 0.846488
H -0.319923 2.728467 0.619167
C -3.583588 3.719868 0.763576
H -4.192054 4.607142 0.937116
Cl -4.836695 -3.401349 1.814543
Cl -2.163673 -4.488823 -2.747463
Cl -5.894586 2.411988 0.282570
Cl -1.421097 5.280278 1.226445

Table S62. Cartesian coordinates of **TS3**

Ru -0.736769 -0.747043 -1.363677
Si 0.631942 0.262781 3.187026
P -1.687260 0.201282 0.518604
O -4.406764 -0.323461 -1.645549
S -3.588045 0.846674 -2.014528
N 0.088365 -0.062027 1.529624
H -1.715571 1.910223 -1.250455
C -2.099806 0.889789 -1.125254
C -1.858786 1.804070 1.435673
O -3.343986 1.077880 -3.446459
C -1.169202 2.949742 1.011900
H -0.465208 2.892807 0.179759
C -1.354807 4.169989 1.651101
H -0.802272 5.047206 1.305926
C -2.909170 3.138932 3.170568
H -3.591805 3.201299 4.021679
C -2.725661 1.911927 2.527061
H -3.269882 1.036258 2.885174
C -2.865546 -0.929299 1.311806
C -5.088378 -1.699137 1.866139
H -6.170845 -1.557871 1.827161
C -4.251821 -0.749168 1.282428
H -4.687388 0.113859 0.782013
C -4.551098 -2.823229 2.490589
H -5.211917 -3.561648 2.950900
C 0.778793 2.084362 3.633205
H 1.400218 2.657082 2.930261
H 1.241250 2.163349 4.631160
H -0.206374 2.569249 3.679807
C -0.602617 -0.519724 4.366129
H -1.626631 -0.164411 4.183449
H -0.332863 -0.254828 5.401255
H -0.606973 -1.616978 4.287025
C -2.331114 -2.068070 1.925364
H -1.247907 -2.201136 1.923700
C -3.168171 -3.005944 2.521141
H -2.740068 -3.886363 3.006380
C 2.310529 -0.540286 3.515739
H 3.103570 -0.229368 2.818838

H 2.253348 -1.638076 3.475067

H 2.632858 -0.257914 4.531600

C 1.533066 -0.863543 -4.005023

H 1.270745 0.091312 -4.481127

H 1.888025 -1.549215 -4.792233

H 2.372861 -0.689266 -3.316822

C -0.530883 -2.962225 -1.496704

H -0.323541 -3.666676 -0.689183

C -1.871714 -2.682791 -1.887213

C -2.053553 -1.753704 -2.935354

H -3.068645 -1.468301 -3.206076

C -0.966938 -1.132291 -3.621325

H -1.192457 -0.384283 -4.382479

C 0.357100 -1.450442 -3.289159

C 0.543326 -2.353491 -2.193036

H 1.563065 -2.568897 -1.870277

C -2.230129 4.272193 2.734142

H -2.374872 5.230708 3.237613

C -3.050697 -3.329132 -1.236264

H -3.875114 -2.606519 -1.158146

H -3.393259 -4.183552 -1.842559

H -2.803885 -3.697287 -0.232301

C -4.469466 2.288642 -1.423623

H -3.899529 3.187566 -1.688532

H -5.437805 2.280294 -1.941155

H -4.601058 2.218714 -0.336205

P 0.893451 -0.095168 0.070492

C 1.778225 1.484166 -0.271925

C 2.695621 2.052712 0.615583

C 1.442979 2.143052 -1.456289

C 3.245811 3.296489 0.318435

H 2.986247 1.545114 1.535816

C 2.020494 3.379836 -1.739438

H 0.713992 1.682434 -2.131634

C 2.917815 3.975910 -0.855967

H 3.355530 4.949607 -1.076003

C 2.288481 -1.275426 0.218868

C 3.518906 -1.070733 -0.407663

C 2.031020 -2.475977 0.886201

C 4.483144 -2.078643 -0.360403

H 3.737689 -0.139090 -0.931906

C 3.009910 -3.461598 0.922622
H 1.067861 -2.633417 1.373428
C 4.246946 -3.280888 0.302396
H 5.008775 -4.059357 0.336463
Cl 4.346401 4.027427 1.436287
Cl 1.603810 4.198027 -3.206055
Cl 6.006249 -1.831569 -1.142889
Cl 2.691078 -4.942577 1.760659

Table S63. Cartesian coordinates of **4'**
C 4.061456 0.047926 -0.347979
P -0.943967 0.081715 0.337257
O 3.236215 -3.309617 0.300589
Ru 0.649284 -1.076629 -1.016502
Si -1.566489 -0.317550 3.392066
P 2.277158 0.105591 0.028990
O 1.333916 -3.650535 1.912682
S 2.372900 -2.769131 1.364453
C 1.586749 -1.287016 0.891280
H 0.865252 -0.997771 1.687590
C -1.386079 2.791844 0.846802
H -1.540455 2.453890 1.872718
C -1.455476 4.141188 0.511879
C -1.217239 4.586272 -0.788019
H -1.264166 5.646941 -1.034082
C -0.887701 3.638757 -1.754804
C -0.805256 2.285938 -1.443771
H -0.500099 1.563172 -2.199387
C -1.064934 1.862841 -0.141012
C -3.467900 0.116212 -1.055519
H -3.199748 1.075287 -1.501373
C -4.686622 -0.479612 -1.387555
C -5.080726 -1.695840 -0.835268
H -6.034424 -2.150073 -1.102804
C -4.219216 -2.314578 0.072609
C -3.002407 -1.742548 0.421195
H -2.336430 -2.247606 1.122901
C 2.036237 2.852708 0.101089
H 2.167608 2.789569 -0.982678
C 4.620127 -1.051213 -1.015380
H 3.998555 -1.910416 -1.256957

C 6.802394 -0.013376 -0.936134
H 7.870008 -0.037388 -1.167068
C 5.981966 -1.080055 -1.301013
H 6.404507 -1.950246 -1.808520
C 6.255145 1.079350 -0.267370
H 6.890314 1.916277 0.031929
C 4.894218 1.111850 0.029660
H 4.480565 1.970204 0.561139
C 2.039465 1.685446 0.877884
C 1.853876 4.091870 0.705484
H 1.846086 4.997384 0.095184
C 1.656127 4.171195 2.084881
H 1.489115 5.142004 2.556247
C 1.646487 3.011054 2.855985
H 1.477874 3.068624 3.933432
C 1.839205 1.767318 2.257163
H 1.808793 0.858935 2.858656
C -0.591844 0.557377 4.753476
H -1.059575 0.407546 5.739794
H -0.539887 1.640731 4.561644
H 0.439667 0.172799 4.811799
C -1.584800 -2.162484 3.801370
H -1.746655 -2.328700 4.878947
H -0.630135 -2.634179 3.515982
H -2.388254 -2.696035 3.268428
C -3.341306 0.329176 3.440167
H -3.954728 -0.144983 2.657766
H -3.378633 1.418609 3.277967
H -3.813852 0.117691 4.413356
C -0.052318 -3.134906 -1.671137
C -0.963467 -2.175421 -2.155716
H -2.015653 -2.270540 -1.891236
C -0.547469 -1.042129 -2.913844
H -1.295765 -0.312757 -3.229193
C 0.813386 -0.854011 -3.244817
C 1.734158 -1.860910 -2.812604
H 2.793192 -1.733403 -3.041548
C 1.323911 -2.952635 -2.026246
H 2.072159 -3.638466 -1.626574
N -0.785509 -0.010344 1.905796
C -2.620414 -0.526153 -0.153596

C -0.489866 -4.305394 -0.849161
H -0.414672 -5.232626 -1.439706
H 0.142548 -4.408869 0.044885
H -1.532774 -4.192376 -0.523814
C 1.287081 0.302031 -4.070227
H 2.285231 0.629817 -3.747462
H 1.354878 0.007631 -5.130308
H 0.606229 1.161732 -4.007357
C 3.452895 -2.319641 2.711666
H 2.850552 -1.871126 3.511708
H 3.916578 -3.254175 3.054731
H 4.212997 -1.621722 2.336858
Cl -5.736136 0.311894 -2.516105
Cl -4.669280 -3.836925 0.767339
Cl -0.526464 4.161922 -3.371434
Cl -1.804019 5.308257 1.744516

4.2.4 2a’ + $HPAr^F_2$

Table S64. SCF energies, enthalpy and free energy corrections and barriers

	E_{SCF}/E_H	corrH/E_H	corrG/E_H	ΔH/kJ/mol	ΔG/kJ/mol
**TS-Act1**	-4451.224616	0.793237	0.625456	-38.02490646	43.49949404
**Coord**	-4451.25383	0.79477	0.630203	-110.6996654	-20.73690789
**TS-Act2**	-4451.228832	0.791546	0.625407	-53.53449636	32.30097515
**Act'**	-4451.274725	0.796891	0.631686	-159.9907761	-71.70308763
**TS-Act3**	-4451.274007	0.795841	0.635444	-160.86278	-59.95169095
**Act**	-4451.27935	0.796552	0.631118	-173.02436	-85.33791299
**TS1**[a]	-	-	-	-	-
**Int1**	-4451.253690	0.796773	0.62787	-105.074112	-26.4955221
**TS4**	-4451.240109	0.795683	0.628445	-72.27868	10.671371
**Int3**	-4451.257886	0.795859	0.630077	-118.49092	-31.7181404
**TS5**	-4451.245311	0.791146	0.625112	-97.84921	-11.73805915
**Int4**	-4451.247537	0.793042	0.625904	-98.71481	-15.50221224
**TS6**	-4451.235786	0.791954	0.625237	-70.72091	13.59701816
**3‘**	-4451.287523	0.794771	0.632166	-199.15864	-104.044653
**TS2**	-4451.245236	0.795404	0.632601	-86.47281	8.121327874
**Int2**	-4451.261845	0.796154	0.630557	-128.1107999	-40.85230741
**TS3**	-4451.247556	0.794959	0.631161	-93.7316365	-1.749869495
**4‘**	-4451.291588	0.796425	0.629636	-205.4903498	-121.3614533

[a] Transition state not found

Table S65. Cartesian coordinates of **TS-Act1**

Ru 1.688173 0.701872 -1.385792

N 1.910588 -1.355836 -0.688401
O 2.484418 3.351248 0.926128
P 2.526453 -0.867051 0.736093
S 2.615186 2.045437 1.610695
C 2.126186 0.805086 0.555310
C 1.767794 -1.682391 2.173393
O 3.900984 1.733041 2.266220
C 0.629021 -2.480472 2.029510
H 0.243807 -2.685499 1.027743
Si 2.105690 -2.792003 -1.649165
C 0.492103 -2.723992 4.427209
H -0.006740 -3.132726 5.309164
C 1.628511 -1.926285 4.575563
H 2.019071 -1.709457 5.572648
C 2.269117 -1.405981 3.453593
H 3.156999 -0.774966 3.563568
C 4.296576 -1.251294 0.901770
C 4.721399 -2.484243 1.410221
H 3.994183 -3.184545 1.828388
C 6.074132 -2.818017 1.393050
H 6.401175 -3.781511 1.791048
C 7.007281 -1.920343 0.875316
H 8.068296 -2.181459 0.865631
C 6.588708 -0.681705 0.388629
H 7.321373 0.034453 0.009170
C 5.238297 -0.345436 0.405220
H 4.900926 0.633706 0.068710
C 2.346392 -4.336406 -0.597585
H 1.568072 -4.456769 0.170400
H 3.325851 -4.333187 -0.096942
H 2.312422 -5.223524 -1.251010
C 3.605723 -2.664431 -2.783086
H 3.819537 -3.635536 -3.257879
H 4.491456 -2.369809 -2.197183
H 3.467720 -1.926655 -3.587186
C 0.537944 -3.008498 -2.666417
H 0.652828 -3.798279 -3.425452
H 0.275565 -2.072896 -3.181287
H -0.312321 -3.282105 -2.022416
C 4.386144 2.561597 -1.757891
H 4.274400 2.944508 -0.734146

H 4.750667 3.384763 -2.393829
H 5.140461 1.763931 -1.775956
C 3.071048 2.077544 -2.276523
C 1.869037 2.783352 -1.973669
H 1.899568 3.611179 -1.264201
C 0.631047 2.284036 -2.430320
H -0.287278 2.773865 -2.100391
C 0.532757 1.142387 -3.290344
C 1.729366 0.483370 -3.637048
H 1.687885 -0.435195 -4.224854
C 2.971194 0.914002 -3.101602
H 3.869236 0.320813 -3.282072
C -0.006712 -3.003353 3.155224
H -0.895411 -3.627735 3.039667
C 1.426917 2.095263 2.951888
H 1.794140 2.867361 3.641666
H 0.437413 2.369175 2.568640
H 1.402580 1.115937 3.446084
C -0.801113 0.694126 -3.796366
H -1.065402 1.246270 -4.713153
H -0.806057 -0.377275 -4.040356
H -1.590663 0.892663 -3.057249
P -0.883404 0.094980 -0.111053
H -0.387630 -0.119482 1.198123
C -2.233239 -1.154685 -0.069333
C -2.741731 -1.666108 1.130863
C -2.675383 -1.701833 -1.276448
C -3.671912 -2.703422 1.115670
H -2.408192 -1.254741 2.086362
C -3.611559 -2.734728 -1.286929
H -2.274722 -1.328756 -2.221120
C -4.115239 -3.241099 -0.092274
H -4.850417 -4.046827 -0.101221
C -1.791966 1.665289 0.196174
C -3.184293 1.774652 0.136448
C -1.029773 2.824627 0.393856
C -3.799829 3.017832 0.279754
H -3.800953 0.888524 -0.027451
C -1.654175 4.060982 0.541041
H 0.063712 2.770769 0.413827
C -3.041564 4.168546 0.482953

H -3.524668 5.140748 0.583129
C -0.789492 5.279698 0.736415
C -5.302695 3.104025 0.264705
C -4.158384 -3.283673 2.416603
C -4.010225 -3.347740 -2.602520
F -0.074426 5.193053 1.856878
F 0.068915 5.422024 -0.280155
F -1.516583 6.397560 0.804921
F -5.837446 2.183788 -0.542831
F -5.812355 2.901428 1.483805
F -5.723987 4.301403 -0.146693
F -5.177542 -3.986035 -2.520110
F -4.114166 -2.422181 -3.559650
F -3.096834 -4.235221 -3.012064
F -5.362610 -3.839959 2.292490
F -4.226884 -2.358944 3.374044
F -3.325934 -4.241927 2.855557

Table S66. Cartesian coordinates of **Coord**
Ru -0.826797 0.583407 -1.336212
N -2.075551 1.233966 0.340629
O -0.776505 -3.017761 -1.904199
P -2.656545 -0.272901 0.643260
S -1.601177 -2.752596 -0.701580
C -1.464072 -1.126429 -0.253654
C -2.684918 -0.720692 2.406097
O -3.018092 -3.178518 -0.741579
C -2.018231 0.048115 3.363785
H -1.571961 0.999880 3.066836
Si -2.889644 2.772559 0.509717
C -2.512074 -1.611150 5.047290
H -2.445706 -1.959180 6.080824
C -3.176479 -2.383846 4.092951
H -3.628568 -3.336733 4.378297
C -3.266831 -1.942134 2.775368
H -3.781411 -2.546198 2.021212
C -4.404842 -0.414651 0.146579
C -5.440381 -0.088705 1.029178
H -5.222516 0.108011 2.081570
C -6.755267 -0.022142 0.571351
H -7.559709 0.234741 1.264538

C -7.042025 -0.289174 -0.766640
H -8.073124 -0.238020 -1.124698
C -6.014569 -0.642514 -1.642582
H -6.241088 -0.882752 -2.684276
C -4.702092 -0.708922 -1.186553
H -3.895047 -1.023917 -1.843862
C -3.727495 2.931726 2.190437
H -3.006748 2.858333 3.018500
H -4.509315 2.176255 2.350828
H -4.207474 3.922133 2.253689
C -4.217435 3.054666 -0.800256
H -4.793024 3.961939 -0.554378
H -4.920515 2.208624 -0.840657
H -3.799147 3.196475 -1.807571
C -1.611975 4.150709 0.403523
H -2.092355 5.103842 0.131109
H -0.836335 3.935756 -0.344155
H -1.112661 4.306163 1.371924
C -2.517747 -1.088975 -3.965291
H -2.415553 -1.988834 -3.341002
H -2.215185 -1.368392 -4.986932
H -3.566793 -0.768921 -3.996320
C -1.621950 -0.004325 -3.466214
C -0.230757 -0.278734 -3.252478
H 0.121039 -1.301641 -3.392387
C 0.669664 0.756616 -2.900039
H 1.733678 0.529527 -2.793441
C 0.221522 2.095655 -2.673958
C -1.174467 2.295759 -2.707831
H -1.571538 3.276365 -2.439815
C -2.082754 1.277995 -3.151487
H -3.151109 1.494045 -3.199822
C -1.935523 -0.395161 4.683116
H -1.420647 0.212686 5.431032
C -0.901759 -3.776339 0.592255
H -1.090848 -4.813109 0.283868
H 0.172275 -3.592243 0.689353
H -1.419764 -3.553160 1.533528
C 1.185895 3.208586 -2.413149
H 1.414112 3.732372 -3.355820
H 0.782005 3.950239 -1.710635

H 2.134685 2.831509 -2.009248
P 0.598539 0.129659 0.324679
H 0.081992 0.190791 1.635978
C 1.936328 1.399638 0.480507
C 1.595332 2.641185 1.024479
C 3.210922 1.221877 -0.057861
C 2.510556 3.689059 1.018773
H 0.595428 2.794814 1.435715
C 4.123289 2.278945 -0.067223
H 3.504214 0.254991 -0.474751
C 3.782028 3.515355 0.471250
H 4.500002 4.336208 0.467394
C 1.578692 -1.400021 0.342193
C 2.131656 -1.871515 1.535043
C 1.787874 -2.106037 -0.842572
C 2.868819 -3.054593 1.539081
H 1.980963 -1.326838 2.470047
C 2.526633 -3.284618 -0.831194
H 1.332200 -1.763148 -1.767247
C 3.072808 -3.765029 0.357196
H 3.657416 -4.686437 0.364398
C 2.677141 -4.089067 -2.095179
C 3.387260 -3.601924 2.841468
C 5.452262 2.086090 -0.745091
C 2.092978 5.041979 1.527601
F 3.136087 5.762867 1.939215
F 1.480248 5.745438 0.565572
F 1.235786 4.943143 2.547392
F 6.342143 3.006607 -0.375422
F 5.968806 0.884727 -0.481635
F 5.317661 2.172040 -2.076129
F 3.695642 -2.623725 3.696621
F 4.477884 -4.348996 2.663902
F 2.470918 -4.372155 3.437792
F 3.914627 -4.588679 -2.201275
F 1.835143 -5.120028 -2.116337
F 2.447129 -3.349333 -3.181910

Table S67. Cartesian coordinates of **TS-Act2**

Ru 0.962291 0.780067 -1.350332
N 1.839810 -1.048753 -0.526438

O 1.455586 3.761995 0.697879
P 2.631729 -0.288032 0.689699
S 2.075142 2.625070 1.419033
C 1.724926 1.178773 0.596279
C 2.449116 -1.121479 2.292733
O 3.505777 2.733164 1.767180
C 1.484318 -2.118624 2.459156
H 0.923573 -2.465212 1.588543
Si 2.345037 -2.460071 -1.429948
C 1.962220 -2.187823 4.823507
H 1.773695 -2.606850 5.814849
C 2.920357 -1.185130 4.662474
H 3.479196 -0.817884 5.526550
C 3.167914 -0.650518 3.400073
H 3.909626 0.143545 3.269509
C 4.431859 -0.209349 0.444678
C 5.256252 -1.276611 0.820930
H 4.844356 -2.114717 1.387941
C 6.606770 -1.269292 0.480205
H 7.245851 -2.105676 0.772614
C 7.141711 -0.192140 -0.226674
H 8.201694 -0.186490 -0.491955
C 6.329220 0.887124 -0.574319
H 6.753840 1.746695 -1.098529
C 4.979064 0.880474 -0.236037
H 4.340840 1.732805 -0.458589
C 2.820475 -3.874041 -0.278558
H 1.961708 -4.206173 0.324193
H 3.636617 -3.615765 0.410739
H 3.156925 -4.732387 -0.882688
C 3.820829 -2.099972 -2.548808
H 4.186463 -3.035714 -3.002144
H 4.651566 -1.650153 -1.984033
H 3.562686 -1.416069 -3.370971
C 0.918634 -3.079415 -2.482345
H 1.282849 -3.809128 -3.222824
H 0.418223 -2.264933 -3.023464
H 0.165545 -3.583625 -1.861026
C 3.233525 3.391150 -2.035428
H 3.143251 3.673894 -0.977404
H 3.151463 4.318286 -2.624935

H 4.220769 2.953576 -2.228793

C 2.129935 2.468175 -2.434157

C 0.772287 2.810984 -2.128605

H 0.572214 3.689871 -1.515200

C -0.293762 2.020333 -2.610749

H -1.319102 2.315991 -2.378039

C -0.063436 0.840007 -3.387212

C 1.276683 0.432745 -3.534252

H 1.493301 -0.511915 -4.036528

C 2.366186 1.251570 -3.090693

H 3.392517 0.915650 -3.247462

C 1.244611 -2.655798 3.722895

H 0.494006 -3.439734 3.845268

C 1.218320 2.551153 2.989359

H 1.482271 3.473236 3.524492

H 0.136766 2.506365 2.809885

H 1.563074 1.667223 3.541560

C -1.201300 0.098783 -4.015215

H -1.401157 0.508258 -5.019118

H -0.984349 -0.971325 -4.137186

H -2.125328 0.209641 -3.430748

P -0.735366 0.172136 0.089526

H 0.353864 0.503421 1.161658

C -1.603731 -1.430291 0.052411

C -1.846310 -2.098980 1.255534

C -1.992554 -2.031253 -1.147576

C -2.455804 -3.353967 1.252798

H -1.547827 -1.640266 2.201227

C -2.610099 -3.278173 -1.144573

H -1.804343 -1.523389 -2.091205

C -2.844359 -3.948749 0.055633

H -3.330907 -4.925473 0.059376

C -2.065600 1.400827 0.273228

C -3.426309 1.077057 0.279862

C -1.684330 2.741285 0.433181

C -4.385442 2.077156 0.435903

H -3.747889 0.039809 0.165529

C -2.651627 3.728141 0.600711

H -0.624322 3.023766 0.430751

C -4.007857 3.408049 0.600617

H -4.760137 4.186454 0.729235

C -2.190513 5.151078 0.779272
C -5.844087 1.715015 0.363832
C -2.974349 -3.946820 -2.442578
C -2.631306 -4.085998 2.555547
F -2.960383 -3.256780 3.546188
F -3.569362 -5.028497 2.476904
F -1.489612 -4.694120 2.918542
F -4.172679 -4.529202 -2.374330
F -2.998832 -3.079156 -3.458105
F -2.090958 -4.901099 -2.756634
F -6.074877 0.500653 0.870136
F -6.275973 1.699192 -0.903245
F -6.605187 2.585668 1.028971
F -3.218310 5.998677 0.877698
F -1.449713 5.286335 1.879807
F -1.442627 5.546884 -0.256352

Table S68. Cartesian coordinates of **Act'**
Ru -0.916903 0.477058 -1.096070
Si -1.705542 -3.033652 -0.214522
P -2.859790 -0.410045 0.728448
O -3.796918 2.463484 2.046036
S -3.064359 2.582311 0.774999
N -1.823975 -1.278326 -0.157530
H -1.376107 1.243086 1.536676
C -2.004221 1.171646 0.632179
C -3.027028 -1.000633 2.436741
O -3.839830 2.815533 -0.451782
C -1.847021 -1.061654 3.192709
H -0.899756 -0.728763 2.751249
C -1.879129 -1.556813 4.492355
H -0.957829 -1.601437 5.077655
C -4.255299 -1.945472 4.291216
H -5.197437 -2.295721 4.718869
C -4.230677 -1.449777 2.989041
H -5.149388 -1.423892 2.400475
C -4.528684 -0.356453 0.015795
C -6.846441 0.319945 0.022113
H -7.663213 0.886513 0.474719
C -5.581292 0.361942 0.600361
H -5.402544 0.973142 1.487253

C -7.071579 -0.433338 -1.130522
H -8.068712 -0.464386 -1.576283
C -0.415956 -3.754529 0.948022
H 0.620554 -3.593822 0.631342
H -0.580741 -4.842045 1.019281
H -0.537630 -3.331437 1.957075
C -3.322313 -3.834968 0.342276
H -3.516310 -3.646260 1.409189
H -3.217606 -4.925566 0.218004
H -4.206465 -3.516466 -0.227291
C -4.753266 -1.093847 -1.149079
H -3.915273 -1.622473 -1.607636
C -6.023677 -1.139143 -1.717748
H -6.193431 -1.721536 -2.626449
C -1.381205 -3.545342 -1.993909
H -0.528240 -3.013551 -2.436312
H -2.266039 -3.349080 -2.620412
H -1.160149 -4.622276 -2.050962
C -3.712861 1.264537 -3.146045
H -4.229334 1.685124 -2.273263
H -4.172431 0.306934 -3.417433
H -3.856115 1.957768 -3.991286
C -0.070174 2.146828 -2.266396
H 0.484320 3.006159 -1.887775
C 0.632977 0.983050 -2.688230
C -0.120568 -0.135679 -3.120782
H 0.390917 -1.041814 -3.447921
C -1.538309 -0.065618 -3.184516
H -2.092417 -0.954175 -3.495064
C -2.250383 1.112523 -2.881697
C -1.483672 2.182888 -2.328733
H -2.023639 3.062187 -1.974458
C -3.083007 -1.998103 5.042987
H -3.106281 -2.388127 6.063379
C 2.126306 0.974468 -2.754190
H 2.430062 1.235447 -3.781095
H 2.572257 1.710841 -2.075856
H 2.546038 -0.013637 -2.525092
C -1.974482 3.977765 0.962497
H -1.364719 4.116668 0.062718
H -2.652460 4.831750 1.094093

H -1.356049 3.850970 1.858699
P 0.741241 0.132404 0.623309
C 1.968036 1.497949 0.532079
C 1.467370 2.805214 0.606537
C 3.360006 1.354065 0.428144
C 2.302269 3.914807 0.536816
H 0.390381 2.947416 0.687849
C 4.196279 2.467941 0.368431
H 3.809970 0.361795 0.371241
C 3.682375 3.761736 0.416358
H 4.340001 4.628870 0.360244
C 1.860085 -1.280519 0.261461
C 2.734518 -1.716681 1.265585
C 1.833974 -1.996023 -0.936205
C 3.582666 -2.802772 1.053905
H 2.746951 -1.204394 2.231368
C 2.665753 -3.093706 -1.141934
H 1.130748 -1.689527 -1.706746
C 3.555367 -3.502150 -0.151025
H 4.214003 -4.356834 -0.309227
C 2.557750 -3.879889 -2.417360
C 4.562631 -3.185182 2.129778
C 5.669585 2.257407 0.149492
C 1.670640 5.276534 0.552645
F 2.565008 6.259162 0.506200
F 0.916015 5.463474 1.642691
F 0.848277 5.433559 -0.504896
F 6.390082 3.302171 0.563309
F 6.116279 1.174704 0.788585
F 5.939097 2.083124 -1.152020
F 4.012675 -3.102171 3.344228
F 5.624572 -2.372496 2.123771
F 5.014452 -4.431240 1.972445
F 3.731885 -4.389060 -2.790997
F 2.106961 -3.120271 -3.428243
F 1.705935 -4.904378 -2.292294

Table S69. Cartesian coordinates of **TS-Act3**
Ru -0.879177 0.224197 -1.173568
Si -1.826838 -3.053941 0.256611
P -2.905385 -0.271687 0.706679

O -3.744213 2.817819 1.507661

S -2.967741 2.696317 0.263101

N -1.883958 -1.313238 0.014458

H -1.371381 1.422801 1.290347

C -1.974011 1.235723 0.385134

C -3.143019 -0.562790 2.481989

O -3.695357 2.757918 -1.012603

C -1.979665 -0.581534 3.265649

H -1.004771 -0.396992 2.798272

C -2.064988 -0.848150 4.628410

H -1.156847 -0.860865 5.235425

C -4.461481 -1.091561 4.436342

H -5.432362 -1.296084 4.893221

C -4.383906 -0.823920 3.070971

H -5.290630 -0.830492 2.463608

C -4.548281 -0.272867 -0.065997

C -6.827966 0.495148 -0.273661

H -7.628526 1.170146 0.036933

C -5.580851 0.585374 0.337648

H -5.399741 1.338871 1.106846

C -7.055235 -0.443784 -1.280235

H -8.038227 -0.510587 -1.752682

C -0.549198 -3.587595 1.525372

H 0.478155 -3.295170 1.280796

H -0.574616 -4.685004 1.624764

H -0.803693 -3.153873 2.505074

C -3.466421 -3.696628 0.936223

H -3.665745 -3.305955 1.945548

H -3.392033 -4.793667 1.018849

H -4.335071 -3.466822 0.302919

C -4.774271 -1.199696 -1.086817

H -3.951468 -1.840967 -1.408796

C -6.026984 -1.290844 -1.688284

H -6.197883 -2.020993 -2.482896

C -1.539328 -3.875155 -1.410880

H -0.664956 -3.478214 -1.943416

H -2.419939 -3.739603 -2.059230

H -1.379344 -4.956882 -1.283949

C -3.592547 0.840109 -3.396160

H -4.097725 1.424004 -2.616280

H -4.106973 -0.119296 -3.527156

H -3.673068 1.398023 -4.343694
C 0.094928 1.610176 -2.576076
H 0.708775 2.475093 -2.318707
C 0.715666 0.349070 -2.810202
C -0.112591 -0.771090 -3.054643
H 0.339058 -1.751095 -3.213612
C -1.521478 -0.616283 -3.156785
H -2.135273 -1.503165 -3.328792
C -2.148398 0.641594 -3.069195
C -1.313037 1.732965 -2.672738
H -1.792539 2.692797 -2.475805
C -3.305468 -1.102656 5.214777
H -3.370475 -1.313907 6.284835
C 2.202032 0.221854 -2.885589
H 2.496078 0.262278 -3.947144
H 2.715073 1.041273 -2.370682
H 2.556838 -0.734782 -2.480567
C -1.818691 4.054347 0.253093
H -1.081909 3.929299 -0.548406
H -2.444384 4.936692 0.063629
H -1.340843 4.141683 1.235233
P 0.749416 0.117994 0.608037
C 1.925648 1.520911 0.407194
C 1.450080 2.802973 0.724141
C 3.261933 1.423301 -0.003645
C 2.247649 3.933891 0.586640
H 0.434088 2.909482 1.102571
C 4.063741 2.557054 -0.134949
H 3.695289 0.448693 -0.232866
C 3.568445 3.825005 0.150193
H 4.200793 4.707122 0.051592
C 1.925288 -1.277400 0.399994
C 2.968973 -1.434837 1.325873
C 1.765501 -2.267798 -0.569757
C 3.841987 -2.515844 1.244618
H 3.100781 -0.700781 2.124923
C 2.625746 -3.362706 -0.637944
H 0.940655 -2.173522 -1.273868
C 3.678407 -3.493038 0.261793
H 4.358751 -4.343294 0.205857
C 1.658333 5.285666 0.874464

C 5.460170 2.384185 -0.665325

C 5.004607 -2.599758 2.196270

C 2.369995 -4.430483 -1.662275

F 5.417672 -3.858611 2.363889

F 6.049217 -1.897653 1.743996

F 4.693274 -2.110331 3.398940

F 3.474687 -5.110143 -1.968217

F 1.888971 -3.908992 -2.803765

F 1.464781 -5.317421 -1.232559

F 6.206645 3.474526 -0.487283

F 6.085663 1.359510 -0.083310

F 5.438572 2.128529 -1.983445

F 2.591438 6.207645 1.100348

F 0.846907 5.257006 1.939160

F 0.916103 5.720135 -0.159315

Table S70. Cartesian coordinates of **Act**

Ru -0.376137 -1.398851 -0.745179

Si -1.845588 -2.172267 2.494572

P -2.813495 -0.395807 0.224107

O -2.978094 0.049839 -3.126388

S -2.056787 0.972870 -2.454188

N -1.877016 -1.547216 0.853342

H -1.136526 1.217649 -0.366737

C -1.617212 0.367708 -0.865127

C -3.490757 0.803326 1.412874

O -0.819867 1.350078 -3.153431

C -2.719332 1.905056 1.805156

H -1.746692 2.094604 1.346802

C -3.170571 2.760629 2.805826

H -2.554837 3.612186 3.101598

C -5.170778 1.424504 3.042255

H -6.130862 1.234627 3.527499

C -4.723530 0.570370 2.036968

H -5.335097 -0.283861 1.736858

C -4.269275 -1.068437 -0.618714

C -6.381806 -0.821558 -1.762893

H -7.171003 -0.181390 -2.163042

C -5.285253 -0.251644 -1.126754

H -5.231280 0.833477 -1.017445

C -6.472891 -2.208666 -1.891619

H -7.335404 -2.653105 -2.393849
C -1.709104 -0.835236 3.808556
H -0.827852 -0.208862 3.602526
H -1.578950 -1.304246 4.797791
H -2.597173 -0.188340 3.856402
C -3.440860 -3.134898 2.797130
H -4.326429 -2.499174 2.637687
H -3.479969 -3.504247 3.834714
H -3.532204 -4.004237 2.126918
C -4.371470 -2.454610 -0.734168
H -3.580476 -3.070509 -0.307306
C -5.471089 -3.025357 -1.372157
H -5.547329 -4.111773 -1.459344
C -0.378051 -3.321751 2.695398
H 0.545977 -2.727185 2.678846
H -0.314223 -4.092037 1.915029
H -0.437249 -3.828065 3.671857
C 1.827741 -0.523768 -3.233702
H 1.240138 0.352922 -3.536053
H 2.263781 -0.972029 -4.141822
H 2.662342 -0.205140 -2.593866
C 0.676000 -3.369283 -0.900938
H 1.108363 -3.981635 -0.108539
C -0.667450 -3.577909 -1.325435
C -1.192925 -2.760564 -2.355267
H -2.228671 -2.870075 -2.679081
C -0.396920 -1.727679 -2.913239
H -0.842517 -1.067908 -3.657140
C 0.960643 -1.526827 -2.541512
C 1.458089 -2.357924 -1.505991
H 2.478983 -2.204718 -1.157935
C -4.395479 2.518054 3.427404
H -4.747771 3.186034 4.216891
C -1.504111 -4.636695 -0.675661
H -2.368883 -4.897688 -1.299368
H -0.912919 -5.547121 -0.502035
H -1.874494 -4.288077 0.298985
C -2.917441 2.511259 -2.165557
H -2.214300 3.221887 -1.714948
H -3.227070 2.865407 -3.157759
H -3.789792 2.344104 -1.522756

P 0.857033 -0.205981 0.961423
C 0.963103 1.604091 0.573772
C 0.908668 2.492735 1.653111
C 1.039792 2.136039 -0.721349
C 0.926824 3.873759 1.447522
H 0.843038 2.093541 2.668640
C 1.064392 3.514568 -0.923785
H 1.044274 1.465421 -1.581564
C 1.009863 4.392500 0.158938
H 1.036791 5.471203 -0.002243
C 2.632862 -0.634841 0.761646
C 3.597623 0.152084 0.119953
C 3.047497 -1.877262 1.267874
C 4.901444 -0.311348 -0.057610
H 3.331245 1.136507 -0.269287
C 4.344558 -2.342025 1.077452
H 2.328575 -2.507904 1.796278
C 5.289655 -1.564222 0.409174
H 6.306382 -1.926198 0.259340
C 4.683173 -3.722009 1.567070
C 5.863026 0.546203 -0.833419
C 0.781568 4.800657 2.621412
C 1.085462 4.077161 -2.320200
F -0.517263 4.993756 2.923473
F 1.301927 6.003533 2.378699
F 1.362502 4.313849 3.717306
F 1.769066 5.226409 -2.369310
F -0.156707 4.352761 -2.746473
F 1.637122 3.239960 -3.191970
F 5.548978 0.556645 -2.137622
F 5.834718 1.815402 -0.419370
F 7.120573 0.112951 -0.733176
F 4.394819 -3.872606 2.862479
F 5.971960 -4.018107 1.400536
F 3.971481 -4.649880 0.904782

Table S71. Cartesian coordinates of **TS1**
not found

Table S72. Cartesian coordinates of **Int1**
Ru -0.443103 -1.750250 -1.352745

Si -0.492697 -0.512553 3.304607
P -2.471352 -0.458215 0.891680
O -3.781115 -0.876928 -2.603738
S -2.849253 0.205099 -2.233583
N -1.272990 -0.824593 1.818326
H -1.311931 0.735128 -0.683997
C -1.858667 -0.209937 -0.819787
C -3.268396 1.133053 1.317284
O -1.912418 0.669354 -3.269769
C -2.469821 2.284159 1.255808
H -1.421191 2.206036 0.958171
C -2.994361 3.528159 1.585716
H -2.356233 4.413367 1.537736
C -5.123283 2.496031 2.067008
H -6.163599 2.576722 2.391180
C -4.597017 1.246072 1.737378
H -5.225736 0.355056 1.804428
C -3.805267 -1.700964 0.987637
C -5.853086 -2.771023 0.273432
H -6.672933 -2.852162 -0.444215
C -4.867902 -1.803134 0.082364
H -4.911527 -1.167155 -0.801895
C -5.790417 -3.636139 1.365332
H -6.565059 -4.393485 1.508598
C -0.708607 1.251917 3.932025
H -0.276393 1.997169 3.248738
H -0.209196 1.368445 4.907713
H -1.772691 1.503141 4.063312
C -1.140452 -1.666868 4.654522
H -2.204435 -1.474770 4.868206
H -0.581228 -1.529596 5.594256
H -1.037646 -2.723970 4.359153
C -3.746363 -2.572110 2.082457
H -2.904249 -2.486916 2.772104
C -4.734870 -3.535257 2.271121
H -4.678626 -4.210552 3.128244
C 1.352691 -0.856695 3.119325
H 1.773570 -0.313621 2.260356
H 1.551424 -1.926983 2.955301
H 1.907329 -0.550450 4.020619
C -1.479721 -2.569407 -4.444710

H -2.397484 -1.970600 -4.388072

H -1.670784 -3.452875 -5.076514

H -0.700273 -1.962972 -4.925215

C 0.735750 -3.623652 -1.472016

H 1.797983 -3.760473 -1.264179

C -0.211657 -3.714758 -0.408981

C -1.587043 -3.520098 -0.725944

H -2.328092 -3.569134 0.071459

C -1.998275 -3.203257 -2.041717

H -3.041652 -2.950764 -2.229651

C -1.054753 -3.001706 -3.078476

C 0.323873 -3.225284 -2.762415

H 1.078769 -3.038329 -3.528741

C -4.327101 3.636969 1.986748

H -4.742775 4.613804 2.244667

C 0.183875 -4.016556 1.001410

H -0.215612 -3.231917 1.661533

H -0.234759 -4.985846 1.314245

H 1.274102 -4.070533 1.119674

C -3.871950 1.611402 -1.837933

H -3.243756 2.450652 -1.519940

H -4.382699 1.848824 -2.780765

H -4.594948 1.348152 -1.057082

P 1.101752 -0.230522 -0.966109

C 0.908105 1.551135 -0.653153

C 1.413227 2.140063 0.516961

C 0.192515 2.346074 -1.560404

C 1.200813 3.489947 0.772652

H 1.962210 1.539669 1.243899

C -0.020082 3.698140 -1.287104

H -0.226475 1.896294 -2.466331

C 0.485570 4.279674 -0.128763

H 0.328191 5.341575 0.068934

C 2.840473 -0.528552 -0.558952

C 3.881643 0.338495 -0.925456

C 3.158012 -1.697872 0.136442

C 5.199434 0.022524 -0.610778

H 3.663678 1.260439 -1.468477

C 4.480529 -2.018281 0.431222

H 2.348821 -2.348000 0.468046

C 5.511998 -1.163274 0.059373

H 6.548656 -1.415132 0.286061
C 4.746890 -3.293932 1.181032
C 6.308058 0.979823 -0.960644
C 1.694987 4.124349 2.044937
C -0.851451 4.531945 -2.224694
F 6.045655 -3.575393 1.256344
F 4.139443 -4.333393 0.584537
F 4.268480 -3.237741 2.427175
F 7.420400 0.330183 -1.313056
F 6.619741 1.757692 0.080682
F 5.968122 1.783909 -1.969382
F 2.415106 3.280456 2.780939
F 2.450121 5.196368 1.792560
F 0.666515 4.540697 2.796148
F -0.473892 5.812312 -2.211193
F -0.786685 4.095493 -3.477101
F -2.146854 4.508718 -1.860241

Table S73. Cartesian coordinates of **TS4**
Ru -0.355287 -1.546681 -1.164017
Si -3.135229 -2.270912 2.825215
P -2.943830 -0.268952 0.594950
O -3.370373 -0.310066 -2.886604
S -2.356370 0.616190 -2.363080
N -2.642126 -1.638077 1.300765
H -1.200006 0.970923 -0.444807
C -1.758702 0.085557 -0.778876
C -2.764547 1.174075 1.715300
O -1.164212 0.889126 -3.188548
C -1.468991 1.627969 2.002964
H -0.608745 1.181130 1.500708
C -1.255247 2.626009 2.949200
H -0.239908 2.972478 3.151562
C -3.624475 2.707953 3.387666
H -4.474206 3.118650 3.938211
C -3.838123 1.709867 2.437731
H -4.851117 1.339899 2.272223
C -4.658359 -0.220594 -0.051975
C -6.744001 0.841275 -0.688224
H -7.349590 1.745671 -0.783720
C -5.445129 0.928994 -0.190109

H -5.053675 1.904457 0.104581
C -7.269527 -0.395126 -1.057773
H -8.286483 -0.462038 -1.451357
C -1.805410 -1.929266 4.115334
H -0.836224 -2.355116 3.810317
H -2.066606 -2.345952 5.101364
H -1.667163 -0.841626 4.228289
C -4.770505 -1.574996 3.466357
H -4.665835 -0.526520 3.783468
H -5.117291 -2.152470 4.338869
H -5.555575 -1.622851 2.694634
C -5.204732 -1.459334 -0.405953
H -4.601813 -2.358961 -0.263953
C -6.497062 -1.546666 -0.913397
H -6.906020 -2.520036 -1.193952
C -3.378118 -4.133142 2.638030
H -2.463673 -4.643034 2.301177
H -4.172874 -4.344535 1.903947
H -3.680062 -4.587060 3.595627
C -1.079791 -1.981368 -4.455005
H -2.040667 -1.454521 -4.476130
H -1.105813 -2.804929 -5.188409
H -0.294665 -1.272814 -4.749147
C 0.794651 -3.415593 -1.420435
H 1.827129 -3.600558 -1.120835
C -0.259610 -3.629291 -0.487398
C -1.597570 -3.363358 -0.902695
H -2.400264 -3.452002 -0.172564
C -1.860664 -2.844720 -2.187464
H -2.872562 -2.532586 -2.449316
C -0.811434 -2.542927 -3.097370
C 0.523614 -2.828900 -2.676444
H 1.352990 -2.557488 -3.332462
C -2.336213 3.175056 3.638684
H -2.170945 3.959411 4.380989
C 0.010860 -4.130843 0.894976
H -0.412920 -5.139404 1.021144
H 1.087023 -4.194652 1.100836
H -0.466637 -3.467560 1.629019
C -3.138648 2.210933 -2.194980
H -2.496676 2.883245 -1.613939

H -3.234397 2.578872 -3.224855
H -4.123551 2.090932 -1.736862
P 1.249956 -0.208541 -0.454425
C 1.331279 1.588401 -0.209245
C 2.024817 2.118325 0.889670
C 0.682001 2.465734 -1.091040
C 2.056753 3.491885 1.108641
H 2.546098 1.451517 1.580601
C 0.704352 3.838748 -0.846129
H 0.168955 2.065841 -1.973229
C 1.390969 4.361994 0.246728
H 1.424362 5.438886 0.416009
C 2.966855 -0.726349 -0.178510
C 3.198447 -1.912487 0.523979
C 4.069003 -0.022587 -0.689019
C 4.491444 -2.410378 0.677502
H 2.351230 -2.442064 0.963703
C 5.358140 -0.514970 -0.515728
H 3.918279 0.914675 -1.228890
C 5.580245 -1.716775 0.161144
H 6.592614 -2.100962 0.290948
C 2.769534 4.031543 2.319257
C -0.050368 4.770821 -1.755570
C 6.528932 0.216016 -1.118428
C 4.673507 -3.699072 1.431169
F -1.312802 4.939475 -1.321358
F -0.125940 4.305666 -2.999310
F 0.506985 5.981711 -1.799087
F 3.154347 5.295589 2.145941
F 3.849347 3.308680 2.617262
F 1.973575 4.008122 3.401024
F 6.272003 1.515450 -1.274036
F 7.619304 0.102399 -0.356657
F 6.836875 -0.276068 -2.322968
F 4.360826 -3.561701 2.721403
F 5.923118 -4.152523 1.361128
F 3.867437 -4.654267 0.937968

Table S74. Cartesian coordinates of **Int3**

Ru -0.319297 -1.681568 -0.877309
P -2.667353 -0.353615 0.868530

O -3.593800 -0.595743 -2.345357
S -2.557324 0.411137 -2.068480
N -3.895667 -1.291131 0.785632
H -1.059227 0.890755 -0.405943
C -1.642761 -0.017282 -0.614805
C -1.384080 -1.064637 1.952902
O -1.592844 0.709154 -3.138719
C -0.074916 -1.275050 1.501997
H 0.346037 -0.720925 0.598310
C 0.863468 -1.900932 2.329876
H 1.890488 -2.038954 1.994089
C -0.823045 -2.139421 4.043370
H -1.118519 -2.484848 5.036859
C -1.756583 -1.513640 3.221017
H -2.792247 -1.387278 3.547427
C -3.002150 1.275631 1.632376
C -2.254812 3.454897 2.379143
H -1.449380 4.180533 2.511110
C -1.982444 2.220873 1.796368
H -0.961307 1.994181 1.481029
C -3.550740 3.752672 2.803858
H -3.766128 4.722702 3.258087
C -4.291473 1.570176 2.080920
H -5.075576 0.817840 1.975929
C -4.566321 2.809005 2.659300
H -5.578533 3.035714 3.002340
C 0.522383 -2.211451 -4.085081
H 0.011883 -1.281277 -4.370616
H 0.382783 -2.955932 -4.885664
H 1.594699 -1.996481 -3.987435
C 0.193772 -3.835844 -0.624573
H 0.842659 -4.266409 0.142706
C -1.212786 -3.715612 -0.357216
C -2.010178 -3.091831 -1.329801
H -3.059721 -2.887665 -1.123928
C -1.425053 -2.578186 -2.524489
H -2.056887 -2.026586 -3.219342
C -0.048671 -2.738961 -2.811361
C 0.760270 -3.402269 -1.836672
H 1.831288 -3.517633 -2.011571
C 0.487119 -2.330568 3.599229

H 1.223476 -2.814803 4.244418

C -1.802436 -4.199771 0.927359

H -2.100052 -5.256225 0.826069

H -1.078698 -4.129471 1.751313

H -2.691437 -3.605206 1.181613

C -3.401833 1.935134 -1.697570

H -2.674895 2.700406 -1.403153

H -3.901379 2.219596 -2.633264

H -4.139688 1.760266 -0.905483

P 1.359187 -0.113822 -1.649953

C 1.118390 1.556845 -0.896084

C 1.534215 1.893960 0.399281

C 0.487364 2.546201 -1.667030

C 1.340800 3.180053 0.903054

H 2.015088 1.145086 1.032530

C 0.288926 3.826367 -1.153245

H 0.135862 2.296607 -2.670568

C 0.722298 4.158078 0.129662

H 0.574279 5.164760 0.522218

C 2.948664 -0.524281 -0.801569

C 4.030366 0.373799 -0.886851

C 3.192003 -1.755439 -0.191946

C 5.277569 0.049035 -0.367955

H 3.895715 1.341779 -1.374782

C 4.448417 -2.087798 0.317350

H 2.377386 -2.472199 -0.107101

C 5.504968 -1.191171 0.237281

H 6.489817 -1.450509 0.627298

Si -5.522042 -1.590536 0.338292

C -6.365598 -0.153540 -0.545070

H -6.366694 0.775812 0.045793

H -5.850665 0.033813 -1.499202

H -7.415405 -0.406957 -0.765533

C -6.483117 -1.984501 1.914085

H -6.484379 -1.129419 2.609532

H -7.532243 -2.242592 1.697148

H -6.024059 -2.836497 2.440714

C -5.629323 -3.082197 -0.806076

H -5.169307 -2.848743 -1.778995

H -5.126715 -3.963928 -0.377332

H -6.681382 -3.353568 -0.989270

C 1.822241 3.506090 2.290316
C -0.436751 4.861409 -1.969571
C 6.401275 1.049882 -0.416934
C 4.604502 -3.415362 1.001147
F 6.204645 1.976783 -1.355344
F 6.529926 1.687177 0.751810
F 7.571783 0.456393 -0.669342
F 5.877832 -3.761229 1.164688
F 4.034428 -3.403715 2.219495
F 3.997752 -4.394128 0.313200
F 1.668656 2.465306 3.114428
F 3.115353 3.833618 2.302836
F 1.147434 4.537546 2.815315
F -1.751996 4.860472 -1.686636
F -0.317967 4.643393 -3.276308
F 0.008774 6.093116 -1.713873

Table S75. Cartesian coordinates of **TS5**
Ru -0.308392 -1.738732 -0.712569
P -2.719364 -0.360268 0.888439
O -3.483164 -0.713884 -2.395962
S -2.463191 0.300668 -2.087092
N -3.898066 -1.361791 0.783940
H -1.061677 0.831862 -0.353301
C -1.637919 -0.079172 -0.567237
C -1.383360 -0.900806 1.983693
O -1.435026 0.567795 -3.106408
C -0.234909 -1.444137 1.377775
H 0.649664 -0.799440 0.184300
C 0.769042 -1.940236 2.224403
H 1.689701 -2.351881 1.810258
C -0.530522 -1.384895 4.192567
H -0.642275 -1.363270 5.278771
C -1.535010 -0.880602 3.372320
H -2.450626 -0.469359 3.806934
C -3.171209 1.292534 1.535738
C -2.555031 3.547336 2.175187
H -1.788957 4.316543 2.295259
C -2.203988 2.294153 1.683142
H -1.161570 2.090545 1.429395
C -3.880052 3.808991 2.529091

H -4.157968 4.793179 2.913636
C -4.489202 1.553111 1.915499
H -5.234637 0.760561 1.830307
C -4.843960 2.810305 2.405307
H -5.878531 3.007388 2.695959
C 0.605745 -2.481148 -4.004264
H -0.153289 -2.170475 -4.732475
H 1.232129 -3.271107 -4.444423
H 1.249349 -1.605911 -3.797147
C 0.200651 -3.919076 -0.469310
H 0.836108 -4.345683 0.310073
C -1.205540 -3.782838 -0.232622
C -1.973298 -3.164368 -1.236589
H -3.021469 -2.936266 -1.050803
C -1.386612 -2.758995 -2.473408
H -2.022681 -2.265451 -3.206982
C -0.023327 -2.946372 -2.735914
C 0.776314 -3.495988 -1.682415
H 1.850062 -3.617780 -1.840384
C 0.621371 -1.913525 3.611204
H 1.424463 -2.306837 4.239560
C -1.833808 -4.216265 1.051552
H -2.209526 -5.247235 0.950137
H -1.112378 -4.191026 1.879578
H -2.679425 -3.555282 1.293544
C -3.329554 1.834054 -1.815035
H -2.619602 2.612634 -1.513483
H -3.779306 2.080947 -2.786008
H -4.107432 1.684951 -1.056579
P 1.360369 -0.193038 -1.509635
C 1.098840 1.519385 -0.860970
C 1.388909 1.888616 0.458717
C 0.574941 2.481667 -1.731941
C 1.169745 3.194339 0.893214
H 1.778194 1.149013 1.163063
C 0.349489 3.784367 -1.285904
H 0.315428 2.195799 -2.753305
C 0.652418 4.151418 0.022781
H 0.479006 5.172564 0.364326
C 2.988788 -0.517149 -0.694820
C 4.008692 0.448385 -0.741464

C 3.303215 -1.761969 -0.150910
C 5.276415 0.171817 -0.240947
H 3.811384 1.435654 -1.164618
C 4.573385 -2.037519 0.353083
H 2.533124 -2.530551 -0.102811
C 5.573165 -1.074061 0.317354
H 6.564808 -1.283625 0.719589
Si -5.509040 -1.697424 0.311507
C -6.328916 -0.329433 -0.697111
H -6.381553 0.631317 -0.161682
H -5.761548 -0.183462 -1.628742
H -7.359234 -0.617612 -0.962581
C -6.530848 -2.007448 1.867951
H -6.570041 -1.114844 2.513126
H -7.567264 -2.290676 1.622851
H -6.084767 -2.820936 2.462060
C -5.570190 -3.259562 -0.741843
H -5.101858 -3.082281 -1.722857
H -5.057994 -4.103171 -0.252102
H -6.614508 -3.561447 -0.921792
C -0.277062 4.791035 -2.213147
C 1.509391 3.565608 2.311725
C 6.365275 1.207533 -0.339147
C 4.805009 -3.385494 0.974617
F 0.812501 4.633872 2.719290
F 1.240516 2.563675 3.151957
F 2.803084 3.862187 2.447478
F 0.059508 6.040096 -1.887060
F -1.617634 4.722033 -2.160589
F 0.076436 4.586011 -3.479521
F 7.129030 1.004764 -1.417768
F 7.171289 1.169750 0.725058
F 5.868454 2.442116 -0.430836
F 4.361257 -4.368243 0.177837
F 4.136126 -3.506956 2.132494
F 6.089603 -3.616044 1.229726

Table S76. Cartesian coordinates of **Int4**
Ru -0.318296 -1.786145 -0.613122
P -2.810429 -0.358038 0.830095
O -3.363640 -0.744585 -2.508547

S -2.360038 0.264350 -2.135587
N -3.996354 -1.341716 0.655812
H -1.060329 0.792623 -0.321665
C -1.637015 -0.111533 -0.563519
C -1.536863 -0.874318 2.001534
O -1.264872 0.520557 -3.088837
C -0.376743 -1.437614 1.441399
H 0.843725 -0.780625 0.002634
C 0.622272 -1.854609 2.336502
H 1.553561 -2.289346 1.968198
C -0.708736 -1.179289 4.248332
H -0.831822 -1.080797 5.329092
C -1.710110 -0.751521 3.383613
H -2.632014 -0.313165 3.776709
C -3.279584 1.316693 1.408815
C -2.664321 3.570623 2.053327
H -1.892116 4.323729 2.225408
C -2.303777 2.298760 1.619701
H -1.248748 2.062844 1.463825
C -4.008090 3.871268 2.284899
H -4.293414 4.870043 2.623726
C -4.617989 1.616411 1.667878
H -5.372710 0.839320 1.534985
C -4.982275 2.892463 2.098862
H -6.032750 3.119903 2.295005
C 0.758103 -2.591947 -3.836083
H 1.820333 -2.375716 -3.655690
H 0.285746 -1.683603 -4.235705
H 0.694188 -3.387577 -4.595559
C 0.161561 -3.954470 -0.286772
H 0.749804 -4.367570 0.535240
C -1.251962 -3.786052 -0.129063
C -1.958177 -3.184599 -1.191247
H -3.010537 -2.936525 -1.060802
C -1.306223 -2.823360 -2.409205
H -1.896461 -2.338525 -3.185884
C 0.069719 -3.020695 -2.582415
C 0.802497 -3.562869 -1.477915
H 1.883648 -3.687200 -1.570169
C 0.459012 -1.727275 3.716371
H 1.260551 -2.059080 4.381702

C -1.957114 -4.178862 1.127747

H -2.337782 -5.208260 1.027631

H -1.283856 -4.139330 1.994602

H -2.808096 -3.504283 1.304236

C -3.230339 1.806901 -1.935579

H -2.538090 2.581542 -1.586152

H -3.605730 2.051374 -2.938240

H -4.062598 1.669176 -1.235069

P 1.383479 -0.201909 -1.443980

C 1.128077 1.505575 -0.780438

C 1.376729 1.838194 0.556887

C 0.644856 2.487743 -1.649869

C 1.149870 3.134675 1.012680

H 1.728367 1.075850 1.256909

C 0.418666 3.783786 -1.183364

H 0.411739 2.222972 -2.683370

C 0.674912 4.115834 0.143764

H 0.494791 5.129927 0.502578

C 3.047734 -0.512946 -0.691902

C 4.087546 0.408188 -0.889966

C 3.344005 -1.707659 -0.039883

C 5.371680 0.134171 -0.431188

H 3.896762 1.351383 -1.407282

C 4.634206 -1.984549 0.410343

H 2.548032 -2.433395 0.130450

C 5.660269 -1.068294 0.219773

H 6.671447 -1.285926 0.565257

Si -5.578082 -1.673846 0.095755

C -6.317638 -0.330278 -1.004518

H -6.404214 0.644663 -0.499870

H -5.684441 -0.210342 -1.896597

H -7.327814 -0.622359 -1.335203

C -6.702047 -1.925900 1.590971

H -6.769894 -1.013569 2.205442

H -7.724431 -2.204305 1.288117

H -6.305743 -2.725900 2.236429

C -5.588144 -3.266664 -0.913195

H -5.056827 -3.123257 -1.867379

H -5.114401 -4.097741 -0.366558

H -6.621333 -3.567483 -1.150099

C 4.864200 -3.289799 1.118995

C 6.464417 1.157991 -0.593393
C 1.431427 3.472585 2.452668
C -0.166571 4.814635 -2.111129
F 0.266440 4.657121 -3.360030
F -1.505892 4.725191 -2.143061
F 0.131583 6.055444 -1.723595
F 0.704428 4.520790 2.859243
F 1.146046 2.446001 3.255258
F 2.714792 3.783908 2.643294
F 6.202751 2.009207 -1.586653
F 6.616963 1.880308 0.520699
F 7.639749 0.578755 -0.850760
F 6.149017 -3.504309 1.387202
F 4.194035 -3.335631 2.279097
F 4.421294 -4.320092 0.383694

Table S77. Cartesian coordinates of **TS6**
Ru -0.440957 -1.882984 -0.473446
P -2.983597 -0.382087 0.811190
O -3.274865 -0.699293 -2.582666
S -2.286868 0.275186 -2.097236
N -4.203536 -1.287388 0.508605
H -1.116608 0.726407 -0.182707
C -1.702544 -0.156716 -0.482879
C -1.844027 -1.004758 2.066805
O -1.114526 0.533590 -2.955705
C -0.676840 -1.616800 1.573017
H 0.912988 -0.594600 0.655767
C 0.223786 -2.117703 2.530684
H 1.157123 -2.594091 2.214731
C -1.218813 -1.436147 4.356783
H -1.424905 -1.372014 5.427584
C -2.122086 -0.917611 3.433740
H -3.045803 -0.438896 3.771927
C -3.410195 1.304484 1.395610
C -2.762460 3.535057 2.089597
H -1.978246 4.263333 2.305589
C -2.416473 2.255145 1.666479
H -1.358970 1.994823 1.563530
C -4.107041 3.875448 2.251660
H -4.379129 4.880873 2.581772

C -4.750739 1.645537 1.584066
H -5.518793 0.891746 1.399198
C -5.099371 2.928781 2.006217
H -6.151568 3.187546 2.146656
C 1.155743 -2.440449 -3.496984
H 2.128671 -2.133095 -3.086263
H 0.702307 -1.574683 -3.998361
H 1.340743 -3.226928 -4.246345
C -0.029899 -4.049004 -0.207096
H 0.416538 -4.524726 0.668239
C -1.443091 -3.812340 -0.237638
C -1.958721 -3.124381 -1.367819
H -3.010235 -2.839461 -1.380866
C -1.128440 -2.751880 -2.471604
H -1.577973 -2.212678 -3.304667
C 0.254697 -2.945936 -2.416884
C 0.795051 -3.586807 -1.247692
H 1.876264 -3.722878 -1.171558
C -0.042514 -2.030758 3.898350
H 0.680198 -2.432510 4.613904
C -2.342891 -4.240794 0.874951
H -2.770380 -5.230874 0.647236
H -1.796971 -4.306850 1.825898
H -3.165538 -3.518493 0.988216
C -3.136126 1.833258 -1.926391
H -2.459205 2.578805 -1.493889
H -3.417597 2.118721 -2.948674
H -4.027467 1.695124 -1.303075
P 1.387972 -0.196298 -0.652878
C 1.210948 1.607978 -0.391985
C 1.493390 2.185240 0.853011
C 0.751413 2.412634 -1.438188
C 1.314390 3.551904 1.045427
H 1.851590 1.565381 1.677943
C 0.576235 3.782604 -1.232775
H 0.484945 1.957244 -2.395321
C 0.858216 4.357591 0.002066
H 0.730773 5.431004 0.150881
C 3.141558 -0.544231 -0.287227
C 4.170945 0.336346 -0.636889
C 3.470881 -1.778939 0.274641

C 5.499615 -0.020841 -0.415817
H 3.942304 1.306081 -1.084496
C 4.800623 -2.134146 0.478438
H 2.673711 -2.470819 0.556690
C 5.827408 -1.259726 0.134959
H 6.869433 -1.541013 0.289126
Si -5.721809 -1.566729 -0.223685
C -6.336669 -0.147506 -1.306934
H -6.426417 0.804007 -0.759496
H -5.639492 -0.008247 -2.147004
H -7.329744 -0.386094 -1.721720
C -6.987920 -1.874927 1.141724
H -7.101395 -0.992204 1.791866
H -7.980258 -2.119466 0.729462
H -6.665584 -2.712650 1.780430
C -5.654484 -3.098459 -1.319650
H -5.033010 -2.903634 -2.207771
H -5.239713 -3.965140 -0.780819
H -6.662829 -3.371457 -1.670267
C 5.083087 -3.490727 1.064118
C 6.595907 0.962339 -0.734514
C 1.551995 4.159293 2.402286
C 0.029051 4.633530 -2.349107
F -1.310546 4.579560 -2.379612
F 0.365852 5.915570 -2.200477
F 0.467752 4.226292 -3.537230
F 0.414516 4.204940 3.111538
F 2.004373 5.409692 2.307149
F 2.430636 3.454239 3.112454
F 6.249794 1.776400 -1.733674
F 6.875893 1.729371 0.323266
F 7.723869 0.341579 -1.083930
F 6.384222 -3.707015 1.236221
F 4.610353 -4.460142 0.267626
F 4.481095 -3.639984 2.248714

Table S78. Cartesian coordinates of **3’**
Ru 0.118521 -1.695543 -1.111510
O 1.532216 -2.610140 2.226603
S 0.825232 -1.330338 2.074306
P 2.840240 -0.410140 -0.048980

N 3.805161 -1.630225 -0.008188

C 1.099740 -0.628828 0.480173

H 0.666194 0.375608 0.576790

P -1.387356 -0.006000 -1.242864

C 3.507227 1.084544 0.780445

O -0.630585 -1.319164 2.326725

C 2.694977 2.170916 1.121448

H 1.624791 2.137025 0.916702

C 3.236146 3.299688 1.728614

H 2.584354 4.131088 2.008148

Si 4.836520 -2.553540 1.001690

C 5.427441 2.292229 1.631527

H 6.501767 2.338948 1.824947

C 4.880267 1.157808 1.032456

H 5.516065 0.315679 0.751248

C 2.424717 0.156913 -1.716759

C 1.241984 -0.390323 -2.253022

C 0.937038 -0.046213 -3.582082

H 0.034143 -0.446205 -4.055540

C 1.749736 0.817958 -4.319256

H 1.476152 1.071961 -5.347130

C 2.897089 1.371594 -3.749131

H 3.518508 2.065760 -4.318735

C 3.239022 1.029930 -2.443389

H 4.136459 1.450415 -1.980989

C 4.830781 -2.022816 2.813464

H 5.069109 -0.955440 2.943341

H 5.569912 -2.603955 3.388699

H 3.835740 -2.219840 3.240544

H -1.761804 0.353242 -2.567376

C 4.352532 -4.374648 0.963114

H 3.379290 -4.520707 1.457256

H 5.097029 -4.982717 1.501913

H 4.289433 -4.758809 -0.067562

C 6.599373 -2.420910 0.335596

H 6.629703 -2.717696 -0.724935

H 7.289991 -3.073931 0.893486

H 6.989154 -1.392057 0.399854

C 2.617243 -3.298463 -2.733718

H 2.564474 -2.689220 -3.646303

H 2.960201 -4.309099 -3.008119

H 3.349897 -2.846722 -2.046807
C 1.286134 -3.378413 -2.059353
C 1.188316 -3.612324 -0.674003
H 2.094731 -3.669157 -0.074265
C -0.080054 -3.698254 -0.020382
H -0.098516 -3.864716 1.056345
C -1.280330 -3.533442 -0.726697
C -1.181223 -3.203308 -2.118623
H -2.094099 -3.005908 -2.686306
C 0.065227 -3.150275 -2.775405
H 0.107279 -2.901029 -3.836995
C -3.057907 -0.198093 -0.527247
C -3.182989 -0.639378 0.792314
H -2.286222 -0.890508 1.375516
C -4.452195 -0.781394 1.353567
C -5.595209 -0.515881 0.606437
H -6.583714 -0.647344 1.048126
C -5.465023 -0.091795 -0.715911
C -4.203001 0.074149 -1.281502
H -4.118749 0.410709 -2.317460
C -0.894048 1.668223 -0.658450
C -1.165588 2.096254 0.642691
H -1.712956 1.455401 1.338064
C -0.706383 3.340800 1.078929
C 0.017433 4.168950 0.227089
H 0.374254 5.139912 0.572166
C 0.312078 3.726708 -1.060926
C -0.138583 2.487576 -1.505845
H 0.117895 2.154814 -2.513565
C 4.606139 3.361058 1.986304
H 5.033734 4.245826 2.463842
C -2.607203 -3.708847 -0.061715
H -3.389540 -3.111085 -0.548777
H -2.911619 -4.766756 -0.122752
H -2.562782 -3.431472 0.999007
C 1.514682 -0.208320 3.277547
H 2.596069 -0.112375 3.123315
H 1.008016 0.760989 3.198531
H 1.301770 -0.675126 4.248529
C 1.205700 4.558147 -1.943607
C -0.991754 3.766193 2.494129

C -6.694925 0.239162 -1.518853
C -4.556516 -1.260813 2.778527
F 2.492619 4.286671 -1.709760
F 0.976256 4.329068 -3.236729
F 1.030886 5.863857 -1.721399
F -0.209997 4.781729 2.869904
F -2.257856 4.152338 2.650678
F -0.779188 2.756051 3.346985
F -7.050044 1.516958 -1.358469
F -6.491259 0.046338 -2.825060
F -7.735595 -0.510755 -1.152648
F -5.820370 -1.263865 3.208850
F -3.848259 -0.486743 3.599082
F -4.089088 -2.507750 2.902848

Table S79. Cartesian coordinates of **TS2**
Ru -0.956466 -1.134994 -1.866777
Si 0.672207 0.343711 2.939059
P -1.624094 -0.624743 1.226311
O -4.474240 -1.429780 -0.733589
S -3.891167 -0.087020 -0.902924
N -0.134178 -0.071467 1.441467
H -1.951793 0.999309 -0.408360
C -2.180880 -0.078675 -0.431966
C -2.737064 0.111695 2.466118
O -3.963562 0.547785 -2.226367
C -2.718376 1.510302 2.570876
H -2.037322 2.091097 1.943470
C -3.559729 2.158634 3.467635
H -3.537831 3.248389 3.541719
C -4.443694 0.026835 4.178077
H -5.115252 -0.557178 4.811589
C -3.599643 -0.627963 3.280741
H -3.611237 -1.717699 3.219032
C -1.724020 -2.426044 1.447122
C -2.910370 -4.528907 1.529226
H -3.820920 -5.106504 1.352447
C -2.890751 -3.169378 1.221885
H -3.766293 -2.686525 0.780877
C -1.778403 -5.153526 2.056849
H -1.804479 -6.218765 2.298663

C 0.454038 2.161637 3.411381
H 0.323138 2.819404 2.542027
H 1.331147 2.521496 3.972420
H -0.429298 2.283259 4.056687
C 0.058183 -0.649508 4.422424
H -1.016148 -0.489782 4.600511
H 0.598262 -0.308224 5.321288
H 0.229505 -1.730996 4.319567
C -0.584005 -3.061291 1.950706
H 0.327910 -2.479668 2.093704
C -0.609888 -4.420291 2.256598
H 0.291211 -4.903730 2.640780
C 2.509878 -0.033516 2.767675
H 3.008657 0.500341 1.945210
H 2.675796 -1.109986 2.609824
H 3.020284 0.250311 3.702221
C -2.250655 -0.294107 -4.911932
H -3.089140 0.232336 -4.436043
H -2.607398 -0.752710 -5.849565
H -1.474344 0.440441 -5.168717
C 0.169222 -2.637122 -3.020205
H 1.245071 -2.816546 -2.962396
C -0.707044 -3.365826 -2.165122
C -2.089009 -3.047684 -2.222868
H -2.792062 -3.538976 -1.550276
C -2.573553 -2.067549 -3.127976
H -3.631315 -1.808920 -3.098043
C -1.715865 -1.338276 -3.984432
C -0.321504 -1.628028 -3.888415
H 0.385120 -1.046819 -4.483663
C -4.427990 1.417250 4.271483
H -5.089787 1.926015 4.976160
C -0.203854 -4.480850 -1.307803
H -0.808610 -4.588048 -0.400181
H -0.263107 -5.430720 -1.865473
H 0.845770 -4.337526 -1.020008
C -4.831336 1.006436 0.151382
H -4.374680 2.003522 0.133928
H -5.819845 1.035183 -0.326283
H -4.901327 0.607250 1.168418
P 0.564844 0.039460 -0.903852

C 0.545670 1.845680 -0.686292
C 1.422848 2.553411 0.144673
C -0.433585 2.551891 -1.391886
C 1.311352 3.933446 0.269374
H 2.183853 2.029318 0.722303
C -0.582985 3.925806 -1.208007
H -1.094591 2.004426 -2.073019
C 0.294774 4.628916 -0.388722
H 0.187919 5.707710 -0.264854
C 2.283144 -0.480146 -0.675766
C 3.426483 0.278840 -0.945135
C 2.431034 -1.778689 -0.177117
C 4.690226 -0.252289 -0.692943
H 3.339677 1.287428 -1.354736
C 3.695525 -2.301918 0.071132
H 1.530482 -2.360836 0.036034
C 4.834748 -1.540651 -0.178537
H 5.826381 -1.944542 0.026617
C 5.902936 0.611093 -0.916097
C 3.792506 -3.684016 0.654550
C 2.259020 4.702315 1.150964
C -1.748482 4.614740 -1.863635
F -2.892255 4.302516 -1.234210
F -1.888465 4.246511 -3.136119
F -1.629562 5.942952 -1.830558
F 1.592303 5.421861 2.059872
F 2.996765 5.559103 0.439232
F 3.095581 3.897605 1.807555
F 7.013047 -0.116391 -1.050272
F 5.771585 1.363384 -2.011291
F 6.092759 1.443637 0.112779
F 3.100005 -3.770966 1.801067
F 3.277183 -4.602552 -0.173201
F 5.048374 -4.037993 0.915218

Table S80. Cartesian coordinates of **Int2**
Ru 1.017828 0.042144 -1.801735
Si -0.144798 -0.115675 3.056135
P 2.091983 -0.333669 0.850350
O 4.532652 -1.054717 -1.521899
S 3.501256 -2.083654 -1.304146

N 0.487097 -0.123736 1.355637
H 1.435133 -2.265471 -0.321657
C 2.052380 -1.387540 -0.580321
C 2.815197 -1.290087 2.243226
O 3.063534 -2.888187 -2.446672
C 2.259935 -2.543391 2.544438
H 1.357080 -2.878444 2.027343
C 2.846107 -3.364411 3.501298
H 2.397990 -4.333170 3.734916
C 4.575264 -1.716613 3.852224
H 5.484587 -1.390354 4.362291
C 3.982575 -0.886767 2.900395
H 4.426325 0.084992 2.674270
C 3.005560 1.218243 0.837342
C 4.981531 2.537725 0.406246
H 5.976293 2.623967 -0.037490
C 4.288521 1.336195 0.284797
H 4.720887 0.497047 -0.263085
C 4.416656 3.626309 1.070966
H 4.964700 4.567820 1.150525
C -0.891508 -1.786345 3.506283
H -1.051384 -2.422826 2.625093
H -1.866732 -1.667096 4.002550
H -0.223161 -2.323609 4.195904
C 1.163771 0.324011 4.330195
H 1.804254 -0.527472 4.594189
H 0.619713 0.632510 5.238861
H 1.809353 1.161171 4.029218
C 2.454384 2.302318 1.538704
H 1.469259 2.210074 1.995994
C 3.147791 3.504745 1.636702
H 2.687922 4.348149 2.155971
C -1.423381 1.257200 3.179169
H -2.289960 1.171924 2.511427
H -0.965421 2.240806 2.991292
H -1.800937 1.256445 4.214970
C -1.326232 -0.788102 -4.149443
H -1.204199 -1.879069 -4.106872
H -1.632569 -0.521482 -5.174836
H -2.149207 -0.510334 -3.474471
C 1.080966 1.961639 -2.892090

H 1.000344 2.973518 -2.487555
C 2.360298 1.381581 -3.111264
C 2.383952 0.048904 -3.600964
H 3.346307 -0.457420 -3.679306
C 1.209813 -0.671050 -3.960754
H 1.302482 -1.696673 -4.319000
C -0.059736 -0.080832 -3.775279
C -0.099020 1.246374 -3.236386
H -1.070670 1.706832 -3.039784
C 4.007569 -2.951938 4.158497
H 4.470861 -3.597582 4.908069
C 3.625852 2.135432 -2.865260
H 4.429620 1.448501 -2.568021
H 3.948038 2.658637 -3.781871
H 3.496552 2.885927 -2.073321
C 4.210352 -3.238933 -0.138828
H 3.464492 -4.000271 0.119211
H 5.050759 -3.695461 -0.678587
H 4.562611 -2.708136 0.753677
P -0.407338 0.068998 -0.167108
C -1.676035 -1.277544 -0.001610
C -2.757843 -1.310733 0.882692
C -1.481360 -2.359156 -0.863581
C -3.621452 -2.404428 0.903624
H -2.946057 -0.483903 1.568759
C -2.338168 -3.458650 -0.831199
H -0.640788 -2.300371 -1.568698
C -3.415688 -3.486958 0.049888
H -4.094311 -4.340223 0.066581
C -1.396328 1.605042 -0.016327
C -2.770936 1.705287 -0.233027
C -0.651759 2.776189 0.146343
C -3.390812 2.958032 -0.239953
H -3.376580 0.812738 -0.399728
C -1.277501 4.016601 0.172187
H 0.435118 2.704809 0.224663
C -2.655567 4.118329 -0.017262
H -3.147156 5.091354 -0.003973
C -4.865156 3.035808 -0.531380
C -0.428953 5.229509 0.431939
C -4.745333 -2.420672 1.903388

C -2.077944 -4.601258 -1.776269
F -2.939550 -5.604092 -1.597140
F -0.846070 -5.089734 -1.610497
F -2.176333 -4.209380 -3.051174
F -4.296640 -2.726346 3.129267
F -5.681786 -3.315426 1.590237
F -5.332627 -1.224505 1.993078
F -5.106054 2.908383 -1.840594
F -5.533808 2.057802 0.086269
F -5.389311 4.198372 -0.140405
F -1.114058 6.364685 0.308638
F 0.078232 5.199281 1.675570
F 0.611971 5.285939 -0.405067

Table S81. Cartesian coordinates of **TS3**
Ru -1.091283 -0.650571 -1.437344
Si 0.158236 -0.233099 3.236632
P -2.118592 -0.039780 0.557284
O -4.769437 -0.349532 -1.716455
S -4.002358 0.898114 -1.889192
N -0.357858 -0.341273 1.538715
H -2.189767 1.912915 -0.940635
C -2.526891 0.868866 -0.976178
C -2.419354 1.385507 1.710454
O -3.749284 1.353141 -3.264384
C -1.823218 2.633822 1.476479
H -1.123851 2.766199 0.649006
C -2.100000 3.721589 2.296416
H -1.619580 4.682365 2.096492
C -3.565915 2.349715 3.621957
H -4.250349 2.226446 4.464909
C -3.289361 1.255934 2.797083
H -3.765033 0.296510 3.008079
C -3.237794 -1.347308 1.134951
C -5.418572 -2.318081 1.511037
H -6.506813 -2.234007 1.468106
C -4.631725 -1.243091 1.100790
H -5.110363 -0.338847 0.728990
C -4.824762 -3.492985 1.968375
H -5.447183 -4.330022 2.293508
C 0.203138 1.510279 3.937478

H 0.786454 2.215828 3.329182
H 0.664772 1.473388 4.938193
H -0.808311 1.925275 4.049526
C -1.029693 -1.254219 4.269855
H -2.073136 -0.939253 4.129391
H -0.780403 -1.131341 5.336175
H -0.964273 -2.325371 4.027734
C -2.645804 -2.534904 1.579915
H -1.557001 -2.606306 1.584820
C -3.434006 -3.599580 2.004549
H -2.961181 -4.518581 2.358977
C 1.879155 -0.982778 3.453991
H 2.658924 -0.518046 2.831538
H 1.890624 -2.062236 3.242730
H 2.176612 -0.846582 4.506892
C 1.220153 -0.254207 -4.014309
H 0.965171 0.798255 -4.202230
H 1.518569 -0.705949 -4.974739
H 2.097543 -0.287913 -3.352350
C -0.780663 -2.811167 -1.909641
H -0.549980 -3.626757 -1.221569
C -2.127190 -2.530778 -2.279044
C -2.336162 -1.457516 -3.173653
H -3.359489 -1.178008 -3.418485
C -1.270825 -0.687586 -3.729756
H -1.520089 0.159936 -4.369586
C 0.062316 -0.997662 -3.423526
C 0.273014 -2.055999 -2.482709
H 1.297992 -2.279016 -2.182470
C -2.976933 3.585678 3.374381
H -3.194114 4.440063 4.019329
C -3.283557 -3.323350 -1.763767
H -4.149006 -2.663599 -1.611218
H -3.561929 -4.099803 -2.494965
H -3.040395 -3.817515 -0.814461
C -4.953855 2.194137 -1.103126
H -4.421112 3.146119 -1.217548
H -5.914326 2.224255 -1.634324
H -5.095576 1.952920 -0.041774
P 0.469380 -0.129985 0.106199
C 1.262694 1.528067 0.016257

C 2.166578 2.023621 0.959812
C 0.854787 2.348740 -1.034622
C 2.626739 3.334859 0.863142
H 2.518965 1.391543 1.777444
C 1.323074 3.658451 -1.131189
H 0.144756 1.937359 -1.763141
C 2.205877 4.160809 -0.180612
H 2.570919 5.185851 -0.253480
C 1.914593 -1.249302 0.106037
C 3.130941 -0.926189 -0.493160
C 1.710348 -2.546233 0.581246
C 4.125640 -1.897332 -0.625412
H 3.306012 0.083534 -0.872799
C 2.709127 -3.505352 0.459543
H 0.750205 -2.797414 1.037937
C 3.924490 -3.189195 -0.148029
H 4.703795 -3.944276 -0.251831
C 3.538183 3.871276 1.934420
C 0.813490 4.512971 -2.259516
C 5.398914 -1.524166 -1.335686
C 2.442273 -4.881671 1.003742
F 1.014577 3.924051 -3.442101
F -0.503742 4.715490 -2.141287
F 1.404645 5.707005 -2.293003
F 2.841320 4.229962 3.019763
F 4.212673 4.944971 1.522591
F 4.424938 2.952885 2.323886
F 5.162150 -1.269395 -2.629040
F 5.939291 -0.420415 -0.814394
F 6.310324 -2.493908 -1.276905
F 1.307110 -5.384095 0.499953
F 2.294929 -4.853492 2.333356
F 3.423819 -5.735809 0.722913

Table S82. Cartesian coordinates of **4’**
C 4.275855 -0.923010 -0.611338
F -6.361529 0.790287 -2.305472
P -0.570270 -0.059558 0.503798
O 2.969706 -4.054670 -0.187274
Ru 0.666030 -1.366246 -1.062152
F -4.486289 1.015345 -3.345115

Si -1.023346 -0.600185 3.583964

P 2.562343 -0.560786 -0.100885

O 1.253337 -4.265365 1.644887

S 2.329403 -3.491770 1.012699

C 1.710557 -1.883823 0.727488

H 1.120480 -1.563909 1.612056

C 0.535639 4.057981 -2.674129

C -0.233760 4.951770 2.198953

C -0.410026 2.632109 1.247967

H -0.580034 2.232102 2.249593

C -0.198246 3.991166 1.042151

C 0.069190 4.484629 -0.236622

H 0.253149 5.548456 -0.394093

C 0.138535 3.595521 -1.303029

C -0.063551 2.230327 -1.097189

H 0.047728 1.526066 -1.923695

C -0.351033 1.744031 0.172811

C -4.961223 -2.875775 0.927771

C -5.080686 1.120586 -2.146464

C -3.065903 0.555167 -0.797837

H -2.591882 1.437976 -1.233624

C -4.386938 0.250778 -1.134823

C -5.027135 -0.851764 -0.577614

H -6.059846 -1.083102 -0.838511

C -4.325948 -1.654089 0.322982

C -3.004955 -1.363193 0.649017

H -2.451953 -2.007664 1.337507

C 2.926871 2.159793 0.223746

H 2.984132 2.185321 -0.867809

F -1.357299 5.674265 2.206398

C 4.555365 -1.966231 -1.503565

H 3.746991 -2.590347 -1.874914

F -5.029156 2.409242 -1.802615

C 6.919371 -1.489829 -1.350642

H 7.949344 -1.710555 -1.640571

F -4.809275 -2.897888 2.254419

C 5.869036 -2.247423 -1.866525

H 6.071337 -3.071874 -2.553804

F -6.265855 -2.947514 0.665140

C 6.650219 -0.453439 -0.458981

H 7.466745 0.142025 -0.044171

F -4.395270 -3.994795 0.451620
C 5.337708 -0.170029 -0.087782
H 5.140465 0.641696 0.614228
F -0.151778 4.323987 3.372391
C 2.705342 0.946806 0.891389
F 0.785793 5.821021 2.135818
C 3.062258 3.338391 0.949789
H 3.227453 4.282501 0.426319
C 2.965132 3.315700 2.342246
H 3.052292 4.245166 2.908742
C 2.731049 2.113411 3.005287
H 2.636850 2.094306 4.092988
C 2.601835 0.927721 2.283837
H 2.398487 -0.009268 2.801692
C 0.150281 0.106750 4.883584
H -0.248972 -0.039571 5.900023
H 0.298071 1.187559 4.729158
H 1.137362 -0.381525 4.840012
C -1.218860 -2.450309 3.903656
H -1.345279 -2.661693 4.977889
H -0.342848 -3.009848 3.537398
H -2.104828 -2.857514 3.389785
C -2.705407 0.228591 3.823738
H -3.468644 -0.212178 3.163741
H -2.659257 1.308760 3.610282
H -3.054798 0.106153 4.862054
C -0.344817 -3.273226 -1.761847
C -1.197873 -2.185971 -2.033994
H -2.208631 -2.198581 -1.628616
C -0.760716 -1.043865 -2.767976
H -1.456255 -0.217484 -2.926460
C 0.548262 -0.972566 -3.290263
C 1.403122 -2.094251 -3.051599
H 2.427289 -2.055926 -3.426235
C 0.982341 -3.205303 -2.298234
H 1.691496 -3.998394 -2.057886
N -0.349917 -0.318340 2.041720
C -2.358830 -0.261862 0.081525
F 0.511249 5.383020 -2.795343
F 1.781574 3.651482 -2.974779
F -0.266897 3.546639 -3.617799

C -0.808778 -4.459329 -0.978671
H -0.925969 -5.327759 -1.646617
H -0.086289 -4.719852 -0.192180
H -1.778679 -4.260488 -0.503349
C 1.017842 0.181568 -4.120792
H 2.025235 0.504840 -3.823667
H 1.058230 -0.112558 -5.182107
H 0.341641 1.043010 -4.045605
C 3.620541 -3.289326 2.227665
H 3.192344 -2.823613 3.124166
H 3.982411 -4.301115 2.454438
H 4.422278 -2.676473 1.795099

4.2.5 2b' + $HPPh_2$

Table S83. SCF energies, enthalpy and free energy corrections and barriers

	E_{SCF}/E_H	corrH/E_H	corrG/E_H	ΔH/kJ/mol	ΔG/kJ/mol
**TS-Act1**	-3130.458715	0.741003	0.60946	-36.23141691	31.37520809
**Coord**	-3130.492666	0.74236	0.61304	-121.8081191	-48.36500763
**TS-Act2**	-3130.467819	0.738779	0.609806	-65.97245079	8.381709213
**Act'**	-3130.504516	0.743995	0.616687	-148.6277854	-69.90216791
**TS-Act3**	-3130.500443	0.743375	0.618587	-139.5615401	-54.21966259
**Act**	-3130.506418	0.743976	0.614715	-153.672001	-80.07398503
**TS1**	-3130.483866	0.743044	0.614424	-96.90764083	-21.62667933
**Int1**	-3130.486189	0.744034	0.61342	-100.4066972	-30.36098269
**TS4**	-3130.467374	0.743228	0.614831	-53.1246453	22.7418027
**Int3**	-3130.475908	0.742763	0.614833	-76.75188737	0.340669128
**TS5**	-3130.468285	0.738146	0.610295	-68.85892549	8.441045512
**Int4**	-3130.473701	0.740318	0.611134	-77.3753649	-3.57518536
**TS6**	-3130.464946	0.739196	0.609237	-57.33691874	14.42849826
**3'**	-3130.518457	0.741961	0.612826	-190.5707518	-116.6419228
**TS2**	-3130.47476	0.742726	0.615759	-73.83374914	5.787163857
**Int2**	-3130.490762	0.743378	0.614523	-114.1368807	-39.47291172
**TS3**	-3130.483521	0.742738	0.616441	-96.80522008	-15.42522208
**4'**	-3130.519623	0.74391	0.61435	-188.5140401	-115.7010486

Table S84. Cartesian coordinates of **TS-Act1**
Ru 0.880812 -0.727251 -1.450633
N -1.056028 -0.568825 -0.551920
O 3.372047 -2.652881 0.344213
P -0.672890 -1.519498 0.747734
S 2.145263 -2.535776 1.162486

C 1.000649 -1.584633 0.338483

C -1.069049 -0.728917 2.329349

O 1.539345 -3.774249 1.689606

C -1.395135 0.630605 2.388051

H -1.539479 1.201964 1.469710

C -1.351569 0.535264 4.799790

H -1.461407 1.029769 5.767903

C -1.029815 -0.822425 4.745183

H -0.886553 -1.389675 5.667829

C -0.887613 -1.458005 3.514297

H -0.620510 -2.518225 3.466281

C -1.557637 -3.105421 0.766602

C -2.765308 -3.276904 1.453749

H -3.167755 -2.470126 2.072000

C -3.451120 -4.486010 1.367963

H -4.392799 -4.615785 1.906133

C -2.931766 -5.528540 0.601242

H -3.469009 -6.477879 0.537594

C -1.720155 -5.366620 -0.070347

H -1.300124 -6.191248 -0.650662

C -1.031226 -4.160762 0.014487

H -0.061984 -4.034297 -0.467738

C 1.876582 -3.669920 -2.491269

H 2.376961 -3.852623 -1.530476

H 2.483223 -4.125604 -3.290858

H 0.893771 -4.160464 -2.494502

C 1.746814 -2.201007 -2.734001

C 2.776747 -1.300787 -2.327292

H 3.611686 -1.684083 -1.739432

C 2.599192 0.090563 -2.489522

H 3.337736 0.771454 -2.062376

C 1.462909 0.642405 -3.165653

C 0.482767 -0.257895 -3.631538

H -0.437142 0.124639 -4.076998

C 0.593379 -1.647983 -3.373753

H -0.239347 -2.307182 -3.626503

C -1.534370 1.260201 3.623140

H -1.781793 2.323566 3.658378

C 2.635740 -1.620242 2.623549

H 3.344634 -2.271176 3.153491

H 3.117140 -0.678527 2.335410

H 1.749890 -1.432755 3.243204
C 1.344603 2.121945 -3.353452
H 1.950319 2.445351 -4.215765
H 0.306293 2.427745 -3.542923
H 1.711481 2.661514 -2.468492
P 1.300721 1.713506 0.372509
H 1.293583 0.949783 1.568887
C 0.502529 3.237824 1.019258
C 0.864638 3.822801 2.241584
C -0.576769 3.768709 0.303865
C 0.159424 4.917448 2.733227
H 1.703483 3.413504 2.810904
C -1.286006 4.864308 0.798375
H -0.881744 3.302762 -0.636733
C -0.918277 5.438637 2.012761
H 0.447486 5.365910 3.687317
H -2.132525 5.262477 0.233993
H -1.473684 6.294679 2.403565
C 3.085383 2.138251 0.341529
C 3.538093 3.452363 0.173239
C 4.024489 1.092879 0.355446
C 4.901142 3.718223 0.038379
H 2.821015 4.277159 0.157313
C 5.384266 1.364734 0.230411
H 3.693483 0.053076 0.436940
C 5.828008 2.678567 0.071076
H 5.239197 4.749654 -0.088914
H 6.100191 0.539440 0.252154
H 6.895063 2.889824 -0.029966
C -2.198400 0.106878 -0.849296
C -3.437736 -0.125509 -0.200218
C -2.160840 1.129605 -1.829967
C -4.558617 0.625618 -0.503647
H -3.513483 -0.914431 0.547180
C -3.278948 1.880963 -2.136659
H -1.206261 1.330171 -2.313610
C -4.479109 1.629604 -1.469252
H -5.512219 0.450097 -0.005166
H -3.248320 2.678279 -2.880091
N -5.650405 2.425657 -1.777258
O -5.537186 3.297825 -2.617351

O -6.677976 2.179791 -1.176631

Table S85. Cartesian coordinates of **Coord**

Ru 0.601637 0.358103 -1.317053
N -1.105833 -0.191850 -0.152177
O 3.354462 -2.144477 -1.060533
P -0.520697 -1.662981 0.375497
S 2.301280 -2.464738 -0.068172
C 1.069910 -1.320716 -0.132843
C -0.751469 -1.929676 2.157753
O 1.720697 -3.828851 -0.082068
C -1.206438 -0.917686 3.010017
H -1.553395 0.031159 2.594857
C -0.800392 -2.343396 4.917697
H -0.823005 -2.506253 5.997871
C -0.338846 -3.351718 4.070129
H 0.005264 -4.302014 4.485140
C -0.315983 -3.151832 2.692107
H 0.059168 -3.931848 2.021903
C -1.397274 -3.067274 -0.389263
C -2.511715 -3.676644 0.197726
H -2.847098 -3.372583 1.192835
C -3.185416 -4.695149 -0.472907
H -4.054633 -5.168124 -0.010134
C -2.744202 -5.114704 -1.727308
H -3.271316 -5.916261 -2.250434
C -1.617852 -4.527204 -2.303644
H -1.251309 -4.877589 -3.271557
C -0.943229 -3.511533 -1.633740
H -0.029555 -3.078919 -2.038442
C 1.589044 -2.007710 -3.745737
H 2.151399 -2.468102 -2.918872
H 2.265801 -1.955596 -4.613378
H 0.731340 -2.633911 -4.021247
C 1.167710 -0.628408 -3.365874
C 2.142428 0.298700 -2.870534
H 3.166144 -0.051332 -2.735663
C 1.794913 1.644913 -2.595042
H 2.567443 2.337917 -2.252200
C 0.454169 2.118128 -2.753403
C -0.514631 1.139354 -3.065423

H -1.569360 1.422236 -3.071852
C -0.165078 -0.203409 -3.416887
H -0.952086 -0.910965 -3.683271
C -1.234970 -1.129281 4.387468
H -1.603129 -0.342060 5.049565
C 3.119882 -2.397726 1.526501
H 3.855346 -3.213430 1.521311
H 3.612647 -1.429947 1.661891
H 2.365925 -2.566276 2.306148
C 0.103022 3.560618 -2.574122
H 0.327128 4.118267 -3.498283
H -0.967401 3.690973 -2.364387
H 0.675604 4.019870 -1.757000
P 1.336579 1.253236 0.621605
H 0.632030 0.792240 1.757719
C 1.095839 3.063927 0.799407
C -0.157600 3.555273 1.183597
C 2.111219 3.967280 0.462617
C -0.397207 4.927051 1.214157
H -0.958530 2.861234 1.451341
C 1.869398 5.340225 0.494095
H 3.101271 3.594953 0.185285
C 0.614287 5.822120 0.864328
H 0.425575 6.897900 0.888016
C 3.075698 1.068462 1.122833
C 3.451966 1.393836 2.433021
C 4.040736 0.632409 0.213578
C 4.780832 1.273377 2.827122
H 2.700267 1.738573 3.148517
C 5.373357 0.520216 0.609327
H 3.737289 0.313288 -0.782032
C 5.744140 0.839409 1.913337
H 6.786643 0.741828 2.225495
C -2.334154 0.376809 -0.023061
C -3.450515 -0.288519 0.547635
C -2.528661 1.717432 -0.444823
C -4.671771 0.345273 0.685590
H -3.351376 -1.320691 0.878419
C -3.746404 2.353967 -0.304509
H -1.672229 2.248669 -0.857753
C -4.821695 1.665734 0.260357

H -5.529853 -0.167476 1.120939
H -3.889235 3.388968 -0.617127
N -6.099576 2.330662 0.411473
O -6.187774 3.481864 0.028607
O -7.010774 1.702565 0.914309
H -1.380106 5.298037 1.514454
H 2.668326 6.037753 0.231235
H 5.067987 1.516605 3.852748
H 6.116950 0.156276 -0.102773

Table S86. Cartesian coordinates of **TS-Act2**
Ru -1.146069 -0.050455 -1.088519
N 0.792759 0.369578 -0.264799
O -3.923252 1.161447 0.893430
P 0.274208 1.688664 0.582245
S -2.748855 2.038624 1.078458
C -1.354236 1.148587 0.685312
C 1.078947 1.962921 2.191362
O -2.829371 3.371918 0.439478
C 0.754566 1.090085 3.239919
H 0.006057 0.310417 3.074926
C 2.350798 2.205639 4.665253
H 2.849889 2.300353 5.632610
C 2.673678 3.078671 3.627470
H 3.424581 3.857195 3.780016
C 2.041504 2.959103 2.391083
H 2.299304 3.638778 1.575855
C 0.680852 3.173066 -0.387478
C 1.629489 3.091480 -1.413514
H 2.128918 2.143127 -1.621623
C 1.931865 4.221131 -2.172051
H 2.674796 4.155235 -2.970435
C 1.286608 5.429915 -1.912062
H 1.520818 6.313592 -2.510597
C 0.337315 5.509618 -0.892839
H -0.181609 6.451122 -0.698994
C 0.031535 4.385927 -0.129225
H -0.754674 4.438749 0.626356
C -1.851251 2.927146 -2.713967
H -2.221302 3.335309 -1.761991
H -2.516501 3.277877 -3.520308

H -0.844358 3.321722 -2.904778

C -1.855444 1.433679 -2.672927

C -2.934031 0.726765 -2.043983

H -3.706713 1.283974 -1.511880

C -3.005016 -0.682694 -2.046267

H -3.834409 -1.179093 -1.540775

C -1.960651 -1.463221 -2.636979

C -0.860995 -0.777704 -3.196921

H -0.039290 -1.354342 -3.624889

C -0.810824 0.650986 -3.199301

H 0.075276 1.153814 -3.594381

C 1.390191 1.211249 4.471382

H 1.136911 0.527346 5.284759

C -2.639855 2.359847 2.834002

H -3.524046 2.961039 3.084246

H -2.650699 1.403312 3.370064

H -1.719777 2.919715 3.047480

C -2.054911 -2.955459 -2.669674

H -1.070017 -3.419630 -2.815872

H -2.495262 -3.348308 -1.743011

H -2.702376 -3.264746 -3.506354

P -1.128393 -1.584286 0.677042

H -1.251237 -0.225931 1.474335

C 0.363107 -2.602600 0.896843

C 0.888920 -2.785592 2.182845

C 1.047944 -3.140251 -0.198998

C 2.078859 -3.485735 2.366289

H 0.362978 -2.365471 3.044382

C 2.238907 -3.839567 -0.015780

H 0.653399 -2.981044 -1.204485

C 2.758463 -4.009119 1.266342

H 2.484860 -3.614143 3.372552

H 2.775626 -4.236071 -0.880739

H 3.702033 -4.541454 1.407182

C -2.546668 -2.704366 0.866822

C -2.407562 -4.098069 0.892733

C -3.824527 -2.133253 0.993808

C -3.532261 -4.911845 1.028376

H -1.415000 -4.547160 0.804707

C -4.940445 -2.954743 1.127857

H -3.936729 -1.042381 0.990997

C -4.800035 -4.344232 1.141686
H -3.414186 -5.998237 1.045047
H -5.930527 -2.502851 1.227812
H -5.679625 -4.983872 1.246998
C 2.048148 -0.134346 -0.475688
C 2.384954 -0.678838 -1.736607
C 3.031746 -0.181945 0.541861
C 3.614726 -1.267427 -1.967108
H 1.637932 -0.636065 -2.526633
C 4.261122 -0.773514 0.317314
H 2.799431 0.209414 1.531280
C 4.546599 -1.324427 -0.932312
H 3.873774 -1.696785 -2.935210
H 5.008705 -0.844807 1.107378
N 5.818057 -1.988228 -1.150139
O 6.613043 -2.007238 -0.231280
O 6.012836 -2.495375 -2.237944

Table S87. Cartesian coordinates of **Act'**
Ru -1.173501 -0.220620 -0.932703
P 0.849391 -1.548157 0.571615
O -0.962885 -4.114891 1.698543
S -1.525206 -3.388455 0.548642
N 0.846469 -0.112186 -0.222330
H -1.270174 -1.363097 1.578043
C -0.935024 -1.724691 0.591084
C 1.571120 -1.527461 2.235235
O -1.376270 -3.996329 -0.781529
C 0.983414 -0.684674 3.188919
H 0.102199 -0.088298 2.918613
C 1.529110 -0.599881 4.465982
H 1.069288 0.058577 5.206458
C 3.242072 -2.189571 3.850182
H 4.124844 -2.778572 4.108605
C 2.704350 -2.278901 2.568329
H 3.170796 -2.930730 1.826954
C 1.753244 -2.802411 -0.367671
C 2.452546 -5.077910 -0.755974
H 2.406247 -6.137631 -0.495878
C 1.707656 -4.160136 -0.021619
H 1.077838 -4.498370 0.804359

C 3.248373 -4.651063 -1.819439
H 3.832897 -5.377633 -2.388933
C 2.547300 -2.374193 -1.437182
H 2.576742 -1.316580 -1.705190
C 3.298770 -3.300013 -2.156992
H 3.922613 -2.962344 -2.987565
C -0.209935 -2.471880 -3.417872
H -0.234718 -3.298266 -2.694129
H 0.832418 -2.171177 -3.582196
H -0.607236 -2.843540 -4.376888
C -3.143030 -0.464243 -1.928719
H -4.112540 -0.661508 -1.469114
C -2.759460 0.879094 -2.179694
C -1.469373 1.116485 -2.715979
H -1.132693 2.142148 -2.873137
C -0.620799 0.025387 -3.050868
H 0.386815 0.235023 -3.418462
C -1.037183 -1.324458 -2.934646
C -2.285949 -1.535782 -2.291883
H -2.573911 -2.564769 -2.071820
C 2.656845 -1.351231 4.797751
H 3.082753 -1.282714 5.801614
C -3.739960 1.988065 -1.979783
H -4.347167 2.071804 -2.896265
H -4.417404 1.794116 -1.139658
H -3.250532 2.955602 -1.818291
C -3.270572 -3.207715 0.864114
H -3.728997 -2.604224 0.072547
H -3.666618 -4.231836 0.855973
H -3.410289 -2.749104 1.850582
P -1.488373 1.135397 1.067257
C -3.236078 1.659290 1.191831
C -4.215548 0.647824 1.190690
C -3.664656 2.977584 1.410119
C -5.564479 0.941762 1.354013
H -3.899738 -0.388136 1.045287
C -5.018270 3.273820 1.573093
H -2.931662 3.785850 1.449885
C -5.977670 2.264110 1.536053
H -7.036862 2.501026 1.659351
C -0.569367 2.712859 0.824099

C 0.566680 2.910267 1.622706
C -0.876098 3.694822 -0.129644
C 1.378220 4.032540 1.462742
H 0.827723 2.159278 2.372247
C -0.077085 4.824134 -0.285056
H -1.753683 3.568317 -0.764627
C 1.060559 4.993321 0.506134
H 1.702905 5.866127 0.367759
C 1.872730 0.779252 -0.402011
C 1.692607 1.875095 -1.274591
C 3.122876 0.661993 0.254115
C 2.679112 2.823849 -1.453717
H 0.728116 1.989223 -1.761946
C 4.115389 1.609620 0.074158
H 3.307416 -0.169889 0.933869
C 3.889444 2.693453 -0.772417
H 2.526995 3.686443 -2.101681
H 5.072624 1.532985 0.590178
N 4.910871 3.710340 -0.926390
O 5.966189 3.548288 -0.345284
O 4.651184 4.672600 -1.623085
H -0.335992 5.574388 -1.036878
H 2.269252 4.150545 2.083933
H -5.324485 4.311331 1.731324
H -6.302104 0.134666 1.340982

Table S88. Cartesian coordinates of **TS-Act3**
Ru -0.836124 -0.234997 -1.182495
P 1.130117 -1.285496 0.618978
O -0.290229 -4.221770 1.235876
S -0.750095 -3.521661 0.024882
N 1.011996 0.155378 -0.159196
H -1.139452 -1.611745 1.235536
C -0.576988 -1.778719 0.300474
C 1.525815 -1.160387 2.383891
O -0.167913 -3.920547 -1.265129
C 0.747673 -0.286716 3.156837
H -0.071442 0.271987 2.683359
C 1.024346 -0.133222 4.511625
H 0.416656 0.548667 5.110786
C 2.852981 -1.701706 4.328763

H 3.678552 -2.252185 4.785273

C 2.585290 -1.862622 2.971526

H 3.207473 -2.529501 2.372514

C 2.412519 -2.324038 -0.133252

C 3.621294 -4.400640 -0.368055

H 3.743146 -5.455298 -0.111730

C 2.582715 -3.674206 0.205845

H 1.887954 -4.161210 0.892883

C 4.494602 -3.792929 -1.270139

H 5.309001 -4.369692 -1.715059

C 3.284983 -1.717755 -1.043893

H 3.149622 -0.669669 -1.313623

C 4.326497 -2.451642 -1.606368

H 5.006501 -1.970583 -2.312913

C 1.109736 -1.844421 -3.588234

H 1.166612 -2.749139 -2.966985

H 2.047526 -1.283746 -3.488436

H 1.017371 -2.155656 -4.641929

C -2.461327 -0.837284 -2.573750

H -3.411147 -1.330742 -2.364067

C -2.406064 0.580144 -2.582046

C -1.157512 1.202699 -2.856439

H -1.083820 2.290880 -2.827455

C -0.013623 0.408233 -3.123622

H 0.949920 0.902679 -3.267931

C -0.067081 -1.010492 -3.196593

C -1.303431 -1.608307 -2.853796

H -1.342522 -2.696826 -2.787450

C 2.073885 -0.839678 5.098934

H 2.289780 -0.714067 6.162696

C -3.647659 1.384508 -2.387635

H -4.203701 1.414544 -3.339018

H -4.298962 0.936093 -1.624907

H -3.417423 2.414232 -2.091250

C -2.503611 -3.809993 -0.071860

H -2.914024 -3.328628 -0.966048

H -2.588531 -4.902537 -0.144820

H -2.988102 -3.442205 0.840307

P -1.758412 0.876883 0.776297

C -3.451780 0.290580 1.173369

C -4.055833 -0.806226 0.548017

C -4.129949 0.872606 2.262039
C -5.285185 -1.308008 0.979432
H -3.538521 -1.261819 -0.294745
C -5.363140 0.388043 2.683425
H -3.676041 1.719345 2.784583
C -5.948747 -0.709558 2.046631
H -5.730379 -2.167383 0.469811
H -5.870241 0.864252 3.526589
H -6.914227 -1.093347 2.384244
C -2.054492 2.672300 0.511101
C -0.942172 3.521933 0.635144
C -3.303658 3.266542 0.267881
C -1.055891 4.897494 0.455751
H 0.028369 3.093905 0.891948
C -3.423976 4.646233 0.108457
H -4.198216 2.643185 0.214856
C -2.299794 5.468009 0.184390
H -0.167978 5.528570 0.548251
H -4.408800 5.082660 -0.078866
H -2.395695 6.548512 0.053684
C 1.887383 1.207233 -0.129723
C 1.625026 2.359840 -0.906541
C 3.076605 1.201177 0.644053
C 2.493524 3.435513 -0.922229
H 0.694408 2.400695 -1.468366
C 3.946740 2.276563 0.631585
H 3.317984 0.339016 1.266727
C 3.657109 3.390882 -0.154934
H 2.284023 4.326787 -1.514200
H 4.860527 2.272874 1.226082
N 4.571485 4.517956 -0.169737
O 5.576845 4.439985 0.507960
O 4.278042 5.474516 -0.859409

Table S89. Cartesian coordinates of **Act**
Ru -0.790798 -0.036358 -1.191475
P 1.327481 -1.228028 0.281531
O 0.076447 -3.753727 -1.525658
S -0.821607 -3.328977 -0.445954
N 1.131301 0.293245 -0.278587
H -0.883359 -1.602386 1.087755

C -0.386969 -1.718762 0.112575
C 1.845269 -1.280716 2.017940
O -2.268274 -3.379551 -0.695958
C 1.141662 -0.439032 2.893073
H 0.362989 0.230508 2.503575
C 1.453471 -0.439875 4.248428
H 0.904515 0.216673 4.926952
C 3.178985 -2.088756 3.862529
H 3.983274 -2.723905 4.240572
C 2.874560 -2.095719 2.502425
H 3.451333 -2.723800 1.820425
C 2.549680 -2.129567 -0.701338
C 3.791955 -4.108230 -1.317069
H 3.986091 -5.176123 -1.197151
C 2.811096 -3.496021 -0.545127
H 2.242485 -4.097572 0.163960
C 4.513254 -3.365490 -2.252523
H 5.280159 -3.851871 -2.859813
C 3.272737 -1.388507 -1.641384
H 3.061202 -0.324951 -1.760913
C 4.253487 -2.006739 -2.414279
H 4.816017 -1.421264 -3.144931
C -3.981143 -0.893849 -2.119342
H -3.928805 -1.920121 -1.732949
H -4.602366 -0.899055 -3.030375
H -4.476747 -0.252608 -1.378056
C -1.033786 1.491927 -2.789150
H -0.833846 2.563200 -2.737252
C -0.011065 0.590889 -3.216958
C -0.297934 -0.792675 -3.269940
H 0.473993 -1.509501 -3.553264
C -1.565647 -1.261418 -2.838736
H -1.746129 -2.335823 -2.803099
C -2.612683 -0.384072 -2.441455
C -2.302854 1.002172 -2.405875
H -3.052871 1.705586 -2.040996
C 2.467295 -1.266313 4.734563
H 2.711917 -1.261113 5.799407
C 1.333016 1.117578 -3.610242
H 2.053009 0.302132 -3.753986
H 1.250808 1.669600 -4.560098

H 1.727568 1.811996 -2.855028
C -0.566696 -4.394111 0.968085
H -1.262705 -4.079171 1.756188
H -0.803202 -5.410595 0.626522
H 0.471633 -4.331595 1.317853
P -1.564912 1.041213 0.837318
C -2.630683 -0.075131 1.856184
C -2.775414 0.251626 3.214307
C -3.270548 -1.227466 1.382901
C -3.541774 -0.540586 4.066555
H -2.276076 1.145448 3.600845
C -4.021415 -2.035202 2.237829
H -3.154650 -1.514716 0.338409
C -4.163650 -1.692040 3.581557
H -4.754839 -2.320941 4.251978
C -2.828398 2.295322 0.373044
C -4.214286 2.074912 0.414793
C -2.377182 3.554629 -0.059354
C -5.109989 3.061750 0.004155
H -4.595615 1.115986 0.773537
C -3.270294 4.536413 -0.480630
H -1.305378 3.769391 -0.048283
C -4.644417 4.292780 -0.456291
H -5.347600 5.063664 -0.780065
C 1.880640 1.407131 -0.015144
C 3.166577 1.350360 0.576244
C 1.374328 2.679288 -0.365008
C 3.897846 2.503012 0.806759
H 3.597538 0.387140 0.854477
C 2.103229 3.831658 -0.139958
H 0.381809 2.721263 -0.811081
C 3.366393 3.740061 0.446464
H 4.888472 2.466933 1.260389
H 1.714149 4.815619 -0.403267
N 4.138187 4.947269 0.680997
O 5.229593 4.829272 1.201987
O 3.648359 6.006009 0.341405
H -2.891841 5.506161 -0.814503
H -6.184554 2.864230 0.044231
H -4.497155 -2.936321 1.841882
H -3.646266 -0.263537 5.118834

Table S90. Cartesian coordinates of **TS1**

Ru 0.018411 -1.815089 -0.458987
P 1.817129 0.727634 -0.246506
O 3.201059 -2.081623 1.583483
S 2.023996 -1.378391 2.124312
N 0.695082 0.981953 -1.358842
H 0.411442 0.053527 1.450645
C 1.134022 -0.528177 0.858380
C 2.288755 2.127290 0.828270
O 1.052011 -2.177697 2.884956
C 1.349880 2.598529 1.760807
H 0.356096 2.148182 1.830059
C 1.666401 3.662448 2.597565
H 0.923264 4.020241 3.313817
C 3.856750 3.807944 1.591393
H 4.839211 4.280795 1.523151
C 3.541805 2.744890 0.744594
H 4.276938 2.390995 0.018370
C 3.318779 0.290634 -1.162148
C 5.537060 -0.636393 -1.383337
H 6.365152 -1.210847 -0.961678
C 4.390474 -0.426496 -0.618577
H 4.315165 -0.860771 0.379392
C 5.628154 -0.122336 -2.676477
H 6.531385 -0.288351 -3.268761
C 3.409960 0.804876 -2.461749
H 2.555940 1.347948 -2.872915
C 4.564097 0.601553 -3.213958
H 4.629099 1.004954 -4.227157
C -0.832693 -4.499644 1.199076
H -0.309146 -4.126480 2.090021
H -0.750722 -5.598884 1.171322
H -1.896234 -4.233848 1.278336
C -0.434705 -2.937367 -2.288975
H -1.076096 -2.607146 -3.109235
C 0.969409 -2.638825 -2.346125
C 1.760552 -3.024748 -1.247260
H 2.818526 -2.760194 -1.219372
C 1.168243 -3.611934 -0.094304
H 1.791790 -3.789203 0.781350

C -0.227317 -3.900525 -0.029558
C -1.011520 -3.602853 -1.188945
H -2.088984 -3.774523 -1.163665
C 2.922810 4.266292 2.518338
H 3.172120 5.099568 3.179503
C 1.532821 -1.912483 -3.524637
H 1.000978 -0.961455 -3.678133
H 2.595583 -1.683735 -3.378385
H 1.428065 -2.519755 -4.438064
C 2.644850 -0.157235 3.268415
H 1.801296 0.416962 3.671423
H 3.135597 -0.738603 4.060315
H 3.361913 0.502763 2.764820
P -1.822903 -0.734288 0.207372
C -1.880454 0.382732 1.654714
C -2.363975 1.685461 1.461522
C -1.409959 0.009970 2.924829
C -2.379240 2.598293 2.515632
H -2.712852 1.989619 0.471274
C -1.419757 0.929085 3.974143
H -0.997422 -0.991581 3.076521
C -1.904890 2.222575 3.772421
H -2.753839 3.610616 2.346161
H -1.047069 0.629384 4.957148
H -1.913062 2.939332 4.597536
C -3.445394 -1.549607 0.235609
C -4.173711 -1.763487 1.415834
C -3.995258 -1.985459 -0.982028
C -5.403137 -2.417499 1.378720
H -3.771057 -1.415631 2.369975
C -5.221874 -2.642581 -1.015657
H -3.456721 -1.784619 -1.912717
C -5.929202 -2.864142 0.166622
H -5.956201 -2.579608 2.307274
H -5.637050 -2.969849 -1.972156
H -6.895074 -3.373923 0.141315
C -0.298692 1.894437 -1.516173
C -1.448609 1.503564 -2.254422
C -0.267933 3.228595 -1.031754
C -2.511480 2.364550 -2.454459
H -1.471619 0.490144 -2.654666

C -1.332668 4.091063 -1.222948
H 0.618026 3.589247 -0.511244
C -2.460756 3.653091 -1.917962
H -3.398468 2.059948 -3.010441
H -1.310386 5.114197 -0.846825
N -3.583446 4.551988 -2.100279
O -3.515949 5.655247 -1.592319
O -4.532627 4.152130 -2.746071

Table S91. Cartesian coordinates of **Int1**
Ru 0.388513 -1.984223 -0.031410
P 1.676057 0.993555 -0.499945
O 3.646966 -1.280773 1.596762
S 2.407697 -0.677537 2.120998
N 0.513536 0.993911 -1.582184
H 0.528071 0.329043 1.373603
C 1.301776 -0.217387 0.812095
C 1.940198 2.566938 0.397977
O 1.634256 -1.464943 3.092901
C 0.948937 2.999462 1.293315
H 0.042978 2.408613 1.452053
C 1.100187 4.196788 1.984349
H 0.318086 4.518982 2.675639
C 3.225861 4.558228 0.898891
H 4.118902 5.167750 0.742001
C 3.074941 3.361280 0.198303
H 3.848723 3.040124 -0.502722
C 3.195636 0.721063 -1.449742
C 5.524333 0.130622 -1.699471
H 6.446784 -0.267273 -1.270122
C 4.385556 0.231067 -0.900621
H 4.417098 -0.113999 0.134367
C 5.487940 0.528949 -3.034899
H 6.384407 0.448970 -3.654738
C 3.160721 1.122895 -2.791367
H 2.217994 1.491005 -3.202370
C 4.304482 1.028166 -3.579224
H 4.269487 1.341010 -4.625426
C 0.379254 -4.684552 1.829157
H 0.831920 -4.104971 2.645684
H 0.766794 -5.715904 1.869237

H -0.707288 -4.715716 1.986231
C 0.069793 -3.298968 -1.745207
H -0.692412 -3.201346 -2.521470
C 1.344124 -2.680047 -1.954847
C 2.291624 -2.794231 -0.912411
H 3.244722 -2.270624 -0.995833
C 1.987236 -3.460145 0.303185
H 2.710915 -3.424341 1.116894
C 0.711801 -4.052400 0.515058
C -0.229157 -4.012695 -0.562268
H -1.228414 -4.428517 -0.419135
C 2.242431 4.976142 1.793382
H 2.363385 5.913888 2.340822
C 1.624210 -1.929281 -3.218267
H 2.670169 -1.600775 -3.258787
H 1.423651 -2.561681 -4.096932
H 0.991075 -1.030108 -3.273709
C 2.902612 0.798940 2.994726
H 2.008924 1.310807 3.372421
H 3.523972 0.435102 3.823841
H 3.478146 1.458453 2.334058
P -1.514729 -1.137958 0.633343
C -1.787990 0.257002 1.768526
C -2.608561 1.326379 1.378600
C -1.151946 0.300277 3.020411
C -2.781654 2.424569 2.217215
H -3.103491 1.303117 0.405612
C -1.327417 1.404380 3.854600
H -0.498097 -0.523122 3.322882
C -2.139898 2.466881 3.456054
H -2.277161 3.328676 4.114069
C -3.177338 -1.784016 0.337890
C -4.215254 -1.645546 1.273419
C -3.443103 -2.446837 -0.871530
C -5.478675 -2.165005 1.005865
H -4.028903 -1.125616 2.215843
C -4.706671 -2.966414 -1.134895
H -2.649881 -2.529207 -1.617534
C -5.729034 -2.827514 -0.195821
H -6.723864 -3.227348 -0.404846
C -0.722012 1.550272 -1.658798

C -1.794237 0.760776 -2.154891

C -1.013160 2.901719 -1.335510

C -3.076869 1.264610 -2.271689

H -1.574465 -0.270260 -2.432927

C -2.295666 3.409110 -1.441282

H -0.203335 3.553871 -1.008681

C -3.328971 2.585352 -1.891604

H -3.903249 0.650658 -2.631001

H -2.522663 4.443477 -1.181671

N -4.680378 3.105834 -1.958187

O -4.877370 4.228702 -1.532556

O -5.543687 2.389184 -2.425453

H -0.828596 1.429245 4.826797

H -3.414741 3.253143 1.890511

H -6.276890 -2.049403 1.742962

H -4.900091 -3.469838 -2.085212

Table S92. Cartesian coordinates of **TS4**

Ru -1.417599 0.555405 -1.308989

P 1.344544 -0.867480 0.140526

O 0.262969 -2.459165 -2.692889

S -0.651685 -2.525549 -1.544921

N 1.738292 0.672053 -0.082244

H -0.916701 -1.484837 0.464463

C -0.370766 -1.165746 -0.434795

C 1.342267 -1.302496 1.917713

O -2.099806 -2.587691 -1.803847

C 0.229667 -0.912308 2.677400

H -0.626936 -0.436775 2.196084

C 0.207913 -1.105371 4.054771

H -0.676721 -0.808541 4.621841

C 2.431291 -2.029775 3.960532

H 3.304775 -2.455445 4.459646

C 2.453131 -1.842676 2.579613

H 3.348903 -2.123086 2.024521

C 2.524957 -1.983402 -0.698056

C 3.812401 -4.020021 -0.957047

H 4.090265 -5.012200 -0.593825

C 2.886668 -3.258068 -0.246090

H 2.452433 -3.663456 0.669894

C 4.384046 -3.515234 -2.123108

H 5.110086 -4.112974 -2.679022
C 3.114751 -1.480171 -1.863769
H 2.851575 -0.476139 -2.203329
C 4.034356 -2.243088 -2.574132
H 4.484826 -1.840468 -3.483903
C -2.000500 -0.862247 -4.375796
H -1.251307 -1.661286 -4.332954
H -2.105537 -0.526525 -5.421366
H -2.959847 -1.276649 -4.040192
C -2.190977 2.360356 -2.286144
H -2.964374 3.020935 -1.891396
C -0.827992 2.574818 -1.932059
C 0.152055 1.669139 -2.431682
H 1.189426 1.781115 -2.122568
C -0.232330 0.559175 -3.213708
H 0.510818 -0.188586 -3.493970
C -1.593048 0.297141 -3.526776
C -2.565393 1.212116 -3.023372
H -3.623664 1.005236 -3.193493
C 1.306906 -1.670675 4.699959
H 1.291378 -1.821441 5.781925
C -0.423667 3.725854 -1.063624
H 0.279593 4.383265 -1.597814
H -1.292119 4.328459 -0.767808
H 0.074890 3.356672 -0.156860
C -0.317970 -4.051778 -0.677074
H -0.878287 -4.052470 0.266948
H -0.691769 -4.838457 -1.345464
H 0.756862 -4.168130 -0.514443
P -2.807356 0.477462 0.387402
C -3.134198 -0.820412 1.614291
C -3.348608 -0.484009 2.962363
C -3.155238 -2.172477 1.236825
C -3.552919 -1.479020 3.912875
H -3.349976 0.566854 3.263959
C -3.348967 -3.164374 2.198270
H -3.028821 -2.435148 0.182042
C -3.542656 -2.823404 3.535536
H -3.717698 -1.204806 4.957946
H -3.366352 -4.213980 1.893323
H -3.697791 -3.603355 4.284686

C -4.100907 1.701099 0.739195
C -3.775987 3.065315 0.770266
C -5.434070 1.317277 0.956279
C -4.760960 4.025051 0.994306
H -2.733953 3.365320 0.639527
C -6.415946 2.278036 1.180072
H -5.699504 0.257077 0.945899
C -6.083931 3.633773 1.195756
H -4.491517 5.083713 1.024221
H -7.450939 1.966726 1.341245
H -6.856998 4.385068 1.373294
C 2.925552 1.258246 0.244239
C 3.030940 2.669072 0.101761
C 4.093243 0.582998 0.693884
C 4.195319 3.352941 0.389319
H 2.152230 3.214928 -0.239123
C 5.263185 1.261859 0.985908
H 4.082895 -0.500942 0.797155
C 5.314849 2.646393 0.835052
H 4.265418 4.435557 0.280833
H 6.154764 0.736989 1.330140
N 6.539350 3.357378 1.141351
O 7.491706 2.708732 1.530608
O 6.547740 4.564320 0.991852

Table S93. Cartesian coordinates of **Int3**
Ru 1.163213 -1.327914 -0.268083
P -1.093358 0.618108 0.816284
O -1.991549 -0.435057 -2.155889
S -0.807427 0.431700 -2.213587
N -2.297223 -0.374038 1.101794
H 0.662389 1.303082 -0.655666
C -0.009083 0.432491 -0.635781
C 0.103091 0.265770 2.135749
O 0.196404 0.167698 -3.252694
C 1.386892 -0.204952 1.830412
H 1.886175 -0.015670 0.815095
C 2.262360 -0.569621 2.858724
H 3.273638 -0.896038 2.612179
C 0.556991 -0.040724 4.487318
H 0.228682 0.015761 5.527731

C -0.314004 0.330117 3.465954
H -1.332524 0.652782 3.695283
C -1.516575 2.382901 1.013672
C -0.949367 4.729529 0.854808
H -0.238676 5.509591 0.571921
C -0.613808 3.393286 0.658218
H 0.361618 3.150643 0.230554
C -2.184936 5.066193 1.408964
H -2.449219 6.115877 1.557829
C -2.749500 2.723753 1.582602
H -3.451118 1.935607 1.864533
C -3.081943 4.063559 1.774940
H -4.049197 4.324158 2.210535
C 1.944808 -2.818714 -3.169627
H 1.453715 -2.009476 -3.727496
H 1.802031 -3.768900 -3.709540
H 3.018900 -2.594802 -3.121444
C 1.552561 -3.328651 0.614904
H 2.179287 -3.541857 1.484336
C 0.155259 -3.064751 0.821459
C -0.618911 -2.729295 -0.300700
H -1.662917 -2.439160 -0.180345
C -0.011445 -2.619219 -1.585836
H -0.619652 -2.269113 -2.419116
C 1.353016 -2.923186 -1.804258
C 2.134025 -3.298663 -0.666408
H 3.199400 -3.500011 -0.790848
C 1.845074 -0.486599 4.184237
H 2.530508 -0.766578 4.987458
C -0.443931 -3.098216 2.190219
H -0.819335 -4.110192 2.413150
H 0.298573 -2.834738 2.956672
H -1.280721 -2.387202 2.246736
C -1.378481 2.098336 -2.501133
H -0.512089 2.771256 -2.511236
H -1.862447 2.066656 -3.486384
H -2.095736 2.394113 -1.726022
P 3.031737 -0.199922 -1.348179
C 2.834951 1.634847 -1.201351
C 3.125190 2.371643 -0.039948
C 2.358588 2.331112 -2.324188

C 2.952663 3.754256 -0.004471
H 3.507952 1.857914 0.845532
C 2.174469 3.713750 -2.284864
H 2.116827 1.768685 -3.229084
C 2.475290 4.431861 -1.127511
H 2.345206 5.516631 -1.102366
C 4.531356 -0.393056 -0.281853
C 5.627496 0.463660 -0.501483
C 4.716581 -1.447818 0.623406
C 6.832973 0.284511 0.168604
H 5.531577 1.286409 -1.214680
C 5.927343 -1.636077 1.291649
H 3.890122 -2.130394 0.819366
C 6.992697 -0.767444 1.072580
H 7.940854 -0.908896 1.596239
H 3.189840 4.305506 0.909028
H 7.661281 0.971759 -0.022319
H 1.810139 4.236399 -3.173417
H 6.033051 -2.468023 1.993344
C -3.573096 -0.497467 0.638144
C -4.205939 0.367696 -0.288315
C -4.346016 -1.572616 1.145957
C -5.522310 0.182827 -0.667981
H -3.639595 1.187870 -0.726855
C -5.661191 -1.767875 0.766699
H -3.872655 -2.247186 1.862093
C -6.248802 -0.883777 -0.138725
H -6.008879 0.847801 -1.381708
H -6.255521 -2.593626 1.158468
N -7.630434 -1.077052 -0.536778
O -8.229432 -2.024100 -0.065481
O -8.113194 -0.279924 -1.317424

Table S94. Cartesian coordinates of **TS5**
Ru 1.094391 -1.338049 -0.136804
P -1.103381 0.724289 0.816042
O -1.972919 -0.503800 -2.161591
S -0.776068 0.345770 -2.217899
N -2.285585 -0.271029 1.181234
H 0.669091 1.268068 -0.668148
C -0.025559 0.417427 -0.618432

C 0.170650 0.504502 2.071779
O 0.262031 0.024036 -3.206757
C 1.244567 -0.345569 1.736716
H 2.110555 -0.236954 0.519165
C 2.175149 -0.633459 2.747744
H 3.042914 -1.255111 2.523289
C 0.950636 0.715995 4.345366
H 0.839638 1.122937 5.352769
C 0.017040 1.021193 3.358759
H -0.843398 1.656153 3.587275
C -1.564554 2.488698 0.870683
C -1.027625 4.825508 0.544247
H -0.324253 5.594179 0.215268
C -0.670451 3.483732 0.454611
H 0.315354 3.220562 0.064473
C -2.276858 5.183771 1.053536
H -2.558173 6.237523 1.119449
C -2.809785 2.852201 1.395769
H -3.501904 2.076524 1.730470
C -3.164281 4.197990 1.481960
H -4.140844 4.475625 1.885049
C 1.868669 -3.059041 -3.043797
H 1.130389 -2.839656 -3.824210
H 2.362807 -4.017146 -3.264791
H 2.632755 -2.260145 -3.068747
C 1.436536 -3.361788 0.758176
H 2.055464 -3.579463 1.631229
C 0.050845 -3.050960 0.946213
C -0.699196 -2.714565 -0.196774
H -1.732339 -2.382562 -0.093844
C -0.108373 -2.735860 -1.496279
H -0.722309 -2.435063 -2.344296
C 1.233550 -3.089461 -1.696344
C 2.014508 -3.361458 -0.526802
H 3.076371 -3.587503 -0.643417
C 2.026652 -0.114268 4.033586
H 2.770229 -0.355818 4.797300
C -0.566577 -3.015669 2.306284
H -1.084307 -3.967036 2.508313
H 0.191709 -2.861385 3.085901
H -1.299491 -2.195905 2.353518

C -1.322107 2.001543 -2.602896

H -0.451544 2.669204 -2.613251

H -1.768116 1.925158 -3.603540

H -2.067000 2.337326 -1.871360

P 2.989238 -0.286240 -1.242633

C 2.844611 1.556792 -1.205866

C 3.078654 2.331535 -0.057745

C 2.462315 2.204179 -2.389041

C 2.940867 3.717100 -0.095672

H 3.376616 1.844291 0.874549

C 2.317409 3.592354 -2.423593

H 2.255099 1.604144 -3.278128

C 2.559878 4.351780 -1.279886

H 2.454868 5.439162 -1.309993

C 4.536919 -0.469189 -0.247262

C 5.613074 0.403760 -0.484146

C 4.757262 -1.551472 0.615589

C 6.845263 0.209694 0.133653

H 5.484452 1.250395 -1.162825

C 5.990588 -1.748568 1.234508

H 3.942661 -2.247685 0.816794

C 7.042275 -0.866108 0.999464

H 8.009408 -1.015826 1.484612

H 7.661958 0.908080 -0.065871

H 6.126946 -2.597734 1.909261

H 3.125987 4.304615 0.806897

H 2.023977 4.083432 -3.355021

C -3.554396 -0.451877 0.720549

C -4.187664 0.317617 -0.287856

C -4.320589 -1.489416 1.312651

C -5.495424 0.079123 -0.666545

H -3.625138 1.100810 -0.793918

C -5.626974 -1.737725 0.934958

H -3.849030 -2.088848 2.093731

C -6.214684 -0.947300 -0.053768

H -5.980567 0.669517 -1.444001

H -6.215344 -2.533778 1.391856

N -7.586301 -1.197439 -0.451584

O -8.177923 -2.110035 0.092202

O -8.070337 -0.479722 -1.305516

Table S95. Cartesian coordinates of **Int4**

Ru 1.078519 -1.345515 0.017264
P -1.171832 0.733148 0.860818
O -1.918620 -0.581625 -2.124592
S -0.732785 0.284138 -2.152509
N -2.370505 -0.257294 1.188187
H 0.658214 1.244622 -0.571446
C -0.037586 0.394021 -0.529516
C 0.072787 0.556335 2.141820
O 0.343162 -0.038533 -3.101750
C 1.119858 -0.328598 1.825632
H 2.402289 -0.337329 0.272600
C 2.092064 -0.527584 2.819842
H 2.941696 -1.186828 2.628983
C 0.951842 0.977484 4.344882
H 0.892804 1.476315 5.314703
C -0.022912 1.200191 3.378389
H -0.860217 1.874760 3.578270
C -1.633603 2.499410 0.843451
C -1.072912 4.826362 0.483844
H -0.351810 5.585216 0.170692
C -0.716363 3.481998 0.447354
H 0.287122 3.205897 0.114470
C -2.344592 5.200657 0.921417
H -2.625093 6.256402 0.946093
C -2.900868 2.879846 1.298177
H -3.610477 2.114797 1.620488
C -3.254769 4.228350 1.331925
H -4.248536 4.518456 1.680546
C 1.891520 -3.208748 -2.848772
H 2.644161 -2.403274 -2.922312
H 1.157465 -3.050711 -3.647994
H 2.400574 -4.171570 -3.006443
C 1.427712 -3.332346 0.967932
H 2.037677 -3.523716 1.852760
C 0.043650 -3.004072 1.123457
C -0.688793 -2.697003 -0.044947
H -1.719108 -2.351415 0.039901
C -0.098263 -2.802994 -1.341755
H -0.708675 -2.548103 -2.207582
C 1.242770 -3.162363 -1.507655

C 2.014558 -3.360109 -0.314163
H 3.082036 -3.572011 -0.410151
C 2.008626 0.112214 4.056837
H 2.786430 -0.064322 4.804800
C -0.604776 -2.930724 2.467108
H -1.115522 -3.883041 2.682869
H 0.132172 -2.742101 3.259034
H -1.349869 -2.120808 2.470814
C -1.287409 1.925047 -2.587975
H -0.426510 2.605106 -2.581685
H -1.698001 1.823322 -3.601449
H -2.060817 2.264946 -1.888821
P 2.987659 -0.289894 -1.216503
C 2.865683 1.550993 -1.203970
C 3.111246 2.316662 -0.053609
C 2.485580 2.195432 -2.387361
C 2.977627 3.702179 -0.089401
H 3.401664 1.821759 0.877081
C 2.354465 3.585279 -2.420393
H 2.262421 1.595669 -3.272667
C 2.600282 4.339004 -1.274075
H 2.498676 5.426780 -1.301534
C 4.632380 -0.482226 -0.391391
C 5.713722 0.279992 -0.857428
C 4.873871 -1.452003 0.588137
C 6.994005 0.081627 -0.347435
H 5.550986 1.043587 -1.622874
C 6.155853 -1.651445 1.097981
H 4.042380 -2.048457 0.969037
C 7.221901 -0.884820 0.632388
H 8.226832 -1.037581 1.032283
H 3.164111 4.288226 0.813651
H 2.061588 4.080646 -3.349535
H 7.822278 0.689736 -0.719465
H 6.319486 -2.407287 1.870260
C -3.617054 -0.449664 0.676876
C -4.402771 -1.483990 1.249609
C -4.211769 0.302464 -0.367901
C -5.690229 -1.745301 0.820043
H -3.960890 -2.069644 2.058105
C -5.500711 0.051066 -0.798679

H -3.633203 1.083245 -0.859336
C -6.239950 -0.971727 -0.203602
H -6.293086 -2.538834 1.262221
H -5.955638 0.628212 -1.603864
N -7.591248 -1.235595 -0.656311
O -8.042546 -0.532433 -1.540090
O -8.200880 -2.144680 -0.126353

Table S96. Cartesian coordinates of **TS6**

Ru 1.039518 -1.378040 0.155190
P -1.261692 0.684367 0.911681
O -1.835518 -0.635306 -2.130757
S -0.677188 0.264514 -2.083424
N -2.477932 -0.309103 1.149228
H 0.623222 1.222141 -0.429864
C -0.065773 0.363275 -0.427118
C -0.085130 0.493181 2.255211
O 0.450459 -0.007475 -2.990270
C 0.978725 -0.385024 1.973924
H 2.634601 -0.046763 0.673508
C 1.907061 -0.587356 3.010864
H 2.765163 -1.248005 2.853494
C 0.693415 0.897081 4.498187
H 0.588289 1.385082 5.469677
C -0.238965 1.126293 3.491207
H -1.085495 1.797753 3.660891
C -1.711713 2.455605 0.891279
C -1.119582 4.783194 0.584910
H -0.378425 5.540558 0.318422
C -0.768838 3.437147 0.555230
H 0.250500 3.159191 0.274126
C -2.411189 5.161044 0.956444
H -2.686811 6.218186 0.975884
C -2.999414 2.840083 1.279837
H -3.729886 2.076001 1.554873
C -3.347439 4.190429 1.307613
H -4.357124 4.483181 1.604518
C 2.254942 -3.027191 -2.639005
H 3.293087 -2.723575 -2.441169
H 1.809295 -2.301237 -3.332683
H 2.276515 -4.017546 -3.121773

C 1.380925 -3.351127 1.100618
H 1.900850 -3.539590 2.041669
C -0.016567 -3.035476 1.118344
C -0.626890 -2.719774 -0.125988
H -1.665988 -2.387768 -0.143870
C 0.092604 -2.806124 -1.360268
H -0.424573 -2.545104 -2.283124
C 1.463040 -3.076807 -1.372709
C 2.104434 -3.312692 -0.106140
H 3.185475 -3.468290 -0.086895
C 1.766998 0.040054 4.249684
H 2.509807 -0.140688 5.031691
C -0.803005 -3.007379 2.387760
H -1.307955 -3.976165 2.533620
H -0.154755 -2.817833 3.254015
H -1.563861 -2.213958 2.332830
C -1.251521 1.898785 -2.521486
H -0.408095 2.598539 -2.469451
H -1.616587 1.802566 -3.552728
H -2.060775 2.212747 -1.851623
P 2.997792 -0.056093 -0.728892
C 2.901469 1.757907 -0.954780
C 3.147557 2.643264 0.105474
C 2.515330 2.266245 -2.202075
C 3.004878 4.016300 -0.080094
H 3.439624 2.250851 1.082706
C 2.378234 3.643057 -2.383736
H 2.277305 1.572926 -3.012071
C 2.620068 4.517325 -1.325075
H 2.507471 5.594745 -1.468923
C 4.763229 -0.417333 -0.426453
C 5.747349 0.172572 -1.228934
C 5.145588 -1.348348 0.547853
C 7.089171 -0.158064 -1.050828
H 5.463733 0.902350 -1.991982
C 6.487285 -1.680308 0.718089
H 4.385634 -1.804579 1.188162
C 7.464207 -1.086741 -0.081007
H 8.516680 -1.345353 0.054687
H 7.848258 0.314247 -1.679072
H 3.190000 4.698272 0.753143

H 6.772476 -2.401184 1.488245
H 2.075851 4.033367 -3.358644
C -3.690868 -0.494577 0.561664
C -4.230805 0.285368 -0.492495
C -4.501737 -1.549178 1.057584
C -5.490645 0.040181 -1.005019
H -3.632723 1.086961 -0.923384
C -5.760061 -1.804331 0.546524
H -4.102875 -2.157393 1.871613
C -6.255700 -1.003912 -0.484460
H -5.902677 0.639188 -1.817303
H -6.381619 -2.614068 0.929483
N -7.576271 -1.261261 -1.022198
O -8.209940 -2.188167 -0.554772
O -7.980434 -0.535257 -1.910588

Table S97. Cartesian coordinates of **3’**
Ru 1.089095 -1.145220 0.776295
O -1.328041 -1.245420 -2.090415
S -0.235509 -0.265556 -2.091073
P -1.233068 0.988943 0.574548
N -2.466529 0.107305 1.052146
C 0.103151 0.303147 -0.461372
H 0.786206 1.150139 -0.621755
P 3.056357 -0.069978 0.342566
C -1.715154 2.620836 -0.086555
O 1.046135 -0.654720 -2.710694
C -0.737013 3.521579 -0.528518
H 0.321572 3.255081 -0.485048
C -1.105827 4.771417 -1.015184
H -0.335578 5.467304 -1.356423
C -3.428937 4.246193 -0.608895
H -4.483694 4.529137 -0.636683
C -3.062570 2.994033 -0.114920
H -3.823847 2.300081 0.246940
C -0.176818 1.303971 1.996224
C 0.883295 0.386512 2.145243
C 1.678366 0.542590 3.294623
H 2.514442 -0.141447 3.472765
C 1.437297 1.559023 4.220401
H 2.079473 1.649563 5.101149

C 0.388369 2.461443 4.032011
H 0.206111 3.257893 4.756932
C -0.429538 2.326214 2.913996
H -1.265630 3.012405 2.751675
H 3.830924 0.134990 1.516579
C -1.007183 -1.968554 3.313220
H -0.413850 -1.501045 4.110573
H -1.548344 -2.828988 3.738550
H -1.738918 -1.236062 2.939918
C -0.137615 -2.421792 2.185431
C -0.656793 -2.561016 0.882730
H -1.694384 -2.293296 0.683883
C 0.167483 -2.998768 -0.198402
H -0.276855 -3.065169 -1.191609
C 1.524338 -3.304802 -0.013526
C 2.073412 -3.071653 1.289134
H 3.142847 -3.229176 1.450592
C 1.261752 -2.667399 2.370795
H 1.707070 -2.511465 3.354686
C 4.339380 -0.849930 -0.692126
C 4.046929 -1.234408 -2.006968
H 3.034078 -1.096465 -2.397250
C 5.038741 -1.814858 -2.795000
H 4.806794 -2.107672 -3.821766
C 6.313173 -2.038159 -2.274734
H 7.084544 -2.501967 -2.894234
C 6.601622 -1.673203 -0.960112
H 7.597623 -1.848878 -0.546816
C 5.620371 -1.074350 -0.172508
H 5.855790 -0.775465 0.852741
C 3.001950 1.670834 -0.238421
C 2.952675 1.981825 -1.603600
H 2.965280 1.184369 -2.348925
C 2.862785 3.310549 -2.015330
H 2.830526 3.542833 -3.082524
C 2.819641 4.338125 -1.072690
H 2.756096 5.379067 -1.398788
C 2.851918 4.031582 0.286899
H 2.804704 4.828538 1.032370
C 2.938877 2.704449 0.704994
H 2.944781 2.472803 1.772777

C -2.453725 5.132496 -1.062352
H -2.743749 6.112432 -1.449123
C 2.353018 -3.859796 -1.126137
H 3.420039 -3.644881 -0.985810
H 2.230175 -4.954705 -1.166647
H 2.037020 -3.444241 -2.092145
C -0.792289 1.143226 -3.039611
H -1.725640 1.540985 -2.622568
H -0.006862 1.909206 -3.033851
H -0.953246 0.756526 -4.054734
C -3.605164 -0.346195 0.464904
C -4.532498 -1.040990 1.287253
C -3.958289 -0.180485 -0.898871
C -5.729098 -1.521917 0.791274
H -4.276286 -1.175551 2.339820
C -5.155154 -0.656788 -1.400859
H -3.253943 0.298667 -1.576271
C -6.042048 -1.323630 -0.555228
H -6.441645 -2.050696 1.424819
H -5.421751 -0.532673 -2.450599
N -7.297233 -1.820714 -1.081323
O -7.543158 -1.616779 -2.254948
O -8.037924 -2.413367 -0.320039

Table S98. Cartesian coordinates of **TS2**
Ru -2.155088 0.902177 -0.375102
P 0.058604 -1.310359 -0.133754
O -3.162317 -2.703151 -0.210568
S -2.757184 -2.008581 1.023300
N 1.093109 -0.048770 0.119453
H -1.138726 -0.569891 1.718502
C -1.474110 -0.829225 0.699627
C 0.764252 -2.760452 0.704502
O -3.784294 -1.310495 1.803655
C 1.222854 -2.560483 2.014756
H 1.210854 -1.555286 2.447427
C 1.695178 -3.637312 2.756962
H 2.053149 -3.476551 3.776449
C 1.278179 -5.114615 0.891470
H 1.306237 -6.112706 0.448399
C 0.803146 -4.039666 0.140616

H 0.463939 -4.198305 -0.885302
C -0.108087 -1.719646 -1.889150
C -1.043179 -3.008577 -3.705714
H -1.772846 -3.736535 -4.068041
C -1.034426 -2.662862 -2.355979
H -1.769584 -3.089805 -1.669931
C -0.129736 -2.432889 -4.589646
H -0.135029 -2.717496 -5.644527
C 0.786660 -1.118175 -2.783439
H 1.485057 -0.359001 -2.429436
C 0.780117 -1.483615 -4.127498
H 1.486197 -1.015705 -4.817068
C -5.260273 1.562270 0.914754
H -5.238264 0.635626 1.504778
H -6.299274 1.753002 0.596620
H -4.947254 2.396098 1.559475
C -2.728891 2.385942 -1.890035
H -2.130022 3.240705 -2.211526
C -2.610753 1.143059 -2.580747
C -3.365560 0.051274 -2.084180
H -3.267918 -0.932776 -2.543781
C -4.216819 0.202452 -0.955673
H -4.722877 -0.679971 -0.564561
C -4.354359 1.431822 -0.268502
C -3.564364 2.519383 -0.749213
H -3.589101 3.473238 -0.218680
C 1.719012 -4.916951 2.198993
H 2.091637 -5.761266 2.783459
C -1.787388 1.020048 -3.822598
H -1.504618 -0.023310 -4.007357
H -2.359160 1.377619 -4.695557
H -0.868431 1.620080 -3.759972
C -2.130837 -3.266430 2.128601
H -1.720316 -2.786566 3.025305
H -3.017367 -3.861363 2.385690
H -1.378591 -3.884856 1.627180
P -0.386784 1.659965 0.572515
C -0.060785 1.441085 2.366654
C 1.205983 1.277594 2.945290
C -1.191455 1.427771 3.197270
C 1.335647 1.107906 4.321582

H 2.099084 1.262970 2.319899
C -1.059663 1.249442 4.573771
H -2.178494 1.528462 2.735202
C 0.204186 1.091923 5.139144
H 2.329551 0.979519 4.757467
H -1.951906 1.230814 5.203984
H 0.310078 0.953817 6.217995
C 0.583316 3.105111 0.083467
C 1.083944 4.051774 0.988116
C 0.816651 3.272606 -1.289695
C 1.808234 5.146366 0.520907
H 0.908546 3.927557 2.059134
C 1.548439 4.362018 -1.750312
H 0.429931 2.520905 -1.983470
C 2.046515 5.300607 -0.845066
H 2.193321 5.883436 1.229602
H 1.739768 4.477818 -2.819756
H 2.624838 6.154521 -1.205530
C 2.454114 -0.079395 -0.085184
C 3.211615 1.096771 0.153336
C 3.174730 -1.212019 -0.551487
C 4.580719 1.136370 -0.034764
H 2.697508 2.004077 0.462156
C 4.545555 -1.176449 -0.738326
H 2.651119 -2.140919 -0.771017
C 5.250256 -0.004389 -0.474867
H 5.149301 2.048416 0.147703
H 5.089739 -2.052865 -1.091070
N 6.687511 0.032219 -0.671089
O 7.230939 -0.980011 -1.067191
O 7.265093 1.073295 -0.427715

Table S99. Cartesian coordinates of **Int2**

Ru 2.128223 0.616506 0.109025
P 0.132982 -1.283106 -0.116020
O 2.983842 -2.918798 -0.558625
S 2.589245 -2.118745 -1.731435
N -0.936647 0.051609 -0.193837
H 1.016541 -0.482531 -2.197259
C 1.424768 -0.890390 -1.260323
C -0.980598 -2.564018 -0.868610

O 3.634775 -1.478739 -2.533622
C -1.473107 -2.311338 -2.158227
H -1.253132 -1.358806 -2.647608
C -2.249669 -3.252807 -2.822286
H -2.634859 -3.034234 -3.820999
C -2.058237 -4.736229 -0.931010
H -2.288634 -5.685854 -0.442395
C -1.288708 -3.784204 -0.259592
H -0.935598 -3.995247 0.751315
C 0.452622 -1.904746 1.548544
C 1.280962 -3.572698 3.090402
H 1.836334 -4.496928 3.266866
C 1.147155 -3.099127 1.785298
H 1.613179 -3.631964 0.956182
C 0.720173 -2.879898 4.160053
H 0.825505 -3.260622 5.178472
C -0.109037 -1.205000 2.628397
H -0.641563 -0.269283 2.451501
C 0.024980 -1.690530 3.922713
H -0.418048 -1.135033 4.752730
C 3.672296 3.468923 -1.036273
H 3.707798 3.201769 -2.101422
H 4.537149 4.116834 -0.814734
H 2.759847 4.058215 -0.867603
C 3.175520 1.117846 1.996931
H 2.781001 1.184593 3.013522
C 3.776287 -0.090979 1.557414
C 4.263326 -0.111633 0.226367
H 4.644432 -1.050780 -0.173625
C 4.253158 1.030630 -0.625178
H 4.628089 0.931039 -1.644145
C 3.692313 2.242685 -0.178798
C 3.135852 2.256290 1.142178
H 2.647547 3.167029 1.498319
C -2.539807 -4.474297 -2.210325
H -3.147796 -5.217458 -2.731342
C 3.901094 -1.287693 2.441679
H 3.827814 -2.207688 1.845965
H 4.876396 -1.290714 2.957605
H 3.110261 -1.303041 3.204413
C 1.755454 -3.251136 -2.835147

H 1.370977 -2.698311 -3.700983
H 2.534315 -3.958379 -3.150077
H 0.948229 -3.768491 -2.303424
P 0.119558 1.476210 -0.043912
C -0.254146 2.465509 -1.541532
C -0.525167 1.846945 -2.770151
C -0.037241 3.850856 -1.521748
C -0.589197 2.591622 -3.944109
H -0.699869 0.769174 -2.802625
C -0.099619 4.595586 -2.697741
H 0.179239 4.353548 -0.575141
C -0.375404 3.970113 -3.912775
H -0.425648 4.555093 -4.833810
C -0.695476 2.433259 1.293824
C -1.840401 3.217756 1.101173
C -0.168955 2.305388 2.584759
C -2.451282 3.848056 2.182965
H -2.255092 3.345757 0.100258
C -0.777898 2.938345 3.665991
H 0.723977 1.691257 2.720568
C -1.923215 3.708076 3.466085
H -2.404659 4.204504 4.312109
C -2.338640 -0.006032 -0.094867
C -3.134672 0.877516 -0.843992
C -2.972220 -0.918163 0.768177
C -4.516852 0.857731 -0.730328
H -2.661165 1.576136 -1.531993
C -4.353589 -0.956799 0.871097
H -2.376084 -1.602319 1.370646
C -5.115083 -0.063385 0.123219
H -5.146549 1.534899 -1.307416
H -4.856681 -1.661182 1.533242
N -6.570860 -0.094765 0.235645
O -7.058627 -0.923844 0.974094
O -7.201241 0.710628 -0.416355
H -0.809115 2.092857 -4.891110
H 0.066591 5.675079 -2.663191
H -3.347171 4.452263 2.021978
H -0.355255 2.831833 4.667973

Table S100. Cartesian coordinates of **TS3**

Ru -1.834410 0.727225 -0.340314

P -0.468772 -1.148346 -0.084843

O -3.962499 -2.220161 0.113777

S -3.371276 -1.686094 1.356445

N 1.119237 0.046116 -0.231116

H -1.453990 -0.684638 2.064248

C -1.868199 -0.874524 1.062214

C 0.484742 -2.263726 1.061252

O -4.229696 -0.833954 2.195238

C 1.109473 -1.757711 2.207388

H 1.146307 -0.680323 2.382195

C 1.700635 -2.617115 3.128912

H 2.187472 -2.202903 4.015212

C 1.067557 -4.507257 1.777751

H 1.056737 -5.584959 1.596440

C 0.465930 -3.647936 0.857059

H -0.007786 -4.063677 -0.035678

C -0.622296 -2.145688 -1.596001

C -1.808569 -3.678278 -3.041125

H -2.666353 -4.331781 -3.217972

C -1.716186 -2.989563 -1.832529

H -2.513877 -3.075352 -1.091079

C -0.821648 -3.538994 -4.017104

H -0.898511 -4.086723 -4.959274

C 0.358816 -1.989886 -2.585874

H 1.196403 -1.309580 -2.425462

C 0.259407 -2.689661 -3.786346

H 1.032860 -2.564326 -4.547815

C -4.320704 2.654442 1.111293

H -4.534647 1.856015 1.836775

H -5.269861 3.162442 0.871931

H -3.654503 3.391933 1.581767

C -2.123285 2.166499 -2.041233

H -1.341137 2.714166 -2.569097

C -2.509966 0.882820 -2.496610

C -3.511495 0.199199 -1.745360

H -3.812533 -0.812970 -2.016429

C -4.083090 0.789348 -0.595225

H -4.799842 0.207661 -0.014433

C -3.697020 2.071480 -0.117336

C -2.676517 2.725225 -0.851776

H -2.289664 3.683303 -0.495703
C 1.684943 -3.995925 2.916906
H 2.158187 -4.668940 3.635572
C -1.947889 0.296824 -3.751675
H -1.966798 -0.800057 -3.719483
H -2.543552 0.621347 -4.620778
H -0.910079 0.619870 -3.911598
C -2.932196 -3.099777 2.356567
H -2.471099 -2.748717 3.287869
H -3.873173 -3.626234 2.563708
H -2.235840 -3.735613 1.795446
P 0.224746 1.433410 0.247101
C 0.541998 1.809525 2.014838
C 1.808429 2.004190 2.587452
C -0.592065 1.853956 2.837808
C 1.930710 2.224563 3.957019
H 2.707900 1.971580 1.973030
C -0.466368 2.085743 4.206578
H -1.573150 1.685615 2.380102
C 0.795797 2.265999 4.768355
H 0.898513 2.439289 5.842342
C 0.876406 2.869462 -0.664543
C 0.902391 4.146407 -0.096089
C 1.263762 2.695904 -2.000140
C 1.312467 5.240732 -0.858609
H 0.607430 4.284133 0.947389
C 1.681173 3.788139 -2.753547
H 1.243025 1.693255 -2.436075
C 1.703330 5.063354 -2.184289
H 2.030887 5.920509 -2.777108
C 2.487211 -0.158792 -0.280477
C 3.420171 0.901059 -0.185688
C 3.003070 -1.459123 -0.493894
C 4.783756 0.674512 -0.269901
H 3.067714 1.924765 -0.071638
C 4.365228 -1.689383 -0.573067
H 2.322113 -2.302403 -0.584147
C 5.253393 -0.622663 -0.455679
H 5.500540 1.492840 -0.198038
H 4.759940 -2.693943 -0.725621
N 6.685412 -0.863414 -0.537410

O 7.056151 -2.005673 -0.714762
O 7.425322 0.092855 -0.423189
H 1.331647 6.236881 -0.410360
H 1.994630 3.645758 -3.790402
H 2.922124 2.365669 4.393964
H -1.359794 2.116012 4.834559

Table S101. Cartesian coordinates of **4'**
C 3.473758 -1.811059 0.027411
P -0.841663 0.758774 0.470069
O 3.468066 -0.041067 -2.768096
Ru 1.421039 1.257952 -0.069851
P 1.854315 -0.974625 0.128117
O 1.252937 0.304962 -3.921651
S 2.056657 -0.426250 -2.935549
C 1.197271 -0.383908 -1.415799
H 0.119161 -0.595705 -1.574237
C -2.024767 -0.740379 2.548509
H -2.681094 -1.144608 1.777104
C -2.170495 -1.160574 3.869313
C -1.320131 -0.677247 4.862139
H -1.438729 -1.009264 5.896449
C -0.306610 0.219895 4.525947
C -0.159530 0.637289 3.205241
H 0.655115 1.307855 2.928042
C -1.026425 0.178188 2.208030
C -2.061379 3.089026 1.606473
H -1.711446 2.745698 2.583287
C -2.764541 4.290821 1.503016
C -3.240223 4.720640 0.265717
H -3.795465 5.658310 0.187899
C -3.019315 3.941218 -0.871955
C -2.313810 2.745815 -0.773308
H -2.139301 2.127745 -1.658494
C 0.980060 -2.427384 2.308575
H 1.734009 -1.861308 2.861771
C 4.630669 -1.110795 -0.333737
H 4.563211 -0.055820 -0.583660
C 5.935527 -3.136634 -0.167956
H 6.895878 -3.652180 -0.242915
C 5.852073 -1.770375 -0.430816

H 6.743570 -1.211295 -0.723449
C 4.787748 -3.843486 0.185828
H 4.841819 -4.915685 0.387960
C 3.562521 -3.187926 0.283768
H 2.670727 -3.751713 0.562729
C 0.792202 -2.195572 0.939585
C 0.201302 -3.372298 2.967283
H 0.346291 -3.544952 4.035638
C -0.777944 -4.078470 2.266965
H -1.398223 -4.811995 2.787517
C -0.974680 -3.841268 0.908091
H -1.750607 -4.380097 0.359903
C -0.188132 -2.904308 0.240592
H -0.351007 -2.708700 -0.818727
C 1.661023 3.094002 -1.377324
C 0.954435 3.454799 -0.213236
H -0.033034 3.904101 -0.314466
C 1.449647 3.176098 1.094559
H 0.837896 3.442733 1.958097
C 2.698221 2.543979 1.276387
C 3.442929 2.224939 0.097304
H 4.411538 1.736922 0.208909
C 2.935492 2.467350 -1.190793
H 3.492998 2.112030 -2.058325
N -1.526860 -0.289952 -0.565436
C -1.820299 2.317068 0.466933
C 1.128465 3.374244 -2.745151
H 1.658245 4.233768 -3.186983
H 1.264327 2.501484 -3.400280
H 0.057811 3.615270 -2.703214
C 3.277180 2.280379 2.631430
H 3.792519 1.310120 2.659123
H 4.011879 3.061312 2.886894
H 2.503739 2.285092 3.410927
C 2.058894 -2.143178 -3.421253
H 1.021199 -2.489393 -3.505437
H 2.557447 -2.178220 -4.399143
H 2.621649 -2.721377 -2.676589
H 0.377622 0.588931 5.294474
H -2.952502 -1.881175 4.120216
H -2.949942 4.889058 2.398547

H -3.406759 4.264126 -1.841226

C -2.823020 -0.614547 -0.781290

C -3.097626 -1.586103 -1.787099

C -3.951655 -0.077347 -0.096464

C -4.380159 -2.006064 -2.072765

H -2.249620 -1.989364 -2.344901

C -5.239269 -0.492584 -0.383572

H -3.802132 0.679163 0.674901

C -5.456409 -1.460318 -1.365555

H -4.582281 -2.752400 -2.841616

H -6.099615 -0.081419 0.145498

N -6.802426 -1.900365 -1.654542

O -7.714942 -1.401386 -1.021776

O -6.952548 -2.748823 -2.514615

4.2.6 2b' + HPAr$^{Cl}{}_2$

Table S102. SCF energies, enthalpy and free energy corrections and barriers

	E_{SCF}/E_H	corrH/E_H	corrG/E_H	ΔH/kJ/mol	ΔG/kJ/mol
**TS-Act1[a]**	-	-	-	-	-
**Coord**	-4968.367802	0.70849	0.566044	-120.1525156	-45.70121208
**TS-Act2**	-4968.347755	0.70543	0.565868	-75.55159804	6.47164746
**Act'**	-4968.388162	0.710449	0.570227	-168.4646561	-88.17424064
**TS-Act3**	-4968.38385	0.70976	0.572563	-158.9512619	-70.71870892
**Act**	-4968.387899	0.710406	0.56681	-167.8873087	-96.45533019
**TS1[a]**	-	-	-	-	-
**Int1**	-4968.363953	0.710346	0.567712	-105.1738543	-31.2161448
**TS4**	-4968.345499	0.709543	0.568893	-58.82994607	20.33675543
**Int3**	-4968.358316	0.709225	0.567958	-93.31675499	-15.76998699
**TS5**	-4968.347773	0.704602	0.562447	-77.77290231	-2.557578314
**Int4**	-4968.351614	0.70652	0.563218	-82.82236893	-10.61849343
**TS6**	-4968.340713	0.705392	0.562553	-57.16283233	16.25664967
**3‘**	-4968.39389	0.708353	0.566348	-189.0057017	-113.3965527
**TS2**	-4968.350887	0.708911	0.56751	-74.6356136	2.559337399
**Int2**	-4968.369248	0.709621	0.566986	-120.9783404	-47.02325636
**TS3**	-4968.358601	0.708662	0.568322	-95.54273265	-15.56212615
**4‘**	-4968.401248	0.710226	0.569473	-203.4074094	-124.5111344

[a] Transition states not found

Table S103. Cartesian coordinates of **TS-Act1**

not found

Table S104. Cartesian coordinates of **Coord**

Ru -0.015768 -0.220797 -1.499181

N -1.679909 -0.241654 -0.158903

O 2.080388 -3.239315 -1.251484

P -1.421212 -1.768146 0.459574

S 1.075566 -3.275669 -0.164294

C 0.164433 -1.856190 -0.173450

C -1.540757 -1.852961 2.269260

O 0.182812 -4.454324 -0.077091

C -1.648666 -0.707880 3.065933

H -1.797397 0.269716 2.601888

C -1.411498 -2.067674 5.048773

H -1.363845 -2.151614 6.137039

C -1.297925 -3.210822 4.255965

H -1.156927 -4.189119 4.721413

C -1.365523 -3.109392 2.868768

H -1.264631 -4.000127 2.240338

C -2.672787 -2.957650 -0.119755

C -3.843187 -3.228081 0.598090

H -3.997605 -2.790484 1.587918

C -4.808752 -4.078602 0.064074

H -5.720683 -4.287229 0.628102

C -4.605880 -4.667733 -1.183297

H -5.362497 -5.337477 -1.599082

C -3.428887 -4.419722 -1.889900

H -3.254250 -4.906580 -2.852382

C -2.463121 -3.572082 -1.357086

H -1.514242 -3.410123 -1.865213

C 0.116412 -2.913785 -3.791809

H 0.631664 -3.453745 -2.983257

H 0.692383 -3.082570 -4.715507

H -0.891642 -3.319934 -3.941443

C 0.086444 -1.452640 -3.493174

C 1.303808 -0.772137 -3.162172

H 2.217889 -1.361375 -3.079163

C 1.327542 0.633035 -2.982182

H 2.277617 1.130034 -2.769705

C 0.135829 1.418845 -3.069728

C -1.070927 0.699942 -3.215335

H -2.018781 1.239488 -3.160972

C -1.098942 -0.707632 -3.473982

H -2.058498 -1.208441 -3.613401

C -1.588901 -0.819120 4.454215
H -1.686374 0.074856 5.074538
C 2.023781 -3.375897 1.353778
H 2.528557 -4.350817 1.326181
H 2.753965 -2.562392 1.401726
H 1.328382 -3.327085 2.201480
C 0.173054 2.911566 -2.988636
H 0.409617 3.334685 -3.978528
H -0.796649 3.324693 -2.678939
H 0.941342 3.259355 -2.285182
P 1.082783 0.541609 0.307197
H 0.396839 0.384556 1.532193
C 1.348496 2.365296 0.332706
C 0.284207 3.183959 0.720385
C 2.528861 2.932830 -0.154716
C 0.398421 4.565313 0.585659
H -0.645993 2.759342 1.102130
C 2.622324 4.319996 -0.267672
H 3.378282 2.310435 -0.443066
C 1.563475 5.152029 0.091263
H 1.643833 6.234086 -0.010762
C 2.755090 -0.060259 0.700658
C 3.284977 0.158406 1.976829
C 3.506895 -0.703366 -0.280700
C 4.571810 -0.291687 2.259247
H 2.706788 0.663576 2.752737
C 4.797049 -1.134566 0.025159
H 3.073088 -0.930410 -1.251218
C 5.342685 -0.938627 1.291840
H 6.348438 -1.287749 1.525296
Cl 5.233100 -0.048211 3.838002
Cl 5.727762 -1.941305 -1.183118
Cl 4.077508 5.024228 -0.882423
Cl -0.938474 5.570930 1.020441
C -2.705719 0.635629 0.024979
C -3.886019 0.319445 0.745004
C -2.595334 1.951844 -0.489538
C -4.878449 1.262248 0.944651
H -4.021543 -0.683551 1.145923
C -3.580794 2.898802 -0.284663
H -1.684409 2.211310 -1.027061

C -4.725431 2.550962 0.433261
H -5.786792 1.020376 1.497043
H -3.478724 3.918789 -0.656100
N -5.763098 3.540109 0.652876
O -5.595620 4.648609 0.183359
O -6.739052 3.204328 1.294313

Table S105. Cartesian coordinates of **TS-Act2**

Ru 0.560244 -0.965949 -1.214420
N -1.321281 -0.369794 -0.378041
O 2.385646 -3.412833 0.706258
P -1.503174 -1.754792 0.516754
S 0.958058 -3.493211 1.079986
C 0.191169 -2.047675 0.602011
C -2.300930 -1.562039 2.142996
O 0.239822 -4.721556 0.672031
C -1.588878 -0.885232 3.142687
H -0.570737 -0.543518 2.938049
C -3.462924 -1.113573 4.644551
H -3.919329 -0.935674 5.621079
C -4.171998 -1.799307 3.659054
H -5.182028 -2.161807 3.862907
C -3.596591 -2.021748 2.409823
H -4.157659 -2.550669 1.636129
C -2.601024 -2.872872 -0.405766
C -3.353100 -2.368490 -1.473243
H -3.288139 -1.309898 -1.733248
C -4.180730 -3.219335 -2.203818
H -4.769427 -2.822424 -3.034171
C -4.255875 -4.572275 -1.873707
H -4.901338 -5.239540 -2.449954
C -3.504030 -5.075189 -0.811369
H -3.550579 -6.137492 -0.561220
C -2.676547 -4.231310 -0.074926
H -2.046767 -4.637637 0.718873
C -0.229056 -4.051962 -2.581780
H -0.082353 -4.486186 -1.581420
H 0.218027 -4.737570 -3.320113
H -1.305880 -3.984021 -2.784312
C 0.431401 -2.715875 -2.681448
C 1.756095 -2.519517 -2.162736

H 2.241797 -3.321186 -1.603866
C 2.430231 -1.290134 -2.304281
H 3.432680 -1.179737 -1.887849
C 1.792581 -0.170282 -2.929526
C 0.463079 -0.335513 -3.369261
H -0.067412 0.509102 -3.811500
C -0.203898 -1.593490 -3.240545
H -1.247661 -1.676045 -3.550616
C -2.170081 -0.657049 4.386424
H -1.612066 -0.122223 5.158232
C 0.919868 -3.502143 2.866244
H 1.395238 -4.443790 3.171756
H 1.485891 -2.639282 3.237687
H -0.123136 -3.467331 3.206722
C 2.532717 1.116156 -3.116110
H 3.167154 1.051113 -4.015038
H 1.846347 1.963018 -3.252518
H 3.193609 1.321772 -2.261669
P 1.353504 0.437930 0.465707
H 0.758476 -0.707626 1.349627
C 0.657872 2.123462 0.541057
C -0.352554 2.402700 1.465273
C 1.004443 3.084569 -0.415991
C -1.020050 3.625446 1.405738
H -0.656880 1.664371 2.207008
C 0.326965 4.298724 -0.448892
H 1.772123 2.883201 -1.162067
C -0.695125 4.587835 0.452961
H -1.252330 5.522244 0.386155
C 3.157340 0.601595 0.663463
C 3.798309 1.834649 0.824946
C 3.903327 -0.587974 0.681234
C 5.184140 1.868654 0.977489
H 3.238706 2.770166 0.840474
C 5.284110 -0.515722 0.835139
H 3.412851 -1.565337 0.598615
C 5.946739 0.704631 0.978004
H 7.029757 0.743878 1.091924
C -2.139387 0.706905 -0.561099
C -1.765252 1.710967 -1.485165
C -3.343997 0.899819 0.161450

C -2.486103 2.882074 -1.607424
H -0.848559 1.562616 -2.053599
C -4.077604 2.064795 0.027267
H -3.681133 0.143089 0.868586
C -3.634619 3.065393 -0.836010
H -2.162660 3.682082 -2.274089
H -4.984735 2.233020 0.607734
N -4.341052 4.327431 -0.893635
O -5.411403 4.408128 -0.325067
O -3.812604 5.242245 -1.498775
Cl 5.975677 3.397504 1.163640
Cl 6.215014 -1.974840 0.836447
Cl -2.309637 3.939946 2.512429
Cl 0.712357 5.444216 -1.688320

Table S106. Cartesian coordinates of **Act'**
Ru -0.420216 -1.023973 -1.036425
P 1.912393 -1.375980 0.560827
O 1.255434 -4.396050 1.776157
S 0.463298 -4.016123 0.595720
N 1.350912 -0.084068 -0.280890
H -0.130637 -2.019285 1.522509
C 0.345992 -2.252648 0.555061
C 2.516841 -1.024505 2.232357
O 0.861122 -4.577074 -0.703002
C 1.623808 -0.433153 3.137119
H 0.589972 -0.232436 2.829651
C 2.057560 -0.093145 4.414244
H 1.360674 0.370100 5.116303
C 4.264978 -0.931064 3.896935
H 5.297581 -1.126331 4.194401
C 3.840544 -1.271455 2.614540
H 4.542175 -1.723339 1.910718
C 3.269373 -2.173988 -0.327402
C 4.820640 -3.988503 -0.667961
H 5.185743 -4.982085 -0.399168
C 3.748925 -3.441288 0.031380
H 3.276746 -4.002068 0.841184
C 5.422811 -3.277592 -1.706375
H 6.265967 -3.713150 -2.247855
C 3.870522 -1.461095 -1.371161

H 3.490870 -0.475887 -1.648741
C 4.951622 -2.012882 -2.054145
H 5.423523 -1.452532 -2.864291
C 1.443469 -2.854392 -3.348266
H 1.693834 -3.601868 -2.583309
H 2.303572 -2.189307 -3.495487
H 1.260563 -3.383607 -4.298042
C -2.103616 -2.043590 -2.074115
H -2.950525 -2.574437 -1.636102
C -2.244016 -0.670932 -2.413020
C -1.113975 0.010471 -2.923680
H -1.186954 1.075692 -3.149257
C 0.102401 -0.689535 -3.154823
H 0.972751 -0.131741 -3.509168
C 0.222449 -2.086349 -2.956802
C -0.887751 -2.724258 -2.337538
H -0.778317 -3.773303 -2.059096
C 3.376805 -0.341403 4.794885
H 3.714911 -0.072454 5.798383
C -3.581450 -0.010130 -2.325671
H -4.187235 -0.355481 -3.179070
H -4.117989 -0.279256 -1.408151
H -3.514403 1.082699 -2.395654
C -1.212768 -4.533781 0.911724
H -1.865797 -4.216263 0.090665
H -1.162622 -5.629492 0.964781
H -1.545276 -4.120191 1.871562
P -1.319529 0.143169 0.900325
C -3.130036 -0.096496 0.940549
C -3.579466 -1.427377 0.904655
C -4.078562 0.924082 1.091947
C -4.934443 -1.715533 0.959632
H -2.859085 -2.238262 0.802319
C -5.435051 0.605258 1.158359
H -3.775414 1.970400 1.144211
C -5.893261 -0.708961 1.082712
H -6.957179 -0.939584 1.119482
C -1.142406 1.952012 0.598187
C -0.261383 2.655870 1.427232
C -1.751574 2.626646 -0.465651
C 0.019742 3.996908 1.167753

H 0.234443 2.156288 2.260593
C -1.454856 3.962491 -0.706572
H -2.436500 2.103063 -1.128967
C -0.566033 4.671626 0.100017
H -0.305820 5.706931 -0.119778
Cl -6.593313 1.883918 1.320107
Cl -5.444066 -3.372501 0.838880
Cl 1.154224 4.837556 2.169055
Cl -2.152663 4.742723 -2.091094
C 1.906833 1.160503 -0.449240
C 1.351464 2.040117 -1.403307
C 3.010573 1.624845 0.306891
C 1.810606 3.335647 -1.543432
H 0.502194 1.696503 -1.986989
C 3.478402 2.919642 0.163176
H 3.479296 0.975503 1.045921
C 2.864990 3.778036 -0.746490
H 1.348205 4.028358 -2.246663
H 4.303511 3.294689 0.768613
N 3.289532 5.162201 -0.827522
O 4.282807 5.488833 -0.210754
O 2.617210 5.918131 -1.502784

Table S107. Cartesian coordinates of **TS-Act3**

Ru 0.037997 -0.517459 -1.346104
P 1.988179 -1.174542 0.656353
O 1.213817 -4.352969 1.084232
S 0.726193 -3.746859 -0.166008
N 1.628995 0.229922 -0.114690
H -0.201284 -2.013633 1.012419
C 0.474630 -2.016467 0.139497
C 2.142795 -1.032770 2.456100
O 1.512480 -3.962092 -1.389742
C 1.129524 -0.343136 3.137311
H 0.284500 0.074301 2.574763
C 1.207231 -0.190676 4.517807
H 0.417317 0.348505 5.045499
C 3.301807 -1.395001 4.544260
H 4.153123 -1.803307 5.093290
C 3.234606 -1.552335 3.162090
H 4.036556 -2.073356 2.636858

C 3.548853 -1.865943 0.044844
C 5.216449 -3.605913 -0.097604
H 5.542474 -4.621244 0.138237
C 3.979070 -3.163202 0.359618
H 3.337059 -3.832153 0.935812
C 6.031135 -2.766757 -0.857521
H 7.002315 -3.121401 -1.210830
C 4.364586 -1.027414 -0.723885
H 4.029204 -0.020638 -0.976119
C 5.605210 -1.477420 -1.168558
H 6.238328 -0.815690 -1.763752
C 2.567253 -1.567769 -3.494248
H 2.750210 -2.458896 -2.877851
H 3.333654 -0.815251 -3.271295
H 2.676772 -1.853462 -4.553394
C -1.234505 -1.427976 -2.937088
H -2.064647 -2.132225 -2.860151
C -1.503248 -0.035387 -2.932762
C -0.406121 0.862573 -3.045114
H -0.586884 1.937959 -3.006413
C 0.912129 0.357471 -3.169575
H 1.747537 1.061105 -3.191514
C 1.192698 -1.034485 -3.251255
C 0.093537 -1.907545 -3.072528
H 0.295540 -2.978289 -3.015964
C 2.290308 -0.716524 5.222210
H 2.349369 -0.592211 6.306132
C -2.910688 0.460165 -2.900306
H -3.327477 0.405708 -3.919215
H -3.540379 -0.156639 -2.244952
H -2.968906 1.501157 -2.563180
C -0.894390 -4.421340 -0.443553
H -1.324274 -4.019287 -1.366719
H -0.721772 -5.501811 -0.537874
H -1.535066 -4.209601 0.420083
P -1.308089 0.305154 0.498969
C -2.801371 -0.737149 0.756508
C -3.077490 -1.860981 -0.024469
C -3.645690 -0.453160 1.843904
C -4.170525 -2.673626 0.267676
H -2.432189 -2.085889 -0.869335

C -4.739816 -1.269840 2.107732
H -3.451813 0.408274 2.486285
C -5.021999 -2.396971 1.330451
H -5.878610 -3.032544 1.552453
C -2.108655 1.922911 0.144488
C -1.294183 3.062539 0.235861
C -3.467677 2.095808 -0.155231
C -1.816745 4.322937 -0.030762
H -0.247969 2.970486 0.526835
C -3.971391 3.371827 -0.396417
H -4.144938 1.243627 -0.210327
C -3.161317 4.504216 -0.354307
H -3.566053 5.496055 -0.552896
C 2.216351 1.458257 0.044929
C 1.786069 2.550103 -0.742781
C 3.266085 1.696270 0.968702
C 2.361367 3.803101 -0.623570
H 0.960023 2.393707 -1.433119
C 3.843934 2.947346 1.090356
H 3.630784 0.886817 1.602043
C 3.392738 3.997768 0.292471
H 2.012902 4.647843 -1.217451
H 4.649522 3.132578 1.801111
N 4.002560 5.310466 0.419839
O 4.892852 5.442075 1.235582
O 3.587267 6.197388 -0.298003
Cl -5.779358 -0.897346 3.441816
Cl -4.477576 -4.058293 -0.738475
Cl -5.650703 3.552506 -0.791841
Cl -0.775583 5.707201 0.053272

Table S108. Cartesian coordinates of **Act**
Ru 0.059384 0.025598 -1.420205
P 2.177688 -0.992662 0.191258
O 1.821057 -3.259993 -2.240773
S 0.699106 -3.273752 -1.296075
N 1.711418 0.537613 -0.140706
H 0.033810 -1.980199 0.503599
C 0.665526 -1.773862 -0.373158
C 2.433737 -1.298332 1.959402
O -0.645760 -3.533121 -1.825730

C 1.428512 -0.829988 2.819451
H 0.576249 -0.269532 2.413516
C 1.530996 -1.055611 4.187898
H 0.746257 -0.690741 4.854192
C 3.643648 -2.182708 3.853839
H 4.515222 -2.700416 4.260598
C 3.549898 -1.962652 2.480722
H 4.354287 -2.295484 1.821701
C 3.703534 -1.412807 -0.682053
C 5.440195 -2.941726 -1.376140
H 5.853903 -3.951977 -1.401416
C 4.244194 -2.703997 -0.708652
H 3.731713 -3.535991 -0.225266
C 6.100675 -1.897473 -2.024644
H 7.038272 -2.090033 -2.551211
C 4.366365 -0.368694 -1.334778
H 3.937845 0.634414 -1.313123
C 5.564224 -0.612301 -2.003582
H 6.078787 0.207892 -2.509055
C -2.709450 -1.221566 -3.023170
H -2.492775 -2.271828 -2.788010
H -3.173758 -1.182589 -4.022353
H -3.440104 -0.831729 -2.301521
C -0.295818 1.769227 -2.762077
H -0.349003 2.827764 -2.502339
C 0.949574 1.185080 -3.145088
C 0.983500 -0.189645 -3.474888
H 1.925599 -0.676365 -3.730744
C -0.198051 -0.967500 -3.368622
H -0.141810 -2.040173 -3.551332
C -1.455322 -0.406628 -3.015534
C -1.464292 0.977736 -2.694628
H -2.395067 1.437652 -2.360272
C 2.634521 -1.735345 4.705158
H 2.713462 -1.907939 5.781077
C 2.186284 2.024373 -3.203204
H 3.081251 1.403648 -3.335712
H 2.122381 2.715976 -4.058363
H 2.303499 2.630103 -2.293644
C 0.969895 -4.567214 -0.092410
H 0.082538 -4.618176 0.551309

H 1.073691 -5.494844 -0.670912
H 1.875603 -4.369687 0.495060
P -1.201216 0.477877 0.587244
C -2.173412 -0.981872 1.178224
C -2.653471 -0.907356 2.494788
C -2.404525 -2.144213 0.434724
C -3.356153 -1.976413 3.042138
H -2.474061 -0.012732 3.095277
C -3.086050 -3.212912 1.017298
H -2.031360 -2.239079 -0.584561
C -3.576919 -3.148110 2.318626
H -4.110165 -3.989226 2.760963
C -2.609679 1.567638 0.129966
C -3.904256 1.102611 -0.140795
C -2.347514 2.941004 0.007191
C -4.887476 1.993853 -0.565105
H -4.151102 0.045178 -0.036573
C -3.342565 3.806309 -0.434561
H -1.365603 3.340393 0.263750
C -4.628189 3.353330 -0.730504
H -5.403996 4.038444 -1.070417
C 2.134929 1.706664 0.433595
C 3.287695 1.792053 1.251305
C 1.413354 2.896352 0.190563
C 3.684485 2.999815 1.800015
H 3.880008 0.898784 1.456533
C 1.807200 4.105002 0.734080
H 0.528602 2.828593 -0.440164
C 2.945021 4.152059 1.539872
H 4.570425 3.073925 2.430956
H 1.246985 5.023010 0.552684
N 3.368783 5.419353 2.111272
O 4.359466 5.418821 2.814178
O 2.707942 6.404196 1.850862
Cl -2.975835 5.490943 -0.619201
Cl -6.473083 1.391522 -0.924285
Cl -3.318635 -4.670291 0.105819
Cl -3.946518 -1.868916 4.668295

Table S109. Cartesian coordinates of **TS1**
not found

Table S110. Cartesian coordinates of **Int1**

Ru 0.593965 -2.047910 0.444977
P 2.364883 0.382607 -0.855020
O 3.893248 -1.357309 1.997146
S 2.760306 -0.439409 2.215322
N 1.222098 0.205121 -1.943777
H 1.100241 0.549597 1.043981
C 1.777648 -0.265033 0.746337
C 2.882932 2.095782 -0.473212
O 1.834075 -0.758211 3.313534
C 1.965726 2.941930 0.169623
H 0.961151 2.588277 0.413379
C 2.319590 4.245415 0.498589
H 1.596257 4.887566 1.006116
C 4.504403 3.894719 -0.467068
H 5.499933 4.267137 -0.719560
C 4.151654 2.586582 -0.800576
H 4.871591 1.941706 -1.309497
C 3.826239 -0.417301 -1.565159
C 6.023830 -1.412261 -1.451929
H 6.859859 -1.785750 -0.856056
C 4.907992 -0.879353 -0.807240
H 4.866932 -0.864921 0.283491
C 6.073320 -1.472558 -2.843935
H 6.951609 -1.889713 -3.342690
C 3.877945 -0.475439 -2.963766
H 3.018176 -0.118062 -3.534990
C 4.999759 -1.000474 -3.599371
H 5.033422 -1.045223 -4.690432
C 0.153784 -3.966436 3.062083
H 0.683292 -3.220195 3.670685
H 0.385061 -4.971124 3.452354
H -0.926250 -3.795141 3.163165
C 0.070406 -3.785818 -0.777334
H -0.665338 -3.836300 -1.583198
C 1.424638 -3.455573 -1.105922
C 2.342921 -3.368297 -0.034436
H 3.366507 -3.047665 -0.231609
C 1.936438 -3.556375 1.311294
H 2.655484 -3.367588 2.107893

C 0.583127 -3.852618 1.634233
C -0.337530 -4.027118 0.552937
H -1.389122 -4.226680 0.768218
C 3.593774 4.722620 0.186457
H 3.874987 5.744669 0.450641
C 1.817461 -3.190712 -2.524909
H 2.906948 -3.108294 -2.624291
H 1.466358 -4.001307 -3.181326
H 1.379532 -2.239310 -2.865945
C 3.469600 1.143359 2.631391
H 2.667214 1.869262 2.812927
H 4.030565 0.954512 3.556520
H 4.138686 1.482648 1.831486
P -1.163741 -0.763970 0.707093
C -1.246038 0.958367 1.286834
C -1.956039 1.904536 0.535012
C -0.537148 1.356348 2.430887
C -1.944206 3.240634 0.925853
H -2.501552 1.610489 -0.362936
C -0.517683 2.706974 2.774160
H 0.023250 0.626705 3.022917
C -1.220171 3.662174 2.042489
H -1.203891 4.713134 2.331202
C -2.902401 -1.231593 0.521489
C -3.920074 -0.673265 1.307821
C -3.226664 -2.193293 -0.445480
C -5.236225 -1.086226 1.118541
H -3.696358 0.079332 2.065022
C -4.548785 -2.593393 -0.607189
H -2.451704 -2.609338 -1.088634
C -5.572065 -2.048770 0.166422
H -6.607093 -2.359098 0.026220
Cl 0.433093 3.223737 4.129566
Cl -2.813301 4.404579 -0.007460
Cl -6.488862 -0.396100 2.090575
Cl -4.931773 -3.781040 -1.807267
C 0.093014 0.891826 -2.262814
C -1.091902 0.160167 -2.540770
C 0.027387 2.302409 -2.403424
C -2.274258 0.791903 -2.883377
H -1.043095 -0.926637 -2.468290

C -1.151047 2.940379 -2.748643

H 0.932002 2.891938 -2.253946

C -2.304066 2.185793 -2.967823

H -3.189348 0.230500 -3.075209

H -1.205329 4.024281 -2.848673

N -3.551067 2.858686 -3.279995

O -3.532776 4.069321 -3.382803

O -4.546179 2.172812 -3.411409

Table S111. Cartesian coordinates of **TS4**

Ru -0.601919 0.679919 -1.696887

P 2.062250 -0.715452 -0.025503

O 1.585478 -1.873484 -3.225184

S 0.530961 -2.225302 -2.264295

N 2.306927 0.868535 0.051069

H -0.130973 -1.573559 -0.179149

C 0.497933 -1.060006 -0.920068

C 1.822677 -1.434748 1.639791

O -0.855243 -2.348388 -2.749079

C 0.590548 -1.187091 2.263841

H -0.192172 -0.643988 1.729832

C 0.359871 -1.604973 3.570681

H -0.609423 -1.418126 4.037688

C 2.605824 -2.473729 3.686560

H 3.406411 -2.963604 4.245385

C 2.837144 -2.059981 2.375673

H 3.821092 -2.227186 1.936011

C 3.472640 -1.569366 -0.812910

C 4.982760 -3.427230 -1.188028

H 5.295354 -4.448761 -0.959526

C 3.881957 -2.878739 -0.532438

H 3.347091 -3.480142 0.204949

C 5.683326 -2.673562 -2.127529

H 6.546314 -3.104561 -2.640254

C 4.191096 -0.816114 -1.749172

H 3.887384 0.213351 -1.950719

C 5.286153 -1.366478 -2.405604

H 5.836334 -0.769402 -3.136006

C -0.545105 -0.227756 -4.996480

H 0.274205 -0.954683 -4.956572

H -0.531915 0.273658 -5.978893

H -1.491683 -0.773491 -4.891982
C -1.406054 2.564857 -2.498940
H -2.297816 3.089588 -2.153139
C -0.149097 2.822846 -1.882128
C 0.987091 2.090025 -2.333357
H 1.941300 2.222375 -1.827505
C 0.857321 1.108878 -3.339921
H 1.711265 0.481184 -3.597808
C -0.399550 0.799642 -3.922657
C -1.532108 1.533766 -3.457962
H -2.521539 1.272666 -3.838120
C 1.366025 -2.257381 4.282590
H 1.185792 -2.585463 5.308879
C -0.009948 3.844050 -0.795759
H 0.698114 4.630046 -1.100572
H -0.968710 4.327964 -0.567574
H 0.378893 3.374224 0.118356
C 0.904707 -3.853087 -1.638049
H 0.195039 -4.113941 -0.842715
H 0.755543 -4.514134 -2.502117
H 1.943453 -3.899068 -1.301419
P -2.220030 0.186025 -0.288656
C -2.536369 -1.303514 0.698953
C -2.934285 -1.190835 2.040249
C -2.310236 -2.569642 0.140201
C -3.065112 -2.339070 2.812292
H -3.116882 -0.213449 2.490527
C -2.436742 -3.697562 0.948850
H -2.036020 -2.665537 -0.914770
C -2.810980 -3.605955 2.286817
H -2.901801 -4.497248 2.907055
C -3.638699 1.248273 0.100926
C -3.404688 2.610457 0.324042
C -4.950456 0.754555 0.141491
C -4.476715 3.468779 0.550507
H -2.384641 2.992827 0.341820
C -6.002599 1.632230 0.389121
H -5.157167 -0.304201 -0.023291
C -5.786877 2.997212 0.587475
H -6.621531 3.673074 0.772255
C 3.347625 1.486511 0.683477

C 3.336570 2.906052 0.749461
C 4.468406 0.836849 1.267506
C 4.352390 3.621888 1.351753
H 2.488929 3.432656 0.312564
C 5.488965 1.547246 1.875216
H 4.543475 -0.249049 1.229406
C 5.430987 2.938818 1.917677
H 4.334832 4.710917 1.401021
H 6.346073 1.042704 2.322035
N 6.500321 3.683365 2.553222
O 7.420973 3.053873 3.037421
O 6.416984 4.896245 2.565878
Cl -3.501499 -2.193554 4.482256
Cl -2.117944 -5.262714 0.274858
Cl -7.621507 1.025291 0.438332
Cl -4.168079 5.154871 0.795218

Table S112. Cartesian coordinates of **Int3**

Ru 0.438558 -1.466044 -0.693844
P -1.656355 0.234053 0.992060
O -2.728061 0.023732 -2.094649
S -1.503435 0.828269 -1.996512
N -2.907009 -0.730424 1.074979
H 0.069896 1.205400 -0.342720
C -0.637792 0.382011 -0.518066
C -0.430564 -0.510841 2.106429
O -0.562142 0.793278 -3.124083
C 0.813606 -0.945354 1.630295
H 1.281642 -0.525872 0.674504
C 1.692722 -1.620467 2.484383
H 2.670629 -1.936261 2.121045
C 0.076094 -1.451101 4.272491
H -0.215495 -1.654171 5.305467
C -0.800792 -0.779073 3.425112
H -1.790354 -0.476522 3.776326
C -1.932538 1.906565 1.664336
C -1.077837 4.091607 2.261016
H -0.245583 4.798115 2.298872
C -0.882291 2.832468 1.703392
H 0.103237 2.570248 1.313289
C -2.327043 4.435439 2.780020

H -2.483850 5.426017 3.213461

C -3.175913 2.244965 2.209348

H -3.989529 1.516759 2.202055

C -3.371618 3.512374 2.757133

H -4.346632 3.776778 3.172679

C 0.979975 -2.223284 -3.920914

H 0.532027 -1.267051 -4.224725

H 0.725192 -2.992832 -4.667597

H 2.070705 -2.099302 -3.898979

C 0.738998 -3.645798 -0.363522

H 1.391415 -4.105921 0.383191

C -0.627224 -3.371544 -0.012690

C -1.429226 -2.720549 -0.963839

H -2.445903 -2.419566 -0.710184

C -0.878202 -2.320195 -2.215889

H -1.501242 -1.741133 -2.896613

C 0.451641 -2.630699 -2.586566

C 1.259375 -3.322890 -1.629995

H 2.301581 -3.544435 -1.867487

C 1.321990 -1.870539 3.802549

H 2.012521 -2.393426 4.467960

C -1.165427 -3.726822 1.335105

H -1.601492 -4.738657 1.308917

H -0.373294 -3.718275 2.096719

H -1.947509 -3.010304 1.624557

C -1.999760 2.531854 -1.812008

H -1.109319 3.170690 -1.778919

H -2.593824 2.750781 -2.709363

H -2.607691 2.652294 -0.906584

P 2.309197 -0.203564 -1.596091

C 2.231656 1.553192 -1.020040

C 2.601456 1.969210 0.269126

C 1.727141 2.500793 -1.921619

C 2.460465 3.305390 0.634380

C 1.584002 3.827866 -1.521841

H 1.412409 2.191671 -2.919177

C 1.952646 4.256557 -0.249023

C 3.829551 -0.703442 -0.670494

C 4.973205 0.112869 -0.730147

C 3.937204 -1.936328 -0.018635

C 6.159201 -0.296378 -0.130359

H 4.945224 1.075517 -1.243572
C 5.134645 -2.323859 0.576627
H 3.075241 -2.595320 0.049922
C 6.266348 -1.518343 0.538051
H 7.198964 -1.823001 1.011412
C -4.203276 -0.714104 0.653600
C -4.838373 0.349887 -0.032558
C -4.992820 -1.855246 0.944612
C -6.173281 0.286403 -0.387551
H -4.258748 1.228881 -0.309810
C -6.326952 -1.928717 0.588011
H -4.517907 -2.682947 1.474936
C -6.915675 -0.852055 -0.075103
H -6.661949 1.104198 -0.917444
H -6.934683 -2.805187 0.813933
N -8.317113 -0.918242 -0.448026
O -8.929050 -1.931751 -0.173567
O -8.799781 0.044177 -1.011691
Cl 7.548393 0.734896 -0.207023
Cl 5.185807 -3.838231 1.427320
H 2.998443 1.259442 0.996899
H 1.839071 5.298618 0.048535
Cl 2.865715 3.794781 2.247912
Cl 0.909015 4.980043 -2.627940

Table S113. Cartesian coordinates of **TS5**
Ru 0.393658 -1.461669 -0.632039
P -1.717890 0.355424 0.884083
O -2.539187 0.164526 -2.397770
S -1.288323 0.897144 -2.144668
N -2.935383 -0.635045 1.024363
H 0.107199 1.203792 -0.337597
C -0.617976 0.412248 -0.580776
C -0.436715 -0.236243 2.008740
O -0.226504 0.803334 -3.156486
C 0.569116 -1.045623 1.442875
H 1.498254 -0.666746 0.257067
C 1.469126 -1.662956 2.326340
H 2.278673 -2.283193 1.939576
C 0.358876 -0.680053 4.243715
H 0.282170 -0.539403 5.323980

C -0.551226 -0.064620 3.389469
H -1.364677 0.545366 3.792026
C -2.088172 2.075067 1.352921
C -1.289576 4.283234 1.943513
H -0.456406 4.955385 2.161936
C -1.031624 2.955345 1.619220
H 0.000029 2.598183 1.593175
C -2.606847 4.741288 2.000023
H -2.811769 5.785003 2.249864
C -3.408628 2.529864 1.436664
H -4.237423 1.843864 1.250682
C -3.662466 3.864664 1.752331
H -4.694278 4.218578 1.810130
C 1.039302 -2.353733 -3.926599
H 0.329601 -1.832818 -4.580186
H 1.412770 -3.254781 -4.436480
H 1.893786 -1.676188 -3.751068
C 0.594762 -3.681873 -0.351036
H 1.197171 -4.184829 0.408738
C -0.764417 -3.342893 -0.054975
C -1.488604 -2.642640 -1.039911
H -2.495107 -2.285516 -0.822390
C -0.905513 -2.337311 -2.306668
H -1.498422 -1.767601 -3.021568
C 0.407847 -2.710464 -2.624493
C 1.168454 -3.355028 -1.595658
H 2.210618 -3.614017 -1.795337
C 1.363362 -1.483530 3.705563
H 2.083680 -1.974861 4.364413
C -1.380989 -3.661086 1.267703
H -1.951997 -4.600473 1.195123
H -0.616605 -3.778560 2.047538
H -2.068155 -2.852091 1.557968
C -1.718859 2.622578 -2.014124
H -0.816930 3.212663 -1.813017
H -2.133268 2.881188 -2.997690
H -2.470210 2.760415 -1.226872
P 2.353950 -0.273750 -1.435496
C 2.322171 1.491694 -0.881662
C 2.536823 1.877416 0.450058
C 1.999860 2.458418 -1.839531

C 2.413330 3.217513 0.803647
H 2.782617 1.140814 1.217714
C 1.875955 3.792685 -1.452480
H 1.797304 2.164224 -2.869901
C 2.083202 4.193806 -0.135833
H 1.977695 5.238862 0.154598
C 3.863142 -0.813389 -0.510474
C 4.995212 0.014526 -0.469723
C 3.956719 -2.098391 0.032112
C 6.169428 -0.441865 0.123041
H 4.972164 1.020532 -0.891513
C 5.138082 -2.529207 0.627046
H 3.097754 -2.765588 0.016162
C 6.264470 -1.714983 0.686349
H 7.186031 -2.055173 1.157342
C -4.251706 -0.725812 0.689216
C -4.862485 -0.075295 -0.411393
C -5.074457 -1.555145 1.494814
C -6.214967 -0.223388 -0.670497
H -4.240595 0.506442 -1.091040
C -6.424332 -1.706235 1.238137
H -4.611187 -2.062515 2.343123
C -6.993493 -1.032129 0.156357
H -6.687958 0.272898 -1.518385
H -7.060829 -2.334450 1.861779
N -8.411959 -1.181736 -0.115028
O -9.054607 -1.910984 0.614780
O -8.877449 -0.568392 -1.055268
Cl 1.417316 4.978957 -2.627982
Cl 2.611594 3.683983 2.461069
Cl 7.550933 0.599557 0.174266
Cl 5.183446 -4.110410 1.339362

Table S114. Cartesian coordinates of **Int4**
Ru 0.371275 -1.540491 -0.381928
P -1.769206 0.346818 1.030721
O -2.627310 -0.136340 -2.172725
S -1.401885 0.657733 -2.016571
N -2.999903 -0.644357 1.151452
H 0.047125 1.135917 -0.279064
C -0.674480 0.321645 -0.438625

C -0.494994 -0.182985 2.180396
O -0.359762 0.531129 -3.048922
C 0.507613 -0.998172 1.624247
H 1.718626 -0.672473 0.076606
C 1.490253 -1.474861 2.507692
H 2.305075 -2.102459 2.141055
C 0.449187 -0.351877 4.390771
H 0.433193 -0.105098 5.454490
C -0.536728 0.136195 3.540436
H -1.341247 0.768130 3.926845
C -2.133708 2.082897 1.455562
C -1.361380 4.349581 1.817858
H -0.546245 5.076949 1.826529
C -1.103478 3.032546 1.453862
H -0.085856 2.740023 1.187303
C -2.653663 4.728780 2.185976
H -2.859233 5.764018 2.468435
C -3.420515 2.460175 1.852364
H -4.219661 1.716944 1.883024
C -3.678505 3.784440 2.207593
H -4.687166 4.076214 2.508779
C 0.986207 -2.655029 -3.634018
H 2.075728 -2.525638 -3.571796
H 0.555905 -1.736163 -4.056141
H 0.777214 -3.494795 -4.315929
C 0.636875 -3.720507 0.052621
H 1.261172 -4.152301 0.837416
C -0.722692 -3.379938 0.339533
C -1.472757 -2.766024 -0.687656
H -2.481725 -2.410590 -0.478550
C -0.921633 -2.559552 -1.988505
H -1.538885 -2.061354 -2.735629
C 0.396970 -2.922585 -2.289027
C 1.183014 -3.467757 -1.222577
H 2.232062 -3.705725 -1.412978
C 1.460712 -1.157428 3.866119
H 2.245804 -1.542856 4.522125
C -1.324827 -3.607885 1.687056
H -1.878430 -4.560653 1.685625
H -0.554946 -3.652177 2.468654
H -2.026138 -2.792237 1.919373

C -1.885869 2.375891 -2.010196
H -0.994767 3.008003 -1.916217
H -2.366938 2.534884 -2.984573
H -2.592860 2.565797 -1.193067
P 2.291488 -0.320351 -1.402821
C 2.250024 1.471299 -0.950217
C 2.524900 1.924076 0.347745
C 1.858768 2.375441 -1.941245
C 2.395101 3.279308 0.637507
H 2.813001 1.228115 1.138366
C 1.735184 3.727377 -1.619226
H 1.598995 2.020534 -2.939435
C 2.004276 4.198222 -0.337497
H 1.896684 5.255835 -0.096947
C 3.916267 -0.760539 -0.628213
C 5.035655 0.047586 -0.863982
C 4.073643 -1.950466 0.085493
C 6.279735 -0.339028 -0.371292
H 4.949660 0.983120 -1.419784
C 5.326373 -2.312907 0.571536
H 3.215651 -2.590210 0.287833
C 6.448520 -1.519144 0.354055
H 7.426221 -1.805552 0.740213
C -4.268654 -0.702745 0.661919
C -5.073426 -1.796995 1.073706
C -4.864613 0.237242 -0.216159
C -6.382329 -1.939159 0.652322
H -4.629863 -2.527539 1.752926
C -6.174692 0.104132 -0.637740
H -4.269146 1.066700 -0.593995
C -6.932904 -0.982343 -0.201398
H -7.000828 -2.777932 0.972582
H -6.631127 0.825510 -1.315755
N -8.307030 -1.121289 -0.644391
O -8.758072 -0.260339 -1.374760
O -8.932068 -2.090363 -0.259340
Cl 7.654402 0.671361 -0.656849
Cl 5.475505 -3.774910 1.489992
Cl 2.660535 3.837454 2.253748
Cl 1.192760 4.841586 -2.827661

Table S115. Cartesian coordinates of **TS6**

Ru 0.333701 -1.638779 -0.157343
P -1.921002 0.248819 1.078621
O -2.514369 -0.296202 -2.188764
S -1.328888 0.530890 -1.936360
N -3.168715 -0.728588 1.079586
H -0.024259 1.046146 -0.102243
C -0.723614 0.219800 -0.303190
C -0.751252 -0.292560 2.331089
O -0.204159 0.433254 -2.882823
C 0.292855 -1.102906 1.846352
H 1.929378 -0.438463 0.699422
C 1.214177 -1.569424 2.801040
H 2.056908 -2.193761 2.488964
C 0.029481 -0.458608 4.603716
H -0.067555 -0.215576 5.664075
C -0.894623 0.026940 3.683737
H -1.726230 0.656804 4.012140
C -2.293026 1.992513 1.476963
C -1.531277 4.261685 1.855543
H -0.715219 4.987253 1.892413
C -1.261499 2.941353 1.511607
H -0.232929 2.647852 1.287611
C -2.837007 4.645524 2.167843
H -3.051743 5.683087 2.434545
C -3.594088 2.376443 1.816279
H -4.395815 1.635018 1.814983
C -3.863844 3.703498 2.152534
H -4.883762 3.999460 2.408410
C 1.506215 -2.627918 -3.264390
H 2.551416 -2.406044 -3.004074
H 1.082769 -1.751625 -3.773920
H 1.505001 -3.481634 -3.961045
C 0.601719 -3.792532 0.296331
H 1.109980 -4.218265 1.163456
C -0.783830 -3.441634 0.392457
C -1.376517 -2.822663 -0.741219
H -2.403543 -2.459971 -0.677156
C -0.653554 -2.637113 -1.961920
H -1.157118 -2.149289 -2.795889
C 0.707054 -2.945470 -2.042222

C 1.331219 -3.500480 -0.871357
H 2.403847 -3.705802 -0.896045
C 1.084632 -1.254409 4.154714
H 1.821017 -1.634124 4.868325
C -1.575253 -3.685798 1.634914
H -2.112604 -4.644243 1.549580
H -0.925794 -3.727290 2.519712
H -2.309542 -2.877045 1.769295
C -1.849346 2.238870 -1.981683
H -0.980090 2.888325 -1.823982
H -2.255844 2.383039 -2.991608
H -2.620050 2.422208 -1.223015
P 2.332088 -0.208755 -0.671584
C 2.271156 1.619697 -0.579089
C 2.477727 2.286906 0.636050
C 1.904905 2.332825 -1.724725
C 2.297098 3.666950 0.691643
H 2.746847 1.738061 1.540144
C 1.737426 3.714885 -1.636951
H 1.679252 1.809958 -2.655120
C 1.928655 4.396909 -0.438009
H 1.777178 5.474827 -0.379873
C 4.082616 -0.645532 -0.384255
C 5.114056 0.151166 -0.888889
C 4.382041 -1.840091 0.279069
C 6.436009 -0.257066 -0.714882
H 4.903983 1.088086 -1.407629
C 5.710179 -2.224235 0.433340
H 3.587240 -2.465517 0.689131
C 6.755705 -1.444426 -0.058862
H 7.793055 -1.752655 0.067613
Cl 6.069830 -3.700682 1.261011
Cl 7.712552 0.732775 -1.330074
Cl 2.475613 4.491089 2.201835
Cl 1.227553 4.597472 -3.033646
C -4.389573 -0.754448 0.478725
C -4.897233 0.225652 -0.410886
C -5.240244 -1.850316 0.778503
C -6.166883 0.128462 -0.949061
H -4.264598 1.063833 -0.698461
C -6.508842 -1.957259 0.240371

H -4.865497 -2.612567 1.464147
C -6.972653 -0.961913 -0.621347
H -6.554725 0.881516 -1.635265
H -7.162371 -2.798063 0.474044
N -8.304378 -1.063477 -1.185526
O -8.973194 -2.035297 -0.891257
O -8.679875 -0.170352 -1.920436

Table S116. Cartesian coordinates of **3'**
Ru 0.434321 -1.215197 1.110329
O -1.520899 -1.662052 -2.062250
S -0.548756 -0.564112 -1.963529
P -1.930789 0.805976 0.512181
N -3.189887 -0.022060 0.995817
C -0.469462 0.088562 -0.329313
H 0.179577 0.967203 -0.460856
P 2.398769 -0.101395 0.830756
C -2.387693 2.317020 -0.398668
O 0.836518 -0.841284 -2.393163
C -1.405447 3.269648 -0.698246
H -0.382008 3.131220 -0.342879
C -1.728073 4.399592 -1.441980
H -0.954356 5.134938 -1.674903
C -4.022638 3.652795 -1.575440
H -5.049798 3.804036 -1.915040
C -3.703454 2.520221 -0.825776
H -4.475651 1.788802 -0.579029
C -0.999624 1.300852 1.970983
C 0.044291 0.419294 2.318264
C 0.710994 0.694288 3.525120
H 1.521429 0.038941 3.860687
C 0.377584 1.799012 4.311132
H 0.924773 1.985127 5.239541
C -0.640424 2.670443 3.920352
H -0.889357 3.540584 4.531438
C -1.344661 2.408634 2.748688
H -2.162780 3.063198 2.435292
H 3.047597 0.201453 2.057951
C -1.917160 -1.918967 3.456380
H -1.430235 -1.372732 4.275452
H -2.476522 -2.766049 3.884706

H -2.624737 -1.243536 2.951599
C -0.912752 -2.421891 2.471116
C -1.271881 -2.677282 1.131792
H -2.287078 -2.463915 0.796085
C -0.317319 -3.169331 0.189195
H -0.640616 -3.325902 -0.839967
C 1.016794 -3.411726 0.551307
C 1.403041 -3.062974 1.886343
H 2.449219 -3.173532 2.182927
C 0.461334 -2.604848 2.832335
H 0.785123 -2.360768 3.845369
C 3.813977 -0.882827 -0.022406
C 3.644435 -1.401360 -1.309164
H 2.673023 -1.344417 -1.809177
C 4.726067 -2.020124 -1.931469
C 5.963256 -2.140069 -1.299893
H 6.797642 -2.635471 -1.796352
C 6.106755 -1.619609 -0.015284
C 5.045410 -0.989051 0.632048
H 5.192908 -0.586956 1.636074
C 2.347453 1.604226 0.149001
C 2.321402 1.821670 -1.233017
H 2.366297 0.990803 -1.939199
C 2.163812 3.121701 -1.707745
C 2.054033 4.209651 -0.843084
H 1.935118 5.221723 -1.229778
C 2.069482 3.967227 0.528921
C 2.211385 2.676279 1.035844
H 2.184912 2.519089 2.115129
C -3.037325 4.587681 -1.888815
H -3.291312 5.471733 -2.478440
C 1.985449 -4.023995 -0.408645
H 3.023683 -3.779433 -0.149790
H 1.883473 -5.121353 -0.383604
H 1.792768 -3.690255 -1.436909
C -1.124578 0.729445 -3.048351
H -2.144104 1.025470 -2.772172
H -0.436890 1.583070 -3.001048
H -1.108511 0.276823 -4.048882
C -4.290854 -0.575905 0.418943
C -5.439955 -0.752907 1.234154

C -4.374319 -1.012935 -0.926765
C -6.604828 -1.302929 0.733997
H -5.381313 -0.423995 2.273370
C -5.540365 -1.564950 -1.430580
H -3.487986 -0.960857 -1.559338
C -6.654281 -1.703047 -0.603391
H -7.492149 -1.430280 1.354669
H -5.605649 -1.905467 -2.464381
N -7.876654 -2.275863 -1.135053
O -7.888616 -2.602679 -2.305895
O -8.822225 -2.395712 -0.380163
Cl 1.870701 5.289409 1.622855
Cl 2.047594 3.391693 -3.413597
Cl 4.519672 -2.691608 -3.510553
Cl 7.630396 -1.762632 0.790619

Table S117. Cartesian coordinates of **TS2**
Ru 1.944696 -1.325114 -0.747499
P 0.692952 1.571950 -0.468111
O 4.160072 1.727981 -0.724715
S 3.618589 1.205464 0.540979
N -0.712854 0.780651 -0.136560
H 1.628664 0.446440 1.346160
C 1.983326 0.555955 0.306661
C 0.611726 3.186778 0.360748
O 4.382493 0.182539 1.260712
C 0.136999 3.186452 1.680242
H -0.221789 2.256647 2.129756
C 0.110848 4.368396 2.411756
H -0.267801 4.358918 3.436372
C 1.012300 5.564431 0.515822
H 1.348001 6.496930 0.056538
C 1.038975 4.382786 -0.224590
H 1.391062 4.395124 -1.258207
C 0.908491 1.864262 -2.242148
C 2.141350 2.697079 -4.145598
H 3.057966 3.118810 -4.564545
C 2.077921 2.418076 -2.782060
H 2.946959 2.585153 -2.142234
C 1.046528 2.441330 -4.971817
H 1.099716 2.672673 -6.038305

C -0.179215 1.580401 -3.077901
H -1.077692 1.118104 -2.667347
C -0.110962 1.878665 -4.436935
H -0.966171 1.660087 -5.080171
C 4.811714 -2.888899 0.334010
H 5.131503 -1.984837 0.869038
H 5.703883 -3.399552 -0.065908
H 4.328328 -3.567506 1.051242
C 1.885676 -2.990675 -2.184945
H 1.023760 -3.626874 -2.396985
C 2.089386 -1.807571 -2.953420
C 3.185519 -0.979691 -2.597262
H 3.353739 -0.040721 -3.125632
C 4.053686 -1.340678 -1.531822
H 4.842861 -0.644770 -1.246998
C 3.874237 -2.522544 -0.771939
C 2.743129 -3.324401 -1.103874
H 2.525184 -4.212021 -0.506925
C 0.554405 5.558687 1.832587
H 0.532367 6.487488 2.407135
C 1.233983 -1.501332 -4.141115
H 1.180053 -0.421722 -4.325050
H 1.659466 -1.977573 -5.040361
H 0.211958 -1.888557 -4.021340
C 3.540059 2.586789 1.672363
H 3.040642 2.268983 2.595708
H 4.593160 2.825211 1.872736
H 3.027849 3.438782 1.212967
P 0.077553 -1.374399 0.298114
C -0.050021 -1.023339 2.100627
C -1.043861 -0.242928 2.702242
C 0.992943 -1.542183 2.878026
C -0.967553 0.024722 4.066080
H -1.855911 0.192262 2.121449
C 1.035229 -1.271938 4.245318
H 1.786755 -2.119177 2.398257
C 0.062674 -0.484218 4.856650
H 0.111394 -0.261526 5.922557
C -1.395887 -2.338425 -0.111941
C -2.208893 -2.979750 0.829209
C -1.693799 -2.418708 -1.477033

C -3.317400 -3.697283 0.383483
H -1.993158 -2.919949 1.897141
C -2.805747 -3.141468 -1.892302
H -1.061846 -1.896216 -2.197487
C -3.632629 -3.787278 -0.973048
H -4.506907 -4.347071 -1.304710
C -1.977869 1.305805 -0.280222
C -3.095813 0.507527 0.073059
C -2.258276 2.605355 -0.780140
C -4.393288 0.972060 -0.044968
H -2.932315 -0.509153 0.423393
C -3.555133 3.074046 -0.898039
H -1.443930 3.262957 -1.080861
C -4.621720 2.259156 -0.526894
H -5.244598 0.348019 0.227637
H -3.761719 4.074727 -1.278300
N -5.982348 2.752434 -0.651716
O -6.137871 3.877395 -1.082913
O -6.884241 2.010850 -0.317772
Cl -2.150916 1.062609 4.789759
Cl 2.333872 -1.901185 5.197216
Cl -4.331970 -4.493688 1.535061
Cl -3.171486 -3.236568 -3.580872

Table S118. Cartesian coordinates of **Int2**
Ru 1.970969 -1.114827 -0.481430
P 1.068593 1.474202 0.340708
O 4.391267 1.528382 0.503994
S 3.759351 0.775333 1.600534
N -0.464876 0.720332 0.366096
H 1.651792 -0.057757 2.069372
C 2.125440 0.300026 1.141340
C 0.725765 2.913867 1.435046
O 4.440730 -0.404378 2.136269
C 0.269518 2.645281 2.734923
H 0.065811 1.616567 3.043494
C 0.064648 3.678521 3.640801
H -0.300388 3.455389 4.645860
C 0.773275 5.275153 1.976007
H 0.967977 6.306451 1.673008
C 0.969470 4.239205 1.061658

H 1.306916 4.469301 0.049365
C 1.512460 2.171791 -1.263168
C 2.906598 3.531656 -2.693741
H 3.812517 4.125626 -2.835175
C 2.662790 2.950520 -1.449490
H 3.384102 3.062893 -0.639951
C 2.017416 3.351887 -3.750383
H 2.219159 3.808296 -4.722074
C 0.613400 2.000493 -2.326816
H -0.282920 1.395950 -2.187801
C 0.868037 2.579481 -3.563308
H 0.166889 2.416568 -4.385091
C 2.147073 -4.499225 0.072387
H 2.388636 -4.463750 1.143638
H 2.606880 -5.407114 -0.352822
H 1.056925 -4.602890 -0.024209
C 2.525079 -1.679560 -2.552220
H 2.062418 -1.393909 -3.500084
C 3.635627 -0.942585 -2.063665
C 4.186451 -1.370409 -0.828497
H 4.979405 -0.773321 -0.378956
C 3.732958 -2.525073 -0.129437
H 4.193801 -2.777060 0.825898
C 2.647511 -3.274204 -0.626733
C 2.045501 -2.819408 -1.845371
H 1.174637 -3.353180 -2.234367
C 0.321896 4.998442 3.263588
H 0.163229 5.811514 3.975629
C 4.207564 0.224450 -2.799452
H 4.601782 0.963054 -2.088124
H 5.035086 -0.094271 -3.455906
H 3.445608 0.713311 -3.422323
C 3.623799 1.918590 2.967907
H 3.087252 1.435708 3.793831
H 4.661993 2.126788 3.259282
H 3.117294 2.836057 2.645823
P -0.171541 -0.960361 -0.159451
C -0.868353 -1.954227 1.216772
C -0.758704 -1.514368 2.541210
C -1.259079 -3.274398 0.960244
C -1.034516 -2.390114 3.585659

H -0.465314 -0.488573 2.764315
C -1.522414 -4.132580 2.024488
H -1.352835 -3.644305 -0.062835
C -1.415217 -3.710687 3.349205
H -1.623295 -4.391697 4.173699
C -1.435648 -1.156415 -1.483333
C -2.794800 -1.375379 -1.242394
C -0.981579 -0.953797 -2.788575
C -3.687936 -1.358361 -2.312161
H -3.171688 -1.556800 -0.236368
C -1.893229 -0.933980 -3.839287
H 0.083434 -0.789524 -2.960732
C -3.254696 -1.133002 -3.618680
H -3.965223 -1.112615 -4.445047
Cl -0.902543 -1.824719 5.217047
Cl -1.983034 -5.768059 1.695330
Cl -5.372784 -1.603309 -2.011267
Cl -1.322580 -0.637706 -5.448202
C -1.700178 1.391331 0.464060
C -2.768486 0.801139 1.160961
C -1.906076 2.637754 -0.154066
C -4.007380 1.422504 1.222227
H -2.621721 -0.150786 1.668060
C -3.134637 3.274737 -0.077064
H -1.098490 3.113899 -0.708486
C -4.178024 2.656805 0.605344
H -4.844906 0.973432 1.756028
H -3.306365 4.241467 -0.550029
N -5.477777 3.321478 0.679242
O -5.584320 4.402447 0.140699
O -6.365402 2.749973 1.274770

Table S119. Cartesian coordinates of **TS3**
Ru 1.625453 -1.089048 -0.703566
P 1.358910 1.135475 0.044331
O 4.822284 0.635916 -0.013335
S 4.166869 -0.011579 1.140481
N -0.559385 0.924646 -0.172656
H 2.024041 -0.217083 1.953030
C 2.440396 -0.017410 0.954423
C 1.063968 2.303906 1.477596

O 4.642062 -1.348183 1.522356
C 0.289837 1.884519 2.567349
H -0.237342 0.929170 2.530034
C 0.167962 2.676995 3.704571
H -0.447254 2.331016 4.538877
C 1.584080 4.339737 2.694749
H 2.086015 5.309879 2.730416
C 1.710008 3.542455 1.555484
H 2.302594 3.908589 0.714812
C 1.952062 2.230043 -1.279651
C 3.601580 3.621165 -2.370196
H 4.600962 4.062476 -2.380359
C 3.224773 2.812140 -1.298587
H 3.942139 2.589343 -0.509197
C 2.721309 3.859119 -3.423241
H 3.021782 4.497708 -4.257156
C 1.074820 2.455329 -2.350954
H 0.090116 1.984422 -2.348428
C 1.456445 3.269608 -3.412776
H 0.760562 3.444053 -4.236667
C 1.110914 -4.536757 -0.671985
H 1.498127 -4.738780 0.336336
H 1.299498 -5.429068 -1.291504
H 0.020850 -4.406413 -0.607197
C 1.687234 -1.430351 -2.897703
H 1.141770 -0.890700 -3.674249
C 3.011985 -1.041122 -2.544333
C 3.642494 -1.767502 -1.509708
H 4.617523 -1.431475 -1.158797
C 3.041258 -2.896991 -0.876397
H 3.574796 -3.386382 -0.060783
C 1.769431 -3.334753 -1.274935
C 1.096627 -2.557319 -2.272915
H 0.077169 -2.835357 -2.548041
C 0.815804 3.910507 3.773702
H 0.714660 4.537319 4.662580
C 3.722077 0.079142 -3.229404
H 4.422538 0.562390 -2.535979
H 4.295257 -0.311893 -4.086244
H 3.017628 0.832690 -3.606409
C 4.450229 1.053082 2.547376

H 3.986167 0.602314 3.433214
H 5.540172 1.106330 2.668383
H 4.023252 2.043717 2.344039
P -0.493721 -0.782452 -0.074773
C -0.999779 -1.341802 1.606523
C -2.164748 -0.955331 2.281263
C -0.076696 -2.184998 2.232659
C -2.382128 -1.416340 3.577878
H -2.897048 -0.285711 1.833525
C -0.327383 -2.646404 3.523439
H 0.844407 -2.450439 1.703762
C -1.476029 -2.266654 4.213023
H -1.662483 -2.620707 5.227006
C -1.790264 -1.449323 -1.172718
C -2.510312 -2.596871 -0.838107
C -1.942379 -0.854463 -2.429361
C -3.382146 -3.147479 -1.779208
H -2.402673 -3.066135 0.141327
C -2.823035 -1.420205 -3.344910
H -1.374535 0.039782 -2.691419
C -3.551199 -2.570869 -3.035654
H -4.236655 -3.007758 -3.761648
Cl -3.805369 -0.921040 4.425370
Cl 0.814874 -3.695981 4.285734
Cl -4.266985 -4.576584 -1.378496
Cl -3.015190 -0.694062 -4.901217
C -1.626337 1.807005 -0.101282
C -1.388221 3.198437 -0.138572
C -2.968944 1.370021 -0.063148
C -2.434058 4.103796 -0.091612
H -0.367978 3.570109 -0.201824
C -4.018600 2.273745 -0.024564
H -3.197060 0.305498 -0.093348
C -3.744985 3.637936 -0.027731
H -2.253145 5.178546 -0.108478
H -5.055997 1.940118 0.005974
N -4.846458 4.590272 0.021564
O -5.973620 4.143682 0.074524
O -4.569414 5.771324 0.006700

Table S120. Cartesian coordinates of **4‘**

C -4.033059 1.279529 -0.427820
P 0.659577 -0.357684 0.107290
O -4.025294 -0.888304 -2.589837
Ru -1.464569 -1.361889 -0.154481
P -2.288880 0.765786 -0.342824
O -1.976914 -1.505307 -3.927218
S -2.699940 -0.521993 -3.111442
C -1.649795 -0.023848 -1.804146
H -0.657710 0.308408 -2.160408
C 1.521214 1.783620 1.761532
H 2.128525 2.124063 0.923449
C 1.508440 2.519764 2.944830
C 0.724138 2.146180 4.035113
H 0.719321 2.738703 4.949779
C -0.073010 1.011514 3.909094
C -0.082789 0.262011 2.738286
H -0.750734 -0.593214 2.639112
C 0.725565 0.640336 1.664150
C 2.239095 -2.097778 1.744234
H 1.825363 -1.610563 2.628571
C 3.111368 -3.176332 1.896827
C 3.669015 -3.823040 0.796063
H 4.345469 -4.667334 0.927197
C 3.344391 -3.357985 -0.479454
C 2.482509 -2.281164 -0.661030
H 2.241031 -1.925343 -1.664879
C -1.711877 2.885783 1.324204
H -2.390332 2.386726 2.021797
C -5.062412 0.474108 0.060206
H -4.820763 -0.487348 0.508438
C -6.692411 2.121990 -0.608598
H -7.731985 2.449640 -0.681785
C -6.387243 0.892909 -0.027935
H -7.185287 0.253841 0.356325
C -5.667998 2.938746 -1.087572
H -5.901696 3.908338 -1.533270
C -4.342252 2.526271 -0.989730
H -3.540762 3.181668 -1.340164
C -1.450523 2.318951 0.069172
C -1.098436 4.078999 1.689098
H -1.294648 4.511538 2.672552

C -0.218968 4.710744 0.808015
H 0.277203 5.637091 1.104642
C 0.037098 4.151730 -0.442039
H 0.731120 4.636830 -1.131977
C -0.579983 2.959194 -0.816817
H -0.362298 2.512739 -1.786062
C -1.230654 -3.456818 -1.001190
C -0.599741 -3.397972 0.259815
H 0.455919 -3.658359 0.326337
C -1.267174 -2.953087 1.434534
H -0.717974 -2.913210 2.376903
C -2.619092 -2.544445 1.379276
C -3.285489 -2.651032 0.115928
H -4.328054 -2.351019 0.015022
C -2.604127 -3.068547 -1.041379
H -3.132006 -3.049605 -1.995024
N 1.140367 0.499394 -1.179591
C 1.913952 -1.664918 0.458288
C -0.522838 -3.924438 -2.231488
H -0.805688 -4.965981 -2.455221
H -0.807476 -3.295981 -3.087424
H 0.566976 -3.891903 -2.100280
C -3.347566 -2.101492 2.610304
H -4.123911 -1.361208 2.376271
H -3.840133 -2.965363 3.085421
H -2.666487 -1.656469 3.348767
C -2.922308 0.930788 -4.120458
H -1.940756 1.281078 -4.463175
H -3.536244 0.610779 -4.972932
H -3.446868 1.693394 -3.531254
C 2.350898 1.030458 -1.489502
C 2.446775 1.781689 -2.694341
C 3.543501 0.906152 -0.722594
C 3.624143 2.381762 -3.092918
H 1.549886 1.863839 -3.312126
C 4.727284 1.502681 -1.120160
H 3.531957 0.329535 0.203522
C 4.767129 2.244847 -2.300162
H 3.691132 2.959987 -4.014924
H 5.638033 1.409562 -0.527781
N 6.004039 2.877599 -2.708664

O 6.979501 2.732939 -1.996830
O 5.998690 3.520901 -3.741100
Cl 3.502136 -3.730595 3.489218
Cl 4.012420 -4.153663 -1.861868
Cl -1.106830 0.543486 5.222540
Cl 2.464049 3.957282 3.057619

4.2.7 2b' + HPAr$^F_2$

Table S121. SCF energies, enthalpy and free energy corrections and barriers

	E_{SCF}/E_H	corrH/E_H	corrG/E_H	ΔH/kJ/mol	ΔG/kJ/mol
**TS-Act1[a]**					
**Coord**	-4478.017178	0.777363	0.6108	-120.3993126	-30.72273458
**TS-Act2**	-4477.99128	0.774007	0.608739	-61.21324369	31.86335681
**Act'**	-4478.041216	0.779201	0.614948	-178.6844937	-82.94301066
**TS-Act3**	-4478.035975	0.778563	0.617416	-166.5989758	-62.70268984
**Act**	-4478.040759	0.779073	0.612069	-177.8210192	-89.30228672
**TS1[a]**					
**Int1**	-4478.0141	0.77897	0.611891	-108.097191	-19.77537102
**TS4**	-4477.995306	0.778292	0.611085	-60.53347549	27.45228051
**Int3**	-4478.009563	0.777985	0.610618	-98.77230769	-11.20663169
**TS5**	-4477.998664	0.773499	0.605191	-81.93413603	3.160944469
**Int4**	-4478.00175	0.775357	0.604649	-85.15782995	-6.36394945
**TS6**	-4477.989852	0.77413	0.605349	-57.14279977	26.71041923
**3'**	-4478.043173	0.777012	0.612362	-189.5706568	-94.87149732
**TS2**	-4478.00128	0.777764	0.610987	-77.60484406	11.50987694
**Int2**	-4478.020381	0.778328	0.609242	-126.273475	-43.22103351
**TS3**	-4478.007432	0.777438	0.611326	-94.61430334	-3.753624838
**4'**	-4478.050882	0.779016	0.612719	-204.5480316	-114.1730706

[a] Transition states not found

Table S122. Cartesian coordinates of **TS-Act1**
not found

Table S123. Cartesian coordinates of **Coord**
Ru -0.448480 -0.391698 -1.541930
N -2.050018 -0.651575 -0.149228
O 1.971906 -3.140812 -1.519197
P -1.606898 -2.164681 0.391264
S 1.002327 -3.375237 -0.423838
C -0.040042 -2.051624 -0.291069
C -1.639724 -2.336553 2.198419
O 0.235765 -4.640995 -0.435501

C -1.825305 -1.241116 3.048833
H -2.094768 -0.266689 2.635124
C -1.361677 -2.647010 4.958231
H -1.254919 -2.768505 6.038676
C -1.173633 -3.740976 4.112057
H -0.916529 -4.717980 4.527838
C -1.315227 -3.591686 2.734802
H -1.161470 -4.444271 2.065502
C -2.748017 -3.455644 -0.196301
C -3.837631 -3.897165 0.563153
H -3.982055 -3.536178 1.584795
C -4.733973 -4.819373 0.027576
H -5.582511 -5.161357 0.624409
C -4.542518 -5.308919 -1.263742
H -5.244465 -6.034896 -1.680938
C -3.444726 -4.888078 -2.014598
H -3.275925 -5.294851 -3.014517
C -2.547773 -3.968924 -1.480543
H -1.655975 -3.672257 -2.028705
C -0.174487 -2.951060 -3.974257
H 0.401756 -3.489850 -3.207731
H 0.391534 -3.024995 -4.916238
H -1.149244 -3.431294 -4.124999
C -0.316115 -1.514038 -3.599286
C 0.852100 -0.749496 -3.273767
H 1.814694 -1.262028 -3.248262
C 0.765660 0.642305 -3.029244
H 1.678543 1.209380 -2.829185
C -0.488854 1.328054 -3.033947
C -1.637480 0.518660 -3.175819
H -2.624573 0.973101 -3.069484
C -1.556910 -0.872467 -3.505137
H -2.476383 -1.444917 -3.639475
C -1.690231 -1.400477 4.427129
H -1.848127 -0.546492 5.089877
C 1.992408 -3.514923 1.063848
H 2.591329 -4.426361 0.935526
H 2.640831 -2.641489 1.179166
H 1.318745 -3.614569 1.924282
C -0.568252 2.814656 -2.889168
H -0.620866 3.283280 -3.885289

H -1.459895 3.131906 -2.330729
H 0.319084 3.216804 -2.383130
P 0.633369 0.378808 0.265377
H 0.000225 0.139460 1.505553
C 0.754736 2.217349 0.319076
C -0.379288 2.951283 0.680464
C 1.878656 2.893301 -0.157446
C -0.397929 4.334557 0.530551
H -1.270716 2.442020 1.055080
C 1.856513 4.282205 -0.295026
H 2.782034 2.340528 -0.428054
C 0.719995 5.010549 0.042210
H 0.704122 6.094939 -0.073310
C 2.355112 -0.108182 0.584484
C 2.915754 0.047498 1.854511
C 3.124071 -0.634310 -0.452821
C 4.234480 -0.341695 2.081465
H 2.327482 0.464027 2.675538
C 4.441214 -1.017017 -0.219507
H 2.677911 -0.796764 -1.430336
C 5.004197 -0.871124 1.046166
H 6.039402 -1.165272 1.227516
C -3.165509 0.088922 0.109632
C -4.262443 -0.395149 0.866846
C -3.243488 1.423871 -0.359072
C -5.354288 0.408006 1.143033
H -4.254289 -1.419087 1.236205
C -4.329198 2.232843 -0.077943
H -2.402699 1.810708 -0.933228
C -5.386467 1.720537 0.673177
H -6.198663 0.036347 1.724072
H -4.372275 3.268042 -0.416597
N -6.530019 2.563237 0.971103
O -6.526974 3.695247 0.530427
O -7.422723 2.087223 1.643911
C -1.675938 5.084634 0.797681
C 3.047973 4.968099 -0.906646
C 4.809919 -0.249517 3.469807
C 5.241107 -1.663963 -1.319954
F -2.464928 5.058182 -0.289781
F -2.367444 4.543343 1.798282

F -1.451375 6.363929 1.090289
F 3.054218 4.795920 -2.236437
F 4.193716 4.467079 -0.442752
F 3.046081 6.279014 -0.674055
F 4.570707 -1.367414 4.162786
F 4.273880 0.762620 4.155439
F 6.131261 -0.073386 3.443554
F 5.259607 -2.988130 -1.189297
F 4.743686 -1.376321 -2.524477
F 6.512068 -1.246151 -1.296840

Table S124. Cartesian coordinates of **TS-Act2**
Ru 0.458535 -1.029326 -1.387863
N -1.463431 -1.077477 -0.439751
O 2.874675 -3.326610 0.038572
P -1.189395 -2.409956 0.512925
S 1.664078 -3.404860 0.886953
C 0.522734 -2.258978 0.350767
C -1.788441 -2.260936 2.218088
O 1.044701 -4.729472 1.093144
C -2.425379 -1.113238 2.698486
H -2.690267 -0.305088 2.016549
C -2.438307 -2.053463 4.922076
H -2.692987 -1.972956 5.981471
C -1.803136 -3.201523 4.443951
H -1.559235 -4.017920 5.127734
C -1.479010 -3.312502 3.094302
H -0.969970 -4.205314 2.716064
C -1.996298 -3.919036 -0.096836
C -3.303697 -4.251817 0.278929
H -3.837516 -3.648083 1.017373
C -3.920470 -5.374853 -0.265958
H -4.941085 -5.630064 0.027786
C -3.230738 -6.177907 -1.174437
H -3.714583 -7.061996 -1.596406
C -1.916965 -5.867662 -1.524121
H -1.362654 -6.516359 -2.206592
C -1.299953 -4.743398 -0.983500
H -0.258681 -4.517451 -1.202633
C 1.620606 -4.061838 -2.818589
H 2.018074 -4.307264 -1.823496

H 2.420642 -4.270211 -3.546691

H 0.766976 -4.705774 -3.062529

C 1.268249 -2.613513 -2.898586

C 2.238981 -1.629998 -2.525265

H 3.185616 -1.954140 -2.092081

C 1.982941 -0.256098 -2.734185

H 2.751571 0.471782 -2.464192

C 0.747145 0.204466 -3.286768

C -0.259569 -0.768482 -3.468253

H -1.261399 -0.449240 -3.765084

C 0.005090 -2.166367 -3.319808

H -0.789196 -2.889170 -3.513851

C -2.752106 -1.012266 4.050275

H -3.253739 -0.113320 4.415169

C 2.206959 -2.880243 2.509000

H 2.925875 -3.638351 2.847774

H 2.693190 -1.899789 2.429215

H 1.339210 -2.836924 3.179721

C 0.543356 1.638105 -3.663706

H 0.667153 1.754091 -4.752564

H -0.461669 2.004765 -3.412344

H 1.286317 2.289665 -3.184228

P 0.932012 0.431686 0.337631

H 0.671978 -0.864047 1.184423

C -0.078519 1.937790 0.509095

C -1.056670 1.971356 1.504472

C -0.050452 2.960208 -0.444965

C -2.030023 2.970369 1.504787

H -1.070418 1.195758 2.272002

C -1.020697 3.957272 -0.437200

H 0.715065 2.963319 -1.221387

C -2.027527 3.960819 0.528200

H -2.802897 4.727846 0.515746

C 2.672946 0.927928 0.482598

C 3.088961 2.225097 0.800570

C 3.637218 -0.074832 0.307547

C 4.447533 2.514607 0.919194

H 2.356215 3.018197 0.963472

C 4.989602 0.224160 0.446483

H 3.333485 -1.102207 0.072899

C 5.407231 1.519055 0.745756

H 6.468289 1.748340 0.846999
C -2.597448 -0.359144 -0.693941
C -2.493163 0.844634 -1.430078
C -3.882546 -0.722813 -0.223390
C -3.576280 1.682378 -1.608796
H -1.511474 1.119659 -1.810955
C -4.974194 0.108161 -0.407898
H -4.018392 -1.663541 0.308039
C -4.815551 1.317580 -1.081639
H -3.480576 2.630526 -2.138281
H -5.960826 -0.157595 -0.027941
N -5.945711 2.215276 -1.225900
O -7.026146 1.836948 -0.820498
O -5.741869 3.299182 -1.737666
C 5.985272 -0.892463 0.269429
C 4.880018 3.932362 1.182950
C -1.038072 4.963283 -1.555344
C -3.122689 2.913145 2.538117
F 5.839452 -1.823918 1.212480
F 5.824236 -1.495312 -0.914050
F 7.243598 -0.451230 0.327207
F 6.054834 3.981283 1.812529
F 5.013531 4.615095 0.040148
F 3.987550 4.586836 1.929629
F -1.653252 4.454717 -2.638033
F -1.681441 6.077082 -1.221203
F 0.197885 5.294410 -1.936709
F -2.629577 2.645665 3.753099
F -3.805355 4.051301 2.613016
F -3.997093 1.935579 2.256939

Table S125. Cartesian coordinates of **Act’**

Ru -0.298831 -1.191650 -1.067270
P 1.903650 -2.039541 0.528694
O 0.631218 -4.888836 1.678434
S -0.052185 -4.333893 0.499735
N 1.616602 -0.642166 -0.283799
H -0.233558 -2.280452 1.472805
C 0.191654 -2.581601 0.500221
C 2.559988 -1.844667 2.205799
O 0.243225 -4.933792 -0.808630

C 1.801958 -1.100635 3.121024
H 0.826968 -0.697878 2.820953
C 2.296333 -0.866250 4.399782
H 1.705908 -0.282594 5.109561
C 4.294699 -2.116326 3.864996
H 5.269230 -2.514115 4.156339
C 3.809662 -2.351898 2.580575
H 4.407916 -2.924318 1.869044
C 3.075495 -3.078381 -0.372791
C 4.228470 -5.164288 -0.739444
H 4.383296 -6.215232 -0.485967
C 3.286424 -4.421987 -0.033397
H 2.706166 -4.887934 0.766261
C 4.967241 -4.574260 -1.765320
H 5.707380 -5.163270 -2.312166
C 3.813874 -2.486025 -1.403761
H 3.644846 -1.439991 -1.666124
C 4.763932 -3.235130 -2.093618
H 5.343770 -2.769664 -2.893685
C 1.180077 -3.308395 -3.414953
H 1.278256 -4.102639 -2.662566
H 2.153975 -2.823224 -3.555323
H 0.896076 -3.776409 -4.371909
C -2.145027 -1.836826 -2.137360
H -3.084722 -2.198894 -1.717568
C -2.007886 -0.457749 -2.448668
C -0.760628 -0.001805 -2.938096
H -0.618680 1.059853 -3.148085
C 0.294116 -0.924952 -3.178658
H 1.260171 -0.544490 -3.519317
C 0.133537 -2.321520 -3.009396
C -1.085504 -2.739272 -2.408209
H -1.188793 -3.794673 -2.152726
C 3.541806 -1.373004 4.772396
H 3.928383 -1.186013 5.776958
C -3.193730 0.448404 -2.365984
H -3.782312 0.313416 -3.288136
H -3.846851 0.203851 -1.520379
H -2.920655 1.509674 -2.313356
C -1.802683 -4.512261 0.786359
H -2.369420 -4.061450 -0.036507

H -1.970494 -5.597114 0.818529
H -2.064459 -4.060461 1.750819
P -0.956082 0.090237 0.890054
C -2.775180 0.219950 0.882157
C -3.472188 -0.997070 0.851217
C -3.531001 1.395960 0.976593
C -4.859641 -1.042317 0.849271
H -2.902166 -1.926065 0.796841
C -4.926197 1.346766 0.976669
H -3.033907 2.366269 1.036393
C -5.608171 0.133963 0.904914
H -6.697586 0.104946 0.898824
C -0.407253 1.824162 0.614974
C 0.592862 2.333493 1.452365
C -0.833336 2.617395 -0.451587
C 1.162884 3.582352 1.210448
H 0.954583 1.732826 2.288988
C -0.255432 3.858215 -0.702325
H -1.603319 2.242682 -1.124137
C 0.752510 4.351079 0.123238
H 1.233840 5.305439 -0.095797
C 2.428337 0.454226 -0.450937
C 2.077972 1.434000 -1.405123
C 3.608863 0.666453 0.301324
C 2.827421 2.582680 -1.571353
H 1.166726 1.293907 -1.979432
C 4.365256 1.814346 0.134381
H 3.918045 -0.061800 1.050706
C 3.966376 2.775065 -0.791789
H 2.534259 3.349996 -2.287557
H 5.259060 1.995415 0.731414
N 4.721675 4.007842 -0.923794
O 5.766623 4.099441 -0.313912
O 4.256378 4.877711 -1.633716
C 2.217992 4.116624 2.141458
C -0.642845 4.579113 -1.960602
C -5.696360 2.639765 0.971100
C -5.515568 -2.388545 0.740737
F -0.310160 5.864910 -1.940139
F -0.030301 4.024198 -3.027003
F -1.956653 4.493301 -2.195775

F 3.112017 4.867399 1.494411
F 1.678828 4.882478 3.096407
F 2.880646 3.132007 2.755354
F -5.127032 3.556250 1.756227
F -5.749042 3.158061 -0.262848
F -6.954590 2.473465 1.383540
F -6.840270 -2.324138 0.825708
F -5.082352 -3.227203 1.689455
F -5.217326 -2.972300 -0.438316

Table S126. Cartesian coordinates of **TS-Act3**
Ru 0.562981 -0.678636 -1.340334
P 2.462157 -1.370451 0.701420
O 1.659936 -4.540116 1.083917
S 1.205007 -3.924341 -0.173517
N 2.127603 0.044498 -0.062257
H 0.260225 -2.196890 0.999422
C 0.953848 -2.194694 0.140395
C 2.574526 -1.251156 2.505992
O 2.017995 -4.133385 -1.380245
C 1.546807 -0.567807 3.171731
H 0.715829 -0.141942 2.595587
C 1.592627 -0.430539 4.555202
H 0.791580 0.103985 5.070694
C 3.684618 -1.637892 4.616347
H 4.522723 -2.053239 5.180174
C 3.649222 -1.779708 3.231314
H 4.462953 -2.295312 2.719080
C 4.030417 -2.068098 0.117514
C 5.689809 -3.818211 0.002963
H 6.005223 -4.835821 0.243286
C 4.447146 -3.368468 0.438231
H 3.790631 -4.034652 1.001008
C 6.523260 -2.983056 -0.740829
H 7.498633 -3.343046 -1.076684
C 4.864986 -1.233831 -0.635549
H 4.541352 -0.224573 -0.892587
C 6.110715 -1.690913 -1.058051
H 6.758546 -1.032238 -1.640636
C 3.127799 -1.750633 -3.438721
H 3.289605 -2.643405 -2.818950

H 3.898985 -1.006718 -3.204161
H 3.251125 -2.038819 -4.495620
C -0.683401 -1.565272 -2.968915
H -1.524672 -2.258179 -2.916955
C -0.933631 -0.169262 -2.970336
C 0.176956 0.715177 -3.046817
H 0.009236 1.792865 -3.012966
C 1.491423 0.193875 -3.139384
H 2.335987 0.886720 -3.139347
C 1.755543 -1.200967 -3.221145
C 0.641188 -2.060718 -3.072894
H 0.828354 -3.134203 -3.018790
C 2.658555 -0.965792 5.278463
H 2.692709 -0.853635 6.364744
C -2.336584 0.340419 -2.988483
H -2.724090 0.262329 -4.017273
H -2.992549 -0.256241 -2.340345
H -2.399884 1.391351 -2.686405
C -0.411384 -4.587838 -0.492046
H -0.821451 -4.176173 -1.420215
H -0.243969 -5.668363 -0.594978
H -1.066594 -4.388493 0.362962
P -0.819253 0.143674 0.472526
C -2.370151 -0.827512 0.634950
C -2.646686 -1.958183 -0.135129
C -3.295564 -0.478360 1.633818
C -3.812568 -2.701061 0.055456
H -1.936932 -2.235825 -0.911552
C -4.466452 -1.207702 1.811915
H -3.099993 0.383802 2.276415
C -4.737943 -2.328403 1.024416
H -5.657379 -2.897171 1.168096
C -1.539715 1.795536 0.104475
C -0.712493 2.917370 0.279533
C -2.862232 2.024333 -0.294529
C -1.171671 4.201052 0.001573
H 0.305810 2.784472 0.648743
C -3.322500 3.313358 -0.560743
H -3.554883 1.188869 -0.404737
C -2.482791 4.413573 -0.426099
H -2.847226 5.420467 -0.630933

C 2.742736 1.258932 0.108850
C 2.366809 2.359926 -0.693588
C 3.773551 1.468546 1.059903
C 2.987466 3.591294 -0.571204
H 1.551324 2.229476 -1.402329
C 4.392682 2.699107 1.187815
H 4.092542 0.652195 1.708712
C 4.002581 3.756006 0.367680
H 2.685820 4.443776 -1.178883
H 5.184549 2.861946 1.919155
N 4.658615 5.046766 0.497706
O 5.523957 5.155803 1.342913
O 4.304455 5.936478 -0.247933
C -4.052981 -3.926500 -0.779255
C -5.479556 -0.750709 2.827722
C -4.726264 3.476173 -1.072841
C -0.230861 5.369957 0.114110
F -6.202178 -1.771816 3.295541
F -6.334328 0.127131 2.293990
F -4.897403 -0.152778 3.869319
F -5.348442 -4.162436 -0.967561
F -3.530308 -5.023229 -0.210113
F -3.479862 -3.817546 -1.990794
F -5.595533 2.747467 -0.369193
F -4.816415 3.061773 -2.347601
F -5.133431 4.744506 -1.040073
F -0.877453 6.503922 0.387013
F 0.687456 5.183012 1.061981
F 0.426529 5.562948 -1.041639

Table S127. Cartesian coordinates of **Act**
Ru 0.488564 0.387631 -1.454415
P 2.632844 -0.930713 -0.123831
O 2.175393 -2.681212 -2.964517
S 1.096955 -2.878511 -1.989644
N 2.152124 0.630875 -0.112255
H 0.495363 -1.954206 0.033479
C 1.105638 -1.580504 -0.801642
C 2.921243 -1.632649 1.521711
O -0.273937 -3.030832 -2.492406
C 1.911538 -1.414290 2.471496

H 1.059492 -0.770124 2.223117
C 2.005080 -1.998578 3.729749
H 1.205931 -1.845174 4.458038
C 4.133482 -2.974054 3.122346
H 5.008697 -3.573185 3.383372
C 4.042209 -2.401167 1.854587
H 4.848443 -2.546063 1.132463
C 4.135313 -1.139178 -1.105627
C 5.845133 -2.465895 -2.178518
H 6.249882 -3.442900 -2.450498
C 4.665306 -2.389292 -1.446913
H 4.156746 -3.310326 -1.162684
C 6.500196 -1.300182 -2.577052
H 7.424933 -1.365774 -3.154963
C 4.793193 0.027986 -1.508044
H 4.374139 0.999366 -1.241162
C 5.974951 -0.054469 -2.241313
H 6.485784 0.860301 -2.549889
C -2.268507 -0.547588 -3.308971
H -2.054108 -1.621272 -3.230108
H -2.680710 -0.357222 -4.313627
H -3.043810 -0.271830 -2.581271
C 0.098574 2.361380 -2.427219
H 0.021397 3.349701 -1.970546
C 1.353487 1.883002 -2.909196
C 1.411109 0.599432 -3.503607
H 2.362365 0.188229 -3.844044
C 0.243337 -0.202476 -3.561975
H 0.319936 -1.215450 -3.956152
C -1.024759 0.260563 -3.116194
C -1.055197 1.551758 -2.527649
H -1.997508 1.923870 -2.124408
C 3.113875 -2.782304 4.053427
H 3.186772 -3.239761 5.042822
C 2.575933 2.737725 -2.798772
H 3.481045 2.170698 -3.049491
H 2.497844 3.582088 -3.501994
H 2.684897 3.155614 -1.788248
C 1.423773 -4.373583 -1.067813
H 0.575173 -4.552568 -0.396625
H 1.487067 -5.174565 -1.816404

H 2.363997 -4.285749 -0.508740
P -0.728078 0.436587 0.630919
C -1.603295 -1.159537 0.968504
C -1.826187 -1.471871 2.313954
C -1.979609 -2.098056 -0.001132
C -2.400250 -2.687920 2.685395
H -1.529087 -0.752491 3.082738
C -2.552814 -3.313050 0.372214
H -1.789588 -1.892945 -1.054907
C -2.767495 -3.616319 1.716939
H -3.214284 -4.568139 2.005929
C -2.182214 1.517935 0.356432
C -3.467718 1.075713 0.022836
C -1.963293 2.902373 0.416499
C -4.473368 1.986074 -0.304516
H -3.685243 0.005719 -0.008013
C -2.960680 3.805412 0.065253
H -0.986880 3.276819 0.731845
C -4.229808 3.357273 -0.301813
H -5.014453 4.063460 -0.572439
C -2.614368 5.267706 0.025875
C -5.812345 1.444670 -0.725146
C -2.575743 -2.982389 4.149379
C -2.880914 -4.344274 -0.673865
F -3.134079 -4.174120 4.361532
F -3.336354 -2.063709 4.747790
F -1.392055 -2.976942 4.783907
F -3.905738 -5.115321 -0.296131
F -1.839355 -5.166222 -0.882618
F -3.197657 -3.792754 -1.841711
F -5.717724 0.797087 -1.896782
F -6.287733 0.568443 0.162495
F -6.722204 2.407114 -0.876369
F -1.933636 5.644658 1.110161
F -3.692516 6.042942 -0.073497
F -1.830375 5.537580 -1.033566
C 2.572597 1.656537 0.693156
C 3.722677 1.569630 1.513701
C 1.849971 2.870104 0.701764
C 4.115038 2.635498 2.305875
H 4.315536 0.653409 1.528942

C 2.239102 3.937950 1.489320
H 0.969379 2.937645 0.065030
C 3.373511 3.815175 2.291572
H 4.998561 2.576551 2.941796
H 1.677621 4.872911 1.502803
N 3.792288 4.934246 3.119996
O 4.781454 4.786840 3.809119
O 3.128691 5.949629 3.072172

Table S128. Cartesian coordinates of **TS1**
not found

Table S129. Cartesian coordinates of **Int1**
Ru -0.922216 -2.022311 -0.995300
P -2.707841 -0.220510 1.078478
O -4.305470 -1.201174 -2.133062
S -3.305081 -0.120407 -2.068511
N -1.506738 -0.687927 2.002194
H -1.615991 0.598222 -0.763008
C -2.216636 -0.320159 -0.679320
C -3.263361 1.510332 1.285731
O -2.446681 0.080176 -3.246181
C -2.384503 2.540779 0.917548
H -1.394840 2.308934 0.517489
C -2.749945 3.872379 1.079321
H -2.047758 4.660872 0.800577
C -4.879227 3.175309 1.978721
H -5.858314 3.422477 2.395598
C -4.510705 1.838249 1.828419
H -5.200407 1.046373 2.129735
C -4.114231 -1.247266 1.576529
C -6.271325 -2.285041 1.253961
H -7.112616 -2.525913 0.600018
C -5.203804 -1.542029 0.750035
H -5.207799 -1.237090 -0.297846
C -6.264845 -2.722067 2.577480
H -7.105030 -3.302574 2.966289
C -4.109010 -1.686858 2.907121
H -3.242466 -1.459075 3.531964
C -5.182653 -2.419597 3.404558
H -5.171452 -2.761108 4.442206

C -0.406659 -3.081616 -4.054036
H -1.003264 -2.242180 -4.437110
H -0.565625 -3.953404 -4.709687
H 0.654764 -2.803712 -4.098975
C -0.268176 -4.002749 -0.325996
H 0.484102 -4.241770 0.429663
C -1.634137 -3.869801 0.081820
C -2.574645 -3.532973 -0.919697
H -3.611455 -3.343011 -0.640055
C -2.182221 -3.300541 -2.261826
H -2.924945 -2.934676 -2.970056
C -0.818680 -3.406582 -2.653730
C 0.129740 -3.826641 -1.669404
H 1.187048 -3.893575 -1.933184
C -4.003206 4.192151 1.603597
H -4.294147 5.237999 1.725463
C -2.019441 -4.064712 1.513447
H -3.108954 -4.031785 1.637474
H -1.654608 -5.036172 1.880629
H -1.589160 -3.262668 2.133392
C -4.221429 1.394151 -1.860949
H -3.526513 2.237003 -1.773634
H -4.820240 1.482998 -2.777313
H -4.868258 1.323396 -0.978231
P 0.736597 -0.590152 -0.909320
C 0.678135 1.224043 -0.986537
C 1.307606 1.997483 0.000793
C -0.049095 1.861956 -2.001795
C 1.208204 3.385503 -0.033158
H 1.867225 1.513573 0.804649
C -0.151282 3.253164 -2.013787
H -0.561021 1.266813 -2.764772
C 0.482213 4.020769 -1.040493
H 0.419154 5.109787 -1.069756
C 2.504845 -0.947490 -0.800958
C 3.501025 -0.101661 -1.308814
C 2.895722 -2.114847 -0.135304
C 4.845854 -0.423828 -1.147377
H 3.229357 0.820780 -1.825643
C 4.240689 -2.426107 0.029749
H 2.132113 -2.768720 0.286836

C 5.228478 -1.582797 -0.472309
H 6.283618 -1.818313 -0.329890
C -0.357872 -0.127700 2.464158
C 0.831614 -0.901585 2.426517
C -0.268454 1.161509 3.049480
C 2.038630 -0.414737 2.895288
H 0.761205 -1.907281 2.011788
C 0.933587 1.651782 3.532601
H -1.169800 1.768116 3.139860
C 2.086576 0.872212 3.434445
H 2.952768 -1.007797 2.848997
H 1.005473 2.643001 3.979786
N 3.354146 1.401548 3.907620
O 3.347552 2.487473 4.450395
O 4.348879 0.727038 3.726493
C 1.821020 4.222061 1.059385
C -1.000355 3.931720 -3.054471
C 5.897619 0.469006 -1.752659
C 4.596922 -3.645123 0.834904
F -2.272387 4.045660 -2.627102
F -1.029159 3.255775 -4.197318
F -0.571545 5.168066 -3.313155
F 0.903772 4.525847 1.990095
F 2.285853 5.378570 0.581131
F 2.821512 3.594668 1.667065
F 6.153467 0.118331 -3.018162
F 5.506223 1.744376 -1.768562
F 7.047473 0.399371 -1.082413
F 5.855616 -4.029225 0.637496
F 4.442130 -3.420377 2.144332
F 3.799680 -4.677193 0.523365

Table S130. Cartesian coordinates of **TS4**
Ru 0.055680 0.900034 -1.738405
P 2.662443 -0.576513 -0.081532
O 2.356463 -1.430624 -3.423217
S 1.297621 -1.917758 -2.529520
N 2.805202 0.996996 0.199462
H 0.514367 -1.483537 -0.435358
C 1.160885 -0.881925 -1.089687
C 2.383454 -1.518301 1.461519

O -0.069860 -2.061129 -3.060259
C 1.111119 -1.406490 2.043443
H 0.332906 -0.822733 1.546469
C 0.833297 -2.008762 3.266021
H -0.165451 -1.924593 3.697950
C 3.109383 -2.794935 3.391163
H 3.902735 -3.324078 3.923834
C 3.387706 -2.196554 2.163114
H 4.399924 -2.261602 1.762205
C 4.148691 -1.246773 -0.904270
C 5.779497 -2.956325 -1.441776
H 6.144427 -3.979765 -1.328578
C 4.625335 -2.556623 -0.770693
H 4.100548 -3.275333 -0.139050
C 6.466458 -2.052957 -2.250275
H 7.371060 -2.368013 -2.775405
C 4.854165 -0.342678 -1.707523
H 4.498506 0.686494 -1.790554
C 6.002431 -0.744881 -2.380492
H 6.541995 -0.030927 -3.006437
C 0.260191 0.288520 -5.093755
H 1.106997 -0.406854 -5.096497
H 0.276300 0.878044 -6.025827
H -0.665881 -0.300115 -5.065181
C -0.777058 2.830484 -2.407758
H -1.693175 3.309519 -2.059650
C 0.445217 3.064647 -1.718007
C 1.622262 2.407037 -2.182044
H 2.550941 2.525414 -1.626759
C 1.561873 1.515461 -3.273783
H 2.444074 0.937798 -3.552449
C 0.336082 1.220059 -3.929173
C -0.835754 1.881293 -3.452915
H -1.800802 1.629567 -3.896799
C 1.831774 -2.712999 3.938799
H 1.614872 -3.186048 4.899267
C 0.500227 3.981598 -0.535547
H 1.238286 4.780191 -0.705104
H -0.471388 4.456867 -0.348940
H 0.806117 3.423863 0.360562
C 1.744815 -3.572174 -2.033918

H 1.126547 -3.891459 -1.186781
H 1.522273 -4.189476 -2.914103
H 2.811473 -3.609993 -1.798524
P -1.606019 0.225371 -0.454665
C -1.983308 -1.368316 0.329064
C -2.541631 -1.404370 1.615302
C -1.702694 -2.577820 -0.324664
C -2.797847 -2.621421 2.239909
H -2.781147 -0.472601 2.133723
C -1.942144 -3.789081 0.323234
H -1.310328 -2.562399 -1.347558
C -2.491989 -3.822192 1.602803
H -2.699186 -4.774215 2.092631
C -3.104232 1.195104 -0.129143
C -2.964906 2.543679 0.210020
C -4.397215 0.664920 -0.262781
C -4.085492 3.358025 0.368563
H -1.964632 2.953739 0.361006
C -5.510737 1.478201 -0.083146
H -4.534906 -0.389310 -0.512165
C -5.364688 2.833074 0.225447
H -6.240843 3.468102 0.361696
C 3.786712 1.579851 0.951562
C 3.709215 2.980287 1.176475
C 4.906179 0.911291 1.515791
C 4.663949 3.661571 1.905846
H 2.860432 3.519871 0.758977
C 5.865837 1.586481 2.249981
H 5.030143 -0.159996 1.362422
C 5.744759 2.960735 2.444512
H 4.595932 4.736348 2.076020
H 6.722695 1.067621 2.680559
N 6.751238 3.669200 3.211655
O 7.675612 3.024408 3.667257
O 6.614100 4.868469 3.354965
C -1.571527 -5.081680 -0.353015
C -3.365950 -2.623083 3.634158
C -6.893701 0.915646 -0.284738
C -3.868131 4.807569 0.706997
F -0.304974 -5.425981 -0.054564
F -1.646837 -4.992605 -1.678455

F -2.348878 -6.089954 0.040391
F -3.898198 -3.801290 3.953571
F -4.308246 -1.690918 3.777805
F -2.414561 -2.358218 4.544135
F -6.921747 -0.403496 -0.091237
F -7.771857 1.472025 0.551912
F -7.330658 1.146217 -1.526845
F -3.284034 4.950338 1.897822
F -3.054696 5.384804 -0.194310
F -5.004725 5.498613 0.722441

Table S131. Cartesian coordinates of **Int3**
Ru -0.124117 -1.557061 -0.881835
P -2.141478 -0.035282 1.053177
O -3.265736 0.137160 -2.051939
S -2.038905 0.917870 -1.850132
N -3.394340 -1.000377 1.059245
H -0.428051 1.053692 -0.201635
C -1.159648 0.272928 -0.456732
C -0.889507 -0.902885 2.041600
O -1.096921 1.038233 -2.971801
C 0.331424 -1.294713 1.476237
H 0.768489 -0.779374 0.554537
C 1.232020 -2.065524 2.218207
H 2.193363 -2.348349 1.791918
C -0.316627 -2.077834 4.072398
H -0.573409 -2.389209 5.087458
C -1.214830 -1.308833 3.336518
H -2.186170 -1.036532 3.756561
C -2.402774 1.555612 1.904640
C -1.576724 3.701615 2.663257
H -0.766697 4.431447 2.723322
C -1.378683 2.508886 1.975272
H -0.411380 2.318991 1.505592
C -2.802495 3.951018 3.282685
H -2.960821 4.889766 3.818641
C -3.621849 1.799821 2.546507
H -4.415078 1.050254 2.508362
C -3.820633 3.000664 3.226829
H -4.777282 3.191588 3.718283
C 0.296698 -1.955470 -4.190655

H -0.139686 -0.962679 -4.367985

H -0.002197 -2.629763 -5.009528

H 1.390008 -1.855154 -4.197050

C 0.152778 -3.767230 -0.809212

H 0.822774 -4.320756 -0.146164

C -1.194650 -3.513951 -0.379949

C -2.017792 -2.750696 -1.223910

H -3.020679 -2.469625 -0.902468

C -1.504337 -2.219576 -2.442736

H -2.141538 -1.559006 -3.029829

C -0.193069 -2.503216 -2.892133

C 0.633368 -3.311901 -2.049930

H 1.661091 -3.525403 -2.350020

C 0.905777 -2.455465 3.513939

H 1.614169 -3.054771 4.090046

C -1.691306 -4.014359 0.936941

H -2.132807 -5.016482 0.812629

H -0.874868 -4.092040 1.668365

H -2.459965 -3.333182 1.328448

C -2.530974 2.578167 -1.428967

H -1.641254 3.191834 -1.250685

H -3.075384 2.944892 -2.309449

H -3.183793 2.565714 -0.547706

P 1.731172 -0.225886 -1.702488

C 1.711813 1.471952 -0.969359

C 2.072803 1.753982 0.355634

C 1.304207 2.536874 -1.785825

C 2.027243 3.057391 0.849238

C 1.259699 3.837850 -1.286064

H 1.007738 2.336241 -2.816587

C 1.623401 4.110003 0.031678

C 3.263145 -0.830442 -0.865858

C 4.416374 -0.024191 -0.822159

C 3.388495 -2.141562 -0.399789

C 5.615996 -0.509538 -0.313951

H 4.381025 1.000191 -1.198750

C 4.593882 -2.630797 0.102696

H 2.519377 -2.796131 -0.419300

C 5.722053 -1.821756 0.153800

H 6.668010 -2.202881 0.539883

C -4.697715 -0.931062 0.662862

C -5.336581 0.207184 0.114162
C -5.488316 -2.095496 0.831953
C -6.676334 0.189385 -0.228167
H -4.757314 1.111502 -0.064688
C -6.827438 -2.123172 0.487099
H -5.010851 -2.980245 1.257536
C -7.419240 -0.975433 -0.039682
H -7.168221 1.064587 -0.652926
H -7.436393 -3.017844 0.618684
N -8.826143 -0.994108 -0.399710
O -9.438353 -2.030899 -0.236247
O -9.311195 0.028404 -0.841878
C 4.618396 -4.027343 0.654643
C 6.812050 0.400608 -0.218394
F 3.899353 -4.867949 -0.102338
F 4.073278 -4.068649 1.884638
F 5.850041 -4.516981 0.753267
F 7.951183 -0.265907 -0.422488
F 6.895055 0.959205 0.993993
F 6.754708 1.392473 -1.108380
H 1.593171 5.130236 0.416361
H 2.392908 0.948181 1.020369
C 2.439595 3.321090 2.272204
C 0.777768 4.964658 -2.160690
F 0.981223 4.717297 -3.451093
F -0.541907 5.166418 -1.998551
F 1.382263 6.114766 -1.858681
F 2.080115 2.314428 3.074873
F 3.759293 3.468663 2.387202
F 1.871636 4.437050 2.748385

Table S132. Cartesian coordinates of **TS5**
Ru -0.166170 -1.569544 -0.772164
P -2.225797 0.149362 0.918349
O -3.067163 0.167657 -2.444578
S -1.849234 0.912129 -2.088784
N -3.427915 -0.862947 1.052566
H -0.435884 1.064808 -0.291547
C -1.173080 0.297663 -0.573850
C -0.896556 -0.480488 1.961320
O -0.760612 0.960716 -3.075733

C 0.079982 -1.267143 1.316769

H 0.975889 -0.835069 0.114938

C 1.019384 -1.917857 2.131809

H 1.804469 -2.530317 1.688140

C 0.017172 -0.992484 4.135220

H 0.000942 -0.882197 5.221487

C -0.937038 -0.351510 3.350482

H -1.725388 0.247393 3.814662

C -2.610375 1.835293 1.485529

C -1.845938 4.047216 2.108249

H -1.024404 4.744111 2.287679

C -1.568624 2.746982 1.699693

H -0.531859 2.433084 1.561720

C -3.170002 4.446399 2.299427

H -3.390983 5.468708 2.615111

C -3.934195 2.230598 1.702949

H -4.749111 1.519070 1.555826

C -4.209493 3.539019 2.100963

H -5.244874 3.847541 2.262028

C 0.361544 -2.265954 -4.133449

H -0.370953 -1.706963 -4.727989

H 0.711409 -3.135138 -4.710891

H 1.224481 -1.601114 -3.952577

C 0.048574 -3.805205 -0.628746

H 0.679169 -4.353045 0.075282

C -1.298094 -3.486916 -0.262599

C -2.058631 -2.732704 -1.178547

H -3.058447 -2.396401 -0.904578

C -1.524134 -2.353058 -2.446592

H -2.143678 -1.742601 -3.103015

C -0.221781 -2.700493 -2.832026

C 0.575161 -3.404577 -1.872484

H 1.607795 -3.654795 -2.126147

C 0.986959 -1.782555 3.519978

H 1.739637 -2.297560 4.122025

C -1.864693 -3.883329 1.061096

H -2.449789 -4.810321 0.951291

H -1.071157 -4.059030 1.799640

H -2.530304 -3.088282 1.430113

C -2.350211 2.596112 -1.793057

H -1.489489 3.188651 -1.463174

H -2.708268 2.955221 -2.767254

H -3.156953 2.618487 -1.050324

P 1.752959 -0.317439 -1.568872

C 1.776893 1.390647 -0.854375

C 2.012023 1.642553 0.504518

C 1.519766 2.469896 -1.705839

C 1.982211 2.944225 0.998983

H 2.201889 0.815565 1.193398

C 1.495853 3.772219 -1.203243

H 1.311026 2.283641 -2.760798

C 1.728272 4.018142 0.146979

H 1.705475 5.037632 0.534328

C 3.294546 -0.932735 -0.748183

C 4.444064 -0.127566 -0.691172

C 3.404986 -2.247359 -0.294776

C 5.638626 -0.623663 -0.178592

H 4.408864 0.906662 -1.039765

C 4.599024 -2.740321 0.227673

H 2.532406 -2.897879 -0.326136

C 5.728902 -1.934540 0.294058

H 6.662167 -2.314723 0.710621

C -4.745370 -0.935871 0.711385

C -5.341790 -0.263836 -0.383446

C -5.579791 -1.768474 1.500687

C -6.694167 -0.395298 -0.653478

H -4.711303 0.322915 -1.051365

C -6.929538 -1.902464 1.233325

H -5.126036 -2.292501 2.343962

C -7.484945 -1.207969 0.157273

H -7.156834 0.117573 -1.497179

H -7.576206 -2.533179 1.843853

N -8.903634 -1.340546 -0.125229

O -9.557480 -2.072387 0.591524

O -9.356434 -0.711273 -1.060803

C 4.603256 -4.141635 0.769582

C 6.867562 0.247312 -0.169173

C 2.238592 3.188834 2.462451

C 1.156416 4.918315 -2.118737

F 3.905972 -4.216481 1.915816

F 5.829762 -4.588955 1.019004

F 4.018219 -4.993623 -0.083284

F 7.640476 -0.011191 0.888342
F 6.553687 1.543758 -0.138232
F 7.611662 0.044129 -1.260697
F 1.624695 4.301994 2.883499
F 1.791024 2.175420 3.208503
F 3.538560 3.331331 2.720995
F 1.687493 6.064707 -1.692567
F 1.580865 4.702069 -3.360837
F -0.173027 5.102542 -2.180827

Table S133. Cartesian coordinates of **Int4**
Ru -0.180891 -1.642292 -0.563574
P -2.290726 0.128354 1.031533
O -3.103234 -0.028811 -2.287902
S -1.896568 0.751426 -1.984747
N -3.510908 -0.878900 1.102545
H -0.473657 1.009837 -0.194418
C -1.205816 0.227375 -0.442572
C -0.990395 -0.475209 2.113983
O -0.811902 0.771069 -2.981125
C -0.010032 -1.262098 1.481400
H 1.176530 -0.849123 -0.042707
C 0.986557 -1.807029 2.307365
H 1.782385 -2.423561 1.885616
C 0.014771 -0.784251 4.282033
H 0.033963 -0.597346 5.357721
C -0.990400 -0.238520 3.491182
H -1.776553 0.376556 3.937751
C -2.671719 1.825198 1.577465
C -1.924123 4.068126 2.104763
H -1.115093 4.797314 2.184951
C -1.648070 2.778097 1.665255
H -0.622543 2.505691 1.406939
C -3.229523 4.416933 2.456885
H -3.449579 5.431285 2.797903
C -3.972137 2.170801 1.957590
H -4.767735 1.424417 1.915661
C -4.248701 3.469046 2.387362
H -5.267778 3.737043 2.675152
C 0.360499 -2.472318 -3.908256
H 1.457571 -2.423404 -3.876102

H -0.018755 -1.484679 -4.207200
H 0.071113 -3.210604 -4.672931
C 0.073113 -3.857406 -0.322771
H 0.709475 -4.363555 0.406151
C -1.277150 -3.534560 0.020252
C -2.042086 -2.829517 -0.935113
H -3.044918 -2.491350 -0.675504
C -1.514698 -2.511267 -2.222876
H -2.142010 -1.942296 -2.908867
C -0.204679 -2.855554 -2.580460
C 0.595337 -3.500311 -1.583120
H 1.636648 -3.735194 -1.815580
C 0.998909 -1.570659 3.682735
H 1.797100 -2.004696 4.290638
C -1.854029 -3.875209 1.354600
H -2.415668 -4.820351 1.281213
H -1.069031 -3.992937 2.113149
H -2.543673 -3.078167 1.670719
C -2.411528 2.446098 -1.777393
H -1.546682 3.067932 -1.520098
H -2.812821 2.738530 -2.757048
H -3.187718 2.511469 -1.005097
P 1.719360 -0.351230 -1.504336
C 1.741333 1.373087 -0.837041
C 1.979140 1.650009 0.516031
C 1.467811 2.426699 -1.712321
C 1.936701 2.961180 0.982104
H 2.168636 0.835797 1.220380
C 1.438529 3.740055 -1.237901
H 1.245505 2.213639 -2.759743
C 1.673264 4.014266 0.105844
H 1.640521 5.041392 0.471960
C 3.344186 -0.903120 -0.800714
C 4.492242 -0.116921 -0.975615
C 3.488001 -2.152862 -0.201757
C 5.733876 -0.573658 -0.544623
H 4.422267 0.864031 -1.451276
C 4.733160 -2.609219 0.227018
H 2.608216 -2.778787 -0.050241
C 5.868228 -1.826169 0.059012
H 6.844761 -2.184711 0.386296

C -4.786382 -0.922379 0.627578
C -5.603438 -1.999923 1.058631
C -5.372598 0.015039 -0.258898
C -6.917401 -2.126987 0.648186
H -5.165802 -2.728118 1.744122
C -6.687735 -0.104493 -0.670869
H -4.763766 0.824326 -0.657875
C -7.458990 -1.172751 -0.214213
H -7.546619 -2.951683 0.983784
H -7.137004 0.614076 -1.356650
N -8.838895 -1.296940 -0.646394
O -9.281632 -0.438619 -1.384322
O -9.474692 -2.251806 -0.244692
C 2.184691 3.243571 2.440951
C 1.081810 4.861097 -2.177771
C 6.947602 0.308302 -0.681435
C 4.794817 -3.950599 0.902722
F 8.042046 -0.407613 -0.947969
F 7.181738 0.982316 0.448540
F 6.798515 1.206384 -1.656562
F 6.042729 -4.365953 1.096024
F 4.188843 -3.915520 2.098745
F 4.157016 -4.883035 0.181442
F 1.778675 2.230394 3.206981
F 3.479177 3.443418 2.691215
F 1.529303 4.341693 2.837926
F 1.563532 6.030657 -1.757573
F 1.543650 4.639791 -3.405961
F -0.250770 4.996650 -2.274315

Table S134. Cartesian coordinates of **TS6**
Ru -0.245825 -1.741671 -0.378875
P -2.450577 -0.044327 1.167344
O -3.081144 -0.076494 -2.149306
S -1.893094 0.699314 -1.777785
N -3.693824 -1.018209 1.037080
H -0.558846 0.912224 0.094559
C -1.272555 0.133827 -0.220530
C -1.260651 -0.765530 2.304863
O -0.772333 0.750531 -2.733597
C -0.235477 -1.507026 1.685775

H 1.325638 -0.614398 0.631188

C 0.696354 -2.118247 2.543971

H 1.526559 -2.700462 2.133356

C -0.441766 -1.270907 4.512709

H -0.516479 -1.184776 5.598883

C -1.375808 -0.645948 3.691990

H -2.193473 -0.062977 4.125206

C -2.839600 1.614568 1.822949

C -2.146740 3.837330 2.492406

H -1.358647 4.584383 2.604064

C -1.840642 2.588882 1.960211

H -0.809138 2.373274 1.668388

C -3.453291 4.121874 2.893972

H -3.694201 5.103785 3.308134

C -4.140944 1.896764 2.251387

H -4.915107 1.131209 2.168029

C -4.446293 3.151369 2.778839

H -5.466549 3.367700 3.103635

C 0.833811 -2.285278 -3.630479

H 1.890874 -2.123645 -3.374164

H 0.415551 -1.337105 -3.994913

H 0.793929 -3.027713 -4.443668

C 0.005199 -3.943761 -0.252606

H 0.530000 -4.498782 0.527347

C -1.373295 -3.600863 -0.070140

C -1.986904 -2.819384 -1.085351

H -3.009209 -2.466142 -0.943388

C -1.291046 -2.457689 -2.281441

H -1.810041 -1.847925 -3.020344

C 0.062865 -2.765282 -2.443791

C 0.707577 -3.494100 -1.386160

H 1.774634 -3.712597 -1.471718

C 0.593908 -2.003761 3.931637

H 1.337495 -2.492706 4.566882

C -2.135269 -4.019173 1.143718

H -2.695178 -4.943772 0.928861

H -1.462851 -4.207336 1.991435

H -2.848010 -3.227695 1.421071

C -2.409841 2.391611 -1.547110

H -1.545951 3.001879 -1.259960

H -2.788912 2.709596 -2.527475

H -3.199515 2.445452 -0.787986
P 1.760535 -0.293875 -0.712041
C 1.794075 1.521365 -0.484341
C 2.076369 2.088287 0.764949
C 1.482414 2.352484 -1.564427
C 2.039693 3.470874 0.929118
H 2.321947 1.448520 1.615836
C 1.452507 3.736381 -1.388958
H 1.219918 1.915131 -2.528948
C 1.731509 4.301118 -0.147636
H 1.715903 5.384636 -0.019776
C 3.480384 -0.835924 -0.426595
C 4.588092 -0.042077 -0.739707
C 3.689127 -2.126386 0.062551
C 5.876611 -0.540564 -0.555384
H 4.453763 0.975018 -1.114291
C 4.978430 -2.614785 0.247830
H 2.829906 -2.752509 0.314583
C 6.084193 -1.827342 -0.059302
H 7.094571 -2.208113 0.092135
C 5.128353 -4.006987 0.798417
C 7.061516 0.306115 -0.942885
C 2.270159 4.066368 2.292670
C 1.050558 4.621206 -2.540450
F -0.282918 4.750034 -2.596738
F 1.558650 5.847624 -2.417414
F 1.450234 4.121904 -3.706685
F 1.120704 4.132849 2.982650
F 2.753892 5.304835 2.214707
F 3.117206 3.333743 3.012758
F 8.127401 0.029778 -0.190584
F 7.407240 0.094835 -2.215988
F 6.795241 1.607655 -0.814997
F 6.401232 -4.380080 0.891237
F 4.495433 -4.896318 0.021461
F 4.582181 -4.103833 2.015978
C -4.916100 -0.968111 0.438965
C -5.441715 0.132677 -0.282780
C -5.747174 -2.112084 0.559275
C -6.709232 0.099702 -0.833798
H -4.825856 1.018714 -0.428445

C -7.013589 -2.154773 0.006683

H -5.359768 -2.967031 1.116515

C -7.494417 -1.043653 -0.687433

H -7.110866 0.945744 -1.391720

H -7.651874 -3.033564 0.101385

N -8.823794 -1.078555 -1.266516

O -9.473913 -2.097185 -1.133789

O -9.214745 -0.086776 -1.851069

Table S135. 5Cartesian coordinates of **3’**

Ru 0.009128 -1.233507 -1.477659

O 2.083273 -2.137331 1.498861

S 1.020569 -1.136238 1.634015

P 2.406444 0.601322 -0.517592

N 3.622709 -0.309243 -0.984320

C 0.898494 -0.164172 0.167674

H 0.248127 0.671787 0.458849

P -1.891293 -0.036518 -1.149980

C 2.891813 2.031047 0.507279

O -0.338645 -1.601392 1.975390

C 1.933697 2.807600 1.168947

H 0.877193 2.542998 1.109176

C 2.316381 3.922163 1.907652

H 1.559738 4.511846 2.430892

C 4.623153 3.515176 1.314286

H 5.678362 3.791921 1.370076

C 4.241049 2.395405 0.576283

H 4.988962 1.794016 0.054559

C 1.609762 1.276751 -1.981833

C 0.555798 0.485998 -2.482905

C -0.023754 0.917554 -3.689399

H -0.842140 0.343932 -4.136585

C 0.403425 2.081682 -4.331735

H -0.077991 2.391325 -5.263426

C 1.425321 2.861556 -3.787661

H 1.739717 3.784101 -4.279674

C 2.039684 2.448840 -2.608734

H 2.847381 3.040423 -2.169413

H -2.515603 0.410637 -2.346853

C 2.447240 -1.823196 -3.761594

H 2.042806 -1.179421 -4.554047

H 3.002456 -2.650378 -4.232132
H 3.141101 -1.233603 -3.142888
C 1.357917 -2.370215 -2.897944
C 1.621042 -2.799029 -1.581401
H 2.622047 -2.687818 -1.163729
C 0.583774 -3.333612 -0.756742
H 0.837590 -3.638585 0.258190
C -0.739378 -3.438915 -1.209927
C -1.024294 -2.912174 -2.511705
H -2.055485 -2.916711 -2.874178
C -0.001358 -2.416649 -3.347811
H -0.249179 -2.037592 -4.340519
C -3.346677 -0.793117 -0.346902
C -3.189899 -1.432523 0.885059
H -2.194767 -1.505035 1.342711
C -4.300775 -1.988871 1.519009
C -5.557181 -1.943724 0.923622
H -6.419929 -2.383245 1.425668
C -5.703548 -1.326937 -0.318728
C -4.607821 -0.741593 -0.948456
H -4.746367 -0.238046 -1.908061
C -1.758367 1.593923 -0.305961
C -1.923214 1.715607 1.075945
H -2.188179 0.848255 1.685134
C -1.713104 2.947461 1.698521
C -1.343868 4.064719 0.955448
H -1.177707 5.024271 1.446713
C -1.158257 3.935338 -0.419061
C -1.365249 2.712351 -1.050649
H -1.199017 2.631238 -2.126590
C 3.664383 4.275586 1.982193
H 3.968251 5.148773 2.564226
C -1.798861 -4.076802 -0.371213
H -2.801462 -3.718379 -0.639865
H -1.783912 -5.168288 -0.524762
H -1.629673 -3.883074 0.695745
C 1.478335 -0.052252 2.975896
H 2.439023 0.433130 2.767551
H 0.683511 0.687315 3.129110
H 1.559964 -0.712430 3.849812
C 4.629228 -0.936563 -0.314580

C 5.538040 -1.713926 -1.079151
C 4.862558 -0.868962 1.082225
C 6.603384 -2.373623 -0.495723
H 5.378224 -1.771498 -2.157445
C 5.928718 -1.521380 1.672391
H 4.177225 -0.302588 1.711491
C 6.798823 -2.272953 0.882643
H 7.301018 -2.969990 -1.084218
H 6.104660 -1.471087 2.747055
N 7.916378 -2.958850 1.501441
O 8.063125 -2.837367 2.702358
O 8.646116 -3.616353 0.784860
C -0.638908 5.109343 -1.207670
C -1.885742 3.056339 3.190138
C -4.101659 -2.659541 2.853945
C -7.044925 -1.331923 -1.003134
F -1.236543 4.114983 3.681370
F -3.167287 3.173837 3.533815
F -1.408546 1.967725 3.807253
F -1.102643 6.263847 -0.723391
F -0.984077 5.035021 -2.492620
F 0.695521 5.165526 -1.151931
F -3.323265 -3.740101 2.736664
F -3.511974 -1.836558 3.720652
F -5.258853 -3.054928 3.387387
F -8.045613 -1.330287 -0.122863
F -7.193158 -0.268819 -1.796932
F -7.189066 -2.419211 -1.768187

Table S136. Cartesian coordinates of **TS2**
Ru -1.553172 -1.649082 -1.357648
P -1.103680 -0.665452 1.607230
O -4.350151 -1.811911 1.216313
S -4.028920 -0.551392 0.527512
N 0.322698 -0.058677 1.030003
H -2.171216 0.658094 0.034307
C -2.279873 -0.417704 0.250113
C -1.588912 0.371692 3.016205
O -4.685204 -0.261230 -0.750676
C -1.427600 1.755438 2.856014
H -0.952772 2.149608 1.954096

C -1.855890 2.626976 3.850698
H -1.715546 3.702015 3.718856
C -2.595530 0.748241 5.179009
H -3.046709 0.353170 6.091951
C -2.164654 -0.131994 4.186650
H -2.276997 -1.209072 4.326717
C -0.927188 -2.372551 2.179914
C -1.803942 -4.418598 3.117786
H -2.658277 -5.018910 3.439075
C -2.020149 -3.141552 2.604128
H -3.035090 -2.754248 2.489861
C -0.509927 -4.929760 3.222281
H -0.347552 -5.927844 3.635944
C 0.369068 -2.899776 2.254786
H 1.220078 -2.321325 1.892975
C 0.574539 -4.171405 2.784399
H 1.588913 -4.571988 2.842944
C -4.100576 -1.574674 -3.668065
H -4.757513 -1.034363 -2.973507
H -4.716197 -2.255580 -4.279597
H -3.631213 -0.846208 -4.344538
C -0.766296 -3.244673 -2.657892
H 0.252381 -3.318194 -3.045193
C -1.089907 -3.873378 -1.420586
C -2.406065 -3.692260 -0.924590
H -2.685472 -4.109122 0.043662
C -3.367407 -2.947678 -1.660817
H -4.348950 -2.781724 -1.217067
C -3.067709 -2.340271 -2.903991
C -1.726310 -2.476996 -3.368913
H -1.428052 -1.973384 -4.290349
C -2.446739 2.124411 5.011442
H -2.782486 2.808862 5.793772
C -0.108208 -4.758733 -0.722757
H -0.279129 -4.768301 0.360127
H -0.213708 -5.793187 -1.090580
H 0.929508 -4.452218 -0.914941
C -4.538226 0.783857 1.599104
H -4.259852 1.735640 1.129687
H -5.631707 0.690403 1.639938
H -4.100323 0.677382 2.597216

P -0.024899 -0.158475 -1.225139
C -0.416981 1.638701 -1.204592
C 0.279976 2.617716 -0.490110
C -1.600784 1.996968 -1.862536
C -0.211548 3.919704 -0.420009
H 1.190095 2.369546 0.056440
C -2.094593 3.296357 -1.773958
H -2.159371 1.220925 -2.395420
C -1.400670 4.268523 -1.057708
H -1.784788 5.287607 -0.991651
C 1.702613 -0.311648 -1.729937
C 2.454591 0.669282 -2.384321
C 2.303433 -1.526175 -1.388576
C 3.797481 0.432780 -2.669867
H 2.000963 1.624207 -2.659212
C 3.643047 -1.755751 -1.682967
H 1.705231 -2.278248 -0.866460
C 4.400598 -0.777000 -2.321196
H 5.453244 -0.952998 -2.545281
C 1.426180 0.230841 1.808804
C 2.581855 0.784340 1.202563
C 1.501872 -0.009328 3.206323
C 3.717412 1.094893 1.929623
H 2.597509 0.949036 0.128966
C 2.634818 0.300460 3.938606
H 0.655991 -0.447542 3.733480
C 3.740527 0.856128 3.300882
H 4.597436 1.520412 1.446763
H 2.680425 0.116156 5.012171
N 4.930499 1.179525 4.070864
O 4.908478 0.953285 5.263660
O 5.874659 1.655488 3.474528
C 0.492119 4.919502 0.457551
C -3.441398 3.617606 -2.364716
C 4.625421 1.528946 -3.287230
C 4.242615 -3.073672 -1.273111
F 5.654106 1.040387 -3.980539
F 5.125390 2.333400 -2.343514
F 3.898285 2.287528 -4.109526
F 5.525771 -3.171886 -1.610184
F 3.590840 -4.094216 -1.848720

F 4.149231 -3.253854 0.047899
F -3.695889 2.886214 -3.447552
F -3.536476 4.905070 -2.704456
F -4.413468 3.371109 -1.476377
F 0.231160 6.174147 0.092509
F 0.092089 4.788002 1.734057
F 1.812402 4.749040 0.442236

Table S137. Cartesian coordinates of **Int2**
Ru 1.783675 -0.340294 -1.674315
P 1.847815 0.796708 0.994521
O 4.994451 0.461223 0.050866
S 4.355528 -0.822507 0.384353
N 0.180460 0.429766 0.954477
H 2.194786 -1.555015 0.789482
C 2.600168 -0.653112 0.302321
C 2.102149 0.924295 2.807975
O 4.732100 -2.022176 -0.364484
C 1.888100 -0.231877 3.574849
H 1.574558 -1.163488 3.097380
C 2.081738 -0.214103 4.950684
H 1.903495 -1.120068 5.534191
C 2.721594 2.109566 4.828226
H 3.046091 3.031009 5.316884
C 2.517610 2.096547 3.448215
H 2.675802 3.009327 2.870891
C 2.244713 2.424486 0.323457
C 3.745482 4.292418 0.012264
H 4.744448 4.723339 0.110322
C 3.518092 2.991678 0.462065
H 4.336151 2.406269 0.881360
C 2.717117 5.035348 -0.561622
H 2.904024 6.053631 -0.910020
C 1.211724 3.175423 -0.255846
H 0.219846 2.736767 -0.364976
C 1.445845 4.471406 -0.697973
H 0.631093 5.035903 -1.156054
C 1.163869 -2.984891 -3.790072
H 1.600000 -3.777393 -3.166284
H 1.295530 -3.272539 -4.846731
H 0.083819 -2.952521 -3.590189

C 1.763835 0.832568 -3.553947
H 1.220846 1.749229 -3.797288
C 3.100800 0.911110 -3.082317
C 3.742610 -0.319662 -2.789514
H 4.734910 -0.293338 -2.339808
C 3.140886 -1.588287 -3.024946
H 3.690359 -2.489344 -2.752231
C 1.822275 -1.668966 -3.515664
C 1.141547 -0.431957 -3.760442
H 0.103749 -0.459609 -4.102112
C 2.502516 0.958829 5.580792
H 2.656414 0.973592 6.662143
C 3.811413 2.212537 -2.903673
H 4.529541 2.142245 -2.075559
H 4.368295 2.482349 -3.817033
H 3.104484 3.024484 -2.683622
C 4.785144 -1.164347 2.085043
H 4.267722 -2.074126 2.414144
H 5.870679 -1.330203 2.068280
H 4.531644 -0.308041 2.720802
P -0.115937 -0.340541 -0.633784
C -0.768263 -1.979760 -0.135346
C -0.325696 -2.592576 1.040772
C -1.472556 -2.753941 -1.066851
C -0.549595 -3.946454 1.268636
H 0.201471 -2.000154 1.789133
C -1.706156 -4.104918 -0.828469
H -1.823184 -2.305130 -2.000004
C -1.236209 -4.718321 0.334566
H -1.397465 -5.783240 0.503768
C -1.617902 0.557317 -1.191161
C -2.915308 0.280917 -0.756451
C -1.398871 1.660001 -2.018805
C -3.967072 1.125004 -1.111489
H -3.117174 -0.595538 -0.138921
C -2.451385 2.498471 -2.373936
H -0.380877 1.851220 -2.367321
C -3.742265 2.239489 -1.916140
H -4.569499 2.894681 -2.191723
C -0.785176 0.922607 1.855397
C -1.868261 0.113088 2.239983

C -0.722733 2.239266 2.345688
C -2.873773 0.608940 3.057459
H -1.920618 -0.919286 1.899682
C -1.710815 2.732964 3.183119
H 0.100394 2.891782 2.057974
C -2.784425 1.915569 3.522615
H -3.725187 -0.004380 3.352071
H -1.675712 3.752059 3.567896
N -3.836333 2.440626 4.392089
O -3.722346 3.584345 4.777705
O -4.752444 1.698779 4.671674
C -0.067838 -4.543763 2.562073
C -5.337092 0.839538 -0.555399
C -2.502580 -4.900467 -1.828424
C -2.156214 3.716941 -3.204685
F 1.123378 -4.033892 2.915340
F -0.910985 -4.277813 3.564870
F 0.063939 -5.865708 2.487362
F -3.815381 -4.770374 -1.616366
F -2.215462 -6.200941 -1.767201
F -2.265509 -4.488814 -3.079262
F -1.269523 3.449348 -4.167557
F -1.626474 4.693257 -2.450171
F -3.250480 4.211276 -3.778855
F -6.284889 1.535754 -1.181799
F -5.397814 1.152420 0.743508
F -5.640351 -0.456459 -0.660709

Table S138. Cartesian coordinates of **TS3**
Ru -1.358635 -0.433183 -1.655472
P -1.821533 -1.090795 0.561206
O -4.955628 -0.897178 -1.192924
S -4.435927 0.387565 -0.687369
N 0.144144 -0.884954 0.956880
H -2.539356 1.196570 0.304690
C -2.767044 0.255153 -0.219577
C -2.197382 -0.649032 2.329868
O -4.607325 1.567831 -1.546387
C -1.708713 0.538022 2.888354
H -1.012185 1.161097 2.325583
C -2.084531 0.933839 4.167642

H -1.676378 1.857916 4.584218
C -3.458991 -1.034540 4.365058
H -4.142031 -1.662027 4.942940
C -3.082302 -1.430821 3.079873
H -3.472706 -2.365095 2.669470
C -2.289611 -2.845465 0.488269
C -3.825682 -4.651196 0.024304
H -4.822299 -4.986236 -0.273276
C -3.565522 -3.284019 0.110362
H -4.339547 -2.557980 -0.148570
C -2.831520 -5.585553 0.314737
H -3.045983 -6.654739 0.249695
C -1.284101 -3.784479 0.762444
H -0.278980 -3.452146 1.027502
C -1.559558 -5.147485 0.680828
H -0.770102 -5.870144 0.899992
C -2.091061 1.940199 -4.064720
H -2.901735 2.320687 -3.426149
H -2.416715 2.023164 -5.114740
H -1.209477 2.585013 -3.939135
C -0.208858 -1.370102 -3.353267
H 0.821745 -1.728262 -3.338087
C -1.254764 -2.258628 -3.007706
C -2.582348 -1.735759 -3.022057
H -3.424696 -2.357459 -2.717316
C -2.827913 -0.390042 -3.375789
H -3.850804 -0.015832 -3.322548
C -1.785830 0.518206 -3.711655
C -0.468910 -0.002127 -3.658702
H 0.376649 0.662880 -3.852535
C -2.962026 0.145386 4.913266
H -3.252815 0.448927 5.921469
C -0.990099 -3.700954 -2.719377
H -1.692322 -4.091987 -1.972195
H -1.112635 -4.294506 -3.640247
H 0.035262 -3.856137 -2.356609
C -5.303120 0.728372 0.835145
H -4.932066 1.672227 1.253040
H -6.363391 0.815927 0.563620
H -5.139160 -0.099942 1.536171
P 0.378910 0.151807 -0.373959

C 0.441389 1.897751 0.203404
C 1.015642 2.343862 1.399987
C -0.279198 2.801284 -0.581383
C 0.848458 3.667403 1.800447
H 1.569218 1.662149 2.046771
C -0.400087 4.135959 -0.197941
H -0.781870 2.427842 -1.480144
C 0.154429 4.574889 0.999702
H 0.040254 5.613411 1.312826
C 2.030171 -0.179039 -1.072313
C 2.847385 0.822536 -1.595013
C 2.405213 -1.518583 -1.202823
C 4.028893 0.479633 -2.254704
H 2.569615 1.874088 -1.487528
C 3.582898 -1.850854 -1.863786
H 1.759873 -2.299277 -0.790488
C 4.402192 -0.854765 -2.393569
H 5.326118 -1.118211 -2.909052
C 1.350709 4.089477 3.155562
C -1.179436 5.066658 -1.089936
C 4.866949 1.572680 -2.864642
C 3.916797 -3.308477 -2.035567
F -0.672764 5.074461 -2.328469
F -2.452469 4.682694 -1.189085
F -1.163627 6.320010 -0.637053
F 0.443561 3.808980 4.102309
F 1.588241 5.399391 3.211251
F 2.473278 3.450973 3.485024
F 4.334715 1.993738 -4.017012
F 4.942668 2.631944 -2.056433
F 6.107610 1.161688 -3.122416
F 2.993218 -3.911850 -2.800330
F 3.929252 -3.947496 -0.865755
F 5.099919 -3.488681 -2.617267
C 0.965648 -1.132852 2.046439
C 2.294905 -0.655634 2.118574
C 0.499426 -1.932493 3.114130
C 3.105668 -0.939300 3.206170
H 2.708362 -0.064658 1.301781
C 1.305283 -2.213333 4.203943
H -0.514959 -2.325013 3.091782

C 2.603922 -1.712548 4.247977
H 4.129647 -0.569656 3.264293
H 0.943134 -2.820993 5.033302
N 3.452017 -2.007790 5.395665
O 2.980699 -2.690101 6.280988
O 4.576838 -1.553130 5.394201

Table S139. Cartesian coordinates of **4'**
C 4.543279 0.088011 0.838255
P -0.434815 -0.192808 0.101355
O 3.816449 -2.891569 2.139054
Ru 1.446454 -1.577368 -0.288743
P 2.727193 0.049368 0.679885
O 1.636518 -3.090063 3.391887
S 2.677627 -2.239535 2.805869
C 1.882797 -1.096647 1.746143
H 0.976527 -0.697458 2.244445
C -0.920802 2.545250 -0.466858
H -1.409585 2.595080 0.506302
C -0.811213 3.697470 -1.238527
C -0.151783 3.676928 -2.468578
H -0.054842 4.586862 -3.062800
C 0.415927 2.487846 -2.908219
C 0.310599 1.329872 -2.137673
H 0.805494 0.411562 -2.457668
C -0.370609 1.342462 -0.923917
C -2.355168 -0.899071 -1.901174
H -1.888176 -0.164711 -2.562253
C -3.388630 -1.702810 -2.387506
C -4.012553 -2.631531 -1.561252
H -4.819435 -3.257052 -1.943552
C -3.595008 -2.748068 -0.234309
C -2.559947 -1.957592 0.255109
H -2.226641 -2.056924 1.292146
C 2.511331 2.704389 -0.068370
H 3.004008 2.387371 -0.991764
C 5.325882 -1.022856 0.501482
H 4.847012 -1.936600 0.161825
C 7.326913 0.165006 1.148777
H 8.412524 0.194833 1.267407
C 6.707987 -0.982981 0.657136

H 7.303562 -1.861311 0.399053
C 6.554982 1.272816 1.493576
H 7.030292 2.174751 1.885734
C 5.170890 1.238147 1.341278
H 4.577773 2.112091 1.615345
C 2.226621 1.770257 0.938136
C 2.156355 4.038454 0.102715
H 2.372549 4.764162 -0.684096
C 1.502383 4.444331 1.267608
H 1.205256 5.488057 1.388390
C 1.208510 3.515311 2.263095
H 0.681059 3.825321 3.167735
C 1.574965 2.179809 2.104731
H 1.333218 1.451870 2.878603
C 1.030917 -3.815367 -0.299745
C 0.194140 -3.194248 -1.246400
H -0.883086 -3.334030 -1.164259
C 0.704328 -2.347381 -2.274177
H 0.005296 -1.879668 -2.970188
C 2.088797 -2.114492 -2.404019
C 2.943925 -2.766777 -1.461595
H 4.018675 -2.595375 -1.531679
C 2.437687 -3.578988 -0.430985
H 3.120209 -3.983468 0.317506
N -0.700027 0.162501 1.656667
C -1.918512 -1.041983 -0.586665
C 0.486846 -4.705176 0.770156
H 0.733106 -5.754700 0.542113
H 0.919289 -4.450041 1.748464
H -0.605878 -4.618717 0.836267
C 2.664134 -1.290616 -3.513468
H 3.425010 -0.590714 -3.141295
H 3.146405 -1.949047 -4.253846
H 1.892043 -0.715926 -4.040429
C 3.363532 -1.250800 4.122363
H 2.551621 -0.699677 4.613022
H 3.823504 -1.961758 4.821739
H 4.119545 -0.574582 3.702051
C -1.782149 0.706411 2.273069
C -1.693329 0.930785 3.674670
C -3.004649 1.074710 1.645703

C -2.727290 1.497619 4.392073
H -0.773243 0.627782 4.178683
C -4.045708 1.641064 2.361659
H -3.132480 0.914002 0.574039
C -3.905159 1.857205 3.731211
H -2.653976 1.671198 5.466025
H -4.979878 1.928106 1.877954
N -4.992110 2.459404 4.477509
O -6.004210 2.751820 3.870736
O -4.829639 2.639819 5.668910
C 1.232367 2.434883 -4.166995
C -1.384553 5.001656 -0.751825
C -3.772247 -1.567024 -3.835731
C -4.204734 -3.788197 0.667800
F -2.749240 -1.926276 -4.627073
F -4.814739 -2.329618 -4.156730
F -4.074025 -0.304546 -4.144475
F -5.360057 -4.248454 0.188617
F -3.379745 -4.840542 0.793970
F -4.422753 -3.312227 1.890335
F 0.862555 1.410887 -4.948775
F 2.532878 2.247941 -3.884858
F 1.136258 3.549433 -4.885698
F -2.453807 5.360522 -1.465568
F -0.487062 5.991910 -0.871414
F -1.749011 4.942659 0.526634

4.2.8 Conc and OxAdd

Table S140. SCF energies, enthalpy and free energy corrections and barriers

	E_{SCF}/E_H	corrH/E_H	corrG/E_H	ΔH/kJ/mol	ΔG/kJ/mol
**Conc**	-3103.681438	0.757451	0.628935	7.517867	78.117562
**OxAdd**	-3103.678998	0.758585	0.627392	16.90093	80.47216

Table S141. Cartesian coordinates of **Conc**

Ru -0.268960 -1.454214 -0.502966
Si 1.360329 0.665344 -2.832860
P 1.657478 0.347112 0.265466
O 1.378361 -0.919999 3.484155
S 0.133051 -0.553467 2.768842
N 1.093613 0.031655 -1.237338
H -1.406510 0.061330 -0.002281

C 0.312394 -0.282902 1.115408

C 2.091260 2.089772 0.568934

O -1.028185 -1.447907 3.002798

C 1.051946 3.002280 0.787627

H 0.019216 2.645811 0.808797

C 1.336388 4.353067 0.969747

H 0.519300 5.056247 1.141498

C 3.694866 3.898407 0.705323

H 4.729450 4.248215 0.673502

C 3.414855 2.544922 0.529937

H 4.228993 1.836073 0.362708

C 3.237146 -0.535158 0.531504

C 4.985017 -1.324826 2.003551

H 5.425989 -1.374075 3.001950

C 3.819296 -0.589863 1.804417

H 3.331583 -0.103936 2.650779

C 5.579303 -2.009248 0.942092

H 6.492243 -2.587130 1.106161

C 1.714909 2.513097 -2.770929

H 0.930788 3.060559 -2.227678

H 1.781723 2.917728 -3.794033

H 2.669304 2.721268 -2.263514

C 2.810963 -0.146401 -3.736350

H 3.783107 0.099980 -3.282151

H 2.835093 0.204519 -4.781345

H 2.711639 -1.244255 -3.760580

C 3.834656 -1.223086 -0.528031

H 3.368914 -1.183345 -1.512281

C 5.002918 -1.957381 -0.325947

H 5.462585 -2.490259 -1.162200

C -0.147982 0.330129 -3.907500

H -0.084840 0.921853 -4.835056

H -1.093845 0.566717 -3.398527

H -0.191869 -0.731013 -4.197033

C 1.590550 -3.558665 1.228773

H 1.439305 -2.934783 2.120981

H 2.584666 -3.334076 0.821429

H 1.572494 -4.616447 1.537806

C -1.841748 -2.973945 -0.379069

H -2.869426 -2.803877 -0.051772

C -1.531879 -2.863430 -1.778885

C -0.182857 -2.974328 -2.157175
H 0.102831 -2.818096 -3.198567
C 0.829575 -3.159161 -1.170635
H 1.879116 -3.127142 -1.467128
C 0.521426 -3.326139 0.210600
C -0.856216 -3.240544 0.590838
H -1.119872 -3.223687 1.649577
C 2.656367 4.802104 0.926836
H 2.877341 5.862847 1.069441
C -2.616842 -2.622965 -2.777949
H -3.075937 -3.582234 -3.068792
H -3.406738 -1.984453 -2.357840
H -2.228920 -2.135937 -3.682356
C -0.333654 1.019732 3.486682
H -1.272315 1.356979 3.031317
H -0.455523 0.840884 4.563608
H 0.469412 1.745363 3.304653
P -2.468849 0.409283 -0.982393
C -3.851487 -0.121698 0.099471
C -3.661237 -0.658040 1.381182
C -5.153901 -0.005738 -0.407339
C -4.761287 -1.063544 2.137211
H -2.657190 -0.782776 1.801505
C -6.249359 -0.401302 0.357190
H -5.309954 0.400832 -1.411221
C -6.054336 -0.933764 1.631758
H -4.594784 -1.482298 3.132805
H -7.259521 -0.300882 -0.047534
H -6.912405 -1.251402 2.229291
C -2.342991 2.199469 -0.567647
C -2.730184 2.724321 0.672781
C -1.825816 3.065341 -1.539207
C -2.595548 4.085872 0.934953
H -3.153074 2.061552 1.431749
C -1.684778 4.426526 -1.273988
H -1.533165 2.668904 -2.514195
C -2.072293 4.939608 -0.037453
H -2.903954 4.483767 1.904956
H -1.275158 5.089114 -2.040102
H -1.972029 6.008277 0.168117

Table S142. Cartesian coordinates of **Conc**

Ru -0.270216 -0.988830 -0.813053
Si 0.905516 2.409501 -2.049652
P 1.483073 0.725001 0.520860
O 1.190681 -1.086873 3.333772
S 0.060440 -1.362763 2.429191
N 1.020845 1.064495 -0.968250
H -1.002354 -2.190621 -0.157651
C 0.386489 -0.631590 0.872176
C 1.268546 2.025943 1.767448
O -0.387230 -2.752327 2.271677
C 0.050260 2.717544 1.763950
H -0.689106 2.502411 0.989578
C -0.211887 3.672875 2.740538
H -1.160551 4.215177 2.727976
C 1.948575 3.244445 3.738006
H 2.689708 3.447994 4.514265
C 2.215924 2.286252 2.761449
H 3.158483 1.734726 2.774199
C 3.216691 0.179449 0.619476
C 4.962704 -1.252045 1.477712
H 5.283162 -2.042969 2.159980
C 3.634876 -0.828266 1.496594
H 2.922108 -1.270203 2.196786
C 5.875316 -0.676548 0.594514
H 6.914241 -1.015468 0.582162
C -0.109074 3.848749 -1.383929
H -1.112999 3.541296 -1.062141
H -0.228360 4.609561 -2.172808
H 0.395574 4.327867 -0.530761
C 2.584108 3.168798 -2.484430
H 3.073948 3.593452 -1.593011
H 2.434234 3.995809 -3.197859
H 3.277496 2.453281 -2.952518
C 4.133647 0.756444 -0.265452
H 3.802759 1.527789 -0.960779
C 5.460140 0.332537 -0.274318
H 6.170039 0.787643 -0.969189
C 0.126499 1.759275 -3.634607
H -0.758234 1.148160 -3.400984
H 0.833940 1.125849 -4.190907

H -0.184409 2.582866 -4.296560
C 0.256179 -4.886466 -0.200471
H -0.271369 -4.437766 0.658082
H 0.949744 -5.643374 0.191111
H -0.489530 -5.388998 -0.835996
C 0.906846 -1.916898 -2.538695
H 0.403391 -1.426188 -3.378268
C 2.321828 -1.736031 -2.393052
C 2.990918 -2.553042 -1.512378
H 4.067101 -2.421343 -1.373743
C 2.324177 -3.578470 -0.794136
H 2.902827 -4.208793 -0.113853
C 0.979928 -3.814790 -0.947347
C 0.239445 -2.953101 -1.833925
H -0.739006 -3.296399 -2.180191
C 0.738999 3.937655 3.728144
H 0.534504 4.687859 4.495857
C 3.020710 -0.695600 -3.204827
H 2.777456 -0.792136 -4.275289
H 2.700657 0.304175 -2.880591
H 4.110558 -0.762280 -3.088030
C -1.325987 -0.440436 3.063823
H -2.189500 -0.600193 2.406419
H -1.513072 -0.853806 4.064528
H -1.042607 0.617959 3.126205
P -2.386508 -0.460942 -1.054339
C -3.698683 -1.293302 -0.089971
C -3.443500 -2.370092 0.772327
C -5.029865 -0.879095 -0.273729
C -4.491990 -3.013297 1.428552
H -2.415566 -2.688059 0.965289
C -6.072151 -1.526773 0.381737
H -5.251197 -0.039691 -0.937714
C -5.807971 -2.598501 1.235358
H -4.268191 -3.840196 2.106914
H -7.100069 -1.191079 0.223820
H -6.626807 -3.103628 1.753200
C -2.938861 1.283107 -0.937391
C -3.159363 1.899325 0.303631
C -3.203957 2.005008 -2.107183
C -3.621751 3.210407 0.370047

H -2.974814 1.338919 1.222788
C -3.677001 3.315903 -2.040445
H -3.048951 1.530436 -3.079101
C -3.883120 3.921516 -0.803212
H -3.786694 3.679768 1.343275
H -3.882498 3.866200 -2.961562
H -4.248674 4.949607 -0.750086

4.2.9 Optimizations of the full structures of 11b and 11b-anti

Table S143. SCF energies, enthalpy and free energy corrections and barriers

	E_{SCF}/E_H	corrH/E_H	corrG/E_H	ΔH/kJ/mol	ΔG/kJ/mol
**11b**	-4748.19761	0.896054	0.718562	-	-
**11b-anti**	-4748.18813	0.895996	0.895996	24.73071347	28.63483197

Table S144. Cartesian coordinates of **11b**

Ru 0.468456 0.697916 -1.322195
N -0.394726 2.133280 0.026066
F 6.021210 1.321740 1.719926
H -1.615360 -0.881242 -0.332208
P -1.856163 1.424926 0.204819
O -2.108075 -1.496061 -2.904633
S -2.599349 -0.252888 -2.295216
C -1.556438 0.052097 -0.907662
C -2.176979 0.723879 1.841746
N 1.929427 6.225652 3.113105
F 5.938609 1.560857 -0.413845
O -2.739899 0.953363 -3.120967
P 1.008932 -0.402679 0.772303
O 3.141771 6.270719 3.155486
F 7.350333 0.185263 0.458078
C -3.232679 -0.189570 1.978569
H -3.868748 -0.435521 1.124125
C -3.458776 -0.810133 3.202747
H -4.264840 -1.539542 3.300139
F 6.025591 -4.301216 -0.584117
O 1.188370 7.001097 3.682608

C -2.637738 -0.519181 4.292973

H -2.805817 -1.020265 5.248537

F 4.173473 -4.985015 0.282475

C -1.588059 0.387079 4.156504

H -0.936055 0.603176 5.005531

F 4.210857 -4.275246 -1.745532

C -1.345307 1.004773 2.930448

H -0.499026 1.682559 2.818321

F -2.609702 -4.674960 -1.509864

C -3.231775 2.537240 -0.179361

F -1.211898 -6.173858 -0.869648

C -4.339287 2.676256 0.665546

H -4.403174 2.097861 1.588830

F -0.579443 -4.626460 -2.229873

C -5.362312 3.558350 0.326724

H -6.225053 3.666033 0.987762

C -5.282180 4.302131 -0.849961

H -6.086798 4.992356 -1.114488

C -4.171493 4.175522 -1.685570

H -4.104948 4.762161 -2.604200

C -4.199760 -0.625992 -1.602068

C -3.143349 3.300473 -1.351733

H -2.281023 3.190113 -2.008250

C -5.624082 -2.164720 -0.435637

H -5.797844 -3.148917 0.005031

C -4.403278 -1.889765 -1.045195

H -3.619554 -2.646528 -1.103603

C -6.624286 -1.190335 -0.399568

H -7.580494 -1.411039 0.080644

C -6.414613 0.056472 -0.987853

H -7.203900 0.811244 -0.973721

C -5.196100 0.346657 -1.599742

H -5.010045 1.312090 -2.073196

C 0.146261 3.135336 0.793710

C -0.649329 4.089229 1.473131

H -1.737023 4.043393 1.394243

C -0.068025 5.092748 2.229109

H -0.671994 5.834898 2.751558

C 1.320875 5.167496 2.322461

C 2.133837 4.239852 1.671812

H 3.216462 4.317495 1.778127

C 1.548347 3.238601 0.917602
H 2.156237 2.490325 0.411047
C 1.240789 -1.998776 -3.344595
H 1.827720 -2.592528 -2.630426
H 1.673842 -2.169423 -4.344010
H 0.204393 -2.361107 -3.364370
C 1.289943 -0.539804 -3.020659
C 0.294154 0.356099 -3.496692
H -0.571452 -0.057170 -4.013298
C 0.385294 1.757388 -3.327065
H -0.416087 2.385425 -3.712705
C 1.461644 2.309441 -2.590378
C 2.451517 1.429643 -2.068496
H 3.295760 1.826087 -1.501444
C 2.349246 0.033428 -2.276609
H 3.106518 -0.623517 -1.848863
C 1.638813 3.801060 -2.428612
H 2.160676 3.960336 -1.472352
C 2.555881 4.296398 -3.551163
H 2.086077 4.144797 -4.535934
H 3.519257 3.765211 -3.552057
H 2.759193 5.371413 -3.432504
C 0.338026 4.588787 -2.384693
H -0.328599 4.209590 -1.599535
H -0.196087 4.551869 -3.347432
H 0.547338 5.646720 -2.169268
C 2.717493 -1.037187 0.570053
C 3.750050 -0.102703 0.744384
H 3.504420 0.906484 1.085363
C 5.073562 -0.429907 0.470306
C 5.419334 -1.720024 0.066947
H 6.456075 -1.979340 -0.145865
C 4.408164 -2.670459 -0.048293
C 3.076317 -2.339930 0.204553
H 2.307231 -3.106104 0.086590
C 6.109148 0.655327 0.566973
C 4.715096 -4.065387 -0.519592
C 0.103205 -2.013242 0.909337
C -0.278472 -2.423682 2.192329
H -0.025586 -1.798595 3.050670
C -1.313224 -4.418749 1.297537

H -1.845133 -5.360270 1.447832
C -0.943380 -4.015904 0.015879
C -0.253797 -2.819454 -0.178521
H -0.000751 -2.499512 -1.189311
C -1.328588 -4.874856 -1.157945
C -0.986002 -3.609506 2.383265
C -1.438401 -4.025655 3.755619
F -0.867155 -3.300654 4.718459
F -2.767531 -3.885328 3.892079
F -1.163878 -5.309934 3.994987

Table S144. Cartesian coordinates of **11b-anti**
Ru 0.620644 -0.484325 -1.325328
N 0.455061 1.535056 -0.603932
F -5.510828 -1.125237 -1.483897
H 3.204839 -0.054734 -0.807471
P 1.883529 1.593234 0.191508
O 3.303125 -2.458173 0.338787
S 2.978586 -1.237571 1.092601
C 2.351774 -0.108145 -0.117764
C 3.087931 2.590167 -0.727118
N -3.607140 5.381668 -1.139370
F -5.130970 0.763448 -2.441532
O 2.165017 -1.333719 2.300530
P -0.536442 -0.857410 0.770908
O -4.628245 5.021124 -1.690466
F -6.614476 0.630122 -0.887818
C 4.429935 2.628541 -0.323293
H 4.744005 2.147718 0.606653
C 5.371757 3.271181 -1.120782
H 6.417758 3.294876 -0.807172
F -4.150705 4.204563 1.651733
O -3.435373 6.480691 -0.654031
C 4.980949 3.875610 -2.317101
H 5.723373 4.378420 -2.941245
F -4.232134 2.923704 3.375007
C 3.646722 3.837015 -2.718336
H 3.340974 4.310563 -3.653887
F -2.332729 3.645178 2.670694
C 2.697671 3.189604 -1.928252

H 1.652960 3.139026 -2.242683
F -4.609663 -4.154783 2.310177
C 1.867066 2.197354 1.896092
F -4.034836 -6.182462 1.852574
C 2.498320 3.401722 2.240019
H 3.024821 3.986566 1.483075
F -4.692866 -4.864824 0.283481
C 2.448690 3.859754 3.553198
H 2.938370 4.799522 3.817647
C 1.768009 3.124498 4.523908
H 1.728014 3.487904 5.553565
C 1.133807 1.930938 4.180985
H 0.599251 1.353761 4.938604
C 4.530206 -0.478514 1.556636
C 1.178432 1.461127 2.871120
H 0.695224 0.516125 2.598409
C 6.814630 0.052154 1.042299
H 7.692950 -0.054227 0.401691
C 5.639972 -0.624899 0.724904
H 5.577860 -1.269916 -0.154051
C 6.871472 0.857544 2.181764
H 7.794567 1.388100 2.426741
C 5.760208 0.975155 3.017377
H 5.811212 1.592781 3.916706
C 4.579601 0.299718 2.711605
H 3.699762 0.369780 3.353455
C -0.486445 2.520657 -0.762520
C -0.386135 3.790955 -0.149030
H 0.473751 4.030043 0.477990
C -1.393416 4.730973 -0.289872
H -1.339197 5.700987 0.204154
C -2.518660 4.424628 -1.050826
C -2.634731 3.192795 -1.694250
H -3.534557 2.973822 -2.269115
C -1.623205 2.262272 -1.558038
H -1.713671 1.278438 -2.011459
C -2.303544 -1.170850 -3.246198
H -2.826460 -1.931124 -2.652451
H -2.473466 -1.416193 -4.307165
H -2.776914 -0.197080 -3.060625
C -0.832906 -1.169480 -2.973695

C 0.002593 -0.120920 -3.429342
H -0.458014 0.782541 -3.836640
C 1.421148 -0.222895 -3.432490
H 2.017546 0.598759 -3.827747
C 2.045042 -1.355821 -2.871156
C 1.224751 -2.363708 -2.275201
H 1.689172 -3.208890 -1.766223
C -0.183891 -2.258385 -2.321973
H -0.787229 -3.039661 -1.856016
C 3.543959 -1.544286 -2.862016
H 3.792633 -1.897827 -1.846956
C 3.909222 -2.667212 -3.837259
H 3.647238 -2.391829 -4.871188
H 3.387384 -3.603239 -3.591596
H 4.990869 -2.866530 -3.801806
C 4.341998 -0.282597 -3.167141
H 4.075181 0.565366 -2.517785
H 4.199539 0.043507 -4.209650
H 5.416690 -0.476136 -3.033415
C -2.151082 0.027166 0.670073
C -3.224240 -0.309607 -0.159709
H -3.176570 -1.212978 -0.764846
C -4.351456 0.504917 -0.243492
C -4.451073 1.664997 0.522481
H -5.323433 2.314814 0.435740
C -3.406927 1.988263 1.385287
C -2.273462 1.181854 1.455780
H -1.457679 1.467333 2.121766
C -5.414844 0.187318 -1.255352
C -3.527391 3.197971 2.271335
C -1.018618 -2.618641 0.832276
C 0.005006 -3.569684 0.675579
H 1.030593 -3.238578 0.495205
C -1.553204 -5.398780 0.987526
H -1.762400 -6.467244 1.030969
C -2.564734 -4.464281 1.190030
C -2.304843 -3.094979 1.123248
H -3.123320 -2.397119 1.307549
C -3.979019 -4.921833 1.416590
C -0.262868 -4.932250 0.737326
C 0.873424 -5.889548 0.506400

F 1.834633 -5.753811 1.419087

F 0.472484 -7.162929 0.527549

F 1.443986 -5.675971 -0.693426

5. References

[1] K.-S. Feichtner, V. H. Gessner, *Inorganics* **2016**, *4*, 40.

[2] A. Schmidt, A. R. Nödling, G. Hilt, Angew. Chem. Int. Ed. 2015, 54, 801.

[3] C. A. Busacca, J. C. Lorenz, N. Grinberg, N. Haddad, M. Hrapchak, B. Latli, H. Lee, P. Sabila, A. Saha, M. Sarvestani, S. Shen, R. Varsolona, X. Wei, C. H. Senanayake, *Org. Lett*. **2005**, *7*, 4277.

[4] a) G. Sheldrick, Acta Crystallogr., Sect. A: Found. Crystallogr. **2008**, *A64*, 112−122. b) G. Sheldrick, Acta Cryst. **2015**, *A71*, 3. c) CrysAlisPro, Agilent Technologies, Version 1.171.36.24 (release 03-12-2012 CrysAlis171 .NET) (compiled Dec 3 2012, 18:21:49). d) CrysAlisPro 1.171.38.43 (Rigaku OD, 2015).

[5] *GaussView, Version 3.0;* Gaussian, Inc.: Pittburgh PA, 2000.

[6] Gaussian 09, Revision E.01, M. J. Frisch, G. W. Trucks, H. B. Schlegel, G. E. Scuseria, M. A. Robb, J. R. Cheeseman, G. Scalmani, V. Barone, B. Mennucci, G. A. Petersson, H. Nakatsuji, M. Caricato, X. Li, H. P. Hratchian, A. F. Izmaylov, J. Bloino, G. Zheng, J. L. Sonnenberg, M. Hada, M. Ehara, K. Toyota, R. Fukuda, J. Hasegawa, M. Ishida, T. Nakajima, Y. Honda, O. Kitao, H. Nakai, T. Vreven, J. A. Montgomery, Jr., J. E. Peralta, F. Ogliaro, M. Bearpark, J. J. Heyd, E. Brothers, K. N. Kudin, V. N. Staroverov, T. Keith, R. Kobayashi, J. Normand, K. Raghavachari, A. Rendell, J. C. Burant, S. S. Iyengar, J. Tomasi, M. Cossi, N. Rega, J. M. Millam, M. Klene, J. E. Knox, J. B. Cross, V. Bakken, C. Adamo, J. Jaramillo, R. Gomperts, R. E. Stratmann, O. Yazyev, A. J. Austin, R. Cammi, C. Pomelli, J. W. Ochterski, R. L. Martin, K. Morokuma, V. G. Zakrzewski, G. A. Voth, P. Salvador, J. J. Dannenberg, S. Dapprich, A. D. Daniels, O. Farkas, J. B. Foresman, J. V. Ortiz, J. Cioslowski, D. J. Fox, Gaussian, Inc., Wallingford CT, 2013.

[7] a) P. Hohenberg, W. Kohn, *Phys. Rev.* **1964**, *136*, B864; b) W. Kohn, L. J. Sham, *Phys. Rev.* **1965**, *140*, A1133.

[8] C. Adamo, V. Barone, *J. Chem. Phys.* **1999**, *110*, 6158.

[9] a) S. Grimme, J. Antony, S. Ehrlich, H. Krieg, *J. Chem. Phys.* **2010**, *132*, 154104; b) S. Grimme, S. Ehrlich, L. Goerigk, *J. Comput. Chem.* **2011**, *32*, 1456; c) D. G. A. Smith, L. A. Burns, K. Patkowski, C. D. Sherrill, *J. Phys. Chem. Lett.* **2016**, *7*, 2197.

[10] a) P. J. Hay, W. R. Wadt, *J. Chem. Phys*. **1985**, *82*, 270; b) P. J. Hay, W. R. Wadt, *J. Chem. Phys.* **1985**, *82*, 284; c) P. J. Hay, W. R. Wadt, *J.Chem. Phys.* **1985**, *82*, 299.

[11] a) D. Andrae, U. Häußermann, M. Dolg, H. Stoll, H. Preuß, *Theor. Chim. Acta* **1990**, *77*, 123. b) F. Weigend, R. Ahlrichs, *Phys. Chem. Chem. Phys.* **2005**, *7*, 3297.

[12] P. Deglmann, F. Furche, R. Ahlrichs, *Chem. Phys. Lett.* **2002**, *362*, 511.
